# Molecular Regulators for the Wnt/β-Catenin Signalling Pathway for Bone Remodelling in Periodontitis: A Scoping Review

**DOI:** 10.3390/ijms27209117

**Published:** 2026-10-14

**Authors:** Hasif Amir Maeraj, Shivaroobini Rajoo, Shahida Mohd-Said, Nurrul Shaqinah Nasruddin, Izatus Shima Taib, Sok Kuan Wong, Putri Ayu Jayusman

**Affiliations:** 1 Department of Craniofacial Diagnostics and Biosciences, Faculty of Dentistry, Universiti Kebangsaan Malaysia, Kuala Lumpur 50300, Malaysia; p167105@siswa.ukm.edu.my (H.A.M.);; 2 Department of Restorative Dentistry, Faculty of Dentistry, Universiti Kebangsaan Malaysia, Kuala Lumpur 50300, Malaysiashahidams@ukm.edu.my (S.M.-S.); 3 Centre for Diagnostics, Therapeutics, and Investigative Studies, Faculty of Health Sciences, Universiti Kebangsaan Malaysia, Jalan Raja Muda Abdul Aziz, Kuala Lumpur 50300, Malaysia; izatusshima@ukm.edu.my; 4 Department of Pharmacology, Faculty of Medicine, Universiti Kebangsaan Malaysia, Jalan Yaacob Latif, Bandar Tun Razak, Cheras, Kuala Lumpur 56000, Malaysia; sokkuan@ukm.edu.my

**Keywords:** Wnt/β-catenin signalling pathway, periodontitis, bone remodelling

## Abstract

Periodontitis is a chronic inflammatory disease that destroys the periodontal ligament, leading to alveolar bone loss and ultimately tooth loss. The Wnt/β-catenin signalling pathway is an important molecular signalling pathway that controls the expression of bone remodelling-related genes. Manipulating this pathway is a recurring theme in recent studies, suggesting a viable therapeutic target. A scoping review was conducted according to PRISMA guidelines to identify the latest molecular regulators used in manipulating the signalling pathway between 2007 and 2025 from databases such as Web of Science, Ovid Medline, and Scopus. Only primary articles relevant to the signalling pathway, periodontitis, and bone homeostasis were selected. A total of 22 articles were identified, using various molecules ranging from plant extracts such as field horsetail to pre-existing drugs. The molecules function by altering the activities of targets within the signalling pathway such as β-catenin and GSK-3β phosphorylation, ultimately increasing osteoblast differentiation for bone growth that was reduced in periodontitis cases. The Wnt/β-catenin signalling pathway is a favoured target for potential therapeutic interventions for periodontitis. However, most studies are limited to in vitro methods and requires further description on the relationships between periodontitis and the signalling pathway.

## 1. Introduction

Periodontitis is a chronic disease caused by inflammation that leads to the inability of the periodontal ligament to be repaired. This leads to alveolar bone loss and ultimately tooth loss if left untreated. Even though the disease is mainly caused by microorganism infection, other factors are associated with the disease that exacerbate the disease, such as immune reactions, environmental factors, and genetics [1]. The threat of this disease is also severe, as periodontitis leads to irreversible damage to the supporting tissue and tooth loss. The inflammatory effects from this disease lead to a decrease in bone tissue precursor cells, which are controlled by many signalling pathways, such as the Wnt/β-catenin signalling pathway [1]. The Wnt/β-catenin signalling pathway is responsible for controlling many processes aside from early tooth generation and bone regeneration. This is seen when mutations in Wnt-pathway-related proteins expose infant patients to tooth agenesis, such as Robinow and Gardner syndrome and other early tooth development problems, in addition to adult-onset diseases such as periodontitis [2,3]. This proves that a controlled system is required for normal tooth growth and regeneration.

The Wnt/β-catenin signalling pathway is an essential cellular process that controls bone formation. In short, the pathway works by ensuring the translocation of cytoplasmic β-catenin into the nucleus, where it can act as a co-activator in expressing bone remodelling-related genes. This is ensured by the binding of Wnt ligands to the receptor complex, consisting of a transmembrane protein Frizzled (FZD) and low-density lipoprotein receptor 5 and 6 (LRP5/6) [4]. The binding will then trigger the deactivation of a degradation complex through phosphorylation and conformational changes in FZD. One of the mechanism that controls the signalling state is through the activity of Glycogen Synthase Kinase-3 beta (GSK-3β). The kinase makes up one of the protein subunits in the degradation complex and primarily functions to phosphorylate β-catenin, marking it for degradation. As mentioned previously, the binding of Wnt ligands deactivates the degradation complex, as the complex forms a complex with Dishevelled (Dvl) [5]. This prevents GSK-3β from phosphorylating β-catenin, therefore enabling β-catenin accumulation and later translocation into the nucleus.

Inflammatory diseases are found to alter the expression profile of the cell, including Wnt-related ligands, therefore causing an imbalance in the activity of the Wnt/β-catenin signalling pathway. For instance, patients suffering from periodontitis were observed to overexpress Sclerostin, a putative Wnt/β-catenin signalling pathway inhibitor [6]. This decreases the expression of osteoblastic proteins, therefore decreasing the rate of bone formation. It is also known that inflammation itself stimulates osteoclastogenesis, which will ultimately lead to alveolar bone resorption [7]. The decreased number of osteoblasts in combination with the hyperactivation of osteoclasts is the main factor in alveolar bone loss in periodontitis patients.

The role of the Wnt/β-catenin signalling pathway is being studied extensively in terms of its role in bone remodelling. This leads to the identification of many different therapeutic targets regarding the Wnt/β-catenin signalling pathway. Therefore, the objective of the review is to highlight the latest discoveries on the molecular regulators used to modulate the activation of the Wnt/β-catenin signalling pathway and the effects it produces in the samples. This review also looks into the current idea of how the regulators function in modulating the Wnt/β-catenin signalling pathway at a molecular level. In short, this review aims to explain the role of the Wnt/β-catenin signalling pathway in reducing the effects of periodontitis on alveolar bone.

## 2. Materials and Methods

### 2.1. Search Strategy and Selection

This scoping review was prepared according to the Preferred Reporting Items for Systematic Reviews and Meta-Analyses extension for Scoping Reviews (PRISMA-ScR) checklists obtained from the PRISMA website [8]. For article selection, three databases were chosen: Web of Science, Ovid Medline, and Scopus. The articles were searched using keywords related to periodontitis, bone remodelling, and the Wnt/β-catenin signalling pathway. The inclusion of articles in this review depends on the following criteria: Published between 2007 and 2025, primary articles, the articles are written in English, and focusing on the Wnt/β-catenin signalling pathway in the context of periodontitis. The studies are also excluded if the article is a review or a secondary study. Figure 1 below shows the PRISMA 2020 flow diagram for the identification of studies [9].

For Web of Science, the final search string used to search was a combination of “Wnt/b-catenin”, “Wnt pathway”, “Wnt/b-catenin signalling system”, “bone remodelling”, “bone metabolism”, “osteoblast”, “osteoclast”, “Periodontal inflammation”, “periodontitis”. The results were filtered between 2007 and 2025, and non-English papers were filtered out. Only articles were selected, and retracted papers were also filtered out. Similar techniques were used for other databases. All articles extracted from all three databases were then saved into Mendeley for duplication detection. The exact search string used was included in the Supplementary Materials.

### 2.2. Data Extraction and Management

For data extraction, the titles and abstracts were scanned to ensure the article was appropriate for this review. The articles were analysed to ensure they included all three required elements: the Wnt/β-catenin signalling pathway, bone remodelling, and periodontitis. Information such as the type of treatment used, the study sample, and the findings were compiled into a table.

## 3. Results

### 3.1. Search Results

The search throughout the three databases revealed 51 articles. However, after the screening process to identify their suitability within the scope of this review, only 22 articles satisfied the inclusion criteria. The remaining 29 articles were excluded from the analysis due to a lack of analysis of their aims in relation to either periodontitis or the Wnt signalling pathway.

### 3.2. Study Characteristic

Table 1, Table 2 and Table 3 below show the summary of the studies identified for this review, where Table 1 summarises studies that use in vitro methods, while Table 2 summarises in vivo related methods. Table 3 contains studies that use both methods.

### 3.3. Study Design

The studies comprised 5 in vivo studies, 13 in vitro studies, and 4 studies that utilised both techniques. For in vivo studies, seven studies used rats as their animal model, including Wistar Rats [25,26,27,30,31] and Sprague–Dawley rats [23,29]. The remaining in vivo studies use gingival crevicular fluid extracted from periodontitis patients. Alternatively, in vitro studies either studied human periodontal ligament stem cells (hPDLSCs) [10,11,12,20,21,28,29], rat mesenchymal stem cells [13], MC3T3-E1 [14,15,18,22], or RAW264.7 cell lines [19].

### 3.4. Modulation

Among the studies, various modulating agents were used. Seven of the studies investigated the effects of plant extract on the Wnt/β-catenin signalling pathway [12,14,15,18,27,29,30]. Chemicals [21,26,28], existing drugs for different diseases [13,17], peptides [11,23], RNAs [10,20,22] and infections by other microorganisms [19,31] were also used in found among the studies. Another study utilises laser treatment as a method to control the activity of the Wnt/β-catenin signalling pathway [24]. Overall, 18 studies aimed to increase the activity of the Wnt/β-catenin signalling pathway and 5 studies identified factors that reduced the activity.

Modulators used in the studies targeted different stages of the Wnt/β-catenin signalling pathway. This includes the expression of the signalling pathway agonists and antagonists, expression of receptor proteins, the activation of GSK-3β, stability of β-catenin, and the protein–protein interaction of nuclear β-catenin. Figure 2 below summarises the methods of modulation based on the studies found for this review.

### 3.5. Methods

A common trend in observing the molecular effects of the modulators on the Wnt/β-catenin signalling pathway includes using Western blotting to measure the expression of β-catenin and GSK-3β phosphorylation in the cells, regardless of whether the experiment is in vitro or in vivo. The stability of β-catenin is an important factor in the pathway, as it helps in expressing bone remodelling-related proteins. In addition, the kinase GSK-3β is responsible for the phosphorylation of β-catenin. Therefore, the phosphorylation state of these two proteins can be used as an indicator of the activity of the pathway. In addition to Western blotting, other methods were also used to detect the changes in the levels of both proteins, including Quantitative Reverse Transcription PCR (qRT-PCR), which quantifies the amount of RNA in the cells. A study that observes the effects of proanthocyanidins quantified the amount of nuclear β-catenin in the cells instead of measuring the overall change in the amount of β-catenin inside the cell [29]. This could be argued as a more reflective method of measuring the activity of the pathway, as it looks into the translocation of the protein.

Other proteins that are used in detecting the effects of the modulators include the Runt-related transcription factor 2 (Runx2), a key transcription factor in osteoblast differentiation [11,12,23]. Some articles also used RT-PCR to reveal changes in Wnt signalling inhibitors such as Sclerostin [17,24] and DKK1 [17,22,27], which prevent the binding of Wnt agonists, and Axin2 [19,26], which is a core protein in the degradation complex in the cell.

The receptors involved in the Wnt signalling pathway are also components affected by the molecular regulators used in the studies. Frizzled (FZD) and lipoprotein receptor-related protein (LRP), which are the receptor and the co-receptor, respectively, are also affected by procaine when tested in Wistar Rat MSCs [13]. In addition to expression, the binding affinity between the receptor and Wnt ligands is also investigated by Cui et al. in [28] in measuring the effectiveness of titanium carbide MXene in increasing the activity of the Wnt signalling pathway.

### 3.6. Effects on Downstream Proteins

Another measure of Wnt signalling activation is the expression of osteogenesis biomarkers during osteogenic differentiation. As the Wnt signalling pathway is known for promoting bone formation, the effects of Wnt pathway modulation are also a recurring theme in the studies, especially when it comes to looking into the clinical effects of the modulators within the context of periodontitis. To measure this, changes in genes related to bone turnover are often identified using techniques such as Western blotting and RT-qPCR. Runt-related transcription factor 2 (Runx2) and Osterix (Osx) are transcription factors responsible for osteoblast differentiation and are affected by the molecular modulators in studies conducted in [11,12,13,23,28]. Other genes used to measure osteoblast differentiation include other transcription factors such as the Lymphoid enhancer factor 1 (Lef1) [12], where Lef1 is a transcription factor alongside T Cell Factor (TCF) that binds to β-catenin once translocated into the nucleus. Other genes that were also affected by the modulators used in the studies include collagen type I alpha 1 chain (Col1a1) [11] and Osteoprotegerin (OPG) [25,30].

### 3.7. Physical Effects of the Treatment

The studies also looked at the physical changes in their samples, especially in reducing the effects of periodontitis. Out of all studies that investigated their molecular regulators through in vivo techniques, five studies utilised micro-computed tomography (Micro-CT) to measure effects in terms of the loss of periodontal ligament in [23,25,26,28,31]. The non-invasive property of the procedure makes it a useful method to image the samples and measure parameters such as the Bone Volume/Tissue Volume (BV/TV) ratio and cementum–enamel junction to alveolar bone crest (CEJ-ABC) distance, which are important characteristics of periodontitis.

## 4. Discussion

The Wnt/β-catenin signalling pathway is a crucial pathway that controls many cellular activities. The pathway itself contains two types: canonical and non-canonical. The canonical pathway functions when a Wnt ligand comes into contact with the receptor FZD, aided by the co-receptor LRP5/6 [32]. This interaction leads to the recruitment and phosphorylation of Axin and GSK-3β by LRP6, inhibiting the proteins from forming the degradation complex alongside APC and CK-1 [33]. As the complex does not form, β-catenin is then not recruited by the complex, increasing its stability and therefore enabling it to be translocated into the nucleus [33]. In terms of bone remodelling in periodontitis, the canonical pathway is mostly responsible for osteoblast formation, which helps build bone tissue. This is shown when most of the molecular regulators used in most studies for this review involve the stability of β-catenin, enabling it to transcribe the appropriate proteins. On the other hand, the non-canonical Wnt/β-catenin signalling pathway does not depend on the stability of β-catenin. Instead, activating the non-canonical signalling pathway inhibits the canonical pathway, thereby impairing the ability to repair bone structures.

Among the studies found in this search, molecular modulators originating from plant extracts are one of the most recurring themes. Aside from being widely available and already being used as traditional medicine, as mentioned by [18], the development of various anti-inflammatory and antimicrobial compounds produced by plants as a product of billions of years of evolution as a survival tool is also useful treatment against disease. Aside from plant extracts, drugs against other diseases have an effect on the Wnt/β-catenin signalling pathway. Statins were initially used to lower cholesterol but were later found to increase the activity of the signalling pathway by reducing Dkk1 expression [17]. As the study reveals more about the relationship between cholesterol and the Wnt signalling pathway, more studies are needed to fully understand the pathway’s complexity and its effects on other molecules.

As periodontitis is a bone and inflammation-related disease, many protein expressions are altered in this disease. A study that uses osteogenic growth peptide in [9] identified changes in proteins related to other signalling pathways related to osteogenic differentiation alongside the Wnt/β-catenin signalling pathway, such as the Transforming Growth Factor beta (TGF-β) superfamily, Bone Morphogenetic Proteins (BMPs), and the Hedgehog (Hh) signalling pathway. TGF-β, one of the key proteins in controlling osteogenic differentiation and also involved in inflammatory signalling, has been known to be affected in periodontitis cases, specifically TGF-β1 [34]. The crosstalk between the Wnt/β-catenin signalling pathway and the TGF-β superfamily is highlighted by Runx2 and Osx [35]. The two master regulator proteins are important transcription factors recruited before osteoblast differentiation in both TGF-β and BMP signalling [35], where the expression of both proteins is controlled by the activated Wnt/β-catenin signalling pathway [36].

In addition to the anti-inflammatory properties of TGF-β and some BMPs, the relationship between the Wnt/β-catenin signalling pathway and the pro-inflammatory pathway is also studied by observing the effects of TNF-α on Wnt-related proteins. The study [16] presents the idea that TNF-α inhibits osteogenic differentiation by activating the non-canonical Wnt/β-catenin signalling pathway. This is seen by the reduced expression of downstream genes related to the non-canonical Wnt pathway but increased stability of β-catenin. This is also supported by the study in [30] that investigated the effects of Equisetum arvense (field horsetail) extract. The study discovered that the extract managed to reduce alveolar bone resorption by reducing the activity of NF-kB pathway-related proteins, including TNF-α.

The crosstalk between the two pathways is also explored by Tarapore et al. in 2016 alongside other pathways [37]. The article reveals that inflammation reduces β-catenin binding to the promoter region [37]. This sheds light on the molecular perspective of the relationship between the pro-inflammatory pathway and periodontitis.

The extensive relationship between inflammation, TNF-α, and the Wnt/β-catenin signalling pathway highlights the cross-talk between the Wnt/β-catenin signalling pathway and other signalling pathways. As osteoblast differentiation is an energy-intensive process [38], tight regulation of protein expression is needed at every level, from histone modification and signalling pathways to mRNA regulation. A study conducted by Wu in [29] observed the level of lysine lactylation, a type of histone modification that promotes osteoblast differentiation. In addition, the crosstalk between the Wnt/β-catenin signalling pathway and TGF-β is also related to the Wnt/β-catenin signalling pathway, as the genes that are often affected through modulations in the studies are related to both pathways. Another signalling pathway that is found to be related to osteogenic differentiation alongside the Wnt/β-catenin signalling pathway is the STAT/Jak pathway, as a study in [25] found STAT3 and β-catenin to be colocalised together and assisting β-catenin translocation into the nucleus.

Most studies found for this review focus on in vitro methods. This allows a better understanding of the effects of manipulating the signalling pathway using various regulators, including the effects the regulators pose to the cells. This understanding can be developed into the suitability of the molecules to be used in an in vivo setting to see whether the effects of the molecules are also applicable in a more biologically complex system.

The lack of in vivo studies also highlights the possibility of manipulating the signalling pathway activity as a therapeutic strategy, as the studies found in this review show many targets in the pathway that can be controlled and manipulated to regulate signalling activity. However, only a small number of studies use human-related samples, either from gingival crevicular fluid or cells from patients. To ensure the molecular regulators are safe to be used in clinical settings, further research on the suitability of treatment is needed. Factors such as the delivery method, dosage, and possible side effects need to be considered, as the role of the Wnt/β-catenin signalling pathway is not only responsible for bone remodelling. Regulators that have been used in vivo have a higher chance of being suitable for clinical periodontitis cases, as they have been shown to work inside living organisms; for instance, parathyroid hormone [25], lithium carbonate [26], and MXene [28], where the studies have examined the effects these molecules have on periodontal ligaments, in addition to Wnt/β-catenin signalling pathway-related molecules. The studies also revealed that periodontitis biomarkers are related to the signalling pathway, such as Sclerostin [24] and Dkk-1 [17,22,27], further strengthening the relationship between the signalling pathway and the disease. However, the expression of Sclerostin was found to remain constant after peroral statin treatment in [17], suggesting that Dkk-1 plays a more prominent role in periodontitis, even though both Dkk-1 and sclerostin are Wnt/β-catenin signalling pathway antagonists.

## 5. Conclusions

In conclusion, the role of the Wnt/β-catenin signalling pathway in periodontitis has been found through many biochemical assays, revealing the relationship between the disease and many other bone-related proteins and signalling pathways. This review also highlights the current molecular regulators that have the potential to be used as treatment against the disease, and the stages that the molecules target within the pathway. The role of remodelling within periodontitis is also shown through the ability of the molecules to increase the ability of the cells to undergo osteoblast differentiation, which is the first step in reducing the effects of bone loss that occurs in periodontitis patients.

This review also finds that the common theme in the studies involves protein expression and bone microstructure studies. Even though there are many different types of experiments being done in studying the effects posed by the molecules used, the variability provides an understanding that there are many ways to understand the mechanism of the regulators used in controlling the signalling pathway. This further confirms the relationship between the Wnt/β-catenin signalling pathway and other bone remodelling-related pathways. This is shown through the changes in expression of master regulator proteins such as Osx and Runx2.

Overall, the Wnt/β-catenin signalling pathway is a focus for developing therapy to reduce alveolar bone loss in periodontitis. This is shown through the many steps in which the molecular regulators used in the studies have various effects on multiple stages of the Wnt signalling pathway and the physical effects that were observed after the treatments. However, more studies are needed to ensure the treatment is both safe and effective for human patients, as the pathway itself is responsible for many other life processes.

## Figures and Tables

**Figure 1:**
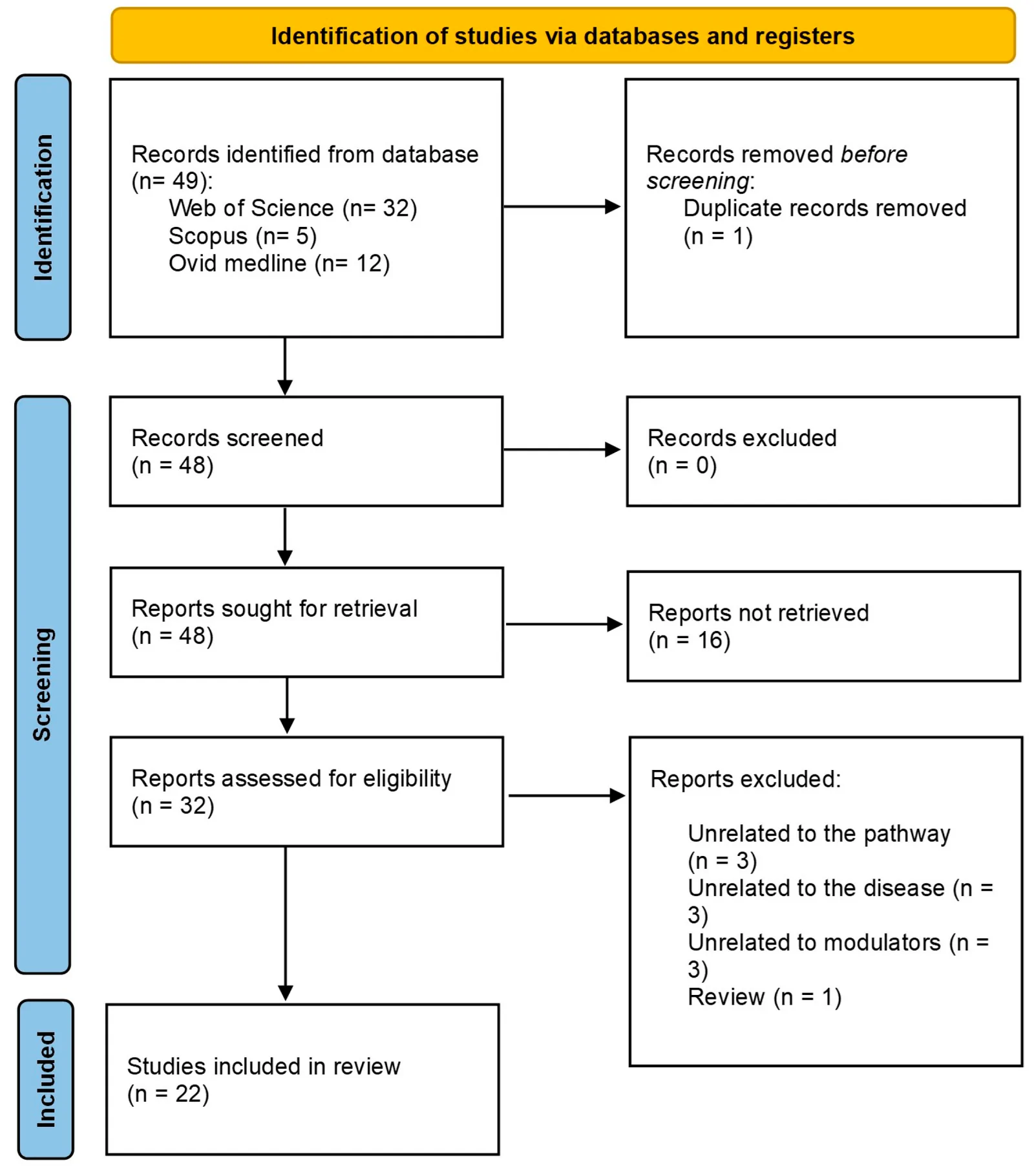
The study identification flow chart according to the PRISMA 2020 flow diagram.

**Figure 2:**
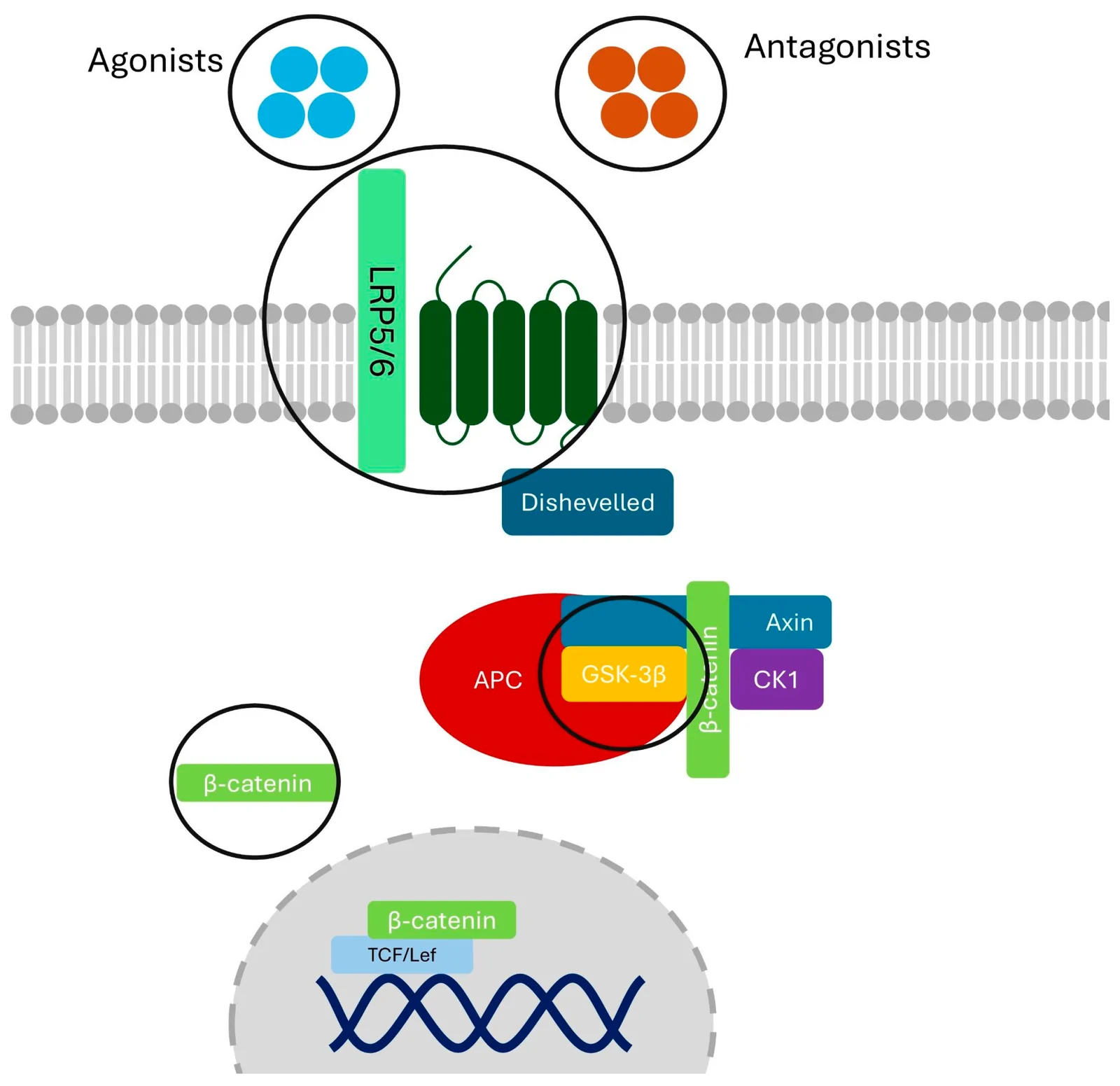
An illustration of the Wnt/β-catenin signalling pathway when no Wnt ligand is bound to the receptors. The targets that are affected by the molecular regulators in manipulating the activity of the signalling pathway are highlighted with black circles.

**Table 1:** The summary of the identified studies using in vitro methods from the search.

Author	Treatment Used	Study Sample	Summary of Results
Yang et al., 2025 [10]	LINC00673 knockdown via shRNA (lnRNA)	human PDLSC	↓ b-catenin ↑ GSK-3β (Western blotting)
Purbantoro et al., 2022 [11]	osteogenic growth peptide (peptide)	human PDLSC	↑ hPDLSC proliferation at low dose ↑ ALP and RUNX2, OSX, COL1A1 (rt-qPCR) ↑ Mineralisation (Alizarin Red S Staining)
Han et al., 2022 [12]	notoginsenoside R1 (NG-R1) (plant extract)	human PDLSC	↑ hPDLSC proliferation (Cell Counting assay) ↑ RUNX2, pGSK-3β, β-catenin, and LEF (Western blot)
Herencia et al., 2016 [13]	Procaine (drug)	Wistar Rat Mesenchymal stem cells	↓ Runx2, Osteocalcin, Osterix mRNA (qRT-PCR) ↓ FZD and LRP expression (qRT-PCR) ↑ GSK-3β (qRT-PCR)
Park, Kim, et al., 2021 [14]	Obacunone (plant extract)	MC3T3-E1 cell line	↑ pGSK-3b ↑ β-catenin (Western blot)
Park, Lee, et al., 2021b [15]	Paeonolide (plant extract)	MC3T3-E1 cell line	↑ Wnt3a expression, pGSK-3β, and β-catenin (Western blot)
X. Li et al., 2020 [16]	TNF-α (signalling molecule)	Sprague–Dawley rat follicle stem cells	↑ pGSK-3β and β-catenin (Western blot)
Duspara et al., 2024 [17]	statin (drug)	gingival crevicular fluid	↑ DKK1 (ELISA)
Park, Lee, et al., 2021a) [18]	paeonoside (plant extract)	MC3T3-E1 cell line	↑ Wnt3a, pGSK-3β, and β-catenin (Western blot)
Chen et al., 2020 [19]	Lipopolysaccharide (infection)	RAW264.7 cell line	↓ β-catenin, DVL2 (Western blot), ↓ LRP5, and Axin2 (RT-PCR)
Wang et al., 2016 [20]	overexpression and knockdown of lncRNA-POIR (lncRNA)	human PDLSCs from periodontitis patients	lncRNA-POIR and miR-182 regulates FoxO1 that competes with TCF-4 to bind with b-catenin (immunoprecipitation)
S. Li et al., 2016 [21]	Dimethyloxalylglycine (chemical)	Human PDLSCs isolated from healthy patients	↓ Wnt signalling (MTT assay). ↓ CEMP1 expression (RT-qPCR)
Y. Zhang et al., 2024 [22]	shRNA to knock down METTL3 (shRNA)	MC3T3-E1 cell line	METTL3 ↓ Dkk3 and Sostdc1 (RT-qPCR). Wnt controls ribosome biogenesis.

**Table 2:** A summary of the identified studies using in vivo methods from the search.

First Author	Treatment Used	Study Sample	Summary of Results
Liang et al., 2022 [23]	LL-37 injection (peptide)	Sprague–Dawley rats	↑ Bone Mineral Density (DXA) and microstructure (micro-CT) ↓ bone loss (ELISA of bone resorption markers) ↑ β-catenin, RUNX2, Osterix (Western blotting).
Pai et al., 2021 [24]	laser irradiation and low-level laser therapy (external)	gingival crevicular fluid	↓ sclerostin (ELISA) ↓ probing pocket depth
C. Zhang et al., 2020 [25]	Intermittent parathyroid hormone (hormone)	Wistar Rats	↑ RANKL (Western blotting) ↓ OPG (immunohistochemistry) β-catenin and STAT translocate into the nucleus (immunofluorescence) ↓ alveolar bone loss (micro-CT)
Kagioka et al., 2023 [26]	lithium carbonate (chemical)	Wistar rats	↑ Axin2 (Immunohistochemistry) ↓ periapical lesion volume (micro-CT)
Lima et al., 2017 [27]	Calendula Officinalis (plant extract)	Wistar rats	↑ Wnt10, β-catenin (immunohistochemistry) ↓ DKK-1 (immunohistochemistry)

**Table 3:** The summary of the identified studies using both in vivo and in vitro methods from the search.

First Author	Treatment Used	Study Sample	Summary of Results
Cui et al., 2021 [28]	Titanium carbide MXene (chemical)	human PDLSC	↑ binding affinity between FZD and Wnt (all-atom-based MD simulation) ↑ β-catenin, Runx2, Ocn, Opn (Western blot) ↑ new bone area (micro-CT)
Wu et al., 2024 [29]	proanthocyanidin (plant extract)	Sprague–Dawley tooth and human PDLSC	↑ osteogenic ability (lactic acid content) ↑ nuclear β-catenin (fluorescence confocal imaging)
Shiba et al., 2022 [30]	Equisetum arvense (plant extract)	Wistar rats	↓ osteoclasts, RANKL (ELISA) ↑ OPG (ELISA) ↑ β-catenin (Western blot)
Dong et al., 2025 [31]	Fusobacterium nucleatum (infection)	Wistar rat cell lines and C57BL/6J male mice	↓ pGSK-3β and β-catenin (Western blot). ANXA2 binds to GSK-3β (Co-immunoprecipitation) ↑ Cementum–enamel junction to alveolar bone crest distance (micro-CT)

## Data Availability

No new data were created or analyzed in this study. Data sharing is not applicable to this article.

## Supplementary Materials

The following supporting information can be downloaded at: https://www.mdpi.com/article/10.3390/ijms27209117/s1.

## Abbreviations

The following abbreviations are used in this manuscript:

LRP5/6	Low-density lipoprotein-related receptor 5 and 6 (LRP5/6)
GSK-3β	Glycogen Synthase Kinase-3 beta
Dvl	Dishevelled
PRISMA-ScR	Preferred Reporting Items for Systematic Reviews and Meta-Analyses extension for Scoping Reviews
hPDLSCs	human periodontal ligament stem cells
qRT-PCR	Quantitative Reverse Transcription PCR
Runx2	Runt-related transcription factor 2
Osx	Osterix
Lef1	Lymphoid enhancer factor 1
TCF	T Cell Factor
Col1a1	collagen type I alpha 1 chain
Opg	Osteoprotegerin
Micro-CT	micro-computed tomography
BV/TV	Bone Volume/Tissue Volume
CEJ-ABC	Cementum-enamel junction to alveolar bone crest
TGF-β	Transforming Growth Factor beta
BMP	Bone Morphogenic Protein
Hh	Hedgehog
