# Supplementary material for: Molecular Regulators for the Wnt/β-Catenin Signalling Pathway for Bone Remodelling in Periodontitis: A Scoping Review
Source: Int J Mol Sci. 2026 Oct 14;27(20):9117. doi: 10.3390/ijms27209117 (PMC13705578; doi:10.3390/ijms27209117)
Supplement: Supplementary file 1 [file ijms-27-09117-s001.zip › Supplementary Data/Papers/both in vivo and in vitro/The surface protein Gbp of Fusobacterium nucleatum inhibits osteogenic differentiation by inactivating the Wntβcatenin pathway via binding to Annexin A2.pdf]

Dong *et al. Journal of Translational Medicine* (2025) 23:540
https://doi.org/10.1186/s12967-025-06569-1

Journal of Translational Medicine

RESEARCH **Open Access**

The surface protein Gbp of *Fusobacterium nucleatum* inhibits osteogenic differentiation by inactivating the Wnt/β-catenin pathway *via* binding to Annexin A2

Rui Dong[1], Meihui Li[2], Xiu Feng Gu[2], Haiting Gao[2], Ziyi Wei[2], Houbao Qi[2*], Jun Zhang[1*] and Qiang Feng[2,3*]

Abstract

Background Periodontitis is a chronic inflammatory disease that significantly impacts periodontal bone regeneration, yet the distinct biological features of osteoblasts in this condition remain poorly understood. This study aims to elucidate the cellular and molecular mechanisms underlying osteoblast dysfunction in periodontitis, with a focus on the role of *Fusobacterium nucleatum* (Fn) and its effector protein, D-galactose-binding periplasmic protein (Gbp).

Method Single-cell RNA sequencing (scRNA-seq) data from human gingival tissues of periodontitis patients (PD) and healthy controls (HC) were analyzed to identify cellular heterogeneity and molecular pathways. An experimental periodontitis model in mice and primary osteoblast cultures were used to investigate the effects of Fn and Gbp on osteogenic differentiation. Transcriptomic analysis, gene set enrichment analysis (GSEA), and protein-protein interaction (PPI) networks were employed to explore the underlying mechanisms.

Results scRNA-seq revealed a reduction in mesenchymal stem cells (MSCs) and osteoblastic lineage cells in PD tissues, with significant downregulation of osteogenic pathways such as Wnt signaling. Fn infection induced alveolar bone destruction in vivo and inhibited osteoblast proliferation, differentiation, and mineralization in vitro. Gbp, an Fn adhesin, similarly impaired osteogenic differentiation by downregulating key osteogenic genes and pathways. Transcriptomic analysis identified shared inflammatory and osteogenic pathways affected by Fn and Gbp, with NF-κB signaling activated and Wnt/β-catenin signaling inhibited. Mechanistically, Gbp interacted with the host protein ANXA2, disrupting the ANXA2/GSK3β complex and inhibiting Wnt/β-catenin signaling, a pivotal route for osteoblast differentiation. ANXA2 knockdown mitigated the Fn/Gbp-induced suppression of osteogenic activity, emphasizing its role in Fn-induced bone loss.

*Correspondence:
Houbao Qi
byqihoubao@126.com
Jun Zhang
zhangj@sdu.edu.cn
Qiang Feng
fengqiangsdu@163.com

Full list of author information is available at the end of the article

© The Author(s) 2025. **Open Access** This article is licensed under a Creative Commons Attribution-NonCommercial-NoDerivatives 4.0 International License, which permits any non-commercial use, sharing, distribution and reproduction in any medium or format, as long as you give appropriate credit to the original author(s) and the source, provide a link to the Creative Commons licence, and indicate if you modified the licensed material. You do not have permission under this licence to share adapted material derived from this article or parts of it. The images or other third party material in this article are included in the article's Creative Commons licence, unless indicated otherwise in a credit line to the material. If material is not included in the article's Creative Commons licence and your intended use is not permitted by statutory regulation or exceeds the permitted use, you will need to obtain permission directly from the copyright holder. To view a copy of this licence, visit http://creativecommons.org/licenses/by-nc-nd/4.0/.

Conclusion This study demonstrates that Fn and its effector Gbp disrupt osteogenic differentiation by inactivating the Wnt/β-catenin pathway binding to ANXA2.

Keywords *Fusobacterium nucleatum*, Osteogenic differentiation, Gbp, ANXA2

Background

Periodontitis is a chronic inflammatory disease characterized by the destruction of alveolar bone and connective tissue, leading to tooth loss if left untreated [1]. Inflammatory cytokines such as interleukin-1 (IL-1), tumor necrosis factor-alpha (TNF-α), and interleukin-6 (IL-6), not only sustain the inflammatory process but also trigger bone degradation while suppressing bone formation mechanisms [2]. Among the various pathogens associated with periodontitis, *Fusobacterium nucleatum* (Fn) has been identified as one of the key players in the progression of the disease [3]. Fn is a Gram-negative anaerobic bacterium that not only contributes to the formation of dental plaque but also interacts with other periodontal pathogens, exacerbating the inflammatory response [4]. Recent studies have highlighted the role of Fn in promoting alveolar bone resorption [5], a hallmark of periodontitis. However, the precise mechanisms by which Fn induces bone destruction and inhibits osteoblast function remain incompletely understood.

Osteoblasts, the primary cells responsible for bone formation, synthesize the bone matrix and play a critical role in maintaining alveolar bone homeostasis [6]. Dysregulation of osteoblast function, particularly during infection, can lead to impaired bone mineralization and increased bone resorption [7]. Emerging evidence suggests that microorganisms can directly affect osteoblasts, leading to reduced bone formation. *Enterococcus faecalis* (*E. faecalis*) was found to inhibit the differentiation and induce apoptosis of osteoblasts [8, 9]. *Porphyromonas gingivalis* (*P. gingivalis*) could invade osteoblasts and inhibit their differentiation and mineralization [10]. We have found that Fn exhibits a notable inhibitory effect on osteoblast proliferation and differentiation [11]. Lipopolysaccharide (LPS), an endotoxin located within the cell wall of Gram-negative bacteria (such as Fn, *P. gingivalis*), hinders the osteogenic differentiation of osteoblast precursors in inflammatory environments by downregulating the Wnt/β-catenin signaling pathway. However, the molecular mechanisms by which Fn regulates osteoblast differentiation are largely unclear.

Recent studies repeatly proved that the surface proteins/adhesin were critical mediators of Fn's pathogenic effects. For examples, Fn colonizes and promotes colorectal carcinogenesis via its FadA [12], Fap2 [13], RadD [14]. In our previous studies, Fn adhesin FadA could bind with PEBP1 in human periodontal ligament stem cells (Osteoblasts) to activate inflammatory response [15]; Fn Gbp promotes THP-1 cell lipid deposition by binding to CypA [16]. Here, we attempt to elucidate the host and virulence factors crucial for Fn-dependent bone loss.

In this study, we firstly compared periodontal osteoblasts between periodontitis patients and healthy individuals using single-cell sequencing data. We built the animal and cell model to prove the potential role of Fn in periodontitis. An HPLC-MS analysis was conducted on the surface toxic protein of Fn and its corresponding host receptor, with the aim of elucidating the primary mechanism by which Fn inhibits osteoblast differentiation. Additionally, time-series RNA-seq was utilized to demonstrate the continuous variations in gene expression profiles and pathways within osteoblasts, both in the presence and absence of Fn and its surface toxic protein stimulation for a long period. This work revealed the pathogenic mechanism by which Fn inhibits osteoblast differentiation, leading to bone loss.

Materials and methods

Bacteria

Fusobacterium nucleatum (ATCC 25586) was anaerobically cultivated in a 37 °C incubator, utilizing an atmosphere composed of 80% N2, 10% H2, and 10% CO2. The culture medium employed was brain–heart infusion (BHI), sourced from Haibo in Qingdao, China, and supplemented with 5 mg/mL of hemin and 1 mg/mL of menadione. The incubation period ranged from 24 to 48 h.

Cell isolation and culture

Primary osteoblasts were isolated from the calvariae of 24 to 48-hour-old fetal Wistar rats through a sequential enzymatic digestion process. The gender-neutral Wistar rats were sourced from the Shandong University Laboratory Animal Center. The entire experimental protocol was approved by the Animal Ethics Committee of the School and Hospital of Stomatology, Cheeloo College of Medicine, Shandong University, and adhered to the guidelines outlined in the National Institutes of Health Guide for the Care and Use of Laboratory Animals. Calvariae, stripped of all soft tissues, were subjected to digestion in 5 ml of alpha-minimum essential medium (α-MEM, supplied by BasalMedia, Shanghai), which contained 0.1% collagenase II (from Sigma-Aldrich, USA) and 0.25% trypsin (from Invitrogen, USA). This digestion process was carried out at 37 °C for a duration of 20 min. Subsequently, the samples were centrifuged at 300×g for 5 min at 4 °C, and the supernatants were discarded. The resulting precipitates were then digested three more times in 5 ml of 0.1% collagenase II, with each digestion lasting

30 min. Finally, the digested cells were collected by centrifugation at 800×g for 10 min. These cells were cultured in α-MEM supplemented with 20% fetal bovine serum in a 37 °C incubator with 5% CO_2. Once the cell monolayer reached 85–90% confluence, the cells were trypsinised and passaged to expand the culture in α-MEM containing 10% fetal bovine serum and no antibiotics.

MC3T3 cells were cultured in DMEM supplemented with 10% fetal bovine serum in a 37 °C incubator with 5% CO_2.

Mouse model experiments

The animal experiments conducted in this study were approved by the Ethics Committee of the School and Hospital of Stomatology, Cheeloo College of Medicine, Shandong University (NO. 20211134) and adhered to the National Institutes of Health's guidelines for the care and use of laboratory animals. C57BL/6J male mice aged 7–8 weeks were obtained from Vital River Laboratory Animal Technology (Beijing, China) and housed at Shandong University's Model Animal Center. Following a week of acclimatization, mice were randomly divided into two groups, with 10 mice in each group and ligated with 4 – 0 thread on the second molar of the maxillae, while the other group without ligature was set as the negative control. Fn was applied to the silk thread at a dose of 1×10^8 CFU every 3 days for 4 weeks. At the end of the experiment, the rats were sacrificed, and their maxillae were harvested and fixed in 4% paraformaldehyde (Solarbio, Beijing, China).

Micro-CT

The maxillary bones were imaged using a Quantum GX2 micro-CT scanner (PerkinElmer, Japan) at settings of 90 kV, 88 μA, and a voxel size of 18 μm. To evaluate alveolar bone loss, six predefined sites around the maxillary second molars in rats were measured: buccal-mesial, buccal-furcation, buccal-distal, palatal-mesial, palatal-furcation, and palatal-distal. Subsequently, the average distances were computed. The maxillary structures were reconstructed utilizing Mimics software and CT Analyzer, with a rectangular area of approximately 1.6 mm^2 beneath the root bifurcation of the maxillary second molar designated as the region of interest (ROI). Within these ROIs, parameters pertaining to alveolar bone were quantified, including bone volume fraction (BV/TV), bone mineral density (BMD), and trabecular spacing (Tb. Sp.).

Assessment of histopathology

The mouse maxillae underwent decalcification using a 12.5% EDTA solution with a pH range of 7.2 to 7.4 for a duration of 2 months. Following decalcification, the maxillae were dehydrated and subsequently embedded in paraffin. Tissue sections, measuring 5 micrometers in thickness and embedded in paraffin, were stained with hematoxylin and eosin (H&E) to enable the observation of morphological alterations in both the alveolar bone and periodontal connective tissue.

Cell proliferation assay

Primary osteoblasts pooled from different rats were cultivated in 24-well plates at a density of 5 × 10^4 cells per well, utilizing osteogenic inductive medium comprising α-MEM enriched with 10% fetal bovine serum, 50 mg/L ascorbic acid, and 10 mmol/L β-glycerophosphate (Sigma). Subsequently, suspensions of Fn were introduced to the osteoblast monolayers at optimal multiplicities of infection (MOIs) of 0, 10, 50, and 100 for 24 h, respectively. This process occurred at 37 °C in a 5% CO_2 environment. A 5-ethynyl-2'-deoxyuridine (EdU) labeling assay was conducted following the instructions provided by the EdU Apollo DNA in vitro kit (RiboBio, Guangzhou, China) to assess the cell proliferation ratio.

Analysis of cell apoptosis

Primary osteoblasts pooled from different rats were plated in 6-well plates at a density of 2 × 10^5 cells per well and treated with various concentrations of Fn (MOIs of 0, 10, 50, and 100). Apoptosis was evaluated at 24 h using the Annexin V-FITC/PI Kit (Beyotime, Beijing, China). Cells were trypsinised, washed with PBS, stained with a propidium iodide-conjugated anti-Annexin V antibody for 15 min in darkness, and analyzed by flow cytometry using a FACScan (Becton Dickinson, Franklin Lakes, NJ, USA).

Single-cell RNA statistical analysis

Single-cell sequencing data of human gingiva tissues was used from GEO database (GSE262668, https://www.ncbi.nlm.nih.gov/geo/query/acc.cgi?acc=GSE262668). The scRNA-seq data was mapped with the human genome (GRCh38) and was analyzed using the R package Seurat v5.0.1. Cells that met one of the following criteria were removed: having fewer than 500 unique molecular identifiers (UMIs), expressing fewer than 200 genes, or having more than 25% transcripts of mitochondrial genes. The R package DoubletFinder v2.0.4 was applied to predict and remove doublets. To remove the batch effect, we applied the batch effect correction analysis by the Harmony package (v1.2.0). Cell grouping was carried on "RunUMAP" function, and the clustering results were displayed using the DimPlot function. The "FindAllMarkers" function in the Seurat package was used to analyze the changes in gene expression between clusters, and the highly expressed genes within the cluster were shown in dotplot. Further functional enrichment analysis was

performed on the results of differential gene expression analysis using the clusterProfiler v4.10.0 package.

Samtools (version 1.17) was employed to extract unaligned sequences from the GRCh38 reference genome. Microbial signal identification from these unaligned FASTQ data was performed using the SAHMI pipeline, followed by taxonomic classification against a standardized microbiome reference database via Kraken2 (version 2.1.3). To refine microbial sequence identification, a two-step denoising strategy was applied: (1) barcode-level filtering retained only taxonomic assignments supported by k-mer correlations detected in ≥ 3 barcodes with ≥ 1 unique k-mer, and (2) sample-level denoising was conducted using default parameters to eliminate spurious signals. Validated microbial sequences were subsequently derived by integrating k-mer correlation analyses with statistical filtering to ensure species-level resolution. Single-cell data were then analyzed to quantify bacterial species per cell, determine the prevalence of bacterial-positive cells across experimental groups, and identify highly recurrent taxa. Additionally, the relative abundance of Fn was calculated as the proportion of cells annotated to this species relative to all bacterial-positive cells.

RNA sequencing (RNA-seq) analysis

Primary osteoblasts pooled from different rats were stimulated with Fn (MO1 = 100) or Gbp (0.1 mg/mL) for 3, 7, 14, 21 days. Total RNA was extracted and were sent to Novogene Tech for subsequent sequencing. We applied fastp (0.23.4) with default parameter to quality control on raw data and the clean data was obtained. The clean data was aligned to the reference genome Rat_mRatBN7.2 using bowtie2 (2-2.5.1). Quantitative analysis was performed using featureCounts (subread 2.0.6) to obtain the expression profile for each sample. The differential analysis was performed using the limma (version 3.58.1) method. The differentially expressed genes were filtered out based on adjusted *p* value < 0.05 and foldchange > 1.5, and ploted in volcano and heatmap. Enrichment of GO and KEGG pathways was implemented using filtered differentially expressed genes, and the significant results were showed in bubble plots with $p < 0.05$. On the other hand, GSEA analysis was performed using all gene sets to compare the pathway changes between the experimental group and the control group, and the significant result ($p < 0.05$) was shown using GseaVis package (version 0.0.5). The ClusterGVis package (version 0.1.1) was used for time-series transcriptome gene expression pattern analysis, where mfuzz method was used for clustering and the results are visualized in heatmap. The interaction network of genes was generated on the String website (https://cn.string-db.org/) and visualized in Cytoscape (3.9.1) and the key subnets was extracted using MCODE modules.

Alkaline phosphatase (ALP) activity and staining

Primary osteoblasts pooled from different rats or MC3T3 cells, at a density of 5 × 10^4 cells per well, were plated onto 6-well plates and subsequently cultivated in osteogenic inductive medium. During this cultivation, the cells were stimulated with Fn suspensions at MO1 = 50 at intervals of 2 to 3 days. On days 3, 7 and 14, the cells were lysed using RIPA buffer for a duration of 30 min while kept on ice. Subsequently, the protein concentration was determined by following the protocol provided with a bicinchoninic acid protein assay kit (CWBIO, Jiangsu, China). At days 3, 7, and 14, the activity of alkaline phosphatase (ALP), recognized as an early osteogenic marker, was detected following the instructions of an ALP activity assay kit, and the absorbance at a wavelength of 520 nm was measured with a microplate reader.

For ALP staining, the BCIP/NBT alkaline phosphatase chromogenic kit from Beyotime, China, was utilized. At days 7 and 14, the medium was discarded, and the cells were thoroughly washed three times with PBS for 3 to 5 min each. Subsequently, the cells were fixed with 4% paraformaldehyde for 15 min and washed again three times with PBS. Next, 1 mL of the staining working solution was added to each well, and the cells were incubated at room temperature in the dark for 20 min. After removing the working solution, the cells were washed three times with double-distilled water and then visualized under a microscope.

Alizarin red staining and calcium quantification

On days 21, the cells were fixed with 4% paraformaldehyde for 10 min and subsequently washed three times with phosphate-buffered saline. To visualize extracellular matrix calcification nodules, the cells were stained with 2% (weight/volume) alizarin red solution at a pH of 4.3, sourced from Sigma-Aldrich. After rinsing with distilled water, the mineralized nodules were scanned within the six-well plates and examined under a light microscope.

For quantification of cellular matrix calcium content, After staining, dispense 500 μL of a 10% cetylpyridinium chloride solution (prepared by dissolving 1 gram in 10 mL of PBS) into each well. Incubate the wells on a shaker at room temperature for 30 min. Carefully aspirate the liquid from each well into appropriately labeled centrifuge tubes, taking care to avoid pipetting up and down to minimize disturbance. If sediment is present, centrifuge the tubes and aspirate the supernatant. Next, take a 96-well plate and add 100 μL of the aforementioned mineralized matrix solution to each well, repeating this process 3 to 5 times to ensure uniform coverage. Finally, use

a microplate reader to measure the optical density (OD) value of each sample at a wavelength of 540 nm.

Quantitative real-time PCR (qRT-PCR)

Cellular RNA was extracted using TRIzol reagent (CWBIO, Beijing, China) and converted to cDNA via the HiFiScript cDNA Synthesis kit (Vazyme). Then, qRT-PCR was conducted with UltraSYBR Mixture (CWBIO) to quantify relative mRNA levels, normalized against β-actin using the 2^(-ΔΔct) method. The primer sequences used in this process are detailed in Table S1.

Western blot analysis

Cells were lysed on ice using a combination of RIPA lysis buffer (Solarbio) and a 1% phosphatase inhibitor (sourced from Boster, Wuhan, China) in a 100:1 ratio. Following sonication, the cells were centrifuged at 12,000 revolutions per minute for 15 min. The supernatant was discarded, and the protein concentration was then determined using a BCA protein detection kit sourced from KeyGEN BioTECH, Nanjing, China. An equal amount of protein (20 micrograms per lane) was subjected to electrophoresis on 10% SDS-PAGE gels and subsequently transferred onto PVDF membranes (Millipore, Billerica, MA, USA). Prior to antibody incubation, the PVDF membranes were soaked in 5% skimmed milk powder for one hour. The membranes were then incubated with primary antibodies (Table S2) overnight at 4 °C. Subsequently, the membranes were incubated with a horseradish peroxidase-conjugated secondary antibody (dilution 1:10,000; Proteintech) for one hour at room temperature. The immunoreactive bands were visualized using enhanced chemiluminescence reagents (Millipore) and scanned using an ultra-sensitive imager (Amersham Imager 600; GE Healthcare Life Sciences, Pittsburgh, PA, USA). The relative protein expressions of bands were provided by the analysis of ImageJ software, which used GAPDH or α-tubulin as the internal control.

Confocal laser scanning microscopy

To observe the cellular localization of Fn, 5×10^4 primary osteoblasts pooled from different rats were plated onto a cell slide within a 12-well plate and allowed to incubate overnight. Subsequently, Fn was stained with CFDA and SE (CFSE) (MCE, Shanghai, China) for 30 min, followed by a PBS wash. The stained Fn was then cocultured with osteoblasts for an hour at 37 °C, and the cells were fixed using 4% Paraformaldehyde. The F-actin and nuclei of Osteoblasts were stained with phalloidin (Abcam, Cambridge, UK) and DAPI (Solarbio, Beijing, China), respectively. The immunofluorescence signals were observed using a confocal laser scanning microscope (Leica, Wetzlar, Germany).

Biotin pull-down assay

The biotin pull-down assay was conducted using the Biotinylated Protein Interaction Pull-Down Kit from Thermo Fisher Scientific (Waltham, USA), following the manufacturer's instructions. Briefly, 1×10^6 primary osteoblasts were grown in a 10 cm dish and labeled with 1 mM EZ-Link Sulfo-NHS-LC-Biotin at 4 °C for 4 h. Membrane proteins were extracted using a Plasma Membrane Protein Isolation and Cell Fractionation Kit from Invent Biotechnologies (Maryland, USA), while surface proteins of Fn were isolated with 1% Triton X-100 in PBS for 1 h at room temperature. Biotinylated OSTEOBLASTS surface proteins were incubated with streptavidin agarose resin for 4 h at 4 °C, followed by an overnight incubation with Fn surface membrane proteins at 4 °C. After washing, elution was performed using elution buffer, and the eluted proteins were analyzed by SDS-PAGE and silver staining. Protein bands were identified through liquid chromatography and mass spectrometry (LC/MS).

Recombinant protein production and purification

The signal peptide-free Gbp was cloned into the pET28a vector, incorporating an N-terminal His tag. *E. coli* BL21 cells harboring the Gbp plasmid were cultivated in Luria-Bertani (LB) medium and induced with 0.5 mM IPTG sourced from Aladdin (Shanghai, China). Subsequently, Gbp was purified employing a His-tag protein purification kit from Byotime (Shanghai, China), followed by desalting, filtration, and concentration using an Amicon® Ultra-30 centrifuge filter manufactured by Millipore (MA, USA).

His pull-down assay

To identify Gbp receptors on the primary osteoblasts membrane, a His pull-down assay was performed using the PolyHis-tagged Protein Interaction Pull-Down Kit from Thermo Fisher Scientific (Waltham, USA), according to the manufacturer's instructions. Cobalt resin was incubated with His-Gbp for 1 h at 4 °C, washed with 10 mM imidazole, and incubated overnight at 4 °C with membrane proteins from osteoblasts. After washing, the resin was eluted with 290 mM imidazole and analyzed by LC/MS to identify osteoblasts membrane proteins.

Coimmunoprecipitation (Co-IP) assay

To perform co-immunoprecipitation (Co-IP) of ANXA2, the anti-ANXA2 antibody was incubated with primary osteoblasts membrane protein, in the presence or absence of recombinant His-Gbp, overnight at 4 °C. Rabbit IgG was used as a negative control. Following this, Protein A/G-magnetic beads (Santa, sc-2003) were added and incubated for an additional 2 h. The beads were then washed thoroughly and boiled in 40 μL of loading dye. The boiled samples were resolved by SDS-PAGE and

immunoblotted using either anti-ANXA2 or anti-His antibody.

Gene silencing

In order to achieve better transfection efficiency, MC3T3 cells were used for ANXA2 silencing. MC3T3 cells seeded in 6-well plates and incubated in a culture medium with 10% FBS overnight. Thereafter, the medium was replaced with a fresh medium and siRNAs were transfected into cells via Lipofectamine 3000 transfection reagents (Invitrogen, Carlsbad, California, USA) in opti-MEM (Genom, China). siRNAs sequences: CCU CCAGAAAGUGUUCGAATT.

Statistical analysis

The cytology experiments were independently replicated three times using cells sourced from three distinct donors, and the results were presented as the mean ± standard deviation (SD). For statistical analysis, GraphPad Prism 9.5 software (MacKiev, Boston, MA, USA) was utilized. The data underwent one-way ANOVA, followed by Tukey's post hoc test or Student's t test for honest significant difference comparisons. Multiple t-tests were conducted to compare variations between groups across different time points. Statistical significance was deemed at a p-value < 0.05.

Results

The function of osteoblasts was inhibited in periodontitis patients

To examine the distinct biological features of osteoblasts in periodontitis, we analyzed single-cell sequencing (scRNA-seq) data (GSE262668) of human gingiva tissues derived from healthy controls (HC), chronic periodontitis (CP) and aggressive periodontitis (AP) patient. Following standard data processing and rigorous quality filtering procedures, we successfully acquired single-cell transcriptomes from a cumulative total of 55,417 individual cells comprised 31,557 cells sourced from HCs, 7,491 cells from CP and 16,269 cells from AP. Unbiased cellular clustering, facilitated by uniform manifold approximation and projection (UMAP) analyses, revealed the presence of twelve distinct clusters by typical cell markers (Fig. 1A, S1A). Interestingly, the percentage of fibroblasts, endothelial cells, and epithelial cells was decreased in the CP and AP group compared with the HC group (Fig. S1B). We further identified the cell populations of the fibroblast cluster by UMAP analyses. The fibroblast cells depicted in Fig. 1A exhibited heterogeneity and were grouped into eight distinct clusters (Fig. 1B). Based on the expression of Decorin (DCN) [17], three clusters (1/2/3) were defined as fibroblasts.

Concretely, the fibroblasts (cluster 1/2/3) were further characterized to identify four subclusters (sC1, sC2, sC5, and sC6) belonging to fibroblasts exhibiting distinct phenotypic traits [18] (sC1, 2, 5, 6) and one subcluster of mesenchymal stem cells, LepR + MSC [19] (sC3) and one subcluster of osteoblastic lineage cells, APOE + OB (sC4) (Fig. 1D). In periodontal tissues, MSCs were distinguished by their fibroblast-like appearance and their capacity to differentiate into multiple cell types, encompassing osteogenic lineage cells [20]. Interestingly, the abundance of LepR + MSC was decreased in the CP and AP group compared with the HC group (Fig. 1E). GO and KEGG analysis showed that MSC were involved in the biological process of regulation of ossification, cell-matrix adhesion and cell junction assembly, and abundant in Wnt, TGF-β and focal adhesion signaling pathway (Fig. 1F and G). Furthermore, subcluster 4 highly expressed APOE and was annotated as APOE + osteoblasts [17] and the genes within this subcluster exhibited enrichment in pathways and biological processes pertinent to osteoblast differentiation, encompassing connective tissue development, regulation of osteoblast differentiation, and the Wnt signaling pathway (Fig. 1H and I). In addition, the bacterial species were annotated in each cell of single-cell data and the proportion of cells annotated to Fn among all annotated bacteria cells were obtained. Fn was enriched in CP group compared with the HC group (Fig. S1C, D). Gene Set Enrichment Analysis (GSEA) revealed that pathways such as NF-κB, TNF, Toll-like receptor signaling, and osteoclast differentiation were activated in APOE + osteoblasts derived from CP, whereas the Wnt signaling pathway was inactivated (Fig. 1J and K, Fig. S1E). These findings underscore the detrimental impact of periodontitis on periodontal bone regeneration and suggest that Fn induced chronic periodontitis may be associated with dysfunction of MSCs and osteoblasts.

Fn induced alveolar bone destruction and inhibited mineralization of osteoblasts

To verify the influence of Fn-induced periodontitis on alveolar bone loss, we developed an experimental periodontitis model in C57BL/6J mice. Fn indeed induced inflammatory responses in mice indicated by an elevation in serum inflammatory cytokines including TNF-α, IL-6, IL-1β, and IL-8 (Fig. 2A). Micro-CT three-dimensional reconstruction unveiled significant alveolar bone resorption in the groups administered Fn orally. Consequently, a reduction in the alveolar crest height, specifically the distance between the cementoenamel junction (CEJ) and the alveolar bone crest (ABC), was observed beneath the bifurcation of the second molar roots (Fig. 2B). Furthermore, histological images showed that Fn infection changed the morphology of the alveolar bone from rounded to irregular (Fig. 2C). Immunohistochemistry (IHC) staining showed the expression level of Col1,

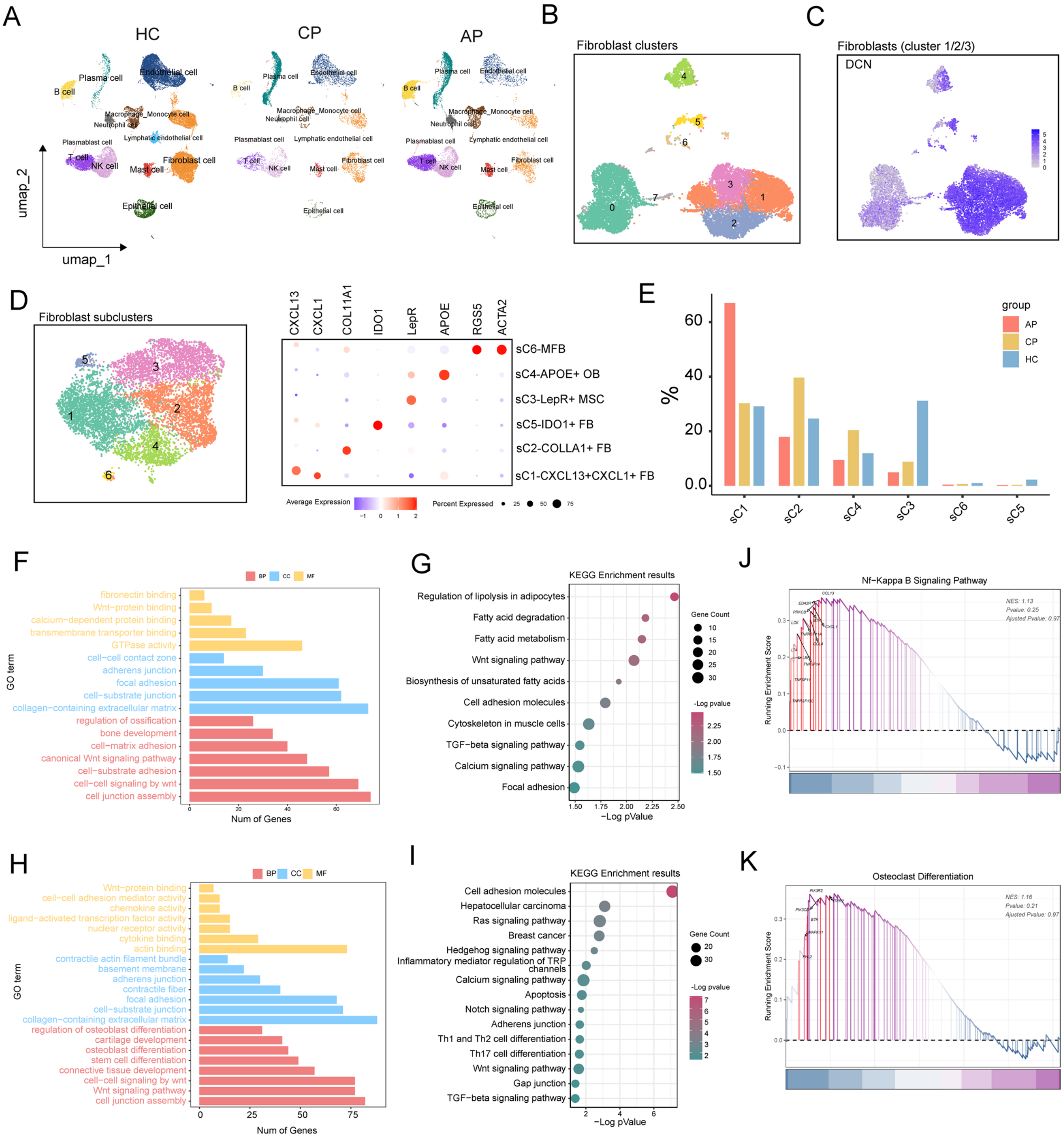

Fig. 1 scRNA-seq analysis of human gingiva tissues. (**A**) Uniform Manifold Approximation and Projection (UMAP) of the 55,417 cells, colored by cell-type annotation from left to right: healthy controls (HC), chronic periodontitis (CP) and aggressive periodontitis (AP). (**B**) UMAP of fibroblasts shown in Fig. 1A. (**C**) UMAP color-coded for expression of DCN to define the clusters of fibroblasts (cluster 1/2/3). (**D**) Left panel: UMAP of fibroblasts (cluster 1/2/3 in C), annotated and colored by clustering. Right panel: dot plots showing distinct expressions of the selected marker genes in each subcluster. MSC: Mesenchymal Stem Cell; OB: Osteoblast; FB: Fibroblast; MFB: myofibroblas. (**E**) The percentage of cells for each of six subclusters as in (D). (**F**) GO annotated by genes of LepR + MSC. (**G**) KEGG pathway annotated by genes of LepR + MSC. (**H**) GO annotated by genes of APOE + OB. (**I**) KEGG pathway annotated by genes of APOE + OB. (**J-K**) Gene Set Enrichment Analysis (GSEA) of NF-κB signaling pathway (J) and osteoclast differentiation (K) used differentially expressed genes (DEGs) in APOE + OB between CP and HC

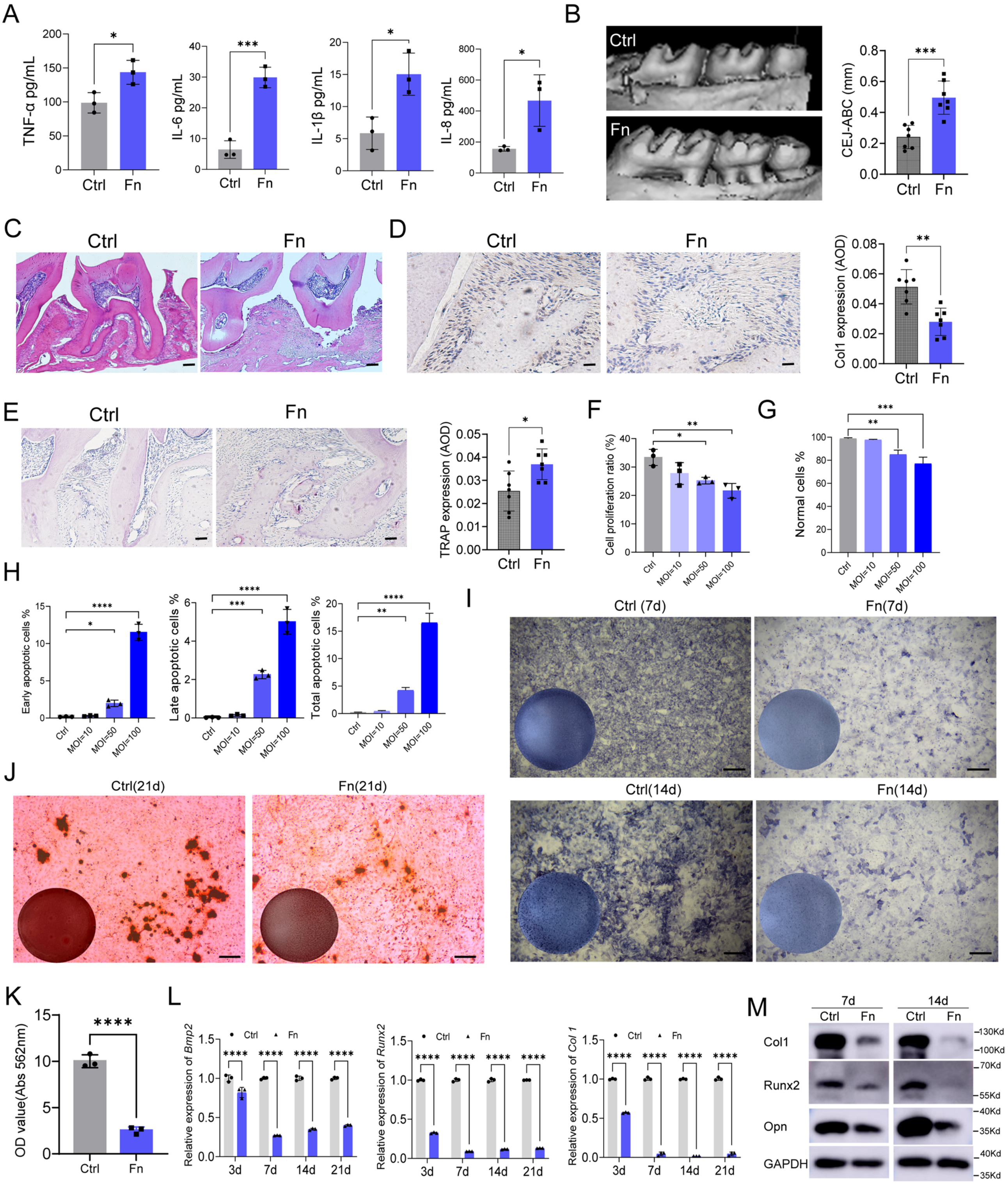

Fig. 2 (See legend on next page.)

an osteogenesis-related protein, was decreased by Fn (Fig. 2D). Tartrate-resistant acid phosphatase (TRAP) staining demonstrated that the activity of TRAP was enhanced by Fn (Fig. 2E). These results suggested that Fn induced alveolar bone destruction in vivo.

Next, a primary rattus calvaria-derived osteoblasts culture [21] with Fn at MOIs of 0, 10, 50, and 100 was

(See figure on previous page.)

Fig. 2 Effects of Fn on the alveolar bone and osteogenesis differentiation. (**A**) ELISA of TNF-a, IL-6, IL-1β and IL-8 in serum from mice experimental periodontitis model (*n* = 3). (**B**) Micro-CT reconstruction of alveolar bone and tooth, and quantitative analyses of CEJ-ABC (*n* = 7). (**C**) Representative images of H&E staining of alveolar bone (*n* = 7). Scale bars, 200 μm. (**D**) Representative images of Col1 staining and quantitative analysis (*n* = 7). (**E**) Representative images of TRAP staining of alveolar bone and quantitative analysis (*n* = 7). (**F**) Statistical results of the cell proliferation ratio of osteoblasts detected by EdU assay. (**G-H**) Statistical analysis of cell apoptosis ratio of osteoblasts analyzed by flow cytometry (*n* = 3). (**I**) Representative images of ALP staining in osteoblasts cocultured with Fn (*n* = 3). (**J**) Representative images of alizarin red staining in osteoblasts cocultured with Fn at 21 days (*n* = 3). (**K**) Calcium quantification from samples in (J). (**L**) Relative mRNA expression of *Col1*, *Runx2* and *Bmp2* in osteoblasts after Fn infection (*n* = 3). (**M**) Protein expression of Col1, Runx2 and Bmp2 in osteoblasts after Fn infection at days 7 and 14 (*n* = 3). Data are presented as the mean ± SD. One-way ANOVA (F-H) and Student's t test (A, B, D, E, K) were used to examine the statistical significance between groups, **P* < 0.05, ***P* < 0.01, and ****P* < 0.001

established to mimic the chronic course of periodontitis. Cell proliferation assay showed that Fn significantly inhibited the rate of primary osteoblast proliferation at MOIs of 50 and 100 (Fig. 2F and Fig. S2A). Cell apoptosis detected by flow cytometry also showed that Fn significantly decreased the normal cell ratio and increased the apoptotic cell ratio at MOIs of 50 and 100, which indicated that Fn from the MOI of 50 significantly induced osteoblasts apoptosis (Fig. 2G-H). Moreover, alkaline phosphatase (ALP) staining showed that ALP activity was inhibited by Fn (Fig. 2I). Alizarin red staining on days 21 showed that the control group exhibited a significant presence of mineralized nodules, whereas the Fn-infected groups showed a reduced number of nodules and minerals (Fig. 2J-K). A reversal assay that suppressing Fn growth by metronidazole was conducted and the ALP analysis showed that metronidazole restored the inhibitory effect of Fn on osteoblast differentiation (Fig. S2B). Furthermore, qRT-PCR revealed that Fn decreased the expression of osteogenesis-related genes, *Col1*, *Runx2* and *Bmp2* in days 3, 7, 14, and 21 during osteogenic induction (Fig. 2L). The protein levels of Col1, Runx2 and Opn were also lowered by Fn infection (Fig. 2M and Fig. S2C). These results suggested that Fn inhibits the osteogenic differentiation capability of primary osteoblasts *in vitro.*

Gbp was an effector of Fn diminished osteogenic differentiation

CFSE-labeled Fn could attach on the surface and invade into plasma of primary osteoblasts by confocal microscopy (Fig. 3A), suggesting Fn may exert its pathogenic effects to osteoblasts by the surface proteins. We thus sought to identify bacterial adhesins that involved in the adhesion process. Biotin pull-down and LC/MS assay was employed to investigate potential interactions between Fn adhesins and membrane proteins of osteoblasts (Fig. 3B, Table S3). Finally, D-galactose-binding periplasmic protein (Gbp), a member of the sugar-binding protein [22] and an important effector of Fn to promote lipid deposition of THP-1 cells identified by us [16], was selected and purified by recombinant protein expression system in *Escherichia coli* (Fig. S3A).

To examine the impact of Gbp on the osteogenic differentiation capability, 0.1 mg/mL Gbp was used to stimulate primary osteoblasts (Fig. S3B). In line with Fn, Gbp significantly weakened ALP activity in osteoblasts during osteogenic induction (Fig. 3C and D). Alizarin red staining showed that Gbp decreased the mineralized nodules (Fig. 3E) and cellular matrix calcium content (Fig. 3F) in osteoblasts. The gene expression of *Col1*, *Runx2* and *Bmp2* in days 3, 7, 14, and 21 and the protein levels of Col1, Runx2 and Opn in days 3 and 7 during osteogenic induction were down-regulated by Gbp (Fig. 3G-H and Fig. S3C). Furthermore, compare to FadA, a known virulence membrane protein of Fn [15], Gbp could more strongly inhibit osteogenic differentiation (Fig. S3D-F). These results indicated that Gbp was an effector of Fn inhibiting the osteogenic differentiation capability of osteoblasts.

Transcriptomic analysis of Fn/Gbp-stimulated osteoblasts

To investigate the dynamics of gene expression in rattus primary osteoblasts during osteogenic differentiation with or without treatment of Fn/Gbp, we analyzed the whole transcriptomes of osteoblasts stimulated with or without Fn (MOIs = 50), Gbp (0.1 mg/mL) for 3, 7, 14, and 21 days. Principal Component Analysis (PCA) conducted on each biological replicate revealed that samples from the same group were tightly clustered, indicating a satisfactory reproducibility of each treatment and a clear distinction in specificity between groups (Fig. 4A). The gene expression profiles of the control group and experimental group at 3, 7, 14, and 21 days were compared by limma (version 3.58.1). Venn plots revealed 651 consistently up-regulated genes and 774 consistently down-regulated genes in the Fn-infected group across the four time points, and the consistent upregulation of 82 genes and down-regulation of 14 genes in the whole process (Fig. 4B and Fig. S4).

Moreover, there were 2484 genes up-regulated by Fn and 950 genes by Gbp, such as *Il6*, *Tnfrsf9* and *Ccl2* that both increased by Fn and Gbp stimulation regardless of the time point (Fig. 4C and D). GO analysis of these differentially expressed genes (DEGs) indicated that they were both mainly involved in the cytokine-mediated signaling pathway, cellular response to lipopolysaccharide and cellular response to interleukin-1 (Fig. S5A and C). KEGG analysis showed that Fn or Gbp stimulation mainly resulted in changes in inflammatory-related pathways,

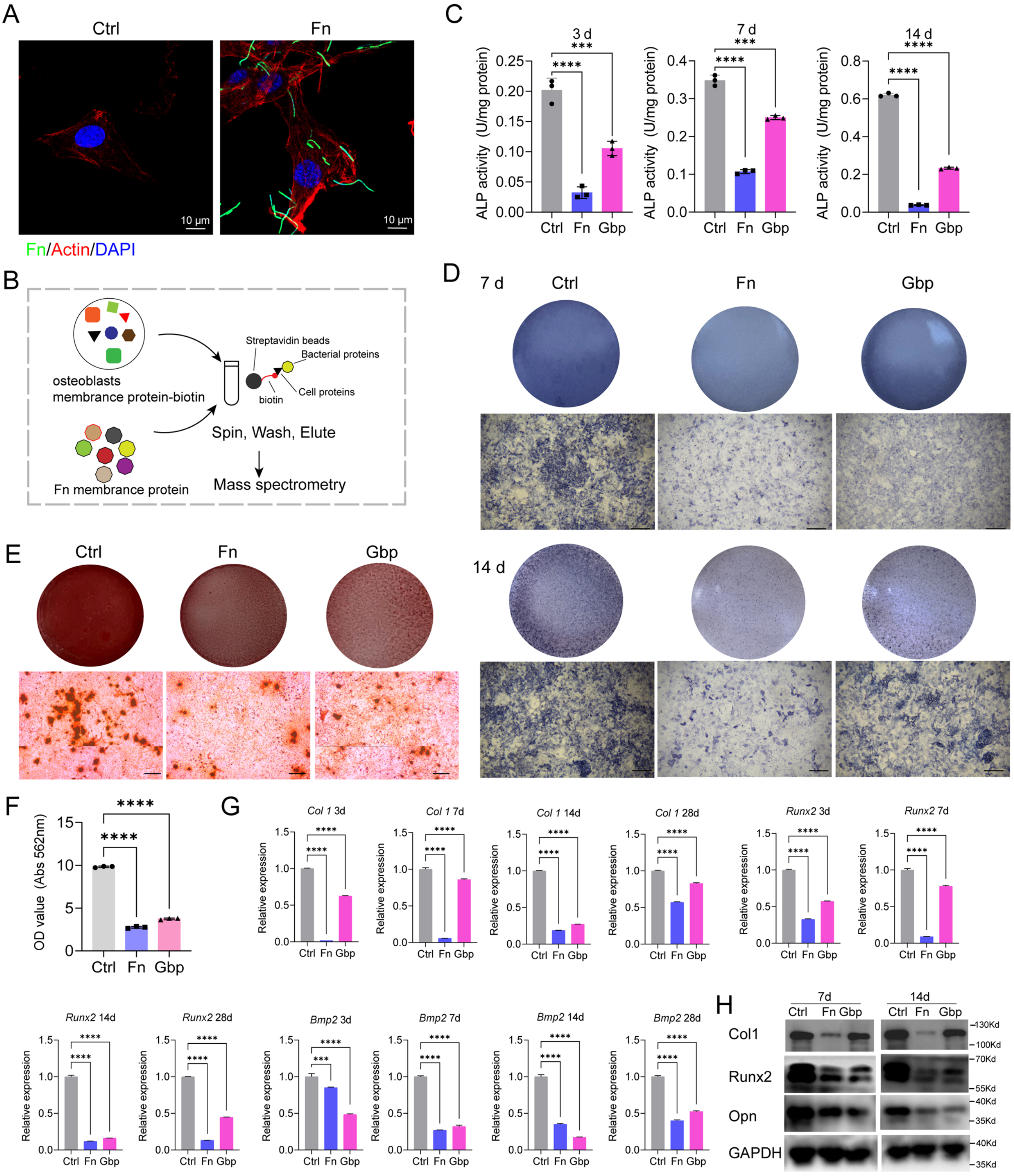

Fig. 3 Gbp was an effector of Fn inhibiting the osteogenic differentiation capability of osteoblasts. (**A**) Representative confocal microscopy images of osteoblasts after co-culture with CFSE- labeled Fn. (**B**) Surface proteins of Fn was identified by mass spectrometry analysis after biotin pull-down assay. (**C**) ALP activity assay in osteoblasts stimulated with Fn (MOI = 50) and Gbp (0.1 mg/mL). (**D**) Representative images of ALP staining in osteoblast stimulated with Fn and Gbp. (**E**) Representative images of alizarin red staining in osteoblasts cocultured with Fn and Gbp at 21 days. (**F**) Calcium quantification from samples in (E). (**G**) Relative mRNA expression of *Col1*, *Runx2* and *Bmp2* in osteoblasts after Fn and Gbp stimulation. (**H**) Protein expression of Col1, Runx2 and Bmp2 in osteoblasts after Fn and Gbp stimulation. Data are presented as the mean ± SD of at least three independent experiments. One-way ANOVA was used to examine the statistical significance between groups, *$P < 0.05$, **$P < 0.01$, and ***$P < 0.001$

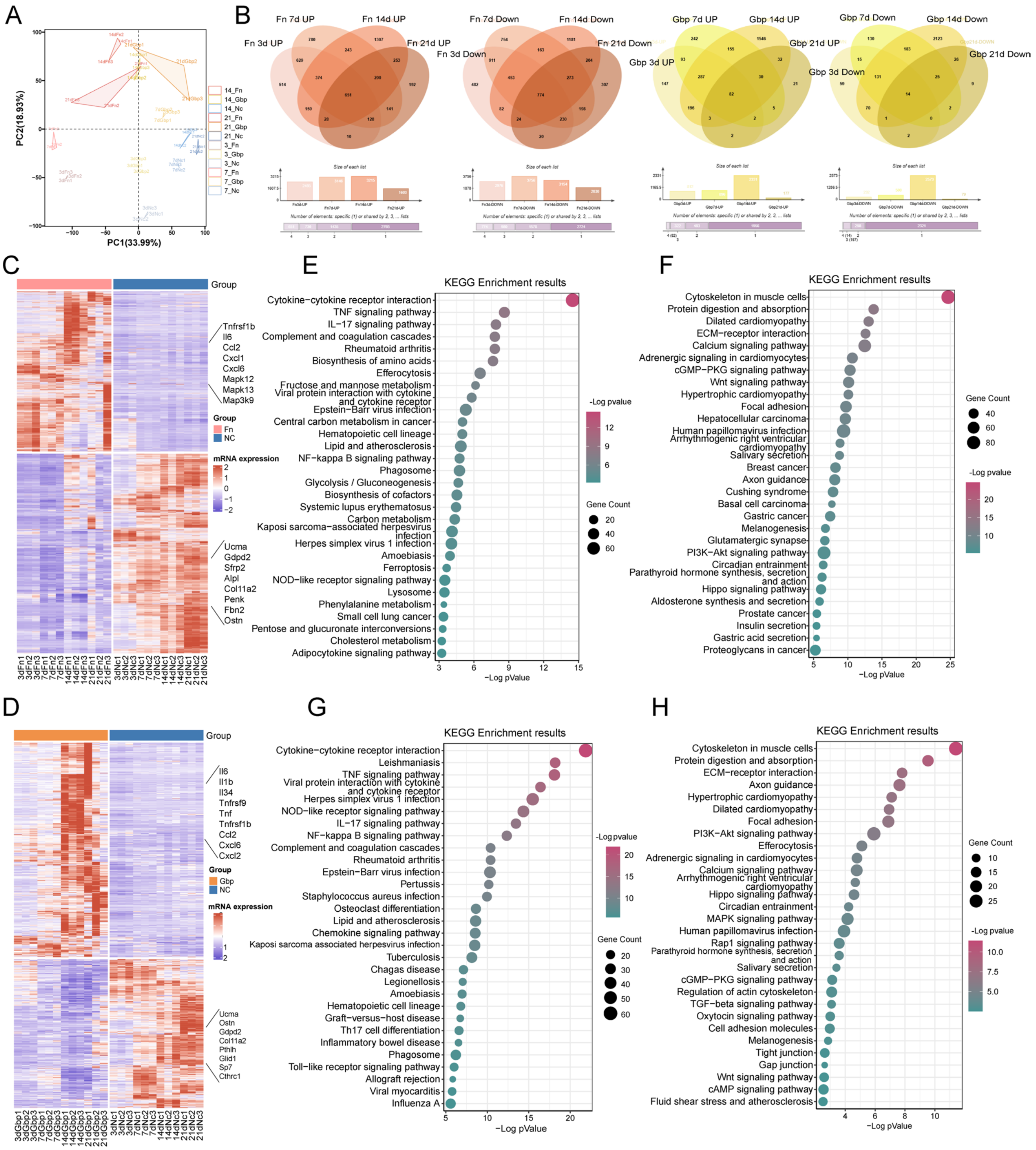

Fig. 4 Transcriptomic analysis of osteoblasts after Fn/Gbp-stimulated at days 3, 7, 14 and 21. (**A**) Principal Component Analysis (PCA) of each biological replicate. (**B**) Venn diagram provided a concise summary of the overlap analysis conducted on upregulated and downregulated DEGs at four distinct time points, separately for the Fn group and the Gbp group. (**C-D**) DEGs of osteoblasts stimulated by Fn (C) and Gbp (D) regardless of the time point. (**E-F**) KEGG pathway annotated by genes up-regulated (E) and down-regulated (F) by Fn. (**G-H**) KEGG pathway annotated by genes up-regulated (G) and down-regulated (H) by Gbp

such as TNF, NF-κ B, and cytokine-cytokine receptor interaction pathways (Fig. 4E and G). In contrast, genes down-regulated by Fn or Gbp stimulation including *Ostn*, *Col11a2* and *Ucma* involved in bone development and osteoblast differentiation biological processes (Fig. S5B and D), and pathways of PI3K-Akt, Wnt, Hippo, etc. (Fig. 4F and H). These results suggested that Fn/Gbp promotes inflammatory responses and inhibits osteogenic-related signaling pathways in osteoblasts.

Time-series genes expression pattern in the Fn/Gbp-induced osteoblasts

The expression patterns of genes were analyzed over time using the time series method in the Mfuzz R package, and those exhibiting similar patterns were grouped into clusters. The findings revealed that Fn and Gbp could respectively induce nine distinct gene expression clusters (Fig. 5A and B). Among them, clusters 2 and 3 maintained a continuous upward trend, whereas cluster 8 consistently declined in Fn groups (Fig. 5A). Cluster 6 exhibited a sustained upward trajectory, whereas Cluster 2 demonstrated a consistent decline in the Gbp groups.

The genes abundant in each cluster were annotated using GO and KEGG databases, from which the primary impacted biological processes and pathways were identified. The results showed that cluster 8 consisting of 1841 genes which were rapidly decreased after Fn stimulation was involved in chondrocyte differentiation, cartilage development and positive regulation of osteoblast differentiation (Fig. 5C). Cluster 2 with 2106 genes continuously down-regulated by Gbp had a relationship with bone development, skeletal system morphogenesis and bone growth, while cluster 6 in Gbp group was mainly involved in cytokine-mediated signaling pathway, canonical NF-κ B signal transduction and cellular response to interleukin-1 (Fig. 5D). Consistent with these results, KEGG analysis showed that cluster 2 of Fn group and cluster 6 of Gbp group were both enriched in pathways related to inflammatory responses, such as MAPK, NF-κ B, and cytokine-cytokine receptor interaction pathways (Fig. 5E and F). Cluster 8 in Fn group and cluster 2 in Gbp group were involved in pathways of osteoblast differentiation, such as Hippo, Wnt and PI3K-Akt signaling pathway (Fig. 5E and F).

Fn/Gbp inhibited osteogenic differentiation by inactivating Wnt/β-catenin pathway

We further analyzed the genes that were co-regulated by Gbp and Fn in rattus primary osteoblasts. Veen diagram showed that 662 up-regulated genes and 551 down-regulated genes at each time point of the two groups (Fig. S6A). Heat-map analysis revealed that overlapping DEGs had a consistent time-series expression pattern in two groups (Fig. S6B). GO and KEGG analysis showed that the up-regulated DEGs involved in the biological process of cellular response to lipopolysaccharide, cytokine-mediated signaling pathway and regulation of inflammatory response, were abundant in TNF, IL-17, NF-κ B and chemokine signaling pathways (Fig. 6A-B). GSEA analysis of these overlapping DEGs also showed that NF-κ B signaling pathway was significantly activated by both Fn and Gbp (Fig. 6C). The down-regulated DEGs were enriched in biological process of chondrocyte differentiation, cartilage development, bone development, and osteoblast differentiation (Fig. 6D), and involved in the Calcium, PI3K-Akt, Hippo, and Wnt signaling pathways (Fig. 6E). GSEA analysis also showed that Wnt signaling pathway was inhibited by both Fn and Gbp (Fig. 6F). Furthermore, the overlapping DEGs from both groups were subjected to protein-protein interaction (PPI) analysis using the STRING database, yielding the identification of 1251 edges and 514 nodes. The up-regulated genes and important nodes were strongly associated with inflammatory response (such as *Il6*, *Il1b* and *Ifng*) (Fig. S7A). Most of the genes in the subnetworks (Fig. S7B) were inflammatory cytokines and participated in NF-κ B signaling pathway, such as *Cxcl1*, *Cxcl6*, *Cxcl3*, *Il6*, *IL1b*, *Nfkbia*, *Nfkb2*, and *Relb*. These results indicated that Gbp and Fn have the same effects on activating inflammatory responses (possibly through the NF-κ B signaling pathway) and inhibiting osteogenic differentiation (possibly via the Wnt signaling pathway).

Next, we screened osteogenic differentiation-related genes that regulated by Fn and Gbp. A total of 48 osteogenic differentiation-related genes were significantly influenced by Fn and Gbp during the whole process. Heat-map showed that *Runx2*, *Bmp3*, *Ostn* and *Ucma* were decreased by Fn and Gbp, whereas *Il6*, *Cebpb* and *Ccl3* were increased (Fig. S8). KEGG analysis showed that these DEGs were significantly enriched in Wnt signaling pathways (Fig. 6G). PPI analysis using the STRING database identified 77 edges and 33 nodes. *Runx2* was shown to be an important node. (Fig. 6H). Western blotting showed that the expression of P-GSK3β and β-catenin was decreased by both Fn and Gbp, indicating that Fn and Gbp inhibit Wnt/β-catenin pathway in osteoblasts (Fig. 6I).

Gbp interacted with ANXA2 to inhibit osteogenic differentiation of osteoblasts

The identification of host proteins capable of interacting with Gbp was undertaken. A His pull-down assay was conducted using His-tagged Gbp in conjunction with primary osteoblast proteins (Fig. 7A, Table S4). Through HLPC-MS analysis, 14 potential proteins overlapped by three independent experiments that could interact with His-Gbp were successfully pinpointed (Fig. 7B). Among these candidates, ANXA2 was a membrane protein

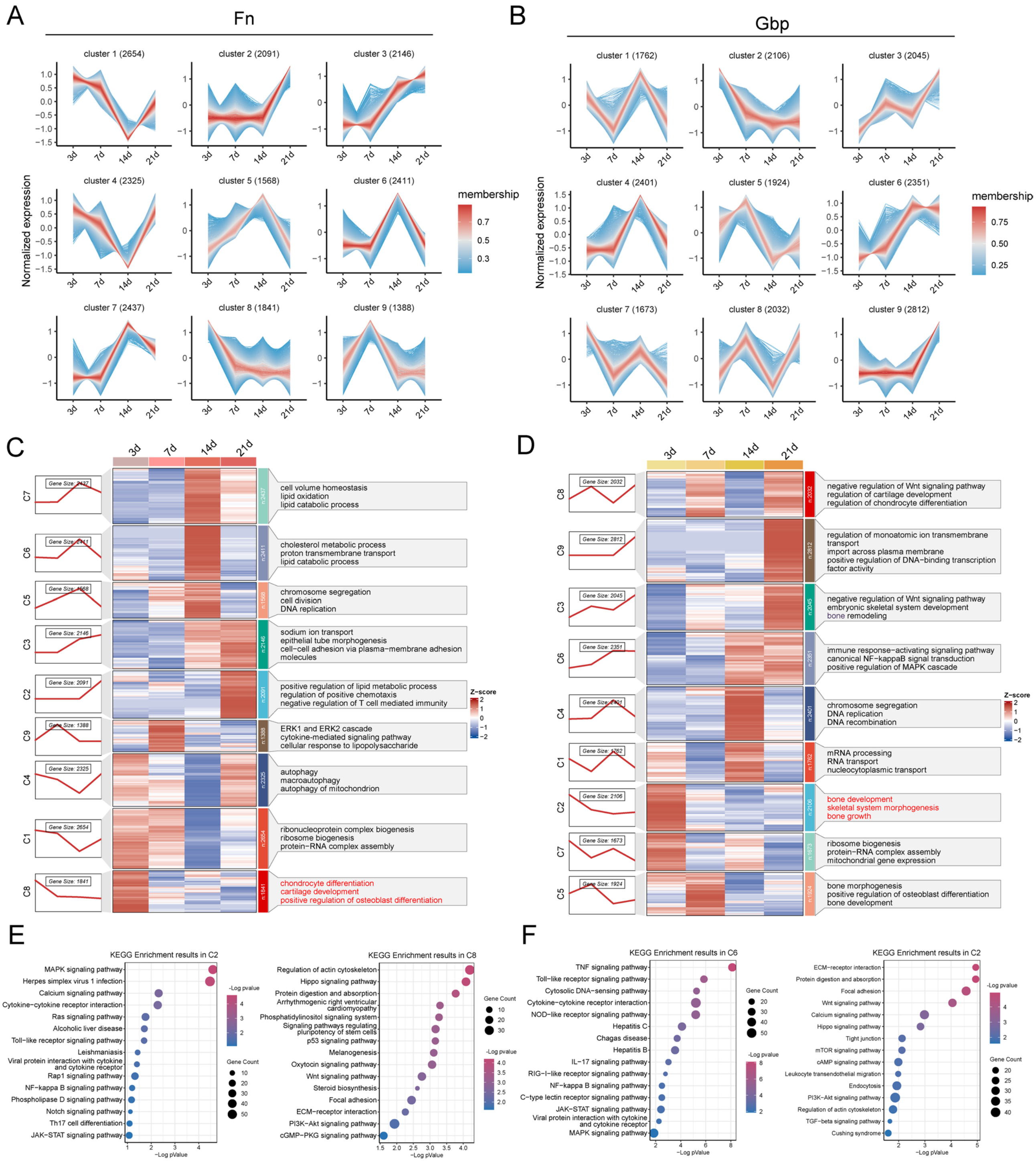

Fig. 5 Time-series genes expression pattern in the Fn/Gbp-induced osteoblasts. (**A**-**B**) Distinct gene expression profiles induced by Fn (A) and Gbp (B) over time using the time series method in the Mfuzz R package. (**C**-**D**) Gene expression trends and GO analysis of each profile over time in osteoblasts stimulated by Fn (C) and Gbp (D). (**E**) KEGG analysis of genes in cluster 2 and 8 of Fn groups. (**F**) KEGG analysis of genes in cluster 6 and 2 of Gbp groups

identified to mediate bacterial adhesion and promote tumorigenesis [23]. ANXA2 was successfully validated to interact with Gbp by co-immunoprecipitation (Co-IP) assay (Fig. 6C).

We investigated the role of ANXA2 in Fn/Gbp inhibited osteogenic differentiation used MC3T3 mouse osteoblasts. Knockdown of ANXA2 by siRNAs (siANXA2) markedly inhibited the attachment and invasion of Fn to osteoblasts (Fig. 7D and Fig. S9). siANXA2 rescued Fn/

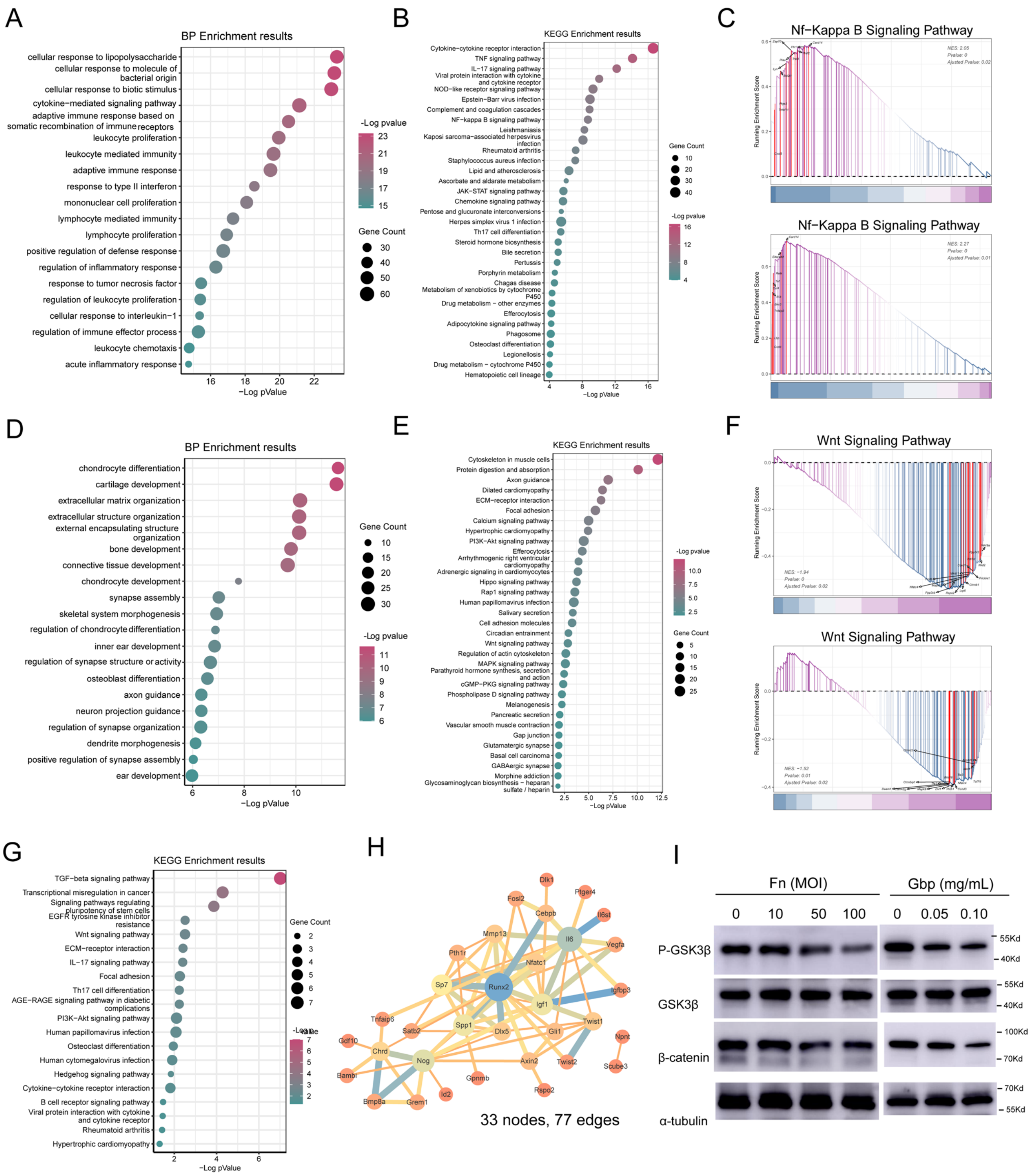

Fig. 6 Transcriptomic analysis of the altered DEGs related to osteogenic differentiation. (**A-B**) Go and KEGG analysis of up-regulated DEGs shared by Fn and Gbp groups. (**C**) GSEA analysis NF-κB pathway in osteoblasts stimulated by Fn (up panel) and Gbp (down panel). (**D-E**) Go and KEGG analysis of down-regulated DEGs shared by Fn and Gbp groups. (**F**) GSEA analysis Wnt pathway in osteoblasts stimulated by Fn (up panel) and Gbp (down panel). (**G**) KEGG analysis of osteogenic differentiation-related genes. (**H**) PPI analysis of osteogenic differentiation-related genes used the STRING database. (**I**) Western blotting analysis the expression of Wnt/β-catenin pathway

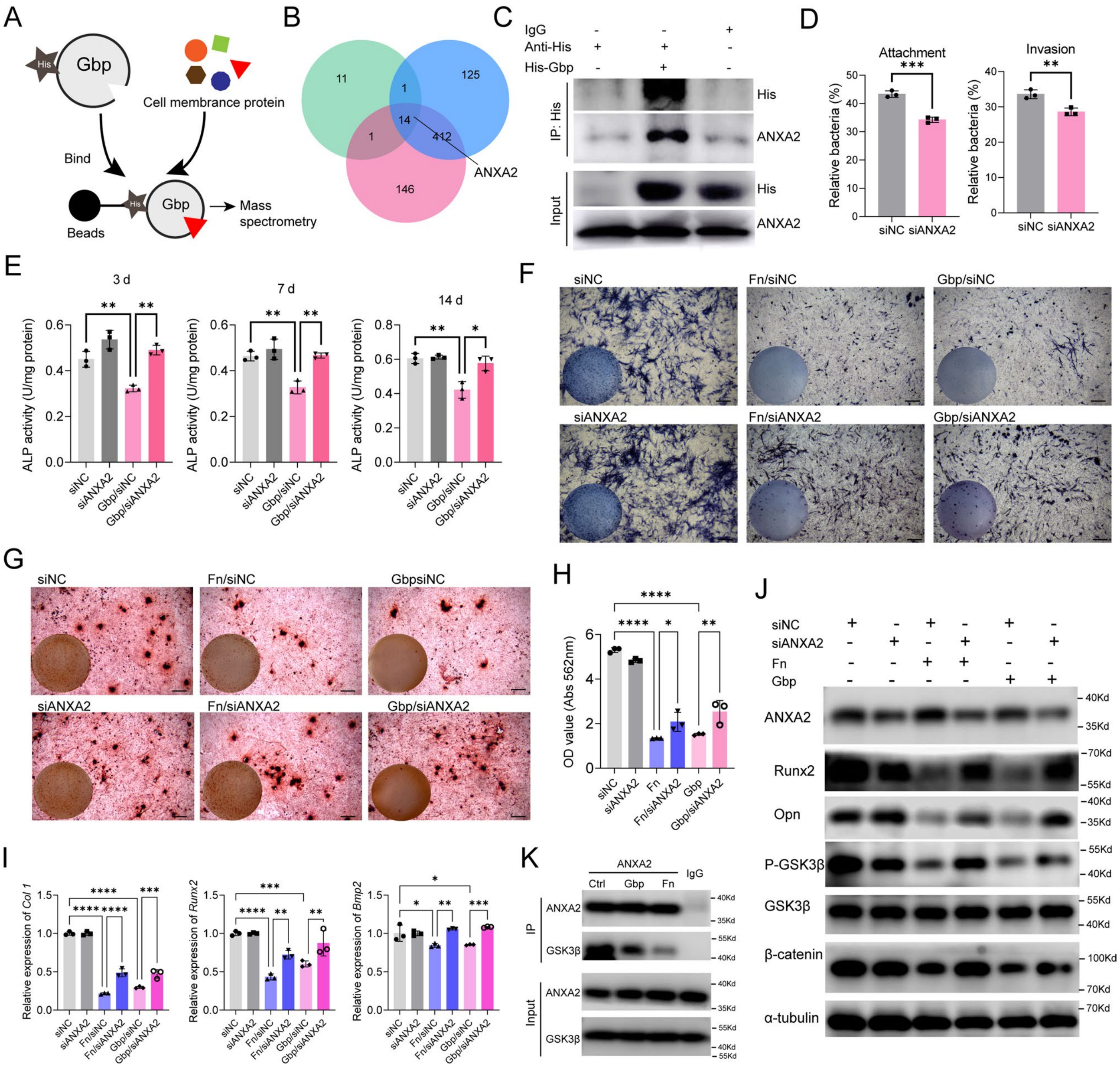

Fig. 7 Gbp bound to ANXA2 on osteoblasts. (**A**) osteoblasts membrane protein was incubated with His-Gbp together with His magnetic beads for His-pull-down assay and mass spectrometry analysis. (**B**) Overlapping membrane proteins from both groups from three independent mass spectrometry analyses. (**C**) Co-IP showing His-Gbp directly bound to ANXA2 in osteoblasts. (**D**) ANXA2 silencing suppressed the attachment and invasion of Fn in osteoblasts ($n = 3$). (**E**) ANXA2 silencing abolished ALP activity induced by Gbp (0.1 mg/mL, $n = 3$). (**F**) Representative images of ALP staining in osteoblast stimulated by Fn/Gbp with or without ANXA2 silencing at 7 and 14 days ($n = 3$). (**G**) Representative images of alizarin red staining in osteoblasts with or without stimulated by Fn/Gbp with or without ANXA2 silencing ($n = 3$). (**H**) Calcium quantification from samples in (G). (I) ANXA2 silencing rescued the gene expression of *Col1*, *Runx2* and *Bmp2* induced by Fn and Gbp ($n = 3$). (**J**) ANXA2 restored the inactivation of Wnt/β-catenin signaling induced by Fn/Gbp. (**K**) Co-IP showing Fn/Gbp inhibits the interaction of ANXA2 and GSK3β. Data are presented as the mean ± SD of at least three independent experiments. One-way ANOVA (E, H, I) and Student's t test (D) were used to examine the statistical significance between groups, $^*P < 0.05$, $^{**}P < 0.01$, and $^{***}P < 0.001$

Gbp-decreased ALP activity (Fig. 7E and F) as well as mineralized nodules and calcium content in osteoblasts (Fig. 7G-H). Furthermore, siANXA2 attenuated Fn/Gbp-induced down-regulation of the gene of *Col1*, *Runx2* and *Bmp2* and the protein of Runx2 and Opn (Fig. 7I and J). In the presence of siANXA2, Fn/Gbp ineffectively inhibited the Wnt/β-catenin signaling pathway (Fig. 7J and Fig.S10A). These results suggested that ANXA2 is essential for Fn/Gbp inhibited osteogenic differentiation of osteoblasts. Co-IP assay showed that ANXA2 could directly bind with GSK-3β in osteoblasts and interaction between ANXA2 and GSK3β was attenuated by Fn/Gbp (Fig. 7K), which indicated that Fn/Gbp-ANXA2 inhibited the Wnt/β-catenin signaling pathway *via* decreasing

the binding of ANXA2/GSK3β. Moreover, systemic inflammation might antagonize Wnt/β-catenin signaling through activating NF-κB pathway [24]. To investigate whether Fn/Gbp-induced NF-κB activity restrained Wnt/β-catenin, we employed the pharmacological NF-κB inhibitor JSH-23 to suppress NF-κB activity. JSH-23 partially rescued Fn/Gbp-induced Wnt/β-catenin inhibition (Fig. S10B), suggesting an inhibitory cross-regulation between these pathways under Fn/Gbp stimulation.

Discussion

The inflammatory process associated with periodontitis leads to characteristic symptoms of bone loss, partly due to the suppression of osteoblast-mediated bone formation [25]. When osteoblasts were either numerically insufficient or functionally impaired, the bone that was resorbed is not adequately replaced, resulting in decoupling. Consequently, this gives rise to a net decrease in bone mass [26]. The scRNA-seq analysis using public date in this study also revealed that the number of LepR + MSC and APOE + osteoblasts was decreased in periodontitis patients compared with healthy individuals. Functional analysis revealed that osteoblast-related signaling pathways, such as Wnt signaling pathway, were suppressed in osteoblasts derived from patients with periodontitis.

The primary etiological factor in periodontitis was the accumulation of pathogenic bacteria in dental plaque. Among these, *P. gingivalis*, *Tannerella forsythia*, and Fn were key pathogens that contribute to disease progression [27]. On the one hand, these bacteria effectively stimulated the host inflammatory response and triggered the release of pro-inflammatory cytokines, including IL-1β, TNF-α, and IL-6, which play a central role in bone resorption [26]. On the other hand, these bacteria directly inhibited osteoblasts activity and function [28]. In a mouse model of periodontitis, it has been observed that *P. gingivalis* invades alveolar osteoblasts, leading to the loss of alveolar bone [29]. *E. faecalis* has been found to inhibit the differentiation and induce apoptosis of osteoblasts in vitro [8, 9]. We have demonstrated that Fn has a direct significant inhibitory effect on osteoblasts function [11]. In this study, we found that Fn caused inflammatory responses and alveolar bone resorption and morphological changes in Fn-induced periodontitis model. However, the precise mechanisms by which Fn induced bone destruction and inhibited osteoblast function need further explore.

Gbp was reported by us to mediate the pathogenic effect of Fn on lipid deposition of THP-1 [16]. In this study, we showed that Gbp acted as a virulence factor on osteoblasts. Furthermore, we developed a long-term in vitro calvarial osteoblasts coculture model, enabling us to study the dynamic changes in osteoblasts following serial stimulation with Fn and Gbp. Gbp was shown to mimic Fn's effects by reducing ALP activity, mineralized nodule formation, and the expression of osteogenic markers (Col1, Runx2, and Bmp2), suggesting that Gbp plays a pivotal role in Fn-induced bone destruction. Next, we proved that Gbp binds to ANXA2 receptor in osteoblasts to mediate the attachment and invasion of Fn to osteoblasts and inhibition of osteoblast differentiation. Knockdown of ANXA2 attenuated the effects of Gbp on ALP activity, mineralized nodule formation, and osteogenic gene expression, suggesting that the Gbp-ANXA2 interaction is critical for Fn's pathogenic effects.

To elucidate the intracellular molecular signaling changes induced by Fn and Gbp in osteoblasts, we conducted time-course RNA sequencing (RNA-seq) at multiple time points and revealed that both Fn and Gbp induce significant changes in the gene expression profiles of osteoblasts, particularly in pathways related to inflammation and osteogenesis. The upregulation of inflammatory genes such as *Il6*, *Tnfrsf9*, and *Ccl2*, coupled with the downregulation of osteogenic genes like Ostn and Col11a2, highlights the dual role of Fn and Gbp in promoting inflammation while suppressing bone formation. These findings are supported by GO and KEGG analyses, which identified enrichment in pathways such as NF-κB, TNF, and Wnt signaling. The activation of NF-κB signaling by Fn and Gbp aligns with previous studies demonstrating its role in inflammatory bone loss, while the inhibition of Wnt signaling provides a mechanistic explanation for the observed suppression of osteoblast differentiation [28]. Furthermore, our western blot assay confirmed that Fn and Gbp-ANXA2 inactivates the Wnt/β-catenin signaling pathway in osteoblasts. ANXA2 could bind with of GSK3β and promotes β-catenin nuclear translocation [30]. Here, we found that Gbp reduces the binding activity of GSK3β with ANXA2, which provides a potential mechanism for the inhibition of Wnt/β-catenin signaling, a key pathway in osteoblast differentiation.

This study highlighted Fn and its effector protein Gbp as key drivers of osteoblast dysfunction, offering novel therapeutic targets. Targeting Fn colonization (e.g., via antibiotics, phage therapy, or probiotics) could mitigate its pathogenic effects on bone homeostasis. Inhibitors of the ANXA2/Gbp interaction or modulators of Wnt/β-catenin signaling could restore osteogenic activity in periodontitis-affected tissues. These strategies may complement or replace conventional treatments) by addressing the underlying molecular pathology. Moreover, the mechanistic insights into Gbp-mediated Wnt inhibition via ANXA2 in bone remodeling might extend beyond periodontitis to other inflammatory bone diseases (e.g., osteoporosis, rheumatoid arthritis-associated bone loss), offering cross-disciplinary therapeutic opportunities.

These advances could transform clinical practice by shifting the paradigm from reactive treatment to proactive prevention. Future investigations should incorporate clinical isolates of Fn from periodontitis patients to reinforce the translational relevance of our findings. Emphasis should be placed on validating these mechanisms in clinical cohorts to evaluate the therapeutic potential of targeting the Gbp-ANXA2 interaction for mitigating periodontitis-associated bone loss.

Conclusions

In summary, this study demonstrates that Fn and its effector Gbp disrupt osteogenic differentiation by inactivating the Wnt/β-catenin pathway and promoting inflammatory responses via NF-κB signaling. ANXA2 plays a pivotal role in mediating these effects, highlighting its potential as a therapeutic target for periodontitis-induced bone loss. These findings provide new insights into the molecular mechanisms underlying periodontal bone regeneration and offer potential strategies for therapeutic intervention.

Abbreviations

ANXA2	Annexin A2
Co-IP	Co-immunoprecipitation
Fn	*Fusobacterium nucleatum*
Gbp	D-galactose-binding periplasmic protein
GO	Gene Ontology Analysis
GSEA	Gene set enrichment analysis
IF	Immunofluorescence
IHC	Immunohistochemistry
KEGG	Kyoto Encyclopedia of Genes and Genomes

Supplementary Information

The online version contains supplementary material available at https://doi.org/10.1186/s12967-025-06569-1.

Supplementary Material 1

Supplementary Material 2

Supplementary Material 3

Acknowledgements

Not applicable.

Author contributions

RD performed cell and mouse experiments. ML performed bioinformatic analyses. XG performed plasmid construction and recombinant protein generation. HG assisted on cell experiments. ZW assisted on mouse experiments. HQ analyzed the experiments and wrote the manuscript. JZ reviewed the manuscript and participated in conception and design. QF designed and supervised the study and revised the manuscript. All authors read and approved the final manuscript.

Funding

This project was supported by grants from the National Natural Science Foundation of China (No. 82270980 and 82071122), the National Clinical Key Specialty (Periodontology) Construction Project, the National Young Scientist Support Foundation (2019), Periodontitis innovation team of Jinan City (2021GXRC021), Major Innovation Projects in Shandong Province (No. 2021SFGC0502), Oral Microbiome Innovation Team of Shandong Province (No. 2020KJK001).

Data availability

scRNA-seq data (GSE262668) of human gingiva tissues was downloaded from the GEO datasets from the National Center for Biotechnology Information (NCBI) database. The RNA-seq data presented in this study were openly available in the Genome Sequence Archive in National Genomics Data Center, China National Center for Bioinformation/Beijing Institute of Genomics, Chinese Academy of Sciences at https://ngdc.cncb.ac.cn/gsa/search?searchTerm=CRA022397, reference number CRA022397. All data, analytic methods, and study materials will be made available to other researchers upon reasonable request.

Declarations

Ethics approval

All animal experiments conducted in this study were approved by the Ethics Committee of the School and Hospital of Stomatology, Cheeloo College of Medicine, Shandong University (NO. 20211134) and adhered to the National Institutes of Health's guidelines for the care and use of laboratory animals.

Consent for publication

All authors agreed on the manuscript.

Competing interests

The authors have no competing interests to declare.

Author details

[1]Department of Orthodontics, School and Hospital of Stomatology, Cheeloo College of Medicine, Shandong Key Laboratory of Oral Tissue Regeneration and Shandong Engineering Laboratory for Dental Materials and Oral Tissue Regeneration, Shandong University, Jinan, China
[2]Department of Human Microbiome, School and Hospital of Stomatology, Cheeloo College of Medicine, Shandong Key Laboratory of Oral Tissue Regeneration & Shandong Engineering Research Center of Dental Materials and Oral Tissue Regeneration & Shandong Provincial Clinical Research Center for Oral Diseases, Shandong University, Jinan, China
[3]BOP Joint Oral Microbiome Laboratory, Shandong University, Jinan, China

Received: 15 January 2025 / Accepted: 4 May 2025
Published online: 14 May 2025

References

1. Slots J. Periodontitis: facts, fallacies and the future. Periodontol 2000. 2017;75:7–23.
2. Mundy GR. Osteoporosis and inflammation. Nutr Rev. 2007;65:S147–151.
3. Zhang L, Zhang D, Liu C, Tang B, Cui Y, Guo D, Duan M, Tu Y, Zheng H, Ning X, et al. Outer membrane vesicles derived from Fusobacterium nucleatum trigger periodontitis through host overimmunity. Adv Sci (Weinh). 2024;11:e2400882.
4. Kolenbrander PE. Multispecies communities: interspecies interactions influence growth on saliva as sole nutritional source. Int J Oral Sci. 2011;3:49–54.
5. Idrus E, Harsono TS, Lestari W, Suniarti DF. Fusobacterium nucleatum mechanism of action in alveolar bone destruction: scoping review. J Indian Soc Periodontol. 2024;28:290–6.
6. Omi M, Mishina Y. Roles of osteoclasts in alveolar bone remodeling. Genesis. 2022;60:e23490.
7. Li Y, Ling J, Jiang Q. Inflammasomes in alveolar bone loss. Front Immunol. 2021;12:691013.
8. Wang L, Jin H, Ye D, Wang J, Ao X, Dong M, Niu W. Enterococcus faecalis lipoteichoic acid-induced NLRP3 inflammasome via the activation of the nuclear factor kappa B pathway. J Endod. 2016;42:1093–100.
9. Li Y, Tong Z, Ling J. Effect of the three Enterococcus faecalis strains on apoptosis in MC3T3 cells. Oral Dis. 2019;25:309–18.
10. Zhang W, Swearingen EB, Ju J, Rigney T, Tribble GD. Porphyromonas gingivalis invades osteoblasts and inhibits bone formation. Microbes Infect. 2010;12:838–45.
11. Gao H, Sun T, Yang F, Yuan J, Yang M, Kang W, Tang D, Zhang J, Feng Q. The pathogenic effects of Fusobacterium nucleatum on the proliferation,

osteogenic differentiation, and transcriptome of osteoblasts. Front Cell Dev Biol. 2020;8:807.
12. Rubinstein MR, Wang X, Liu W, Hao Y, Cai G, Han YW. Fusobacterium nucleatum promotes colorectal carcinogenesis by modulating E-cadherin/β-catenin signaling via its FadA adhesin. Cell Host Microbe. 2013;14:195–206.
13. Abed J, Emgård JE, Zamir G, Faroja M, Almogy G, Grenov A, Sol A, Naor R, Pikarsky E, Atlan KA, et al. Fap2 mediates Fusobacterium nucleatum colorectal adenocarcinoma enrichment by binding to tumor-expressed Gal-GalNAc. Cell Host Microbe. 2016;20:215–25.
14. Zhang L, Leng XX, Qi J, Wang N, Han JX, Tao ZH, Zhuang ZY, Ren Y, Xie YL, Jiang SS, et al. The adhesin RadD enhances Fusobacterium nucleatum tumour colonization and colorectal carcinogenesis. Nat Microbiol. 2024;9:2292–307.
15. Wang Y, Wang L, Sun T, Shen S, Li Z, Ma X, Gu X, Zhang X, Peng A, Xu X, Feng Q. Study of the inflammatory activating process in the early stage of Fusobacterium nucleatum infected PDLSCs. Int J Oral Sci. 2023;15:8.
16. Shen S, Sun T, Ding X, Gu X, Wang Y, Ma X, Li Z, Gao H, Ge S, Feng Q. The exoprotein Gbp of Fusobacterium nucleatum promotes THP-1 cell lipid deposition by binding to CypA and activating PI3K-AKT/MAPK/NF-κB pathways. J Adv Res. 2024;57:93–105.
17. Chen Y, Wang H, Yang Q, Zhao W, Chen Y, Ni Q, Li W, Shi J, Zhang W, Li L, et al. Single-cell RNA landscape of the osteoimmunology microenvironment in periodontitis. Theranostics. 2022;12:1074–96.
18. Baryawno N, Przybylski D, Kowalczyk MS, Kfoury Y, Severe N, Gustafsson K, Kokkaliaris KD, Mercier F, Tabaka M, Hofree M, et al. A cellular taxonomy of the bone marrow stroma in homeostasis and leukemia. Cell. 2019;177:1915–32. e1916.
19. Zhou BO, Yue R, Murphy MM, Peyer JG, Morrison SJ. Leptin-receptor-expressing mesenchymal stromal cells represent the main source of bone formed by adult bone marrow. Cell Stem Cell. 2014;15:154–68.
20. Alvarez C, Monasterio G, Cavalla F, Córdova LA, Hernández M, Heymann D, Garlet GP, Sorsa T, Pärnänen P, Lee HM, et al. Osteoimmunology of oral and maxillofacial diseases: translational applications based on biological mechanisms. Front Immunol. 2019;10:1664.
21. McCarthy TL, Centrella M, Canalis E. Further biochemical and molecular characterization of primary rat parietal bone cell cultures. J Bone Min Res. 1988;3:401–8.
22. Borrok MJ, Kiessling LL, Forest KT. Conformational changes of glucose/galactose-binding protein illuminated by open, unliganded, and ultra-high-resolution ligand-bound structures. Protein Sci. 2007;16:1032–41.
23. Fu K, Cheung AHK, Wong CC, Liu W, Zhou Y, Wang F, Huang P, Yuan K, Coker OO, Pan Y, et al. Streptococcus anginosus promotes gastric inflammation, atrophy, and tumorigenesis in mice. Cell. 2024;187:882–e896817.
24. Huang X, Wei P, Fang C, Yu M, Yang S, Qiu L, Wang Y, Xu A, Hoo RLC, Chang J. Compromised endothelial Wnt/β-catenin signaling mediates the blood-brain barrier disruption and leads to neuroinflammation in endotoxemia. J Neuroinflammation. 2024;21:265.
25. Alghamdi B, Jeon HH, Ni J, Qiu D, Liu A, Hong JJ, Ali M, Wang A, Troka M, Graves DT. Osteoimmunology in periodontitis and orthodontic tooth movement. Curr Osteoporos Rep. 2023;21:128–46.
26. Graves DT, Li J, Cochran DL. Inflammation and uncoupling as mechanisms of periodontal bone loss. J Dent Res. 2011;90:143–53.
27. Socransky SS, Haffajee AD, Cugini MA, Smith C, Kent RL Jr. Microbial complexes in subgingival plaque. J Clin Periodontol. 1998;25:134–44.
28. Pan D, Hao Y, Tao Y, Li B, Cheng L. The influence of microorganisms on bone homeostasis in apical periodontitis. Arch Oral Biol. 2024;170:106153.
29. Zhang W, Ju J, Rigney T, Tribble G. Porphyromonas gingivalis infection increases osteoclastic bone resorption and osteoblastic bone formation in a periodontitis mouse model. BMC Oral Health. 2014;14:89.
30. Peng M, Yang L, Liao J, Le X, Dai F, Sun R, Wu F, Jiang Y, Tian R, Shao B, et al. The novel DNA methylation marker FIBIN suppresses non-small cell lung cancer metastasis by negatively regulating ANXA2. Cell Signal. 2024;120:111197.

Publisher's note

Springer Nature remains neutral with regard to jurisdictional claims in published maps and institutional affiliations.
