# Supplementary material for: Molecular Regulators for the Wnt/β-Catenin Signalling Pathway for Bone Remodelling in Periodontitis: A Scoping Review
Source: Int J Mol Sci. 2026 Oct 14;27(20):9117. doi: 10.3390/ijms27209117 (PMC13705578; doi:10.3390/ijms27209117)
Supplement: Supplementary file 1 [file ijms-27-09117-s001.zip › Supplementary Data/Papers/both in vivo and in vitro/Ultrathin 2D Titanium Carbide MXene (Ti3C2Tx) Nanoflakes Activate WNTHIF1αMediated Metabolism Reprogramming for Periodontal Regeneration.pdf]

ADVANCED HEALTHCARE MATERIALS
www.advhealthmat.de

Check for updates

Ultrathin 2D Titanium Carbide MXene ($Ti_3C_2T_x$) Nanoflakes Activate WNT/HIF-1α-Mediated Metabolism Reprogramming for Periodontal Regeneration

Di Cui, Na Kong, Liang Ding, Yachong Guo, Wenrong Yang,* and Fuhua Yan**

Periodontal defect regeneration in severe periodontitis relies on the differentiation and proliferation of periodontal ligament cells (PDLCs). Recently, an emerging 2D nanomaterial, MXene ($Ti_3C_2T_x$), has gained more and more attention due to the extensive antibacterial and anticancer activity, while its potential biomedical application on tissue regeneration remains unclear. Through a combination of experimental and multiscale simulation schemes, $Ti_3C_2T_x$ has exhibited satisfactory biocompatibility and induced distinguish osteogenic differentiation of human PDLCs (hPDLCs), with upregulated osteogenesis-related genes. $Ti_3C_2T_x$ manages to activate the Wnt/β-catenin signaling pathway by enhancing the Wnt-Frizzled complex binding, thus stabilizing HIF-1α and altering metabolic reprogramming into glycolysis. In vivo, hPDLCs pretreated by $Ti_3C_2T_x$ display excellent performance in new bone formation and osteoclast inhibition with enhanced RUNX2, HIF-1α, and β-catenin in an experimental rat model of periodontal fenestration defects, indicating that this material has high efficiency of periodontal regeneration promotion. It is demonstrated in this work that $Ti_3C_2T_x$ has highly efficient therapeutic effects in osteogenic differentiation and periodontal defect repairment.

1. Introduction

As a disease caused by bacteria, periodontitis is characterized by the loss of periodontal tissue support.[1] Despite successful elimination of inflammation and decades of efforts to reconstruct the periodontal apparatus, periodontal regeneration remains a challenging clinical issue for dentists.[2–4] Human periodontal ligament cells (hPDLCs), typical seed cells with stem-cell-like properties, are extracted from the periodontal ligament and reported as the most promising seed cell for periodontal regeneration.[5] The cells we used in the present study is a heterogeneous population of periodontal ligament cell (PDLC) fibroblasts, which consisted of multiple cell populations, such as osteoblasts, cementoblasts, endothelial cells, fibroblasts, epithelial cell rests of Malassez, and sensory cells.[6–8] Taking the fact that periodontal tissue is a combination of alveolar bone, gingiva tissue, periodontal ligament, and cementum, it is preferred to choose the heterogeneous resource cell population, which is more representative of periodontal ligament tissues, as compared to a highly-purified multipotent stem cell population.[9,10] This kind of stem cell-based tissue engineering predominantly relies on the stem cells expansion in vitro.[11–13]

Recently, the development of biomaterials with stem cells has shown the promising results for tissue regeneration. Due to its ultrathin structures and intriguing physicochemical properties, MXene has attracted increased attention as a new family member of 2D nanomaterials.[14] They were synthesized by the extraction of the A-element layer from the MAX phases, where the meaning of M is a transition metal carbide in the early phase; A denotes an A group element, and X is C or N.[15,16] MXene has exhibited great potential in application of antibacterial and anticancer.[17,18] Rasool et al. reported that $Ti_3C_2T_x$ showed an antibacterial

D. Cui, L. Ding, F. Yan
Nanjing Stomatological Hospital
Medical School of Nanjing University
Nanjing, Jiangsu 210008, China
E-mail: yanfh@nju.edu.cn

N. Kong, W. Yang
School of Life and Environmental Science
Deakin University
Waurn Ponds, Victoria 3216, Australia
E-mail: wenrong.yang@deakin.edu.au

Y. Guo
Kuang Yaming Honors School
Nanjing University
Nanjing 210023, China
E-mail: yguo@nju.edu.cn

Y. Guo
Institute Theory of Polymers
Leibniz-Institut für Polymerforschung Dresden
Dresden 01069, Germany

The ORCID identification number(s) for the author(s) of this article can be found under https://doi.org/10.1002/adhm.202101215

© 2021 The Authors. Advanced Healthcare Materials published by Wiley-VCH GmbH. This is an open access article under the terms of the Creative Commons Attribution-NonCommercial License, which permits use, distribution and reproduction in any medium, provided the original work is properly cited and is not used for commercial purposes.

DOI: 10.1002/adhm.202101215

efficiency toward Gram-negative *E. coli* and Gram-positive *B. subtilis*. Xing et al. utilized cellulose/MXene hydrogels in anticancer photothermal therapy and found it highly efficient in destruction. According to Pan et al.,[19] in vitro, 2D MXene with 3D-printed scaffolds could upregulate osteoblast-related gene expression, subsequently stimulating the cell proliferation and the osteogenesis of bone mesenchymal stem cells and accelerating the growth of newborn bone tissue in vitro. These exciting research progresses have been made, however, the effect of MXene on hPDLC osteogenesis and periodontal regeneration is currently unclear.

Metabolic reprogramming is a prerequisite for the establishment of stem cells.[20,21] At the prime stage, undifferentiated stem cells remain in a low oxygen niche to maintain stemness. When cells divide, extracellular acidification increases during the early period, indicating that metabolism switches to glycolysis.[22] Hypoxia-inducible factors-1α (HIF-1α) performs as a mediator in hypoxic effects and glucose utilization.[23,24] In well-oxygenated cells, prolyl hydroxylase (PHD) hydroxylates HIF-1α in a consistent way.[25] HIF-1α stabilization enhances the WNT/β-catenin signaling pathway, leading to angiogenesis as well as osteogenesis.[26,27] Our previous reports demonstrated that hBD3, combined with AuNPs and lithium-calcium-silicate bioceramics, could activate the WNT/β-catenin pathway in hPDLCs osteogenesis.[28,29] Therefore, metabolic reprogramming-related HIF-1α/WNT signaling may participate in hPDLC osteogenic differentiation.

For the better expansion of stem cells, hypoxic chambers have been utilized to achieve and maintain a hypoxic state for cell culture in a long term, which is time-consuming and costly. Therefore, hypoxia-mimicking agents, for example, deferoxamine (DFO) and cobalt chloride ($CoCl_2$), aiming to obtain better cell expansion in a much easier way, have been put into practice.[30] Like $CoCl_2$, MXene also consumes oxygen;[31] thus, we speculated that MXene application could mimic the hypoxic environment in hPDLCs. The purpose of this study is to detect the function of WNT/HIF-1α-mediated metabolic reprogramming in hPDLCs and periodontal tissue defects treated with MXene.

2. Experimental Section

2.1. The Synthesis of the $Ti_3C_2T_x$ Dispersion

The minimal intensive layer delamination method was applied to synthesize $Ti_3C_2T_x$ in an etching solution with 1.6 g LiF (99%, Sigma-Aldrich Pty, Ltd.) and 20 mL of 9 M HCl. One gram of Ti_3AlC_2 powder (Carbon-Ukraine, Ltd., particle size < 40 µm) was gently poured into the etchant at 35 °C, to be thoroughly mixed with constant stirring for 24 h. The washing was performed using the centrifugation method on a Centrifuge-5810R (Eppendorf AG, Germany). The acidic suspension was washed with water under the centrifugation of 3500 rpm (10 min per cycle). Notably, when the pH approached 6, self-delamination occurred after shaking by hand. Subsequently, at the rate of 1500 rpm for 1 h, multilayer $Ti_3C_2T_x$ and unreacted Ti_3AlC_2 were centrifugated. The dark green supernatant of the delaminated $Ti_3C_2T_x$ was diluted to the desired concentration.

2.2. Molecular Dynamic Simulation

Gromacs package was used to carry out atomic molecular dynamic simulations at constant temperature at 300 K and pressure at 1 bar. In the first step, the $Ti_3C_2T_x$ at 5 nm in length was positioned at the top of the membrane solvated with water molecules with an area of 10 × 10 nm^2 and total lipids of ≈275. The TIP3P model was used to model water molecules, 1-palmitoyl-2-oleyol-phosphatidylcholine (POPC) lipid was modeled with AMBER14 force field,[32] and $Ti_3C_2T_x$ was modeled with previously optimized parameters.[33] When simulating in the SETTLE algorithm, bond angles, and lengths of water molecules were constrained.[34] While all other bond lengths were in constraint of the LINCS algorithm,[35] which allowed a step at 2 fs. Each simulation was optimized, thermalized, equilibrated, and continuously run in the isothermal–isobaric (NPT) ensemble. The total simulation time was 3–4 µs. The Molecular Mechanics-Poisson–Boltzmann Solvent-Accessible surface area (MMPBSA) method was adopted to measure the binding free energies of the protein–protein complexes.[36] The structure of the WNT-Frizzled signaling complex was obtained from recent work done by Hirai et al.[37] The $Ti_3C_2T_x$ was positioned near the complex ≈5–7 nm to the center, the simulation setup was similar to that described previously for the $Ti_3C_2T_x$ cell membrane system, the total simulation time was ≈2 µs, and the final production trajectory for MMPBSA analysis was 20 ns with a time step of 2.0 fs in integration method.

2.3. Cell Culture

We cultured primary hPDLCs (ScienCell Research Laboratories, Catalogue #2630, Carlsbad, USA) in Dulbecco's modified eagle medium (DMEM) with 10% foetal bovine serum (FBS) (ScienCell) and 1% penicillin/streptomycin (HyClone, Logan, USA). Only cells within passages 2 and 6 were used in the following studies of hPDLCs osteogenic differentiation in the osteogenic induction medium: α-MEM (Gibco, Grand Island, NY), 10% fetal bovine serum (HyClone, South Logan, UT), 1% penicillin-streptomycin solution (containing 10 000 U mL^{-1}, and 10 000 µg mL^{-1} penicillin-streptomycin) (HyClone, South Logan, UT), 10–7 mol L^{-1} dexamethasone (Sigma-Aldrich, St. Louis, MO), 50 mg L^{-1} vitamin C (Sigma-Aldrich, St. Louis, MO) and 10 × 10^{-3} M β-glycerophosphate disodium (Sigma-Aldrich, St. Louis, MO).

2.4. Biocompatibility and Cytotoxicity of $Ti_3C_2T_x$

To analyze the biocompatibility and cytotoxicity of $Ti_3C_2T_x$ by hPDLCs, cells were incubated with $Ti_3C_2T_x$ at five different concentrations (30, 60, 90, 180, 360 mg L^{-1}), and hPDLCs without $Ti_3C_2T_x$ were the control group.

$Ti_3C_2T_x$ at different concentrations mentioned above was added to coculture with the cells. After incubation for 3, 6, 12, 24, 48, and 72 h, CCK-8 assays (Dojindo Molecular Technologies, Tokyo, Japan) were used to study hPDLCs cell proliferation rate. A SpectraMax M3 microplate reader (Molecular Devices, Sunnyvale, USA) was utilized to examine the absorbance at 450 nm wavelength.

© 2021 The Authors. Advanced Healthcare Materials published by Wiley-VCH GmbH

21922659, 2021, 22, Downloaded from https://advanced.onlinelibrary.wiley.com/doi/10.1002/adhm.202101215 by National Institutes Of Health Malaysia, Wiley Online Library on [01/02/2026]. See the Terms and Conditions (https://onlinelibrary.wiley.com/terms-and-conditions) on Wiley Online Library for rules of use; OA articles are governed by the applicable Creative Commons License

To evaluate the effect of $Ti_3C_2T_x$ on hPDLC apoptosis, cells were marked with annexin V and propidium iodide (PI) and then inspected under the flow cytometry A nonspecific fluorescent dye, 2′,7′-dichlorofluorescein diacetate (DCF-DA, Sigma-Aldrich) was applied to monitor the intracellular reactive oxygen species (ROS) level after cells were cocultured with $Ti_3C_2T_x$ for 12 and 24 h. Incubated with DCF−DA solution for 30 min, cells were treated with 100 µL Triton-X solution at 0.1% (Sigma-Aldrich) v/v. The fluorescence set at 485 and 520 nm were analyzed by a fluorescence microplate reader (SpectraMax M3, Molecular Devices, Sunnyvale, USA). Additionally, ROS expression was further confirmed by confocal microscopic imaging at 24 h.

2.5. Alkaline Phosphatase (ALP) Analysis

An ALP assay kit (Beyotime Institute of Biotechnology, Shanghai, China) was utilized to perform the assays of ALP activity after cells were incubated with $Ti_3C_2T_x$ for 7 d. The cells were lysed and centrifugated at 1.5×10^4 rpm, followed by the addition of the supernatant and chromogenic agent solution (50 µL, respectively). After 20 min incubation at 37 °C in the dark, the plates were analyzed by a SpectraMax M3 microplate reader to measure the absorbance at 405 nm. A BCIP/NBT ALP staining kit (Beyotime Institute of Biotechnology, Shanghai, China) was utilized to perform ALP staining on the 7th day according to the manufacturer's instructions.

2.6. ARS Staining

As the description in the ALP activity experiment, cells were treated and stained by ARS staining solution (Sigma-Aldrich) for ARS staining. An inverted optical microscope (Olympus IMT-2, Tokyo, Japan) was used to examine the plates and a digital camera (Canon EOS 70D, USA) was used to take photos. Maintained with cetylpyridinium chloride (Sigma-Aldrich), ARS was quantified by measuring the absorbance at the wavelength of 562 nm.

2.7. Western Blot

We detected changes in osteogenic proteins, autophagic proteins and WNT/β-catenin signaling pathway-associated proteins by western blot. hPDLCs were cultured with osteogenic differentiation medium with $Ti_3C_2T_x$ (60 mg L^{-1}) alone or cotreated with 10 µg mL^{-1} ICG-001 (Selleckchem, TX), a WNT/β-catenin signaling pathway inhibitor, for 7 d. The primary antibodies were produced by Abcam (ALP, OPN, HK-2, PFKB3, and SDH), Proteintech (Axin2 and HIF-1α) and Cell Signaling Technology (RUNX2 and β-catenin), with internal control of β-actin (Proteintech, China). A Tanon-5200 chemiluminescent imaging system (Tanon, Shanghai, China) was selected to apply for observing the proteins after 1 h incubation of the membranes with secondary antibody.

2.8. Real-Time qPCR

Real-time quantitative polymerase chain reaction (qPCR) was utilized to measure osteogenic genes, autophagic genes, and WNT/β-catenin signaling pathway-associated genes. hPDLCs were treated as described above for western blot assay. The comparative $2^{-\Delta\Delta Ct}$ method was applied, with the primers shown in **Table 1** and the samples normalized to β-actin.

2.9. Metabolite Profiling by Gas Chromatography-Mass Spectrometry (GC-MS)

hPDLCs at a density of 1×10^7 per well were seeded a 15 cm dish and incubated overnight. Subsequently, the hPDLCs were treated with $Ti_3C_2T_x$ at a concentration of 60 mg L^{-1} for 7 d. Then, cellular metabolites were extracted to be analyzed by GC-MS, and an Agilent 7890B gas chromatography system, combined with an Agilent 5977A MSD system (Agilent Technologies, Inc., USA) was adopted to measure the derivatized samples.

2.10. Oxygen Consumption Rate (OCR) Measurement by Seahorse XF96

hPDLCs were treated with $Ti_3C_2T_x$ at a concentration of 60 mg L^{-1} for 7 d. An XF96 Extracellular Flux Analyzer and the XF cell stress test kit (Agilent Technologies, Inc., USA) were used to measure the OCRs. First, 1×10^{-3} м oligomycin was added to the cells to interfere with the ATP-coupled respiration, then 1.5×10^{-3} м carbonyl cyanide 4-trifluoromethoxyhenylhydrazone (FCCP) was added to distribute the respiratory chain, followed by 0.5×10^{-3} м antimycin A with 0.5×10^{-3} м rotenone to inhibit mitochondrial respiration.

2.11. In Vivo Cytocompatibility Analysis

All the animal experiments abided by the regulations and guidelines for institutional animal care issued by Nanjing University, Nanjing, China (No. SYXK 2009-0017). 7-week-old male SD rats at the sample size of 18 were involved and injected with pentobarbital sodium (Merck Millipore) to be anesthetized, and the establishment of periodontal defects was successful as previously described.[38] Next, $Ti_3C_2T_x$ at different concentrations (0, 60, and 180 mg L^{-1}) was randomly implanted into the rat periodontal defects. Twenty-four days later, rats were overdosedly injected with anesthetics for euthanasia. Samples of heart, liver, spline, lung, kidney, and dental and periodontal tissues were harvested for light microscopy. Thin sections (4 µm) of tissues were applied with hematoxylin and eosin (H&E) and Masson–Goldner trichrome staining before histopathological examination. A light microscope (MZ3000 Micros, Austria) was used to examine all of the slides were examined with and compared with the control tissue sections.

2.12. Histopathological Analysis of the Jaw

Twenty male SD rats at 7 weeks of age were involved in this part of examination. Then, periodontal fenestration defects were made in the suitable jaws as previously described. A total of 10^6 hPDLCs, treated with or without 60 mg L^{-1} $Ti_3C_2T_x$ for 24 h, were

© 2021 The Authors. Advanced Healthcare Materials published by Wiley-VCH GmbH

21922659, 2021, 22, Downloaded from https://advanced.onlinelibrary.wiley.com/doi/10.1002/adhm.202101215 by National Institutes Of Health Malaysia, Wiley Online Library on [01/02/2026]. See the Terms and Conditions (https://onlinelibrary.wiley.com/terms-and-conditions) on Wiley Online Library for rules of use; OA articles are governed by the applicable Creative Commons License

Table 1. Primer sequences.

Primer	Forward primer sequence (5′-3′)	Reverse primer sequence (5′-3′)
RUNX2	GGAGTGGACGAGGCAAGAGTTT	AGCTTCTGTCTGTGCCTTCTGG
ALP	GACCCTTGACCCCCACAAT	GCTCGTACTGCATGTCCCCT
OPN	CTGAACGCGCCTTCTGATTG	ACATCGGAATGCTCATTGCTCT
OCN	GCAAAGGTGCAGCCTTTGTG	GGAAAGAAGGGTGCCTGGAGAG
HIF-1α	GGGAGATCTGGGGACAGGAGGATCGCC	GGGAAGCTCATAAAAAACTTTAGATTC
β-catenin	CTTCACCTGACAGATCCAAGTC	CCTTCCATCCCTTCCTGTTTAG
Axin2	TACACTCCTTATTGGGCGATCA	TTGGCTACTCGTAAAGTTTTGGT
HK2	GAGCCACCACTCACCCTACT	CCAGGCATTCGGCAATGTG
PFKFB3	TTGGCGTCCCCACAAAAGT	AGTTGTAGGAGCTGTACTGCTT
SDHa	CAGCATGTGTTACCAAGCTGT	GGTGTCGTAGAAATGCCACCT
SDHb	ACCTTCCGAAGATCATGCAGA	GTGCAAGCTAGAGTGTTGCCT
β-actin	GGGACCTGACTGACTACCTC	ACTCGTCATACTCCTGCTTG

collected with Matrigel Matrix (Corning) and placed into the defects randomly. The rats were split into three groups ($N = 6$ per group) according to the different treatments: 1) the Ctrl group, performed a sham operation; 2) the PDLC group, with hPDLCs; and 3) the MX group, with hPDLCs cultured with 60 mg L^{-1} $Ti_3C_2T_x$. The extra two blank rats were used as normal controls to observe normal periodontal tissues. Twenty-four days later, the maxillary jaws were hemisected, and collected the right half ($N =$ 6) for micro-CT (Bruker Micro-CT, Kontich, Billerica, MA) and histological analysis to evaluate the results of the repair of periodontal defects. The tissue sections (4 µm) were arranged for H&E, Goldner's trichrome and TRAP staining for histological analysis. A camera installed on a computer (1×71, Olympus Co.) was applied to evaluate the sections, and the examiner was blind to the previous procedure. Image-Pro Plus 6.0 software was used to measure the newly formed alveolar bone and calculate the total number of osteoclasts which were TRAP-positive multinucleated.

2.13. Statistical Analysis

Data were demonstrated in the value of the mean ± standard deviation (SD). Student's *t*-test or one-way ANOVA were applied in accordance with the conditions to determine significant differences after normalization evaluation. This experiment regarded 2-tailed P value <0.05 as statistically significant ($N = 5$). SPSS 20.0 was used to process all the data.

3. Results

3.1. Synthesis and Characterization of $Ti_3C_2T_x$

Herein, $Ti_3C_2T_x$ flakes were fabricated by etching Al atoms from bulk Ti_3AlC_2 with a mixture of lithium fluoride (LiF) and hydrochloric acid (HCl).[39] During washing, self-delamination occurred after shaking by hand when the pH approached 6. The exfoliated $Ti_3C_2T_x$ nanoflakes formed a stable dispersion. **Figure 1**a illustrates the crystal structures of Ti_3AlC_2 and $Ti_3C_2T_x$. Delamination and the removal of aluminum were confirmed by the downshifting of the (002) peak and the disappearance of the aluminum peak at 2 θ of 39° in the X-ray diffraction (XRD) spectra (Figure 1b). Scanning electron microscopy (SEM) images confirmed that the block Ti_3AlC_2 (Figure 1c) was exfoliated into $Ti_3C_2T_x$ nanoflakes (Figure 1d). As shown in Figure 1a, $Ti_3C_2T_x$ could exfoliate into monolayers.[40] To confirm this, we observed the $Ti_3C_2T_x$ nanoflakes by atomic force microscopy (AFM), and the data showed a thickness of 1.5 nm (Figure 1e).

3.2. Interaction between $Ti_3C_2T_x$ and hPDLCs

To further elucidate the interaction between the nanomaterial and cell membrane, we carried out simulations of $Ti_3C_2T_x$ in the absence and presence of a lipid bilayer composed of POPC (Figure 1g). We focused on an atomic model of $Ti_3C_2T_x$ in the vicinity of lipid bilayers that can spontaneously insert into the core of the lipid bilayer efficiently (Figure 1f). Once embedded into lipid bilayers, $Ti_3C_2T_x$, which mimicked protein channels, enhanced the transportation of small solute molecules. This indicates that the $Ti_3C_2T_x$ has the potential to realize the function of synthetic ion channels.[41] We also investigated the influence of $Ti_3C_2T_x$ in lipid bilayers. In contrast to the widely acknowledged view that $Ti_3C_2T_x$ could be embedded into the lipid core without disrupting the membrane, we proved that $Ti_3C_2T_x$ could destabilize the lipid bilayer in its proximity and facilitate a small nanodomain. Due to the strong hydrophobicity, we observed spontaneous coverage of monolayer lipids around $Ti_3C_2T_x$, and the lipids were clearly oriented toward the surface of the $Ti_3C_2T_x$, forming a hydrophilic shield (Figure 1i). In addition to the density profile, the vicinity of the $Ti_3C_2T_x$ is featured by a decreased lipid deuterium order parameter, $S_{CD} = \frac{1}{2} \langle 3\cos^2\theta_i - 1 \rangle$, where θ_i is the angle between the bilayer normal and the vector that joins the respective carbon atom C_i to its H (deuterium) atom, while angular brackets refer to the average of total C–H bonds i in each alkyl chain of lipid (Figure 1h). The rearrangement of lipids would significantly change the surface properties of $Ti_3C_2T_x$, while forming hydrophilic pores in the membrane by a first-order phase transition, leading to translocation, as previously discussed.[42]

© 2021 The Authors. Advanced Healthcare Materials published by Wiley-VCH GmbH

21922659, 2021, 22, Downloaded from https://advanced.onlinelibrary.wiley.com/doi/10.1002/adhm.202101215 by National Institutes Of Health Malaysia, Wiley Online Library on [01/02/2026]. See the Terms and Conditions (https://onlinelibrary.wiley.com/terms-and-conditions) on Wiley Online Library for rules of use; OA articles are governed by the applicable Creative Commons License

21922659, 2021, 22, Downloaded from https://advanced.onlinelibrary.wiley.com/doi/10.1002/adhm.202101215 by National Institutes Of Health Malaysia, Wiley Online Library on [01/02/2026]. See the Terms and Conditions (https://onlinelibrary.wiley.com/terms-and-conditions) on Wiley Online Library for rules of use; OA articles are governed by the applicable Creative Commons License

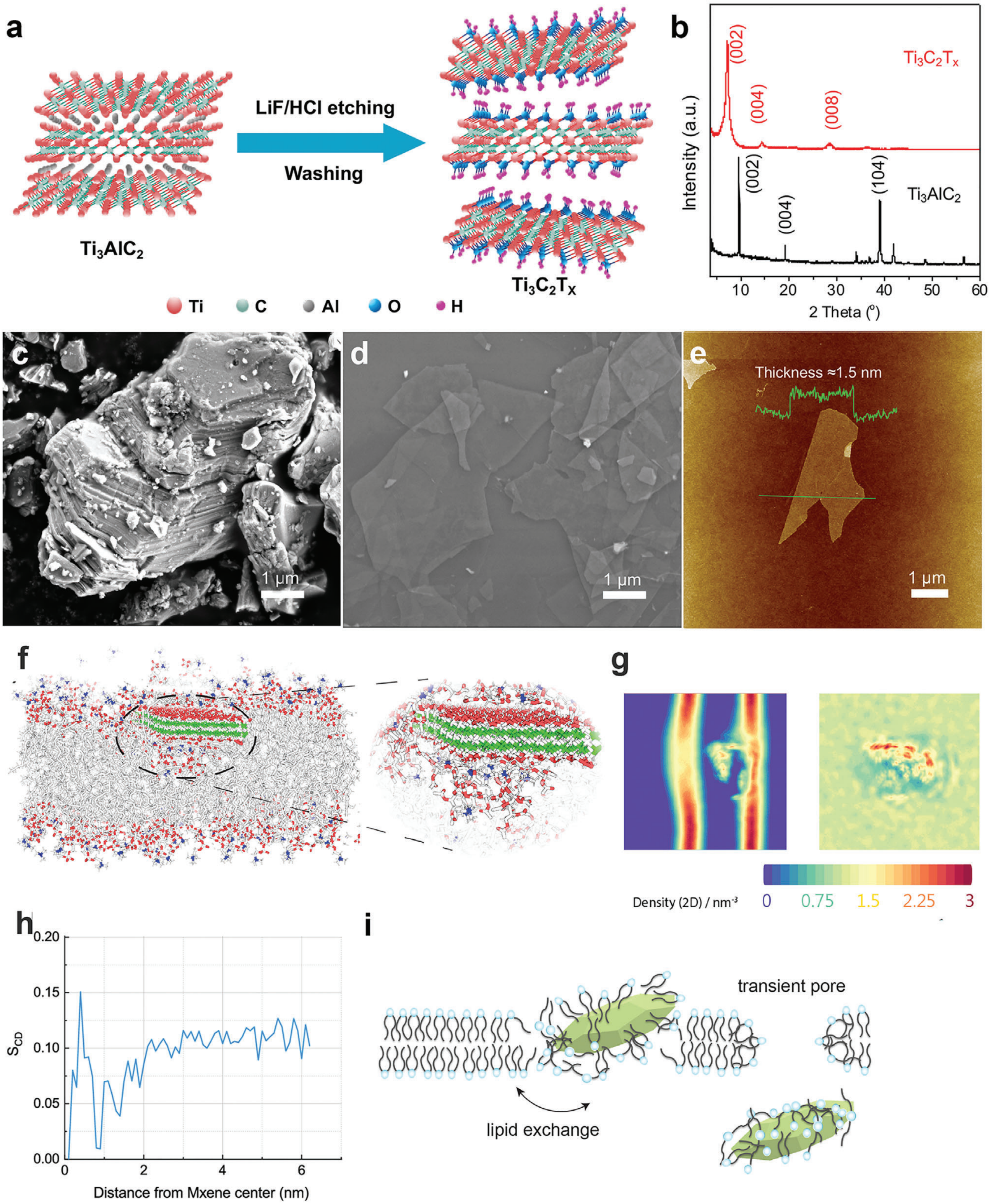

Figure 1. Generation of the 2D nanomaterial $Ti_3C_2T_x$. a) Schematic illustration of the synthesis of $Ti_3C_2T_x$ nanoflakes. b) XRD patterns of Ti_3AlC_2 and $Ti_3C_2T_x$. SEM images of c) Ti_3AlC_2 particles and d) $Ti_3C_2T_x$ flakes on silicon wafers. e) AFM image of single $Ti_3C_2T_x$ flakes; the inset shows the thickness profile along the green line. f) Schematic illustration of $Ti_3C_2T_x$ embedded in the membrane core from molecular dynamics simulation. g) A 2D density map of the PC head group of the POPC lipid bilayer. h) Orientational order parameter S_{CD} of the bilayer with embedded $Ti_3C_2T_x$. i) Schematic illustration of trapping and translocation of $Ti_3C_2T_x$ through transient pores.

 © 2021 The Authors. Advanced Healthcare Materials published by Wiley-VCH GmbH

The uptake and localization of the $Ti_3C_2T_X$ in hPDLCs were demonstrated by transmission electron microscope (TEM). In the $Ti_3C_2T_X$-treated groups (Figure S1, Supporting Information), $Ti_3C_2T_X$ appeared in the cytoplasm. Furthermore, it was noted that no $Ti_3C_2T_X$ were found in the mitochondria or nucleus.

Based on the previous discussion, employing a multiscale theoretical approach, we discovered that $Ti_3C_2T_x$ could be rapidly embedded in the cell membrane, destabilize the proximity region, and even rearrange the lipids in its vicinity and open pores, triggering spontaneous translocation into the cell.

3.3. In Vitro Biocompatibility Analysis

To evaluate the biological activity, we incubated $Ti_3C_2T_x$ at a range of different concentrations (0, 30, 60, 90, 180, and 360 mg L^{-1}) with hPDLCs for a time gradient. The effects on the proliferation of hPDLCs were determined by CCK-8 and flow cytometric analyses. No significant inhibition of proliferation was noted in the CCK-8 assays, revealing good biocompatibility of $Ti_3C_2T_x$ with hPDLCs (**Figure 2**a). Moreover, as shown in the flow cytometric analysis, the ratio of apoptotic cells after treatment with the nanoparticles for 24 h was approximately fivefold higher with $Ti_3C_2T_x$ at a concentration of 360 mg L^{-1} than that with a concentration of 0 mg L^{-1} (Figure 2b,c). No significant increase in the ratio was detectable with the other four concentrations.

ROS are critical in cell metabolism and survival.[43] Nanoparticles were shown to enhance the generation of ROS in cells. To further elucidate the cellular changes of $Ti_3C_2T_x$, we monitored the level of intracellular ROS in the presence of a nonspecific fluorescent dye. No obvious ROS was observed after a 12 h incubation with $Ti_3C_2T_x$ at concentrations less than 90 mg L^{-1} (Figure 2d,e); thus, we chose concentrations of 30, 60, and 90 mg L^{-1} for the following osteogenic analysis.

3.4. Osteogenic Effect of $Ti_3C_2T_x$ on hPDLCs

After the biosecurity was confirmed, $Ti_3C_2T_x$ was expected to induce osteogenic differentiation in hPDLCs and promote periodontal tissue repair. To verify this hypothesis, we incubated hPDLCs with $Ti_3C_2T_x$ at concentrations of 0, 30, 60, and 90 mg L^{-1} and then stained them with an ALP assay kit (Beyotime Institute of Biotechnology, Shanghai, China) (**Figure 3**a). ALP activity was further detected. In particular, 60 mg L^{-1} $Ti_3C_2T_x$ demonstrated the most significant increase in the ALP activity on day 7 among all the groups (Figure 3c). More excitingly, after incubation for 21 d, alizarin red S (ARS) staining showed that $Ti_3C_2T_x$ (60 mg L^{-1}) displayed the most significant enhancement of mineralized nodule formation (Figure 3b).

Moreover, RT-PCR and western blotting were conducted to analyze osteoblast-related molecular expression in the hPDLCs treated with $Ti_3C_2T_x$ in different groups. The expression of ALP, RUNX2, OPN, and OCN in the 60 mg L^{-1} groups were considerably strengthened at day 7, implying that $Ti_3C_2T_x$ at a concentration of 60 mg L^{-1} showed the strongest promotion of the osteogenic differentiation of hPDLCs in vitro (Figure 3d; and Figure S2, Supporting Information). PCR of ALP and RUNX2 were elevated by $Ti_3C_2T_x$ at a concentration of 30, 60, and 90 mg L^{-1}, and reached a peak analysis revealed that the expression at 60 mg L^{-1} (Figure 3e). Both OPN and OCN were significantly increased in hPDLCs treated by 60 mg L^{-1} MXene. It is widely accepted that Runx2, as a critical transcription factor at the initial stage of osteogenesis, triggers other genes expressions, such as the ALP and collagen type I (COL I) early on and the later OPN and osteocalcin (OCN).[44,45] OPN is a negative regulator of osteoblast precursors, whose expression level is low at the beginning of osteogenesis but gradually grows with time.[46] In the present research, OPN was substantially upregulated on day 7, suggesting OPN could become a regulator in the negative feedback for osteogenic differentiation.

3.5. The Application of $Ti_3C_2T_x$ Enhanced the HIF-1α/WNT Signaling Pathway

Encouraged by the excellent osteogenic effect of $Ti_3C_2T_x$, we assessed the impact on HIF-1α/WNT signaling in vitro. The WNT/β-catenin pathway is a critical regulator of bone development.[47] Additionally, rigorous analyses indicated that β-catenin can accelerate the HIF-1-mediated transcription by binding to the promoter region of HIF-1 target genes, thereby enhancing cell survival and its adaptation to hypoxia.[48] HIF-1α plays a crucial role in tissue regeneration, which could become a possible solution in bettering cell therapeutic outcomes.[49,50]

To validate the effects of $Ti_3C_2T_x$ on the intrinsic atomic interaction and binding affinity, we performed all atom-based MD simulations for the WNT-Frizzled (an essential receptor in WNT signaling) signaling complex[51] with and without $Ti_3C_2T_x$ (**Figure 4**a). The binding energy $\Delta G_{\text{binding}}$ calculated from the MM-PBSA calculation showed a 20% increase in binding affinity with $Ti_3C_2T_x$ (−291.961 kJ mol^{-1}) compared to that without treatment (−241.565 kJ mol^{-1}). The binding energy decomposition analysis further showed that the significant contribution was the van der Waals interaction, suggesting that the signaling pathway could be enhanced by hydrophobic interactions in the presence of $Ti_3C_2T_x$. Then, we utilized western blot and PCR analyses to confirm the effect of $Ti_3C_2T_x$ on HIF-1α and related molecules in the WNT/β-catenin pathway. As shown in Figure 4b,c, HIF-1α together with β-catenin were notably upregulated on day 7 in the presence of $Ti_3C_2T_x$ (60 mg L^{-1}). Moreover, the expression of Axin2, a WNT signaling negative regulator, was enhanced on day 7 but showed no significant change with $Ti_3C_2T_x$. To verify whether $Ti_3C_2T_x$ accelerated osteogenesis via the HIF-1α/WNT signaling pathway, we applied ICG-001, a nonspecific inhibitor of the WNT/β-catenin signaling pathway, to hPDLCs. Our results revealed that the ALP activity and calcium deposition of the hPDLCs downregulated by ICG-001 were reversed by $Ti_3C_2T_x$ to different degrees (Figure 4d–f). PCR and western blot data showed that HIF-1α, β-catenin, and Axin2 were inhibited by ICG-001, while $Ti_3C_2T_x$ stimulation partially rescued the expression of these three proteins (Figure 4g,h).

In short, these results indicated that the presence of $Ti_3C_2T_x$ in hPDLCs could activate the HIF-1α/WNT signaling pathway in an Axin2-independent manner.

© 2021 The Authors. Advanced Healthcare Materials published by Wiley-VCH GmbH

21922659, 2021, 22, Downloaded from https://advanced.onlinelibrary.wiley.com/doi/10.1002/adhm.202101215 by National Institutes Of Health Malaysia, Wiley Online Library on [01/02/2026]. See the Terms and Conditions (https://onlinelibrary.wiley.com/terms-and-conditions) on Wiley Online Library for rules of use; OA articles are governed by the applicable Creative Commons License

21922659, 2021, 22, Downloaded from https://advanced.onlinelibrary.wiley.com/doi/10.1002/adhm.202101215 by National Institutes Of Health Malaysia, Wiley Online Library on [01/02/2026]. See the Terms and Conditions (https://onlinelibrary.wiley.com/terms-and-conditions) on Wiley Online Library for rules of use; OA articles are governed by the applicable Creative Commons License

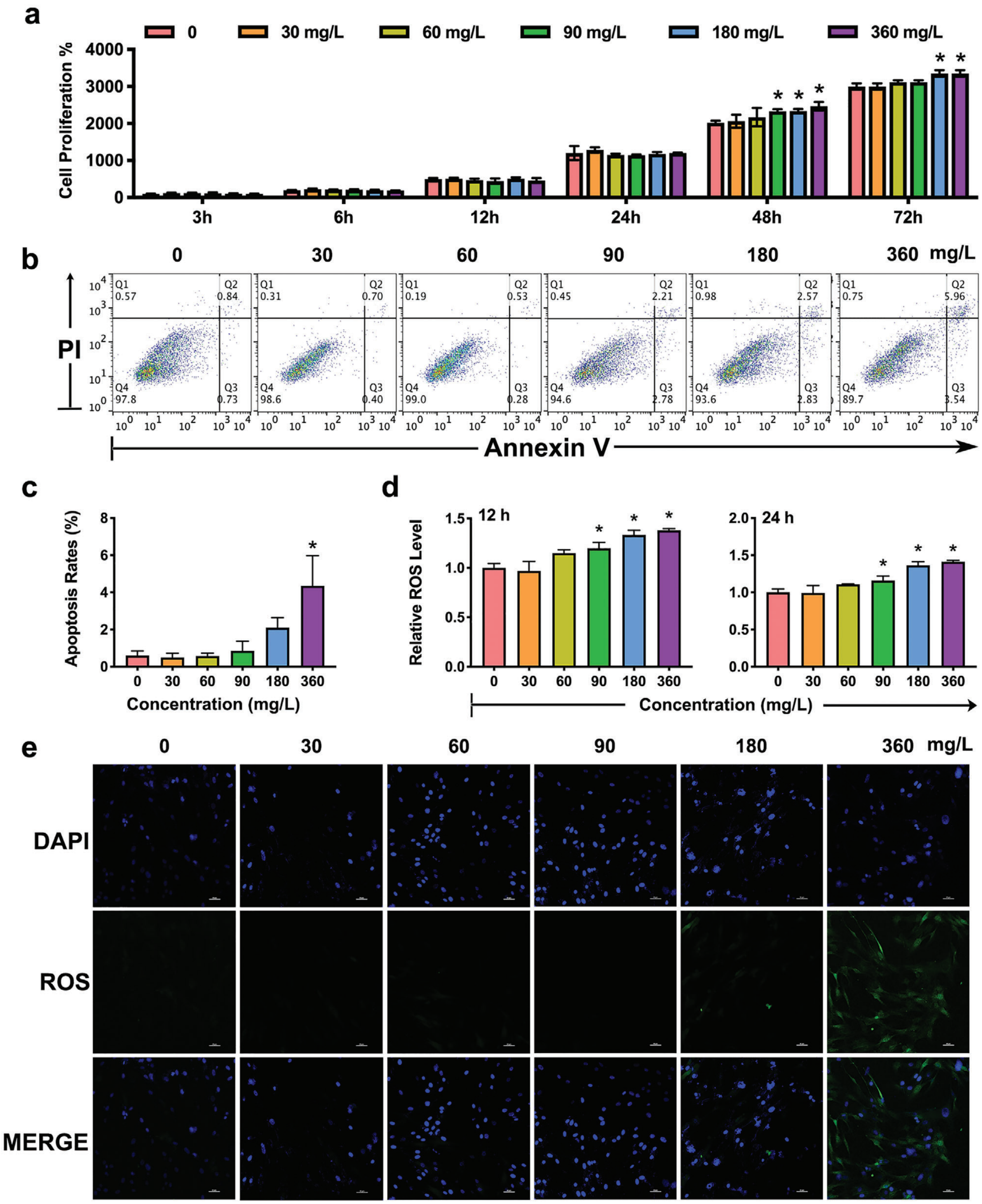

Figure 2. The biological activity of $Ti_3C_2T_x$ nanoparticles. hPDLCs were treated with $Ti_3C_2T_x$ (0, 30, 60, 90, 180, and 360 mg L^{-1}). a) The effects of $Ti_3C_2T_x$ on the proliferation of hPDLCs were measured using CCK-8 assays at 3, 6, 12, 24, 48, and 72 h. The apoptosis of the hPDLCs stimulated by $Ti_3C_2T_x$ was detected by flow cytometry b) and calculated c). d) Production of ROS during incubation with various concentrations of the 2D nanoparticles. e) Images were taken by a fluorescence microscope. $^*P < 0.05$, $^{**}P < 0.01$, $^{***}P < 0.001$, $^{****}P < 0.0001$.

© 2021 The Authors. Advanced Healthcare Materials published by Wiley-VCH GmbH

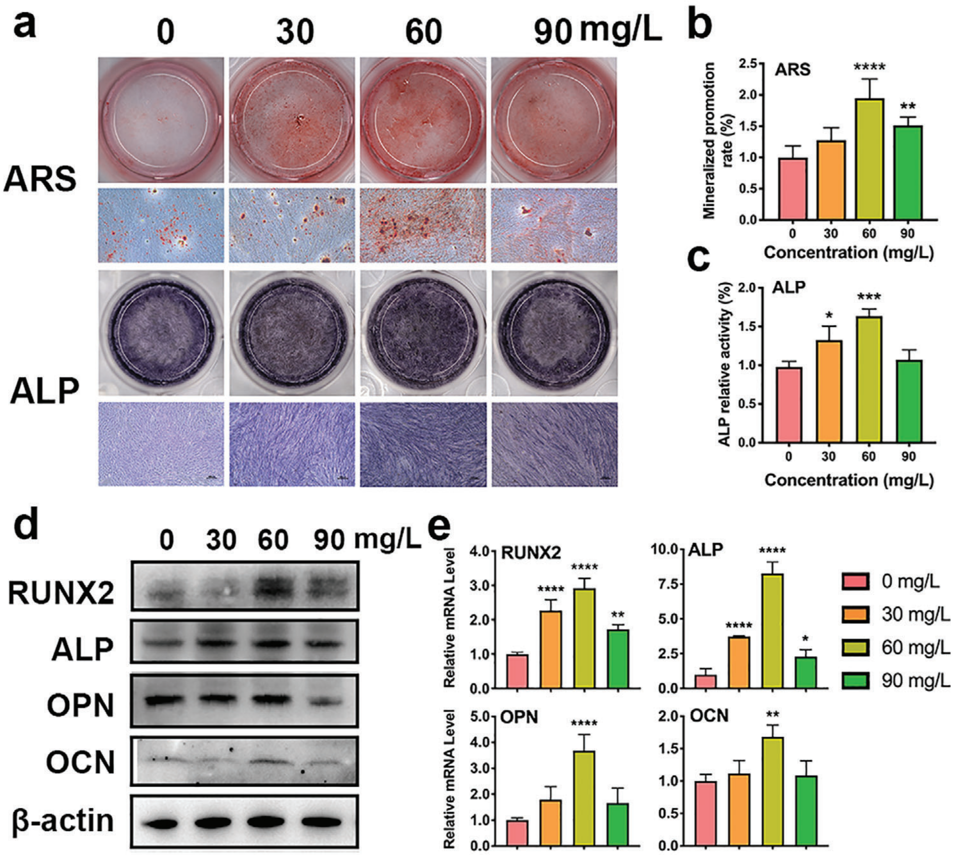

Figure 3. Effects of $Ti_3C_2T_x$ on hPDLC osteogenic differentiation. a) ALP staining on day 7 and mineralized nodules stained with ARS on day 21. b) Mineralized nodule levels on day 21. c) ALP activity levels on day 7. d) Relative protein levels of the osteogenic factors were determined by western blots on day 7. e) Real-time PCR analysis of the relative mRNA levels on day 5. $^{*}P < 0.05$, $^{**}P < 0.01$, $^{***}P < 0.001$, $^{****}P < 0.0001$.

3.6. Metabolic Reprogramming in hPDLCs

The function of HIF-1 is to balance oxygen supply and demand and coordinate metabolic reprogramming at the transcriptional level.[24] Since $Ti_3C_2T_x$ enhanced HIF-1α expression, we examined the influence of $Ti_3C_2T_x$ on cell metabolism. GC-MS was applied to explore overall variations in hPDLCs cultured with or without 60 mg L^{-1} $Ti_3C_2T_x$. $Ti_3C_2T_x$ triggered a metabolic change in hPDLCs after 7 d of treatment. Based on fold change values larger than 1.5 and a *t*-test value at the 95% level, metabolites for the heatmap (**Figure 5**a) were selected from the volcano results (Figure 5c). We found that 4 metabolites were downregulated, while 29 metabolites, such as succinate and glucose-6-phosphate, were upregulated. Major sources of metabolite variability were identified by principal component analysis (PCA) (Figure 5b). Additionally, we explored pathway mapping of KEGG website and revealed the influence of $Ti_3C_2T_x$ on multiple pathways (Figure 5d), among which the pathway with the most improvement was ABC transporters.

The cellular bioenergetics were analyzed in hPDLCs stimulated with or without $Ti_3C_2T_x$ through the OCR to detect mitochondrial respiration by a Seahorse Bioanalyzer. $Ti_3C_2T_x$ application significantly affected the hPDLCs OCR (Figure 5e). Although basal respiration and ATP production were not affected, the spare capacity and maximum respiration were reduced in cells treated with $Ti_3C_2T_x$, which indicated the energy production shifting from mitochondrial oxidative phosphorylation to glycolysis in the cytosol (Figure S3, Supporting Information). Based on the profound changes of metabolic profiling revealed in carbohydrate compounds, we then explored the effect of $Ti_3C_2T_x$ application on critical genes involved in hypoxia and glycolysis (Figure 5f,g). HIF-1α can induce various genes involved in glycolysis and osteogenesis.[52] First, the alteration of HIF-1α revealed an elevation of the levels of HIF-1α in the cells treated by $Ti_3C_2T_x$. What's more, $Ti_3C_2T_x$ altered genes in oxidative phosphorylation and glycolysis by increasing transcription of hexokinase 2 (HK2) and 6-phosphofructo-2-kinase/fructose-2,6-biphosphatase 3 (PFKB3) and decreasing succinate dehydrogenase (SDH). Oxidation of succinate into fumarate was metabolized by SDH,[53] and impaired SDH activity explained the succinate accumulation in the cytosol.

3.7. In Vivo Periodontal Regeneration Experiment

The histocompatibility of $Ti_3C_2T_x$ was tested by histopathological assessment of the heart, kidneys, liver, lungs, and spleen (Figure S4, Supporting Information). Revealed by histopathological

© 2021 The Authors. Advanced Healthcare Materials published by Wiley-VCH GmbH

21922659, 2021, 22, Downloaded from https://advanced.onlinelibrary.wiley.com/doi/10.1002/adhm.202101215 by National Institutes Of Health Malaysia, Wiley Online Library on [01/02/2026]. See the Terms and Conditions (https://onlinelibrary.wiley.com/terms-and-conditions) on Wiley Online Library for rules of use; OA articles are governed by the applicable Creative Commons License

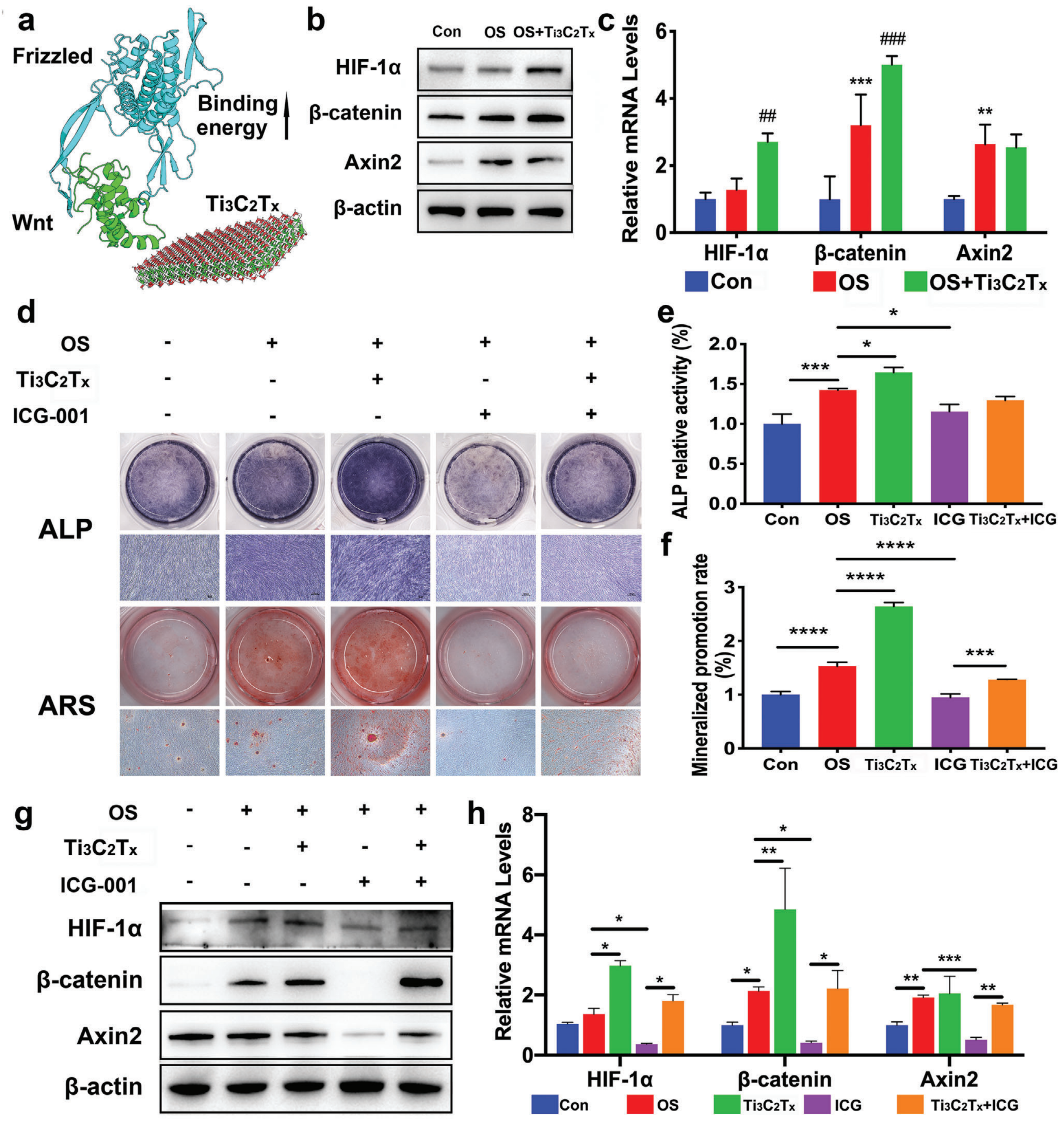

Figure 4. Effects of the HIF-1α/WNT signaling pathway on osteogenic differentiation of hPDLCs induced by $Ti_3C_2T_x$. hPDLCs were treated with $Ti_3C_2T_x$ (60 mg L^{-1}). a) Binding energy difference estimated in the presence of $Ti_3C_2T_x$ in the vicinity of the Wnt-Frizzled complex. b) Relative protein levels of HIF-1α, β-catenin, and Axin2 determined by western blots on day 7. c) The mRNA expression of HIF-1α, β-catenin, and Axin2 on day 5 analyzed by real-time PCR. For analysis of the role of the WNT/β-catenin signaling pathway in the osteogenic differentiation promoted by $Ti_3C_2T_x$, hPDLCs were incubated with ICG-001 (10×10^{-6} M). d) ALP staining on day 7 and mineralized nodules stained with ARS on day 21. e) ALP activity levels on day 7. f) Mineralized nodule levels on day 21. ICG-001 blocked the protein expression of HIF-1α, β-catenin, and Axin2 on day 7 g) and the mRNA expression h) on day 5. $^{*}P < 0.05$, $^{**}P < 0.01$, $^{***}P < 0.001$, $^{****}P < 0.0001$, compared with the Con group or the corresponding group. $^{\#\#}P < 0.01$, $^{\#\#\#}P < 0.001$, compared with the PDLC group.

 © 2021 The Authors. Advanced Healthcare Materials published by Wiley-VCH GmbH

21922659, 2021, 22, Downloaded from https://advanced.onlinelibrary.wiley.com/doi/10.1002/adhm.202101215 by National Institutes Of Health Malaysia, Wiley Online Library on [01/02/2026]. See the Terms and Conditions (https://onlinelibrary.wiley.com/terms-and-conditions) on Wiley Online Library for rules of use; OA articles are governed by the applicable Creative Commons License

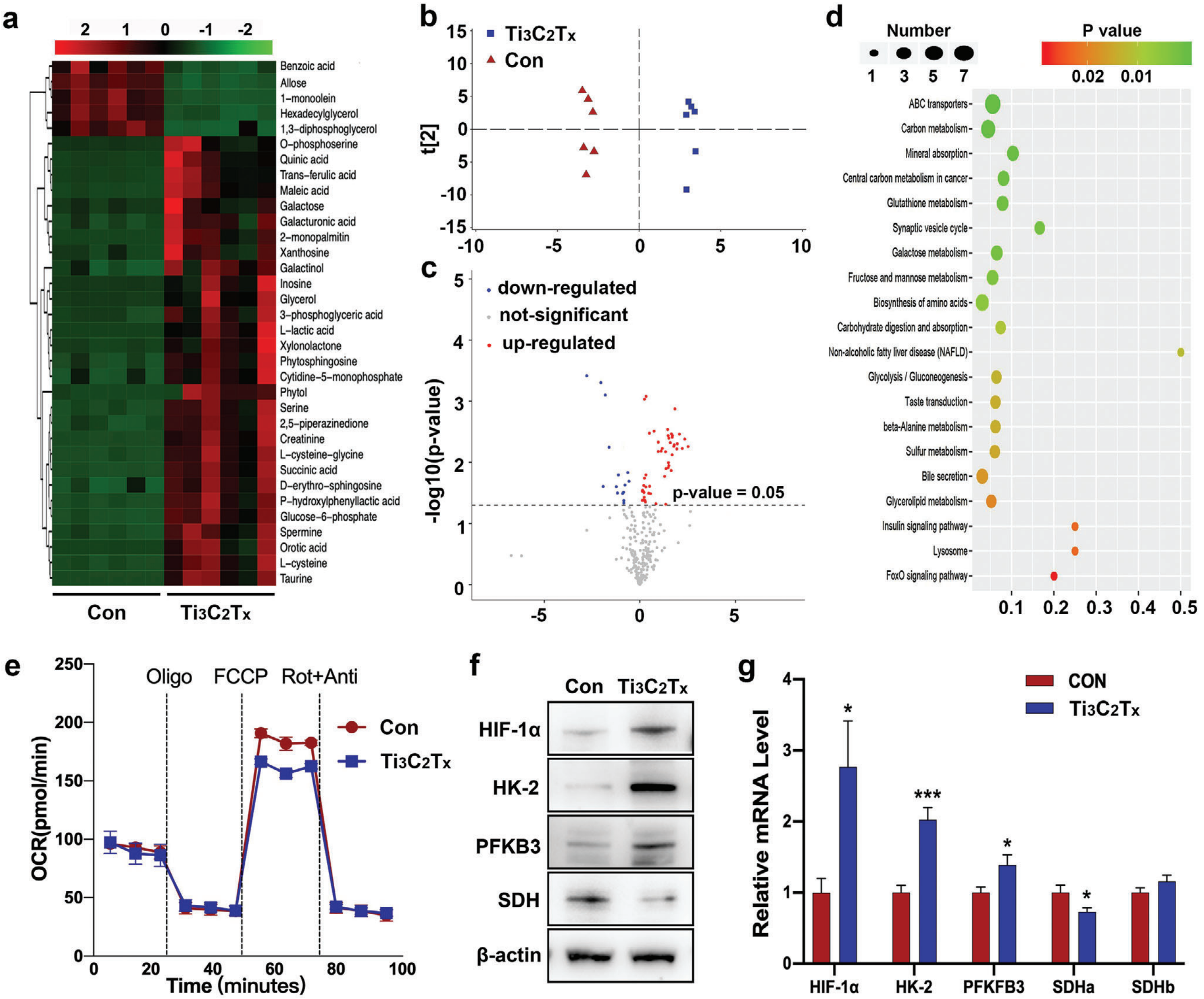

Figure 5. $Ti_3C_2T_x$ triggered metabolic reprogramming in hPDLCs. GC-MS was utilized to measure changes in the metabolites of the cells. a) Heatmap of metabolites that were differentially regulated. Red, upregulation; green, downregulation. b) PCA plot based on intracellular metabolites. c) Volcano plot of metabolites that were differentially regulated. Red, upregulation; blue, downregulation; gray, not significant. d) Pathway enrichment analysis by KEGG. e) Basal OCR and real-time changes in the OCR in the PDLSCs stimulated with $Ti_3C_2T_x$ for 7 d. Cell cultures were treated sequentially with oligomycin, FCCP, and rotenone + antimycin A. f) Protein levels of the indicated genes were determined by western blots. g) Transcription of genes at 5 d was measured by qPCR. *$P < 0.05$, ***$P < 0.001$, versus the control group.

analysis, exposure to different doses of $Ti_3C_2T_x$ had no apparent negative impact on the examined tissues. Comparable results were shown between all groups.

We were inspired by the efficient effects of $Ti_3C_2T_x$ in hPDLCs osteogenic promotion and good biosafety and decided to further demonstrate the ability of $Ti_3C_2T_x$ in tissue repairment in vivo. According to the guide of the flowchart, hPDLCs were treated with 60 mg L^{-1} $Ti_3C_2T_x$ in vitro 24 h before applied to rat experimental periodontal defects (**Figure 6**a). Based on microcomputed tomography (micro-CT) analysis, we observed loss of bone quality caused by periodontal tissue resection (Figure 6b–d). Notably, after implantation for 24 d, compared with that of the hPDLCs alone, an increase of nearly onefold in new bone area was observed in the MX group, indicating that $Ti_3C_2T_x$ further improved periodontal repair enhanced by hPDLCs (Figure 6e–h). Additionally, TRAP staining analysis showed that the number of osteoclasts experienced some noticeable declines under the influence of $Ti_3C_2T_x$ (Figure 6f). Immunohistochemical staining to assess the repair of alveolar defects in the three groups on day 24 showed more RUNX2-, HIF-1α-, and β-catenin-positive markers in the $Ti_3C_2T_x$ groups than in the other groups (Figure 6g,h). The $Ti_3C_2T_x$-treated hPDLCs improved the capacity to form new alveolar bone through HIF-1α and β-catenin upregulation in periodontal tissues. These results also suggested that the effects of $Ti_3C_2T_x$ on hPDLC was consistent between the studies on in vitro and in vivo.

© 2021 The Authors. Advanced Healthcare Materials published by Wiley-VCH GmbH

21922659, 2021, 22, Downloaded from https://advanced.onlinelibrary.wiley.com/doi/10.1002/adhm.202101215 by National Institutes Of Health Malaysia, Wiley Online Library on [01/02/2026]. See the Terms and Conditions (https://onlinelibrary.wiley.com/terms-and-conditions) on Wiley Online Library for rules of use; OA articles are governed by the applicable Creative Commons License

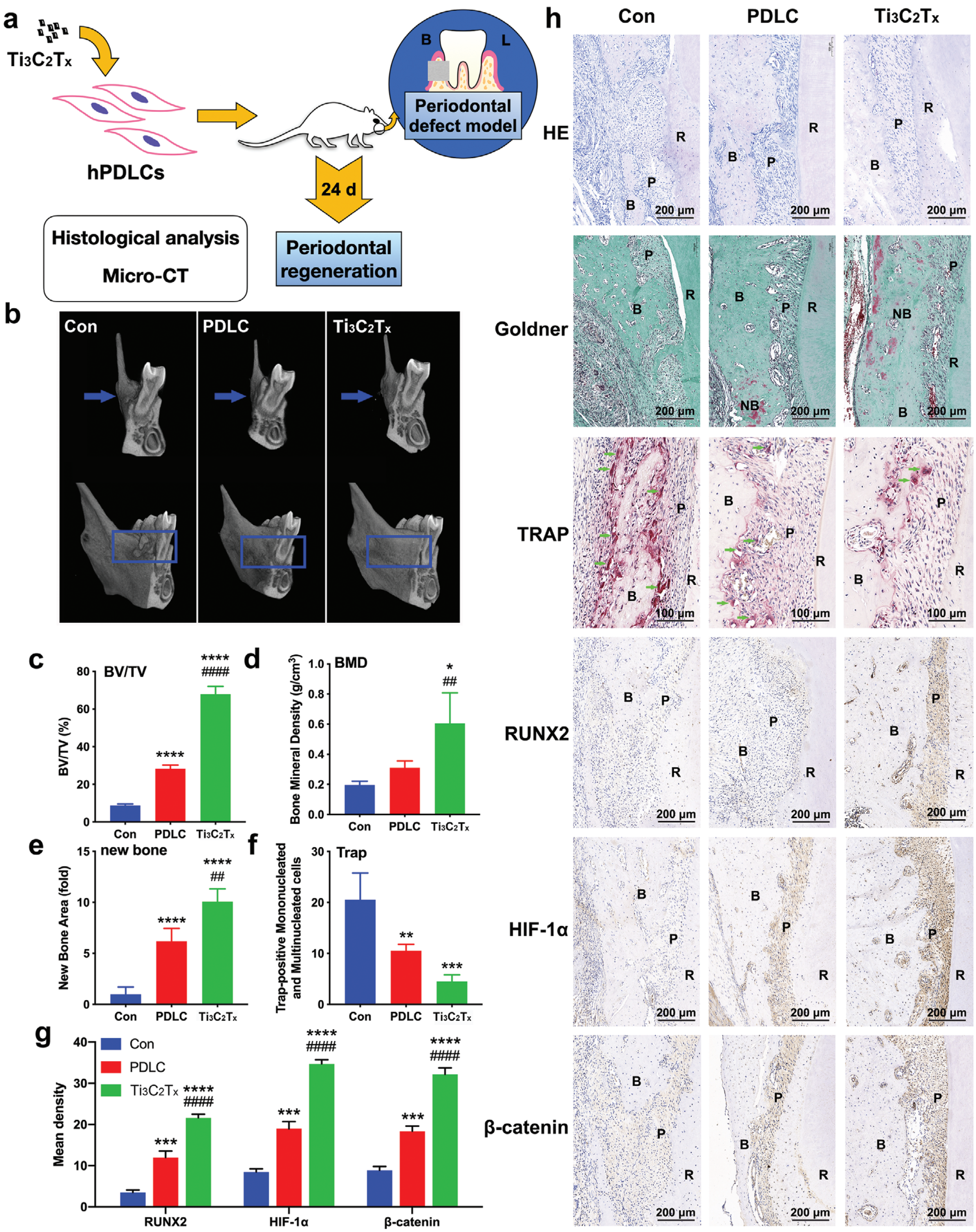

Figure 6. $Ti_3C_2T_x$-treated hPDLCs showed better in vitro periodontal regeneration potential in rats. a) Illustration of the animal experiment. b) Mesiodistal section images and 3D reconstructed digitized images of the maxillary first molars analyzed by micro-CT. BV/TV c) and bone mineral density d) of the defects. Newly formed alveolar bone e) and osteoclast quantity analysis f). g) The corresponding quantitative analysis of RUNX2, HIF-1*α*, and *β*-catenin expression in immunohistochemical staining images. h) H&E, Goldner's Masson trichrome, and TRAP staining (green arrows indicate osteoblasts) and immunohistochemical staining images of periodontal defects. **P* < 0.05, ***P* < 0.01, ****P* < 0.001, *****P* < 0.0001, compared with Con. ##*P* < 0.01, ####*P* < 0.0001, compared with PDLC. B = alveolar bone; P = periodontal ligament; R = root; NB = newly formed alveolar bone.

© 2021 The Authors. Advanced Healthcare Materials published by Wiley-VCH GmbH

21922659, 2021, 22, Downloaded from https://advanced.onlinelibrary.wiley.com/doi/10.1002/adhm.202101215 by National Institutes Of Health Malaysia, Wiley Online Library on [01/02/2026]. See the Terms and Conditions (https://onlinelibrary.wiley.com/terms-and-conditions) on Wiley Online Library for rules of use; OA articles are governed by the applicable Creative Commons License

4. Discussion

The repair of periodontal tissue has long been known to underlie the resolution of severe periodontitis, which has few satisfactory treatments.[54] These principles have become increasingly important given the severe decrease in quality of life and substantial disease burden. During periodontium regeneration, hPDLCs function as progenitors. We hypothesized that 2D nanoflake $Ti_3C_2T_x$ would yield essential insights into hPDLC osteogenic differentiation and periodontal defect repair. Here, we showed that these analyses converged on a single important cell signaling pathway. We proposed that $Ti_3C_2T_x$ application activated the metabolic reprogramming-related HIF-1α/WNT signaling pathway, switches cell metabolism to glycolysis, and supported tissue regeneration. Our data suggested that targeting this pathway with $Ti_3C_2T_x$ could enhance the resolution of tissue destruction resulting from severe periodontitis.

2D nanomaterials and the related composite materials have been used to gain a promoting effect in tissue regeneration based on stem cells. Graphene, the first known 2D material, and its derivatives, like graphene oxide (GO), showed the ability to influence stem cell proliferation and differentiation.[55] Since attempted for the first time, MXene has always been compared with graphene. The two kinds of 2D materials have some similarities. For example, both are carbon-based 2D ultrathin nanoflakes, represented by the suffix "ene" in the names.[56] However, MXene has advantages when compared with its predecessor. First, it is demonstrated that $Ti_3C_2T_x$ had a good biocapacity at higher concentrations. According to the report by Akhavan et al., GO with large sizes was used to induce cytotoxicity at a concentration of 100 μg mL^{-1}, while the smaller sizes of GO did harm to cells even at a low concentration of 1 μg mL^{-1}.[57] In our study, $Ti_3C_2T_x$ exhibited good biological security below 180 μg mL^{-1}. Another claimed a bisafety concertation up to 500 μg mL^{-1} for normal cells.[58] Moreover, $Ti_3C_2T_x$ is highly selective against tumor cells than normal ones, making it a potential 2D material for tissue repair after tumor operation.[19] In addition, $Ti_3C_2T_x$ is fully surface functionalized, allowing it to dissolve in solvent easily and combine with other materials.[59] In fact, the ideal graphene is highly chemically inert because of its crystal lattice, resulting its difficulty to dissolve in water and combine with other biomaterials.[60] This disadvantage drove the attempts of GO, the oxidized form of graphene. Reported by Wei et al., 0.1 μg mL^{-1} GO had a significant positive effect on the cell proliferation of bone marrow-derived mesenchymal stem cells. However, a reduction of cell proliferation by GO was observed at the concentration between 1 and 10μg mL^{-1} .[61] In addition, GO could promote the differentiation of several lines of stem cell, including mouse embryonic stem cells toward dopamine neural cells,[62] induced pluripotent stem cells toward endodermal cells (where graphene showed inhibiting effects).[63] The potential mechanism might be the bind between the surface functional groups of GO and stem cell surface receptors. Although MXene also exhibited effects on differentiation of stem cells, they might go through different pathways from graphene. Particularly, it was indicated that Ti-based species, released by the interaction of water and oxygen with MXene, could promote the growth of cells.[64] This study demonstrated that $Ti_3C_2T_x$ could interact with cell membrane, enhance glucose metabolism, and induce metabolic reconfiguration in hPDLCs. Another by Guo et al. demonstrated that $Ti_3C_2T_x$ together with electric stimulation changed the metabolic activities in neural stem cells, by decreasing glucose and increasing lactic acid.[65]

It is known that biological membranes are far more complex in structure as compared with lipid bilayers, containing large number of proteins (integral and peripheral), complex coting of polymeric sugars with glycosylation, besides, lateral heterogeneities, underlying cytoskeleton and transmembrane asymmetry could further complicate the question.[66] Due to the abbe diffraction limit, it is quite challenging to access the full molecular-scale details in aqueous solution by experiment alone, computational modeling soon has become an essential tool to investigate lipid membranes and bridge the gap between experiments and theory.[67,68] However, it is barely possible to reconstruct cell membranes fully using computer technology, due to the availability of computer resources limits the scales that can be accessed. Thus, a variety of heterogeneous and idealized biological membrane models have been adopted in recent investigations to represent the lipid membranes and their interactions with nanomaterials,[69,70] among which POPC is one of the most abundant lipids in biological membrane, and has been widely applied as lipid bilayer basement.[71–74] Thus, in the current study, all-atom classical molecular dynamics (MD) simulations were utilized to investigate the interaction between $Ti_3C_2T_x$ and the bilayer structure on POPC. We stress the fact that to compare the interaction of nanomaterial with cell membrane between our theoretical studies with real biological membrane quantitatively is debatable which requires further comparison to fill the gap, but gives a powerful way to qualitatively derive the interaction through simulation techniques. It would indeed be great to simulate the real biological cell membrane with atomistic models and confront them with currently available experimental data. It is also likely that future studies will sooner or later bring new information that will challenge the theoretical community for developing more accurate biological membrane models.

Our analysis revealed that fully embedded $Ti_3C_2T_x$ could reorganize lipids and result in the formation of pores with little energy cost, and finally leave the bilayer. This mechanism is very similar to the previously discovered mechanism of spontaneous translocation of nanoparticles through a first-order phase transition.[42] Once embedded, $Ti_3C_2T_x$ behaves similar to synthetic ion channels, which can transport common physiological ions, such as Na^+ and K^+.[41] Bioelectric signals, originating from the flux of positive and negative ions across membranes, regulate the proliferation and differentiation of stem cells.[75] Shields et al. used gene microarray to detect osteoblastic differentiation of mesenchymal stem cells induced by BMP2 and found that Na+, K+ -ATPase (NKA) was observed to be significantly upregulated.[76] Moreover, NKA regulates intracellular calcium concentration and upregulates osteogenic gene expression.[77–79] In this study, we proposed that MXene, which mimics protein channels, could facilitate flux of ions such as Na+/K+. Therefore, the regulation of Na+/K+ flux might be a potential contribution on osteogenesis enhanced by MXene, which needs further study. $Ti_3C_2T_x$ destabilizes the lipid bilayer in its proximity and facilitates a small nanodomain. Due to the limitations of current methods, nanodomains cannot be observed directly in living cells and are associated with a known

© 2021 The Authors. Advanced Healthcare Materials published by Wiley-VCH GmbH
21922659, 2021, 22, Downloaded from https://advanced.onlinelibrary.wiley.com/doi/10.1002/adhm.202101215 by National Institutes Of Health Malaysia, Wiley Online Library on [01/02/2026]. See the Terms and Conditions (https://onlinelibrary.wiley.com/terms-and-conditions) on Wiley Online Library for rules of use; OA articles are governed by the applicable Creative Commons License

mechanism to organize membrane receptors into signaling microclusters.[80,81] We speculated that the nanodomain caused by $Ti_3C_2T_x$ modulates Frizzled proteins, could make it easier for Wnts to bind with its receptor; thus, the WNT pathway was activated, and β-catenin accumulation was enhanced.

For the purpose of assessing the biotoxicity of $Ti_3C_2T_x$, we analyzed the proliferation of hPDLCs incubated with 30, 60, 90, 180, and 360 mg L^{-1} $Ti_3C_2T_x$. The results showed that $Ti_3C_2T_x$ was nontoxic to hPDLCs in this concentration range. This finding is consistent with previous reports, demonstrating that $Ti_3C_2T_x$, both $Ti_3C_2T_X$ and Ti_2NT_X, showed lower toxic effects against normal cells than cancerous ones.[58,82] We observed that $Ti_3C_2T_x$ at low concentrations, in agreement with previous reports, displayed no induction of apoptosis.[58,83] Therefore, we came to the conclusion that $Ti_3C_2T_x$ at low concentrations had good biocompatibility.

ROS was slightly induced by $Ti_3C_2T_x$ at a concentration of 60 mg L^{-1}. Although numerous studies have indicated that ROS can result in cell damage, apoptosis, necrosis, and inflammatory responses,[43,84,85] multiple targets affected by ROS have been gradually discovered in recent years.[86] ROS plays a physiological role in signaling instead of becoming cytotoxic and resulting from too strong signal, as with any signaling mechanism. For example, increased ROS from hypoxic mitochondria undertakes significant responsibility in the prevention of the HIF-1α degradation, the main transcription factor in the adaption of cells to the hypoxic state.[87] Low-level ROS that is typically observed with osteogenesis results from the energetic demands of differentiation.[88] ROS has rarely been reported to participate in the osteogenic effect of $Ti_3C_2T_x$, and further research is needed.

Metabolic alterations are tightly associated with proliferation and osteogenesis, and the effect on osteogenic differentiation remains in dispute.[89–93] Such controversies may be on account of different culture systems, such as donor species, cell types, hypoxic severity, and methods of induction. In our study, $Ti_3C_2T_x$ application enhanced succinate accumulation. Succinate can function as an intracellular messenger to stabilize HIF-1α in hPDLCs, thus altering gene expression and generating a state of pseudohypoxia.[94] The activation of glycolysis is necessary for high proliferation in a pseudohypoxic state at the beginning stages of osteogenesis and enhanced bone matrix protein biosynthesis afterward. Moreover, further studies may help elucidate metabolic regulation, including interactions between HIF-1α and the Notch pathway.

As hPDLCs have been used as the potential seed cells for osteogenesis, the current study focused on the effects of $Ti_3C_2T_x$ on hPDLCs osteogenic differentiation. In the previous studies, HIF-1α accumulation was related to the upregulation of osteogenesis genes, such as COL I, RUNX2, ALP, and OCN.[24,95] The studies showed that key transcription factor in osteogenesis, RUNX2 induced the expression of ALP and COL I at an early stage and OPN and OCN at a late stage.[44,45] ALP can be used to assess early-phase osteogenesis.[96] Late-stage osteogenic differentiation is marked by mineralized nodule deposition, which is usually detected by ARS.[97] The results of ALP activity and ARS staining indicated that $Ti_3C_2T_x$ exhibited the ability to enhance osteogenic differentiation on hPDLCs at not only the early but also the late stage of osteogenic differentiation. It is reported that even though OPN acts as a negative feedback factor in the regulation of osteogenic differentiation, an increase in its mRNA expression remained till 2 weeks.[98] While the expression of OCN, remarking osteogenesis, rises to a peak in the late stage.[99] In the present study, all tested osteogenic mRNA and protein were increased by $Ti_3C_2T_x$, indicating the osteogenic-differentiation-induction ability of $Ti_3C_2T_x$ on hPDLCs.

In this study, we demonstrated that MXene nanoflakes could enhance hPDLCs osteogenesis and bone regeneration. There is little study that MXene nanoflakes are used to promote differentiation of other lineages. A recent study found that together with electrical stimulation, $Ti_3C_2T_x$ MXene films could promote neural differentiation of neural stem cells cultured on it.[65] Increased angiogenesis was observed in quail eggs incubated in hypoxic conditions, comparing with eggs in normoxia.[100] HIF-1α plays a critical role in the regulation of angiogenesis.[101] In this study, we demonstrated that MXene nanoflakes could enhance hPDLCs osteogenesis and bone regeneration, which revealed that $Ti_3C_2T_x$ could provide a hypoxic environment and stabilize HIF-1α. Therefore, we hypothesize $Ti_3C_2T_x$ might have some effects in angiogenesis, which need further investigation.

5. Conclusion

In summary, this study investigated the application of $Ti_3C_2T_x$ in periodontal regeneration. We demonstrated that $Ti_3C_2T_x$ could promote hPDLC osteogenic differentiation, thus improving periodontal tissue regeneration. The potential mechanism may involve the modulation of the metabolic reprogramming-related HIF-1α/WNT signaling pathway. These findings imply that $Ti_3C_2T_x$ might be a potential therapeutic option in periodontal tissue engineering and treatment of periodontitis.

Supporting Information

Supporting Information is available from the Wiley Online Library or from the author.

Acknowledgements

The authors would like to thank the colleagues, including Dr. Changxing Chen, Binyan Luo, Yuezhen Huang, Dr. Shuang Zhang, Wenqi Su, and Yunhe Zhao, for their generous assistance with the animal experiments. This study was supported by the National Natural Science Foundation Project (Nos. 81771078 and 11804151), the Nanjing Clinical Research Center for Oral Diseases (No. 2019060009), and the Jiangsu Provincial Medical Innovation Team (No. CXTDB2017014).

Author Contributions

D.C. was responsible for the design of the study, the acquisition and analysis of data, and the draft of the work. N.K. carried out the representative experiments and interpretation of data. L.D. assisted in the design of the study and the revision of the manuscript. Y.G. performed atomic molecular dynamics simulations and assisted in the design of the study. W.Y. provided $Ti_3C_2T_x$ and assisted in the design of the study. F.Y. made substantial contributions to the conception of the work and financed this research.

Conflict of Interest

The authors declare no conflict of interest.

© 2021 The Authors. Advanced Healthcare Materials published by Wiley-VCH GmbH

21922659, 2021, 22, Downloaded from https://advanced.onlinelibrary.wiley.com/doi/10.1002/adhm.202101215 by National Institutes Of Health Malaysia, Wiley Online Library on [01/02/2026]. See the Terms and Conditions (https://onlinelibrary.wiley.com/terms-and-conditions) on Wiley Online Library for rules of use; OA articles are governed by the applicable Creative Commons License

21922659, 2021, 22, Downloaded from https://advanced.onlinelibrary.wiley.com/doi/10.1002/adhm.202101215 by National Institutes Of Health Malaysia, Wiley Online Library on [01/02/2026]. See the Terms and Conditions (https://onlinelibrary.wiley.com/terms-and-conditions) on Wiley Online Library for rules of use; OA articles are governed by the applicable Creative Commons License

Data Availability Statement

Research data are not shared.

Keywords

hPDLCs, metabolic reprogramming, molecular dynamics simulations, MXenes, periodontal tissue engineering

Received: June 21, 2021
Revised: September 8, 2021
Published online: September 29, 2021

[1] D. F. Kinane, P. G. Stathopoulou, P. N. Papapanou, *Nat. Rev. Dis. Primers* **2017**, *3*, 17038.
[2] S. A. Tassi, N. Z. Sergio, M. Y. O. Misawa, C. C. Villar, *J. Periodontal Res.* **2017**, *52*, 793.
[3] M. S. Tonetti, S. Jepsen, L. Jin, J. Otomo-Corgel, *J. Clin. Periodontol.* **2017**, *44*, 456.
[4] A. Kumar, J. Mahendra, S. Samuel, J. Govindraj, T. Loganathan, Y. Vashum, L. Mahendra, T. Krishnamoorthy, *J. Periodontol.* **2019**, *90*, 61.
[5] B.-M. Seo, M. Miura, S. Gronthos, P. Mark Bartold, S. Batouli, J. Brahim, M. Young, P. Gehron Robey, C. Y. Wang, S. Shi, *Lancet* **2004**, *364*, 149.
[6] J. A. D'Errico, H. Ouyang, J. E. Berry, R. L. MacNeil, C. Strayhorn, M. J. Imperiale, N. L. Harris, H. Goldberg, M. J. Somerman, *Bone* **1999**, *25*, 39.
[7] P. Lekic, J. Rojas, C. Birek, H. Tenenbaum, C. A. McCulloch, *J. Periodontal Res.* **2001**, *36*, 71.
[8] J. Xiong, S. Gronthos, P. M. Bartold, *Periodontology 2000* **2013**, *63*, 217.
[9] T. Akizuki, S. Oda, M. Komaki, H. Tsuchioka, N. Kawakatsu, A. Kikuchi, M. Yamato, T. Okano, I. Ishikawa, *J. Periodontal Res.* **2005**, *40*, 245.
[10] T. Iwata, M. Yamato, Z. Zhang, S. Mukobata, K. Washio, T. Ando, J. Feijen, T. Okano, I. Ishikawa, *J. Clin. Periodontol.* **2010**, *37*, 1088.
[11] N. Gjorevski, N. Sachs, A. Manfrin, S. Giger, M. E. Bragina, P. Ordóñez-Morán, H. Clevers, M. P. Lutolf, *Nature* **2016**, *539*, 560.
[12] F. M. Chen, Y. Jin, *Tissue Eng., Part B* **2010**, *16*, 219.
[13] Z. Zhang, M. J. Gupte, X. Jin, P. X. Ma, *Adv. Funct. Mater.* **2015**, *21*, 350.
[14] H. Lin, X. Wang, L. Yu, Y. Chen, J. Shi, *Nano Lett.* **2017**, *17*, 384
[15] M. Ghidiu, M. R. Lukatskaya, M. Q. Zhao, Y. Gogotsi, M. W. Barsoum, *Nature* **2014**, *516*, 78.
[16] L. Li, X. Wang, H. Gu, G. Yao, H. Yu, Z. Tian, B. Li, L. Chen, *Small Methods* **2019**, *3*, 1900337.
[17] K. Rasool, M. Helal, A. Ali, C. E. Ren, Y. Gogotsi, K. A. Mahmoud, *ACS Nano* **2016**, *10*, 3674.
[18] C. Xing, S. Chen, X. Liang, Q. Liu, M. Qu, Q. Zou, J. Li, H. Tan, L. Liu, D. Fan, H. Zhang, *ACS Appl. Mater. Interfaces* **2018**, *10*, 27631.
[19] S. Pan, J. Yin, L. Yu, C. Zhang, Y. Zhu, Y. Gao, Y. Chen, *Adv. Sci.* **2020**, *7*, 1901511.
[20] J. G. Ryall, T. Cliff, S. Dalton, V. Sartorelli, *Cell Stem Cell* **2015**, *17*, 651.
[21] J. S. Harrison, P. Rameshwar, V. Chang, P. Bandari, *Blood* **2002**, *99*, 394.
[22] Y. S. Kida, T. Kawamura, Z. Wei, T. Sogo, S. Jacinto, A. Shigeno-Kamitsuji, E. Yoshihara, C. Liddle, J. R. Ecker, R. T. Yu, A. R. Atkins, M. Downes, R. M. Evans, *Cell Stem Cell* **2015**, *16*, 547.
[23] G. L. Wang, L. Semenza, *J. Biol. Chem.* **1994**, *269*, 23757.
[24] G. L. Semenza, *J. Clin. Invest.* **2013**, *123*, 3664.
[25] W. Luo, H. Hu, R. Chang, J. Zhong, M. Knabel, R. O'Meally, R. N. Cole, A. Pandey, G. L. Semenza, *Cell* **2011**, *145*, 732.
[26] M. Yang, C.-J. Li, X. Sun, Q. Guo, Y. Xiao, T. Su, M.-L. Tu, H. Peng, Q. Lu, Q. Liu, H.-B. He, T.-J. Jiang, M.-X. Lei, M. Wan, X. Cao, X.-H. Luo, *Nat. Commun.* **2017**, *8*.
[27] S. Stegen, I. Stockmans, K. Moermans, B. Thienpont, P. H. Maxwell, P. Carmeliet, G. Carmeliet, *Nat. Commun.* **2018**, *9*, 2557.
[28] J. Zhou, Y. Zhang, L. Li, H. Fu, W. Yang, F. Yan, *Int. J. Nanomed.* **2018**, *13*, 555.
[29] Q. Zhang, L. Chen, B. Chen, C. Chen, J. Chang, Y. Xiao, C. Wu, F. Yan, *Appl. Mater. Today* **2019**, *16*, 375.
[30] T. Osathanon, P. Vivatbutsiri, W. Sukarawan, W. Sriarj, P. Pavasant, S. Sooampon, *Arch. Oral Biol.* **2015**, *60*, 29.
[31] Y. Chae, S. J. Kim, S.-Y. Cho, J. Choi, K. Maleski, B.-J. Lee, H.-T. Jung, Y. Gogotsi, Y. Lee, C. W. Ahn, *Nanoscale* **2019**, *11*, 8387.
[32] M. Saeedimasine, A. Montanino, S. Kleiven, A. Villa, *Sci. Rep.* **2019**, *29*, 8000.
[33] E. G. Brandt, A. P. Lyubartsev, *J. Phys. Chem. C* **2015**, *119*, 18110.
[34] P. Eastman, V. S. Pande, *J. Comput. Chem.* **2010**, *31*, 1268.
[35] B. Hess, H. Bekker, H. J. C. Berendsen, J. G. J. Fraaije, *J. Chem. Theory Comput.* **2008**, *4*, 116.
[36] S. Genheden, U. Ryde, *Exp. Opin. Drug Discovery* **2015**, *10*, 449.
[37] H. Hirai, T. Arimori, K. Matoba, E. Mihara, J. Takagi **2019**, *26*, 372.
[38] C. Ni, J. Zhou, N. Kong, T. Bian, Y. Zhang, X. Huang, Y. Xiao, W. Yang, F. Yan, *Biomaterials* **2019**, *206*, 115.
[39] J. Zhang, S. Seyedin, S. Qin, Z. Wang, S. Moradi, F. Yang, P. A. Lynch, W. Yang, J. Liu, X. Wang, J. M. Razal, *Small* **2019**, *15*, 1804732.
[40] J. Zhang, N. Kong, S. Uzun, A. Levitt, S. Seyedin, P. A. Lynch, S. Qin, M. Han, W. Yang, J. Liu, X. Wang, Y. Gogotsi, J. M. Razal, *Adv. Mater.* **2020**, *32*, 2001093.
[41] G. W. Gokel, S. Negin, *Chem. Res.* **2013**, *46*, 2824.
[42] Y. Guo, E. Terazzi, R. Seemann, J. B. Fleury, V. A. Baulin, *Sci. Adv.* **2016**, *2*, 1600261.
[43] R. P. Nishanth, R. G. Jyotsna, J. J. Schlager, S. M. Hussain, P. Reddanna, *Nanotoxicology* **2011**, *5*, 502.
[44] T. Komori, *Cell Tissue Res.* **2009**, *339*, 189.
[45] S. Chava, S. Chennakesavulu, B. M. Gayatri, A. B. M. Reddy, *Cell Death Dis.* **2018**, *9*, 754.
[46] W. Huang, B. Carlsen, G. Rudkin, M. Berry, K. Ishida, D. T. Yamaguchi, T. A. Miller, *Bone* **2004**, *34*, 799.
[47] R. Baron, M. Kneissel, *Nat. Med.* **2013**, *19*, 179.
[48] A. Kaidi, A. C. Williams, C. Paraskeva, *Nat. Cell Biol.* **2007**, *9*, 210.
[49] C. Janda, L. Dang, C. You, J. Chang, W. Lau, Z. Zhong, K. Yan, O. Marecic, D. Siepe, X. Li, J. Moody, B. Williams, H. Clevers, J. Piehler, D. Baker, C. Kuo, K. C. Garcia, *Nature* **2017**, *545*, 234.
[50] C. Chen, Q. Tang, Y. Zhang, M. Dai, Y. Jiang, H. Wang, M. Yu, W. Jing, W. Tian, *Cell Proliferation* **2017**, *50*, 12363.
[51] T. Alam, H. Maruyama, C. Li, I. P. Strahil, M. Kunihiro, *Nat. Commun.* **2016**, *7*, 10388.
[52] N. S. Chandel, D. S. McClintock, C. E. Feliciano, T. M. Wood, J. A. Melendez, A. M. Rodriguez, P. T. Schumacker, *J. Biol. Chem.* **2000**, *275*, 25130.
[53] E. Mills, L. A. J. O'Neill, *Trends Cell Biol.* **2014**, *24*, 313.
[54] N. J. Kassebaum, E. Bernabé, M. Dahiya, B. Bhandari, C. J. Murray, W. Marcenes, *J. Dent. Res.* **2014**, *93*, 1045.
[55] D. H. Lee, H. D. Yun, E. D. Jung, J. H. Chu, Y. S. Nam, S. Song, S.-H. Seok, M. H. Song, S.-Y. Kwon, *ACS Appl. Mater. Interfaces* **2019**, *11*, 21069.
[56] S. R. Shin, Y. C. Li, H. L. Jang, P. Khoshakhlagh, M. Akbari, A. Nasajpour, Y. S. Zhang, A. Tamayol, A. Khademhosseini, *Adv. Drug Delivery Rev.* **2016**, *105*, 255.
[57] O. Akhavan, E. Ghaderi, A. Akhavan, *Biomaterials* **2012**, *33*, 8017.

© 2021 The Authors. Advanced Healthcare Materials published by Wiley-VCH GmbH

[58] A. M. Jastrzebska, A. Szuplewska, T. Wojciechowski, M. Chudy, W. Ziemkowska, L. Chlubny, A. Rozmyslowska, A. Olszyna, *J. Hazard. Mater.* **2017**, *339*, https://doi.org/10.1016/j.jhazmat.2017.06.004.
[59] R. Ibragimova, M. J. Puska, H.-P. Komsa, *ACS Nano* **2019**, *13*, 9171.
[60] G. Y. Xu, J. Abbott, L. Qin, K. Y. M. Yeung, Y. Song, H. Yoon, J. Kong, D. Ham, *Nat. Commun.* **2014**, *5*, 4866.
[61] C. Wei, Z. Liu, F. Jiang, B. Zeng, M. Huang, D. Yu, *Cell Proliferation* **2017**, *50*, 12367.
[62] D. Yang, T. Li, M. Xu, F. Gao, J. Yang, Z. Yang, W. Le, *Nanomedicine* **2014**, *9*, 2445.
[63] G. Y. Chen, D. W. Pang, S. M. Hwang, H. Y. Tuan, Y. C. Hu, *Biomaterials* **2012**, *33*, 418.
[64] Y. Bai, Y. Deng, Y. Zheng, Y. Li, R. Zhang, Y. Lv, Q. Zhao, S. Wei, *Mater. Sci. Eng., C* **2016**, *59*, 565.
[65] R. Guo, M. Xiao, W. Zhao, S. Zhou, Y. Hu, M. Liao, S. Wang, X. Yang, R. Chai, M. Tang, *Acta Biomater.* **2020**, S1742706120307492. https://doi.org/10.1016/j.actbio.2020.12.035.
[66] H. Watson, *Essays Biochem.* **2015**, *59*, 43.
[67] S. J. Marrink, V. Corradi, P. C. T. Souza, H. I. Ingolfsson, D. P. Tieleman, M. S. P. Sansom, *Chem. Rev.* **2019**, *119*, 6184.
[68] G. Enkavi, M. Javanainen, W. Kulig, T. Rog, I. Vattulainen, *Chem. Rev.* **2019**, *119*, 5607.
[69] S. H. Chen, J. M. Perez-Aguilar, R. Zhou, *Nanoscale* **2020**, *12*, 7939.
[70] Y. Liu, D. Zhang, Y. Zhang, Y. Tang, L. Xu, H. He, J. Wu, J. Zheng, *J. Phys. Chem. B* **2020**, *124*, 7830.
[71] T. S. Carpenter, D. A. Kirshner, E. Y. Lau, S. E. Wong, J. P. Nilmeier, F. C. Lightsteon, *Biophys. J.* **2014**, *107*, 630.
[72] A. Shamloo, M. Z. Pedram, H. Heidari, A. Alasty, J. Magn, *Magn. Mater.* **2016**, *410*, 187.
[73] N. Q. Thai, P. E. Theodorakis, M. S. Li, *J. Chem. Inf. Model.* **2020**, *60*, 3057.
[74] A. Goliaei, U. Adhikari, M. L. Berkowitz, *ACS Chem. Neurosci.* **2015**, *6*, 1296.
[75] A. D. Berendsen, E. L. Pinnow, A. Maeda, A. C. Brown, N. Mccartney-Francis, V. Kram, R. T. Owens, P. G. Robey, K. Holmbeck, L. F. de Castro, T. M. Kilts, M. F. Young, *Matrix Biol.* **2014**, *35*, 223.
[76] L. B. Shields, S. D. Raque, G. H. Glassman, M. Campbell, T. Vitaz, J. Harpring, C. B. Shields, *Spine* **2006**, *31*, 542.
[77] D. I. Lee, M. G. Klein, W. Zhu, R. P. Xiao, V. Gerzanich, K. Y. Xu, *Mol. Pharmacol.* **2009**, *75*, 774.
[78] K. Y. Xu, E. Takimoto, N. S. Fedarko, *Biochem. Biophys. Res. Commun.* **2006**, *349*, 582.
[79] Z. Tang, S. Chen, Y. Ni, R. Zhao, X. Zhu, X. Yang, X. Zhang, *Acta Biomater.* **2021**, *129*, 293.
[80] I. Levental, S. Veatch, *J. Mol. Biol.* **2016**, *428*, 4749.
[81] Y. Ma, E. Hinde, K. Gaus, *Essays Biochem.* **2015**, *57*, 93.
[82] A. Szuplewska, A. Rozmysowska-Wojciechowska, S. Poniak, T. Wojciechowski, A. Jastrzbska, *J. Nanabiotechnol.* **2019**, *17*, 114.
[83] F. Meng, M. Seredych, C. Chen, V. Gura, S. Mikhalovsky, S. Sandeman, G. Ingavle, T. Ozulumba, L. Miao, B. Anasori, Y. Gogotsi, *ACS Nano* **2018**, *12*, 10518.
[84] R. Zhou, A. S. Yazdi, P. Menu, J. Tschopp, *Nature* **2011**, *469*, 221.
[85] E. T. Chouchani, V. R. Pell, E. Gaude, D. Aksentijević, S. Y. Sundier, E. L. Robb, A. Logan, S. M. Nadtochiy, E. N. J. Ord, A. C. Smith, F. Eyassu, R. Shirley, C.-H. Hu, A. J. Dare, A. M. James, S. Rogatti, R. C. Hartley, S. Eaton, A. S. H. Costa, P. S. Brookes, S. M. Davidson, M. R. Duchen, K. Saeb-Parsy, M. J. Shattock, A. J. Robinson, L. M. Work, C. Frezza, T. Krieg, M. P. Murphy, *Nature* **2014**, *515*, 431.
[86] C. Nathan, A. Cunningham-Bussel, *Nat. Rev. Immunol.* **2013**, *13*, 349.
[87] J. K. Brunelle, E. L. Bell, N. M. Quesada, K. Vercauteren, V. Tiranti, M. Zeviani, R. C. Scarpulla, N. S. Chandel, *Cell Metab.* **2005**, *1*, 409.
[88] W. Oripiriyakul, M. Tsimbouri, P. Childs, P. Campsie, J. Wells, M. A. Fernandez-Yague, K. Burgess, K. E. Tanner, M. Tassieri, D. Meek, M. Vassalli, M. J. P. Biggs, M. Salmeron-Sanchez, R. O. C. Oreffo, S. Reid, M. J. Dalby, *Bio. Rxiv.* **2020**, *14*, 10027.
[89] S. H. Hsu, C. T. Chen, Y. H. Wei, *Stem Cells* **2013**, *31*, 2779.
[90] J. H. Kim, S. M. Yoon, S. U. Song, S. Park, W. Kim, I. Park, J. Lee, J. Sung, *Int. J. Mol. Sci.* **2016**, *17*, 1389.
[91] C. Fotia, A. Massa, F. Boriani, N. Baldini, D. Granchi, *J. Cell Biochem.* **2015**, *116*, 1442.
[92] M. Wagegg, T. Gaber, F. L. Lohanathaetal, *PLoS One* **2012**, *7*, 46483.
[93] J. N. Regan, J. Lim, Y. Shi, K. S. Joeng, J. M. Arbeit, R. V. Shohet, F. Long, *Proc. Natl. Acad. Sci. USA* **2014**, *111*, 8673.
[94] H. Mao, A. Yang, Y. Zhao, L. Lei, H. Li, *Stem Cells Inter.* **2020**, *2020*, 2016809.
[95] D. Zou, W. Han, S. You, D. Ye, L. Wang, S. Wang, J. Zhao, W. Zhang, X. Jiang, X. Zhang, Y. Huang, *Cell Prolif.* **2011**, *44*, 234.
[96] L. Zhao, L. Liu, Z. Wu, Y. Zhang, P. K. Chu, *Biomaterials* **2012**, *33*, 2629.
[97] S. H. Shin, J. J. Yoo, H. N. Kim, J. Nam, H. J. Kim, *Connect. Tissue Res.* **2012**, *53*, 318.
[98] W. B. Huang, B. Carlsen, G. Rudkin, M. Berry, K. Ishida, D. T. Yamaguchi, T. A. Mille, *Bone* **2004**, *34*, 799.
[99] A. Forghani, L. Kriegh, K. Hogan, C. Chen, G. Brewer, T. B. Tighe, R. Devireddy, D. Hayes, *J. Biomed. Mater. Res.* **2017**, *105*, 1346.
[100] O. Naňka, P. Valášek, M. Dvořáková, M. Grim, *Dev. Dyn.* **2006**, *235*, 723.
[101] Y. Yang, M. Sun, L. Wang, B. Jiao, *J. Cell. Biochem.* **2013**, *114*, 967.

© 2021 The Authors. Advanced Healthcare Materials published by Wiley-VCH GmbH

21922659, 2021, 22, Downloaded from https://advanced.onlinelibrary.wiley.com/doi/10.1002/adhm.202101215 by National Institutes Of Health Malaysia, Wiley Online Library on [01/02/2026]. See the Terms and Conditions (https://onlinelibrary.wiley.com/terms-and-conditions) on Wiley Online Library for rules of use; OA articles are governed by the applicable Creative Commons License
