# Supplementary material for: Molecular Regulators for the Wnt/β-Catenin Signalling Pathway for Bone Remodelling in Periodontitis: A Scoping Review
Source: Int J Mol Sci. 2026 Oct 14;27(20):9117. doi: 10.3390/ijms27209117 (PMC13705578; doi:10.3390/ijms27209117)
Supplement: Supplementary file 1 [file ijms-27-09117-s001.zip › Supplementary Data/Papers/in vitro/Autophagy negative-regulating Wnt signaling enhanced inflammatory osteoclastogenesis from Pre-OCs in vitro.pdf]

Biomedicine & Pharmacotherapy 126 (2020) 110093

Contents lists available at ScienceDirect

Biomedicine & Pharmacotherapy

journal homepage: www.elsevier.com/locate/biopha

Autophagy negative-regulating Wnt signaling enhanced inflammatory osteoclastogenesis from Pre-OCs *in vitro*

Lili Chen[a,1], Yuting Yang[a,b,1], Jiaqi Bao[a], Zhongxiu Wang[a], Mengjiao Xia[a], Anna Dai[a], Jingyi Tan[a], Lili Zhou[a], Yanmin Wu[a], Weilian Sun[a,*]

[a] *Department of Periodontology, The Second Affiliated Hospital of Zhejiang University, School of Medicine, Hangzhou 310009, PR China*
[b] *Cancer Institute, The Second Affiliated Hospital of Zhejiang University, School of Medicine, Hangzhou 310009, PR China*

ARTICLE INFO

Keywords:
Osteoclast
Lipopolysaccharide
Autophagy
Wnt signaling

ABSTRACT

Periodontitis thereby the alveolar bone loss induced by inflammation, is a wide-spread phenomenon around the world. It is an ongoing challenge faced by clinicians worldwide. This study aimed to identify the effects of lipopolysaccharide (LPS) on osteoclasts (OCs) differentiation *in vitro* and to investigate its molecular mechanism. For bone marrow derived macrophages (considered as Pro-OCs), LPS impaired their differentiation into OCs in a dose-dependent manner. In contrast, it promoted Pre-OCs (referred to receptor activator of nuclear factor-κB ligand (RANKL) pretreated Pro-OCs) and differentiated to OCs with increased maximum diameter, quantity, the covering area and the fusion index *in vitro*. It also facilitated OCs proliferation, bone resorption and OCs related genes expression. Furthermore, it was revealed that LPS enhanced OCs genesis from Pre-OCs *via* activating autophagy pathway consequently elevated the accumulation of TRAP, Cts K and NFATC1, specific genes of OCs. The members of Wnt signaling were expressed as at lower states during the LPS induced OCs formation, but they could be rescued in the presence of autophagy inhibitor. The most promising observation was the direct interaction of LC3B and Dvl2, indicating that the crosstalk between above pathways existed in OCs. Taken together, we consider that LPS activates autophagy which negatively regulates Wnt signaling *via* autophagic degradation of Dvl2 is significant for osteoclastogenesis from Pre-OCs *in vitro.* Our study sheds light on the fact that autophagy inhibitors will become a new, potentially applicable therapeutic option in the treatment of periodontal bone loss.

1. Introduction

Periodontitis is a chronic inflammatory disease involved in the teeth supporting tissue, resulting in alveolar bone destruction and thereby dysfunctional of dentition [1]. This is a matter of concern for clinicians. The dynamic balance between osteolysis and osteogenesis is the key for alveolar bone homeostasis [2]. When engaged in inflammation, it leans to bone resorption due to the overactive osteoclasts (OCs). OCs characterized as multi-nucleated, tartrate-resistant acid phosphatase (TRAP)-positive cells, are derived from hematopoietic cells and differentiate under the regulation of macrophage colony-stimulating factor (M-CSF) [3] and receptor activator of nuclear factor-κB ligand (RANKL) [4]. Interaction between RANKL and its receptor activator of nuclear factor-κB (RANK) activated various downstream cascaded signaling pathways, which are necessary for the formation and function of OCs [5].

Gram-negative anaerobic bacteria in unattached subgingival plaque are generally considered to be the advancing front of periodontitis [1,6]. Lipopolysaccharide (LPS), their main pathogenic structure, has been reported to proliferate OCs *in vivo* [7,8] but had dual effects upon RANKL mediated osteoclastogenesis *in vitro*. However, though some researches demonstrated that LPS enhanced osteoclastogenesis independent of RANKL, more acknowledged was that it exerted promotion only in Pre-OCs [9–11].

Autophagy is used to self-digest the dysfunctional organelles and long-lived proteins in eukaryotic cells, which can be upregulated to provide nutrients under cellular metabolic stress, *e.g.* infection and cancer [12]. During autophagy, cytoplasmic component is sequestered into double-membrane vesicles called autophagosomes and then catabolized after fusion with lysosomes [13]. Deregulation of it has been association with inflammatory diseases, cancer, neurodegenerative diseases, et al. [14]. In periodontitis, autophagy related genes and

* Corresponding author.
E-mail address: weiliansun@zju.edu.cn (W. Sun).
[1] These authors contributed equally.

https://doi.org/10.1016/j.biopha.2020.110093
Received 8 January 2020; Received in revised form 7 March 2020; Accepted 9 March 2020
0753-3322/ © 2020 The Authors. Published by Elsevier Masson SAS. This is an open access article under the CC BY-NC-ND license (http://creativecommons.org/licenses/BY-NC-ND/4.0/).

autophagosomes were exhibited increased basal level [15], indicating that autophagy enhanced by inflammation was associated with periodontal damnification. Newly studies indicated that autophagy also attenuated cell differentiation [16,17] and modulated bone physiology and pathophysiology [18]. Atg5, Atg7 and LC3, the key components of autophagy flux, were found important to generate ruffled border of OCs and bone resorption *in vivo* and *vitro* [19], suggesting that increased autophagy was also critical for OCs function. Typical medicine, such as kaempferol, could predictably impair OCs formation and function *via* downregulating autophagy and thereby help to alleviate bone loss [20]. This implied that impairment of autophagy resulted in aberrant osteoclastogenesis. Since the OCs isolated from rheumatoid arthritis was detected as having a high expression of Atg7 and Beclin1, it was also supported that inflammation might induce autophagy in OCs [21].

However, the way in which autophagy mediated OCs differentiation in the presence of LPS remained to be fully elucidated. Wnt signaling (referred to the β catenin pathway) was another pathway that was important to defend against cellular stress [22] and to facilitate bone genesis [23,24]. It was found to be negatively regulated by autophagy in neurodegenerative diseases and colon cancer [25,26]. Unlike promoting osteoblastogenesis, it effected in OCs formation with multiform. Some research showed that it inhibited OCs differentiation both *in vivo* and *vitro*, although necessary for proliferation of OCs progenitors [27–29]. Thus, Wnt signaling agonist could be used to treat osteolytic lesion by inhibiting OCs formation [30,31]. Some other literature indicated that constitutive activation of β catenin also facilitated osteoclastogenesis [32]. Up to now, few studies reported the effects of Wnt signaling in alveolar bone destruction of periodontitis let alone whether the crosstalk between autophagy and Wnt signaling influenced the OCs differentiation within the stimulation of LPS.

Herein, the effects of LPS on different stages of OCs formation *in vitro* was investigated. We further analyzed the effects of autophagy flux and Wnt signaling and clarified their crosstalk in LPS induced OCs. Our strategy may provide new insight for curing alveolar bone loss caused by periodontitis.

2. Materials and methods

2.1. Cell culture and OCs formation

RAW264.7 cell line was purchased from Cell Bank of the Chinese Academy of Sciences (Shanghai, China), cultured with H-DMEM (Gibico, USA) supplemented with 10 % fetal bovine serum (FBS, Biological industries, Israel) and 1% penicillin-streptomycin (TBD, China).

Bone marrow cells were flushed from femora and tibias of C57BL/6 male mice aged 4–6 weeks with α-MEM (Gibico, USA) containing 10 % FBS and 1% penicillin-streptomycin. The suspension was cultured with 30 ng/ml M-CSF (Novoprotein, China) for three days. The final remained adherent cells were bone marrow derived macrophages (BMMs). RAW264.7 and BMMs were defined as Pro-OCs. Animals were cared and used according the guidelines of the Second Affiliated Hospital of Zhejiang University School of Medicine. The approval number of animal ethics committee is 2017-052 in February 10, 2017.

The induction medium for osteoclast composed of basic medium, 30 ng/ml M-CSF and 50 ng/ml RANKL (R&D Systems, USA). Pre-OCs incubated in induction medium for 48 h could form Pre-OCs.

To evaluate the effect of LPS in Pro-OCs, both BMMs and RAW264.7 cells were treated with 0, 0.78, 1.56, 3.123, 6.25, 12.5, 25, 50, and 100 ng/ml LPS (Sigma, USA) in addition of induction medium for 4 days. To investigate the influence of LPS in Pre-OCs, BMMs and RAW264.7 cells were incubated in induction medium for 48 h, and then added different dose of LPS for two more days. To evaluate the effect of 3-methyladenine (3MA) in LPS induced OCs, various doses of it was added simultaneously with LPS in Pre-OCs.

Pre-OCs cultured with RANKL was considered as control group. Pre-OCs cultured with RANKL and 50 ng/ml LPS was considered as LPS group. Pre-OCs cultured with 2 mM 3MA addition to LPS condition was considered as 3MA group.

All cells were cultured at 37°C with 5% CO_2 in a humidified incubator.

2.2. Tartrate-resistant acid phosphatase(TRAP) staining

Removing medium with micropipettor, and washed cells softly with phosphate-buffered saline (PBS, TBD, China) twice. Then, 100 μl paraformaldehyde (4%) was put into each well, and cells were fixed for 20 min at room temperature. These cells were stained with Leukocyte Acid Phosphatase Kit (Sigma-Aldrich, USA), following the protocol. Purple stained cells with more than three nuclei were considered as osteoclasts. Images were acquired by Leica light microscope (Solms, Germany).

2.3. Bone resorption assay

OCs were seeded on bone slices, and incubated with LPS in the presence or absence of 3MA for 7 days. Medium was renewed every other day. The slices were cleaned with a soft brush and subsequently rinsed with PBS. A scanning electron microscope (JSM-6700 F, Tokyo) was used to visualize the absorption pits.

2.4. Cell viability assay

Cells were seeded in 96-well plates and incubated in different medium till time points. Cell viability was assessed by Cell Counting Kit 8(CCK-8, Boster, China) following the protocol. Briefly, 10 μl CCK-8 solution was added to each well at indicated time and incubated in dark at 37 °C with 5% CO_2 for 1 h. Optical density (OD) values at 450 nm were determined by a spectrophotometer (Bio-Rad, CA, US).

2.5. RNA isolation and quantitative real-time polymerase chain reaction (qPCR)

Cells were harvested by TRIzol (Invitrogen, USA) and the total RNA was isolated according to the protocol. 1 μg RNA of each sample was reverse-transcribed using a PrimeScript Reverse Transcription Master Mix Kit (Takara, Japan). The expression levels of genes were quantified by SYBR Green Master Mix kit (Takara, Japan) on an ABI 7500 Fast real-Time PCR System (Applied Biosystems, Thermo Fisher Scientific, Inc.). The copy numbers were calculated using the $2^{-\Delta\Delta CT}$ method, normalized to the glyceraldehyde-3-phosphate (GAPDH) gene. The primers of target genes were as follows:

5′-CACTCCCACCCTGAGATTTGT-3′ and 5′-CCCCAGAGACATGATGAAGTCA-3′ for TRAP; 5′-CACTCCCACCCTGAGATTTGT-3′ and 5′-TTGCAGCTAGGAAGTACGTCT-3′ for NFATC1; 5′-CTCGGCGTTTAATTTGGGAGA-3′ and 5′-TCGAGAGGGAGGTATTCTGAGT-3′ for Cts K; 5′-ACCAGGATGGTGCATACCTCT-3′ and 5′-CCCATTACAAGCAAACCAGAAGT-3′ for Axin2; 5′-CCGTTCGCCTTCATTATGGA-3′ and 5′-GGCAAGGTTTCGAATCAATCC-3′ for β catenin; 5′-AAGGGTGCTGTGTACTGGAC-3′ and 5′-AGAAGAGAACCTTACGGGACG-3′ for LRP5; 5′-TGTGCTTCGAGATGTGTGGTT-3′ and 5′-GTCAAATAGCTGACTCTTGGCAA-3′ for Atg5; 5′-CCAGTCCGTTGAAGTCCTCT-3′ and 5′-AGATGACTCAGCCAGCCTTT-3′ for Atg7; 5′-TCCTGGGCATTGTGATCCAT-3′ and 5′-AAGTGAGGGTTGGGAGATGG-3′ for Atg12; 5′-GGTTGTCTCCTGCGACTTCA-3′ and 5′-TGGTCCAGGGTTTCTTACTCC-3′ for GAPDH.

2.6. Western blot analysis

Cells were washed with cold PBS twice and extracted by lysate buffer (Thermo Fisher Scientific, USA). After quantified by BCA kit (Thermo Fisher Scientific, USA), 25 μg proteins were subjected to SDS-PAGE and transferred onto polyvinylidene fluoride membranes (Bio-

Rad, USA). They were blocked with 5% skim milk for 1 h at room temperature and incubated with antibody against NFATC1 (#8032, Cell Signaling), Cts K (#187647, Abcam), Beclin1 (#207612, Abcam), Atg5 (#ET1611-38, HUABIO), Atg7 (#85583, Cell Signaling), LC3B (#192890, Abcam), P62 (#EM0704, HUABIO), Dvl2 (#3224 T, Cell Signaling), β catenin (#32572, Abcam) and β actin (#8226, Abcam) overnight at 4 °C. Membranes then washed three times with TBST and incubated at room temperature for 1 h with mouse or rabbit horseradish peroxidase conjugated secondary antibody (#HA1006 and #HA1001, HUABIO). Blots were visualized by enhanced chemiluminescence kit (Thermo Fisher Scientific, USA) and further analyzed by using ImageJ software (National Institutes of Health, MD, USA).

2.7. Immunoprecipitation

Cells were lysed in cold RIPA buffer Yeasen Biotech, China with the addition of protease inhibitor cocktail Bimake, USA for 2 h at 4℃, and then centrifuged for 20 min at 13,000 rpm to collect supernatant. 20 μl protein A/G PLUS-agarose beads Santa Cruz Biotechnology, USA were incubated with 0.5 μg anti-LC3B antibody or IgG for 20 min at room temperature followed by immunoprecipitate with cell lysate for 12 h at 4℃. Samples were centrifuged and washed with cold RIPA for five times, then boiled with 1x loading buffer FD Bio Science, China and analyzed by immune blot.

2.8. Statistical analysis

Each experiment was performed three times independently. Values were expressed as the mean ± standard deviation (SD). Groups were analyzed by one-way ANOVA with Prism software (GraphPad, CA, USA). P value < 0.05 was considered statistically significant.

3. Result

3.1. LPS promotes Pre-OCs but inhibits Pro-OCs differentiating into mature OCs in vitro

BMMs (Pro-OCs) were isolated, stimulated with RANKL and LPS simultaneously for 4 days and then stained with Leukocyte Acid Phosphatase Kit. Number of purple stained OCs rapidly decreased in a concentration-dependent manner. The concentration was up than 25 ng/ml, few OCs could be observed (Fig. 1A). Similar results were observed in RAW264.7 (Fig. 1B). However, LPS was conducive to drive the RANKL primed BMMs into OCs. The maximum diameter, the quantity, the covering area and the fusion index of OCs induced by LPS were significantly increased compared to the control group. LPS at 50 ng/ml showed statistically significant promotion (Fig. 1C-D). Similar results were also confirmed by RAW264.7 cells line (Fig. A.1B). The dual effects of LPS indicated that LPS could only promote RANKL induced OCs formation in Pre-OCs but not Pro-OCs.

To identify whether LPS affected the proliferation of Pre-OCs, we examined cell viability by CCK-8. Both 25 ng/ml and 50 ng/ml LPS promoted Pre-OCs proliferation significantly after 48 h stimulation compared to the none treated group (Fig. 2D), suggesting that LPS protected OCs from apoptosis. Real-time PCR and western blot were used to further investigate the molecular effect of LPS on Pre-OCs differentiation. The results were shown that the transcription and translation of NFATC1, TRAP and Cts K, which were vital for OCs genesis were significantly upregulated by two LPS groups. And LPS at 50 ng/ml was shown as a stronger accelerator for osteoclastogenesis (Fig. 2A–C, E–G). Next, we implanted the induced OCs on bone slices to observe their osteolytic ability. OCs induced by LPS produced more absorption pits than those formed in response to RANKL only (Fig. 2H-I). It was briefly indicated that LPS could proliferate Pre-OCs and promote them to express OCs related genes, thus encouraging them to differentiate to bioactive OCs *in vitro*.

3.2. LPS promotes OCs formation via upregulating autophagy

Autophagy has been reported crucial for anti-infection [33,34] and necessarily for RANKL induced osteoclastogenesis [19,35]. Thus, we conjectured that LPS promoted Pre-OCs differentiation by activating autophagy. The autophagy related proteins were measured by western blot. As shown in Fig. 3A–D, Beclin1, Atg5, Atg7 and LC3B accumulated more in LPS conditions (25 ng/ml and 50 ng/ml) at different time points, and higher concentration of LPS accompanied with higher expression level. P62 has been regarded as an autophagy adaptor, signing the autophagy flux [36]. It was upregulated by LPS also in a concentration dependent manner (Fig. 3A-D). Next, we analyzed the mRNA level of Atg5, Atg7 and Atg12, key genes to form autophagosome, 50 ng/ml LPS led a higher transcription level than ctrl group (Fig. 3E). Since LPS at 50 ng/ml presented significant promotion without distinct toxicity, we chose this concentration for further research.

3-MA was an inhibitor of PI3K that aimed to block autophagy signaling at the early stage [37], therefore reduced autophagy related proteins (Fig. 3A, D). It (0.5 mM, 1 mM, 1.5 mM and 2 mM) consistently impaired osteoclastogenensis in LPS (referred to 50 ng/ml) stimulated Pre-OCs. When the dose was up to 2 mM, there were few multinuclear $TRAP^+$ cells (Fig. 3G). 3-MA lower than 4 nM had no detrimental effect on the cell viability (Fig. 3F). Then, we analyzed the influence of inhibition of autophagy on OCs related genes. As shown in Fig. 4A-C, 3MA reversed the mRNA and protein level of NFATC1, Cts K and TRAP promoted by LPS. In addition, the number of bone absorption pits elevated by LPS was also significantly dropped (Fig. 2J).

3.3. Autophagy downregulates Wnt signaling in LPS induced OCs formation

The β catenin was the key point for Wnt pathway, which would be significantly decreased during Pre-OCs differentiation. At the late stage of osteoclastogenesis (cultured after 24 h), hardly blots of it could be analyzed. When stimulated by LPS, degradation progress could be proceeded (Fig. 4G-H). Further measurements in mRNA showed that LPS downregulated the transcription level of β catenin too (Fig. 4E). Dvl2 and LRP5 were membrane proteins that determined to start Wnt signaling [24]. Their expression was also decreased during OCs formation, and further downregulated by LPS (Fig. 4D, G). Additionally, LPS significantly reduced the mRNA level of Axin2, downstream of Wnt pathway (Fig. 4F). It was suggested that Wnt/β catenin pathway was downregulated in osteoclastogenesis and LPS proceeded the degradation of it to promote OCs formation.

Considering the autophagy as a "cleaner" in cells, we hypothesized that Wnt related proteins were metabolized by it in LPS induced OCs formation. As expected, blocking the autophagy with 3MA rescued the protein level of Dvl2 and β catenin (Fig. 4G, I) as well as the mRNA of LRP5, β catenin and Axin2 significantly (Fig. 4D-F). These results suggested that autophagy negatively regulated Wnt signaling. Then we tried to found the relationship between these two pathways. Gao et al. reported a negative correlation between Dvl2 and Beclin1 in the late stages of tumors and observed co-localization of Dvl2 and LC3B in Hela cells [26]. Therefore, we hypothesized that the autophagic degradation of Dvl2 was an intersection of autophagy and Wnt signaling in OCs. Indeed, it was observed that the direct interaction between LC3B and Dvl2 in LPS induced OCs (Fig. 4J).

4. Discussion

We demonstrated that LPS promoted osteoclastogenesis conditionally *in vitro*. LPS inhibited Pro-OCs differentiation in a concentration-dependent manner. 25 ng/ml LPS almost completely blocked OCs generation from BMMs. However, numbers of mature OCs rapidly increased together with more expressed OCs related genes when it was applied to Pre-OCs, suggesting that LPS accelerated the OCs differentiation and promoted cells proliferation in Pre-OCs. So why LPS

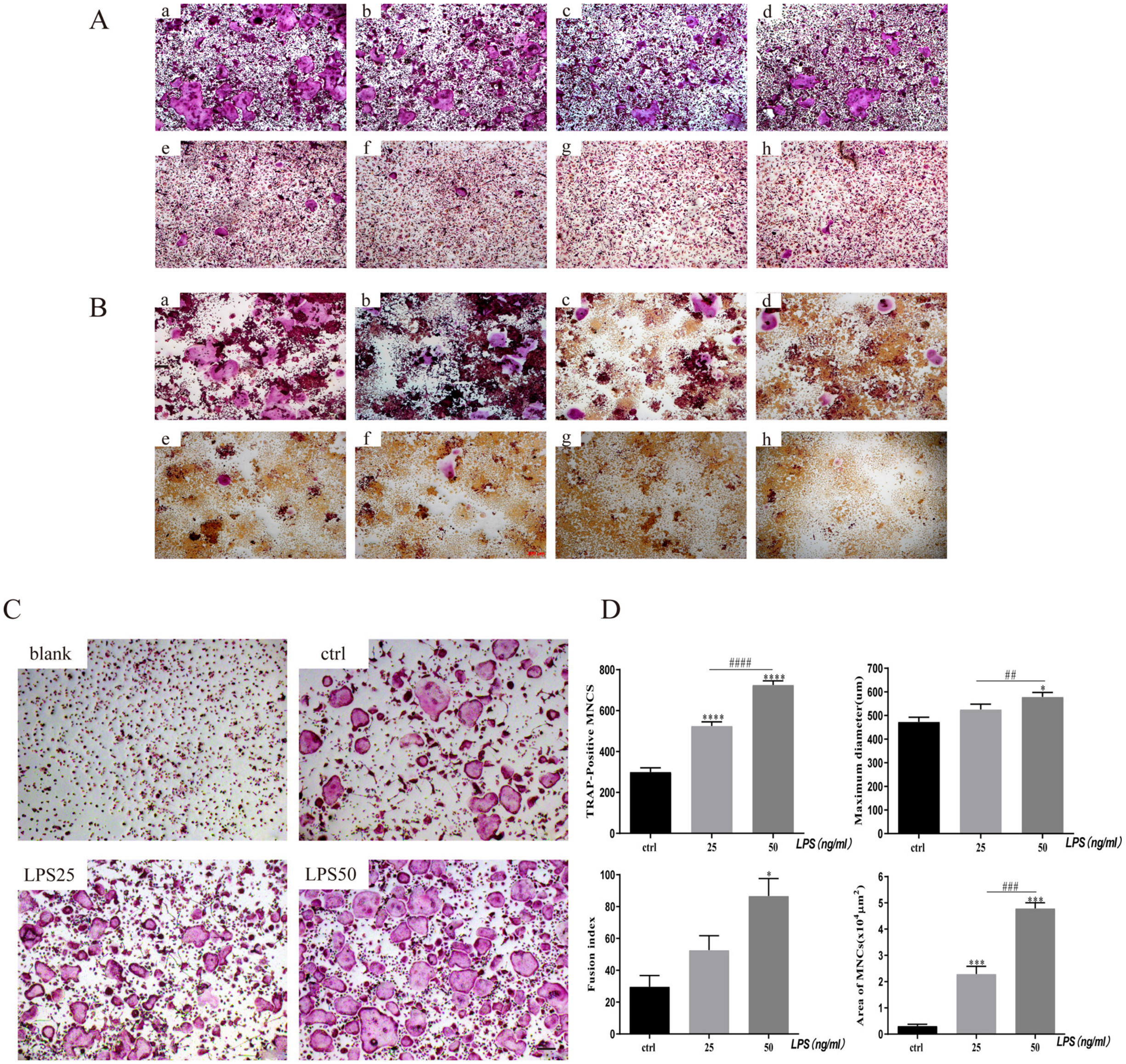

Fig. 1. LPS exerted positive effects on osteoclastogenesis in Pre-OCs but impacted Pro-OCs differentiation *in vitro*. The representative TRAP$^+$ multinucleated cells of different concentration of LPS treatment concomitantly with RANKL in BMMs (A) and RAW 264.7 cell line (B) were presented. Both fig a referred to RANKL stimulation only, while fig b to h referred to LPS at 0.78, 1.56, 3.13, 6.25, 12.50, 25.00, and 50.00 ng/ml, respectively. The number of TRAP positive OCs induced by LPS in Pre-OCs were counted (D) and representative photos of C. TRAP$^+$ cells were measured with maximum diameter and area of mature OCs. Fusion index was defined as the average number of nuclei per OCs, indicating the quantity of cells that contributed to fusion (D). Scale bar: 200 μm. $*P < 0.05$; $**P < 0.01$; $***P < 0.001$; $****P < 0.0001$ compared to the control group. $\#\#P < 0.01$; $\#\#\#P < 0.001$; $\#\#\#\#P < 0.0001$ compared to 25 ng/ml LPS group. Each experiment was performed three times independently.

caused discrepant effect in stages of OCs *in vitro*? OCs generated from hematopoietic stem cells *via* monocyte-macrophage lineage. As with immune cells, Pro-OCs maintained phagocytic function and expressed 9 types Toll-like receptors (TLRs). LPS was the ligand to TLR4. Other TLRs ligands like PNG, ploy(I:C) RNA and CpG DNA exhibited similar inhibition in Pro-OC [10], suggesting that activation of TLRs cascades could impact the osteoclastogenic signaling induced by RANKL. For mature OCs, LPS extended their survival [38]. As well, stimulation of TLR2 *via* PNG also prolonged the lifetime of OCs [10], suggesting that activation of TLRs pathways protected mature OCs from apoptosis. Thus, we conjectured that the dominant position of RANKL-RANK cascades determined the differentiation to OCs and reversed the negative effect of TLRs signaling. Our previous animal experiments observed that bacterial infection caused alveolar bone loss consistent with more OCs genesis [39]. Since LPS could only enhanced Pre-OCs differentiation *in vitro*, how can we explain these conflicts? It has been reported that alveolar bone destruction due to the enhanced osteoclastogenesis in periodontitis was mediated by T cells immunity [40]. Significant accumulation in the proportion of memory T cells characterized as CD4 and CD69 double positive was observed in periodontitis tissues compared to the healthy gingiva [41]. However, for immune deficient mice, infection didn't cause significant alveolar bone loss, suggesting that LPS didn't have a direct effect on osteoclastogenesis [42]. Regulatory T cells was essential to control violent immune response thus minimize collateral organic damage. It has been proposed as new therapy strategy to periodontitis [43]. Enhanced T cells response to bacterial infection could express more RANKL into periodontal tissue which was required for facilitating OCs formation. Therefore, it seemed like that alveolar bone loss caused by periodontitis may be the side effect of exacerbated T cells immune responses. Moreover, co-cultured mouse spleen cells with osteoblasts successfully incubated mature OCs [44]. Transdifferentiation of in-mature dendritic cells into osteoclasts were observed

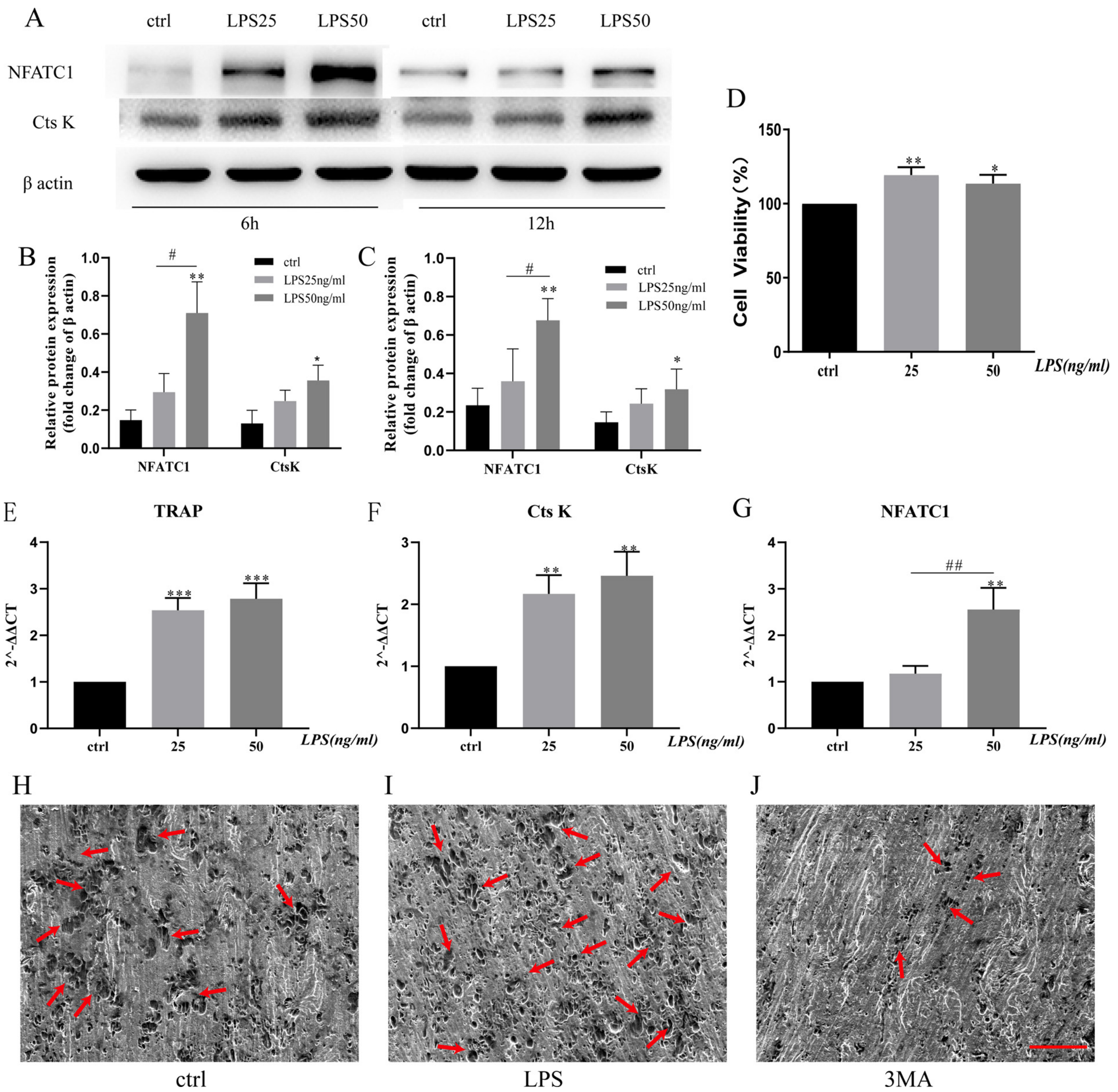

Fig. 2. LPS promoted OCs related genes expression and enhanced osteolytic activity in Pre-OCs. Both 25 ng/ml and 50 ng/ml LPS increased the accumulation of NFATC1 and Cts K at 6 h and 12 h detected by western blot (A) and bolts of three parallel experiments were quantitated as B (6 h) and C (12 h). Pre-OCs stimulated with LPS for 9 h were analyzed using primer of NFATC1 (G), TRAP (E) and Cts K (F) by qPCR. The mRNA fold of control group was set at 1. Incubation with LPS after 48 h for Pre-OCs, cell viability was measured. OD value of control group was set as 100 % (D). Bone slices were used to exam the osteolytic ability of OCs induced by different medium. Representative photos of resorption pits (red arrow referred) of three groups as shown (H-J). Scale bar: 200 μm. $^{*}P < 0.05$; $^{**}P < 0.01$; $^{***}P < 0.001$ compared to the control group. $^{\#\#}P < 0.01$ compared to 25 ng/ml LPS group. Each experiment was performed three times independently.

under inflammation condition [45,46]. THP1 was human monocyte lineage which was elucidated differentiation into osteoclasts under RANKL and MCSF with addition of PMA [47]. The transdifferentiation potentiality of various immune cells suggesting that bacteria induced periodontal inflammation may contribute to the transdifferentiate of them into osteoclasts thereby resulted in increased alveolar bone destruction. Therefore, the active bone absorption in periodontitis due to inflammation seemed like an indirect outcome of enhanced immune responses.

Here we provided evidences that LPS advanced osteoclastogenesis *via* promoting autophagic flux. Beclin1, Atg5, Atg7 and LC3B, important markers of autophagy, were elevated during OCs formation. Blockage of autophagic flux with 3MA decreased autophagic proteins as well as the number of OCs and bone absorption pits induced by LPS. High expression of TRAP, Cts K and NFATC1 responsive to LPS stimulation was reversed by 3MA. These results indicated that LPS activated autophagy which was critical for the differentiation and activity of OCs, suggesting that inhibitor of autophagy may be a potential therapy to periodontal bone loss. Other studies also found a role of autophagy in OCs. Myeloid deletion of Atg5 or Atg7 impaired OCs activity and resulted in dysfunctional ruffled borders and fewer resorption pits, but had no effect on OCs differentiation [19]. Deficiency of LC3B impaired formation of actin ring in OCs thus reduced bone absorption [48]. These findings implied that autophagy played a critical role in forming ruffled border, thus affected OCs activity.

Recent research demonstrated that autophagy could be selective. The tight interaction between selected cargo and phagopore distinguished it from nonselective. Selected cargo needed to be labeled first, mainly ubiquitinated, then co-aggregated and recruited to receptors. Finally Atg8s (LC3B was one type of Atg8) acted as adaptors attaching cargo-receptor complex to the inner membrane of phagophore [49,50]. Due to its diverse domains, P62 was identified as main

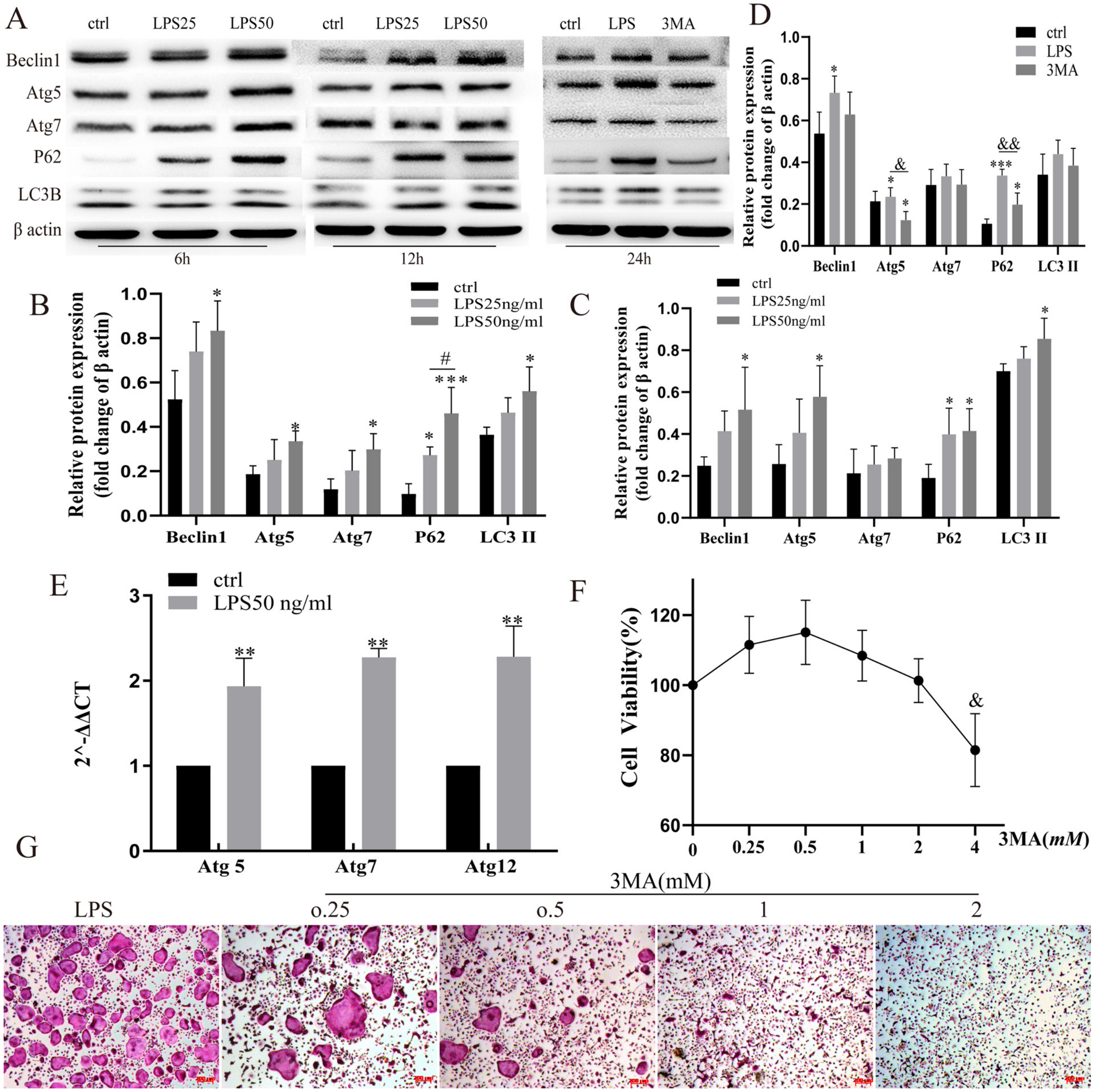

Fig. 3. LPS enhanced autophagy was critical for osteogenesis. Pre-OCs were incubated with 25 ng/ml, 50 ng/ml LPS with or without 3MA for 6 h, 12 h, and 24 h. Cell lysis were harvested and detected with antibody of Beclin1, Atg5, Atg7, P62 and LC3B (A). Bolts of three parallel experiments were quantitated as B (6 h), C (12 h) and D (24 h). Pre-OCs treated with LPS for 9 h and was analyzed using primer of Atg5, Atg7 and Atg12 *via* qPCR. The expression level of control group was set at 1 (E). Pre-OCs incubated with 50 ng/ml LPS and different doses of 3MA for 48 h, then were analyzed of their cell viability with CCK-8. Absorbance of 50 ng/ml LPS group was set as 100 % (F). Representative photos of OCs formed under 3MA were shown (G). Scale bar: 200 μm. $^{*}P < 0.05$; $^{**}P < 0.01$; $^{***}P < 0.001$ compared to the control group. $\#P < 0.05$ compared to 25 ng/ml LPS group. &P < 0.05; &&P < 0.01 compared to 50 ng/ml LPS group. Each experiment was performed three times independently.

autophagic receptor [36,51]. During osteoclastogenesis, we observed that the protein level of P62 was significantly enhanced but rapidly decreased by 3MA, suggesting that P62 played an important role in autophagic flux thereby affected OCs genesis. Deficient of P62 predominantly suppressed OCs formation *in vitro* [52]. Its mutation was found association with Paget's disease [53]. Thus, we considered that selective autophagy led by P62 rather the nonselective play a more important role in OCs formation.

Activation of Wnt signaling was vital to osteogenesis [23,24]. In our study, LPS continuously depressed β catenin negatively correlated with the elevated differentiation to OCs, suggesting that it may play as an inhibiting feedback in OCs formation. This view was supported by the activation of Wnt signaling, which caused by LiCl or Wnt 3a in the multiple myeloma cells or bone marrow significantly suppressed osteoclastogenesis [54]. Then, we tried to figure out which down-regulated the Wnt signaling in OCs. The participation of autophagy in negative regulation to Wnt signaling has been demonstrated in late stage of colon cancer [26] and protective to neuro ischemia [55]. To our expectation, blocking autophagy with 3MA rescued Dvl2, β catenin and other downstream genes in Pre-OCs stimulated with LPS. Moreover, we found direct interaction between LC3B and Dvl2. It seemed like that LC3B selectively recruited Dvl2 to autophagosomes, which fused with lysosome and completed degradation later. P62 as a scaffold receptor, was found interacted with Dvl2 and composed a trimer with LC3B [26,56]. P62 may also be functioned as a link between Dvl2 and LC3B in OCs.

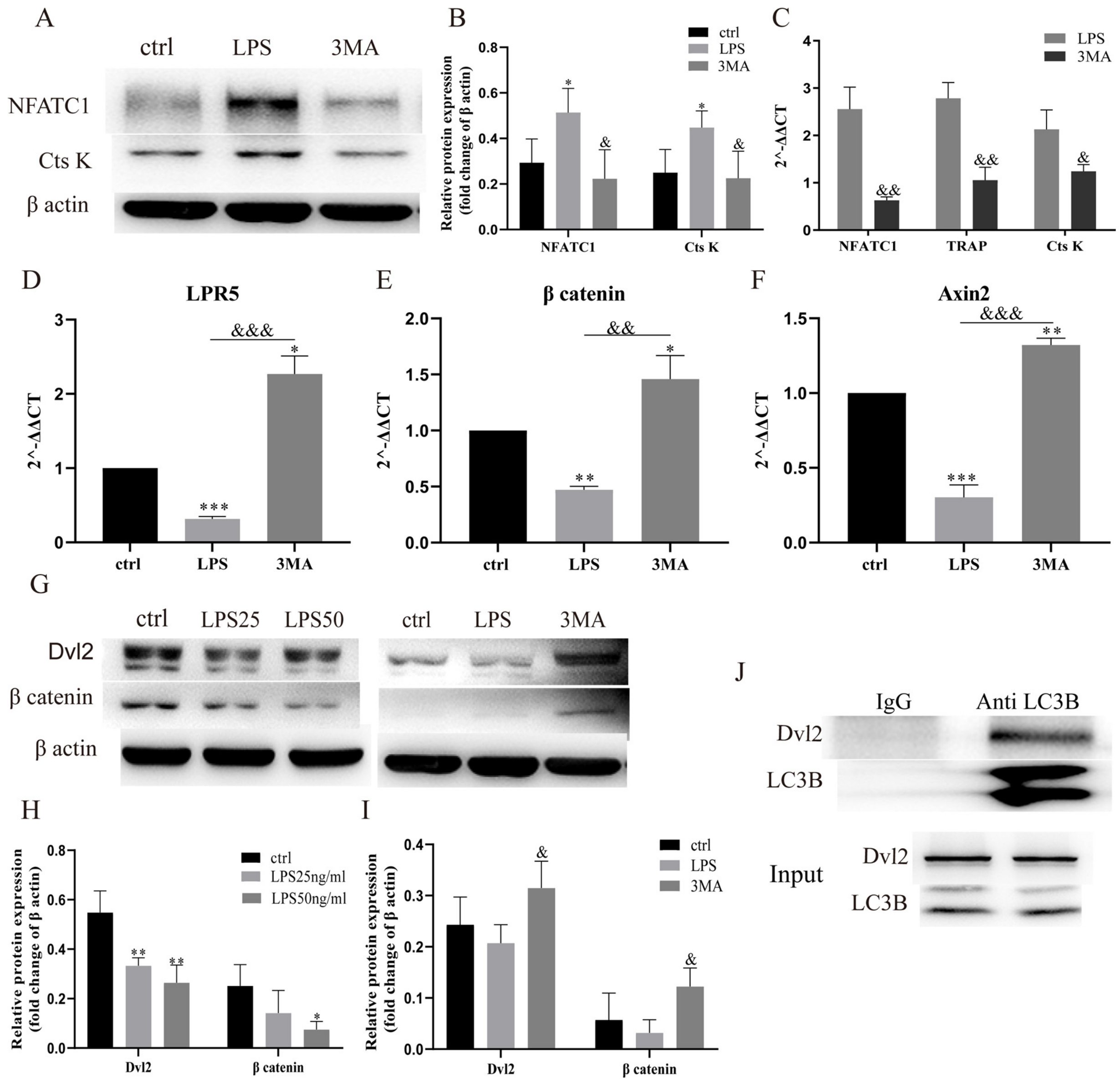

Fig. 4. Autophagy promoted osteoclastogenesis *via* degrading Wnt signaling. Pre-OCs were incubated with 50 ng/ml LPS in the presence or absence of 2 mM 3MA for 24 h. Cell lysis were collected and analyzed with NFATC1, Cts K and TRAP by western blots (A). Bolts of three parallel experiments were quantitated as B. The mRNA level of them was exhibited as C. Protein level of Dvl2 and β catenin in different concentration of LPS with or without 3MA was analyzed by western blot (G) and quantitated with other two parallel results (H for 12 h and I for 24 h). Pre-OCs cultured in 50 ng/ml LPS with or without 2 mM 3MA for 9 h were analyzed with LRP5, β catenin and Axin2, genes of Wnt signaling by qPCR (D-F). The mRNA fold of control group was set at 1. Immunoprecipitation of Dvl2 to LC3B was operated in LPS induced OCs (J). Ig G from rabbit was set as the negative control. *$P < 0.05$; **$P < 0.01$; ***$P < 0.001$ compared to the control group. &$P < 0.05$; &&$P < 0.01$; &&& $P < 0.001$ compared to 50 ng/ml LPS group. Each experiment was performed three times independently.

5. Conclusion

Our studies revealed that LPS enhanced osteoclastogenesis only from Pre-OCs *in vitro*. Activated autophagy pathway and its receptor, P62 recruited Dvl2 to LC3B. Dvl2 was degraded *via* autophygosome therefore inhibiting the downstream of Wnt signaling. In this way, OCs differentiation was encouraged. Our findings shed light on a new regulating effect of autophagy in OCs genesis, and suggest that selective autophagy might be a potential targeted therapy to reduce inflammatory alveolar bone loss in periodontitis.

Fundings

This work was supported by A Project Supported by Scientific Research Fund of Zhejiang Provincial Education Department [grant number Y201942090]; Natural Science Foundation of Zhejiang Province [grant number LY16H140002, LQ19H140003]; National Natural Science Foundation of China [grant number 81771072, 81800972] and the Science and Technology Department of Zhejiang Province [grant number LGF18H140003].

Declaration of Competing Interest

All authors declare that there are no conflicts of interest associated with this study.

Appendix A. Supplementary data

Supplementary material related to this article can be found, in the online version, at doi:https://doi.org/10.1016/j.biopha.2020.110093.

References

[1] D.F. Kinane, P.G. Stathopoulou, P.N. Papapanou, Periodontal diseases, Nat. Rev. Dis. Primers 3 (2017) 17038.
[2] M.N. Weitzmann, Bone and the immune system, Toxicol. Pathol. 45 (2017) 911–924.
[3] F.P. Ross, S.L. Teitelbaum, Alphavbeta3 and macrophage colony-stimulating factor: partners in osteoclast biology, Immunol. Rev. 208 (2005) 88–105.
[4] J.E. Horton, L.G. Raisz, H.A. Simmons, J.J. Oppenheim, S.E. Mergenhagen, Bone resorbing activity in supernatant fluid from cultured human peripheral blood leukocytes, Science (New York, N.Y.) 177 (1972) 793–795.
[5] S. Publications, Advances in the regulation of osteoclasts and osteoclast functions, Dent. Res. 92 (10) (2013) 860–867.
[6] J.D. Harvey, Periodontal microbiology, Dent. Clin. North Am. 61 (2017) 253–269.
[7] P. Orcel, M. Feuga, J. Bielakoff, M.C. De Vernejoul, Local bone injections of LPS and M-CSF increase bone resorption by different pathways in vivo in rats, Am. J. Physiol. 264 (1993) E391–E397.
[8] C. Wei, Q. Liu, F. Song, X. Lin, Y. Su, J. Xu, L. Huang, S. Zong, J. Zhao, Artesunate inhibits RANKL-induced osteoclastogenesis and bone resorption in vitro and prevents LPS-induced bone loss in vivo, J. Cell. Physiol. 233 (2018) 476–485.
[9] O. Sul, H. Park, H. Son, H. Choi, Lipopolysaccharide (LPS)-induced autophagy is responsible for enhanced osteoclastogenesis, Mol. Cells 11 (2017) 880–887.
[10] M. Takami, N. Kim, J. Rho, Y. Choi, Stimulation by toll-like receptors inhibits osteoclast differentiation, J. Immunol. (Baltim. Md.: 1950) 169 (2002) 1516–1523.
[11] W. Zou, Z. Bar Shavit, Dual modulation of osteoclast differentiation by lipopolysaccharide, J. Bone Miner. Res. 17 (2002) 1211–1218.
[12] P. Ravanan, I.F. Srikumar, P. Talwar, Autophagy: the spotlight for cellular stress responses, Life Sci. 188 (2017) 53–67.
[13] N. Mizushima, Autophagy: process and function, Gene Dev. 21 (2007) 2861–2873.
[14] B. Levine, G. Kroemer, Autophagy in the pathogenesis of disease, Cell 132 (2008) 27–42.
[15] Y. An, W. Liu, P. Xue, Y. Zhang, Q. Wang, Y. Jin, Increased autophagy is required to protect periodontal ligament stem cells from apoptosis in inflammatory microenvironment, J. Clin. Periodontol. 43 (2016) 618–625.
[16] T. Riffelmacher, F.C. Richter, A.K. Simon, Autophagy dictates metabolism and differentiation of inflammatory immune cells, Autophagy 14 (2018) 199–206.
[17] D.J. Puleston, H. Zhang, T.J. Powell, E. Lipina, S. Sims, I. Panse, A.S. Watson, V. Cerundolo, A.R. Townsend, P. Klenerman, A.K. Simon, Autophagy is a critical regulator of memory CD8(+) T cell formation, ELIFE 3 (2014) e3706.
[18] V. Pierrefite-Carle, S. Santucci-Darmanin, V. Breuil, O. Camuzard, G.F. Carle, Autophagy in bone: self-eating to stay in balance, Ageing Res. Rev. 24 (2015) 206–217.
[19] C.J. DeSelm, B.C. Miller, W. Zou, W.L. Beatty, E. van Meel, Y. Takahata, J. Klumperman, S.A. Tooze, S.L. Teitelbaum, H.W. Virgin, Autophagy proteins regulate the secretory component of osteoclastic bone resorption, Dev. Cell 21 (2011) 966–974.
[20] C.J. Kim, S.H. Shin, B.J. Kim, C.H. Kim, J.H. Kim, H.M. Kang, B.S. Park, I.R. Kim, The effects of kaempferol-inhibited autophagy on osteoclast formation, Int. J. Mol. Sci. 19 (2018) 125.
[21] N. Lin, C. Beyer, A. Giessl, T. Kireva, C. Scholtysek, S. Uderhardt, L.E. Munoz, C. Dees, A. Distler, S. Wirtz, G. Kronke, B. Spencer, O. Distler, G. Schett, J.H.W. Distler, Autophagy regulates TNFalpha-mediated joint destruction in experimentalarthritis, Ann. Rheum. Dis. 72 (2013) 761–768.
[22] F. Rijsewijk, M. Schuermann, E. Wagenaar, P. Parren, D. Weigel, R. Nusse, The Drosophila homolog of the mouse mammary oncogene int-1 is identical to the segment polarity gene wingless, Cell 50 (1987) 649–657.
[23] U.H. Lerner, C. Ohlsson, The WNT system: background and its role in bone, J. Intern. Med. 277 (2015) 630–649.
[24] M.A. Rudnicki, B.O. Williams, Wnt signaling in bone and muscle, Bone 80 (2015) 60–66.
[25] K.J. Petherick, A.C. Williams, J.D. Lane, P. Ordonez-Moran, J. Huelsken, T.J. Collard, H.J. Smartt, J. Batson, K. Malik, C. Paraskeva, A. Greenhough, Autolysosomal beta-catenin degradation regulates Wnt-autophagy-p62 crosstalk, EMBO J. 32 (2013) 1903–1916.
[26] C. Gao, W. Cao, L. Bao, W. Zuo, G. Xie, Autophagy negatively regulates Wnt signalling by promoting dishevelled degradation, Nat. Cell Biol. (2010).
[27] M.M. Weivoda, M. Ruan, C.M. Hachfeld, L. Pederson, A. Howe, R.A. Davey, J.D. Zajac, Y. Kobayashi, B.O. Williams, J.J. Westendorf, S. Khosla, M.J. Oursler, Wnt signaling inhibits osteoclast differentiation by activating canonical and non-canonical cAMP/PKA pathways, J. Bone Miner. Res. 31 (2016) 65–75.
[28] Wnt Signaling Inhibits Osteoclast Differentiation by Activating Canonical and Noncanonical cAMP/PKA Pathways.
[29] W. Wei, D. Zeve, J.M. Suh, X. Wang, Y. Du, J.E. Zerwekh, P.C. Dechow, J.M. Graff, Y. Wan, Biphasic and dosage-dependent regulation of osteoclastogenesis by beta-catenin, Mol. Cell. Biol. 31 (2011) 4706–4719.
[30] Z. Ping, X. Hu, L. Wang, J. Shi, Y. Tao, X. Wu, Z. Hou, X. Guo, W. Zhang, H. Yang, Y. Xu, Z. Wang, D. Geng, Melatonin attenuates titanium particle-induced osteolysis via activation of Wnt/beta-catenin signaling pathway, Acta Biomater. 51 (2017) 513–525.
[31] L. Du, M. Nong, J. Zhao, X. Peng, S. Zong, G. Zeng, Polygonatum sibiricum polysaccharide inhibits osteoporosis by promoting osteoblast formation and blocking osteoclastogenesis through Wnt/beta-catenin signalling pathway, Sci. REP-UK 6 (2016) 32261.
[32] X. Sui, S. Deng, M. Liu, L. Fan, Y. Wang, H. Xu, Y. Sun, A. Kishen, Q. Zhang, Constitutive activation of beta-catenin in differentiated osteoclasts induces bone loss in mice, Cell. Physiol. Biochem. 48 (2018) 2091–2102.
[33] Y. Romeo, B. Lemaitre, Autophagy in immunity and inflammation.
[34] P. Lapaquette, J. Guzzo, L. Bretillon, M. Bringer, Cellular and molecular connections between autophagy and inflammation, Mediat. Inflamm. 2015 (2015) 398483.
[35] R. Li, G. Chen, J. Ren, W. Zhang, Z. Wu, B. Liu, Y. Zhao, Y. Zhao, The adaptor protein p62 is involved in RANKL-induced autophagy and osteoclastogenesis, J. Histochem. Cytochem. 62 (2014) 879–888.
[36] T. Lamark, S. Svenning, T. Johansen, Regulation of selective autophagy: the p62/SQSTM1 paradigm, Essays Biochem. 61 (2017) 609–624.
[37] Y. Wu, H. Tan, G. Shui, C. Bauvy, Q. Huang, M.R. Wenk, C. Ong, P. Codogno, H. Shen, Dual role of 3-Methyladenine in modulation of autophagy via different temporal patterns of inhibition on class I and III phosphoinositide 3-Kinase, J. Biol. Chem. 285 (2010) 10850–10861.
[38] K. Suda, J. Woo, M. Takami, P.M. Sexton, K. Nagai, Lipopolysaccharide supports survival and fusion of preosteoclasts independent of TNF-alpha, IL-1, and RANKL, J. Cell. Physiol. 190 (2002) 101–108.
[39] L. Song, J. Tan, Z. Wang, P. Ding, Q. Tang, M. Xia, Y. Wei, L. Chen, Interleukin-17A facilitates osteoclast differentiation and bone resorption via activation of autophagy in mouse bone marrow macrophages, Mol. Med. Rep. 19 (2019) 4743–4752.
[40] Y.T. Teng, H. Nguyen, X. Gao, Y.Y. Kong, R.M. Gorczynski, B. Singh, R.P. Ellen, J.M. Penninger, Functional human T-cell immunity and osteoprotegerin ligand control alveolar bonedestruction in periodontal infection, J. Clin. Invest. 106 (2000) R59–R67.
[41] R. Mahanonda, C. Champaiboon, K. Subbalekha, N. Sa-Ard-Iam, A. Yongyuth, B. Isaraphithakkul, P. Rerkyen, O. Charatkulangkun, S. Pichyangkul, Memory T cell subsets in healthy gingiva and periodontitis tissues, J. Periodontol. 89 (2018) 1121–1130.
[42] Y.T. Teng, H. Nguyen, X. Gao, Y.Y. Kong, R.M. Gorczynski, B. Singh, R.P. Ellen, J.M. Penninger, Functional human T-cell immunity and osteoprotegerin ligand control alveolar bonedestruction in periodontal infection, J. Clin. Invest. 106 (2000) R59–R67.
[43] C. Alvarez, C. Rojas, L. Rojas, E.A. Cafferata, G. Monasterio, R. Vernal, Regulatory T Lymphocytes in periodontitis: a translational view, Mediat. Inflamm. 2018 (2018) 7806912.
[44] N. Udagawa, N. Takahashi, T. Akatsu, T. Sasaki, A. Yamaguchi, H. Kodama, T.J. Martin, T. Suda, The bone marrow-derived stromal cell lines MC3T3-G2/PA6 and ST2 supportosteoclast-like cell differentiation in cocultures with mouse spleen cells, Endocrinology 125 (1989) 1805–1813.
[45] A. Krishnamurthy, A.J. Ytterberg, M. Sun, K. Sakuraba, J. Steen, V. Joshua, N.K. Tarasova, V. Malmstrom, H. Wahamaa, B. Rethi, A.I. Catrina, Citrullination controls dendritic cell transdifferentiation into osteoclasts, J. Immunol. (Baltim., Md. : 1950) 202 (2019) 3143–3150.
[46] O. Laperine, C. Blin-Wakkach, J. Guicheux, S. Beck-Cormier, P. Lesclous, Dendritic-cell-derived osteoclasts: a new game changer inbone-resorption-associated diseases, Drug Discov. Today 21 (2016) 1345–1354.
[47] Z.H. Li, Y. Si, G. Xu, X.M. Chen, H. Xiong, L. Lai, Y.Q. Zheng, Z.G. Zhang, High-dose PMA with RANKL and MCSF induces THP1 cell differentiation into humanfunctional osteoclasts in vitro, Mol. Med. Rep. 16 (2017) 8380–8384.
[48] Y. Chung, S. Yoon, B. Choi, D.H. Sohn, K. Yoon, W. Kim, D. Kim, E. Chang, Microtubule-associated protein light chain 3 regulates Cdc42-dependent actin ringformation in osteoclast, Int. J. Biochem. Cell Biol. 44 (2012) 989–997.
[49] S. Svenning, T. Johansen, Selective autophagy, Essays Biochem. 55 (2013) 79–92.
[50] V. Rogov, V. Dotsch, T. Johansen, V. Kirkin, Interactions between autophagy receptors and ubiquitin-like proteins form themolecular basis for selective autophagy, Mol. Cell 53 (2014) 167–178.
[51] S. Pankiv, T.H. Clausen, T. Lamark, A. Brech, J. Bruun, H. Outzen, A. Overvatn, G. Bjorkoy, T. Johansen, p62/SQSTM1 binds directly to Atg8/LC3 to facilitate degradation of ubiquitinated protein aggregates by autophagy, J. Biol. Chem. 282 (2007) 24131–24145.
[52] F. Zach, F. Polzer, A. Mueller, A. Gessner, p62/sequestosome 1 deficiency accelerates osteoclastogenesis in vitro and leads to Paget's disease–like bone phenotypes in mice, JBC 293 (2018) 9530–9541.
[53] M. Seton, M. Hansen, D.H. Solomon, The implications of the sequestosome 1 mutation P392L in patients with Paget's disease in a United States cohort, Calcified Tissue Int. 98 (2016) 489–496.
[54] Y. Qiang, Y. Chen, N. Brown, B. Hu, J. Epstein, B. Barlogie, J.D.J. Shaughnessy, Characterization of Wnt/beta-catenin signalling in osteoclasts in multiplemyeloma, Brit. J. Haematol. 148 (2010) 726–738.
[55] Z. Shi, J. Deng, S. Fu, L. Wang, Q. Wang, B. Liu, Y. Li, J. Deng, Protective effect of autophagy in neural ischemia and hypoxia: negativeregulation of the Wnt/beta-catenin pathway, Int. J. Mol. Med. 40 (2017) 1699–1708.
[56] B. Ma, B. Liu, W. Cao, C. Gao, Z. Qi, Y. Ning, Y. Chen, The Wnt signaling antagonist Dapper1 accelerates Dishevelled2 degradation via promoting its ubiquitination and aggregate-induced autophagy, J. Biol. Chem. 290 (2015) 12346–12354.
