# Supplementary material for: Molecular Regulators for the Wnt/β-Catenin Signalling Pathway for Bone Remodelling in Periodontitis: A Scoping Review
Source: Int J Mol Sci. 2026 Oct 14;27(20):9117. doi: 10.3390/ijms27209117 (PMC13705578; doi:10.3390/ijms27209117)
Supplement: Supplementary file 1 [file ijms-27-09117-s001.zip › Supplementary Data/Papers/in vitro/Biological Mechanisms of Paeonoside in the Differentiation of Pre-Osteoblasts and the Formation of Mineralized Nodules.pdf]

International Journal of *Molecular Sciences*

MDPI

*Article*

# Biological Mechanisms of Paeonoside in the Differentiation of Pre-Osteoblasts and the Formation of Mineralized Nodules

**Kyung-Ran Park [1], Joon Yeop Lee [2], Myounglae Cho [2], Jin Tae Hong [3,*] and Hyung-Mun Yun [1,*]**

[1] Department of Oral and Maxillofacial Pathology, School of Dentistry, Kyung Hee University, Seoul 02447, Korea; rudfks282@naver.com
[2] National Institute for Korean Medicine Development, Gyeongsan 38540, Korea; chool9090@nikom.or.kr (J.Y.L.); meanglae@nikom.or.kr (M.C.)
[3] College of Pharmacy and Medical Research Center, Chungbuk National University, Chungbuk 28160, Korea
* Correspondence: jinthong@chungbuk.ac.kr (J.T.H.); yunhm@khu.ac.kr (H.-M.Y.); Tel.: +82-02-961-0691 (J.T.H.); Fax: +82-02-960-1457 (J.T.H.)

**Abstract:** *Paeonia suffruticosa* is a magnificent and long-lived woody plant that has traditionally been used to treat various diseases including inflammatory, neurological, cancer, and cardiovascular diseases. In the present study, we demonstrated the biological mechanisms of paeonoside (PASI) isolated from the dried roots of *P. suffruticosa* in pre-osteoblasts. Herein, we found that PASI has no cytotoxic effects on pre-osteoblasts. Migration assay showed that PASI promoted wound healing and transmigration in osteoblast differentiation. PASI increased early osteoblast differentiation and mineralized nodule formation. In addition, PASI enhanced the expression of Wnt3a and bone morphogenetic protein 2 (BMP2) and activated their downstream molecules, Smad1/5/8 and β-catenin, leading to increases in runt-related transcription factor 2 (RUNX2) expression during osteoblast differentiation. Furthermore, PASI-mediated osteoblast differentiation was attenuated by inhibiting the BMP2 and Wnt3a pathways, which was accompanied by reduction in the expression of RUNX2 in the nucleus. Taken together, our findings provide evidence that PASI enhances osteoblast differentiation and mineralized nodules by regulating RUNX2 expression through the BMP2 and Wnt3a pathways, suggesting a potential role for PASI targeting osteoblasts to treat bone diseases including osteoporosis and periodontitis.

**Keywords:** bone mineralization; BMP2; osteoblast differentiation; paeonoside; RUNX2; Wnt3a

check for updates

**Citation:** Park, K.-R.; Lee, J.Y.; Cho, M.; Hong, J.T.; Yun, H.-M. Biological Mechanisms of Paeonoside in the Differentiation of Pre-Osteoblasts and the Formation of Mineralized Nodules. *Int. J. Mol. Sci.* **2021**, *22*, 6899. https://doi.org/10.3390/ijms22136899

Academic Editor: Chih-Hsin Tang

Received: 10 June 2021
Accepted: 23 June 2021
Published: 27 June 2021

**Publisher's Note:** MDPI stays neutral with regard to jurisdictional claims in published maps and institutional affiliations.

**Copyright:** © 2021 by the authors. Licensee MDPI, Basel, Switzerland. This article is an open access article distributed under the terms and conditions of the Creative Commons Attribution (CC BY) license (https://creativecommons.org/licenses/by/4.0/).

## 1. Introduction

Bone tissue is dynamically and continuously renewed through various cellular events in bone cells including osteoblasts, osteocytes, and osteoclasts to maintain a healthy skeleton [1,2]. Specifically, osteoblasts play an important role in bone development, formation, and regeneration via the synthesis and release of bone proteins and the mineralization of organic bone matrix [2,3]. Malfunction of the physiological and dynamic process pathologically induces bone diseases including osteoporosis and periodontal disease [4].

Bone morphogenetic proteins (BMPs) and Wnts stimulate various intracellular signaling and play crucial roles in multiple stages such as mesoderm patterning, osteoblast differentiation, bone formation, and craniofacial and limb development [5–7]. BMPs are multi-functional growth factors belonging to the transforming growth factor-beta superfamily. Among them, BMP2 is the first BMP to have been well-studied in the BMP2-Smad1/5/8 pathway and approved by the Food and Drug Administration for its use in bone diseases [8]. Wnt3a/β-catenin signaling is a well-studied pathway to regulate the proteasomal degradation of β-catenin [9]. The BMP2 and Wnt3a signaling pathways induce osteoblast differentiation by regulating runt-related transcription factor 2 (RUNX2) that is an important transcription factor [8,10,11].

*Paeonia suffruticosa* has been used as a medicinal plant for thousands of year and has beneficial effects in the treatment of various diseases including cancer, cardiovascular,

inflammatory, and neurological diseases [12]. Paeonoside (PASI) is a bioactive compound identified in *P. suffruticosa* [13,14]. PASI possesses anti-diabetic activities by glycogen synthesis and glucose uptake through AMPK activation in HepG2 cells and contributes to glucose homeostasis [15]. It was also reported as a protective compound against sepsis-induced lethality [16]. Our group recently reported that another bioactive compound, paeonolide, identified from *P. suffruticosa* regulates core-binding factor subunit alpha 1 in bone-forming cells [17]. However, the biological effects and mechanisms of PASI have not yet been demonstrated in osteoblasts.

In the present study, we isolated PASI from the roots of *P. suffruticosa* and investigated the biological activities and underlying mechanism of PASI in osteoblast differentiation and mineralized nodule formation in pre-osteoblast MC3T3-E1 cells.

## 2. Results

### 2.1. PASI Does Not Affect Cell Toxicity in Pre-Osteoblasts

The $^{1}$H and $^{13}$C nuclear magnetic resonance (NMR) spectra, high-performance liquid chromatography (HPLC) chromatogram, and chemical structure of 99.9% purity PASI from the dried roots of *P. suffruticosa* are shown in Figure 1A–C. At concentrations ranging from 0.1 to 100 μM, PASI was applied to pre-osteoblast MC3T3-E1 cells for 24 h to determine cytotoxic potential. No cytotoxic effects of PASI were measured by using an 3-[4,5-dimethylthiazol-2-yl]-2,5-diphenyltetrazolium bromide (MTT) assay (Figure 1D). The doses of PASI below 100 μM were chosen in all subsequent experiments.

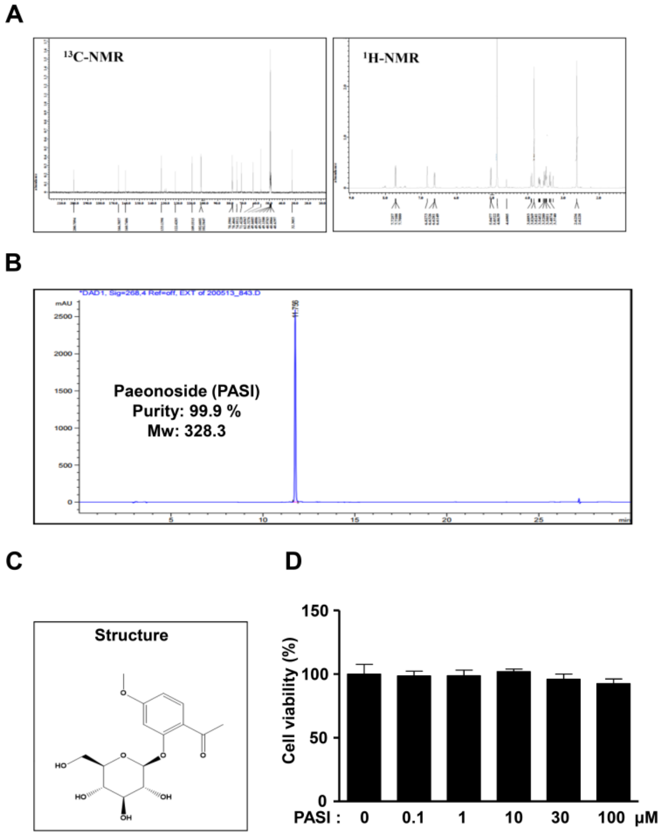


**Figure 1.** Effects of PASI on cytotoxicity against pre-osteoblasts. (**A**–**C**) PASI (99.9 % purity) obtained from the dried roots of *Paeonia suffruticosa* was analyzed by $^{1}$H and $^{13}$C NMR spectra (**A**), HPLC chromatogram (**B**), and chemical structure (**C**). (**D**) Cells were treated with 0.1–100 μM PASI for 24 h. Cell viability (%) was measured by using an MTT assay. Data are expressed as the mean ± S.E.M. of experiments.

## 2.2. *PASI Enhances Cell Migration during Differentiation of Pre-Osteoblasts*

To investigate whether PASI affects cell migration, we induced the differentiation of pre-osteoblasts using an osteogenic supplement (OS) medium in the presence or absence of PASI for 24 h, after which cell migration was observed using wound healing assay. The results showed that 1–30 µM PASI significantly recovered the wound area, compared to OS medium alone in a dose-dependent manner (Figure 2A,B). Next, we used a Boyden chamber assay to validate the effect of PASI. The assay showed that 1–30 µM PASI significantly promoted transmigration in a dose-dependent manner (Figure 2C,D).

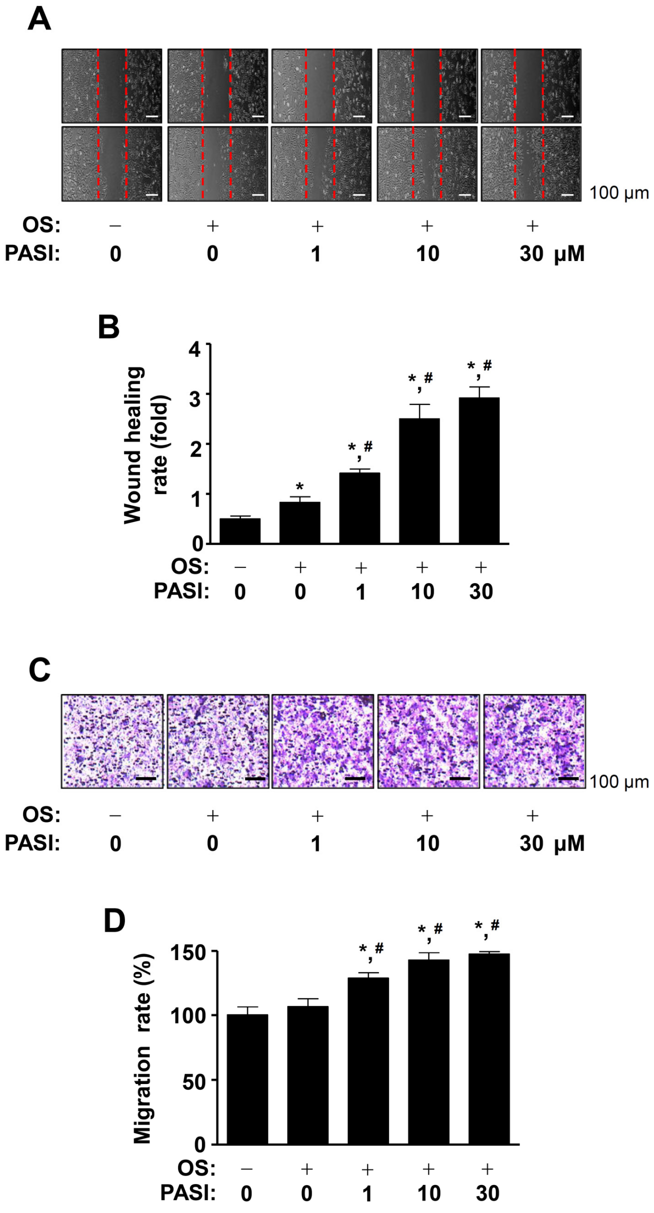


**Figure 2.** Effects of PASI on migration during osteoblast differentiation. (**A**,**B**) The wound healing rate (fold) was detected under a light microscope (**A**) and exhibited as a bar graph (**B**). (**C**,**D**) The Boyden chamber assay was carried out. The migration rate (%) was detected under a light microscope (**C**) and exhibited as a bar graph (**D**). Data are expressed as the mean ± S.E.M. of experiments. * $p < 0.05$, # $p < 0.05$ indicate a statistically significant difference compared to the control and OS, respectively.

## 2.3. *PASI Promotes ALP Staining and Activity during Differentiation of Pre-Osteoblasts*

Next, we stimulated osteoblast differentiation in OS with 1–30 µM PASI for seven days to examine the biological activities of PASI in early osteoblast differentiation. Osteoblast

differentiation was measured by using alkaline phosphatase (ALP) staining as an early osteoblast differentiation marker. The results showed that 1–30 μM PASI promoted ALP staining in a dose-dependent manner (Figure 3A, upper). Under the same conditions, we subsequently performed an ALP enzymatic activity assay, and the activities were detected using a spectrophotometer. The results showed that 1–30 μM PASI significantly promoted the ALP activity in a dose-dependent manner (Figure 3A, bottom). The increased ALP-positive cells in response to 1–30 μM PASI treatment were detected using a light microscope (Figure 3B).

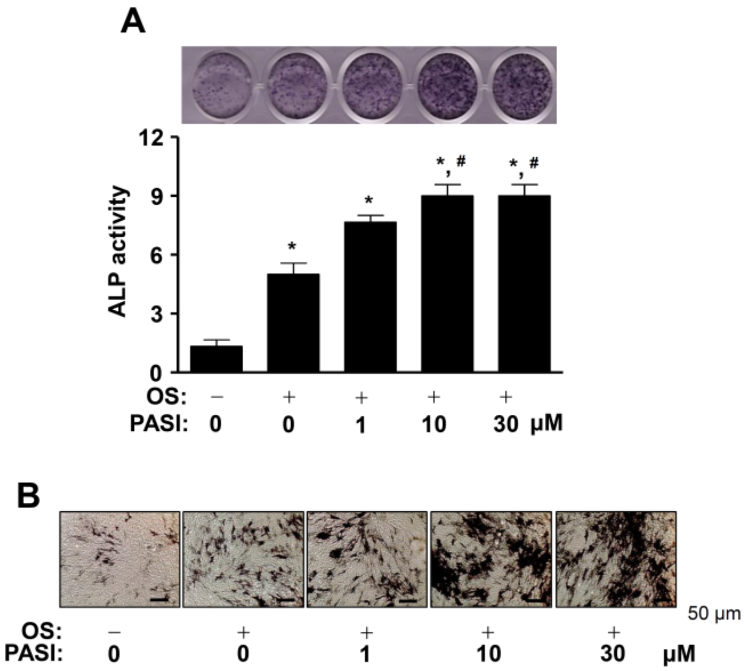


**Figure 3.** Effects of PASI on the staining and activity of ALP during early osteoblast differentiation. (**A**) The staining of ALP was visualized using a scanner (*upper*). The activity of ALP was analyzed by using a spectrophotometer and exhibited as a bar graph (*bottom*). (**B**) ALP-positive cells were detected under light microscopy. Data are expressed as the mean ± S.E.M. of experiments. * $p < 0.05$, # $p < 0.05$ indicate a statistically significant difference compared to the control and OS, respectively.

### *2.4. PASI Promotes ARS Staining during Differentiation of Pre-Osteoblasts*

PASI-mediated osteoblast differentiation was further assessed using Alizarin red S (ARS) staining to investigate the biological activities of PASI in late osteoblast differentiation. Osteoblast differentiation was induced in OS with 1–30 μM PASI for 14 days, after which the formation of mineralized nodules was detected using a scanner. The results showed that 1–30 μM PASI potentiated mineralized nodule formation in a dose-dependent manner (Figure 4A, upper). PASI-mediated late osteoblast differentiation was confirmed by the quantification of ARS stains (Figure 4A, bottom). The mineralized nodules were observed and validated under a light microscope (Figure 4B).

### *2.5. PASI Activates BMP2 and Wnt3a/β-Catenin Signaling during Differentiation of Pre-Osteoblasts*

To understand the intracellular signaling involved in PASI-mediated osteoblast differentiation, BMP2 and Wnt3a/β-catenin signaling were analyzed. First, 1–10 μM PASI elevated the expression of BMP2 and Wnt3a, compared to that with OS (Figure 5A). Second, 1–10 μM PASI stimulated Smad1/5/8 phosphorylation, GSK3β phosphorylation, and β-catenin expression, compared to that with OS (Figure 5B). Finally, 1–10 μM PASI upregulated RUNX2 expression, which is a main convergence gene of the BMP2 and Wnt3a/β-catenin signaling pathways, leading to osteoblast differentiation (Figure 5B).

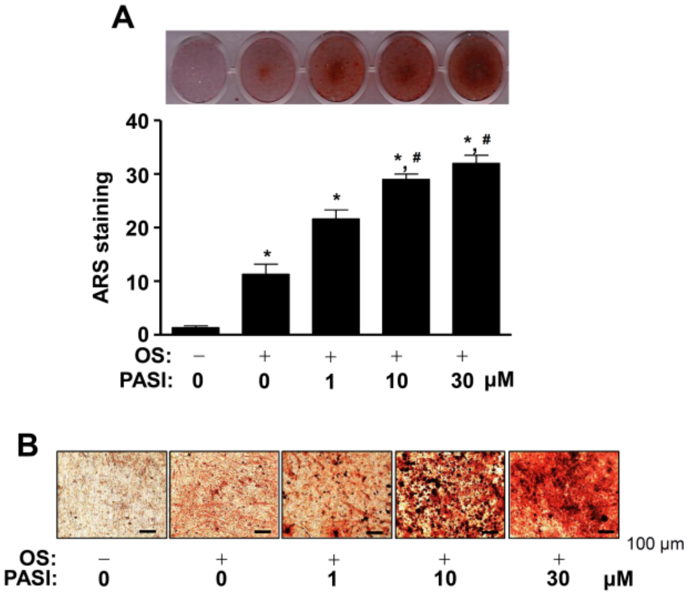


**Figure 4.** Effects of PASI on the staining of ARS during late osteoblast differentiation. (**A**) The staining of ARS was visualized using a scanner (upper). The quantification of ARS stains was analyzed by using a spectrophotometer and exhibited as a bar graph (bottom). (**B**) The formation of the nodules was detected under a light microscope. Data are expressed as the mean ± S.E.M. of experiments. * $p < 0.05$, # $p < 0.05$ indicate a statistically significant difference compared to the control and OS, respectively.

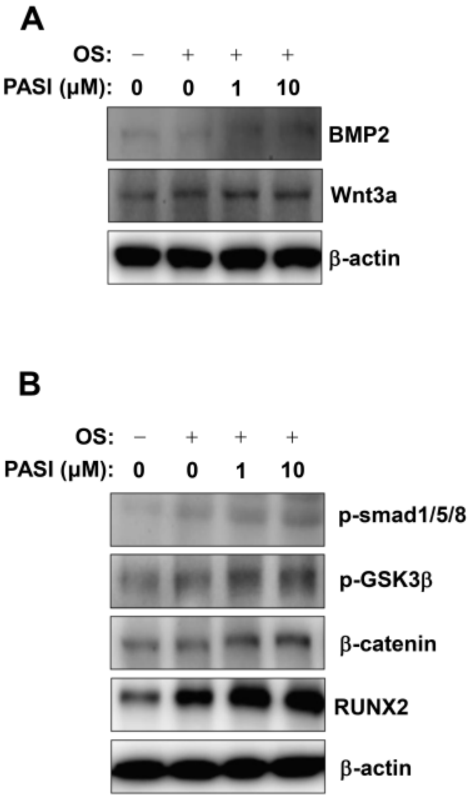


**Figure 5.** Effects of PASI on BMP2 and Wnt3a/β-catenin signaling during osteoblast differentiation. (**A**,**B**) The expression of BMP2 and Wnt3a proteins (**A**), and the phosphorylation of Smad1/5/8 (p-Smad1/5/8) and GSK3β (p-GSK3β) proteins, and the expression of β-catenin protein (**B**) were analyzed using western blot analysis. β-actin was used as an internal control to normalize the level of total lysates.

### *2.6. PASI-Mediated BMP2 and Wnt3a/β-Catenin Signaling Enhances RUNX2 Expression during Differentiation of Pre-Osteoblasts*

To clarify the effect on osteoblast differentiation of PASI-mediated BMP2 and Wnt3a/β-catenin signaling, 10 µM PASI was treated with Noggin and PKF118-310 (PKF), which are inhibitors against BMP2 and Wnt3a/β-catenin signaling, respectively. The pretreatment of Noggin and PKF significantly attenuated the increased RUNX2 expression in response to 10 µM PASI during osteoblast differentiation (Figure 6A,B). Immunofluorescence observation showed that Noggin and PKF blocked the RUNX2 expression increased by 10 µM PASI in the nucleus during osteoblast differentiation (Figure 6C).

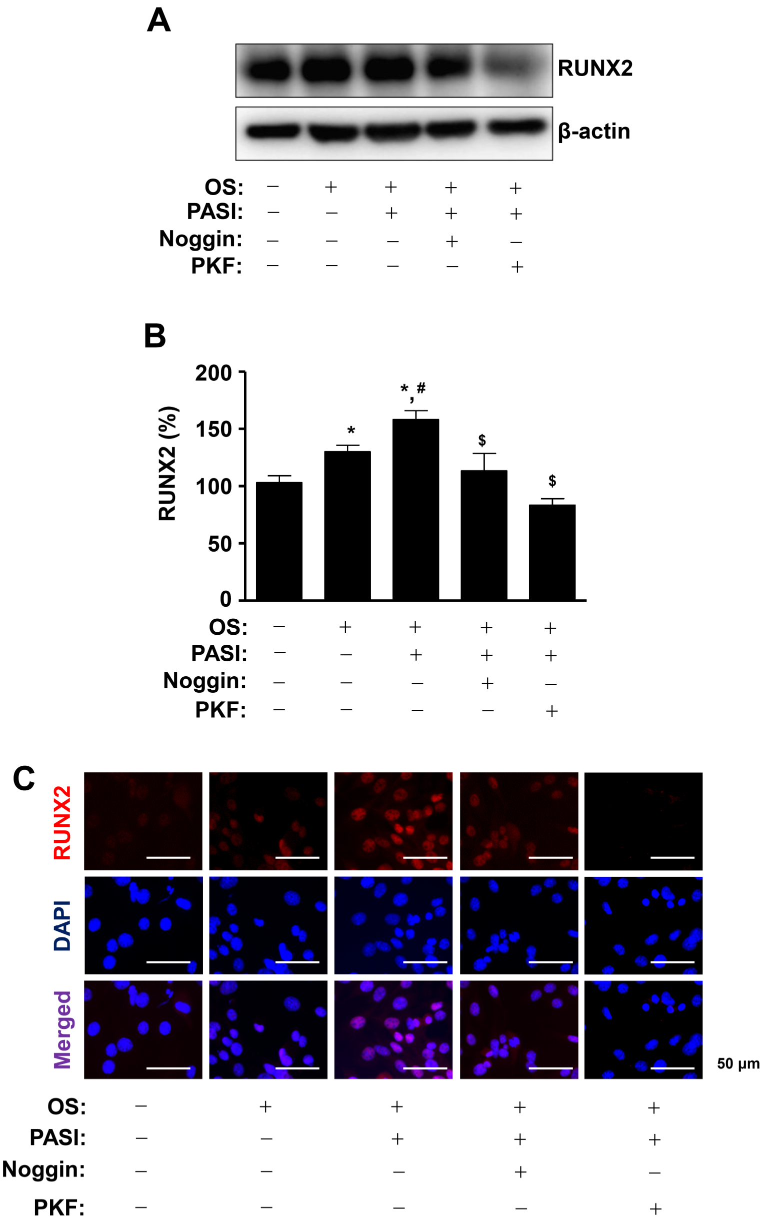


**Figure 6.** PASI promotes the expression of nuclear RUNX2 via BMP2 and Wnt3a/β-catenin signaling during osteoblast differentiation. (**A**,**B**) The expression of RUNX2 protein was analyzed using western blot analysis. β-actin was used as an internal control to normalize the level of total lysates (**A**). The expression levels of RUNX2 (%) are presented as a bar graph (**B**). (**C**) The nuclear expression of RUNX2 was examined using immunefluorescence analysis. The nucleus was stained with a nuclear DAPI marker. RUNX2-and DAPI-positively-stained cells were merged (purple, bottom). Data are expressed as the mean ± S.E.M. of experiments. * $p < 0.05$, # $p < 0.05$, and \$ $p < 0.05$ indicate a statistically significant difference compared to the control, OS, and OS + PASI, respectively.

### 2.7. PASI-Stimulated BMP2 and Wnt3a/β-Catenin Signaling Promotes Osteoblast Differentiation

We further investigated whether PASI potentiated early and late osteoblast differentiation by stimulating BMP2 and Wnt3a/β-catenin signaling. The pretreatment of Noggin and PKF significantly attenuated PASI-mediated ALP staining and activity during early osteoblast differentiation (Figure 7A,B). PASI stimulated the mineralized nodule formation during late osteoblast differentiation, whereas the pretreatment of Noggin and PKF significantly abolished the PASI-mediated effects (Figure 7C,D).

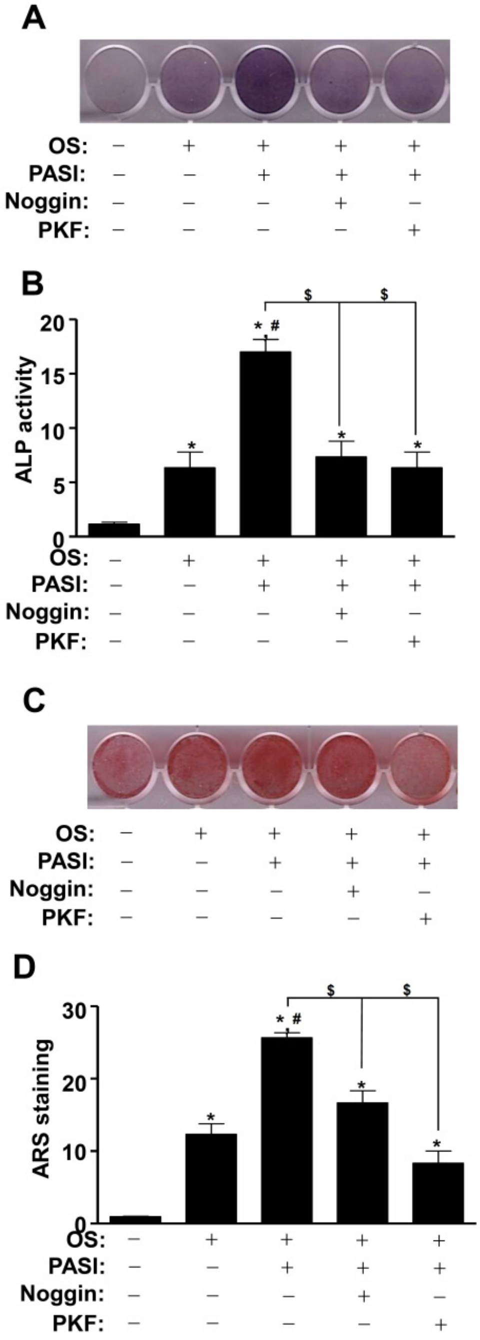


**Figure 7.** Blockage of BMP2 and Wnt3a/β-catenin signaling attenuated PASI-mediated osteoblast differentiation. (**A**,**B**) The staining of ALP was visualized using a scanner (**A**), and the activity of ALP was analyzed using a spectrophotometer and exhibited as a bar graph (**B**). (**C**,**D**) The staining of ARS was visualized using a scanner (**C**), and the quantification was presented as a bar graph (**D**) (bottom). Data are expressed as the mean ± S.E.M. of experiments. * $p < 0.05$, # $p < 0.05$, and $ $p < 0.05$ indicate a statistically significant difference compared to the control, OS, and OS + PASI, respectively.

## 3. Discussion

Osteoblasts are tightly regulated throughout life for bone formation, bone regeneration, and repair processes after bone damage including migration, differentiation, and mineralization [6,18,19]. The dysregulation leads to bone diseases including periodontitis and osteoporosis with age [4,20–24]. Therefore, research targeting osteoblasts is necessary for the development of anabolic drugs [18,25,26]. Recently, our group demonstrated the biological activities and mechanisms of natural compounds in osteoblasts [27–31]. In the present study, we demonstrated the novel function of PASI isolated from *P. suffruticosa* in pre-osteoblasts.

The migration into specific niches from the bone marrow, circulating blood, and periosteum is required for bone regeneration and repair [32–34]. Organic bone matrix and mineralization are achieved during differentiation of pre-osteoblasts [6,35,36]. In the present study, our data demonstrated that PASI promotes migration, ALP activity and its expression, and mineralized nodules. It was reported that ALP expression is increased in immature osteoblasts, and calcium deposition and mineralization are induced by mature osteoblasts [37–39]. Therefore, these data suggest that PASI promotes bone regeneration and repair through the migration, differentiation, and maturation of osteoblasts.

BMP2 and Wnt3a regulate osteoblast differentiation and bone formation [40–42]. BMP2 activates Smad1/5/8 proteins by binding to BMP receptors, induces the translocation of Smad1/5/8 proteins from the cytosol to the nucleus, and then Smad1/5/8 and Smad4 complexes regulate gene transcription in the nucleus [43]. Wnt3a interacts with Frizzled and LRP5/6 receptors, inactivates the GSK3β protein, stabilizes cytoplasmic β-catenin, and induces its nuclear translocation, leading to the regulation of gene expression in the nucleus [44]. Consequently, the signaling pathways promote the transcription of the RUNX2 gene for osteoblast differentiation and maturation [45–48]. In the present study, PASI enhanced BMP2 expression and Smad1/5/8 phosphorylation, as well as promoted Wnt3a expression and β-catenin stabilization, leading to RUNX2 expression. In addition, we demonstrated the nuclear expression of RUNX2 through PASI-stimulated BMP2 and Wnt3a signaling using Noggin and PKF. Noggin interacts with and antagonizes the action of BMP2 [49]. PKF disrupts β-catenin-TCF and inhibits β-catenin-regulated transcription activity [50,51]. It was reported that the BMP2 and Wnt3a/β-catenin signaling pathways are convergent to RUNX2 expression during osteoblast differentiation [52–54]. Previous studies also showed that the genetic modification of RUNX2 caused impaired bone formation and gene expression [55]. These results suggest that PASI-mediated RUNX2 upregulation leads to osteoblast differentiation and bone formation through the functional cross-talk between BMP2 and Wnt3a/β-catenin signaling.

Diet habits affect bone health and are important for reducing the risk of osteoporosis [56]. A dietary intake of calcium, vitamin D, and proteins also helps improve bone strength through bone mineralization such as hydroxyapatite crystals [57]. A soy-based diet that contain isoflavones such as soy milk and Tofu is thought to contain estrogen-like compounds that may help inhibit bone loss and prevent osteoporosis because estrogen hormones help protect bone loss against postmenopausal osteoporosis [58]. It was reported that the combination of soy isoflavones and milk basic protein preserves bone mineral density, and the combination of resveratrol which is found in red wine, red grape skins, and peanuts also prevents bone loss [59,60]. In the present study, we demonstrated that PASI promotes osteoblast differentiation and mineralization. Thus, the combination of PASI and adequate dietary intake could be a strategic approach for postmenopausal osteoporosis.

Osteoblasts secrete the receptor activator of nuclear factor kappa B ligand (RANKL), which is a key osteoclastogenic cytokine. In addition, osteoblasts mainly synthesize oteoprotegerin (OPG) as a circulating decoy receptor of RANKL [61]. The soluble RANKL/OPG system controls the differentiation and function of osteoclasts, thereby clinically determining bone status [62]. Growing evidence suggests that epigenetic modification plays a critical role in the formation of normal bone and the pathogenesis of bone diseases [63]. Epigenetic modification affects gene expression without alterations in genomic DNA [64].

Thus, two concerns of the RANKL/OPG system and epigenetic modification need to be explored to provide additional information on PASI-mediated osteoblast differentiation in future studies.

Currently, many pretreatment methods such as chemical, physical–chemical, and biological methods are being explored [65,66]. It was reported that hydrogen peroxide presoaking prior to ammonia fiber expansion pretreatment had a high advantage in agriculture [66]. Thus, any potential approaches to pretreat and extract PASI from the dried roots of *P. suffruticosa* including presoaking, liquid ammonia pretreatment, and others should be also considered.

In conclusion, we provide novel evidence that PASI from *P. suffruticosa* has biological effects as a regulator of RUNX2 by facilitating BMP2 and Wnt3a/β-catenin signaling (Figure 8). Traditional natural compounds have received interest in bone diseases since the compounds have been used in various diseases for centuries with safety and efficiency [18,25,26,67,68]. Thus, our findings suggest that PASI as an anabolic compound may be developed and used to prevent and alleviate bone diseases by increasing osteoblast differentiation and bone formation.

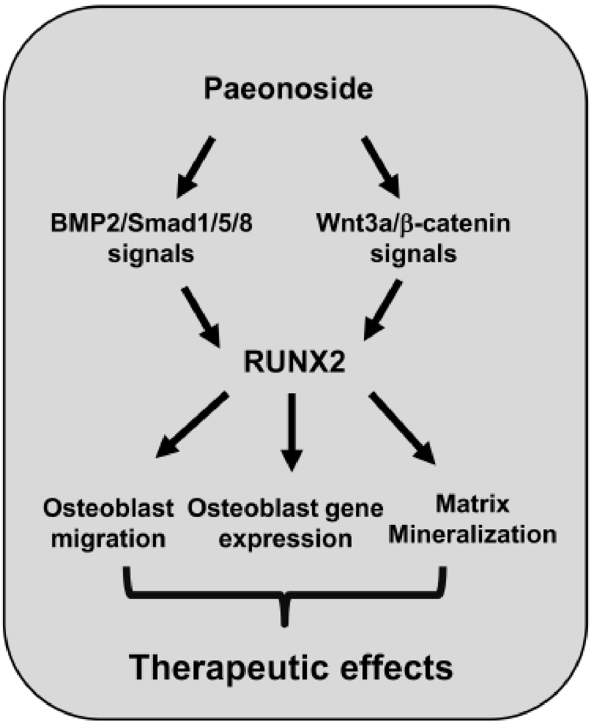


**Figure 8.** Schematic showing PASI-mediated osteoblast differentiation and mineralization.

## 4. Materials and Methods

### *4.1. General Material*

Dried roots of *P. suffruticosa* were purchased from a commercial oriental drug store, Human Herb, Gyoengsan, Korea. The organic solvents methanol (MeOH), n-hexane, ethyl acetate (EtOAc), dichloromethane ($CH_2Cl_2$), and butyl alcohol (BuOH) were obtained from Duksan Chemical (Anseong, Gyeonggi, Korea). The silica gel 60 (230–400 mesh ASTM, Merck, Darmstadt, Germany), ODS-A (12 nm, S-150 m, YMC, Tokyo, Japan), and Sephadex LH-20 column (GE Healthcare, Sweden) were used for open column chromatography. The NMR spectra were recorded on a Jeol ECA-500 spectrometer, operating at 500 MHz for $^1$H and 125 MHz for $^{13}$C NMR spectra. The determination of the HPLC spectrum was recorded on an Agilent 1200 series (Agilent Technologies, Santa Clara, CA, USA) equipped with a photodiode array detector (PDA) and evaporative light scattering detector (ELSD).

### *4.2. Extraction and Isolation from Dried Roots of P. suffruticosa*

Dried roots of *P. suffruticosa* (2 kg) were extracted with 95% MeOH for 2 h (3 × 500 mL). The MeOH extract (386.3 g) was suspended in 1000 mL of distilled water and the solvent was partitioned with same volume of n-Hexane, EtOAc, and BuOH. The BuOH soluble fraction (72.8 g) was separated into 12 fractions (PSB 1~12) by silica chromatography eluted with an isocratic of $CH_2Cl_2$, MeOH, and $H_2O$ (50:1:0.1). The fraction PSB 3 was re-chromatographed on a Sephadex LH-20 column with 30% aqueous MeOH (*v*/*v*) to obtain eight fractions (PSB 3-1~PSB 3-8). The subfraction PSB 3-6 was separated by reversed-phase column chromatography (ODS-A) eluted with 35% aqueous MeOH (*v*/*v*) to obtain Paeonoside (PASI) (160 mg). The structure of PASI was based on spectroscopic data and a comparison with the literature [15].

### *4.3. Paeonoside (PASI)*

Colorless amorphous powder; EI-MS m/z = 328.31 $[M]^+$, molecular formula $C_{15}H_{20}O_8$; $^1$H-NMR (500 MHz, $CD_3OD$) δ: 7.71 (1H, dd, J = 1.4, 8.9 Hz, H-6), 6.83 (1H, s, H-3), 6.62 (1H, dd, J = 2.3, 8.9 Hz, H-5), 5.04 (1H, d, J = 7.8 Hz, H-1′), 3.90 (1H, d, J = 12.1 Hz, H-6′b), 3.82 (3H, s, -OCH3), 3.68 (1H, m, H-6′a), 3.51 (3H, m, H-2′, 5′, 3′), 3.37 (1H, m, H-4′), 2.62 (3H, s, H-8); $^{13}$C-NMR (125 MHz, $CD_3OD$) δ: 200.7 (C-7), 166.4 (C-4), 160.7 (C-2), 133.1 (C-6), 122.4 (C-1), 109.6 (C-5), 102.7 (C-3), 102.6 (C-1′), 78.6 (C-3′), 78.4 (C-5′), 74.9 (C-2′), 71.4 (C-4′), 62.7 (C-6′), 56.3 (4-OCH3), 32.3 (C-8).

### *4.4. Cell Culture of Pre-Osteoblast MC3T3-E1*

MC3T3-E1 cells were purchased from the American Type Culture Collection (ATCC, CRL-2593) (Manassas, VA, USA) and cultured as previously described [17].

### *4.5. Differentiation of Pre-Osteoblast MC3T3-E1*

Differentiation was induced as previously described [17]. Briefly, the cells were incubated in OS containing 50 μg/mL L-AA and 10 mM β-GP, and fresh medium was replaced every two days.

### *4.6. MTT Assay*

Cell viability was measured using an MTT assay to detect NADH-dependent dehydrogenase activity as previously described [17].

### *4.7. Migration Assays*

Wound-healing assay and Boyden chamber assay were carried out as previously described [17].

### *4.8. ALP Activity and Staining Assay*

Osteoblast differentiation was induced using OS containing with PASI for seven days. ALP activity and staining assay were performed as previously described [17].

### *4.9. ARS Staining and Quantification*

Osteoblast differentiation was induced using OS containing PASI for 14 days, and ARS staining and quantification were carried out as previously described [17].

### *4.10. Western Blot Analysis*

Western blot analysis was carried out as previously described [69].

### *4.11. Immunofluorescence Analysis*

Immunofluorescence was carried out as previously described [17].

### 4.12. Statistical Analysis

Data were analyzed using the Prism Version 5 program (GraphPad Software, Inc., San Diego, CA, USA). All numeric values are expressed as the mean ± S.E.M. Differences were considered statistically significant when * $p < 0.05$.

**Author Contributions:** Conceptualization, K.-R.P.; software, K.-R.P.; formal analysis; methodology, K.-R.P. and J.Y.L.; K.-R.P.; investigation, K.-R.P.; validation, K.-R.P. and J.Y.L.; resources, K.-R.P., J.Y.L., J.T.H. and M.C.; writing—original draft preparation, K.-R.P.; writing—review and editing, K.-R.P., J.T.H. and H.-M.Y.; supervision, H.-M.Y.; project administration, H.-M.Y.; funding acquisition, J.T.H. and H.-M.Y. All authors have read and agreed to the published version of the manuscript.

**Funding:** This work was supported by the National Research Foundation of Korea [NRF] grant funded by the Korea government (MSIP) (2017R1A5A2015541; 2018R1D1A1B07043282).

**Institutional Review Board Statement:** Not applicable.

**Informed Consent Statement:** Not applicable.

**Data Availability Statement:** Data is contained within the article.

**Acknowledgments:** We appreciate the Bioevaluation Center (Korea Research Institute of Bioscience and Biotechnology, Korea) for experimental assistance.

**Conflicts of Interest:** The authors declare no conflict of interest.

## Abbreviations

| | |
|---|---|
| ALP | Alkaline phosphatase |
| ARS | Alizarin red S |
| β-GP | β-glycerophosphate |
| BMP | Bone morphogenetic protein |
| L-AA | L-ascorbic acid |
| MTT | 3-[4,5-dimethylthiazol-2-yl]-2,5-diphenyltetrazolium bromide |
| PASI | Paeonoside |
| OS | Osteogenic supplement |
| RUNX2 | Runt-related transcription factor 2 |

## References

1. Feng, X.; McDonald, J.M. Disorders of bone remodeling. *Annu. Rev. Pathol.* **2011**, *6*, 121–145. [CrossRef]
2. Rucci, N. Molecular biology of bone remodelling. *Clin. Cases Miner. Bone Metab.* **2008**, *5*, 49–56.
3. Marie, P.J. Osteoblast dysfunctions in bone diseases: From cellular and molecular mechanisms to therapeutic strategies. *Cell Mol. Life Sci.* **2015**, *72*, 1347–1361. [CrossRef] [PubMed]
4. Esfahanian, V.; Shamami, M.S. Relationship between osteoporosis and periodontal disease: Review of the literature. *J. Dent.* **2012**, *9*, 256–264.
5. Fakhry, M.; Hamade, E.; Badran, B.; Buchet, R.; Magne, D. Molecular mechanisms of mesenchymal stem cell differentiation towards osteoblasts. *World J. Stem Cells* **2013**, *5*, 136–148. [CrossRef] [PubMed]
6. Zheng, X.; Dai, J.; Zhang, H.; Ge, Z. MicroRNA-221 promotes cell proliferation, migration, and differentiation by regulation of ZFPM2 in osteoblasts. *Braz. J. Med. Biol. Res.* **2018**, *51*, e7574. [CrossRef] [PubMed]
7. Beederman, M.; Lamplot, J.D.; Nan, G.; Wang, J.; Liu, X.; Yin, L.; Li, R.; Shui, W.; Zhang, H.; Kim, S.H.; et al. BMP signaling in mesenchymal stem cell differentiation and bone formation. *J. Biomed. Sci. Eng.* **2013**, *6*, 32–52. [CrossRef]
8. Halloran, D.; Durbano, H.W.; Nohe, A. Bone Morphogenetic Protein-2 in Development and Bone Homeostasis. *J. Dev. Biol.* **2020**, *8*, 19. [CrossRef]
9. Moorer, M.C.; Riddle, R.C. Regulation of Osteoblast Metabolism by Wnt Signaling. *Endocrinol. Metab.* **2018**, *33*, 318–330. [CrossRef]
10. Li, Z.; Xu, Z.; Duan, C.; Liu, W.; Sun, J.; Han, B. Role of TCF/LEF Transcription Factors in Bone Development and Osteogenesis. *Int. J. Med. Sci.* **2018**, *15*, 1415–1422. [CrossRef]
11. Komori, T. Regulation of Proliferation, Differentiation and Functions of Osteoblasts by Runx2. *Int. J. Mol. Sci.* **2019**, *20*, 1694. [CrossRef]
12. Zhang, L.; Li, D.C.; Liu, L.F. Paeonol: Pharmacological effects and mechanisms of action. *Int. Immunopharmacol.* **2019**, *72*, 413–421. [CrossRef] [PubMed]

13. Ryu, G.; Park, E.K.; Joo, J.H.; Lee, B.H.; Choi, B.W.; Jung, D.S.; Lee, N.H. A new antioxidant monoterpene glycoside, alpha-benzoyloxypaeoniflorin from Paeonia suffruticosa. *Arch. Pharm. Res.* **2001**, *24*, 105–108. [CrossRef]
14. Chen, G.; Zhang, L.; Zhu, Y. Determination of glycosides and sugars in Moutan Cortex by capillary electrophoresis with electrochemical detection. *J. Pharm. Biomed. Anal.* **2006**, *41*, 129–134. [CrossRef] [PubMed]
15. Ha, D.T.; Trung, T.N.; Hien, T.T.; Dao, T.T.; Yim, N.; Ngoc, T.M.; Oh, W.K.; Bae, K. Selected compounds derived from Moutan Cortex stimulated glucose uptake and glycogen synthesis via AMPK activation in human HepG2 cells. *J. Ethnopharmacol.* **2010**, *131*, 417–424. [CrossRef] [PubMed]
16. Li, G.; Seo, C.S.; Lee, K.S.; Kim, H.J.; Chang, H.W.; Jung, J.S.; Song, D.K.; Son, J.K. Protective constituents against sepsis in mice from the root cortex of Paeonia suffruticosa. *Arch. Pharm. Res.* **2004**, *27*, 1123–1126. [CrossRef]
17. Park, K.R.; Lee, J.Y.; Cho, M.; Hong, J.T.; Yun, H.M. Paeonolide as a Novel Regulator of Core-Binding Factor Subunit Alpha-1 in Bone-Forming Cells. *Int. J. Mol. Sci.* **2021**, *22*, 4924. [CrossRef]
18. An, J.; Yang, H.; Zhang, Q.; Liu, C.; Zhao, J.; Zhang, L.; Chen, B. Natural products for treatment of osteoporosis: The effects and mechanisms on promoting osteoblast-mediated bone formation. *Life Sci.* **2016**, *147*, 46–58. [CrossRef]
19. Histing, T.; Stenger, D.; Kuntz, S.; Scheuer, C.; Tami, A.; Garcia, P.; Holstein, J.H.; Klein, M.; Pohlemann, T.; Menger, M.D. Increased osteoblast and osteoclast activity in female senescence-accelerated, osteoporotic SAMP6 mice during fracture healing. *J. Surg. Res.* **2012**, *175*, 271–277. [CrossRef]
20. Wang, C.J.; McCauley, L.K. Osteoporosis and Periodontitis. *Curr. Osteoporos. Rep.* **2016**, *14*, 284–291. [CrossRef]
21. Irfan, U.M.; Dawson, D.V.; Bissada, N.F. Epidemiology of periodontal disease: A review and clinical perspectives. *J. Int. Acad. Periodontol.* **2001**, *3*, 14–21.
22. Liang, Y.; Luan, X.; Liu, X. Recent advances in periodontal regeneration: A biomaterial perspective. *Bioact. Mater.* **2020**, *5*, 297–308. [CrossRef]
23. Wright, N.C.; Looker, A.C.; Saag, K.G.; Curtis, J.R.; Delzell, E.S.; Randall, S.; Dawson-Hughes, B. The recent prevalence of osteoporosis and low bone mass in the United States based on bone mineral density at the femoral neck or lumbar spine. *J. Bone Miner. Res.* **2014**, *29*, 2520–2526. [CrossRef]
24. Cai, S.; Yu, H.; Li, Y.; He, X.; Yan, L.; Huang, X.; Wang, P. Bone mineral density measurement combined with vertebral fracture assessment increases diagnosis of osteoporosis in postmenopausal women. *Skelet. Radiol.* **2020**, *49*, 273–280. [CrossRef]
25. Lee, W.C.; Guntur, A.R.; Long, F.; Rosen, C.J. Energy Metabolism of the Osteoblast: Implications for Osteoporosis. *Endocr. Rev.* **2017**, *38*, 255–266. [CrossRef] [PubMed]
26. Russow, G.; Jahn, D.; Appelt, J.; Mardian, S.; Tsitsilonis, S.; Keller, J. Anabolic Therapies in Osteoporosis and Bone Regeneration. *Int. J. Mol. Sci.* **2018**, *20*, 83. [CrossRef] [PubMed]
27. Park, K.R.; Kim, S.; Cho, M.; Kang, S.W.; Yun, H.M. Effects of PIN on Osteoblast Differentiation and Matrix Mineralization through Runt-Related Transcription Factor. *Int. J. Mol. Sci.* **2020**, *21*, 9579. [CrossRef]
28. Park, K.R.; Kwon, Y.J.; Park, J.E.; Yun, H.M. 7-HYB, a Phenolic Compound Isolated from Myristica fragrans Houtt Increases Cell Migration, Osteoblast Differentiation, and Mineralization through BMP2 and beta-catenin Signaling. *Int. J. Mol. Sci.* **2020**, *21*, 8059. [CrossRef] [PubMed]
29. Park, K.R.; Leem, H.H.; Cho, M.; Kang, S.W.; Yun, H.M. Effects of the amide alkaloid piperyline on apoptosis, autophagy, and differentiation of pre-osteoblasts. *Phytomedicine* **2020**, *79*, 153347. [CrossRef] [PubMed]
30. Park, K.R.; Lee, J.Y.; Kim, B.M.; Kang, S.W.; Yun, H.M. TMARg, a Novel Anthraquinone Isolated from Rubia cordifolia Nakai, Increases Osteogenesis and Mineralization through BMP2 and beta-Catenin Signaling. *Int. J. Mol. Sci.* **2020**, *21*, 5332. [CrossRef] [PubMed]
31. Park, K.R.; Lee, H.; Cho, M.; Yun, H.M. A Phytochemical Constituent, (E)-Methyl-Cinnamate Isolated from Alpinia katsumadai Hayata Suppresses Cell Survival, Migration, and Differentiation in Pre-Osteoblasts. *Int. J. Mol. Sci.* **2020**, *21*, 3700. [CrossRef]
32. Infante, A.; Rodriguez, C.I. Osteogenesis and aging: Lessons from mesenchymal stem cells. *Stem Cell Res. Ther.* **2018**, *9*, 244. [CrossRef]
33. Granero-Molto, F.; Weis, J.A.; Miga, M.I.; Landis, B.; Myers, T.J.; O'Rear, L.; Longobardi, L.; Jansen, E.D.; Mortlock, D.P.; Spagnoli, A. Regenerative effects of transplanted mesenchymal stem cells in fracture healing. *Stem Cells* **2009**, *27*, 1887–1898. [CrossRef] [PubMed]
34. Ichida, M.; Yui, Y.; Yoshioka, K.; Tanaka, T.; Wakamatsu, T.; Yoshikawa, H.; Itoh, K. Changes in cell migration of mesenchymal cells during osteogenic differentiation. *FEBS Lett.* **2011**, *585*, 4018–4024. [CrossRef] [PubMed]
35. Karsenty, G.; Kronenberg, H.M.; Settembre, C. Genetic control of bone formation. *Annu. Rev. Cell Dev. Biol.* **2009**, *25*, 629–648. [CrossRef]
36. Karsenty, G.; Wagner, E.F. Reaching a genetic and molecular understanding of skeletal development. *Dev. Cell* **2002**, *2*, 389–406. [CrossRef]
37. Guntur, A.R.; Rosen, C.J. The skeleton: A multi-functional complex organ: New insights into osteoblasts and their role in bone formation: The central role of PI3Kinase. *J. Endocrinol.* **2011**, *211*, 123–130. [CrossRef]
38. Lee, H.S.; Jung, E.Y.; Bae, S.H.; Kwon, K.H.; Kim, J.M.; Suh, H.J. Stimulation of osteoblastic differentiation and mineralization in MC3T3-E1 cells by yeast hydrolysate. *Phytother. Res.* **2011**, *25*, 716–723. [CrossRef]
39. Kim, M.B.; Song, Y.; Hwang, J.K. Kirenol stimulates osteoblast differentiation through activation of the BMP and Wnt/beta-catenin signaling pathways in MC3T3-E1 cells. *Fitoterapia* **2014**, *98*, 59–65. [CrossRef] [PubMed]

40. Garcia, J.; Delany, A.M. MicroRNAs regulating TGFbeta and BMP signaling in the osteoblast lineage. *Bone* **2021**, *143*, 115791. [CrossRef]
41. Lotz, E.M.; Berger, M.B.; Boyan, B.D.; Schwartz, Z. Regulation of mesenchymal stem cell differentiation on microstructured titanium surfaces by semaphorin 3A. *Bone* **2020**, *134*, 115260. [CrossRef] [PubMed]
42. Wang, M.; Li, J.; Ye, Y.; He, S.; Song, J. SHED-derived conditioned exosomes enhance the osteogenic differentiation of PDLSCs via Wnt and BMP signaling in vitro. *Differentiation* **2020**, *111*, 1–11. [CrossRef]
43. Miyazono, K.; Kamiya, Y.; Morikawa, M. Bone morphogenetic protein receptors and signal transduction. *J. Biochem.* **2010**, *147*, 35–51. [CrossRef]
44. MacDonald, B.T.; He, X. Frizzled and LRP5/6 receptors for Wnt/beta-catenin signaling. *Cold Spring Harb. Perspect. Biol.* **2012**, *4*, a007880. [CrossRef] [PubMed]
45. Gaur, T.; Lengner, C.J.; Hovhannisyan, H.; Bhat, R.A.; Bodine, P.V.; Komm, B.S.; Javed, A.; van Wijnen, A.J.; Stein, J.L.; Stein, G.S.; et al. Canonical WNT signaling promotes osteogenesis by directly stimulating Runx2 gene expression. *J. Biol. Chem.* **2005**, *280*, 33132–33140. [CrossRef] [PubMed]
46. Rawadi, G.; Vayssiere, B.; Dunn, F.; Baron, R.; Roman-Roman, S. BMP-2 controls alkaline phosphatase expression and osteoblast mineralization by a Wnt autocrine loop. *J. Bone Miner. Res.* **2003**, *18*, 1842–1853. [CrossRef]
47. Chen, G.; Deng, C.; Li, Y.P. TGF-beta and BMP signaling in osteoblast differentiation and bone formation. *Int. J. Biol. Sci.* **2012**, *8*, 272–288. [CrossRef] [PubMed]
48. Artigas, N.; Urena, C.; Rodriguez-Carballo, E.; Rosa, J.L.; Ventura, F. Mitogen-activated protein kinase (MAPK)-regulated interactions between Osterix and Runx2 are critical for the transcriptional osteogenic program. *J. Biol. Chem.* **2014**, *289*, 27105–27117. [CrossRef]
49. Hsu, M.N.; Yu, F.J.; Chang, Y.H.; Huang, K.L.; Pham, N.N.; Truong, V.A.; Lin, M.W.; Kieu Nguyen, N.T.; Hwang, S.M.; Hu, Y.C. CRISPR interference-mediated noggin knockdown promotes BMP2-induced osteogenesis and calvarial bone healing. *Biomaterials* **2020**, *252*, 120094. [CrossRef]
50. Doghman, M.; Cazareth, J.; Lalli, E. The T cell factor/beta-catenin antagonist PKF115-584 inhibits proliferation of adrenocortical carcinoma cells. *J. Clin. Endocrinol. Metab.* **2008**, *93*, 3222–3225. [CrossRef] [PubMed]
51. Sinnberg, T.; Menzel, M.; Ewerth, D.; Sauer, B.; Schwarz, M.; Schaller, M.; Garbe, C.; Schittek, B. beta-Catenin signaling increases during melanoma progression and promotes tumor cell survival and chemoresistance. *PLoS ONE* **2011**, *6*, e23429. [CrossRef]
52. Phimphilai, M.; Zhao, Z.; Boules, H.; Roca, H.; Franceschi, R.T. BMP signaling is required for RUNX2-dependent induction of the osteoblast phenotype. *J. Bone Miner. Res.* **2006**, *21*, 637–646. [CrossRef]
53. Zhang, R.; Oyajobi, B.O.; Harris, S.E.; Chen, D.; Tsao, C.; Deng, H.W.; Zhao, M. Wnt/beta-catenin signaling activates bone morphogenetic protein 2 expression in osteoblasts. *Bone* **2013**, *52*, 145–156. [CrossRef]
54. Lin, G.L.; Hankenson, K.D. Integration of BMP, Wnt, and notch signaling pathways in osteoblast differentiation. *J. Cell Biochem.* **2011**, *112*, 3491–3501. [CrossRef]
55. Liu, T.M.; Lee, E.H. Transcriptional regulatory cascades in Runx2-dependent bone development. *Tissue Eng. Part B Rev.* **2013**, *19*, 254–263. [CrossRef] [PubMed]
56. Kanis, J.A.; Cooper, C.; Rizzoli, R.; Reginster, J.Y. Correction to: European guidance for the diagnosis and management of osteoporosis in postmenopausal women. *Osteoporos. Int.* **2020**, *31*, 801. [CrossRef]
57. Munoz-Garach, A.; Garcia-Fontana, B.; Munoz-Torres, M. Nutrients and Dietary Patterns Related to Osteoporosis. *Nutrients* **2020**, *12*, 1986. [CrossRef] [PubMed]
58. Zheng, X.; Lee, S.K.; Chun, O.K. Soy Isoflavones and Osteoporotic Bone Loss: A Review with an Emphasis on Modulation of Bone Remodeling. *J. Med. Food* **2016**, *19*, 1–14. [CrossRef]
59. Matsumoto, Y.; Tousen, Y.; Nishide, Y.; Tadaishi, M.; Kato, K.; Ishimi, Y. Combined effects of soy isoflavones and milk basic protein on bone mineral density in hind-limb unloaded mice. *J. Clin. Biochem. Nutr.* **2016**, *58*, 141–145. [CrossRef] [PubMed]
60. Tousen, Y.; Ichimaru, R.; Kondo, T.; Inada, M.; Miyaura, C.; Ishimi, Y. The Combination of Soy Isoflavones and Resveratrol Preserve Bone Mineral Density in Hindlimb-Unloaded Mice. *Nutrients* **2020**, *12*, 2043. [CrossRef]
61. Theill, L.E.; Boyle, W.J.; Penninger, J.M. RANK-L and RANK: T cells, bone loss, and mammalian evolution. *Annu. Rev. Immunol.* **2002**, *20*, 795–823. [CrossRef] [PubMed]
62. Lee, D.W.; Kwon, J.Y.; Kim, H.K.; Lee, H.J.; Kim, E.S.; Kim, H.J.; Lee, H.B. Propofol attenuates osteoclastogenesis by lowering RANKL/OPG ratio in mouse osteoblasts. *Int. J. Med. Sci.* **2018**, *15*, 723–729. [CrossRef]
63. Raut, N.; Wicks, S.M.; Lawal, T.O.; Mahady, G.B. Epigenetic regulation of bone remodeling by natural compounds. *Pharmacol. Res.* **2019**, *147*, 104350. [CrossRef] [PubMed]
64. Ghayor, C.; Weber, F.E. Epigenetic Regulation of Bone Remodeling and Its Impacts in Osteoporosis. *Int. J. Mol. Sci.* **2016**, *17*, 1446. [CrossRef]
65. Fu, J.; Wu, H.; Deng, R.; Sun, M. Deciphering the metabolic profile and pharmacological mechanisms of Achyranthes bidentata blume saponins using ultra-performance liquid chromatography quadrupole time-of-flight mass spectrometry coupled with network pharmacology-based investigation. *J. Ethnopharmacol.* **2021**, *274*, 114067. [CrossRef]
66. Zhao, C.; Qiao, X.; Shao, Q.; Hassan, M.; Ma, Z. Evolution of the Lignin Chemical Structure during the Bioethanol Production Process and Its Inhibition to Enzymatic Hydrolysis. *Energy Fuels* **2020**, *34*, 5938–5947. [CrossRef]

67. Soelaiman, I.N.; Das, S.; Shuid, A.N.; Mo, H.; Mohamed, N. Use of medicinal plants and natural products for treatment of osteoporosis and its complications. *Evid Based Complement. Alternat Med.* **2013**, *2013*, 764701. [CrossRef]
68. Whelan, A.M.; Jurgens, T.M.; Bowles, S.K. Natural health products in the prevention and treatment of osteoporosis: Systematic review of randomized controlled trials. *Ann. Pharmacother* **2006**, *40*, 836–849. [CrossRef]
69. Park, K.R.; Kim, E.C.; Hong, J.T.; Yun, H.M. Dysregulation of 5-hydroxytryptamine 6 receptor accelerates maturation of bone-resorbing osteoclasts and induces bone loss. *Theranostics* **2018**, *8*, 3087–3098. [CrossRef] [PubMed]
