# Supplementary material for: Molecular Regulators for the Wnt/β-Catenin Signalling Pathway for Bone Remodelling in Periodontitis: A Scoping Review
Source: Int J Mol Sci. 2026 Oct 14;27(20):9117. doi: 10.3390/ijms27209117 (PMC13705578; doi:10.3390/ijms27209117)
Supplement: Supplementary file 1 [file ijms-27-09117-s001.zip › Supplementary Data/Papers/in vitro/Changes in Dickkopf-1, but Not Sclerostin, in Gingival Crevicular Fluid Are Associated with Peroral Statin Treatment in Patients with Periodontitis.pdf]

medicina

MDPI

Article

Changes in Dickkopf-1, but Not Sclerostin, in Gingival Crevicular Fluid Are Associated with Peroral Statin Treatment in Patients with Periodontitis

Kristina Duspara [1,2,3], Renata Sikora [1,4], Ana Petrovic [1], Lucija Kuna Roguljic [1], Anita Matic [1], Kristina Kralik [2], Hrvoje Roguljic [2], Tomislav Kizivat [2,5], Mirjana Duspara [3], Dunja Igrec [1], Kristina Bojanic [1,2,4], Robert Smolic [1], Aleksandar Vcev [1], Magdalena Wyszyńska [6], George Y. Wu [7] and Martina Smolic [1,*]

1 Faculty of Dental Medicine and Health Osijek, J. J. Strossmayer University of Osijek, 31000 Osijek, Croatia; dusparakristina5@gmail.com (K.D.); rsikora@fdmz.hr (R.S.); anapetrovic@fdmz.hr (A.P.); lkuna@fdmz.hr (L.K.R.); anita.matic@fdmz.hr (A.M.); digrec@fdmz.hr (D.I.); kbojanic@mefos.hr (K.B.); rsmolic@fdmz.hr (R.S.); avcev@fdmz.hr (A.V.)
2 Faculty of Medicine Osijek, J. J. Strossmayer University of Osijek, 31000 Osijek, Croatia; kristina.kralik@mefos.hr (K.K.); hrvoje.roguljic@mefos.hr (H.R.); tkizivat@mefos.hr (T.K.)
3 Public Health Scientific Institution Medical Center "Dr. Mustafa Sehovic", 75000 Tuzla, Bosnia and Herzegovina; duspara.mirjana@gmail.com
4 Health Center Osijek-Baranja County, 31000 Osijek, Croatia
5 University Hospital Centre Osijek, 31000 Osijek, Croatia
6 Division of Medical Sciences in Zabrze, Medical University of Silesia in Katowice, 15 Poniatowskiego Street, 40-055 Katowice, Poland; magdalena.wyszynska@sum.edu.pl
7 University of Connecticut Health Center, Farmington, CT 06030, USA; wu@uchc.edu
* Correspondence: msmolic@fdmz.hr

check for updates

Citation: Duspara, K.; Sikora, R.; Petrovic, A.; Kuna Roguljic, L.; Matic, A.; Kralik, K.; Roguljic, H.; Kizivat, T.; Duspara, M.; Igrec, D.; et al. Changes in Dickkopf-1, but Not Sclerostin, in Gingival Crevicular Fluid Are Associated with Peroral Statin Treatment in Patients with Periodontitis. *Medicina* **2024**, *60*, 508. https://doi.org/10.3390/medicina60030508

Academic Editor: Bruno Chrcanovic

Received: 1 February 2024
Revised: 14 March 2024
Accepted: 15 March 2024
Published: 20 March 2024

Copyright: © 2024 by the authors. Licensee MDPI, Basel, Switzerland. This article is an open access article distributed under the terms and conditions of the Creative Commons Attribution (CC BY) license (https://creativecommons.org/licenses/by/4.0/).

Abstract: *Background and Objectives*: Periodontitis is marked by the destruction of alveolar bone. Sclerostin (SOST) and dickkopf-1 (DKK-1) act as inhibitors of the Wingless-type (Wnt) signaling pathway, a key regulator of bone metabolism. Recent studies have suggested that statins play a role in bone resorption and formation by influencing Wnt signaling. The aim of this study was to determine the levels of SOST and DKK-1 in periodontal patients with and without peroral statins treatment in their therapy. *Materials and Methods:* A total of 79 patients with diagnosed periodontitis were divided into two groups: 39 patients on statin therapy (SP group) and 40 patients without statin therapy as a control group (P group). The periodontal clinical examination probing (pocket) depth (PD) and gingival recession (GR) were measured, and approximal plaque was detected, while vertical and horizontal bone resorption was measured using a panoramic radiograph image. Clinical attachment loss (CAL) values were calculated using PD and GR values. Gingival crevicular fluid (GCF) was collected and used for measuring SOST and DKK-1 levels. A questionnaire was used to assess lifestyle habits and statin intake. Patients' medical records were used to obtain biochemical parameters. *Results:* There was no significant difference in sclerostin concentration between the SP and P group. DKK-1 values were significantly higher in the SP group compared to the control group ($p = 0.04$). Also, PD ($p = 0.001$) and GR ($p = 0.03$) were significantly higher in the SP group. The level of DKK-1 had a positive relationship with the PD, the greater the PD, the higher the level of DKK-1 (Rho = 0.350), while there was no significant association with other parameters. *Conclusions:* Peroral statins in periodontal patients are associated with GCF levels of DKK-1 but not with sclerostin levels.

Keywords: DKK-1; GCF; periodontitis; sclerostin; statins; Wnt signaling pathway

1. Introduction

Periodontitis is a chronic inflammatory disease, characterized by the progressive destruction of supporting structures of the teeth, primarily alveolar bone, gingiva, and

periodontal ligament. The underlying pathophysiological mechanisms of periodontitis involve a complex interplay of periodontal pathogens and host immune response, as well as environmental factors such as lifestyle habits. In general, specific pathogenic bacteria (dental plaque biofilm) in the oral cavity initiate the host immune response, resulting in the release of proinflammatory cytokines and matrix metalloproteinases (MMPs). This immune response leads to sustained inflammation, while the increased activity of MMPs causes degradation of the extracellular matrix, resulting in destruction of the periodontal soft tissue and alveolar bone [1–3]. Finally, as this process progresses, periodontitis manifests clinically as inflammation of the gingiva and gingival bleeding upon stimulus (probing), periodontal pockets and gingival recession development, resorption of alveolar bone, and clinical attachment loss (CAL), ultimately leading to tooth mobility and loss [1,4].

1.1. Sclerostin (SOST) and Dickkopf-1 (DKK-1) in Periodontitis Pathogenesis

Bone tissue is constantly adapting to biomechanical stress and pathophysiological processes in various disorders via the synergism of bone resorption and formation. The Wingless-type (Wnt) signaling pathway and its inhibitors sclerostin (SOST) and dickkopf-1 (DKK-1) have been identified as central regulators of bone and cartilage formation [5,6], while dysregulation of this signaling pathway has been implicated in many bone tissue disorders such as osteoporosis [7,8], as well as periodontitis [9,10]. As it has been shown that alveolar bone metabolism has a 30–35% annual bone turnover rate [11], these findings imply that Wnt signaling and its manipulation are essential and a promising method for maintaining and regenerating alveolar bone homeostasis [12].

The Wnt signaling pathway [13,14] as well as its inhibition [15] during various stages of odontogenesis have been shown to dysregulate proliferation, differentiation, and epithelial–mesenchymal interaction in tooth root formation and early development of the teeth [12,16,17].

The glycoprotein SOST, produced by osteocytes [18], binds to LRP-5, suppresses osteoblastogenesis, and blocks the Wnt pathway and signals of bone morphogenetic protein (BMP) [9]. Moreover, targeting sclerostin in the periodontal mineralizing ligament can stimulate periodontal tissue regeneration [14]. DKK-1 with anti-anabolic effect is associated with bone resorption, however, with processes of mucosal soft tissue healing, as well [14] showing the need for further elucidation. Both DKK-1 and SOST have been shown to be up-regulated in chronic periodontal patients [10,19,20]. Hence, biomarkers such as DKK-1 and SOST emerge as promising candidates as biomarkers, as well as pharmacotherapeutic targets.

While regenerative medicine is considered promising and the most researched approach to treating periodontitis [21], alternative possibilities of Wnt signaling modulation have been demonstrated recently. Particularly statin-mediated regulation of the Wnt pathway, its inhibitors, and bone metabolism-altering effects have been extensively researched as potential biomarkers and pharmacotherapeutic approaches in various disorders.

1.2. Statins as Bone Metabolism Modulators

Numerous studies have indicated that statins, lipid-lowering agents, exhibit a wide range of health benefits beyond their cholesterol reduction, particularly in modulating bone metabolism. Therefore, their potential has been implicated as beneficial in systemic bone disorders as well as in periodontitis [22]. Recent studies have demonstrated their role in bone resorption inhibition [22]. Specifically, statins have been shown to inhibit the osteoblasts' apoptosis, which prevents bone resorption [23,24] and fractures [25]. Also, it has been found that they exert pleiotropic antioxidant and anti-inflammatory properties, resulting in statin-mediated reduction of reactive oxidative species (ROS) and C reactive protein (CRP) [22,26].

A plethora of recent studies have found compelling evidence of statin therapeutic potential in modulating the Wnt signaling pathway [27] in cardiovascular and metabolic

diseases [28], cancer [29–31], and neurological disorders [32], as well as bone disorders, such as osteoporosis [33,34] and fracture healing [35], as well as in periodontitis [9,36–38].

Finally, recent studies have implicated the statin-mediated regulation of DKK-1 and SOST activity as a mechanism of their Wnt pathway-modulating effects [28,39,40]. However, the evidence of their corellation remains scarce, especially in the context of periodontitis.

Both DKK-1 and SOST are found in gingival crevicular fluid (GCF), an inflammatory exudate localized between the tooth and the gingival margin [41]. The complexity of the GCF composition is reflected in the presence of numerous other biomarkers such as proteins, cytokines, lipid compounds, and enzymes which can be valuable for the diagnosis and prognosis of periodontal disease. The presence of these components and their different concentrations in the GCF during the periodontal inflammatory process imply the potential of utilizing GCF for the detection of periodontitis biomarkers in its early diagnosis and prognosis [42].

The aim of this study was to determine the levels of the Wnt inhibitors SOST and DKK-1 in periodontal patients' GCF with and without peroral statins, as well as to investigate the association of these proteins with clinical parameters and lifestyle habits.

2. Materials and Methods

2.1. Study Design

The research was designed as a case–control study. The study protocol was approved by the Ethical Committee for Research at the Public Health Scientific Institution Medical Center Dr. Mustafa Sehovic Tuzla (Ethical Approval Code: 16-01-2378-2/22). All participants in the research signed an informed consent form before participation. The research was conducted at the Public Health Scientific Institution Medical Center Dr. Mustafa Sehovic Tuzla and Laboratory for Translational Medicine at the Faculty of Dental Medicine and Health, University of Osijek, from January to June 2023.

2.2. Participants

A total of 79 patients from the Public Health Scientific Institution Medical Center Dr. Mustafa Sehovic Tuzla were included in the study and were divided into two groups. The research group consisted of 39 subjects diagnosed with periodontitis with statin therapy (SP group), while the control group consisted of 40 subjects diagnosed with periodontitis without statin therapy (P group). The control group was matched to the case group with similar sociodemographic features and clinical parameters.

Inclusion criteria: Diagnosed periodontitis indicated for clinical treatment with PD $\geq$ 3 mm in more than two teeth or CAL $\geq$ 3 mm in non-adjacent teeth according to guidelines [4], patients not taking statins and those on statin therapy for at least six months. CAL values were calculated in mm based on PD and GR values [43]. The criteria for excluding patients from the study were a history of diagnosed gastrointestinal, liver, or kidney disease with chronic therapy included for those diseases, corticosteroid use for more than 3 months (chronic therapy) or drugs for the treatment of osteoporosis [9,18]. Also, patients with PD < 3 mm and CAL < 3 mm, patients whose gums were bleeding during examination and paper point application, and patients receiving statins for less than 6 months were excluded. Each patient was graded based on the periodontitis disease grade and stage according to most recent guidelines [4].

Basic characteristics of participants including data on gender, age, occupation and professional training, height and weight, smoking, presence of diabetes mellitus, physical activity, and statin therapy (which type and dose) were collected by a questionnaire.

Biochemical analysis for lipid status, as well as CRP and glycated hemoglobin (HbA1c) values, were obtained from the patients' medical records (for both groups). Biochemical results for lipid status were based on the values of cholesterol (mmol/L), triglycerides (mmol/L), LDL (mmol/L), and HDL (mmol/L).

During the periodontal clinical examination, PD and GR were measured and approximal plaque was detected, while vertical and horizontal bone resorption was measured using

a panoramic radiograph image. PD was measured with a periodontal probe graduated in millimeters. All sides of the tooth (oral, vestibular, mesial, and distal) were measured and GCF was sampled from the deepest pocket (≥3 mm). GR was measured with a periodontal probe from the cemento-enamel junction (CEJ) to the free edge of the gingiva, and the values are expressed in millimeters. The presence of approximal plaque was detected by coating the approximal surfaces of the teeth with gentian violet, and the staining of the approximal surface was a sign of the presence of plaque.

All patients were examined by the same team of periodontology specialists who established the presence of periodontitis in accordance with known diagnostic guidelines [4,44].

2.3. Gingival Crevicular Fluid (GCF) Sampling

GCF sampling was performed in the Specialist Periodontology Clinic at the Public Health Scientific Institution Medical Center Dr. Mustafa Sehovic in Tuzla according to the protocol conducted in two studies by Guentsch [45] and Rezaei Esfarhood [19]. The working area where the teeth with the highest pocket depth values were recorded during the clinical examination was isolated with cotton rolls and air-dried before GCF sampling. A sterile, appropriate-size paper point based on the depth of the periodontal pocket was applied with sterile tweezers to the limit of minimal resistance and was measured for 30 s to soak it with GCF. All paper points with visible signs of blood were discarded and the measurement was repeated after 15 min. The paper points were placed into an Eppendorf test tube with 500 µL of 0.9% NaCl solution and stored overnight at +4 °C. After a minimum of 12 h, the sample was centrifuged at 400 g/4 min, paper points were separated, and the supernatant was stored at −20 °C and then transferred at −80 °C until laboratory analysis. For each patient, we took a GCF sample in two tubes, as well as three paper points in each to collect as much fluid as possible. Patients were examined in the morning (from 07:00 to 10:00 a.m.). It has been shown previously that GCF levels do not change from 08:00 a.m. to 06:00 p.m. [46]. In our study, we included patients with diagnosed periodontitis who did not have a periodontological procedure for a period of 6 months. Patients whose GCF samples were contaminated with blood (mostly due to increased current inflammation) were excluded.

2.4. DKK-1 ELISA

The GCF concentration of DKK-1 was measured by an ELISA kit (Elabscience®, Houston, TX, USA) according to the manufacturer's instructions. The GCF samples were transferred from −80 °C to −20 °C the day before and taken out to room temperature on the day of the research, then briefly vortexed.

Absorbance was determined spectrophotometrically. Per the manufacturer's instructions, a standard curve was created using absorbance values from which the concentration of DKK-1 expressed in pg/mL was obtained for each sample in duplicate. The analysis was conducted in the Laboratory for Translational Medicine at the Faculty of Dental Medicine and Health, University of Osijek.

2.5. SOST ELISA

The GCF concentration of SOST was measured by an ELISA kit (Elabscience®, Houston, USA) according to the manufacturer's instructions. The GCF samples were transferred from −80 °C to −20 °C the day before and taken out to room temperature on the day of the research, then briefly vortexed.

Absorbance was determined spectrophotometrically. Per the manufacturer's instructions, a standard curve was created using absorbance values from which the concentration of SOST expressed in pg/mL was obtained for each sample in duplicate. The analysis was conducted in the Laboratory for Translational Medicine at the Faculty of Dental Medicine and Health, University of Osijek.

2.6. Statistical Analysis

Power analysis: According to previous studies [9,10,45,47], to observe an effect of d = 0.65 in the difference of numerical variables between two independent groups, with a significance level of 0.05 and a power of 0.80, the minimum required sample size is 78 patients (39 who received statins and have periodontitis and 39 patients only with periodontitis without statin therapy).

Absolute and relative frequencies were used to present categorical data. The Shapiro–Wilk test was used to test the normality of distribution of the continuous variables. The median and the interquartile range (IQR) was used for continuous data description. The Mann–Whitney U test was used to compare the median between two groups, while the Chi-square test and Fisher's exact test were used to analyze the differences between proportions. The association between non-normally distributed variables was determined by the Spearman's rho test. All p values were two-sided. The level of significance was set at alpha of 0.05. The statistical analysis was performed using MedCalc® Statistical Software version 22.006 (MedCalc Software Ltd., Ostend, Belgium; https://www.medcalc.org; 2023).

3. Results

The research was conducted on 79 respondents, of whom 40 (51%) had periodontitis, and 39 (49%) had periodontitis while taking statins. Regarding gender, 54 (68%) were women. The median age of the respondents was 59 years, ranging from a minimum of 18 to a maximum of 83 years. Patients were divided into stages and grades (Table 1) of periodontitis according to the newest guidelines [4], the majority of patients in both groups were in Stage IV, in the P group the majority were in Grade B and in SP in Grade C (Table 1). There were significantly older respondents who took statins in addition to periodontitis (62 years vs. 54 years) (Mann–Whitney U test, $p < 0.001$). The median body mass index (BMI) was 26.93 kg/m^2, ranging from a minimum of 15.87 kg/m^2 to a maximum of 24.29 kg/m^2. A total of 61 (76%) respondents had a secondary vocational education. Oral hygiene was average for 35 (44%) respondents, and poor for 14 (18%). One (1%) subject had liver damage, two (3%) had kidney damage, and nine (11%) had diabetes, with significantly more subjects taking statins (Fisher's exact test, $p = 0.03$). The most common drug for diabetes was metformin. Of the respondents, 28 (36%) smoked cigarettes, of whom 16 (57%) smoked 11–20 cigarettes per day, 7 (9%) consumed alcohol at least once a week, and 44 (57%) engaged in physical activity, most commonly walking (Table 2).

Table 1. Patients divided in periodontal stages and grades.

	Number (%) of Respondents			
	**Periodontitis (n = 40)**	**Periodontitis + Statins) (n = 39)**	**Total (n = 79)**	***p* ***
Stage				
II	8 (20)	1 (2.6)	9 (11.4)	
III	11 (27.5)	17 (43.6)	28 (35.4)	0.03
IV	21 (52.5)	21 (53.8)	42 (53.2)	
Grade				
A	8 (20)	2 (5.1)	10 (12.7)	
B	21 (52.5)	18 (46.2)	39 (49.4)	0.05
C	11 (27.5)	19 (48.7)	30 (38)	

* Chi-square test.

Out of the total number of subjects included in study, 39 (49.4%) had been taking statins for longer than 6 months. There were a total of 39 patients in the SP group. The most common choice was rosuvastatin or atorvastatin, while one respondent took simvastatin, and two (5%) respondents took monacolin K/lovastatin. In both groups, approximal plaque was found in 60 (75.9%) subjects, vertical bone resorption in 39 (49.4), horizontal bone resorption in 79 (100), and elevated CRP values were recorded in 8 (10.1%) respondents (Table 3).

Table 2. Basic characteristics of respondents.

	Periodontitis	Periodontitis + Statins	Total	*p* Value
Gender [n (%)]				
Men	12 (30)	13 (33)	25 (32)	0.75 *
Women	28 (70)	26 (67)	54 (68)	
Age (years) [Median (IQR)]	54 (45.25–60.5)	62 (59–67)	59 (48–64)	<0.001 ‡
Body mass (kg) [Median (IQR)]	75 (65.5–86.75)	83 (70–90)	80 (70–90)	0.17 ‡
Body height (cm) [Median (IQR)]	168 (165–174.75)	170 (165–174)	170 (165–174)	0.74 ‡
BMI (kg/m^2) [Median (IQR)]	25.9 (23.08–28.97)	28.33 (25.1–31.14)	26.93 (24.24–30.49)	0.05 ‡
Level of education [n (%)]				
Primary Education	31 (78)	29 (74)	60 (76)	0.85†
Secondary Education	2 (5)	2 (5)	4 (5)	
Higher Secondary Education	6 (15)	5 (13)	11 (14)	
University Education	1 (2)	3 (8)	4 (5)	
Oral hygiene [n (%)]				
Good	18 (45)	12 (31)	30 (38)	0.09 *
Average	13 (33)	22 (56)	35 (44)	
Bad	9 (23)	5 (13)	14 (18)	
Diagnosed liver damage [n (%)]	1 (3)	0	1 (1)	>0.99 †
Diagnosed kidney damage	2 (5)	0	2 (3)	0.49 †
Diabetes mellitus [n (%)]	1 (3)	8 (21)	9 (11)	0.01 †
Medicine for diabetes mellitus [n (%)]				
Metformin	1/1	5/8	6/9	>0.99 †
Insulin	0	2/8	2/9	
Glimepiride	0	1/8	1/9	
Smoking cigarettes [n (%)]	18 (46)	10 (26)	28 (36)	0.06 *
How many cigarettes a day [n (%)]				
Up to 10	7 (39)	4 (40)	11 (39)	>0.99 *
11–20	10 (56)	6 (60)	16 (57)	
20 or more	1 (6)	0 (0)	1 (4)	
Alcohol drinking [n (%)]	4 (10)	3 (8)	7 (8)	>0.99 *
How much alcohol per week [n (%)]				
1x week	4/4	3/3	7/7	-
Physical activity [n (%)]	24 (63)	19 (50)	43 (57)	0.25 *
Which physical activity				
Walking	16 (64)	18 (95)	34 (77)	0.11 †
Running	3 (12)	0 (0)	3 (7)	
Cycling	2 (8)	0 (0)	2 (5)	
Agricultural work	4 (16)	1 (5)	5 (11)	

* χ^2 test; † Fisher's exact test; ‡ Mann–Whitney U test. BMI—body mass index.

Mean and range measures of lipid status, CRP, and HbA1c for both groups of patients are shown in Table 4.

There were significant higher HbA1c values in the SP group, which is consistent with more frequent B and C grades.

There was no significant difference in sclerostin concentration between the groups, while DKK-1 values were significantly higher in the SP group compared to the P group (Mann–Whitney U test, $p = 0.04$). PD (Mann–Whitney U test, $p = 0.001$), GR (Mann–Whitney U test, $p = 0.03$), and CAL (Mann–Whitney U test, $p = 0.001$) were significantly higher in the SP group (Table 5).

Table 3. Clinical characteristics of subjects.

	Total Number (%) of Respondents
Approximal plaque	60 (75.9)
Vertical bone resorption	39 (49.4)
Horizontal bone resorption	79 (100)
Elevated CRP values	8 (10.1)

CRP—C-reactive protein.

Table 4. Lipid status, CRP, and HbA1c values in the group of subjects taking statins.

	Median (Interquartile Range) According to Groups		Differences (95% CI)	*p* *
	Periodontitis	Periodontitis + Statins		
Total cholesterol (mmol/L) [n = 19:37]	5.70 (5.13–6.87)	5.34 (4.19–6.2)	−0.80 (−1.5 to 0.08)	0.07
Triglycerides (mmol/L) [n = 19:37]	1.52 (1.16–2.42)	1.41 (1.09–2.36)	−0.08 (−0.45 to 0.34)	0.72
LDL (mmol/L) [n = 12:30]	3.64 (3.02–4.33)	2.80 (1.77–3.71)	−0.98 (−1.77 to −0.15)	0.02
HDL (mmol/L) [n = 12:30]	1.20 (1.09–1.50)	1.31 (1.12–1.73)	0.08 (−0.18 to 0.33)	0.40
CRP [n = 4:4]	4.4 (3.95–56.8)	3.9 (0.73–37.83)	−3.1	0.69
HbA1c [n = 3:16]	5.1	6.6 (5.83–8.13)	1.4	0.01

* Mann–Whitney U test; LDL—low-density lipoprotein; HDL—high-density lipoprotein; HbA1c—glycated hemoglobin.

Table 5. Differences in sclerostin and DKK-1 values.

	Median (Interquartile Range) According to Groups		Differences (95% CI)	*p* *
	Periodontitis	Periodontitis + Statins		
PD (mm)	5 (4–6)	6 (5–7)	1 (0 to 2)	0.001
GR (mm)	2 (1–3)	3 (2–3)	1 (0 to1)	0.03
CAL (mm)	6.5 (5–8.5)	8 (7–10)	2 (1 to 3)	0.001
Sclerostin	15.43 (5.79–27.2)	14.36 (6.50–37.21)	0 (−7.14 to 8.57)	0.96
DKK-1	4.71 (2.84–6.64)	5.84 (4.31–7.91)	1.33 (0 to 2.4)	0.04

* Mann–Whitney U test; PD—probing depth; GR—gingival recession; CAL—clinical attachment loss; DKK-1—dickkopf-1.

Using Spearman's correlation coefficient, we evaluated the association of DKK-1 and sclerostin with age, BMI, lipid status, HbA1c, PD (mm), and GR (mm). We observed that the level of DKK-1 had a significant positive relationship with the depth of probing; that is, the greater the depth of probing, the higher the level of DKK-1 and vice versa (rho = 0.350), while there was no significant association of DKK-1 with other values. There was no significant correlation between sclerostin levels and the observed periodontal parameters (Table 6 and Figure 1).

Table 6. Correlation between DKK-1 and sclerostin with observed variables in patients on statins (Spearman's correlation coefficient).

	Spearman's Correlation Coefficient Rho (*p* Value)	
	**Sclerostin**	**DKK-1 Keep It Hyphen**
Age	−0.108 (0.51)	−0.097 (0.56)
BMI	−0.293 (0.07)	−0.175 (0.29)
Total cholesterol	−0.118 (0.49)	0.052 (0.76)
Triglycerides	−0.221 (0.19)	−0.086 (0.61)
LDL	−0.031 (0.87)	0.156 (0.41)
HDL	0.142 (0.45)	0.129 (0.50)
HbA1c	0.149 (0.58)	−0.096 (0.72)
PD	−0.026 (0.88)	0.350 (0.03)
GR	−0.084 (0.61)	0.186 (0.26)

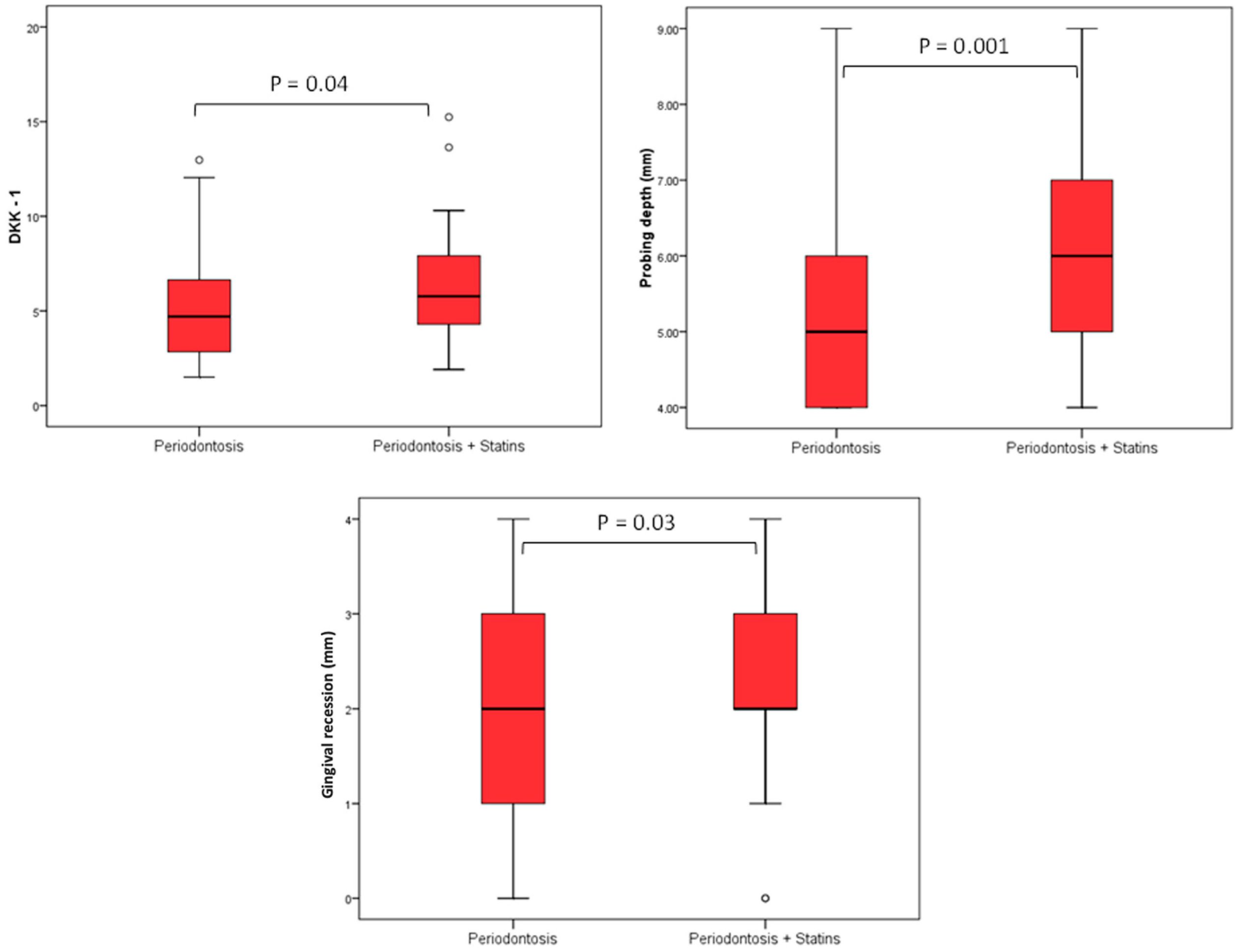

Figure 1. Differences in DKK-1 values, PD, and GR in relation to groups. The level of DKK-1 had a significant positive relationship with the PD; that is, the greater the PD the higher the level of DKK-1 (rho = 0.350), while there was no significant association of DKK-1 with other values, and this follows in Figure 2 as well.

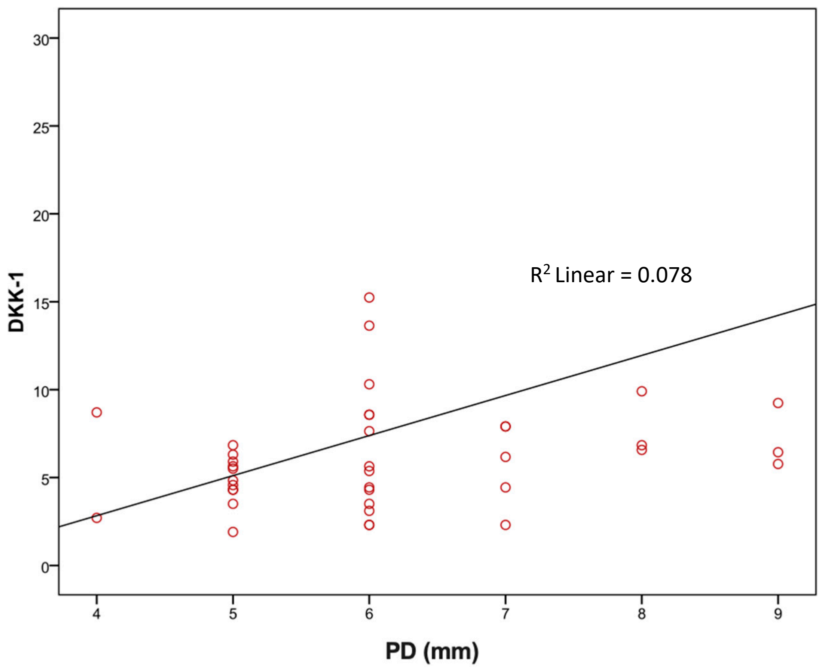

Figure 2. Correlation of PD with DKK-1 (rho = 0.350; p = 0.03).

4. Discussion

According to our knowledge, this is the first study that combines the described method of GCF sampling with the analysis of Wnt proteins in periodontitis patients as well as periodontitis patients taking statins. The research was designed first to determine the concentrations of DKK-1 and sclerostin from GCF in both groups of patients. The second part of the research combined data on BMI, lifestyle habits, level of education, and metabolic diseases, while the third part was based on oral hygiene, clinical periodontal parameters, and biochemical findings related to lipid status, as well as the type of statin used in SP.

Currently widely used traditional assessment tools, such as PD, CAL, and imaging methods, lack insight into the current activity and state of disease. In this study, we calculated that CAL values are significantly higher in the SP group (Table 5). We also used CAL values and data regarding the presence of approximal plaque and horizontal and vertical bone resorption, as well as smoking and diabetes, to define periodontal stages and grades for each patient (Table 1). The results showed that in the total number of respondents, the majority are in Stage IV (53.2%) which is advanced periodontitis and in Grade B (49.4%) as well as in the P group Stage IV and Grade B, but not in the SP group where the majority of patients are in Grade C (48.7%) (Table 1). With staging, it is important to assess the severity of periodontitis and tissue damage, as well as how clinical treatments can improve balance in the oral cavity, while grading is important to estimate the risks and impact on overall health [4]. In the SP group, there were more diabetics and smokers (the majority were consuming more than 10 cigarettes/day) and the values of HbA1c were higher than 7.0% which all indicated that these patients can expect a rapid progression of periodontitis. Biological fluids-based molecular diagnostics have been investigated in the recent decade, reflecting the pathophysiological process and providing insight into the periodontitis state to a much greater extent than traditional tools [48]. Wnt signaling, due to its well-established role in bone homeostasis and periodontal tissues regulation, as well as its inhibitors DKK-1 and sclerostin have recently shown potential as therapeutic targets for periodontitis [12]. Due to statins' ability to regulate inflammation and alveolar bone metabolism, their interaction with Wnt signaling pathways has been observed and investigated with the aim of extending therapeutic options for periodontitis control [49]. The results of our study, comparing DKK-1 levels in the GCF between statin-treated patients and those with no statin therapy, showed notably higher levels of DKK-1 in the SP

group compared to the ones without antilipemic therapy. Previous in vitro studies mostly investigated DKK-1 and statins' correlation in cancer research and found statin-mediated downregulation of DKK-1 protein in endothelial and breast cancer cell lines [50]. To our knowledge, no in vitro study has been conducted in bone cell lines for the purposes of investigating statin-mediated DKK-1 regulation. However, murine models of periodontitis investigating the direct effects of statins on Wnt pathways and DKK-1 levels showed reduced statin-mediated DKK-1 expression levels, which is in accordance with our results in humans [37]. It was also observed that the level of DKK-1 had a significant positive relationship with the PD; that is, the greater the PD the higher the level of DKK-1 (Figure 2), while there was no significant association of DKK-1 with other clinical parameters. There was no significant association of sclerostin with the observed variables (Table 6). Therefore, our results also provide valuable evidence supporting the existing data regarding the correlation of DKK-1 with the severity of periodontitis.

Ozden et al. observed that periodontal patients had higher levels of DKK-1 and that sclerostin was even higher in more severe cases [9]. However, the latter was not found in the current study, where the difference was not significant between groups for sclerostin. Also, contrary to the mentioned study, the values of DKK-1 were elevated in the SP group and statins did not decrease DKK-1 levels, probably due to the presence of more patients with a higher grade of disease and diabetes in the SP group compared with the P group This is consistent with a study by Crandall et al. [51] which concluded that statins can increase the risk of diabetes (10–12%) when compared to a placebo, as well as Corrao et al. which stated that even for the development of a new state of diabetes, statins (all doses) can increase the risk of developing diabetes by 32% [52]. In another study where diabetes was diagnosed prior to statin therapy, statins enhanced the diabetes risk with patients who already had a risk of developing diabetes [53]. A period of 6 months' statin intake is probably not sufficient for this severe (Stage III) periodontitis with the ability of losing teeth and advanced (Stage IV) periodontitis with serious potential of losing teeth and dentation, in order to significantly lower DKK-1 levels.

We also noticed that the majority of patients in the current study were middle-aged females. The median BMI was in the overweight range, and that there were significantly older subjects in the SP group. Although liver and kidney disease was an exclusion criteria, three patients were not excluded—one patient reported liver damage, while two patients reported kidney damage; however, using patients records, it was determined that these patients had no current kidney or liver condition, no accompanying therapy, and had the damage at a younger age. Although statin-associated liver injury is not an uncommon occurrence [54], other causes such as alcohol use and obesity suggested that the liver issues were unrelated to the therapy. There are significantly more patients in the SP group with diagnosed diabetes, mostly on metformin. This agrees with previous research and conclusions that diabetes leads to complications in the periodontia, causing inflammatory processes and prolonging the duration of osteoclastogenesis, thus leading to alveolar bone resorption [55,56]. We noticed that the clinical parameters of periodontitis were worse in the SP group which correlates with this statement. Alcohol consumption was not a frequent phenomenon among patients, but it was expressed in both groups. It is also important that oral hygiene was average to poor in the SP group and most patients smoked cigarettes especially in the P group. This is an expected outcome considering that cigarettes damage the tissue of the oral mucosa which can lead to constant inflammatory processes [56]. Smokers were present in both groups, but we noticed that these are the patients with the worst hygiene and clinical parameters of periodontitis. Therefore, these results of our research agree with studies where the worsening of periodontitis was observed in smokers [55,56]. In the case–control study by Battancs et al., it was noted that the process of cigarette smoke on the gums can be compared to repeated chemical-aggressive tissue attacks, which, if damaged as in periodontitis, cause the formation of the most severe forms of periodontitis [56].

It was expected that most patients have approximal plaque and vertical and horizontal bone resorption as well as PD and GR. In the SP group, there were significantly higher levels of PD and GR. This result is not surprising given the contradiction of additional information related to the reduction or increased value of periodontal clinical parameters in statin patients, which is supported by the review article from 2021 by Di Spirito et al. [57]. In the SP group, CRP levels were low, while periodontal clinical parameters were significantly expressed in the majority of patients. From the results shown in Table 3, we can conclude that the inflammatory status of patients is relatively calm in the advanced stage of periodontitis considering the incidence of bone resorption probably due to systematic statin administration. Therefore, we can conclude the possible effectiveness of anti-inflammatory systematic statin application, which could have a good benefit with the occasional intervention of local statin administration in order to maintain a good periodontal status.

Biochemical parameters related to the level of total cholesterol, LDL, and triglycerides, as well as the values of CRP, are higher in the P group. In our SP group, the CRP level are within reference levels, which can be positively correlated with the anti-inflammatory and antioxidant effects of statins as also previously mentioned by Zhang et al. [38].

For further research in this area, it is important to highlight the limitations of our study, primarily the small sample size and the differences between the groups with and without statin therapy. Our study would greatly benefit and the results would be more relevant if our groups were matched for age, chronic conditions, and other characteristics. With our sample, we were not able to achieve this. The complete elimination of patients with other systemic conditions, however, is challenging to achieve in patients with statin therapy, mostly since statins are rarely used for one isolated condition, and more often for the treatment of multiple diseases or their comorbidities. Nevertheless, we believe that despite using a convenient sample, the results of our study should not be negligible, and considering previous knowledge, they show promising results for further research in this area. However, for a better relevance, the study should be designed as a multicenter study with a larger sample size and normalized groups.

Another limitation of the study is whether and to what extent the patients were adherent to their statin therapy. However, the information about patients' regular statin intake for the minimum of a 6 month period were obtained on the basis of personal statements, as well as on the basis of regular lipid status controls from the patients record during the statin intake period. In order for the results of this future study to be as significant as possible, we suggest monitoring the condition of the same patients before and after non-surgical periodontal treatment.

5. Conclusions

The evidence from this study is twofold. Firstly, GCF sampling with paper points resulted in inexpensive and efficient detection of DKK-1 and SOST in periodontal patients. Secondly, our study demonstrated significantly higher DKK-1 values in patients with statin therapy. Also, the correlation of DKK-1 with PD as a clinical parameter of periodontitis in patients with statin therapy in our study is significant; that is, the greater the PD, the higher the level of DKK-1. No significant association was found between DKK-1 with other values (age, BMI, lipid status, HbA1c, GR). Although sclerostin is known as an important factor in bone destruction and might be considered as a biomarker or potential therapeutic target, there is no significant difference in sclerostin concentration between the groups and clinical parameters, as well as no correlation with either Wnt inhibitor and BMI, lipid status, or HbA1c levels. One might argue that this could be due to certain limitations of our study. Therefore, future studies with a larger sample size and strictly excluding patients with other systemic conditions are required for further confirmation and will be interesting to perform.

Author Contributions: Conceptualization, M.S.; methodology, M.S., L.K.R., A.M. and G.Y.W.; formal analysis, K.K.; investigation, K.D., A.P., L.K.R., A.M., T.K., D.I., K.B., H.R., R.S. (Renata Sikora) and M.W.; resources, M.S., R.S. (Robert Smolic) and A.V.; writing—original draft preparation, K.D., A.P., L.K.R., A.M., T.K., D.I., K.B., H.R., R.S. (Renata Sikora) and M.D.; writing—review and editing, K.D., A.P., L.K.R., A.M., T.K., D.I., K.B., H.R., R.S. (Renata Sikora), M.D. and M.W.; visualization, K.K., K.D. and R.S. (Renata Sikora); supervision, M.S., G.Y.W., A.V., M.W. and R.S. (Robert Smolic); project administration, A.P., L.K.R., R.S. (Robert Smolic), M.S., A.V. and G.Y.W.; funding acquisition, M.S. All authors have read and agreed to the published version of the manuscript.

Funding: This research was funded by the grant from the Croatian Ministry of Science and Education dedicated to multi-year institutional funding of scientific activity at the Josip Juraj Strossmayer University of Osijek, Faculty of Dental Medicine and Health, Osijek, Croatia—grant number IP7-FDMZ-2020 (to M.S.).

Institutional Review Board Statement: This study was conducted in accordance with the Declaration of Helsinki, and approved by the Ethical Committee for Research at the Public Health Scientific Institution Medical Center Dr. Mustafa Sehovic Tuzla, on 19 May 2022 (Ethical Approval Code: 16-01-2378-2/22).

Informed Consent Statement: Informed consent was obtained from all subjects involved in the study. Written informed consent has been obtained from the patient(s) to publish this paper.

Data Availability Statement: Informed consent was obtained from all subjects involved in the study.

Conflicts of Interest: The authors declare no conflicts of interest.

References

1. Lang, N.P.; Bartold, P.M. Periodontal health. *J. Clin. Periodontol.* **2018**, *45* (Suppl. S20), S9–S16. [CrossRef] [PubMed]
2. Kozak, M.; Pawlik, A. The Role of the Oral Microbiome in the Development of Diseases. *Int. J. Mol. Sci.* **2023**, *24*, 5231. [CrossRef] [PubMed]
3. Abdulkareem, A.A.; Al-Taweel, F.B.; Al-Sharqi, A.J.B.; Gul, S.S.; Sha, A.; Chapple, I.L.C. Current concepts in the pathogenesis of periodontitis: From symbiosis to dysbiosis. *J. Oral Microbiol.* **2023**, *15*, 2197779. [CrossRef] [PubMed]
4. Tonetti, M.S.; Greenwell, H.; Kornman, K.S. Staging and grading of periodontitis: Framework and proposal of a new classification and case definition. *J. Periodontol.* **2018**, *89* (Suppl. S1), S159–S172. [CrossRef] [PubMed]
5. Houschyar, K.S.; Tapking, C.; Borrelli, M.R.; Popp, D.; Duscher, D.; Maan, Z.N.; Chelliah, M.P.; Li, J.; Harati, K.; Wallner, C.; et al. Wnt Pathway in Bone Repair and Regeneration—What Do We Know So Far. *Front. Cell Dev. Biol.* **2018**, *6*, 170. [CrossRef] [PubMed]
6. Dincel, A.S.; Jørgensen, N.R.; IOF-IFCC Joint Committee on Bone Metabolism (C-BM). New Emerging Biomarkers for Bone Disease: Sclerostin and Dickkopf-1 (DKK1). *Calcif. Tissue Int.* **2023**, *112*, 243–257. [CrossRef] [PubMed]
7. Li, C.; Huang, Q.; Yang, R.; Guo, X.; Dai, Y.; Zeng, J.; Zeng, Y.; Tao, L.; Li, X.; Zhou, H.; et al. Targeted next generation sequencing of nine osteoporosis-related genes in the Wnt signaling pathway among Chinese postmenopausal women. *Endocrine* **2020**, *68*, 669–678. [CrossRef] [PubMed]
8. Pinzone, J.J.; Hall, B.M.; Thudi, N.K.; Vonau, M.; Qiang, Y.W.; Rosol, T.J.; Shaughnessy, J.D., Jr. The role of Dickkopf-1 in bone development, homeostasis, and disease. *Blood* **2009**, *113*, 517–525. [CrossRef]
9. Ozden, F.O.; Demir, E.; Lutfioglu, M.; Acarel, E.E.; Bilgici, B.; Atmaca, A. Effects of periodontal and bisphosphonate treatment on the gingival crevicular levels of sclerostin and dickkopf-1 in postmenopausal osteoporosis with and without periodontitis. *J. Periodontal Res.* **2022**, *57*, 849–858. [CrossRef]
10. Napimoga, M.H.; Nametala, C.; da Silva, F.L.; Miranda, T.S.; Bossonaro, J.P.; Demasi, A.P.D.; Duarte, P.M. Involvement of the Wnt-β-catenin signalling antagonists, sclerostin and dickkopf-related protein 1, in chronic periodontitis. *J. Clin. Periodontol.* **2014**, *41*, 550–557. [CrossRef]
11. Duan, P.; Bonewald, L.F. The role of the wnt/β-catenin signaling pathway in formation and maintenance of bone and teeth. *Int. J. Biochem. Cell Biol.* **2016**, *77 Pt A*, 23–29. [CrossRef]
12. Bao, J.; Yang, Y.; Xia, M.; Sun, W.; Chen, L. Wnt signaling: An attractive target for periodontitis treatment. *Biomed. Pharmacother.* **2021**, *133*, 110935. [CrossRef]
13. Zhang, R.; Yang, G.; Wu, X.; Xie, J.; Yang, X.; Li, T. Disruption of Wnt/β-catenin signaling in odontoblasts and cementoblasts arrests tooth root development in postnatal mouse teeth. *Int. J. Biol. Sci.* **2013**, *9*, 228–236. [CrossRef]
14. Samiei, M.; Janjić, K.; Cvikl, B.; Moritz, A.; Agis, H. The role of sclerostin and dickkopf-1 in oral tissues—A review from the perspective of the dental disciplines. *F1000Research* **2019**, *8*, 128. [CrossRef]
15. Liu, J.; Ren, X.; Zhang, M.; Lei, Y.; Chen, Y.; He, H. Roles of Wnt3a and Dkk1 in experimental periodontitis. *J. Dent. Sci.* **2017**, *12*, 220–225. [CrossRef]

16. Shen, Y.S.; Chen, X.J.; Wuri, S.N.; Yang, F.; Pang, F.X.; Xu, L.L.; He, W.; Wei, Q.S. Polydatin improves osteogenic differentiation of human bone mesenchymal stem cells by stimulating TAZ expression via BMP2-Wnt/β-catenin signaling pathway. *Stem Cell Res. Ther.* **2020**, *11*, 204. [CrossRef]
17. Tokavanich, N.; Wein, M.N.; English, J.D.; Ono, N.; Ono, W. The Role of Wnt Signaling in Postnatal Tooth Root Development. *Front. Dent. Med.* **2021**, *2*, 769134. [CrossRef] [PubMed]
18. Bojanić, K.; Bilić Ćurčić, I.; Kuna, L.; Kizivat, T.; Smolic, R.; Raguž Lučić, N.; Kralik, K.; Šerić, V.; Ivanac, G.; Tucak-Zorić, S.; et al. Association of Wnt Inhibitors, Bone Mineral Density and Lifestyle Parameters in Women with Breast Cancer Treated with Anastrozole Therapy. *J. Clin. Med.* **2018**, *7*, 287. [CrossRef] [PubMed]
19. Rezaei Esfahrood, Z.; Yadegari, Z.; Veysari, S.K.; Kadkhodazadeh, M. Gingival crevicular fluid levels of sclerostin in chronic periodontitis and healthy subjects. *J. Korean Assoc. Oral Maxillofac. Surg.* **2018**, *44*, 289–292. [CrossRef]
20. Ashifa, N.; Viswanathan, K.; Srinivasan, S.; Kumar, S.; Sundaram, R.; Pavithran, V.K. Assessment of sclerostin levels in the gingival crevicular fluid of patients with periodontitis: A clinico-biochemical crosssectional study. *J. Adv. Periodontol. Implant. Dent.* **2023**, *15*, 3–9. [CrossRef] [PubMed]
21. Shao, Y.H.; Song, Y.; Feng, Q.L.; Deng, Y.; Tang, T. Assessing the Impact of Stem Cell-based Therapy on Periodontal Health: A Meta-analysis of Clinical Studies. *Curr. Stem Cell Res. Ther.* **2024**. [CrossRef] [PubMed]
22. Tahamtan, S.; Shirban, F.; Bagherniya, M.; Johnston, T.P.; Sahebkar, A. The effects of statins on dental and oral health: A review of preclinical and clinical studies. *J. Transl. Med.* **2020**, *18*, 155. [CrossRef]
23. de Carvalho, R.D.P.; Casarin, R.C.V.; de Lima, P.O.; Cogo-Müller, K. Statins with potential to control periodontitis: From biological mechanisms to clinical studies. *J. Oral Biosci.* **2021**, *63*, 232–244. [CrossRef]
24. Cecoro, G.; Piccirillo, A.; Martuscelli, G.; Del Fabbro, M.; Annunziata, M.; Guida, L. Efficacy of locally delivered statins as an adjunct to scaling and root planning in the treatment of periodontitis: A systematic review and meta-analysis. *Eur. Rev. Med. Pharmacol. Sci.* **2021**, *25*, 5737–5754. [PubMed]
25. Lin, S.M.; Wang, J.H.; Liang, C.C.; Huang, H.K. Statin Use Is Associated With Decreased Osteoporosis and Fracture Risks in Stroke Patients. *J. Clin. Endocrinol. Metab.* **2018**, *103*, 3439–3448. [CrossRef] [PubMed]
26. Choudhary, A.; Rawat, U.; Kumar, P.; Mittal, P. Pleotropic effects of statins: The dilemma of wider utilization of statin. *Egypt. Heart J.* **2023**, *75*, 1. [CrossRef]
27. Niedzielski, M.; Broncel, M.; Gorzelak-Pabiś, P.; Woźniak, E. New possible pharmacological targets for statins and ezetimibe. *Biomed. Pharmacother.* **2020**, *129*, 110388. [CrossRef] [PubMed]
28. Staff, P.O. Correction: Relationship of Dickkopf1 (DKK1) with Cardiovascular Disease and Bone Metabolism in Caucasian Type 2 Diabetes Mellitus. *PLoS ONE.* **2015**, *10*, e0117687.
29. Afrin, S.; Ali, M.; El Sabeh, M.; Yang, Q.; Al-Hendy, A.; Borahay, M.A. Simvastatin inhibits stem cell proliferation in human leiomyoma via TGF-β3 and Wnt/β-Catenin pathways. *J. Cell. Mol. Med.* **2022**, *26*, 1684–1698. [CrossRef]
30. Göbel, A.; Browne, A.J.; Thiele, S.; Rauner, M.; Hofbauer, L.C.; Rachner, T.D. Potentiated suppression of Dickkopf-1 in breast cancer by combined administration of the mevalonate pathway inhibitors zoledronic acid and statins. *Breast Cancer Res. Treat.* **2015**, *154*, 623–631. [CrossRef]
31. Xiao, Y.; Liu, Q.; Peng, N.; Li, Y.; Qiu, D.; Yang, T.; Kang, R.; Usmani, A.; Amadasu, E.; Borlongan, C.V.; et al. Lovastatin Inhibits RhoA to Suppress Canonical Wnt/β-Catenin Signaling and Alternative Wnt-YAP/TAZ Signaling in Colon Cancer. *Cell Transplant.* **2022**, *31*, 9636897221075749. [CrossRef]
32. Tong, X.K.; Royea, J.; Hamel, E. Simvastatin rescues memory and granule cell maturation through the Wnt/β-catenin signaling pathway in a mouse model of Alzheimer's disease. *Cell Death Dis.* **2022**, *13*, 325. [CrossRef]
33. Wang, B.X.; Li, K.P.; Yu, T.; Feng, H.Y. Rosuvastatin promotes osteogenic differentiation of mesenchymal stem cells in the rat model of osteoporosis by the Wnt/β-catenin signal. *Eur. Rev. Med. Pharmacol. Sci.* **2019**, *23*, 10161–10168.
34. Zhou, H.; Xie, Y.; Baloch, Z.; Shi, Q.; Huo, Q.; Ma, T. The effect of atorvastatin, 3-hydroxy-3-methylglutaryl coenzyme A reductase inhibitor (HMG-CoA), on the prevention of osteoporosis in ovariectomized rabbits. *J. Bone Miner. Metab.* **2017**, *35*, 245–254. [CrossRef] [PubMed]
35. Zhang, M.; Bian, Y.Q.; Tao, H.M.; Yang, X.F.; Mu, W.D. Simvastatin induces osteogenic differentiation of MSCs via Wnt/β-catenin pathway to promote fracture healing. *Eur. Rev. Med. Pharmacol. Sci.* **2018**, *22*, 2896–2905. [PubMed]
36. Donders, H.C.M.; Veth, E.O.; van't Hof, A.W.J.; de Lange, J.; Loos, B.G. The association between periodontitis and cardiovascular risks in asymptomatic healthy patients. *Int. J. Cardiol. Cardiovasc. Risk Prev.* **2021**, *11*, 200110. [CrossRef]
37. Sousa, L.H.; Linhares, E.V.; Alexandre, J.T.; Lisboa, M.R.; Furlaneto, F.; Freitas, R.; Ribeiro, I.; Val, D.; Marques, M.; Chaves, H.V.; et al. Effects of Atorvastatin on Periodontitis of Rats Subjected to Glucocorticoid-Induced Osteoporosis. *J. Periodontol.* **2016**, *87*, 1206–1216. [CrossRef] [PubMed]
38. Zhang, H.; Zhang, Y.; Chen, X.; Li, J.; Zhang, Z.; Yu, H. Effects of statins on cytokines levels in gingival crevicular fluid and saliva and on clinical periodontal parameters of middle-aged and elderly patients with type 2 diabetes mellitus. *PLoS ONE* **2021**, *16*, e0244806. [CrossRef]
39. Henssler, L.; Kerschbaum, M.; Mukashevich, M.Z.; Rupp, M.; Alt, V. Molecular enhancement of fracture healing—Is there a role for Bone Morphogenetic Protein-2, parathyroid hormone, statins, or sclerostin-antibodies? *Injury* **2021**, *52* (Suppl. S2), S49–S57. [CrossRef] [PubMed]

40. Pontremoli, M.; Brioschi, M.; Baetta, R.; Ghilardi, S.; Banfi, C. Identification of DKK-1 as a novel mediator of statin effects in human endothelial cells. *Sci. Rep.* **2018**, *8*, 16671. [CrossRef]
41. Fatima, T.; Khurshid, Z.; Rehman, A.; Imran, E.; Srivastava, K.C.; Shrivastava, D. Gingival Crevicular Fluid (GCF): A Diagnostic Tool for the Detection of Periodontal Health and Diseases. *Molecules* **2021**, *26*, 1208.
42. Pei, J.; Li, F.; Xie, Y.; Liu, J.; Yu, T.; Feng, X. Microbial and metabolomic analysis of gingival crevicular fluid in general chronic periodontitis patients: Lessons for a predictive, preventive, and personalized medical approach. *EPMA J.* **2020**, *11*, 197–215. [CrossRef]
43. Gehrig, J.S.; Sroda, R.; Saccuzzo, D. *Fundamentals of Periodontal Instrumentation & Advanced Root Instrumentation*, 8th ed.; Wolters Kluwer: Philadelphia, PA, USA, 2017.
44. Caton, J.G.; Armitage, G.; Berglundh, T.; Chapple, I.L.; Jepsen, S.; Kornman, K.S.; Mealey, B.L.; Papapanou, P.N.; Sanz, M.; Tonetti, M.S. A new classification scheme for periodontal and peri-implant diseases and conditions—Introduction and key changes from the 1999 classification. *J. Clin. Periodontol.* **2018**, *45* (Suppl. S20), S1–S8. [CrossRef]
45. Guentsch, A.; Kramesberger, M.; Sroka, A.; Pfister, W.; Potempa, J.; Eick, S. Comparison of gingival crevicular fluid sampling methods in patients with severe chronic periodontitis. *J. Periodontol.* **2011**, *82*, 1051–1060. [CrossRef] [PubMed]
46. Günday, S.; Topcu, A.O.; Ercan, E.; Yamalik, N. Analysis of daytime variations in gingival crevicular fluid: A circadian periodicity? *J. Periodontol.* **2014**, *85*, e47–e56. [CrossRef] [PubMed]
47. Romero-Sánchez, C.; Giraldo, S.; Heredia-P, A.M.; De Avila, J.; Chila-Moreno, L.; Londoño, J.; Valle-Oñate, R.; Bello-Gualtero, J.M.; Bautista-Molano, W. Association of Serum and Crevicular Fluid Dickkopf-1 Levels with Disease Activity and Periodontitis in Patients with Early Rheumatoid Arthritis. *Curr. Rheumatol. Rev.* **2022**, *18*, 124–135. [CrossRef]
48. Ramenzoni, L.L.; Lehner, M.P.; Kaufmann, M.E.; Wiedemeier, D.; Attin, T.; Schmidlin, P.R. Oral Diagnostic Methods for the Detection of Periodontal Disease. *Diagnostics* **2021**, *11*, 571. [CrossRef] [PubMed]
49. Petit, C.; Batool, F.; Bugueno, I.M.; Schwinté, P.; Benkirane-Jessel, N.; Huck, O. Contribution of Statins towards Periodontal Treatment: A Review. *Mediat. Inflamm.* **2019**, *2019*, 6367402. [CrossRef]
50. Rachner, T.D.; Göbel, A.; Thiele, S.; Rauner, M.; Benad-Mehner, P.; Hadji, P.; Bauer, T.; Muders, M.H.; Baretton, G.B.; Jakob, F.; et al. Dickkopf-1 is regulated by the mevalonate pathway in breast cancer. *Breast Cancer Res.* **2014**, *16*, R20. [CrossRef]
51. Crandall, J.P.; Mather, K.; Rajpathak, S.N.; Goldberg, R.B.; Watson, K.; Foo, S.; Ratner, R.; Barrett-Connor, E.; Temprosa, M. Statin use and risk of developing diabetes: Results from the Diabetes Prevention Program. *BMJ Open Diabetes Res. Care* **2017**, *5*, e000438. [CrossRef]
52. Corrao, G.; Ibrahim, B.; Nicotra, F.; Soranna, D.; Merlino, L.; Catapano, A.L.; Tragni, E.; Casula, M.; Grassi, G.; Mancia, G. Statins and the risk of diabetes: Evidence from a large population-based cohort study. *Diabetes Care* **2014**, *37*, 2225–2232. [CrossRef] [PubMed]
53. Robinson, J.G. Statins and diabetes risk: How real is it and what are the mechanisms? *Curr. Opin. Lipidol.* **2015**, *26*, 228–235. [CrossRef] [PubMed]
54. Chirapongsathorn, S.; Sukeepaisarnjaroen, W.; Treeprasertsuk, S.; Chaiteerakij, R.; Surawongsin, P.; Hongthanakorn, C.; Siramolpiwat, S.; Chamroonkul, N.; Bunchorntavakul, C.; Chotiyaputta, W.; et al. Characteristics of Drug-induced Liver Injury in Chronic Liver Disease: Results from the Thai Association for the Study of the Liver (THASL) DILI Registry. *J. Clin. Transl. Hepatol.* **2023**, *11*, 88–96.
55. Zhou, M.; Graves, D.T. Impact of the host response and osteoblast lineage cells on periodontal disease. *Front. Immunol.* **2022**, *13*, 998244. [CrossRef]
56. Battancs, E.; Gheorghita, D.; Nyiraty, S.; Lengyel, C.; Eördegh, G.; Baráth, Z.; Várkonyi, T.; Antal, M. Periodontal Disease in Diabetes Mellitus: A Case-Control Study in Smokers and Non-Smokers. *Diabetes Ther.* **2020**, *11*, 2715–2728. [CrossRef]
57. Di Spirito, F.; Schiavo, L.; Pilone, V.; Lanza, A.; Sbordone, L.; D'Ambrosio, F. Periodontal and Peri-Implant Diseases and Systemically Administered Statins: A Systematic Review. *Dent. J.* **2021**, *9*, 100. [CrossRef] [PubMed]

Disclaimer/Publisher's Note: The statements, opinions and data contained in all publications are solely those of the individual author(s) and contributor(s) and not of MDPI and/or the editor(s). MDPI and/or the editor(s) disclaim responsibility for any injury to people or property resulting from any ideas, methods, instructions or products referred to in the content.
