# Supplementary material for: Molecular Regulators for the Wnt/β-Catenin Signalling Pathway for Bone Remodelling in Periodontitis: A Scoping Review
Source: Int J Mol Sci. 2026 Oct 14;27(20):9117. doi: 10.3390/ijms27209117 (PMC13705578; doi:10.3390/ijms27209117)
Supplement: Supplementary file 1 [file ijms-27-09117-s001.zip › Supplementary Data/Papers/in vitro/LINC00673 binds with leptin to regulate osteogenic function in periodontal ligament stem cells.pdf]

Yang et al. Journal of Orthopaedic Surgery and Research (2025) 20:165
https://doi.org/10.1186/s13018-025-05562-0

Journal of Orthopaedic Surgery and Research

RESEARCH Open Access

# LINC00673 binds with leptin to regulate osteogenic function in periodontal ligament stem cells

Yang Yang[1,2], Peng Yang[1], Yuxing Zhang[1], Zhaoyan Wu[1], Xuxia Wang[3] and Jun Zhang[1*]


## Abstract

**Background** The aim of this study is to investigate whether LINC00673 and leptin are associated with osteogenesis of periodontal ligament stem cells in the microenvironment of advanced glycation end products.

**Methods** LINC00673 expression was detected in PDLSCs by qRT-PCR performed during osteogenic differentiation. By using alkaline phosphatase and Alizarin red S staining, we were able to confirm the role of LINC00673 in regulating osteogenesis in PDLSCs. Assays were performed on nude mice to test bone regeneration in the dorsal region to investigate MiR-188-3p's binding to LINC00673 and leptin (LEP). Western blot was used to detect osteoblast markers (COL1, ALP and RUNX2).

**Results** As predicted, miR-188-3p interacts with LINC00673 directly, and miR-188-3p overexpression increases osteogenic differentiation. However, overexpression of LINC00673 reversed this effect, suggesting that LINC00673 functions as a competing endogenous RNA for miR-188-3p. A regulatory network formed by LINC00673 and miR-188-3p regulates the expression of LEP, a gene that inhibits the canonical Wnt pathway, reducing bone formation in PDLSCs.

**Conclusions** PDLSCs differentiate osteogenically as a result of a regulatory network between lncRNA and miRNA (microRNA), which may serve as a therapeutic target for diabetes-related periodontitis.


**Keywords** Osteogenesis, Advanced glycosylation end products, Long non-coding RNA, LINC00673, Leptin

*Correspondence:
Jun Zhang
zhangj@sdu.edu.cn
[1]Department of Orthodontics, School and Hospital of Stomatology, Cheeloo College of Medicine, Shandong University &Shandong Key Laboratory of Oral Tissue Regeneration & Shandong Engineering Research Center of Dental Materials and Oral Tissue Regeneration & Shandong Provincial Clinical Research Center for Oral Diseases, No.44-1 Wenhua Road West, Shandong 250012 Jinan, China
[2]Department of Stomatology, Baotou Central Hospital, No.61 Huancheng Road, Inner Mongolia 014040 Baotou, China
[3]Department of Oral and Maxillofacial Surgery, School and Hospital of Stomatology, Cheeloo College of Medicine, Shandong University &Shandong Key Laboratory of Oral Tissue Regeneration & Shandong Engineering Research Center of Dental Materials and Oral Tissue Regeneration & Shandong Provincial Clinical Research Center for Oral Diseases, No.44-1 Wenhua Road West, Shandong 250012 Jinan, China

## Introduction

Periodontitis is a prevalent oral disease that results in the deterioration of the tissues that support the teeth, such as the periodontal ligament, cementum, and alveolar bone [1, 2]. Unlike other tissues, periodontal tissue cannot regenerate even after inflammation has been alleviated. This is partially due to the loss of differentiation capacity of periodontal ligament stem cells (PDLSCs) within the inflammatory microenvironment [3]. PDLSCs originate from the periodontal ligament and are a type of mesenchymal stem cells (MSCs). These cells possess the ability to differentiate into multiple cell types and have shown great potential for regenerating supporting tissues in

© The Author(s) 2025. **Open Access** This article is licensed under a Creative Commons Attribution-NonCommercial-NoDerivatives 4.0 International License, which permits any non-commercial use, sharing, distribution and reproduction in any medium or format, as long as you give appropriate credit to the original author(s) and the source, provide a link to the Creative Commons licence, and indicate if you modified the licensed material. You do not have permission under this licence to share adapted material derived from this article or parts of it. The images or other third party material in this article are included in the article's Creative Commons licence, unless indicated otherwise in a credit line to the material. If material is not included in the article's Creative Commons licence and your intended use is not permitted by statutory regulation or exceeds the permitted use, you will need to obtain permission directly from the copyright holder. To view a copy of this licence, visit http://creativecommons.org/licenses/by-nc-nd/4.0/.

recent studies. Studies found that PDLSCs with a neural crest origin are highly appropriate for neural lineage induction [4, 5]. The ultimate objective of periodontal treatment is the effective reconstruction of the supporting structures of the periodontium. Further investigation into the regulation of PDLSCs during osteogenic differentiation could be promising in enhancing the osteogenic potential of PDLSCs and regenerating periodontal tissues.

One characteristic of diabetes mellitus is the accumulation of AGEs in the periodontal tissues as a person ages. In conditions such as chronic hyperglycemia, this accumulation is even more intensified. This can further deteriorate the physical and mechanical properties of the extracellular matrix, such as collagen. The accumulation of AGEs is also linked to an elevation in oxidative damage and a rise in the proinflammatory environment. This phenomenon has the potential to compromise the osseous support of the dental structure, leading to a delay in bone formation and suboptimal quality of the newly generated bone tissue. AGEs is a common link between diabetes and periodontitis, contributing to the destruction of periodontal tissue [6].

LncRNAs, which are a class of RNAs with lengths greater than 200 bases, have recently gained widespread attention due to their critical roles in regulating transcripts during biological and pathological processes. Previous bioinformatics analyses have revealed abnormal expression of several lncRNAs and their associated competing endogenous RNAs in periodontitis. For instance, lncRNAs such as TUG1, H19, MEG3, TWIST1, and lncPCAT1 have been shown to promote osteogenesis in various types of stem cells [7–10]. On the other hand, inhibiting lncRNAs ANCR and DANCR may enhance osteogenic differentiation of PDLSCs [11, 12]. According to a previous study [13, 14], LncRNAs play a significant role in the osteogenic differentiation of PDLSCs. We utilized Microarray Analysis to identify LINC00673 as the focus of our study. Subsequently, we explored how LINC00673 inhibits osteogenic differentiation of PDLSCs through the WNT/β-catenin signaling pathway.

Researchers have conducted studies on the impact of LINC00673, a recently discovered lncRNA, on various types of cancer, such as non-small cell lung cancer [15, 16], tongue cancer [17], gastric cancer [18], and pancreatic cancer [19], among others. The function of LINC00673 in PDLSCs under AGEs microenvironment has never been elucidated. In this study, we investigated the mechanism by which LINC00673 regulates osteogenesis in PDLSCs under the influence of AGEs microenvironment through the LINC00673/miR-188-3p/LEP/WNT/β-catenin signaling pathway. These findings are expected to enhance our understanding of how the microenvironment of AGEs affects osteogenesis through LINC00673 in PDLSCs. This knowledge will contribute to the development of more effective treatment strategies for periodontitis associated with diabetes.

## Materials and methods

### Cell culture

The 293T cells derived from human embryonic kidney were generously provided by Jing Lan's research group at The School and Hospital of Stomatology, Cheeloo College of Medicine, Shandong University. PDLSCs were isolated and cultured following established protocols as per published literature. A cohort of healthy individuals, with a mean age of 18 years, underwent extraction of 27 premolars for either orthodontic treatment or third molar management. To prepare the periodontal tissues, sound premolars or third molar roots were meticulously scraped, sectioned, and subjected to enzymatic digestion using equal amounts of type I collagenase and dispase at 37 °C for a duration of 30 min. Prior to their participation in the study, all patients provided informed consent, and the study protocol was granted approval by the Human Ethics Committee of Shandong University (NO.20220402). This ensured that the study adhered to ethical guidelines and regulations. The 293T cells were cultured at 37 °C with 5% CO2 in high-glucose dulbecco's modified eagle medium (DMEM) (HyClone) supplemented with 10% fetal bovine serum (FBS). Similarly, the PDLSCs were habitually cultured in Alpha Modification of Minimum Essential Medium (α-MEM) (HyClone). PDLSCs were collected and utilized for subsequent investigations following five passages. Osteogenic induction medium contained α-MEM (Corning, USA) supplemented with 10% FBS (Gibco, USA), 10 nM dexamethasone (Dex) (Sigma, USA), 10 mM β-glycerophosphate (Sigma, USA), and 50 mg/L ascorbic acid (Sigma, USA). Cells were incubated with or without AGEs (100 μg/mL, #BS1158P, Bioss Chemical, Beijing, China) and osteogenic induction medium for 24 h.

### Microarray analysis

In this study, a total of six samples were utilized for the purpose of total RNA extraction. These samples consisted of three osteogenic differentiation induced PDLSCs and three AGEs - osteogenic differentiation induced PDLSCs, each obtained from three individuals. Tissue samples were prepared and microarray hybridization was conducted in accordance with the standard protocols provided by the manufacturer. The raw data and array images were obtained using Affymetrix (USA) software. The Affymetrix GeneChip2 platform was utilized for quantile normalization and subsequent data processing. The microarray analysis was conducted by QiMing Bio-tech, a company based in Shanghai, China. Prominently expressed long non-coding RNAs (lncRNAs) were

identified based on statistical significance ($p < 0.05$) and a fold change (FC) of at least 1.5. Gene set enrichment analysis (GSEA) was conducted using GSEA software version 3.0 and visualized through ClusterProfiler. The Gene Ontology (GO) classification, which encompasses GO-BP (biological process), GO-MF (molecular function), and GO-CC (cellular component), was visualized using the DAVID V6.8 webserver (http://david.ncifcrf.gov).

### Cell transfection

Cells were placed into 6-well plates prior to transfection. Upon achieving 80% confluence, the cells were transfected with 2 μg plasmids or 100 nM miRNA inhibitor or small interfering RNA (siRNAs) using Micropoly transfecter Cell Regent (Micropoly, China) in accordance with the manufacturer's protocol. The plasmids and lentiviruses utilized in this study were designed and constructed by GeneChem. The PDLSCs were cultured in six-well plates at a concentration of $2 \times 10^5$ cells per well. Upon reaching 30–40% confluence, the cells underwent transfection with lentiviruses in the presence of polybrene. Oligonucleotide and plasmid transfection were performed using the Micropoly transfecter Cell Regent (Micropoly, China) in accordance with the manufacturer's instructions. Following a 48-hour transfection period, the cells were harvested and subsequently utilized for further analyses.

### Quantitative real-time PCR (qRT-PCR)

Total RNA was extracted from PDLSCs using TRIzol reagent (Accurate Biotechnology) and subjected to reverse transcription. The M-MLV Reverse Transcriptase Kit (Accurate Biotechnology) was used for lncRNA/mRNA reverse transcription, while the miRCURY LNA RT Kit (Vazyme Biotechnology) was used for miRNA reverse transcription. Real-time PCR was conducted on a Roche LightCycler 480 PCR System (Roche, Rotkreuz, Switzerland) using a standard SYBR Green PCR kit (Accurate Biotechnology) in accordance with the manufacturer's instructions. In the case of long non-coding RNAs (lncRNAs) and messenger RNAs (mRNAs), glyceraldehyde-3-phosphate dehydrogenase (GAPDH) was employed as a reference gene. Conversely, for miR-188-3p, U6 small nuclear RNA was utilized as a reference. The 2-ΔΔCT expression method was employed to determine the relative quantitation of gene expression levels. The experimental procedure was replicated thrice. The PCR primers were synthesized by Boshang Biotechnology and their corresponding sequences can be found in Appendix 1: Appendix Table 1.

### Western blot

The protein concentration of the lysate was quantified using the bicinchoninic acid (BCA) kit (Beyotime, Shanghai, China) following the manufacturer's instructions. Prior to quantification, the cells underwent lysis through the utilization of a BCA protein assay kit (Solarbio). Subsequently, 20 μg of total protein was subjected to separation via Sodium Dodecyl Sulfate Polyacrylamide Gel Electrophoresis (SDS-PAGE) and subsequently transferred onto (polyvinylidene fluoride) PVDF membranes (Millipore) for further analysis. A 5% BSA (Sigma-Aldrich) was utilized as a blocking agent for 1 h at room temperature. The membranes were then incubated with primary antibodies overnight at 4 °C, with GAPDH serving as the internal reference. Following the labeling of proteins with Horseradish Peroxidase (HRP), the Beyotime ECL Star Chemiluminescence Substrate Kit (BeyoECL Star Kit) (Beyotime) was utilized to develop the proteins. Subsequently, the proteins were washed thrice with Tris-Buffered Saline with Tween (TBST) and filmed. Appendix Table 2 provides a comprehensive list of primary antibodies employed in the study. Proteins were visualized using Enhanced Chemiluminescence Plus reagents (ECL-plus reagents) (Millipore, Billerica, MA, USA), and the density of the bands was quantified using ImageJ software (Version 1.48u, Bethesda, USA). Experiments were done in triplicate.

### Mineralization assay

The PDLSCs were cultured in a 24-well plate for 7 and 21 days, respectively, with osteogenic induction medium at a density of $1 \times 10^5$ cells per well. The cells were then subjected to Alizarin Red S (ARS) and Alkaline Phosphatase (ALP) staining using the ARS and ALP Staining Kits from Solarbio (Beijing, China) and Nanjing, respectively, in accordance with the manufacturer's instructions.

### In vivo transplantation

Eight-week-old BALB/C male mice were allocated randomly into four groups, each consisting of five mice. The mice were procured from Ziyuan Laboratory Animal Technology, located in Hangzhou, China. For transplantation, a 20 mg hydroxyl apatite-tricalcium phosphate scaffold particles of 1.0 mm in size (HA-TCP; Sichuan Baiamon Bioactive Materials Co. LTD) was loaded with $5 \times 10^6$ cells and subcutaneously implanted into the dorsal region of BALB/C mice for 4 weeks. The administration of anesthesia during all surgical procedures was achieved through inhalation of isoflurane. The animal procedures conducted in this study were approved by the Animal Care Committee of Shandong University. The mice were euthanized via cervical dislocation while under general anesthesia four weeks after the experiment. The xenografts were extracted and subjected to decalcification

using a 10% ethylenediaminetetraacetic acid solution (pH 6.0) for a period of 7 days. The xenografts were subsequently embedded in paraffin, sectioned, and subjected to staining with hematoxylin and eosin (H&E; Beyotime Institute of Biotechnology, Shanghai, China) or Masson's Trichrome stain (Solarbio Biotechnology, Beijing, China) in accordance with the manufacturer's protocols for histological analysis.

### Luciferase reporter assay

Putative binding sequences of miR-188-3p were synthesized and subsequently cloned downstream of the luciferase gene in the pmirGLO luciferase vector, separately in both wild-type and mutant LINC00673 and LEP. The cloning process was carried out by Shuoboyun Biotechnology, located in Shandong, China. The Micropoly transfecter Cell Regent was utilized to transfect miRNA mimics (miR-NC and miR-188-3p) and a dual-luciferase plasmid (Shuoboyun Biotechnology, Shandong, China) into the cells. The luminescent activities of firefly and Renilla luciferase were assessed over a period of 48 h subsequent to transfection, utilizing a dual luciferase assay. All experimental procedures were conducted in triplicate.

### Immunofluorescence assay

PDLSCs were seeded onto a 24-well plate with a cover slip and subsequently fixed in 4% paraformaldehyde for 15 min at 90% confluency. Permeability was attained through the process of permeabilization using 0.1% Triton for a duration of 5 min, followed by blocking with 10% goat serum for 60 min. Following incubation with anti-catenin antibodies (1:250) (#8480, CST, US) at 4 °C overnight, PDLSCs were subsequently incubated with Fluorescein Isothiocyanate (FITC)-conjugated goat anti-mouse antibodies (1:100) (Sigma, USA) for 1 h at room temperature. To ensure precision and minimize errors, the experiment was performed in triplicate. Following treatment, the cells were stained with 4′, 6-diamidino-2-phenylindole (DAPI) for a duration of 5 min and subsequently observed under a Carl-Zeiss Aximot 2 (Olympus IX 71, Germany) microscope.

### Statistical analysis

The statistical analysis was conducted utilizing the Statistical Package for the Social Sciences (SPSS) version 16.0 software (SPSS Inc., Chicago, IL, USA). The data have been presented as the mean and standard deviation of a minimum of three independent experiments. The statistical analysis employed in this study involved the use of Student's t-test for multiple comparisons between two groups, and One-way Analysis of Variance (one-way ANOVA) for multiple comparisons between three or more groups. A significance level of 0.05 was used to determine statistical significance, whereby p-values less than this threshold were considered significant.

## Results

### The expression of LINC00673 underwent significant alterations during osteogenic induction and was found to be closely associated with the process of osteogenic differentiation of PDLSCs

The present study employed microarray technology to evaluate the expression patterns of lncRNAs in PDLSCs under AGEs microenvironments, with the aim of identifying lncRNAs that modulate the osteogenic capacity of PDLSCs. Figure 1A illustrates a heatmap that portrays alterations in lncRNAs. Subsequently, we employed RT-qPCR to validate the microarray findings and ascertained that NR 036488.1 (LINC00673) exhibited the most substantial alteration among the lncRNAs between the normal osteogenic induced group and the AGEs osteogenic induced group. Notably, the expression of NR 036488.1 (LINC00673) gradually increased until the 14th day of induction, as depicted in Fig. 1C.

### The inhibitory effect of LINC00673 on osteogenic differentiation of PDLSCs was observed both in vitro and in vivo

In order to investigate the involvement of LINC00673 in the process of osteogenic differentiation of PDLSCs, we generated shLINC00673 lentiviruses to induce LINC00673 knockdown (shLINC00673), as well as LINC00673 overexpressing plasmids to promote LINC00673 overexpression. The confirmation of transfection effects was carried out through the use of RT-qPCR, as depicted in Fig. 1D. In order to mitigate off-target effects, three distinct shRNA constructs were utilized to transfect LINC00673, as depicted in Fig. 1E.

The overexpression of LINC00673 resulted in a significant reduction in the mRNA levels of osteogenic genes, including Runt-related transcription factor 2 (Runx2), Alkaline phosphatase (ALP), and Collagen type I (Col1), in PDLSCs, as shown in Fig. 1F. In contrast, the deletion of LINC00673 resulted in a significant upregulation of the expression of these genes in PDLSCs, as depicted in Fig. 1K. The protein level expression of Runx2, ALP, and Col1 is regulated by LINC00673, as demonstrated in Fig. 1G and L. In order to investigate the impact of LINC00673 on the osteogenic process, various assays were conducted including ARS, ALP staining, and ALP activity assays. The results indicated that overexpression of LINC00673 led to a decrease in ALP activity and mineralized bone matrix formation in PDLSCs ( Fig. 1H and J ). Conversely, shLINC00673 increased both ALP activity and mineralized bone matrix formation, as demonstrated in Fig. 1M and O.

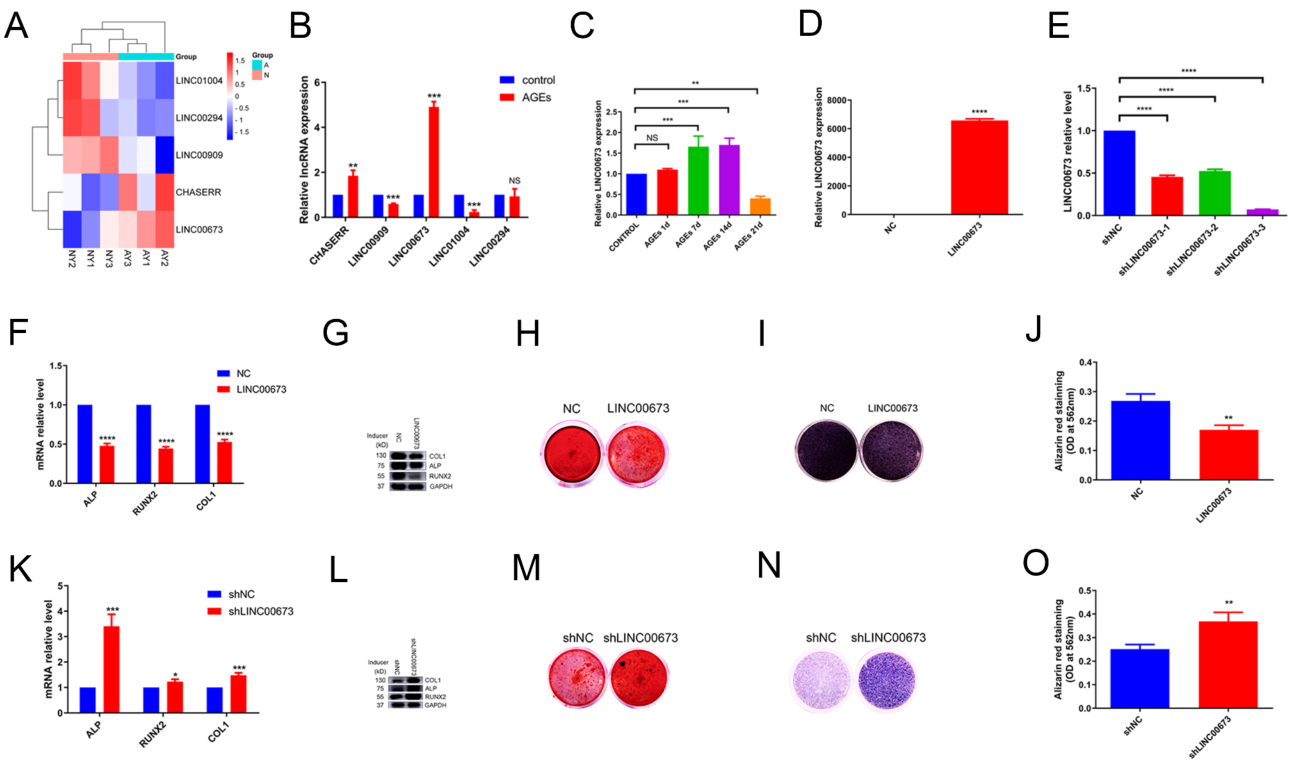


**Fig. 1** LINC00673 suppresses osteogenic differentiation in hPDLSCs. (**A**) A heatmap depicting differentially expressed long noncoding RNAs in PDLSCs treated for 7 days with osteogenic-induced medium. (**B**) qRT-PCR was used to confirm the expression levels of lncRNAs with high fold changes (fold change ≥ 1.5, $P < 0.05$) in AGEs microenvironments 7 days after osteogenic induction. (**C**) LINC00673 expression levels were measured using qRT-PCR at 0, 1, 7, and 14 days. (**D**, **E**) qRT-PCR efficiency of overexpression and interference of LINC00673 in hPDLSCs. (**F**, **G**, **K**, **L**) Following transfection with lenti-LINC00673 or sh-LINC00673 and the corresponding controls, ALP, Runx2 and Col1 expressions were assessed by qPCR and Western blot. (**H**, **I**, **J** and **M**, **N**, **O**) hPDLSCs stained with ALP and Alizarin Red after transfection with lenti-LINC00673 or sh-LINC00673, as well as controls. Data represent the mean ± S.D. (*$P < 0.05$, **$P < 0.01$, ***$P < 0.001$, ****$P < 0.0001$)

BALB/C mice were subcutaneously implanted with PDLSCs loaded with Hydroxyapatite-coated Titanium Plasma Spray (HA-TCP) for a duration of four weeks to investigate the impact of LINC00673 on osteogenesis of PDLSCs in vivo, as shown in Fig. 2E, drawn by FIGDRAW 2.0 (ID: RIIIO0dd6b). After the removal of the implants, the newly generated bone tissue underwent staining with HE and Masson's Trichrome. The study revealed that upregulation of LINC00673 reduced osteoid formation, whereas reduction boosted bone formation, as seen in Fig. 2A-D. The results of this study indicate that LINC00673 inhibits both in vitro and in vivo osteogenesis of PDLSCs.

### The transcript LINC00673 functioned as a molecular sponge for miR-188-3p

The BiBiServ tool was utilized to identify a single putative binding site of miR-188-3p to LINC00673, with the aim of investigating its potential role as a miRNA sponge that competes with mRNA for binding to miRNAs. This is illustrated in Fig. 2J. A miR-188-3P inhibitor was synthesized and its effectiveness was evaluated through qPCR in PDLSCs (Fig. 2H). Luciferase reporter constructs were devised to investigate the direct regulatory effect of LINC00673 on miR-188-3p. The activity of the LINC00673 - Wild Type (WT) reporter was significantly inhibited by MiR-188-3p in 293T cells, as demonstrated in Fig. 2K. However, no significant effect was observed on the LINC00673 - Mutant (MU) reporter. Based on the findings of this study, it can be concluded that there exists a direct regulatory relationship between LINC00673 and miR-188-3p. The expression of MiR-188-3p was observed to increase subsequent to the knockdown of LINC00673. Conversely, the levels of LINC00673 were found to increase following the transfection of a miR-188-3p inhibitor, as depicted in Fig. 2G and I.

### During osteogenesis, the regulatory network involving LINC00673/miR-188-3p targeted LEP

The selection of the target gene LEP was based on the outcomes of microarray data analysis, which identified it as the most compelling candidate. A dual luciferase reporter gene assay was conducted to investigate the direct binding of miR-188-3p to LEP. The findings of the study indicate that miR-188-3p exhibited a significant inhibitory effect on LEP-WT reporter activity

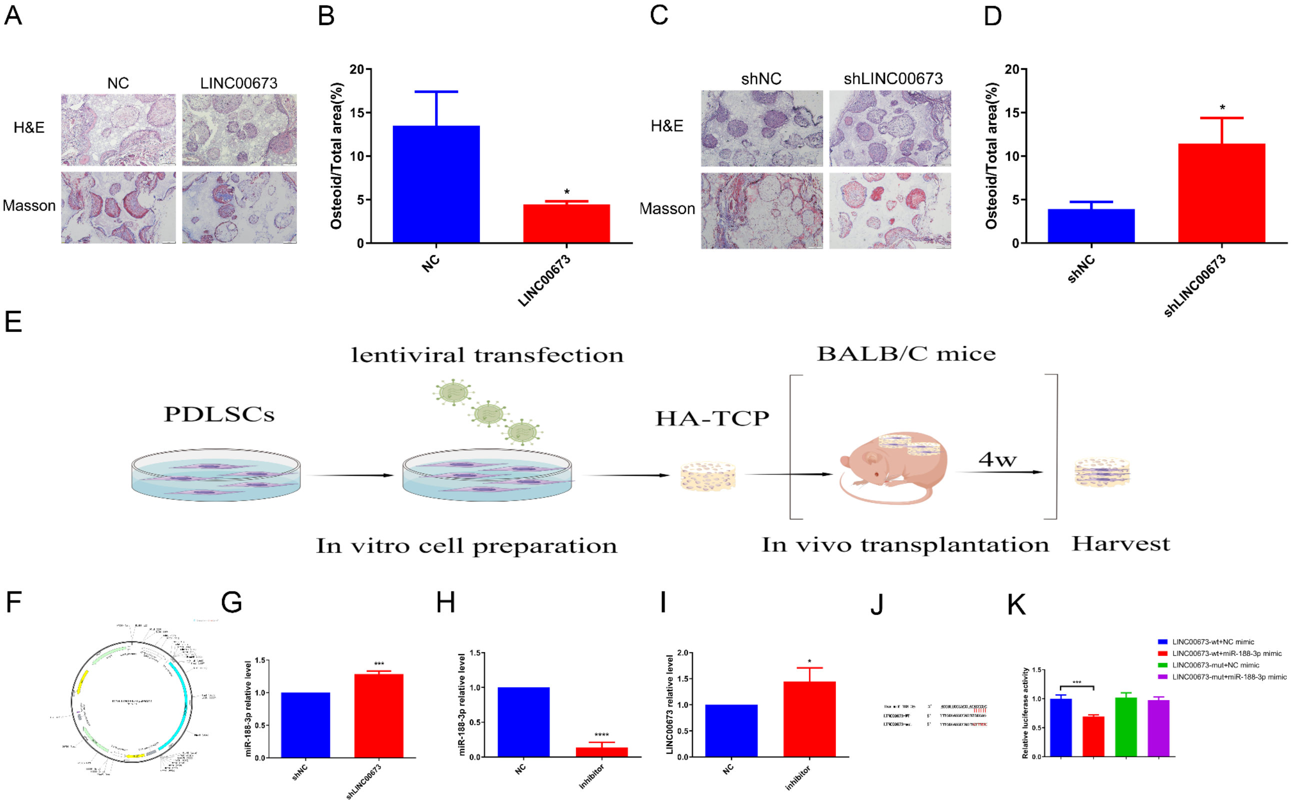


**Fig. 2** ShLINC00673 promotes osteogenesis of hPDLSCs in vivo. (**A**, **C**) The HA-TCP-transfected hPDLSCs were transplanted into the dorsal region of nude mice for four weeks after being mixed with the HA-TCP. The LIN00673 hPDLSCs were stained with H&E and Masson stain in vivo. (n = 5 per group). (**B**, **D**) A quantitative analysis of the new bone area using Image-Pro Plus 6.0. (**E**) The combinations from different groups of PDLSCs were surgically implanted into both sides of the backs of nude mice. n = 5/group. The specimens were harvested four weeks after implantation, decalcified, embedded in paraffin, and stained with H&E and Masson's solution. This figure was drawn by Figdraw. (**F**) Diagram of the plasmid vector pattern. (**G**) The expression of miR-188-3p following sh-LINC00673 transfection and the corresponding controls. (**H**) qPCR was used to determine the transfection effects of miR-188-3p inhibitor. (**I**) The expression of LINC00673 after miR-188-3p inhibitor transfection and the corresponding controls. (**J**) Predicted binding of miR-188-3p to LINC00673 3'-UTR. Wild-type (WT) and mutant (Mut) LINC00673 target sites are displayed. (**K**) Testing the interaction between miR-188-3p and LINC00673 with a dual-luciferase reporter assay. Data represent mean ± SD. (*$P < 0.05$, ***$P < 0.001$, ****$P < 0.0001$)

in 293T, while no discernible impact was observed on LEP-MU reporter activity, as illustrated in Fig. 3H and I. Based on the findings, it can be inferred that there exists a direct regulatory relationship between LEP and miR-188-3p. The results of the study indicate that the expression of LEP mRNA was observed to be elevated in PDLSCs that were transfected with LINC00673 overexpression. Conversely, a decrease in LEP mRNA expression was observed in PDLSCs that were transfected with shLINC00673, as illustrated in Fig. 3B and D. The protein expression of LEP exhibited a similar pattern, as depicted in Fig. 3C and E. The results of this study indicate that LEP operates as a downstream target of the LINC00673/miR-188-3p axis.

## The impact of MiR-188-3p and LEP on the osteogenic differentiation of PDLSCs exhibits opposing effects

The levels of LEP protein and mRNA in PDLSCs were observed to have a significant increase subsequent to treatment with a miR-188-3p inhibitor, as depicted in Fig. 3F and G. The findings suggest that in PDLSCs, LINC00673 has a positive regulatory effect on the expression of LEP, as demonstrated in Fig. 3B and C. Furthermore, an inhibitor of miR-188-3p was synthesized to ascertain its osteogenic properties. The results of QPCR, western blot analyses, and Alizarin red staining indicated that the use of miR-188-3p inhibitors resulted in the suppression of osteogenesis in PDLSCs, as demonstrated in Fig. 3J and N. The impact of LEP on the osteogenic process was subsequently evaluated. The inhibition of LEP resulted in an upregulation of osteoblast-related genes at both the protein and mRNA levels, as demonstrated in Fig. 4C and D. The decline of LEP on osteogenesis was confirmed through the use of ARS and ALP staining, as well as the ALP activity assay, as depicted in Fig. 4E and G.

3.6 The findings suggest that LEP has the potential to modulate the osteogenic differentiation of PDLSCs through the inhibition of the canonical Wnt pathway.

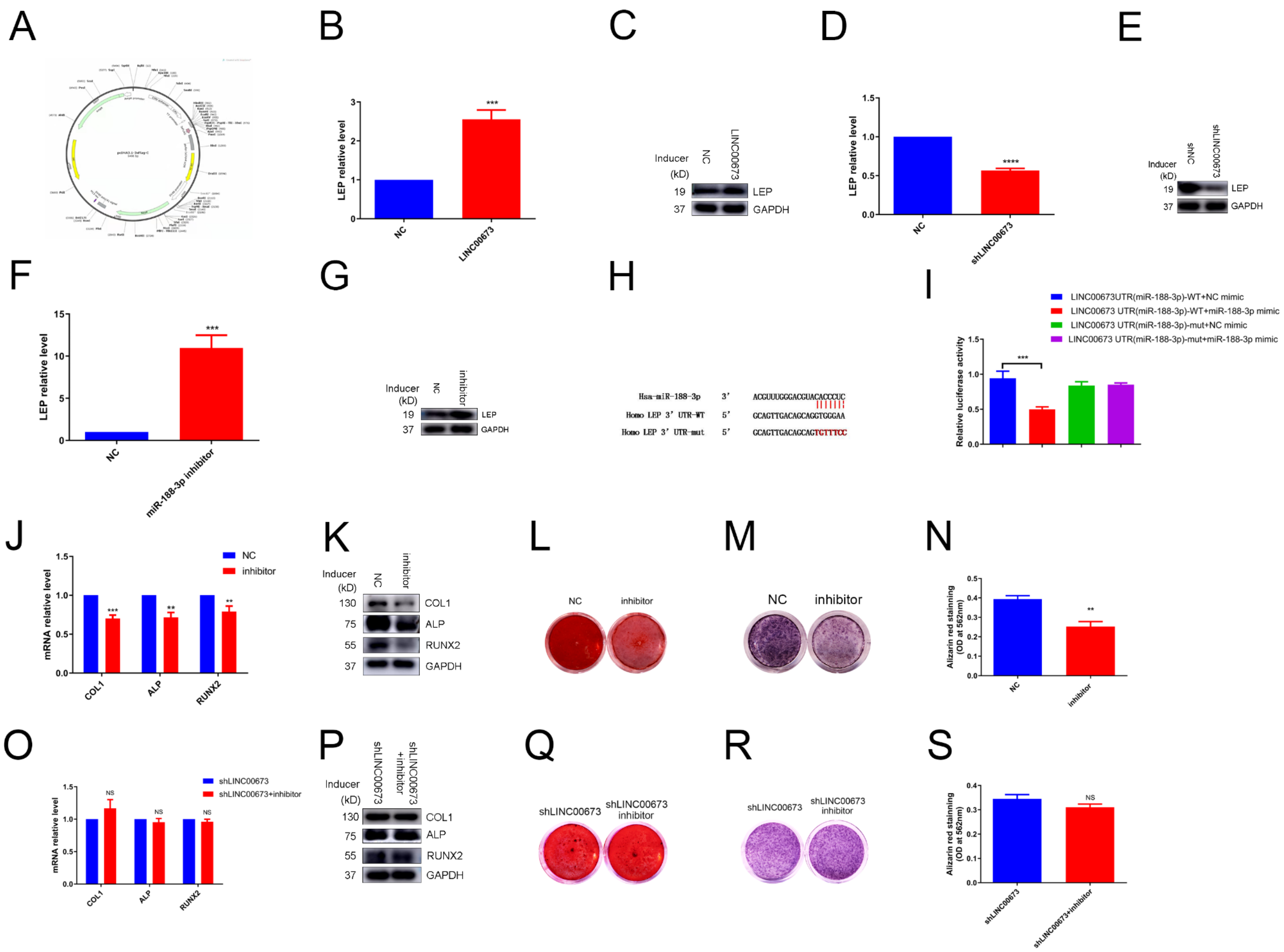

**Fig. 3** LEP was a target of LINC00673/miR-188-3p regulatory network during osteogenesis. (**A**) Diagram of the plasmid vector pattern. (**B**, **C**) LEP mRNA and protein level after transfection with LINC00673 and its controls. (**D**, **E**) LEP mRNA and protein level after transfection with shLINC00673 and its controls. (**F**, **G**) LEP mRNA and protein level after transfection with miR-188-3p inhibitor and its controls. (**H**) A schematic diagram showing the putative binding sites for miR-188-3p in both wild type and mutant LEP 3′-UTRs. (**I**) With dual-luciferase reporter assays, miR-188-3p binding to wild type and mutant LEP 3′- UTR was determined. (**J**, **K**) The expression of Col1, ALP, and Runx2 was measured using qPCR and Western blot after transfection with the miR-188-3p inhibitor and the corresponding controls. (**L**, **M**, **N**) hPDLSCs stained with ALP and Alizarin Red after miR-188-3p transfection, as well as controls. (**O**, **P**) After cotransfection with LINC00673 and a miR-188-3p inhibitor, the expression of Col1, ALP, and Runx2 was measured using qPCR and Western blot. (**Q**, **R**, **S**) ALP and Alizarin red S staining images in PDLSCs co-transfected with LINC00673 and miR-188-3p. Results are presented as mean ± SD (***P* < 0.01, ****P* < 0.001, *****P* < 0.0001)

PDLSCs were subjected to treatment with 20 ng/ml of Licl, a specific activator of the canonical Wnt pathway, in order to investigate its impact on osteogenesis. In comparison to the control cells, the PDLSCs treated with Licl exhibited a heightened ability to undergo osteogenesis following a seven-day period of osteogenic induction, as evidenced by the results presented in Fig. 4K and N.

Wnt signals encompass various pathways, including the canonical "Wnt/β-catenin" pathway, the "Wnt/Ca2+" pathway, and the "Wnt/polarity" pathway, which is also referred to as "planar polarity". According to the Gene Set Enrichment Analysis (GSEA), LINC00673 was found to be potentially linked to the canonical Wnt pathway. In order to investigate the potential involvement of LINC00673/LEP in osteogenic differentiation through the acceleration of the canonical Wnt pathway, we conducted a Western blot analysis to assess the expression levels of total β-catenin and GSK-3β proteins. The findings of the study indicate a significant decrease in the protein level of β-catenin in the group overexpressing LINC00673, while a significant increase was observed in the group with LINC00673 knock-down. The expression of Glycogen Synthase Kinase 3 Beta (GSK-3β) protein was significantly upregulated in the group overexpressing LINC00673, while it was downregulated in the group with LINC00673 knockdown (refer to Fig. 4H and I). Additionally, PDLSCs were subjected to co-transfection with both LINC00673 and Licl. Our findings indicate that the overexpression of LINC00673 resulted in the inhibition of the canonical Wnt pathway, as demonstrated

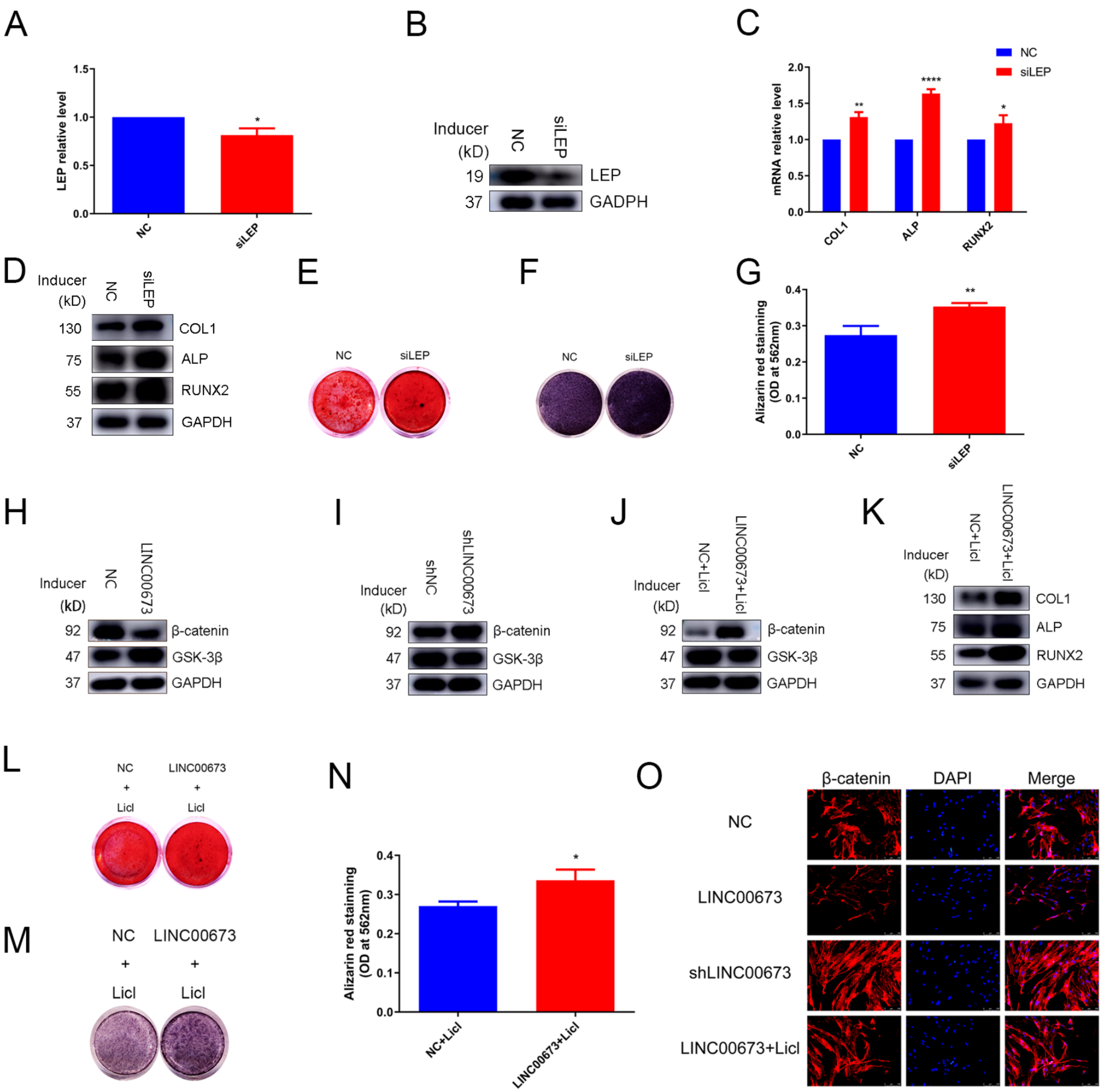


**Fig. 4** LEP may regulates osteogenic differentiation of PDLSCs by inhibiting the canonical Wnt pathway. (**A**, **B**) RT-qPCR and Western blot analysis were used to determine the effects of siLEP transfection in PDLSCs. (**C**, **D**) qPCR and Western blot were used to assess the expression of Col1, ALP, and Runx2 after transfection with siLEP and controls. (**E**, **F**, **G**) Images of ALP and Alizarin red S staining in PDLSCs transfected with siLEP and controls. (**H**, **I**) After transfection with LINC00673, sh-LINC00673, and the corresponding controls, the protein expression of β - catenin and GSK – 3β was measured. (**J**) After being transfected with LINC00673 under Licl treatment or not after osteogenic induction, the protein levels of β - catenin and GSK– 3β were measured. (**K**) Under Licl treatment and the corresponding controls, protein expression of COL1, ALP, and RUNX2 was evaluated. (**L**, **M**, **N**) ARS and ALP staining in PDLSCs transfected with LINC00673 treated with Licl and the corresponding controls. (**O**) By immunofluorescence staining, the expression of LINC00673, sh-LINC00673, LINC00673 + Licl, and control cells, was detected. Data represent the mean ± S.D. (*$P < 0.05$, **$P < 0.01$, ****$P < 0.0001$)

in Fig. 4H. The activation of Licl appears to fully counteract the adverse osteogenic impacts of LINC00673, as demonstrated in Fig. 4J and K. This finding implies that LINC00673 may regulate the process of osteogenic differentiation via the canonical Wnt pathway. On a cellular level, the conclusion was validated through the results of Alizarin Red and ALP staining. The findings indicated an increase in osteogenic activities in the group co-transfected with LINC00673 and Licl, as demonstrated in Appendix 3: Figure 4L and N. The observed trend was confirmed through immunofluorescence staining, as depicted in Fig. 4O.

## Discussion

The utilization of microarray technology has significantly enhanced the capacity of researchers to handle and scrutinize biological data. In order to assess potential mechanisms, it is possible to directly observe the expression of targets such as DNA [20], RNA [21], and signaling pathways [22] through the use of microarray and gene network analysis. In recent years, several studies have utilized IncRNAs as a tool to investigate human diseases. This has resulted in the emergence of a novel approach for identifying prognoses and treatments based on the internal mechanisms of these IncRNAs. Despite the growing interest in lncRNAs and their potential roles in various biological processes, only a limited number of studies have focused on investigating the expression and function of lncRNAs in PDLSCs. For instance, Many studies have found that LINC00673 exhibits different biological roles in non-small cell lung cancer [12], tongue cance [17], and pancreatic cancer [19]. Elevated levels of LINC00673 have been reported in various types of human cancers, such as melanoma [23] and epithelial ovarian cancer (EOC) [24]. Additionally, increased expression of LINC00673 has been observed in breast cancer [25–27], liver cancer [28], and thyroid cancer [29]. Despite the growing interest in the role of long noncoding RNAs (lncRNAs) in various biological processes, the effect of LINC00673 on osteogenic differentiation has not been investigated. Our study aimed to fill this gap by examining the impact of LINC00673 on osteogenesis in PDLSCs under AGEs conditions. Our findings suggest that LINC00673 exerts a negative regulatory effect on osteogenesis in PDLSCs under AGEs conditions. Our study revealed that the overexpression of LINC00673 in PDLSCs led to a significant reduction in both in vivo and in vitro osteogenesis (Fig. 1F and J). Conversely, inhibition of LINC00673 resulted in an increased osteogenic potential of PDLSCs, as demonstrated in Fig. 1K and O. These findings suggest that LINC00673 may play a crucial role in regulating osteogenesis in PDLSCs. Given the sponge effect of LINC00673, a deeper investigation was conducted to elucidate the underlying interaction between LINC00673 and miRNA.

The bioinformatics algorithms have predicted the presence of a miR-188-3p binding site within LINC00673. Numerous noncoding RNAs have been discovered to act as sponges for miR-188-3p. The downregulation of miR-188-3p resulted in the restoration of osteogenic differentiation in dental pulp stem cells (DPSCs) following the silencing of hsa-circ-0026827, as reported by reference [30]. Pei et al. conducted a study which revealed that RP11-283G6.5 functions as a tumor suppressive lncRNA in breast cancer by regulating the miR-188-3p/TMED3/Wnt/β-catenin signaling pathway [27]. The study findings suggest that MiR-188-3p serves as a novel independent prognostic factor for patients with colorectal cancer. This observation can be attributed, at least in part, to the impact of MiR-188-3p on the expression of MLLT4 and the migration of cancer cells, as reported in previous research [31]. The findings obtained from in vivo xenograft nude mice models indicate that lentiviral-based re-expression of miR-188-3p can significantly impede tumor growth [32]. In the present study, it was observed that LINC00673 acted as a sponge and exhibited a negative regulatory effect on the expression of miR-188-3p. The interaction between LINC00673 and miR-188-3p in PDLSCs was confirmed through a luciferase reporter assay. Moreover, the stimulatory effects of LINC00673 on osteogenic differentiation were reversed by miR-188-3p, suggesting the participation of the regulatory network of LINC00673/miR-188-3p in the process of osteogenic differentiation of PDLSCs.

The investigation of miR-188-3p's target genes was further pursued. According to TargetScan analyses, the 3' Untranslated Region (3'- UTR) of the LEP mRNA harbors a potential binding site for miR-188-3p. Leptin, a protein consisting of 167 amino acids, exhibits structural similarities with the cytokine family and is predominantly synthesized by adipocytes. The hormone Leptin is synthesized through the expression of the LEP gene. Leptin has been identified as a biomarker that is linked to obesity and metabolic disorders. New associations have been reported, including but not limited to inflammatory bowel disease, cancer, bone formation, and asthma, among others [33]. The impact of this substance on bone is not limited to its direct effects on osteoblasts and chondrocytes. It also exerts an indirect influence on bone through its effects on the hypothalamus and sympathetic nervous system, as well as its impact on other hormones. The balance of central and peripheral effects of leptin on bone has been a topic of considerable controversy. This balance may be influenced by various factors, including species, body weight, baseline circulating leptin levels, and specific pathologies. The relationship between adiposity and bone density is likely influenced by human leptin, which enables the skeleton to respond adequately to variations in soft tissue mass [34]. Historically, there has been a belief that obesity may have a protective effect on bone health and mineralization [35]. While this may hold some truth, recent studies have shown that obese patients with sarcopenia may experience low bone density and increased fragility [36]. According to previous research, there is evidence to suggest that obesity has a negative impact on bone mass density in rats [37]. The condition of obesity has been found to potentially elevate the likelihood of fractures in human beings [38]. According to a study cited in reference 39, high doses of leptin were found to decrease bone formation and increase bone resorption [39]. The impact of leptin on bone

metabolism remains a topic of significant debate, despite its known influence on this physiological process. The relationship between leptin and bone in cases of obesity is complicated by the presence of leptin resistance, as noted in previous research [40]. The findings of our study indicate that under conditions of AGEs, LINC00673/miR-188-3p complex targets LEP, thereby regulating the proliferation and differentiation of PDLSCs. The binding of miR-188-3p to LEP was confirmed through luciferase reporter assays. The expression of LEP was found to have a negative correlation with miR-188-3p and a positive correlation with LINC00673. This suggests a potential regulatory relationship between LEP, miR-188-3p, and LINC00673. The results of this study indicate that the LEP gene is a downstream target of the LINC00673/miR-188-3p axis.

There is mounting evidence indicating that the canonical Wnt pathway plays a critical role in the process of bone repair. The canonical Wnt signaling pathway plays a crucial role in maintaining the viability, function, and integrity of bone cells. The present study aimed to verify our hypothesis that the regulation of osteogenesis by LINC00673 involves the canonical Wnt pathway. To this end, we conducted an investigation into the potential involvement of the canonical Wnt pathway in the regulation of osteogenesis by LINC00673. Under conditions of AGEs, knockdown of LINC00673 resulted in an increase in the expression of β-catenin protein. Conversely, the overexpression of LINC00673 resulted in the attenuation of the Wnt/β-catenin signaling pathway. Moreover, the activation of the canonical Wnt pathway through Licl effectively nullifies the negative osteogenic impacts of LINC00673 (as shown in Fig. 4K N). This suggests that LINC00673 could potentially regulate the osteogenic differentiation of PDLSCs by means of the canonical Wnt pathway.

However, given the restricted sample size analyzed in this investigation, additional research is required to explore the LINC00673/miR-188-3p/LEP axis in periodontitis PDLSCs. It is crucial to recognize and address the limitations inherent in the study. It is possible that the downstream targets of LINC00673 extend beyond the miR-188-3p/LEP axis. Therefore, further research is required to gain a more comprehensive understanding of the role of LINC00673 in the pathogenesis of periodontitis.

Based on our research, it can be concluded that the presence of LINC00673 inhibits the osteogenesis of PDLSCs when exposed to AGEs. Under conditions of AGEs, LINC00673 functions as an inhibitor of miR-188-3p expression. This inhibition directly regulates LEP and activates the canonical Wnt pathway, thereby regulating the behavior of PDLSCs. The LINC00673/miR-188-3p/LEP axis presents a potential avenue for exploring therapeutic options for periodontal disease associated with diabetes.

## Supplementary Information

The online version contains supplementary material available at https://doi.org/10.1186/s13018-025-05562-0 .

Supplementary Material 1

Supplementary Material 2

Supplementary Material 3

Supplementary Material 4

Supplementary Material 5

**Acknowledgements**

We are grateful to Jing Lan's group at The School and Hospital of Stomatology, Cheeloo College of Medicine, Shandong University (Shandong, China) for their generous donation of 293T cells, which were used in this study.

**Author contributions**

Y Y, X X W, and J Z created the fundamental idea, examined the majority of the data, and authored the initial draft. The remaining authors helped to improve the concepts, run further studies, and finalize the work. All writers have provided their final consent and agree to be held liable for all parts of the work.

**Funding**

This research was funded by the Shandong Province Natural Science Fundation Youth Branch (ZR2021QH340) and the Shandong University Horizontal Scientific Research Fund (1350022003).

**Data availability**

No datasets were generated or analysed during the current study.

## Declarations

**Ethics approval and informed consent**

This work was authorized by Shandong University's Human Subjects Ethics Board (NO. 20020402 & NO. 20220403) and was carried out in compliance with the Helsinki Declaration of 1975, as updated in 2013. Each patient provided written informed consent. All experimental techniques have been authorized by the hospital's Laboratory Animal Care and Use Committees. To participate in the study, each individual provided informed permission.

**Competing interests**

The authors declare no competing interests.

Received: 11 January 2025 / Accepted: 3 February 2025
Published online: 14 February 2025

**References**

1. Reich E, Hiller K-A. Reasons for tooth extraction in the western states of Germany. Commun Dent Oral Epidemiol. 1993;21:379–83.
2. Anastasiadou E, Jacob LS, Slack FJ. Non-coding RNA networks in cancer. Nat Rev Cancer. 2018;18:5–18.
3. Kamali K, Korjan ES, Eftekhar E, Malekzadeh K, Soufi FG. The role of miR-146a on NF-κB expression level in human umbilical vein endothelial cells under hyperglycemic condition. BLL. 2016;117:376–80.
4. Rajan TS, Diomede F, Bramanti P, Trubiani O, Mazzon E. Conditioned medium from human gingival mesenchymal stem cells protects motor-neuron-like NSC-34 cells against scratch-injury-induced cell death. Int J Immunopathol Pharmacol. 2017;30:383–94.
5. Trubiani O, Guarnieri S, Diomede F, Mariggiò MA, Merciaro I, Morabito C, et al. Nuclear translocation of PKCα isoenzyme is involved in neurogenic

commitment of human neural crest-derived periodontal ligament stem cells. Cell Signal. 2016;28:1631–41.
6. Gurav AN. Advanced Glycation End products: a link between Periodontitis and Diabetes Mellitus? Curr Diabetes Rev. 2013;9:355–61.
7. Wu D, Yin L, Sun D, Wang F, Wu Q, Xu Q, et al. Long noncoding RNA TUG1 promotes osteogenic differentiation of human periodontal ligament stem cell through sponging microRNA-222-3p to negatively regulate Smad2/7. Arch Oral Biol. 2020;117:104814.
8. Liao J, Yu X, Hu X, Fan J, Wang J, Zhang Z, et al. lncRNA H19 mediates BMP9-induced osteogenic differentiation of mesenchymal stem cells (MSCs) through notch signaling. Oncotarget. 2017;8:53581–601.
9. Zhuang W, Ge X, Yang S, Huang M, Zhuang W, Chen P, et al. Upregulation of lncRNA MEG3 promotes osteogenic differentiation of mesenchymal stem cells from multiple myeloma patients by targeting BMP4 transcription. Stem Cells. 2015;33:1985–97.
10. Xu Y, Qin W, Guo D, Liu J, Zhang M, Jin Z. LncRNA-TWIST1 promoted osteogenic differentiation both in PPDLSCs and in HPDLSCs by inhibiting TWIST1 expression. Biomed Res Int. 2019;2019:e8735952.
11. Peng W, Deng W, Zhang J, Pei G, Rong Q, Zhu S. Long noncoding RNA ANCR suppresses bone formation of periodontal ligament stem cells via sponging miRNA-758. Biochem Biophys Res Commun. 2018;503:815–21.
12. Zhang J, Tao Z, Wang Y. Long non–coding RNA DANCR regulates the proliferation and osteogenic differentiation of human bone-derived marrow mesenchymal stem cells via the p38 MAPK pathway. Int J Mol Med. 2018;41:213–9.
13. Li X, Huang Y, Han Y, Yang Q, Zheng Y, Li W. LncPVT1 regulates osteogenic differentiation of human periodontal ligament cells via miR-10a-5p/brain-derived neurotrophic factor. J Periodontol. 2022;93:1093–106.
14. Liu Z, He Y, Xu C, Li J, Zeng S, Yang X, et al. The role of PHF8 and TLR4 in osteogenic differentiation of periodontal ligament cells in inflammatory environment. J Periodontol. 2021;92:1049–59.
15. Lu W, Zhang H, Niu Y, Wu Y, Sun W, Li H, et al. Long non-coding RNA linc00673 regulated non-small cell lung cancer proliferation, migration, invasion and epithelial mesenchymal transition by sponging miR-150-5p. Mol Cancer. 2017;16:118.
16. Roth A, Boulay K, Groß M, Polycarpou-Schwarz M, Mallette FA, Regnier M, et al. Targeting LINC00673 expression triggers cellular senescence in lung cancer. RNA Biol. 2018;15:1499–511.
17. Yu J, Liu Y, Gong Z, Zhang S, Guo C, Li X, et al. Overexpression long non-coding RNA LINC00673 is associated with poor prognosis and promotes invasion and metastasis in tongue squamous cell carcinoma. Oncotarget. 2016;8:16621–32.
18. Li N, Cui Z, Huang D, Gao M, Li S, Song M, et al. Association of LINC00673 rs11655237 polymorphism with cancer susceptibility: a meta-analysis based on 23,478 subjects. Genomics. 2020;112:4148–54.
19. Gong Y, Dai H, Shu J, Liu W, Bie P, Zhang L. LNC00673 suppresses proliferation and metastasis of pancreatic cancer via target miR-504/ HNF1A. J Cancer. 2020;11:940–8.
20. Song B-N, Kim S-K, Chu I-S. Bioinformatic identification of prognostic signature defined by copy number alteration and expression of CCNE1 in non-muscle invasive bladder cancer. Exp Mol Med. 2017;49:e282–282.
21. Omori K, Mitsuhashi M, Todorov I, Rawson J, Shiang K-D, Kandeel F, et al. Microassay for glucose-Induced Preproinsulin mRNA expression to assess islet functional potency for islet transplantation. Transplantation. 2010;89:146.
22. Kim WT, Seo S-P, Byun YJ, Kang H-W, Kim Y-J, Lee S-C, et al. Garlic extract in bladder cancer prevention: evidence from T24 bladder cancer cell xenograft model, tissue microarray, and gene network analysis. Int J Oncol. 2017;51:204–12.
23. Schmidt K, Carroll JS, Yee E, Thomas DD, Wert-Lamas L, Neier SC, et al. The lncRNA SLNCR recruits the androgen receptor to EGR1-Bound genes in Melanoma and inhibits expression of tumor suppressor p21. Cell Rep. 2019;27:2493–e25074.
24. Zheng T, Qiu J, Li C, Lin X, Tang X, Hua K. Long noncoding RNA LINC00673 promotes the proliferation and metastasis of epithelial ovarian cancer by associating with opioid growth factor receptor. OTT. 2019;12:6145–56.<\/p>
25. Xia E, Shen Y, Bhandari A, Zhou X, Wang Y, Yang F, et al. Long non-coding RNA LINC00673 promotes breast cancer proliferation and metastasis through regulating B7-H6 and epithelial-mesenchymal transition. Am J Cancer Res. 2018;8:1273–87.
26. Qiao K, Ning S, Wan L, Wu H, Wang Q, Zhang X, et al. LINC00673 is activated by YY1 and promotes the proliferation of breast cancer cells via the miR-515-5p/MARK4/Hippo signaling pathway. J Experimental Clin Cancer Res. 2019;38:418.
27. Pei J, Zhang S, Yang X, Han C, Pan Y, Li J, et al. Long non-coding RNA RP11-283G6.5 confines breast cancer development through modulating miR-188-3p/TMED3/Wnt/β-catenin signalling. RNA Biol. 2021;18:287–302.
28. Yang T, Li J, Wen Y, Tan T, Yang J, Pan J et al. LINC00673 rs11655237 C > T Polymorphism Impacts Hepatoblastoma Susceptibility in Chinese Children. Frontiers in Genetics [Internet]. 2019 [cited 2023 Jan 24];10. Available from: https://www.frontiersin.org/articles/https://doi.org/10.3389/fgene.2019.00506 IF: 2.8 Q2 B3
29. Xia E, Bhandari A, Shen Y, Zhou X, Wang O. lncRNA LINC00673 induces proliferation, metastasis and epithelial-mesenchymal transition in thyroid carcinoma via Kruppel-like factor 2. Int J Oncol. 2018;53:1927–38.
30. Ji F, Zhu L, Pan J, Shen Z, Yang Z, Wang J et al. hsa_circ_0026827 Promotes Osteoblast Differentiation of Human Dental Pulp Stem Cells Through the Beclin1 and RUNX1 Signaling Pathways by Sponging miR-188-3p. Frontiers in Cell and Developmental Biology [Internet]. 2020 [cited 2023 Jan 24];8. Available from: https://www.frontiersin.org/articles/https://doi.org/10.3389/fcell.2020.00470 IF: 4.6 Q1 B2
31. Pichler M, Stiegelbauer V, Vychytilova-Faltejskova P, Ivan C, Ling H, Winter E, et al. Genome-wide miRNA analysis identifies mir-188-3p as a novel prognostic marker and molecular factor involved in colorectal carcinogenesis. Clin Cancer Res. 2017;23:1323–33.
32. Pei J, Zhang J, Yang X, Wu Z, Sun C, Wang Z, et al. TMED3 promotes cell proliferation and motility in breast cancer and is negatively modulated by miR-188-3p. Cancer Cell Int. 2019;19:75.
33. de Luis DA, Perez Castrillón JL, Dueñas A. Leptin and obesity. Minerva Med. 2009;100:229–36.
34. Reid IR, Baldock PA, Cornish J. Effects of Leptin on the Skeleton. Endocr Rev. 2018;39:938–59.
35. Cruz-Jentoft AJ, Baeyens JP, Bauer JM, Boirie Y, Cederholm T, Landi F, et al. Sarcopenia: European consensus on definition and diagnosis: report of the European Working Group on Sarcopenia in Older people. Age Ageing. 2010;39:412–23.
36. Grinspoon S, Thomas E, Pitts S, Gross E, Mickley D, Miller K, et al. Prevalence and predictive factors for Regional Osteopenia in Women with Anorexia Nervosa. Ann Intern Med. 2000;133:790–4.
37. Feresin RG, Johnson SA, Elam ML, Jo E, Arjmandi BH, Hakkak R. Effects of obesity on bone Mass and Quality in Ovariectomized Female Zucker rats. J Obes. 2014;2014:e690123.
38. Caffarelli C, Alessi C, Nuti R, Gonnelli S. Divergent effects of obesity on fragility fractures. CIA. 2014;9:1629–36.
39. Martin A, David V, Malaval L, Lafage-Proust M-H, Vico L, Thomas T. Opposite effects of Leptin on Bone metabolism: a dose-dependent balance related to Energy Intake and insulin-like Growth Factor-I pathway. Endocrinology. 2007;148:3419–25.
40. Hannema SE, Wit JM, Houdijk MECAM, van Haeringen A, Bik EC, Verkerk AJMH, et al. Novel leptin receptor mutations identified in two girls with severe obesity are Associated with increased bone Mineral Density. HRP. 2016;85:412–20.

## Publisher's note

Springer Nature remains neutral with regard to jurisdictional claims in published maps and institutional affiliations.
