# Supplementary material for: Molecular Regulators for the Wnt/β-Catenin Signalling Pathway for Bone Remodelling in Periodontitis: A Scoping Review
Source: Int J Mol Sci. 2026 Oct 14;27(20):9117. doi: 10.3390/ijms27209117 (PMC13705578; doi:10.3390/ijms27209117)
Supplement: Supplementary file 1 [file ijms-27-09117-s001.zip › Supplementary Data/Papers/in vitro/Long noncoding RNA related to periodontitis interacts with miR-182 to upregulate osteogenic differentiation in periodontal mesenchymal stem cells of periodontitis patients-1.pdf]

OPEN

Citation: Cell Death and Disease (2016) 7, e2327; doi:10.1038/cddis.2016.125
Official journal of the Cell Death Differentiation Association

www.nature.com/cddis

Long noncoding RNA related to periodontitis interacts with miR-182 to upregulate osteogenic differentiation in periodontal mesenchymal stem cells of periodontitis patients

L Wang[1,4], F Wu[2,4], Y Song[3], X Li[1], Q Wu[1], Y Duan*[,1] and Z Jin*[,1]

Periodontitis impairs the osteogenic differentiation of human periodontal mesenchymal stem cells (hPDLSCs), but the underlying molecular mechanisms are still poorly understood. Long noncoding RNAs (lncRNAs) have been demonstrated to have significant roles under both physiologic and pathological conditions. In this study, we performed comprehensive lncRNA profiling by lncRNA microarray analysis and identified a novel lncRNA, osteogenesis impairment-related lncRNA of PDLSCs from periodontitis patients (lncRNA-POIR), the expression of which was significantly decreased in PDLSCs from periodontitis patients (pPDLSCs) and was upregulated by osteogenic induction. To study the functions of lncRNA-POIR, we prepared cells with overexpression and knockdown of lncRNA-POIR and found that lncRNA-POIR positively regulated osteogenic differentiation of hPDLSCs and pPDLSCs both *in vitro* and *in vivo*. Using quantitative real-time PCRs (qPCRs) and luciferase reporter assays, we demonstrated that lncRNA-POIR may act as a competing endogenous RNA (ceRNA) for miR-182, leading to derepression of its target gene, *FoxO1*. In this process, lncRNA-POIR and miR-182 suppress each other and form a network to regulate *FoxO1*. *FoxO1* increased bone formation of pPDLSCs by competing with *TCF-4* for *β-catenin* and inhibiting the canonical Wnt pathway. Finally, inflammation increases miR-182 expression through the nuclear factor-κB pathway, and the miR-182 overexpression in the inflammatory microenvironment resulted in an imbalance in the lncRNA-POIR-miR-182 regulatory network. In conclusion, our results provide novel evidence that this lncRNA-miRNA (microRNA) regulatory network has a significant role in osteogenic differentiation of pPDLSCs and that it has potential as a therapeutic target in mesenchymal stem cells during inflammation.
Cell Death and Disease (2016) **7,** e2327; doi:10.1038/cddis.2016.125; published online 11 August 2016

Human periodontal mesenchymal stem cells (hPDLSCs) are a type of mesenchymal stem cells (MSCs) that can be isolated from periodontal ligament tissue and that have the potential to differentiate into multiple cell types, including osteogenic, adipogenic and chondrogenic lineages.[1–5] Because of their osteogenic capacity and tissue origin, hPDLSCs are considered to be excellent cellular source for alveolar bone regeneration and repair.[6–11] However, changes in the microenvironment because of age, hypoxia and inflammation can impair the multiplex differentiation potential of hPDLSCs.[12–16] Periodontitis is a highly prevalent chronic inflammatory bone disease, and it is the main cause of tooth loss. Growing evidence suggests that inflammation suppresses the regenerative capacity of hPDLSCs.[17,18] Although some studies have suggested that the Wnt/*β-catenin* and nuclear factor-κB (NF-κB) pathways may have roles in the impairment of osteogenesis of PDLSCs from periodontitis patients (pPDLSCs), the exact underlying mechanisms were still unknown.[18,19]

Noncoding RNAs (ncRNAs), which have been recently recognized, have no protein-coding capabilities but may act as key regulators to control biological and pathological processes.[20] The main types of small ncRNAs are miRNAs, which regulate gene expression by directly binding to the 3′-UTR region of mRNAs directly.[21] A subset of miRNAs, including miR-29b,[22] miR-204/211 (ref. 23) and miR-182,[24] participate in the regulation of osteogenesis of MSCs. Long noncoding RNAs (lncRNAs) are another important type of ncRNAs that are longer than 200 nt in length. Multiple studies have indicated that lncRNAs are involved in and may be

[1]State Key Laboratory of Military Stomatology and National Clinical Research Center for Oral Diseases and Shaanxi Clinical Research Center for Oral Diseases, Department of Orthodontics, School of Stomatology, The Fourth Military Medical University, Xi'an, China; [2]The 451st Hospital of People's Liberation Army, Xi'an, China and [3]Department of Stomatology, The 323rd Hospital of People's Liberation Army, Xi'an, China
*Corresponding author: Y Duan or Z Jin, State Key Laboratory of Military Stomatology and National Clinical Research, Center for Oral Diseases and Shaanxi Clinical Research Center for Oral Diseases, Department of Orthodontics, School of Stomatology, The Fourth Military Medical University, 145 West Changle Road, Xi'an, Shaanxi 710032, China. Tel: +86 29 84776235 or +86 29 84776481; Fax: +86 29 83223047; E-mail: duanyz@fmmu.edu.cn or zuolinj@fmmu.edu.cn
[4]These authors contributed equally to this work.

Abbreviations: ALP, alkaline phosphatase; ChIP, chromatin immunoprecipitation; Col1, collagen type1; DKK1, dickkopf-1; GO, gene ontology; HA-TCP, hydroxyapatite-tricalcium phosphate; H&E, hematoxylin and eosin; hPDLSCs, human periodontal mesenchymal stem cells; lncRNAs, long noncoding RNAs; lncRNA-POIR, osteogenesis impairment-related lncRNA of periodontal mesenchymal stem cells from periodontitis patients; miRNAs, microRNAs; MSCs, mesenchymal stem cells; NF-κB, nuclear factor-κB; P65, RelA; pPDLSCs, periodontal mesenchymal stem cells from periodontitis patients; qPCR, quantitative real-time PCR; RISC, RNA-induced silencing complex; RIP, RNA-binding protein immunoprecipitation

Received 10.1.16; revised 15.3.16; accepted 29.3.16; Edited by D Aberdam

vital to a variety of diseases associated with aberrant cellular control, including neurological, autoimmune, and cardiovascular conditions, as well as cancer.[25] Moreover, substantial evidence indicates that some lncRNAs, such as MEG3, H19, and lncRNA-ANCR, could regulate stem cell osteogenic differentiation under normal and aberrant conditions.[26–28]

Unlike miRNAs, lncRNAs can either positively or negatively regulate the expression of protein-coding genes through a variety of mechanisms. Recently recognized, a large number of lncRNAs and miRNAs suppress each other as ceRNAs and form an accurate regulatory network to regulate target genes of miRNAs.[29] On the one hand, lncRNAs that regulate transcription involves base pairing with miRNAs, which effectively depletes miRNAs by acting as a sponge or a decoy.[30–32] On the other hand, miRNAs are able to bind to lncRNAs and trigger lncRNAs decay.[33] There are growing evidences that the regulatory network composed of lncRNAs and miRNAs have significant roles in cellular differentiation. For instance, expression of the lncRNA LINCMD1 is induced during myoblast differentiation, and it acts as a sponge of miR-133 and miR-135 to control expression of the transcription factors MAML1 and MEF2C, which activate muscle-specific gene expression.[34] Although roles of lncRNA-miRNA networks in various biological processes have been reported, their functions in the osteogenesis of MSCs under normal or inflammatory conditions remain poorly understood.

In this study, we have performed comprehensive lncRNA profiling and have found that a crucial lncRNA, pPDLSC osteogenesis impairment-related lncRNA (lncRNA-POIR), positively regulates the osteogenic differentiation of hPDLSCs in inflammatory microenvironments. During this process, lncRNA-POIR and miR-182 suppress each other and form a network to regulate the target gene of miR-182, *FoxO1*. *FoxO1* increases bone formation by inhibiting canonical Wnt signaling. Furthermore, aberrant activation of the NF-κB pathway during inflammation may increase miR-182 expression and result in an imbalance in the lncRNA-POIR-miR-182 regulatory network. This report is the first to demonstrate the control of osteogenic differentiation of MSCs by an lncRNA-miRNA regulatory network in an inflammatory microenvironment.

Results

LncRNA-POIR expression, which is significantly altered in hPDLSCs and pPDLSCs, is correlated with osteogenic differentiation of pPDLSCs. To identify lncRNAs that affect the osteogenic potential of pPDLSCs, we assessed the lncRNA expression profiles of two pairs of hPDLSCs and pPDLSCs using microarrays. Our analysis resulted in the identification of 89 lncRNAs and 387 mRNAs that were differentially expressed (fold change >2.0, P-value <0.05) between the two groups of cells (Figure 1a and Supplementary Figure S3A).

The results of Gene Ontology (GO) and pathway analyses of the differentially expressed mRNAs are shown in Supplementary Figures S3B–D.

Next, we confirmed the microarray results using qPCR. The expression changes of the lncRNAs detected using qPCR were consistent with the microarray results (Figure 1b). Then, we chose five lncRNAs with high fold changes (fold change >5.0, P-value <0.05) between the hPDLSCs and pPDLSCs and assessed whether they affect pPDLSC osteogenesis. Among the studied samples, expression of the lncRNA ENST00000446358 (lncRNA-POIR) was the most significantly altered between the non-induced group and osteogenic-induced group (Figure 1c), exhibiting a gradual increase until 14 days after osteogenic induction (Figure 1d). Moreover, the lncRNA-POIR and *Runx2/Col1 (collagen type1)* expression levels were highly correlated at 0, 1, 7 and 14 days after osteogenic induction (Figure 1e).

LncRNA-POIR increases osteogenic differentiation of pPDLSCs. To determine the biological effects of lncRNA-POIR on the osteogenic differentiation of pPDLSCs, we constructed shlncRNA-POIR plasmids for lncRNA-POIR knockdown (shlncRNA-POIR) and lncRNA-POIR-overexpressing lentiviruses for lncRNA-POIR overexpression. To control for potential off-target shRNA effects, three different shRNAs were designed against lncRNA-POIR, and the most efficient construct was selected for transfection (Figure 2a). We also selected cells that stably overexpressed lncRNA-POIR (Figure 2b).

We found that lncRNA-POIR overexpression significantly increased the mRNA levels of osteogenic genes including *Runx2*, *ALP* and *Col1* in pPDLSCs (Figure 2c). Conversely, shlncRNA-POIR decreased the expression of these genes in pPDLSCs (Figure 2d). Alizarin red staining, alkaline phosphatase *(ALP)* staining, and *ALP* activity assay also revealed that lncRNA-POIR overexpression increased *ALP* activity and mineralized bone matrix formation in pPDLSCs, whereas they were decreased by shlncRNA-POIR (Figures 2e–g).

To further evaluate the osteogenic function of lncRNA-POIR in pPDLSCs *in vivo*, pPDLSCs from the control groups, lncRNA-POIR-overexpressing groups, and shlncRNA-POIR groups were loaded onto hydroxyapatite-tricalcium phosphate (HA-TCP) and implanted into NOD/SCID mice for 4 weeks. The new bone tissue stained red with hematoxylin and eosin (H&E) stain and stained blue with Masson's Trichrome stain; in addition, osteoid formation was increased in the lncRNA-POIR-overexpressing group compared with the negative control groups. Conversely, osteoid formation by pPDLSCs was decreased in the shlncRNA-POIR group (Figures 2h and i). These results strongly suggest that lncRNA-POIR promotes the osteogenesis of pPDLSCs both *in vitro and in vivo.*

LncRNA-POIR knockdown decreases osteogenic differentiation of hPDLSCs. To further demonstrate that the downregulation of lncRNA-POIR expression causes decreased osteogenesis in pPDLSCs compared with hPDLSCs, we transfected shlncRNA-POIR plasmids into hPDLSCs and observed the effects on osteogenesis. The results demonstrated that shlncRNA-POIR decreased the mRNA levels of *Runx2, ALP* and *Col1, ALP* activity and mineralized bone matrix formation in hPDLSCs (Figures 3a–d).

Next, hPDLSCs from negative control groups and shlncRNA-POIR groups were loaded onto HA-TCP and implanted into NOD/SCID mice for 4 weeks as described above. The results showed that the hPDLSCs in the

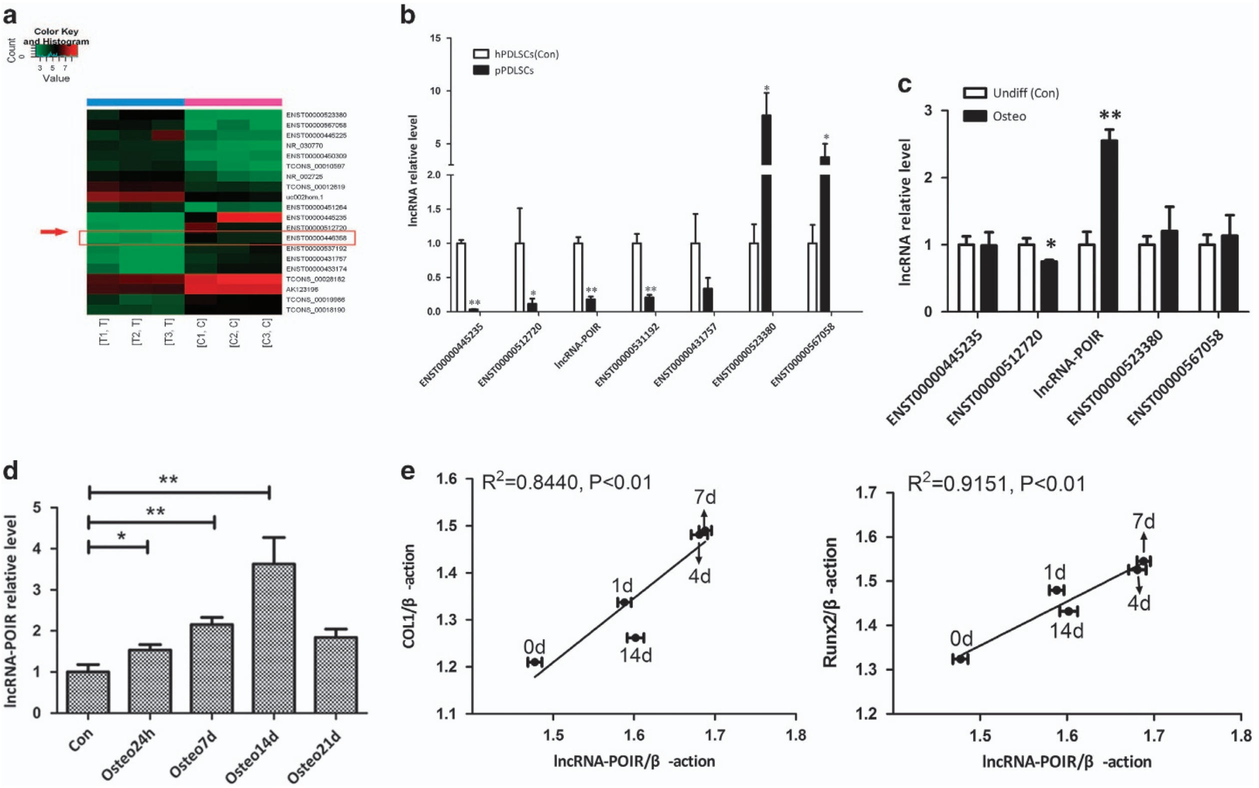

Figure 1 LncRNA-POIR expression, which is significantly inhibited in inflammatory microenvironments, correlates with the osteogenic differentiation of pPDLSCs. (**a**) Heat map of differentially expressed lncRNAs (the top 10 in upregulated lncRNAs and the top 10 in downregulated lncRNAs) between hPDLSCs and pPDLSCs. (**b**) The results were confirmed using qPCR. (**c**) The expression levels of lncRNAs with high fold changes (fold change >5.0, P-value <0.05) were determined by qPCR at 7 days after osteogenic induction. (**d**) The expression levels of lncRNA-POIR were determined by qPCR at 0, 1, 7 and 14 days. (**e**) Correlation analysis between lncRNA-POIR levels and Runx2 and Col1 mRNA levels in pPDLSCs 0, 1, 7 and 14 days after osteogenic induction. All experiments were repeated three times. A total of six samples (three pPDLSCs and three hPDLSCs from six individuals) are tested in lncRNA profiling. Relative expressions of lncRNAs expression were normalized by β-actin in qPCR. All data are the mean ± S.D. $^*P<0.05$, $^{**}P<0.01$ and NS, not significant. C, hPDLSCs; Con, control; lncRNA-POIR, pPDLSCs osteogenesis impaired-related lncRNA, ENST00000446358; Osteo, osteogenic induction; T, pPDLSCs; Undiff, without osteogenic induction

shlncRNA-POIR group formed fewer osteoids than those in the negative control groups (Figures 3e and f).

LncRNA-POIR acts as a sponge of miR-182. Besides, lncRNA-POIR and miR-182 could negatively regulate each other. To determine whether lncRNA-POIR acts as a miRNA sponge that competes with mRNA for binding to miRNAs, we synthesized a miR-182 inhibitor (anti-miR-182) and assessed its efficiency by qPCR. The results revealed that anti-miR-182 significantly inhibited miR-182 expression compared with a blank control and miR-182 inhibitor NC (anti-miR-NC) (Figure 4a). Next, we performed miRNA target site prediction using MicroInspector online software (http: //bioinfo.uni-plovdiv.bg/microinspector). We found that lncRNA-POIR contains a single element complementary to miR-182 (Figure 4b) and that miR-182 expression was promoted by shlncRNA-POIR (Figure 4c). We also found that the lncRNA-POIR level was increased following miR-182 inhibition (Figure 4d), Moreover, lncRNA-POIR and miR-182 expression in pPDLSCs was highly negatively correlated at 0, 1, 7 and 14 days after osteogenic induction (Figure 4e).

To determine whether lncRNA-POIR directly regulates miR-182, we generated luciferase reporter constructs. The results showed that the lncRNA-POIR-wild-type reporter was strongly suppressed by miR-182 (Figure 4f). However, the mutant lncRNA-POIR reporter was not affected by this miRNA. These results indicate that lncRNA-POIR and miR-182 directly regulate each other.

We also found that lncRNA-POIR is associated with the RNA-induced silencing complex (RISC) by performing an RNA-binding protein immunoprecipitation (RIP) assay. The lncRNA-POIR and miR-182 RNA levels were higher in the anti-*Ago2* group compared with the anti-normal IgG group (Figures 4g and h).

LncRNA-POIR modulates FoxO1 through regulation of miR-182. Reports have shown that miR-182 decreases bone formation of MSCs by directly targeting *FoxO1*.[24] Consistent with these findings, the miR-182 inhibitor significantly reduced the *FoxO1* mRNA and protein levels in pPDLSCs (Figures 5a and b). Further, luciferase reporter assay demonstrated that miR-182 inhibited the luciferase activity of the 3′-UTR of the *FoxO1* luciferase reporter (Figures 5c and d). To determine whether lncRNA-POIR affects *FoxO1* expression, we monitored *FoxO1* expression at both the mRNA and protein levels in lncRNA-POIR-overexpressing and shlncRNA-POIR

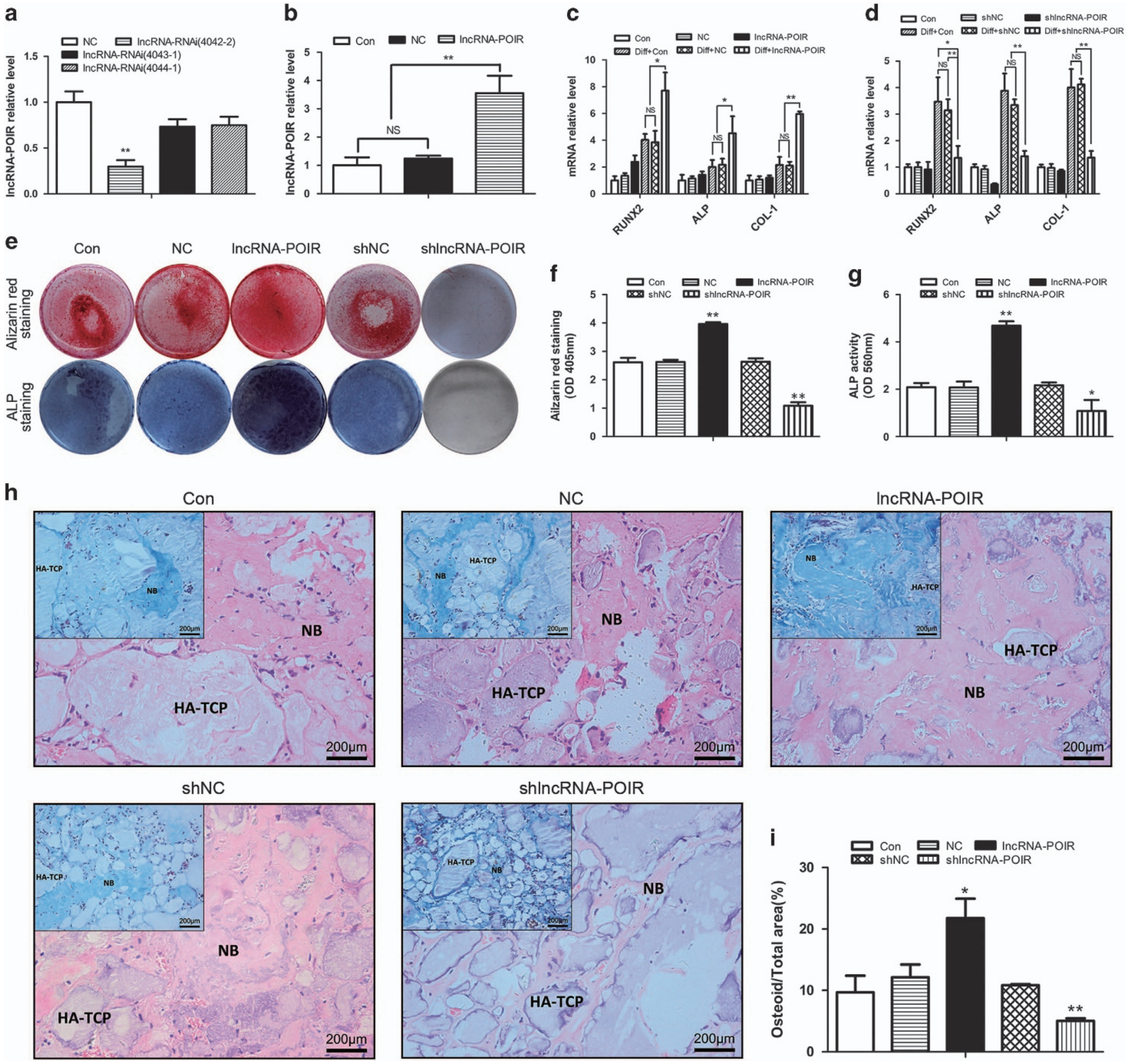

Figure 2 LncRNA-POIR promotes osteogenesis of pPDLSCs. (**a** and **b**) Transfection effects of shlncRNA-POIR plasmids and lncRNA-POIR overexpression lentiviruses were determined by qPCR. (**c** and **d**) Runx2, ALP and Col1 expressions were measured by qPCR at 0 and 7 days after osteogenic induction. (**e**–**g**) Osteogenic differentiations of pPDLSCs were determined by Alizarin Red S, ALP staining and ALP activity assay 7 or 14 days after osteogenic induction. (**h** and **i**) LncRNA-POIR promotes osteogenesis of pPDLSCs *in vivo.* pPDLSCs were mixed with HA-TCP and transplanted into the dorsal region of nude mice for 4 weeks. Then, the results were measured by H&E staining and Masson's trichrome staining. Quantitative analysis of the new bone area determined by Image-Pro Plus 6.0 software (Media Cybernetics, Washington, USA). At least three fields were randomly selected from each transplant. Six implants were engrafted into three mice per treatment. All experiments were repeated three times. Relative expressions of mRNAs and lncRNA-POIR were normalized by β-actin in qPCR. All data are the mean ± S.D. **P*<0.05, ***P*<0.01 and NS, not significant. The scale bar in the micrographs represents 200 nm. Con, Control; diff, osteogenic induction; lncRNA-POIR, lentivirus for upregulating lncRNA-POIR; NB, new bone; NC, lentivirus negative control; OD, optical density; shNC, plasmids negative control, shlncRNA-POIR, plamids for downregulating lncRNA-POIR

pPDLSCs. A significant increase in *FoxO1* expression was observed in response to lncRNA-POIR overexpression. Moreover, shlncRNA-POIR negatively regulated the expression of *FoxO1* (Figures 5e–h).

The miR-182 and FoxO1 have opposite effects on osteogenic differentiation of pPDLSCs. To control for potential off-target si*FoxO1* effects, three different siRNAs were designed against *FoxO1*. Then, we assessed the efficiency of si*FoxO1* by qPCR and western blotting, and the most efficient construct was selected for transfection (Figures 6a and b). Moreover, we assessed the osteogenic function of *FoxO1* using si*FoxO1*. si*FoxO1* was found to inhibit osteogenic processes in pPDLSCs (Figures 6c–f). We also found that the effects of lncRNA-POIR overexpression on osteogenic markers could be offset by si*FoxO1*

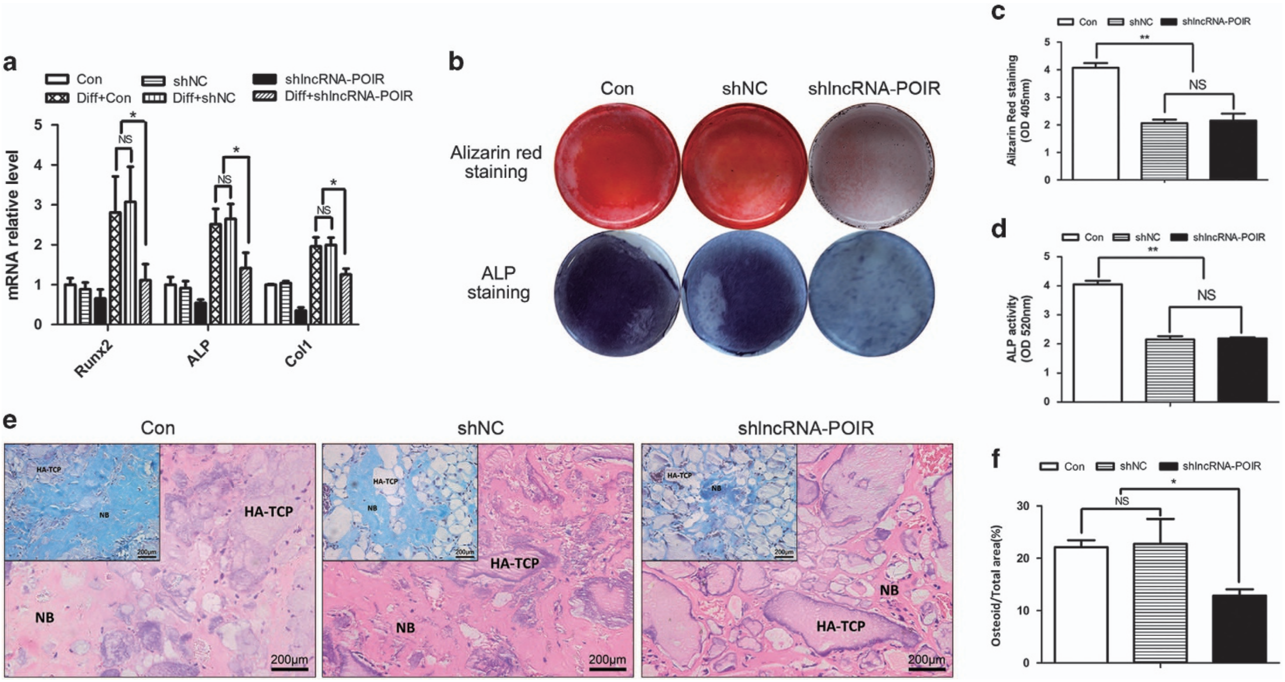

Figure 3 LncRNA-POIR knockdown decreases osteogenic differentiation of hPDLSCs (**a**) Runx2, ALP and Col1 expressions were measured by qPCR at 0 and 7 days after osteogenic induction. (**b**–**d**) Osteogenic differentiations of pPDLSCs were determined by Alizarin Red S, ALP staining and ALP activity assay 7 or 14 days after osteogenic induction. (**e** and **f**) shLncRNA-POIR inhibits osteogenesis of hPDLSCs *in vivo*. hPDLSCs were mixed with HA-TCP and transplanted into the dorsal region of nude mice for 4 weeks. Then, the results were measured by H&E staining and Masson's trichrome staining. Quantitative analysis of the new bone area determined by Image-Pro Plus 6.0 software. At least three fields were randomly selected from each transplant. Six implants were engrafted into three mice per treatment. All experiments were repeated three times. Relative expressions of mRNAs and lncRNA-POIR were normalized by β-actin in qPCR. Data represent mean ± S.D. **P* < 0.05, ***P* < 0.01 and NS, not significant. The scale bar in the micrographs represents 200 nm. Con, control; diff, osteogenic induction; shlncRNA-POIR, plamids for downregulating lncRNA-POIR; shNC, plasmids negative control; NB, new bone; OD, optical density

(Figures 6g and h), which strongly demonstrated that lncRNA-POIR regulated bone formation through *FoxO1*.

Next, we also observed the osteogenic function of *FoxO1* *in vivo* and found that the pPDLSCs in the si*FoxO1* group formed fewer osteoids than those from the negative control groups (Figures 6i and j).

Furthermore, we synthesized a miR-182 inhibitor to identify the osteogenic effects of miR-182. The results of qPCR, western blot analyses, and Alizarin red staining showed that the miR-182 inhibitor promoted the osteogenesis of pPDLSCs (Supplementary Figures S4A–D).

Foxo1 regulates osteogenic differentiation of pPDLSCs through negative regulation of canonical Wnt pathway. To identify the effects of the canonical Wnt pathway on the osteogenesis of pPDLSCs, we treated the pPDLSCs with 50 ng/ml dickkopf-1 (DKK1; a specific inhibitor of the canonical Wnt pathway). After 7 days of osteogenic induction, the DKK1-treated pPDLSCs exhibited an increased osteogenic capacity compared with the control cells (Figures 7a and b).

Next, we assessed whether *FoxO1* inhibits the canonical Wnt pathway and rescues the osteogenesis of pPDLSCs. We found that in the presence of si*FoxO1*, expression of the canonical Wnt pathway targets *cyclin D1*, *Axin* and *c-myc* was significantly upregulated in pPDLSCs (Figures 7c and d). Immunoprecipitation assay revealed that *FoxO1* decreased the amount of *β-catenin* bound to *TCF-4* (Figure 7e). These results indicate that *FoxO1* can impair the binding of *β-catenin* to *TCF-4* without affecting *β-catenin* expression in the total nuclear extracts.

Inhibition of the canonical Wnt pathway via DKK1 completely offsets the negative osteogenic effects of si*FoxO1* (Figures 7f–h), which indicates that *FoxO1* may regulate osteogenic differentiation of pPDLSCs through this pathway.

Overactivation of the NF-κB pathway in inflammation is the main cause of dysregulation of the lncRNA-POIR and miR-182 regulatory network. In this part, we found that the strong upregulation of miR-182 had an important role in suppressing the expression of lncRNA-POIR in pPDLSCs. qPCR revealed that the inhibition of miR-182 reversed the downregulation of lncRNA-POIR expression in the inflammatory environment (Figure 8a). However, the effects of the inflammatory environment on miR-182 upregulation were consistent regardless of whether lncRNA-POIR was inhibited or not (Figure 8b). Western blotting revealed that the NF-κB pathway was activated in pPDLSCs (Figure 8c), and chromatin immunoprecipitation (ChIP) assay showed that *RelA (P65)* and *c-Rel* directly bound to the promoter region of pri-miR-182 (Figure 8d). The binding sites on the promoter region of pri-miR-182 were predicted by JASPAR (http://jaspar.genereg.net/cgi-bin/jaspar_db.pl). These findings indicate that overactivation of the NF-κB pathway is the main cause of dysregulation of the lncRNA-POIR and miR-182 network. To verify this finding, we inhibited the NF-κB

pathway using siIKK*α* (the efficiency of siIKK*α* was determined by qPCR) (Figure 8e) and found that the increase in miR-182 expression was blocked and that lncRNA-POIR expression was increased in the inflammatory microenvironment (Figures 8f and g).

Discussion

Recently, many studies have focused on the functional deficiency of stem cells in several disease conditions. Interestingly, this stem cell dysfunction is always continuous

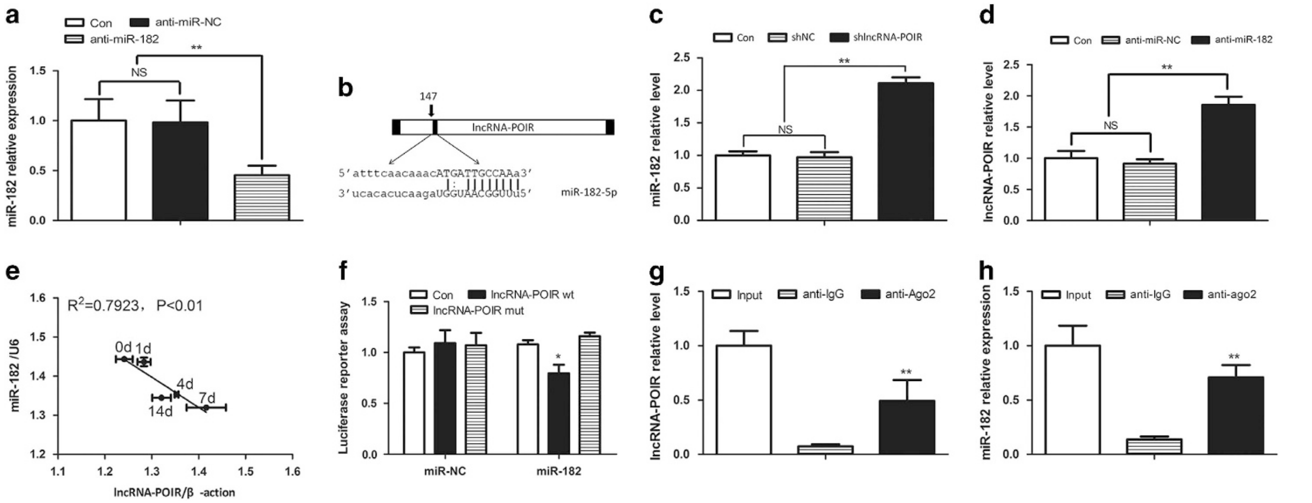

Figure 4 LncRNA-POIR acts as a sponge of miR-182. Besides, lncRNA-POIR and miR-182 could negatively regulate each other. (**a**) Transfection effects of miR-182 inhibitor (anti-miR-182) were determined by qPCR. (**b**) Schematic of the miR-182 putative target site in the lncRNA-POIR. (**c**) After transfection of shlncRNA-POIR in pPDLSCs, the expression of miR-182 was determined by qPCR. (**d**) LncRNA-POIR expression was measured by qPCR in pPDLSCs transfected with anti-miR-182. (**e**) Correlation analysis between lncRNA-POIR levels and miR-182 levels in pPDLSCs 0, 1, 7 and 14 days after osteogenic induction. (**f**) The luciferase reporter assay for the lncRNA-POIR in the presence of miR-182. pPDLSCs were co-transfected with miR-Control or miR-182 and wild-type lncRNA-POIR or mutant lncRNA-POIR. Luciferase constructs values are reported as firefly luciferase activity to *Renilla* luciferase activity. (**g** and **h**) RIP assays were performed using input from cell lysate, normal mouse IgG or anti-Ago2. Relative expression levels of lncRNA-POIR and miR-182 in pPDLSCs were detected by qPCR. All experiments were repeated three times. Relative expressions of lncRNA-POIR and miR-182 were normalized by β-actin and U6 in qPCR, respectively. Data represent mean ± S.D. *$P<0.05$, **$P<0.01$ and NS, not significant. Anti-miR-NC, siPORT reagent alone; anti-miR-182, miR-182 inhibitor; Con, control; lncRNA-POIR wt, lncRNA-POIR wild-type; lncRNA-POIR mut, lncRNA-POIR-mutated type; shlncRNA-POIR, plamids for downregulating lncRNA-POIR; shNC, plasmids negative control

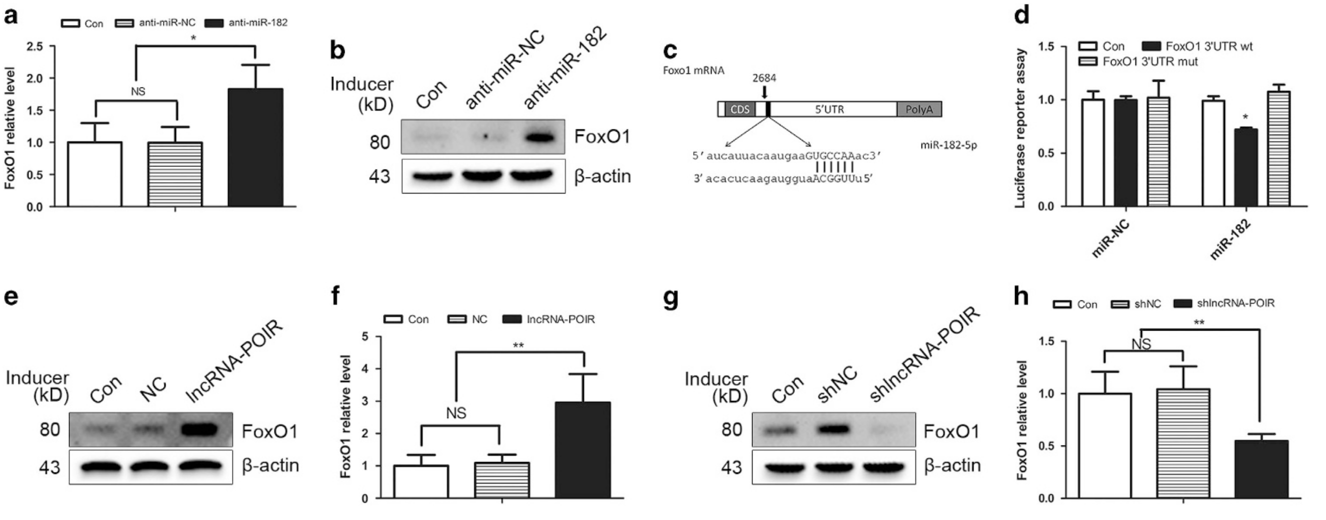

Figure 5 lncRNA-POIR modulated FoxO1 by regulating miR-182 (**a** and **b**) After transfection of anti-miR-182, FoxO1 expression in pPDLSCs was measured by qPCR and western blot. (**c**) Schematic of the miR-182 putative target site in the FoxO1 3′-UTR. (**d**) The luciferase reporter assay for the lncRNA-POIR in the presence of miR-182. PPDLSCs were co-transfected with miR-Control or miR-182 and wild-type FoxO1 3′-UTR or mutant FoxO1 3′-UTR luciferase constructs. Values are reported as firefly luciferase activity to *Renilla* luciferase activity. (**e**–**h**) After transfection of lncRNA-POIR and shlncRNA-POIR in pPDLSCs, FoxO1 expression was measured by qPCR and western blot. All experiments were repeated three times. Relative expressions of FoxO1 were normalized by β-actin in qPCR. Data represent mean ± S.D. *$P<0.05$, **$P<0.01$ and NS, not significant. Anti-miR-NC, siPORT reagent alone; anti-miR-182, miR-182 inhibitor; FoxO1 3′-UTR wt, FoxO1 3′-UTR wild-type; FoxO1 3′-UTR mut, FoxO1 3′-UTR-mutated type, Con, Control; lncRNA-POIR, lentivirus for upregulating lncRNA-POIR; NC, lentivirus negative control; shlncRNA-POIR, plamids for downregulating lncRNA-POIR; shNC, plasmids negative control; UTR, untranslated regions

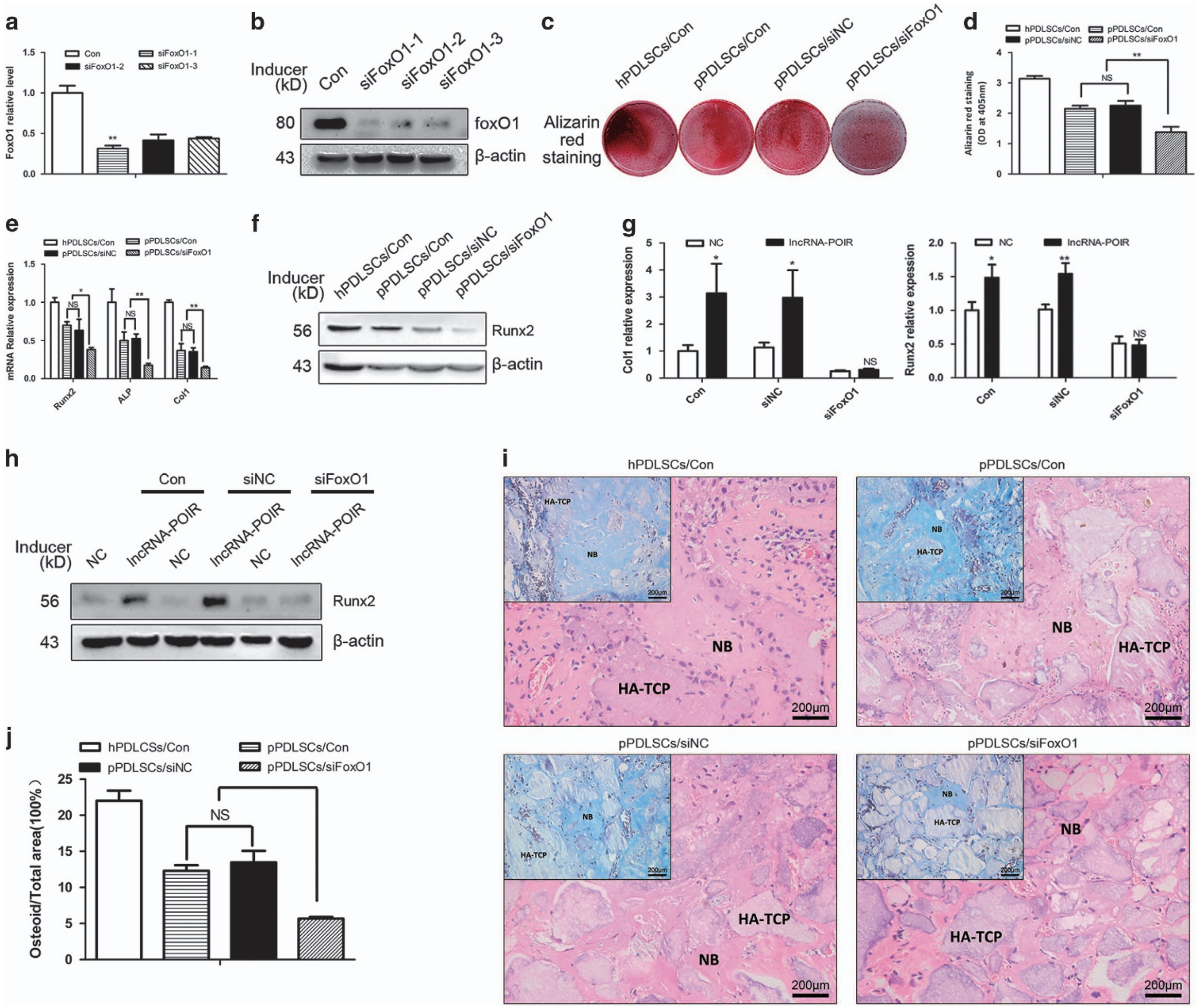

Figure 6 The opposite effects of miR-182 and FoxO1 on osteogenic differentiation. (**a** and **b**) Transfection effects of siFoxO1 was determined by qPCR and western blot. (**c**–**f**) The functions of siFoxO1 on the expression of osteogenic markers of pPDLSCs were measured by Alizarin red staining, qPCR and western blot 7 or 14 days after osteogenic induction. (**g** and **h**) Runx2 and Col1 mRNA levels and Runx2 protein level in pPDLSCs were measured after being transfected with siFoxO1 under the treatment of overexpression of lncRNA-POIR or controls at 7 or 14 days after osteogenic induction. (**i** and **j**) siFoxO1 inhibits osteogenesis of hPDLSCs *in vivo*. hPDLSCs were mixed with HA-TCP and transplanted into the dorsal region of nude mice for 4 weeks. Then, the results were measured by H&E staining and Masson's trichrome staining. Quantitative analysis of the new bone area determined by Image-Pro Plus 6.0 software. At least three fields were randomly selected from each transplant. Six implants were engrafted into three mice per treatment. All experiments were repeated three times. Relative expressions of mRNAs were normalized by β-actin in qPCR. Data represent mean $\pm$ S.D. $^{*}P<0.05$, $^{**}P<0.01$ and NS, not significant. The scale bar in the micrographs represents 200 nm. Con, control; lncRNA-POIR, lentivirus for upregulating lncRNA-POIR; NB, new bone; NC, lentivirus negative control; OD, optical density; siFoxO1, FoxO1 oligo; siNC, negative control

and contributes to the disease pathology.[35,36] Reports have shown that hPDLSCs lose their osteogenic potential in inflammatory microenvironments.[18] Moreover, the function deficiency of pPDLSCs could be long-lasting and irreversible in *ex vivo* expansion without inflammatory supplements. The multidifferentiation potential of pPDLSCs is impaired, even in cells that have been passaged nine times.[37] This impairment might be due to the fact that pPDLSCs secrete *TNF-α* and *IL-1β*, which make cells still subjected to inflammatory microenvironment, after *in vitro* culturing without inflammatory supplements for several weeks.[18] Our results also showed that pPDLSCs that have been passaged seven times still lose their osteogenic ability and secrete cytokines. In fact, these characteristics of pPDLSCs are consistent with hPDLSCs, which were treated with *TNF-α* and *IL-1β*[18] (Supplementary Results and Supplementary Figure S2).

LncRNAs have important roles in numerous developmental pathways, including the maintenance of stem cell pluripotency and the regulation of apoptosis, erythropoiesis and keratinocyte differentiation.[38] As knowledge of lncRNA functions grows, interest in their role in the commitment of MSCs is increasing. Important studies have revealed that lncRNAs

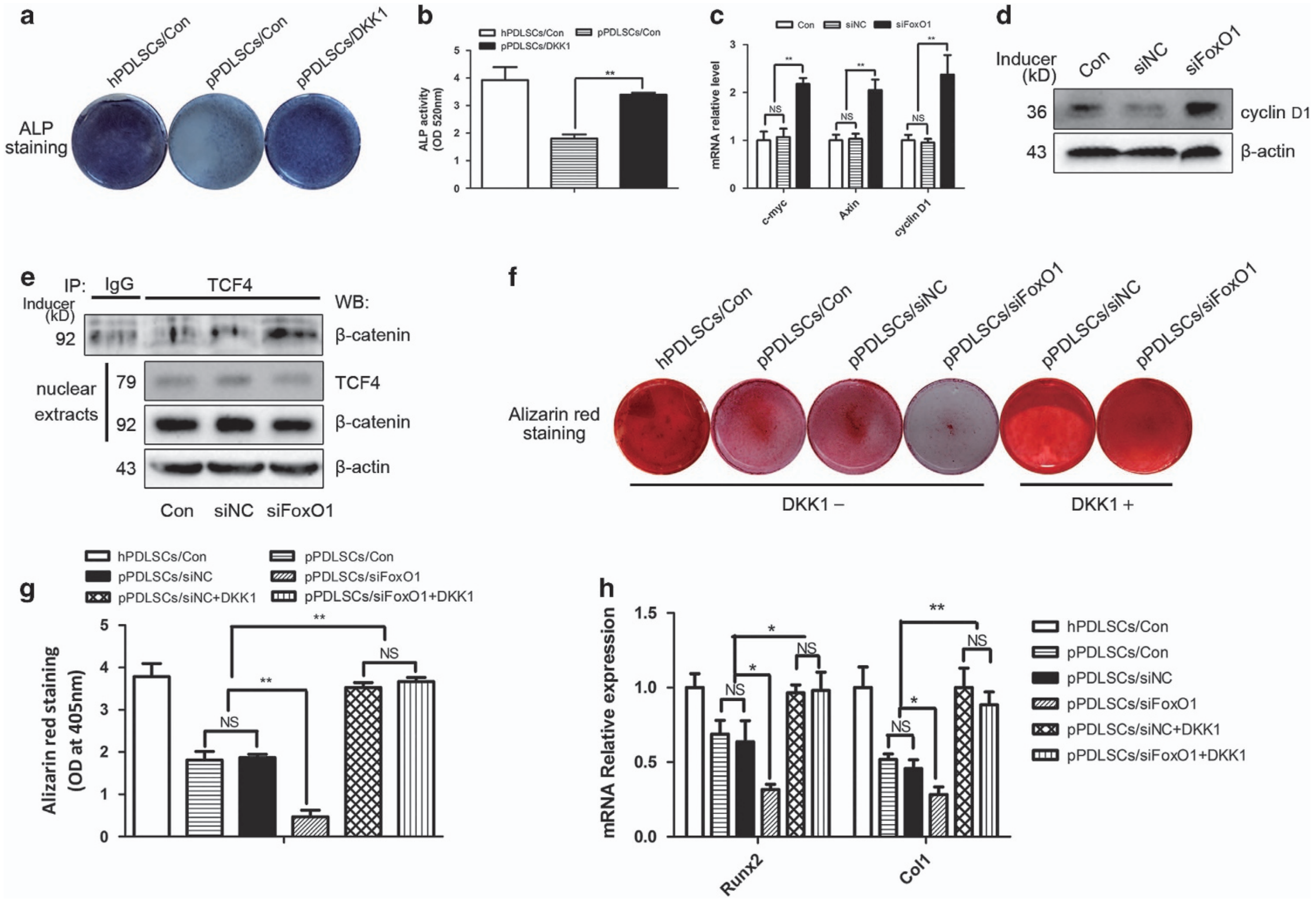

Figure 7 FoxO1 regulate osteogenic differentiation of pPDLSCs through negative regulation of canonical Wnt pathway. (**a** and **b**) ALP staining and ALP activity was detected following 7 days of osteogenic differentiation medium and DKK1 induction. (**c** and **d**) Some targets of canonical Wnt pathway were measured by qPCR and western blot in pPDLSCs after being transfected with siFoxO1. (**e**) pPDLSCs were transfected with siFoxO1, immunoprecipitated with an anti-TCF-4 or anti-IgG antibody and probed with an anti-β-catenin antibody. The TCF-4 or β-catenin expressions in nuclear extracts were also measured. (**f**–**h**) Alizarin Red S and osteogenic gene mRNA levels in pPDLSCs were measured after being transfected with siFoxO1 under the treatment of DKK1 or not at 7 or 14 days after osteogenic induction. All experiments were repeated three times. Relative expressions of mRNAs were normalized by β-actin in qPCR. Data represent mean ± S.D. $^{*}P<0.05$, $^{**}P<0.01$ and NS, not significant. Con, control; DKK1, 50 ng/ml recombinant human DKK1; OD, optical density; siFoxO1, FoxO1 oligo; siNC, negative control

regulate osteogenic capacity under both normal and abnormal conditions.[27,28]

To investigate the role of lncRNAs in the regulation of hPDLSCs osteogenesis in the context of periodontitis, we performed microarray expression profiling of lncRNAs in hPDLSCs and pPDLSCs and confirmed the results by qPCR (Figures 1a and b). The qPCR results showed a similar trend as the microarray results, but there were differences. These differences might be due to differences in either the sample size or the sensitivity of the methods. Therefore, the data from the microarray analysis must be confirmed by qPCR, which is more accurate. (The comparison between the array data and qPCR data could be found in Supplementary Table S2.)

Among the differentially expressed lncRNAs, we chose lncRNA-POIR as a target because its expression was the most significantly altered in pPDLSCs before and after osteogenic induction (Figure 1c) and the relative expression levels of lncRNA-POIR and *Runx2 (Col1)* were strongly correlated ($R>0.8$, $P<0.01$) (Figure 1e). LncRNA-POIR was only recently discovered by lncRNA sequencing technology, and its biological function has not yet been determined. Therefore, to identify the function of lncRNA-POIR, pPDLSCs were constructed with lncRNA-POIR overexpression and knockdown, revealing that lncRNA-POIR promoted bone formation both *in vitro* and *in vivo* (Figure 2). We also observed the effects of lncRNA-POIR in hPDLSCs and found that it affected the osteogenic differentiation of these cells. These results demonstrate that reduced lncRNA-POIR expression in pPDLSCs decreases osteogenic differentiation and that lncRNA-POIR can serve as a target to regulate bone formation in hPDLSCs and pPDLSCs (Figure 3).

LncRNAs, a type of ncRNAs, could regulate their targets in ncRNA networks by interacting with other ncRNAs, similar to miRNAs. In this study, we found that lncRNA-POIR, miR-182 and *FoxO1* were strongly correlated because both lncRNA-POIR and FoxO1 3′-UTR region were found to be targets of miR-182, as determined by bioinformatics analysis and luciferase assay. Interestingly, lncRNA-POIR and miR-182 form an autoregulatory loop by negatively regulating each

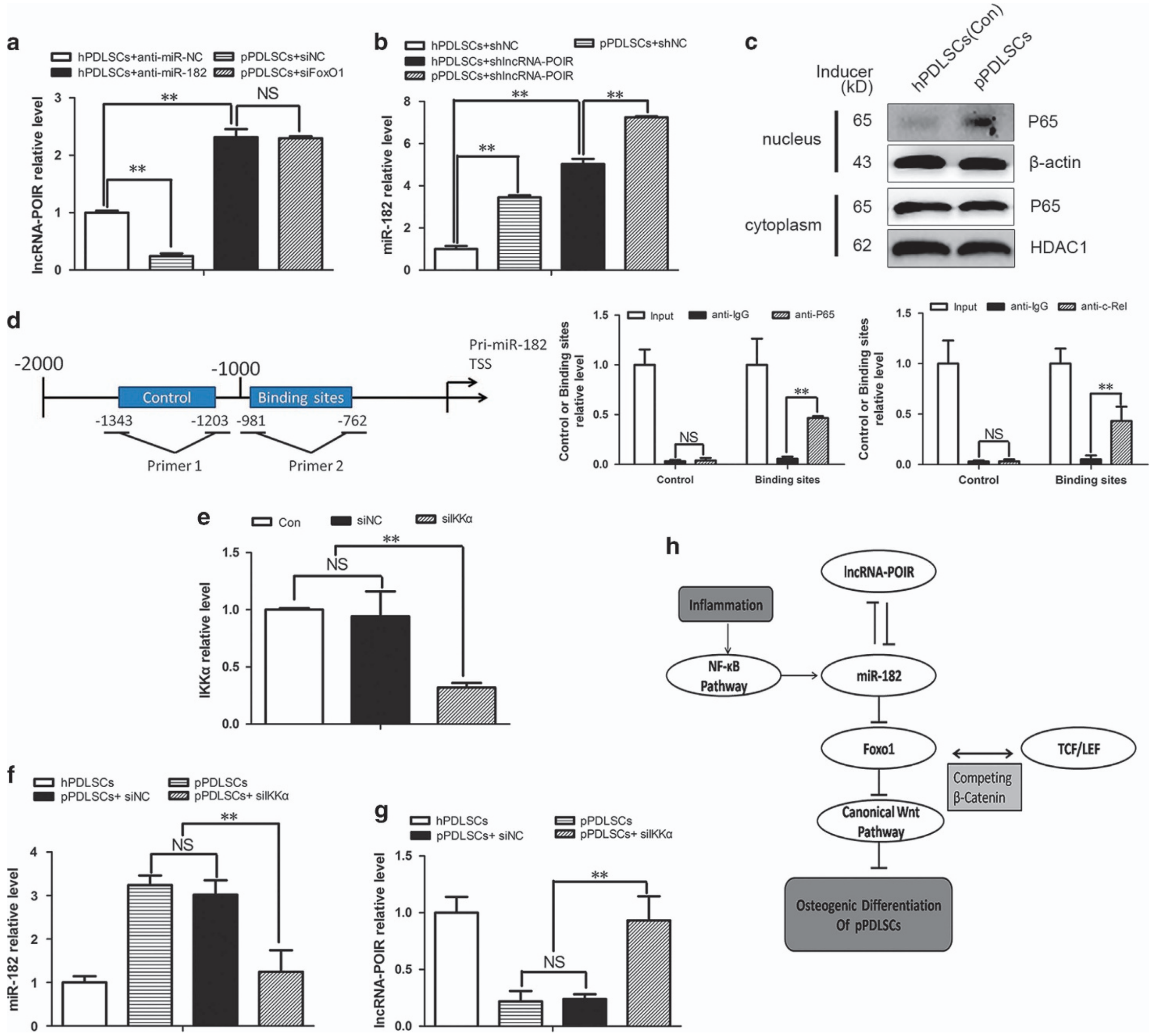

Figure 8 Overactivation of the NF-κB pathway in inflammation is the main cause of dysregulation of the lncRNA-POIR and miR-182 regulatory network. (**a**) After transfection of anti-miR-182, lncRNA-POIR levels in hPDLSCs and pPDLSCs were measured by qPCR. (**b**) After transfection of shlncRNA-POIR, miR-182 levels in hPDLSCs and pPDLSCs were measured by qPCR. (**c**) Western blot was performed to detect the level of P65 in the cytoplasm and nucleus of hPDLSCs and pPDLSCs. β-Actin was used as the control for cytoplasmic P65 and HDAC1 was used as the control for P65 in the nucleus. (**d**) Schematic representation of the human pri-miR-182 promoter region in 2000 bp upstream of the transcription start site (TSS). ChIP assays were performed using input from cell lysate, normal mouse IgG, anti-P65 or anti-c-Rel. Relative expression levels of control and binding regions in pPDLSCs were detected by qPCR. Control: regions without binding sites of P65/c-Rel. Binding regions: regions with several binding sites of P65/c-Rel. (**e**) Transfection effect of siIKKα was determined by qPCR. (**f** and **g**) The pPDLSCs were transfected with IKKα SiRNA for 48 h and qPCR was performed. (**h**) Working model of lncRNA-POIR-miR-182 network in regulating osteogenesis of pPDLSCs. LncRNA-POIR and miR-182 could form a negative regulatory network and lead to a reduction of miR-182 target gene, *FoxO1*, which in turn inhibits canonical Wnt pathway. Besides, inflammation can increase miR-182 expression through the NF-κB pathway and the overexpressed miR-182 in the inflammatory microenvironment resulted in an imbalance in the lncRNA-POIR-miR-182 regulatory network. All experiments were repeated three times. Relative expressions of mRNAs and lncRNA-POIR were normalized by β-actin and relative expression of miR-182 was normalized by U6 in qPCR, respectively. Data represent mean $\pm$ S.D. $^{*}P<0.05$, $^{**}P<0.01$ and NS, not significant. Anti-miR-NC, siPORT reagent alone; anti-miR-182, miR-182 inhibitor; Con, Control; shlncRNA-POIR, plamids for downregulating lncRNA-POIR; shNC, plasmids negative control; siNC, negative control; siIKKα, IKKα oligo

other. This mechanism may have an important role in regulating the biological functions of stem cells. Moreover, the RISC, by which miRNAs recognize their complementary mRNA targets and exert their functions, has an important role in this reciprocal repression.[39–41] In our study, both lncRNA-POIR and miR-182 were found to associate with the RISC by binding to *Ago2*, a core component of the RISC (Figures 4g and h).

FoxO1, as a regulator of the antioxidant process and redox balance, can regulate bone formation in a controversial manner. *FoxO1* has been reported to promote osteogenesis, and its deletion, specifically from osteoblasts and osteoblast

progenitors, has been reported to decrease the osteoblast number, bone formation rate, and bone volume.[42,43] Kim *et al*.[24] have reported that miR-182 negatively regulates osteogenesis by targeting *FoxO1*. Our study revealed similar results in pPDLSCs (Figure 5). Moreover, the positive osteogenic effects of lncRNA-POIR were attenuated by si*FoxO1*, which strongly supports the association of the osteogenic functions of lncRNA-POIR with *FoxO1* (Figure 6).

To further examine the mechanism of *FoxO1* in regulating bone formation in pPDLSCs, we focused on the canonical Wnt pathway, which has a very important role in osteogenesis. The majority of studies have suggested that the canonical Wnt pathway inhibits pPDLSC osteogenic differentiation.[17,18,44] Under inflammatory conditions, the expression of *β-catenin* and its target, *cyclin D1*, are increased, and DKK1, a specific inhibitor of the canonical Wnt pathway, restores the osteogenic capacity of pPDLSCs. Our results showed that DKK1 obviously increased the osteogenic capacity of pPDLSCs, verifying the negative effect of the canonical Wnt pathway on bone formation (Figures 7a and b).

Interestingly, *FoxO1* often acts as a canonical Wnt pathway inhibitor by competing with the *TCF/LEF* complex for *β*-catenin and the expression of *cyclin D1*, a direct downstream target of *β-catenin/TCF*, is inhibited by *FoxO1*.[45–47] Thus, *FoxO1* may regulate osteogenic differentiation by competing with the *TCF/LEF* complex for *β-catenin* and inhibiting the canonical Wnt pathway. Our results are in support of our hypothesis. We found that si*FoxO1* inhibited the expression of the Wnt target genes *cyclin D1*, *c-myc* and *Axin* (Figures 7c and d), and immunoprecipitation analysis demonstrated that *FoxO1* decreased the amount of *β-catenin* bound to *TCF-4* (Figure 7e). These results verify that *FoxO1* regulates the canonical Wnt pathway by competing with *TCF-4* for *β-catenin*. Moreover, we found that the negative osteogenic effect of si*FoxO1* was diminished by DKK1, suggesting that si*FoxO1* inhibited bone formation through regulation of the canonical Wnt pathway (Figures 7f–h). Some studies have reported that *FoxO1* attenuates the differentiation of osteoblasts by inhibiting the canonical Wnt pathway.[45,48] However, the functions of this pathway vary with changes in the microenvironment. Under inflammatory conditions, the canonical Wnt pathway has a negative role in osteogenic differentiation, explaining the *FoxO1*-mediated promotion of bone formation of pPDLSCs, which occurs through regulation of this pathway.

To identify the mechanism that impairs the expression of lncRNA-POIR in pPDLSCs, we found that the strong upregulation of miR-182 had an important role. In the absence of miR-182, the effects of the inflammatory environment on downregulation of lncRNA-POIR expression disappeared (Figure 8a). In inflammation, increased miR-182 caused the low level of lncRNA-POIR expression. Considering lncRNA-POIR and miR-182 often negatively regulate each other, as described above, an abnormally low lncRNA-POIR level may lead to a further increase in miR-182 expression in a vicious circle. These results strongly demonstrate that inflammation may regulate cellar functions through lncRNA-miRNA regulatory network and this mechanism has never been discussed before.

In addition, we observed that miR-182 expression was regulated by the NF-*κ*B pathway. The NF-*κ*B/Rel pathway, which is often activated during inflammation, induces the transcription of downstream genes.[49,50] Chen *et al.*[19] have demonstrated that this pathway is strongly activated in pPDLSCs and that its activation interferes with the bone formation of pPDLSCs. Similarly, our results showed that the NF-*κ*B pathway was upregulated in pPDLSCs (Figure 8c). Further, we found that aberrant activation of this pathway was the key factor leading to miR-182 overexpression during inflammation. This is mainly because *P65* and *P50*, important transcription factors of the NF-*κ*B pathway, directly bind to the promoter regions of pri-miR-182 (Figure 8d). Inhibition of the NF-*κ*B pathway using siIKK*α* resulted in a decrease in the miR-182 level and an increase in the lncRNA-POIR expression in the inflammatory microenvironment (Figures 8f and g). All of these results suggested that the activated NF-*κ*B pathway during inflammation disrupted the balance of the lncRNA-POIR and miR-182 regulatory network.

According to the ceRNA hypothesis, lncRNAs may act as a ceRNA for miRNAs and lead to derepression of target genes.[29] In this process, as targets of miRNAs, lncRNAs are also under the regulation of miRNAs. This lncRNA-miRNA regulatory network has a significant role in mediating cell functions in normal and abnormal conditions.[30–34,40,41] In our study, we indicated an innovative model to depict the core effects of lncRNA-POIR-miR-182 network in osteogenesis of pPDLSCs. In inflammation, this network, which is regulated by the NF-*κ*B pathway in inflammatory microenvironments, may influence the osteogenic differentiation of pPDLSCs through the FoxO1/canonical Wnt pathway (Figure 8h). Our study has provided some new insights into the roles of this lncRNA-miRNA regulatory network in MSC differentiation and provided the new target points and strategies for the periodontitis.

Materials and Methods

Samples and cell cultures. Primary hPDLSC cultures were obtained from 10 individuals, aged 31–40 years, who were undergoing routine premolar extractions for orthodontic reasons or third molar extractions. pPDLSCs were obtained from 10 individuals, aged 27–43 years, who were diagnosed with stable periodontitis and had two-thirds alveolar bone loss and more than one pocket (depth >5 mm). None of these selected subjects showed any clinical evidence of recent infection or systemic disease, and none had a history of smoking or of maxillofacial surgery, radiotherapy, or chemotherapy. All samples were collected at the Dental Clinic of the Fourth Military Medical University. Written informed consent was provided by all participants, and the study was approved by the hospital's ethics committee (license number: IRB-REV-2015038).

The methods used for the culturing and identification of hPDLSCs and pPDLSCs can be found in Supplementary Methods, Results and Supplementary Figure S1.

Microarray and data analysis. A total of six samples (three pPDLSCs and three hPDLSCs from six individuals), which each contained $\sim 1\times10^6$ cells, were prepared, and total RNA was isolated using TRIzol reagent (Invitrogen, Carlsbad, CA, USA). RNA quantity and quality were measured by NanoDrop ND-1000 (Thermo Fisher Scientific, Boston, MA, USA). RNA integrity was assessed by standard denaturing agarose gel electrophoresis. All labeled lncRNAs and mRNAs were hybridized onto an Arraystar Human LncRNA Microarray V3.0 (Arraystar, Kangchen, Shanghai, China), which included 30 586 lncRNAs and 26 109 coding transcripts.

Sample labeling and array hybridization were performed according to the Agilent One-Color Microarray-Based Gene Expression Analysis protocol (Agilent Technologies, Santa Clara, CA, USA) with minor modifications. Briefly, mRNA was purified from total RNA after removal of rRNA (mRNA-ONLY Eukaryotic mRNA Isolation Kit;

Epicentre, San Diego, CA, USA). Then, each sample was amplified and transcribed into fluorescent cRNA along the entire length without 3' bias using a random priming method (Arraystar Flash RNA Labeling Kit; Arraystar, Rockville, MD, USA). The labeled cRNAs were purified with an RNeasy Mini Kit (Qiagen, Hilden, Germany). The concentrations and specific activities of the labeled cRNAs (pmol Cy3 per μg cRNA) were measured using a NanoDrop ND-1000. One microgram of each labeled cRNA was fragmented by adding 5 μl of 10× Blocking Agent and 1 μl of 25× Fragmentation Buffer, and then the mixture was heated to 60 °C for 30 min. Finally, 25 μl of 2×GE Hybridization buffer was added to dilute the labeled cRNA. Fifty microliters of hybridization solution were dispensed onto gasket slides, and lncRNA expression microarrays were assembled. The slides were incubated for 17 h at 65 °C in an Agilent Hybridization Oven (Agilent, Santa Clara, CA, USA). The hybridized arrays were washed, fixed, and scanned using an Agilent DNA Microarray Scanner (part number G2505C).

Agilent Feature Extraction software (version 11.0.1.1) (Agilent, Santa Clara, CA, USA)was used to analyze the acquired array images. Quantile normalization and subsequent data processing were performed using GeneSpring GX v12.1 software package (Agilent Technologies). After quantile normalization of the raw data, lncRNAs and mRNAs that were flagged as Present or Marginal ('All Targets Value') in at least three out of six samples were chosen for further analysis using two-tailed unpaired Student's *t*-test, with adjustments of the *P*-values for multiple testing via Bonferroni correction for *post hoc* analysis.

The data discussed in this publication have been deposited into NCBI's Gene Expression Omnibus and are accessible through the GEO series accession number GSE78074 (https://www.ncbi.nlm.nih.gov/geo/query/acc.cgi?acc= GSE78074).

Mineralization assay. See Supplementary Methods for more details.

Immunoprecipitation. Nuclear proteins were extracted using a Nuclear Extraction Kit (Millipore, Billerica, MA, USA) according to the manufacturer's protocol. To analyze *β-catenin–TCF-4* interactions, cell lysates were immunoprecipitated with a TCF-4 antibody or normal IgG. The immunoprecipitates were resolved by sodium dodecyl sulfate-polyacrylamide gel electrophoresis, and co-immunoprecipitated *β*-catenin was analyzed by western blotting with a *β*-catenin antibody.

Western blot. See Supplementary Methods for details.

qPCR. See Supplementary Methods for details.

Oligonucleotide and cell transfection. Both the miRNA mimics, miRNA inhibitors and siRNAs were synthesized by RiboBio (Guangzhou, China). Oligonucleotide transfection was conducted using Lipofectamine 2000 transfection reagent (Invitrogen) following the manufacturer's recommendations. After 48 h of transfection, the cells were collected and used for further investigations.

Plasmids and lentiviruses. The following plasmids and lentiviruses were designed and constructed by GeneChem (Shanghai GeneChem, Shanghai, China): lentiviruses, pGC-FU-lncRNA-POIR-3FLAG-SV40-EGFP-IRES-puromycin for lncRNA-POIR overexpression and pGC-FU-3FLAG-SV40-EGFP-IRES-puromycin for lncRNA-POIR NC overexpression.

The lncRNA-POIR sequence was amplified using the indicated primers: F: 5′-CCA CCCAATGCTAATGAAG-3′; R: 5′-CGTCGCCGTCCAGCTCGACCAG-3′ and inserted into an SV40 plasmid vector. We used a blank SV40 plasmid vector as an NC. Sequencing was performed to verify the correctness of the sequence. For lentivirus packaging, 293T cells were transferred with an lncRNA-POIR plasmid vector and the packaging plasmids Helper 1.0 and Helper 2.0 (Shanghai GeneChem). The harvested lentiviruses were concentrated, purified and preserved at −80 ºC.

hPDLSCs and pPDLSCs were cultured at a concentration of 2×10^5 cells per well in six-well plates. After the cells had grown to 30–40% confluence, they were transfected with lentiviruses in the presence of polybrene at a multiplicity of transfection of 50.

The sequences of the lncRNA-POIR RNA interference plasmids are as follows: SD11-lncRNA-POIR (4043-1), 5′-TGCTGCACTGCACTGGTAACT-3′; SD11-lncRNA-POIR (4042-2), 5′-CTCCCTCCATGAAGGTTTAAT-3′; SD11-lncRNA-POIR (4044-1), 5′-GACCAGACCAGACCCATAGAA-3′; and SD11-NC, 5′-TTCTCCGAACGTGTCA CGT-3′.

Plasmid transfection was conducted using Lipofectamine 2000 transfection reagent (Invitrogen) following the manufacturer's protocol. After 48 h of transfection, the cells were collected and used for further investigations.

***In vivo* transplantation.** The 6-week-old NOD/SCID mice (Fourth Military Medical University, Xi'an, China) were randomly divided into 12 groups (grouping situation can be found in Supplementary Table S3). Each group included three mice and six transplant complexes (each mouse was transplanted with two complexes in the dorsal region). For a single transplant complex, ~5×10^6 cells were mixed with 20 mg HA-TCP (Sigma, St Louis, MO, USA) and subcutaneously implanted into pockets of NOD/SCID mice in the dorsal region. General anesthesia was administered by intramuscular injection of pentobarbital sodium (0.1 ml/100 g) for all surgical procedures. All animal procedures were approved by the Animal Care Committee of the Fourth Military Medical University (license number: 2015, kq-012). After 4 weeks, the mice were killed by cervical dislocation under general anesthesia. The implants were then removed, fixed with 4% paraformaldehyde and decalcified in 10% EDTA (pH 6.0) for 7 days. For histological analyses, the implants were embedded in paraffin, sectioned and stained with H&E or Masson's Trichrome stain (BaSO Diagnostic Inc., Guangdong, China) according to the manufacturer's instructions.

RNA immunoprecipitation. RIP assay was performed using an RNA Binding Protein Immunoprecipitation Kit (Millipore) according to the manufacturer's instructions with an anti-*Ago2* antibody (2 μg; Millipore) and normal mouse IgG as an NC. qPCR was performed using Taqman Universal PCR Mix as described above. All experiments were repeated three times.

Chromatin immunoprecipitation. ChIP assay was performed with a ChIP Assay Kit (Millipore) according to the manufacturer's instructions with an anti-*P65* antibody (2 μg; Millipore), anti-*c-Rel* antibody (2 μg; Millipore) and normal mouse IgG as an NC. qPCR was performed using Taqman Universal PCR Mix as described above, and the primers used for amplification of the immunoprecipitated DNA are listed in Supplementary Table S1. All experiments were repeated three times.

Luciferase reporter assay. The putative miR-182 target binding sequence in wild-type lncRNA-POIR (lncRNA-POIR wt) and a mutant (lncRNA-POIR mut, with mutation of individual bases in the binding site) were synthesized and cloned downstream of the luciferase gene in pmirGLO luciferase vectors (Promega, Madison, WI, USA).

To determine whether miR-182 directly targets lncRNA-POIR and the *FoxO1* 3′-UTR, we constructed a wild-type *FoxO1* 3′-UTR (FoxO1 3′-UTR wt) reporter plasmid and mutated (FoxO1 3′-UTR mut, with mutation of individual bases in the binding site) *FoxO1* 3′-UTR reporter plasmid with pmirGLO luciferase vectors.

The plasmids, miRNA mimics (miR-NC and miR-182) and *Renilla* luciferase plasmid (Promega, Madison, WI, USA), used as a normalization control, were co-transfected into cells. Firefly and *Renilla* luciferase activities were measured consecutively by Dual Luciferase Assay (Promega) at 48 h after transfection. All experiments were repeated three times.

DKK1 treatment. Cultures in complete medium were treated with 50 ng/ml of a Wnt inhibitor, DKK1 (human recombinant DKK1; PeproTech, Rocky Hill, NJ, USA), and the cultures containing DKK1 were changed every other day. On culture day 7, the cells were harvested and subjected to assays of *in vitro* osteogenic differentiation. ALP activity assay and ALP staining have been described previously.

Statistical analyses. All results are presented as the mean ± S.D. from at least three independent experiments and were analyzed using two-tailed unpaired Student's *t*-test. Pearson's product-moment correlation coefficients (*r*) were used to establish the association of the two variables. For analysis of multiple groups, the *P*-values were adjusted using the Bonferroni correction for *post hoc* analysis. A *P*-value <0.05 was considered statistically significant.

Conflict of Interest

The authors declare no conflict of interest.

Acknowledgements. This work was supported by grants from the National Natural Science Foundation of China (81271176).

1. Seo BM, Miura M, Gronthos S, Bartold PM, Batouli S, Brahim J *et al.* Investigation of multipotent postnatal stem cells from human periodontal ligament. *Lancet* 2004; **364**: 149–155.
2. Akiyama K, Chen C, Gronthos S, Shi S. Lineage differentiation of mesenchymal stem cells from dental pulp, apical papilla, and periodontal ligament. *Methods Mol Biol* 2012; **887**: 111–121.
3. Washio K, Iwata T, Mizutani M, Ando T, Yamato M, Okano T *et al.* Assessment of cell sheets derived from human periodontal ligament cells: a pre-clinical study. *Cell Tissue Res* 2010; **341**: 397–404.
4. Gault P, Black A, Romette JL, Fuente F, Schroeder K, Thillou F *et al.* Tissue-engineered ligament: implant constructs for tooth replacement. *J Clin Periodontol* 2010; **37**: 750–758.
5. Yang H, Gao LN, An Y, Hu CH, Jin F, Zhou J *et al.* Comparison of mesenchymal stem cells derived from gingival tissue and periodontal ligament in different incubation conditions. *Biomaterials* 2013; **34**: 7033–7047.
6. Liu Y, Zheng Y, Ding G, Fang D, Zhang C, Bartold PM *et al.* Periodontal ligament stem cell-mediated treatment for periodontitis in miniature swine. *Stem Cells* 2008; **26**: 1065–1073.
7. Zhu B, Liu W, Zhang H, Zhao X, Duan Y, Li D *et al.* Tissue-specific composite cell aggregates drive periodontium tissue regeneration by reconstructing a regenerative microenvironment. *J Tissue Eng Regen Med* 2015; **9**: 286–295.
8. Aly LA. Stem cells: sources, and regenerative therapies in dental research and practice. *World J Stem Cells* 2015; **7**: 1047–1053.
9. Volponi AA, Gentleman E, Fatscher R, Pang YW, Gentleman MM, Sharpe PT. Composition of mineral produced by dental mesenchymal stem cells. *J Dent Res* 2015; **94**: 1568–1574.
10. Lee J, Pryce BA, Schweitzer R, Ryder MI, Ho SP. Differentiating zones at periodontal ligament-bone and periodontal ligament-cementum entheses. *J Periodontal Res* 2015; **50**: 870–880.
11. Oshima M, Inoue K, Nakajima K, Tachikawa T, Yamazaki H, Isobe T *et al.* Functional tooth restoration by next-generation bio-hybrid implant as a bio-hybrid artificial organ replacement therapy. *Sci Rep* 2014; **4**: 6044.
12. Wu RX, Bi CS, Yu Y, Zhang LL, Chen FM. Age-related decline in the matrix contents and functional properties of human periodontal ligament stem cell sheets. *Acta Biomater* 2015; **22**: 70–82.
13. Zhang J, An Y, Gao LN, Zhang YJ, Jin Y, Chen FM. The effect of aging on the pluripotential capacity and regenerative potential of human periodontal ligament stem cells. *Biomaterials* 2012; **33**: 6974–6986.
14. Lossdörfer S, Kraus D, Jäger A. Aging affects the phenotypic characteristics of human periodontal ligament cells and the cellular response to hormonal stimulation *in vitro*. *J Periodontal Res* 2010; **45**: 764–771.
15. Zheng W, Wang S, Ma D, Tang L, Duan Y, Jin Y. Loss of proliferation and differentiation capacity of aged human periodontal ligament stem cells and rejuvenation by exposure to the young extrinsic environment. *Tissue Eng A* 2009; **15**: 2363–2371.
16. Zhou Y, Fan W, Xiao Y. The effect of hypoxia on the stemness and differentiation capacity of PDLC and DPC. *BioMed Res Int* 2014: 890675.
17. Liu W, Konermann A, Guo T, Jäger A, Zhang L, Jin Y. Canonical Wnt signaling differently modulates osteogenic differentiation of mesenchymal stem cells derived from bone marrow and from periodontal ligament under inflammatory conditions. *Biochim Biophys Acta* 2014; **1840**: 1125–1134.
18. Liu N, Shi S, Deng M, Tang L, Zhang G, Liu N *et al.* High levels of beta-catenin signaling reduce osteogenic differentiation of stem cells in inflammatory microenvironments through inhibition of the noncanonical Wnt pathway. *J Bone Miner Res* 2011; **26**: 2082–2095.
19. Chen X, Hu C, Wang G, Li L, Kong X, Ding Y *et al.* Nuclear factor-κB modulates osteogenesis of periodontal ligament stem cells through competition with β-catenin signaling in inflammatory microenvironments. *Cell Death Dis* 2013; **4**: e510.
20. Mattick JS. RNA regulation: a new genetics? *Nat Rev Genet* 2004; **5**: 316–323.
21. Bartel DP. MicroRNAs: target recognition and regulatory functions. *Cell* 2009; **136**: 215–233.
22. Li Z, Hassan MQ, Jafferji M, Aqeilan RI, Garzon R, Croce CM *et al.* Biological functions of miR-29b contribute to positive regulation of osteoblast differentiation. *J Biol Chem* 2009; **284**: 15676–15684.
23. Zhang Y, Xie RL, Croce CM, Stein JL, Lian JB, van Wijnen AJ *et al.* A program of microRNAs controls osteogenic lineage progression by targeting transcription factor Runx2. *Proc Natl Acad Sci USA* 2011; **108**: 9863–9868.
24. Kim KM, Park SJ, Jung SH, Kim EJ, Jogeswar G, Ajita J *et al.* miR-182 is a negative regulator of osteoblast proliferation, differentiation, and skeletogenesis through targeting FoxO1. *J Bone Miner Res* 2012; **27**: 1669–1679.
25. Batista PJ, Chang HY. Long noncoding RNAs: cellular address codes in development and disease. *Cell* 2013; **152**: 1298–1307.
26. Zhuang W, Ge X, Yang S, Huang M, Zhuang W, Chen P *et al.* Upregulation of lncRNA MEG3 promotes osteogenic differentiation of mesenchymal stem cells from multiple myeloma patients by targeting BMP4 transcription. *Stem Cells* 2015; **33**: 1985–1997.
27. Huang Y, Zheng Y, Jia L, Li W. Long noncoding RNA H19 promotes osteoblast differentiation via TGF-b1/Smad3/HDAC signaling pathway by deriving miR-675. *Stem Cells* 2015; **33**: 3481–3492.
28. Zhu L, Xu PC. Downregulated LncRNA-ANCR promotes osteoblast differentiation by targeting EZH2 and regulating Runx2 expression. *Biochem Biophys Res Commun* 2013; **432**: 612–617.
29. Fan C, Hao Z, Yan J, Li G. Genome-wide identification and functional analysis of lincRNAs acting as miRNA targets or decoys in maize. *BMC Genomics* 2015; **16**: 793.
30. Cai H, Xue Y, Wang P, Wang Z, Li Z, Hu Y *et al.* The long noncoding RNA TUG1 regulates blood–tumor barrier permeability by targeting miR-144. *Oncotarget* 2015; **6**: 19759–19779.
31. Xing CY, Hu XQ, Xie FY, Yu ZJ, Li HY, Wu JB *et al.* Long non-coding RNA HOTAIR modulates c-KIT expression through sponging miR-193a in acute myeloid leukemia. *FEBS Lett* 2015; **589**: 1981–1987.
32. Deng L, Yang SB, Xu FF, Zhang JH. Long noncoding RNA CCAT1 promotes hepatocellular carcinoma progression by functioning as let-7 sponge. *J Exp Clin Cancer Res* 2015; **34**: 18.
33. Yoon JH, Abdelmohsen K, Kim J, Yang X, Martindale JL, Tominaga-Yamanaka K *et al.* Scaffold function of long noncoding RNA HOTAIR in protein ubiquitination. *Nat Commun* 2013; **4**: 2939.
34. Cesana M, Cacchiarelli D, Legnini I, Santini T, Sthandier O, Chinappi M *et al.* A long noncoding RNA controls muscle differentiation by functioning as a competing endogenous RNA. *Cell* 2011; **147**: 358–369.
35. Sun LY, Zhang HY, Feng XB, Hou YY, Lu LW, Fan LM. Abnormality of bone marrow-derived mesenchymal stem cells in patients with systemic lupus erythematosus. *Lupus* 2007; **16**: 121–128.
36. Sun L, Akiyama K, Zhang H, Yamaza T, Hou Y, Zhao S *et al.* Mesenchymal stem cell transplantation reverses multiorgan dysfunction in systemic lupus erythematosus mice and humans. *Stem Cells* 2009; **27**: 1421–1432.
37. Liu Y, Liu W, Hu C, Xue Z, Wang G, Ding B *et al.* MiR-17 modulates osteogenic differentiation through a coherent feed-forward loop in mesenchymal stem cells isolated from periodontal ligaments of patients with periodontitis. *Stem Cells* 2011; **29**: 1804–1816.
38. Khalil AM, Guttman M, Huarte M, Garber M, Raj A, Rivea Morales D *et al.* Many human large intergenic noncoding RNAs associate with chromatin-modifying complexes and affect gene expression. *Proc Natl Acad Sci USA* 2009; **106**: 11667–11672.
39. Kallen AN, Zhou XB, Xu J, Qiao C, Ma J, Yan L *et al.* The imprinted H19 lncRNA antagonizes let-7 microRNAs. *Mol Cell* 2013; **52**: 101–112.
40. Liu XH, Sun M, Nie FQ, Ge YB, Zhang EB, Yin DD *et al.* LncRNA HOTAIR functions as a competing endogenous RNA to regulate HER2 expression by sponging miR-331-3p in gastric cancer. *Mol Cancer* 2014; **13**: 92.
41. Liang WC, Fu WM, Wong CW, Wang Y, Wang WM, Hu GX *et al.* The lncRNA H19 promotes epithelial to mesenchymal transition by functioning as miRNA sponges in colorectal cancer. *Oncotarget* 2015; **26**: 22513–22525.
42. Ambrogini E, Almeida M, Martin-Millan M, Paik JH, Depinho RA, Han L *et al.* FoxO-mediated defense against oxidative stress in osteoblasts is indispensable for skeletal homeostasis in mice. *Cell Metab* 2010; **11**: 136–146.
43. Rached MT, Kode A, Xu L, Yoshikawa Y, Paik JH, Depinho RA *et al.* FoxO1 is a positive regulator of bone formation by favoring protein synthesis and resistance to oxidative stress in osteoblasts. *Cell Metab* 2010; **11**: 147–160.
44. Kong X, Liu Y, Ye R, Zhu B, Zhu Y, Liu X *et al.* GSK3β is a checkpoint for TNF-α-mediated impaired osteogenic differentiation of mesenchymal stem cells in inflammatory microenvironments. *Biochim Biophys Acta* 2013; **1830**: 5119–5129.
45. Iyer S, Ambrogini E, Bartell SM, Han L, Roberson PK, de Cabo R *et al.* FOXOs attenuate bone formation by suppressing Wnt signaling. *J Clin Invest* 2013; **123**: 3409–3419.
46. Guan H, Tan P, Xie L, Mi B, Fang Z, Li J *et al.* FOXO1 inhibits osteosarcoma oncogenesis via Wnt/beta-catenin pathway suppression. *Oncogenesis* 2015; **4**: e166.
47. Ip W, Shao W, Song Z, Chen Z, Wheeler MB, Jin T. Liver-specific expression of dominant-negative transcription factor 7-like 2 causes progressive impairment in glucose homeostasis. *Diabetes* 2015; **64**: 1923–1932.
48. Almeida M, Han L, Martin-Millan M, O'Brien CA, Manolagas SC. Oxidative stress antagonizes Wnt signaling in osteoblast precursors by diverting beta-catenin from T cell factor to forkhead box O-mediated transcription. *J Biol Chem* 2007; **282**: 27298–27305.
49. Tak PP, Firestein GS. NF-kappaB: a key role in inflammatory diseases. *J Clin Invest* 2001; **107**: 7–11.
50. Lawrence T, Gilroy DW, Colville-Nash PR, Willoughby DA. Possible new role for NF-kappaB in the resolution of inflammation. *Nat Med* 2001; **7**: 1291–1297.

Cell Death and Disease **is an open-access journal published by *Nature Publishing Group*. This work is licensed under a Creative Commons Attribution 4.0 International License. The images or other third party material in this article are included in the article's Creative Commons license, unless indicated otherwise in the credit line; if the material is not included under the Creative Commons license, users will need to obtain permission from the license holder to reproduce the material. To view a copy of this license, visit http://creativecommons.org/licenses/by/4.0/**

© The Author(s) 2016

Supplementary Information accompanies this paper on Cell Death and Disease website (http://www.nature.com/cddis)
