# Supplementary material for: Molecular Regulators for the Wnt/β-Catenin Signalling Pathway for Bone Remodelling in Periodontitis: A Scoping Review
Source: Int J Mol Sci. 2026 Oct 14;27(20):9117. doi: 10.3390/ijms27209117 (PMC13705578; doi:10.3390/ijms27209117)
Supplement: Supplementary file 1 [file ijms-27-09117-s001.zip › Supplementary Data/Papers/in vitro/Notoginsenoside R1 Promotes Proliferation and Osteogenic Differentiation of hPDLSCs via Wnt -Catenin Signaling Pathway.pdf]

Dovepress
DRUG DESIGN, DEVELOPMENT AND THERAPY
OPEN ACCESS

Taylor & Francis
Taylor & Francis Group

Drug Design, Development and Therapy

ISSN: 1177-8881 (Online) Journal homepage: www.tandfonline.com/journals/dddt20

Notoginsenoside R1 Promotes Proliferation and Osteogenic Differentiation of hPDLSCs via Wnt/β-Catenin Signaling Pathway

Ruiqi Han, Wenjuan Zhang, Lina Zhang, Jinghua Zou, Yanran Yang, Hongkun Li & Jun Zhang

To cite this article: Ruiqi Han, Wenjuan Zhang, Lina Zhang, Jinghua Zou, Yanran Yang, Hongkun Li & Jun Zhang (2022) Notoginsenoside R1 Promotes Proliferation and Osteogenic Differentiation of hPDLSCs via Wnt/β-Catenin Signaling Pathway, Drug Design, Development and Therapy, , 4399-4409, DOI: 10.2147/DDDT.S387004

To link to this article: https://doi.org/10.2147/DDDT.S387004

© 2022 Han et al.

Published online: 05 Dec 2023.

Submit your article to this journal

Article views: 332

View related articles

View Crossmark data

Citing articles: 8 View citing articles

Full Terms & Conditions of access and use can be found at
https://www.tandfonline.com/action/journalInformation?journalCode=dddt20

Drug Design, Development and Therapy

Dovepress
open access to scientific and medical research

Open Access Full Text Article

ORIGINAL RESEARCH

Notoginsenoside R1 Promotes Proliferation and Osteogenic Differentiation of hPDLSCs via Wnt/β-Catenin Signaling Pathway

Ruiqi Han[1], Wenjuan Zhang[2], Lina Zhang[3], Jinghua Zou[1], Yanran Yang[1], Hongkun Li[1], Jun Zhang[1]

[1]Shandong University & Shandong Key Laboratory of Oral Tissue Regeneration & Shandong Engineering Laboratory for Dental Materials and Oral Tissue Regeneration, Department of Orthodontics, School and Hospital of Stomatology, Cheeloo College of Medicine, Shandong University, Jinan, People's Republic of China; [2]Department of Orthodontics, Affiliated Hospital of Shandong University of Traditional Chinese Medicine, Jinan, People's Republic of China; [3]Department of Orthodontics, Faculty of Stomatology, Liaocheng People's Hospital, Liaocheng, People's Republic of China

Correspondence: Jun Zhang, Shandong University & Shandong Key Laboratory of Oral Tissue Regeneration & Shandong Engineering Laboratory for Dental Materials and Oral Tissue Regeneration, Department of Orthodontics, School and Hospital of Stomatology, Cheeloo College of Medicine, Shandong University, No. 44-1 Wenhua Road West, Jinan, 250012, Tel +86 13953109816, Email zhangj@sdu.edu.cn

Purpose: To investigate the roles of Notoginsenoside R1 (NG-R1) on the proliferation and osteogenic differentiation of human periodontal ligament stem cells (hPDLSCs) and explore its possible mechanism.

Methods: hPDLSCs were isolated and, then characterized by flow cytometry. Cell-counting kit-8 (CCK-8) and colony assays were used to validate the effect of different NG-R1 concentrations on hPDLSCs proliferation and the optimal concentration was determined. Quantitative detection of alkaline phosphatase (ALP) activity at optimal concentration and the mineralization of the cells was investigated by Alizarin Red S staining. qRT-PCR and Western blot were utilized to examine the factors expression levels of ALP, Runx Family Transcription Factor 2 (RUNX2), Collagen I (Col-1) and catenin beta 1 (CTNNB1; β-catenin). In addition, the tankyrase inhibitor XAV-939 was used to explore NG-R1's role in canonical Wnt signaling.

Results: hPDLSCs were positive for surface antigens CD90 while negative for CD34 and CD45, which indicated that we have successfully isolated the hPDLSCs. Furthermore, a concentration of 20μmol NG-R1 dramatically enhanced hPDLSCs proliferation, ALP activity, and mineral deposition. ALP, RUNX2, COL-1, and β-catenin expression were all rised in comparison to control group. After XAV-939 was added to disrupt the canonical Wnt signaling, the impact of NG-R1 appeared to be reversed.

Conclusion: These findings suggest that NG-R1 can stimulate osteogenic differentiation of hPDLSCs, which is probably attributable to canonical Wnt signaling activation.

Keywords: notoginsenoside R1, NG-R1, human periodontal ligament stem cells, hPDLSCs, osteogenesis, β-catenin

Introduction

Periodontitis occurs in approximately 45%-50% of individuals.[1] It is characterized by inflammation caused by microbial and host interactions, which eventually leads to the loss of periodontal attachment.[2] If not intervened, it will lead to the progressive deterioration of gum tissue, periodontal ligaments, and periodontal support tissues, ultimately leading to the loss of teeth. In addition, studies have proved that periodontitis is closely related to systemic diseases such as chronic kidney disease,[3] diabetes,[4] and cerebrovascular disease.[5] Existing clinical treatment techniques, such as periodontal cleaning and scraping of root leveling, can prevent only further development of inflammation but cannot promote the regeneration of periodontitis tissue that has been destroyed. The most ideal treatment for periodontitis is to regenerate periodontal tissue. Periodontal tissue regeneration refers to the surgical or tissue engineering restoration of damaged periodontal tissue.[6] The development of stem cells provides a powerful background for the regeneration of periodontal tissue. hPDLSCs are multidirectionally differentiable, self-renewing stem cells,[7] it can differentiate into fibroblasts, osteoblasts, and cementoblasts.[8] It's the primary source of cells that produce new attachments following periodontitis therapy. Hence, hPDLSCs have broad potential in periodontal tissue regeneration.

Received: 29 August 2022
Accepted: 15 December 2022
Published: 23 December 2022

© 2022 Han et al. This work is published and licensed by Dove Medical Press Limited. The full terms of this license are available at https://www.dovepress.com/terms.php and incorporate the Creative Commons Attribution – Non Commercial (unported, v3.0) License (http://creativecommons.org/licenses/by-nc/3.0/). By accessing the work you hereby accept the Terms. Non-commercial uses of the work are permitted without any further permission from Dove Medical Press Limited, provided the work is properly attributed. For permission for commercial use of this work, please see paragraphs 4.2 and 5 of our Terms (https://www.dovepress.com/terms.php).

Panax notoginseng has a lengthy history as a hemostatic agent,[9] it is also used to treat cardiovascular diseases,[10] and chronic hepatitis.[11] Furthermore, Panax notoginseng has antioxidant, anti-inflammatory, and neuroprotective properties.[9] NG-R1 is a compound isolated from Panax notoginseng that has estrogenic activity.[12] Studies have shown that NG-R1 could relax pulmonary blood arteries, thereby alleviating pulmonary hypertension.[13] In vitro experiments have demonstrated that NG-R1 can promote osteogenesis, which is beneficial postmenopausal ladies who have osteoporosis.[14] However, the effect of NG-R1 on hPDLSCs has not been reported.

Studies have shown that NG-R1 can promote various stem cells differentiation through multiple signaling pathways. Wang et al[15] has demonstrated that NG-R1 can facilitate MC3T3-E1 differentiation through the MAPK and JAK1/STAT3 signaling. In vitro studies have also proven that NG-R1 can promote osteogenic differentiation of rat primary osteoblasts through estrogen receptor signaling.[14] Li et al[16] demonstrated that NG-R1 can reduce the dysfunction of osteoblasts under oxidative stress by blocking the JNK signaling pathway. Wnt signaling pathways cascade controls a vast range of biological processes in all animals during development and adulthood,[17] it is crucial for maintaining the homeostasis of human bones.[18] Numerous studies have found that various traditional Chinese medications enhance osteogenesis of hPDLSCs via the canonical Wnt signaling, which is dependent of β-catenin.[19–21] As a result, we hypothesized that NG-R1 could improve hPDLSCs osteogenic differentiation by regulating the canonical Wnt signaling.

The purposes of this work were to explore whether NG-R1 could affect the proliferation and differentiation of hPDLSCs, and to research its possible mechanism. We hypothesized that this process was related to the canonical Wnt pathway. Also, we hypothesized that this experiment might yield valuable reference data for clinical applications of NG-R1 and hPDLSCs in tissue engineering.

Method

hPDLSCs Cultivation and Identification

This study was conducted in accordance with the Declaration of Helsinki. Approval was given by the Ethics committee of Shandong University Protocol (Protocol No. 20210122). The parental/legal guardians of those involved were made aware of the purpose of the study and supplied consent to participate on their children's behalf. After obtaining consent, premolars that were extracted between the ages of 14–18 years for orthodontic purposes without dental caries or periodontal disease. We placed the centrifuge tube on ice with the pre-cooled α-minimum essential medium (α-MEM; Hyclone, Logan, UT, USA) containing 5% penicillin/streptomycin (Hyclone; GEHealthcare Life Sciences, Logan, UT, USA), and rapidly putted the extracted teeth into the centrifuge tube and transferred it to the laboratory. After discarding the centrifuge tube's medium and gently rinsing the tooth root with phosphate buffered saline (PBS) containing 5% penicillin/streptomycin antibiotics, scraping the periodontal membrane from the middle part of tooth root, and placing the scraped membrane in the culture flask, adding 6mL complete medium including 20% fetal bovine serum (FBS; Biological Industries, Israel) to the culture flask, and keeping the tissue block from attaching successfully in an incubator for about 4 hours, then turning the bottle so that the tissue block is completely immersed in the culture medium. Spindle-shaped hPDLSCs started to emerge from the tissue block's edge after about a week. When the cells had occupied about 80% of the bottle's bottom, rinsing it with PBS and adding 1.5mL of 0.25% trypsin-EDTA solution (Thermo Fisher Scientific Inc) to digest the cells. The flask should be placed in the incubator for 2 minutes to react, then we observed under the microscope until the cells are spherical and floating, added 4mL of complete medium to stop the digestion. Collected the cell suspension in a centrifuge tube, after centrifugation, discard the supernatant, and added 1.5mL of complete medium to resuspend the cell suspension, and re-inoculated in a large plate at a ratio of 1:3. The hPDLSCs were collected and utilized in subsequent experiments between passages 3 and 5. Flow cytometry was used to evaluate stem cell characteristics by detecting fluorescent isothiocyanate (FITC) labeled CD34, CD45, and CD90 (Beckman Coulter, Franklin Lakes, NJ, USA).[20]

Multidirectional Differentiation Assays

To verify multi-directional differentiation capacity, hPDLSCs were assayed by osteogenesis and adipogenic experiments.[21] hPDLSCs were introduced by osteogenic inducing medium (α-MEM containing 10% FBS, 50μg/mL

ascorbic acid, 10mM β-glycerophosphate, and 0.01μM dexamethasone) (Sigma-Aldrich) per well, and after 21 days of osteogenesis induction, it was discarded. Cells were fixed with 4% paraformaldehyde after being rinsed in PBS, and then stained with Alizarin Red-S while being observed under a microscope, scanned, and photographed.

For adipogenic induction experiments, cells were cultured in adipogenic medium (α -MEM supplemented with 500mM isobutyl-methylxanthine, 0.5 M hydrocorti-sone, 60mM indomethacin, 10 mM insulin, and 10% FBS) (Sigma-Aldrich) for 21 days. Removed the induction media after 21 days of adipogenic induction, then washed the dish with PBS, when the cells were fixed, added Oil Red O (Cyagen Bio-sciences, Guangzhou, China) for staining.

CCK-8 Assay

The effect of NG-R1 on hPDLSCs proliferation was examined by CCK-8 experiment,[20] after routine digestion and centrifugation of cells were resuspended, 2000 cells were added to each well in the 96-well plate. After cell adhesion, the culture medium was discarded, 0, 5, 20, 40, 50μmol NG-R1 (Solarbio, Beijing, China) were added respectively, five duplicate wells were measured. On the 1,3 and 5 days of culture, the solution was discarded, and 100μL of CCK-8 working solution (CCK-8; Dojindo Laboratories, Kumamoto, Japan) was added to each well. After two hours of incubation in dark room, the absorbance was measured using a microplate reader.

Colony Formation

After cell digestion and centrifugation, 500 cells were implanted in each well, 2mL of complete medium was added to each well for adherent culture, after the cell adhesion,2mL of 0, 5, 20, 40μmol NG-R1 were added to the six-well plate, after 10 days of culture, the plate was washed three times with PBS, 4% paraformaldehyde was added for cell fixation, the cell fixative solution was discarded and crystal violet (Solarbio)was added for staining.

ALP Activity Measure and Staining Assay

Cells were cultured with various concentrations of NG-R1 (0, 5, 20, 40, 50μmol). After cultured for 7 or 14 days, added the cell lysate (RIPA: PMSF=99:1)(Solarbio, Beijing, China). Following 15 minutes of lysis on ice, the lysate was collected and centrifuged, and the supernatants were extracted to obtain the protein.[22] ALP activity was measured by alkaline phosphatase assay kit (Solarbio, Beijing, China).

We used bicinchoninic acid (BCA) kits to detect protein concentrations. A spectrophotometer (SPECTRAstar, Nano, BMG Labtech, Ortenberg, Germany) was applied to evaluate absorbance at 520nm wavelength.

In order to observe staining, a BCIP/NBT Alkaline Phosphatase Color Development Kit (Beyotime, Shanghai, China) was used, with the intensity of dark blue representing ALP activity.[23]

Alizarin Red S Staining

After 21 days of incubation, we rinsed the plates three times with PBS, and added 2mL of 4% paraformaldehyde per well for cell fixation, and then added Alizarin Red S for staining. After 30 minutes, discarded the dye and added PBS to the plate to rinse it five times. Examining the cells under a microscope, scanning, and photographing them.

The mineral nodules were subsequently dissolved using 10% cetylpyridinium chloride (CPC; Solarbio).[22] The OD_{562} value was determined using a microplate reader to identify the amount of mineralized matrix deposition.

Real-Time PCR

Following osteogenic induction for 7 and 14 days with different concentration of NG-R1 (0 or 20μmol), total RNA was extracted with Trizol (Takara) and reverse transcriptase(Takara) generated cDNA.[21]

qRT-PCR was carried out using Takara's SYBR® Premix Ex TaqTM II and the Roche Light Cycler® 480 Sequence Detection System (Roche Diagnostics GmbH, Mannheim, Germany). Each reaction was repeated three times. The primer sequences used in the present study were as follows: ALP:

5′-GGCGGTGAACGAGAGAATGT-3′and 5′- GGACGTAGTTCTGCTCGTGG-3′; RUNX2:

5′-GGAGTGGACGAGGCAAGAGT-3′ and 5′- AGGCGGTCAGAGAACAAACT-3′; CTNNB1:

5′-GCTGCAACTAAACAGGAAGGG-3′ and 5′- CCCACTTGGCAGACCATCAT −3′;

COL-1:5′-TAAAGGGTCACCGTGGCTTC-3′and 5′-GGGAGACCGTTGAGTCCATC-3′;

GAPDH: 5′-GCACCGTCAAGGCTGAGAAC-3′ and 5′-TGGTGAAGACGCCAGTGGA-3′. The data were analyzed using the $2^{-\Delta\Delta Ct}$ method. The data were averaged after each examination was carried out in three duplicates.

Western Blot

After cultivated with NG-R1 (0,20μmol) for 7 days, cells were lysed for 15 minutes on ice, we centrifuged the samples and then extracted the supernatant to get the protein, and the protein concentration was quantified by using a BCA kit. The protein samples (20μg) were separated by 10% sodium dodecyl sulfate-polyacrylamide gel electrophoresis (SDS-PAGE) and transferred onto a polyvinylidene fluoride (PVDF) membrane, and the PVDF membranes were blocked for 1 hour at room temperature with 5% skim milk. The incubation box was placed in 4°C overnight after selecting appropriate sites to incubated with primary antibodies based on the molecular weight of GAPDH, ALP, RUNX2, COL-1, β-catenin, GSK3β, P-GSK3β (Ser9), and LEF (Abcam, Cambridge, UK).[22] The membranes were washed for 30 minutes by using Tris-buffered saline with 0.1% Tween 20 (TBST) (Solarbio, Beijing, China) and then the secondary antibody was incubated for one hour. We used a chemiluminescent substrate kit obtained from Millipore to create the protein blots.

XAV-939 Treatments

XAV-939 is a proven inhibitor of canonical Wnt pathway, thereby stabilizing Axin and increasing the degradation of β-catenin as a result.[25,26] In this study, hPDLSCs were cultured in four different solutions in 6 well plates as follows: (i)osteogenic induction group was seen as the control group.(ii)osteogenic induction medium+2.5×10^{-6} M XAV-939 group was utilized as a confirmation group for the XAV-939 action.(iii)osteogenic induction medium+20μmol NG-R1 group. (iv) osteogenic induction medium+20μmol NG-R1+2.5×10^{-6} M XAV-939 group. The protein expression was evaluated using the Western blot method after being induced for 7 days.

Statistical Analysis

All experiments were repeated three times. The data were normally distributed and presented as the means ± standard deviations. Statistical analysis was conducted using

GraphPad Prism 6 (GraphPad Software, Inc, La Jolla, CA, USA). Differences were analyzed by the Student's *t*-test or one-way analysis of variance (ANOVA), and $P < 0.05$ was considered statistically significant.

Result

hPDLSCs Cultivation and Identification

After the cells were cultured for approximately 1 week, the primary cells begin to crawl out of the edges of the tissue block. For the tests that followed, we utilized cells from the passage 3 to 5 (Figure 1A) There were red mineralized nodules formed after osteogenesis induction (Figure 1B) and lipid droplets formed after lipogenesis induction (Figure 1C), which were evidence that hPDLSCs had multidirectional differentiation ability. We also found that the hPDLSCs were positive for CD90 expression (Figure 1D), whereas hematopoietic-related surface chronicles CD34 and CD45 were negative (Figure 1E and F).

Proliferation of Cells

On days 1, 3, and 5, the effect of various NG-R1 concentrations (0, 5, 20, 40, or 50μmol) on the proliferation of hPDLSCs was assessed. Though cell proliferation did not significantly differ between the first and third days, 20μmol significantly promoted cell proliferation on day 5, while 50μmol markedly inhibited it (Figure 2A).

The cloning experiment results proved that the colonies formed by 20μmol group were larger and more numerous than other groups (Figure 2B and C).

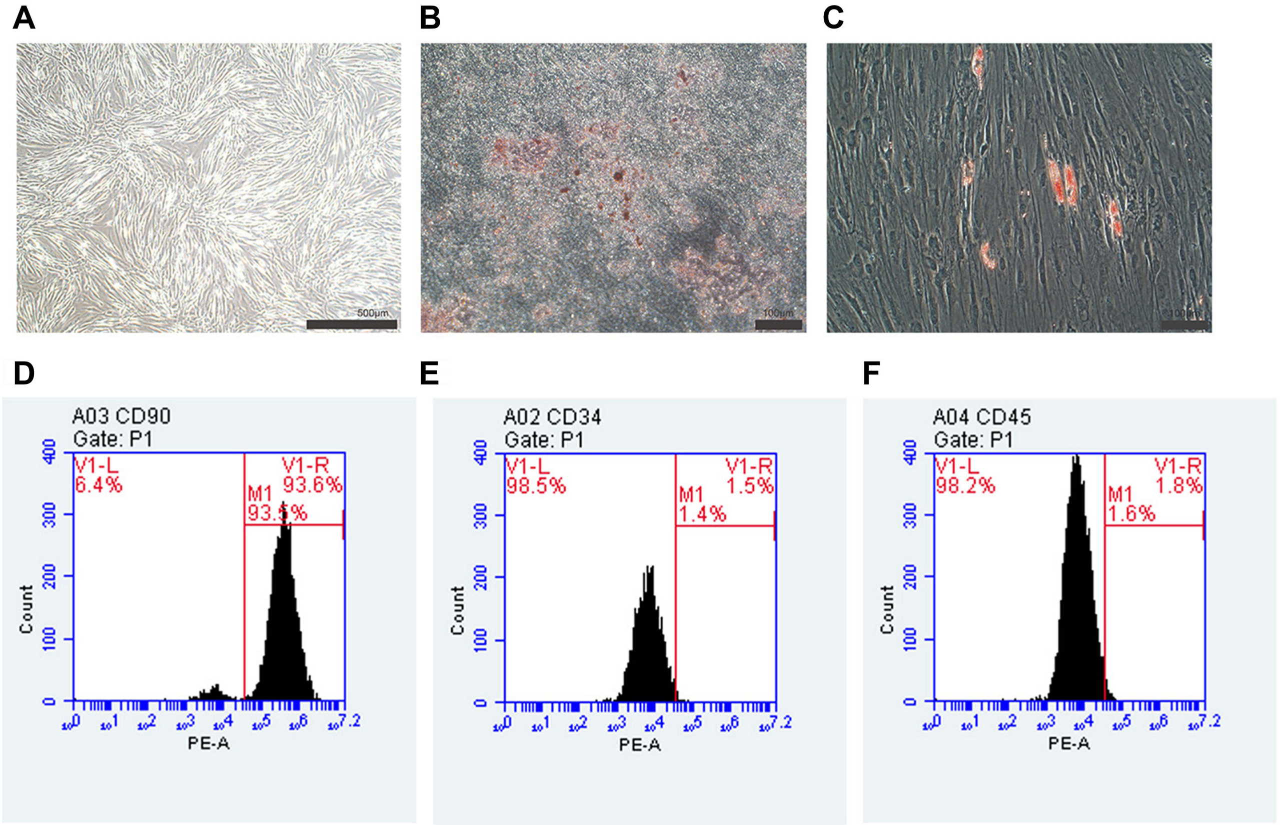

Figure 1 Cultivation and characterization of hPDLSCs. (**A**) hPDLSCs arranged in a swirling pattern after subculturation. Scale bar: 500μm. (**B**) Mineralized nodules formed after osteogenesis induction. Scale bars: 100μm. (**C**) Lipid droplets formed after lipogenesis induction. Scale bars: 100μm. The expression levels of CD90 (**D**), CD34 (**E**), and CD45 (**F**) were determined by flow cytometry.

ALP Activity and Mineralized Nodule Formation are Impacted by NG-R1

The ALP activity experiment showed that on days 7 and 14, the ALP activity of the 20μmol NG-R1 treated hPDLSCs was increased compared with other groups (Figure 3A). ALP staining showed the same trend (Figure 3B). From the results of CCK8 and ALP activity, it can be concluded that 20μmol NG-R1 had a distinguished effect on hPDLSC proliferation, and we therefore selected 20μmol for follow-up experiments.

The Alizarin Red positive nodules indicated that the 20μmol NG-R1 group dramatically increased the deposition of mineralized nodules by hPDLSCs (Figure 3C and D).

NG-R1 Promoted the Osteogenic-Related Factors Expression

After 7 and 14 days cultivation by osteogenesis induction including 20μmol NG-R1, the expression of elements associated to osteogenesis ALP, RUNX2, COL-1 and β-catenin were detected by qRT-PCR, so as to verify the effect of NG-R1 on hPDLSCs osteogenesis at the molecular level. The results of the experiment showed that the expression of osteogenic related factors in the NG-R1 treatment group was increased, and the increase was more obvious on day 14 than day 7(Figure 4A-D).

To shorten the cycle of the experiment, 7 days osteogenesis-induced cells were used in the subsequent experiments in order to perform Western blot. The results revealed that the expression of osteogenesis-related protein increased in NG-R1group (Figure 4E and F), which was consistent with the results of qRT-PCR.

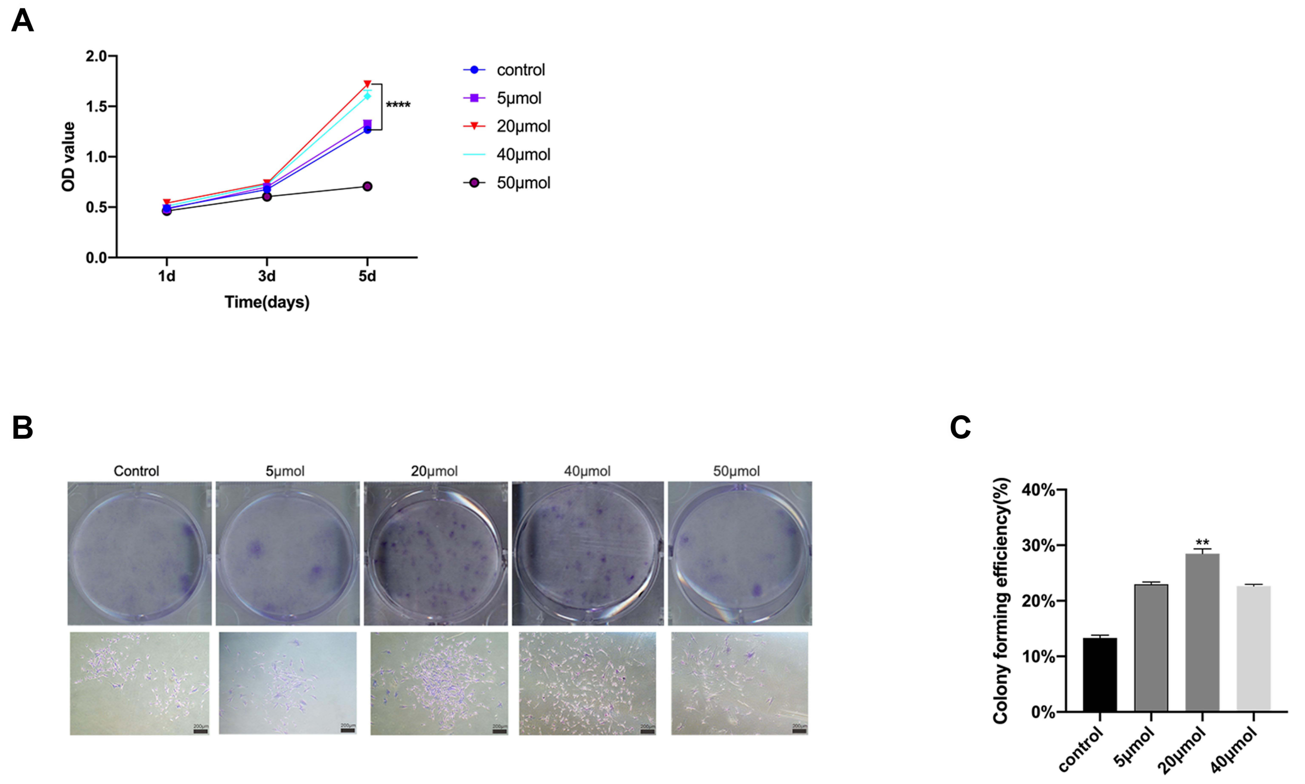

Figure 2 Influences of NG-R1 on hPDLSC proliferation. (**A**) Effects of different concentrations of NG-R1 on hPDLSCs. (**B**) Colonies formed by 20μmol group were larger and more numerous than other groups. (**C**) The rate of clonal formation increased considerably in the 20μmol group. (ANOVA, n = 3). The columns represent the means. Error bars represent standard deviations. (** P < 0.01 ****P < 0.0001).

NG-R1 Can Activate the Canonical Wnt Signaling

The experiments proved NG-R1 could promote osteogenesis of hPDLSCs, and we further investigated its possible mechanism. The experimental results proved that the addition of 20μmol of NG-R1 can significantly increase the expression of osteogenic proteins including ALP, RUNX2, COL-1 and signaling pathway-associated proteins including β-catenin, P-GSK3β, and LEF while there was no significant variance in GSK-3β expression between the four groups, in addition, after the addition of XAV-939, the expression of the above proteins decreased significantly (Figure 5A-H), indicating that the canonical Wnt signaling has been successfully inhibited. Interestingly, after adding 20μmol of NG-R1 and XAV-939 at the same time, NG-R1 can partially reverse the blocking of canonical Wnt signaling by XAV-939 (Figure 5A-H), which is manifested by an increase in the expression level of the protein compared with the XAV-939 group. The above results prove that NG-R1 can promote osteogenesis of hPDLSCs by activating the canonical Wnt signaling.

Discussion

hPDLSCs are multidirectionally differentiable, self-renewing stem cells. They are cells with a high potential for tissue regeneration. hPDLSCs have been shown to differentiate into osteoblasts and cementoblasts, which form alveolar bone and cementum.[24] Furthermore, the hPDLSCs can also differentiate into chondroblast-like cells.[25] So, discovering drugs that promote periodontal tissue regeneration is critical.

In this experiment, hPDLSCs were isolated and then passaged. Flow cytometry showed that the MSC–related surface marker CD90 was positive, while hematopoiesis-related markers CD34 and CD45 were negative, which proved that the hPDLSCs were derived from mesenchyme. In vitro osteogenic and adipogenic induction of hPDLSCs into osteoblasts and adipocytes, as well as colony formation experiments, proved their multidirectional differentiation and colony formation ability.

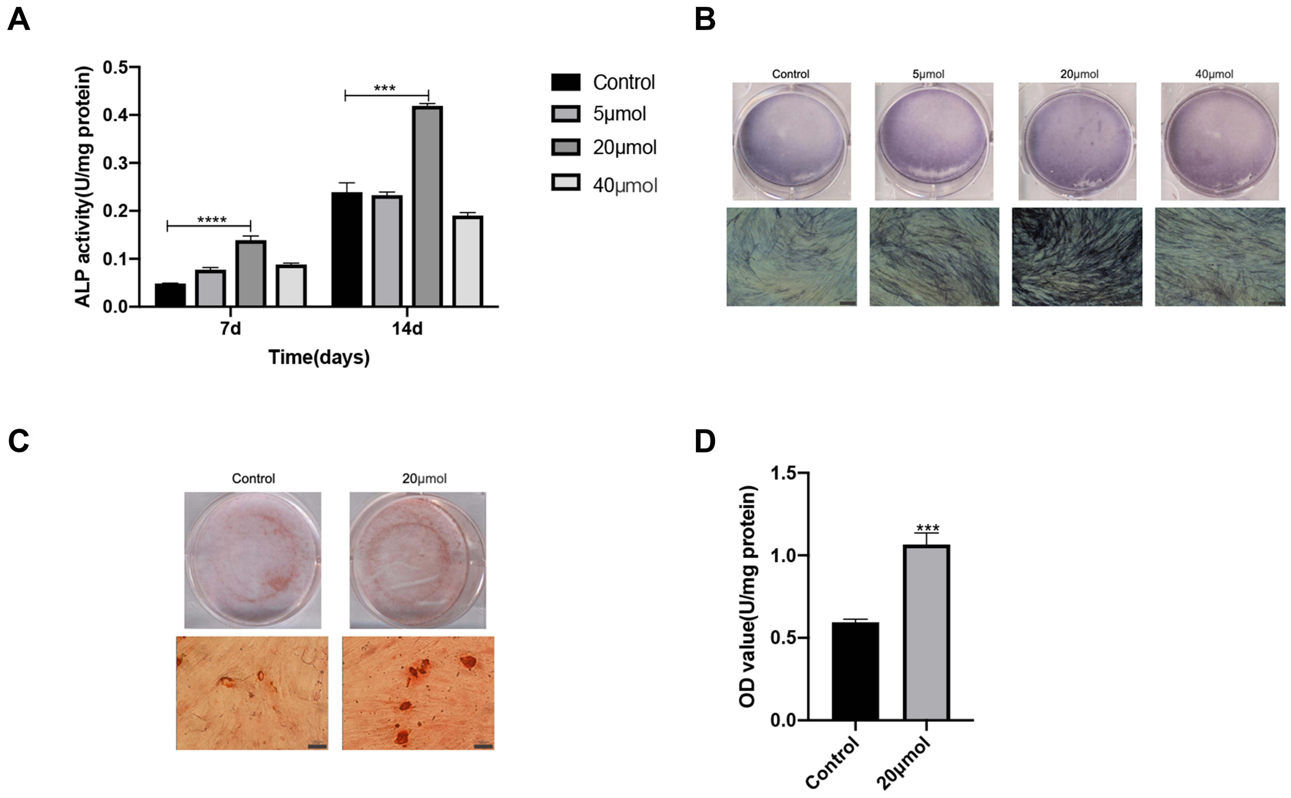

Figure 3 Effects of NG-R1 on ALP activity and mineralized nodule deposition of hPDLSCs(**A**) ALP activity under different concentrations of NG-R1 treatment (ANOVA, n = 3). (**B**)ALP staining under different concentrations of NG-R1 treatment. Scale bar: 100 μm.(**C**) Formation of mineralized nodules after alizarin red staining. Scale bar: 100μm. (**D**) Mineralized nodule formation was quantitatively analyzed at OD_{562} (ANOVA, n = 3). The columns represent the means. Error bars represent standard deviations. (***P < 0.001**** P < 0.0001).

Panax notoginseng is a traditional Chinese herbal medicine, it's is a hemostatic drug with a long history,[26] and has also been used to treat cardiovascular diseases and tumors.[10,27] Saponin is the main component of P. notoginseng.[28] The saponins in Panax notoginseng mainly include Ginsenosides Rg1, Rb1, and NG- R1.[29] Studies have shown that NG-R1 can significantly promote MC3T3-E1 osteogenesis in vitro and also has an anti-osteoporotic effect.[30] Other experiments have shown that NG-R1, through its estrogenic properties, enhances osteoblast differentiation and mineralization, while inhibiting osteoclast-mediated bone resorption.[14] These findings indicate that NG-R1 has the potential to promote bone regeneration. As a result, it is critical to investigate the effect of NG-R1 on the hPDLSCs' ability to proliferate and differentiate, since this can result in the creation of novel medications to support periodontal tissue regeneration.

ALP is considered a precursor to osteogenesis, our experimental results showed that the ALP activity in the 20μmol NG-R1 group was significantly increased. Mineralization can be detected via Alizarin Red S staining, the formation of mineralized nodules was larger and more numerous in the 20μmol NG-R1-treated group, and the Alizarin red staining was deeper in this group. According to the results above, the cells' level of mineralization has increased following treatment with 20μmol NG-R1. The CCK-8 and ALP staining assay demonstrated that 20μmol NG-R1 had the strongest stimulating effect on the proliferation and mineralization of hPDLSCs, Moreover, the results of the cloning formation experiment are consistent with the above conclusions. Therefore, 20μmol NG-R1 was selected for subsequent experiments.

Four osteogenic factors (ALP, RUNX2, Col-1, and β-catenin) were chosen for this study. GAPDH was selected as a standard reference. ALP is a hallmark of early osteogenesis and it's also a hallmark of osteoblast maturation.[31] RUNX2 is a key factor in osteogenesis. Col-1 is a marker of osteogenesis and collagen formation.[32] β-catenin plays a key role in embryogenesis and adulthood.[33] In this experiment, the effect of NG-R1 on hPDLSCs osteogenesis at the molecular level was explored through qRT-PCR and Western blot. The results of qRT-PCR showed that the expression of the above osteogenesis-related genes in NG-R1 group increased significantly, and the Western blot experimental results showed the

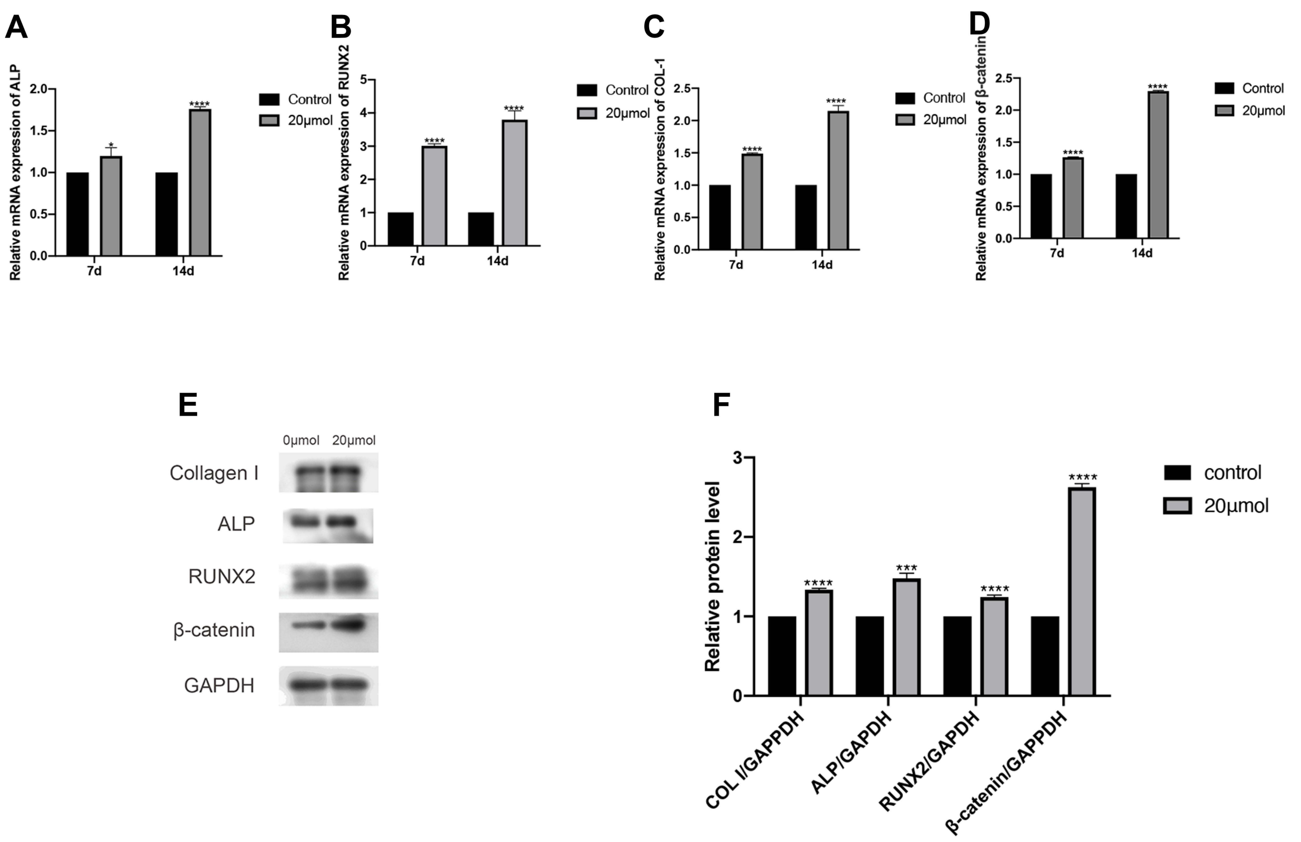

Figure 4 Expression of osteogenesis associated genes ALP (**A**), RUNX2 (**B**), COL-1 (**C**) and β-catenin (**D**)were all enhanced by 20μmol NG-R1 treated. (ANOVA, n = 3). Expression of osteogenesis associated genes detected by Western blot analysis were all enhanced by 20μmol NG-R1 treated (**E** and **F**) (ANOVA, n = 3). The columns represent the means. Error bars represent standard deviations. (*P < 0.05,***P < 0.001, ****P < 0.0001).

same trend, from which we concluded that NG-R1 can affect the osteogenesis ability of hPDLSCs through the regulation of a series of osteogenic related factors.

The Wnt signaling pathway plays an important role in many processes in the body, and this pathway also plays a key role in the function of osteoblasts.[34] The Wnt signaling pathway is divided into two parts: the canonical Wnt signaling pathway (Wnt/β-catenin pathway)and the non- canonical Wnt signaling pathway.[34] According to studies, an increase in fat formation frequently coincides with a decrease in bone formation.[35] Regulation of osteoblast and adipoblast differentiation is largely controlled by the Wnt/β-catenin pathway.[36] The stability of β-catenin is the key to the Wnt/β-catenin pathway. When a Wnt signal is present, it binds to the frizzled receptor on the cell membrane, which transmits the signal to Dishevelled (Dvl) in the cytoplasm and activates it. Activated Dishevelled (Dvl) has the ability to inhibit the activity of the complex formed by Axin, adenomatous polyposiscoli (APC), and GSK3β, thereby preventing β-catenin from being phosphorylated by GSK3β. As only phosphorylated β-catenin can be degraded, unphosphorylated β-catenin accumulates in the cytoplasm, the accumulated β-catenin binds to the transcription factor TCF/LEF, activating TCF's transcriptional activity and regulating the expression of target genes. According to research, phosphorylation of the Ser9 site decreases the action of GSK3β. As a result, the level of P-GSK3β is primarily used to assess GSK3β activity.

XAV-939 can stabilize Axin and subsequently promote β-catenin degradation to block the Wnt signaling pathway.[37] Therefore, in this experiment, we chose it to inhibit the canonical Wnt signaling, and then explored the mechanism of NG-R1 on hPDLSCs proliferation and osteogenesis. According to the experiment's findings, XAV-939 treatment dramatically lowered the expression of pathway-related proteins, which showed that XAV-939 has inhibited the Wnt signaling pathway. At the same time, compared with the NG-R1 group, the expression of osteogenesis-related proteins was reduced in the XAV-939 group, which indicated the osteogenic capacity of NG-R1 is impaired when the Wnt signaling pathway was blocked. In addition, NG-R1+ XAV-939 had a decreased protein expression level compared with the NG-R1 group, this

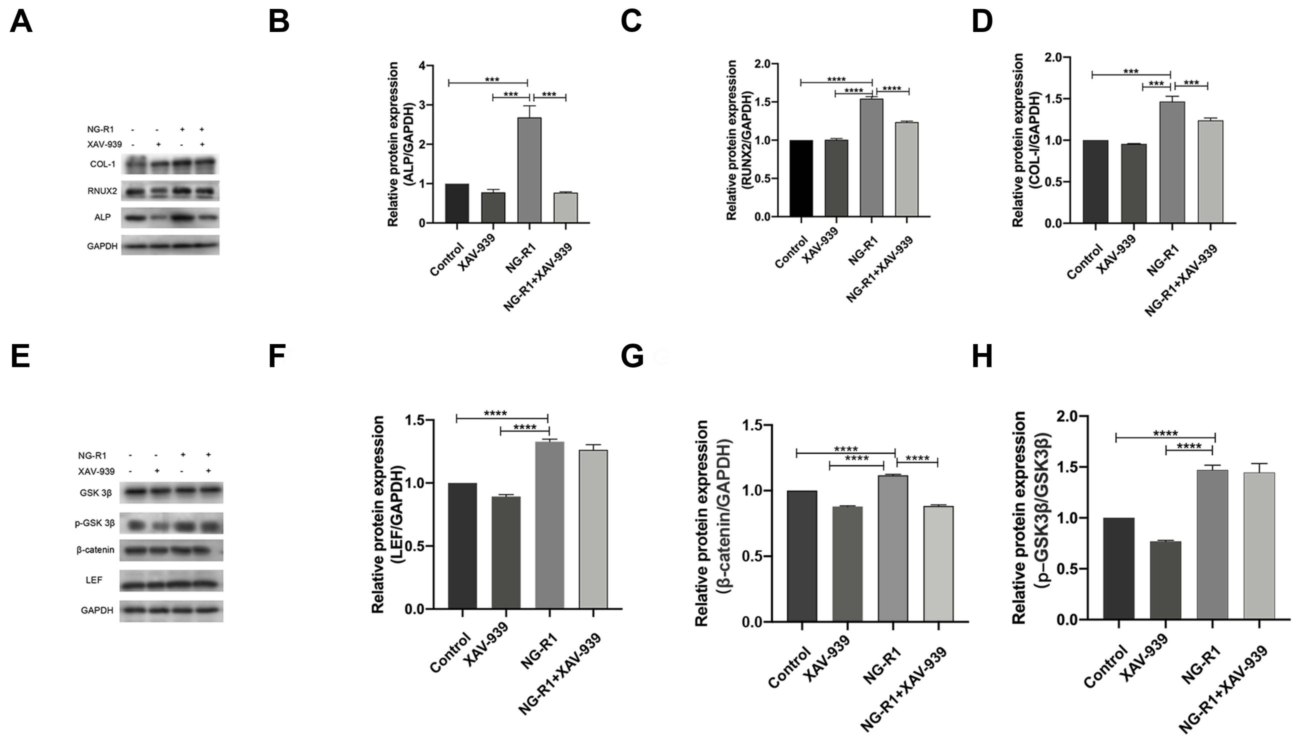

Figure 5 The effects of XAV-939 on NG-R1 treated osteogenic promotion of hPDLSCs. NG-R1 boosted osteogenesis and pathway-related genes expression substantially, whereas XAV-939 inhibited the expression of these genes. (**A-H**) (ANOVA, n = 3). The columns represent the means. Error bars represent standard deviations. (***P < 0.001, ****P < 0.0001).

suggests that the inhibition of the pathway by XAV-939 partially weakened the promotion of osteogenesis by NG-R1. In summary, NG-R1 can promote osteogenesis of hPDLSCs by activating the canonical Wnt signaling.

However, when we looked at the protein expression levels of the NG-R1+XAV-939 group, we discovered that XAV-939 only partially reduced the effect of NG-R1. This suggests that there are other possible mechanisms by which NG-R1 promotes hPDLSCs osteogenesis. For instance, it was claimed that NG-R1 could promote rat primary osteoblasts through estrogen receptor signaling.[14] In future studies, we will explore the precise mechanism by which NG-R1 affects hPDLSCs and investigate its effect on osteogenesis in vivo.

Acknowledgments

This work was supported by the Natural Science Foundation of Shandong Province, China (No. ZR2021QH340).

Disclosure

The authors have no conflicts of interest relevant to this article.

References

1. Genco RJ, Sanz M. Clinical and public health implications of periodontal and systemic diseases: an overview. *Periodontology*. 2020;83(1):7–13. doi:10.1111/prd.12344
2. Tonetti MS, Greenwell H, Kornman KS. Staging and grading of periodontitis: framework and proposal of a new classification and case definition. *J Clin Periodontol*. 2018;45:S149–S161. doi:10.1111/jcpe.12945
3. Ma ZJ, Zhang JZ. Changes in serum zinc level of periodontitis with kidney deficiency. *Chine J Integrated Traditional Western Med*. 1993;13(10):606.
4. Preshaw PM, Bissett SM. Periodontitis and diabetes. *Br Dent J*. 2019;227(7):577–584. doi:10.1038/s41415-019-0794-5
5. Tonetti MS, Van Dyke TE. Periodontitis and atherosclerotic cardiovascular disease: consensus report of the Joint EFP/AAP Workshop on Periodontitis and Systemic Diseases. *J Clin Periodontol*. 2013;40:S24–S29. doi:10.1111/jcpe.12089
6. Carmagnola D, Tarce M, Dellavia C, Rimondini L, Varoni EM. Engineered scaffolds and cell-based therapy for periodontal regeneration. *J Appl Biomater Funct Mater*. 2017;15(4):E303–E312. doi:10.5301/jabfm.5000389

7. Ying M, Zhang B. Daidzein promotes the proliferation and osteogenic differentiation of periodontal ligament stem cell. *Oral Dis*. 2022. doi:10.1111/odi.14113
8. Lekic PC, Nayak BN, Al-Sanea R, Tenenbaum H, Ganss B, McCulloch C. Cell transplantation in wounded mixed connective tissues. *Anatomical Record Part Discoveries Mol Cell Evolutionary Biol*. 2005;287A(2):1256–1263. doi:10.1002/ar.a.20242
9. Wang T, Guo RX, Zhou GH, et al. Traditional uses, botany, phytochemistry, pharmacology and toxicology of Panax notoginseng (Burk.) FH Chen: a review. *J Ethnopharmacol*. 2016;188:234–258. doi:10.1016/j.jep.2016.05.005
10. Zhao G-R, Xiang Z-J, Ye T-X, Yuan Y-J, Guo Z-X. Antioxidant activities of Salvia miltiorrhiza and Panax notoginseng. *Food Chem*. 2006;99 (4):767–774. doi:10.1016/j.foodchem.2005.09.002
11. Park WH, Lee SK, Kim CH. A Korean herbal medicine, Panax notoginseng, prevents liver fibrosis and hepatic microvascular dysfunction in rats. *Life Sci*. 2005;76(15):1675–1690. doi:10.1016/j.lfs.2004.07.030
12. Sun B, Xiao J. Notoginsenoside R1 attenuates cardiac dysfunction in endotoxemic mice: an insight into oestrogen receptor activation and PI3K/Akt signalling. *Br J Pharmacol*. 2013;168(7):1758–1770. doi:10.1111/bph.12063
13. Hu Y. Vasodilation of notoginsenoside R1 on pulmonary arteries of pulmonary hypertensive rats. *Chine Pharmacological Bulletin*. 2013;29(11):1572–1576.
14. Wang T, Wan D, Shao L, Dai J, Jiang C. Notoginsenoside R1 stimulates osteogenic function in primary osteoblasts via estrogen receptor signaling. *Biochem Biophys Res Commun*. 2015;466(2):232–239. doi:10.1016/j.bbrc.2015.09.014
15. Wang C, Sun H, Zhong Y. Notoginsenoside R1 promotes MC3T3-E1 differentiation by up-regulating miR-23a via MAPK and JAK1/STAT3 pathways. *Artif Cells Nanomed Biotechnol*. 2019;47(1):603–609. doi:10.1080/21691401.2019.1573189
16. Li X, Lin H, Zhang X, et al. Notoginsenoside R1 attenuates oxidative stress-induced osteoblast dysfunction through JNK signalling pathway. *J Cell Mol Med*. 2021;25(24):11278–11289. doi:10.1111/jcmm.17054
17. Nusse R. Wnt signaling in disease and in development. *Cell Res*. 2005;15(1):28–32. doi:10.1038/sj.cr.7290260
18. Baron R, Kneissel M. WNT signaling in bone homeostasis and disease: from human mutations to treatments. *Nat Med*. 2013;19(2):179–192. doi:10.1038/nm.3074
19. Zhang L-N, Wang -X-X, Wang Z, Li K-Y. Berberine improves advanced glycation end products-induced osteogenic differentiation responses in human periodontal ligament stem cells through the canonical Wnt/beta-catenin pathway. *Mol Med Rep*. 2019;19(6):5440–5452. doi:10.3892/mmr.2019.10193
20. Zheng D-H, Wang -X-X, Ma D, Zhang L-N, Qiao Q-F, Zhang J. Erythropoietin enhances osteogenic differentiation of human periodontal ligament stem cells via Wnt/beta-catenin signaling pathway. *Drug Des Devel Ther*. 2019;13:2543–2552. doi:10.2147/DDDT.S214116
21. Nie F, Zhang W, Cui Q, Fu Y, Li H, Zhang J. Kaempferol promotes proliferation and osteogenic differentiation of periodontal ligament stem cells via Wnt/beta-catenin signaling pathway. *Life Sci*. 2020;1:258.
22. Cui Q, Li N, Nie F, Yang F, Li H, Zhang J. Vitamin K2 promotes the osteogenic differentiation of periodontal ligament stem cells via the Wnt/beta-catenin signaling pathway. *Arch Oral Biol*. 2021;124.
23. Fu Y, Ma D, Fan F, et al. Noncanonical Wnt5a Signaling Suppresses Hippo/TAZ-Mediated Osteogenesis Partly Through the Canonical Wnt Pathway in SCAPs. *Drug Des Devel Ther*. 2022;16:469–483. doi:10.2147/DDDT.S350698
24. Choi MH, Noh WC, Park JW, Lee JM, Suh JY. Gene expression pattern during osteogenic differentiation of human periodontal ligament cells in vitro. *J Periodontal Implant Sci*. 2011;41(4):167–175. doi:10.5051/jpis.2011.41.4.167
25. Xu JP, Wang W, Kapila Y, Lotz J, Kapila S. Multiple Differentiation Capacity of STRO-1(+)/CD146(+) PDL Mesenchymal Progenitor Cells. *Stem Cells Dev*. 2009;18(3):487–496. doi:10.1089/scd.2008.0113
26. Sun S, Wang C-Z, Tong R, et al. Effects of steaming the root of Panax notoginseng on chemical composition and anticancer activities. *Food Chem*. 2010;118(2):307–314. doi:10.1016/j.foodchem.2009.04.122
27. Wang T, Guo R, Zhou G, et al. Traditional uses, botany, phytochemistry, pharmacology and toxicology of Panax notoginseng (Burk.) FH Chen: a review. *J Ethnopharmacol*. 2016;188:234–258.
28. Wang Q, Mu RF, Liu X, et al. Steaming Changes the Composition of Saponins of Panax notoginseng (Burk.) FH Chen That Function in Treatment of Hyperlipidemia and Obesity. *J Agric Food Chem*. 2020;68(17):4865–4875. doi:10.1021/acs.jafc.0c00746
29. Cao B, Xu Z, Liu C, et al. Protective effects of notoginsenoside R1 on acute lung injury in rats with sepsis. *Ann Translational Med*. 2021;9(12). doi:10.21037/atm-21-2496.
30. Liu Y, Lin Z, Guo J, et al. Notoginsenoside R1 significantly promotes in vitro osteoblastogenesis. *Int J Mol Med*. 2016;38(2):537–544. doi:10.3892/ijmm.2016.2652
31. Hatta M, Daitoku H, Matsuzaki H, et al. Regulation of alkaline phosphatase promoter activity by forkhead transcription factor FKHR. *Int J Mol Med*. 2002;9(2):147–152.
32. Du L, Yang P, Ge S. Stromal cell-derived factor-1 significantly induces proliferation, migration, and collagen type I expression in a human periodontal ligament stem cell subpopulation. *J Periodontol*. 2012;83(3):379–388. doi:10.1902/jop.2011.110201
33. Maes C, Goossens S, Bartunkova S, et al. Increased skeletal VEGF enhances beta-catenin activity and results in excessively ossified bones. *EMBO J*. 2010;29(2):424–441. doi:10.1038/emboj.2009.361
34. Nishimura R, Hata K, Kida J. Regulation of osteoblasts and chondrocytes by Wnt signaling. *Clin Calcium*. 2019;29(3):299–307. doi:10.20837/4201903299
35. Kajkenova O, LeckaCzernik B, Gubrij I, et al. Increased adipogenesis and myelopoiesis in the bone marrow of SAMP6, a murine model of defective osteoblastogenesis and low turnover osteopenia. *J Bone Mineral Res*. 1997;12(11):1772–1779. doi:10.1359/jbmr.1997.12.11.1772
36. Song L, Liu M, Ono N, Bringhurst FR, Kronenberg HM, Guo J. Loss of Wnt/beta-Catenin Signaling Causes Cell Fate Shift of Preosteoblasts From Osteoblasts to Adipocytes. *J Bone Mineral Res*. 2012;27(11):2344–2358. doi:10.1002/jbmr.1694
37. Huang S-MA, Mishina YM, Liu S, et al. Tankyrase inhibition stabilizes axin and antagonizes Wnt signalling. *Nature*. 2009;461(7264):614–620. doi:10.1038/nature08356

Drug Design, Development and Therapy

Dovepress

Publish your work in this journal

Drug Design, Development and Therapy is an international, peer-reviewed open-access journal that spans the spectrum of drug design and development through to clinical applications. Clinical outcomes, patient safety, and programs for the development and effective, safe, and sustained use of medicines are a feature of the journal, which has also been accepted for indexing on PubMed Central. The manuscript management system is completely online and includes a very quick and fair peer-review system, which is all easy to use. Visit http://www.dovepress.com/testimonials.php to read real quotes from published authors.

Submit your manuscript here: https://www.dovepress.com/drug-design-development-and-therapy-journal
