# Supplementary material for: Molecular Regulators for the Wnt/β-Catenin Signalling Pathway for Bone Remodelling in Periodontitis: A Scoping Review
Source: Int J Mol Sci. 2026 Oct 14;27(20):9117. doi: 10.3390/ijms27209117 (PMC13705578; doi:10.3390/ijms27209117)
Supplement: Supplementary file 1 [file ijms-27-09117-s001.zip › Supplementary Data/Papers/in vitro/Osteogenic growth peptide enhances osteogenic differentiation of human periodontal ligament stem cells.pdf]

Heliyon 8 (2022) e09936

Contents lists available at ScienceDirect

Heliyon

journal homepage: www.cell.com/heliyon

Research article

Osteogenic growth peptide enhances osteogenic differentiation of human periodontal ligament stem cells

Steven Dwi Purbantoro [a,b,c], Thanaphum Osathanon [d,e], Sirirat Nantavisai [b,c,f,**], Chenphop Sawangmake [b,c,g,h,*]

[a] The International Graduate Program of Veterinary Science and Technology (VST), Faculty of Veterinary Science, Chulalongkorn University, Bangkok, 10330, Thailand
[b] Veterinary Stem Cell and Bioengineering Innovation Center (VSCBIC), Faculty of Veterinary Science, Chulalongkorn University, Bangkok, 10330, Thailand
[c] Veterinary Stem Cell and Bioengineering Research Unit, Faculty of Veterinary Science, Chulalongkorn University, Bangkok, 10330, Thailand
[d] Department of Anatomy, Faculty of Dentistry, Chulalongkorn University, Bangkok, 10330, Thailand
[e] Dental Stem Cell Biology Research Unit, Faculty of Dentistry, Chulalongkorn University, Bangkok, 10330, Thailand
[f] Academic Affairs, Faculty of Veterinary Science, Chulalongkorn University, Bangkok, 10330, Thailand
[g] Department of Pharmacology, Faculty of Veterinary Science, Chulalongkorn University, Bangkok, 10330, Thailand
[h] Center of Excellence in Regenerative Dentistry, Faculty of Dentistry, Chulalongkorn University, Bangkok, 10330, Thailand

ARTICLE INFO

Keywords:
Osteogenic growth peptide (OGP)
Human periodontal ligament stem cells (hPDLSCs)
Osteogenic differentiation
Bone regeneration

ABSTRACT

Bone tissue engineering consists of three major components namely cells, scaffolds, and signaling molecules to improve bone regeneration. These integrated principles can be applied in patients suffered from bone resorption diseases, such as osteoporosis and periodontitis. Osteogenic growth peptide (OGP) is a fourteen-amino acid sequence peptide that has the potential to regenerate bone tissues. This study aimed to disseminate the osteogenic differentiation of human periodontal ligament stem cells (hPDLSCs) with OGP treatment. OGP was elaborated for proliferation, cytotoxicity, osteogenic differentiation effects, and the involvement of osteogenic related signaling pathways *in vitro*. This study found that OGP at lower concentration shows better effects on cytotoxicity and proliferation. Moreover, OGP at concentration 0.01 nM had the most potential to differentiate hPDLSCs toward osteogenic lineage comparing with higher concentrations of OGP. The phenomenon was mainly involving transforming growth factor-beta (TGF-β), bone morphogenetic protein (BMP), Hedgehog, and Wingless-related (Wnt) pathways. Further, SB-431542 treatment demonstrated the partial involvement of OGP in regulating osteogenic differentiation of hPDLSCs. In conclusion, OGP at low concentration enhances osteogenic differentiation of hPDLSCs by governing TGF-β signaling pathway.

1. Introduction

Bone resorption in osteoporosis and periodontitis has been highlighted as a global health burden to be solved (Lems and Raterman, 2017; Nazir, 2017). The effects of these two diseases have an impact on the quality of life of the patients (Lems and Raterman, 2017; Tonetti et al., 2017). Current treatment using either anti-resorptive or anabolic agent alone has been suggested may not be enough to achieve successful results. Therefore, combination of both agents is needed to accelerate osteogenesis (Chen et al., 2011). To this matter, bone tissue engineering (BTE) can be applied to treat the diseases. Generally, BTE comprises cells, scaffolds, and signaling molecules (Langer and Vacanti, 1993).

Osteogenic growth peptide (OGP) is a tetradecapeptide that was firstly introduced in the post-ablation rat bone marrow serum by Bab and his colleagues (Bab et al., 1988, 1992). It has been introduced to cope with the current problem. Several studies found that OGP had the ability to improve bone anabolic and anti-resorption activity by expressing anabolic- (HO-1, CXCL13, and RhoA/ROCK pathway) and anti-resorptive-related molecules (OPG) (Spreafico et al., 2006; Vanella et al., 2010; Chen et al., 2011; Li et al., 2015). Thus, OGP has the potential to be used for treating osteoporosis and/or periodontitis. However, there are still lacking the basic information of OGP on osteogenic differentiation, especially in human periodontal ligament stem cells (hPDLSCs).

* Corresponding author.
** Corresponding author.
E-mail addresses: sirirat.ra@chula.ac.th (S. Nantavisai), chenphop.s@chula.ac.th (C. Sawangmake).

https://doi.org/10.1016/j.heliyon.2022.e09936
Received 28 December 2021; Received in revised form 9 February 2022; Accepted 7 July 2022
2405-8440/© 2022 The Author(s). Published by Elsevier Ltd. This is an open access article under the CC BY-NC-ND license (http://creativecommons.org/licenses/by-nc-nd/4.0/).

The hPDLSCs were firstly isolated and characterized in 2004 (Seo et al., 2004). These cells were able to differentiate into various cell types, including neuronal like cells, insulin producing cells, adipocytes, and osteoblasts (Sawangmake et al., 2014a). hPDLSCs have a high potency for bone regeneration (Seo et al., 2004; Huang et al., 2009; Song et al., 2015). Interestingly, osteogenic differentiated-hPDLSCs are still able to conserve the immunomodulatory capacity (Tang et al., 2014). Moreover, as one of dental tissue-derived MSCs, hPDLSCs are easy to obtain and expand in a high yield of cells (Dave and Tomar, 2018). Thus, hPDLSCs have become an interest to be selected as a potential candidate to regenerate bone tissues.

The present study aimed to explore the effects of OGP on the osteogenic differentiation in hPDLSCs *in vitro*. In addition, the potentially participated pathway in OGP-modulated osteogenic differentiation was investigated.

2. Materials and methods

2.1. Isolation and culture of hPDLSCs

hPDLSCs were isolated and cultured according to our previous study (Manokawinchoke et al., 2019, 2020). The study protocol was approved by the Human Research Ethic Committee, Faculty of Dentistry, Chulalongkorn University (HREC-DCU, 2018–054) with informed consent from donors. Briefly, periodontal ligament (PDL) was collected from each extracted permanent molar tooth of both male and female donors ($n = 4$) aged 18–35 years old without any pathological conditions and orthodontic treatment scheduled to be removed according to patients' treatment plan. PDL tissues were collected by scrapping at the middle-third area of the root. PDL tissues were then put on a 35 mm culture dish for cell explantation. Cells were maintained in Dulbecco's Modified Eagle Medium (DMEM) (Thermo Fisher Scientific, USA) with 10% fetal bovine serum (FBS) (Thermo Fisher Scientific, USA), 1% Antibiotic-Antimycotic (Thermo Fisher Scientific, USA), and 1% Glutamax (Thermo Fisher Scientific, USA). The isolated cells were incubated at 37 °C with 5% CO_2. Growth medium was changed every 48 h and subcultured at 80% confluence. We employed passage 2–5 of hPDLSCs for the following experiments.

For OGP treatment, OGP was purchased from MedChemExpress (Cat. No. HY-P1563, USA). hPDLSCs were cultured with OGP supplemented in culture medium at concentrations of 0.01, 0.1, 1, 10, and 100 nM according to previous publication (Fei et al., 2010).

In some experiments, cells were treated with a specific inhibitor of TGF-β, SB-431542 (Sigma-Aldrich, USA) at the concentration of 4 μM. Dimethyl sulfoxide (DMSO) (Sigma-Aldrich, USA) was used as the solvent for SB-431542.

2.2. Reverse transcription quantitative polymerase chain reaction (RT-qPCR)

Total RNA was collected by using TRIzol® reagent (Invitrogen, USA) and Direct-Zol RNA isolation kit (ZymoResearch, USA). Conversion of RNA to complementary DNA (cDNA) was performed with Improm-II™ Reverse Transcription System kit (Promega, USA. Quantitative real-time PCR (qPCR) assay was done using PowerUp™ SYBR™ Green Master Mix (Thermo Fisher Scientific, USA) in a Bio-Rad Real-Time PCR Detection System (Bio-Rad, USA). Primers used in this study are presented at Table 1. The *18S* ribosomal RNA was used as the reference gene for normalization of the targeted mRNA expression, calculated using formula $2^{-\Delta Ct}$, whereas $\Delta Ct = Ct^{target\ gene} - Ct^{18S}$. Results compared with control group were calculated as relative mRNA expression, $2^{-\Delta\Delta Ct}$. The $\Delta\Delta Ct$ was obtained from $\Delta Ct^{treatment} - \Delta Ct^{control}$. The Ct value in this procedure is referred to cycle threshold (Sawangmake et al., 2016; Nantavisai et al., 2019).

2.3. Flow cytometry

MSCs surface marker expression on hPDLSCs was evaluated using flow cytometry analysis ($n = 4$). The antibodies were FITC-conjugated anti-human CD105 antibody (Bio Legend, USA), FITC-conjugated anti-human CD73 antibody (Bio Legend, USA), FITC-conjugated anti-human CD90 antibody (Bio Legend, USA), FITC-conjugated anti-human CD44 antibody (Bio Legend, USA), and FITC-conjugated anti-human CD45 antibody (Bio Legend, USA). FITC-conjugated mouse IgG1κ Isotype was employed as the isotype control. The assay was performed by utilizing FACS caliber flow cytometer (BD Biosciences, USA).

Table 1. Primers used for RT-qPCR.

Gene	Forward Primer Sequence (5′-3′)	Reverse Primer Sequence (5′-3′)
REX1	TGGGAAAGCGTTCGTTGAGA	CACCCTTCAAAAGTGCACCG
NANOG	ATGCCTCACACGGAGACTGT	AAGTGGGTTGTTTGCCTTTG
OCT4	TCGAGAACCGAGTGAGAGG	GAACCACACTCGGACCACA
KI67	TCACTCTCATCAGGGTCAGAAG	TCAGAATGGAAGGAAGTCAACTG
RUNX2	CCCCACGACAACCGCACCAT	CACTCCGGCCCACAAATC
OSX	GCCAGAAGCTGTGAAACCTC	GCTGCAAGCTCTCCATAACC
OCN	CTTTGTGTCCAAGCAGGAGG	CTGAAAGCCGATGTGGTCAG
OPN	AGGAGGAGGCAGAGCACA	CTGGTATGGCACAGGTGATG
COL1A1	CTGGCAAAGAAGGCGGCAAA	CTCACCACGATCACCACTCT
ALP	CGAGATACAAGCACTCCCACTTC	CTGTTCAGCTCGTACTGCATGTC
PPARG	GTGACCAGAAGCCTGCATTT	GTCAACCATGGTCATTTCGTT
LPL	CATGGCTGGACGGTAACAGG	CGGACACTGGGTAATGCTCC
COL10A1	TCCCAGCACGCAGAATCCATC	TGTCTTGGTGTTGGGTAGTGG
SOX9	GGCAAGCTCTGGAGACTTCTG	CCCGTTCTTCACCGACTTCC
ID1	CAGGGACCTTCAGTTGGAGC	AACGCATGCCGCCTCG
DLX5	AGCTCTCAACCCCTACCAGT	CGGTCACTTCTTTCTCTGGC
MSX2	CCGGGGCAGGAGTAGGAG	AGCTGGGATGTGGTAAAGGG
PTCH1	GTTCACGTTGCTTTGGCCTT	CGCCAGCACAGCAAAGAAAT
GLI1	CCCAGACAGAGGCCCACTC	CTGGGTGAGGTGCGGATAAC
HHIP	TCCACGATCCAGGCAGATGT	TCCACCAACCAAAGGACCAT
HES1	AGGCGGACATTCTGGAAATG	CGGTACTTCCCCAGCACACTT
HEY1	TAATTGAGAAGCGCCGACGA	GCAACTTCTGCCAGGCATTC
LFNG	GATCTCGCGCCACAAGGAG	ACGTGGCAGAACCACTTCC
BMP2	TGCGGTCTCCTAAAGGTCG	AACTCGAACTCGCTCAGGAC
TMEFF1	TGCTTTCTCAGAAGGGCTGC	CCTGACCCTTCCTCTTCTCCT
CXXC5	GGCAAGAAGAAGCGGAAACG	TCGGAAGCATCACCTTCTCC
LEF1	TCTTCCTTGGTGAACGAGTCT	GATGCTTTCCGTCATCGGG
TCF7	GACAACTACGGGAAGAAGAAGAGG	CAAGCTGGGCTAGAGGAAGAAG
18S	GTGATGCCCTTAGATGTCC	CCATCCAATCGGTAGTAGC

RUNX2, Runt-related transcription factor 2; *OSX*, osterix; *OCN*, osteocalcin; *OPN*, osteopontin; *COL1A1*, collagen type I alpha 1 chain; *ALP*, alkaline phosphatase; *PPARG*, peroxisome proliferator activated receptor gamma; *LPL*, lipoprotein lipase; *COL10A1*, collagen type X alpha 1 chain; *SOX9*, SRY-box transcription factor 9; *ID1*, inhibitor of DNA binding 1; *DLX5*, distal-less homeobox 5; *MSX2*, Msh homeobox 2; *HHIP*, Hedgehog interacting protein; *HES1*, hairy and enhancer of split-1; *HEY1*, Hes related with YPRW motif protein 1; *LFNG*, lunatic fringe; *BMP2*, bone morphogenetic protein 2; *TMEFF1*, tomoregulin 1; *LEF1*, lymphoid enhancer binding factor 1; and *TCF7*, transcription factor 7.

2.4. Colony forming assay

The protocol was modified from previous publications (Nantavisai et al., 2020; Nowwarote et al., 2020). Briefly, hPDLSCs were cultured at the concentration of 500 cells in a 60 mm culture dish (Corning, USA) and maintained in growth medium for 14 days. Cells were washed with phosphate buffer saline (PBS) and then fixed with 100% methanol (Sigma-Aldrich, USA) and stained with crystal violet (Sigma-Aldrich, USA). Fifty aggregated cells were counted and considered as a single colony.

2.5. Osteogenic differentiation

Osteogenic differentiation protocol was modified from our previously reports (Sawangmake et al., 2014a, 2014b). hPDLSCs (3×10^4 cells)

were seeded per well in a 24-well culture plate (Corning, USA). Cells were maintained in osteogenic induction medium containing growth medium supplemented with 50 mg/mL L-ascorbic-2-2phosphate (Sigma-Aldrich, USA), 100 nM dexamethasone (Sigma-Aldrich, USA), and 10 mM β-glycerophosphate (Sigma-Aldrich, USA) for 21 d. The culture medium was changed every 48 h.

2.6. Adipogenic differentiation

Adipogenic induction was performed following our previous study (Nantavisai et al., 2020). A total of 3×10^4 cells was seeded per well in a 24-well culture plate (Corning, USA). Induction was performed for 23 d by culturing the hPDLSCs in adipogenic induction and maintaining media. Induction medium was growth medium supplemented with 1 μM dexamethasone (Sigma-Aldrich, USA), 0.1 nM indomethacin (Sigma, USA), 1 mM 3-isobutyl-1-methylxanthine (IBMX) (Sigma-Aldrich, USA), and 1 μg/mL insulin (Sigma-Aldrich, USA). The maintaining medium was growth media supplemented with 1 μg/mL insulin. Cells were cultured with adipogenic induction medium for 72 h and then changed to maintaining medium for another 24 h. Four cycles of the induction were performed and continued by maintaining the cells in maintaining medium until 23 days of induction. Cells were then stained by Oil Red O (Sigma-Aldrich, USA) and analyzed for adipogenic gene expression by RT-qPCR.

2.7. Chondrogenic differentiation

Chondrogenic induction was performed according to previous published protocol (Nantavisai et al., 2020). Cells were seeded at the density of 5×10^4 cells per well in a 24-well culture plate (Corning, USA) and further maintained in chondrogenic induction medium for 21 days. Chondrogenic induction medium contained DMEM supplemented with 1% Glutamax (Thermo Fisher Scientific, USA), 1% antibiotic-antimycotic (Thermo Fisher Scientific, USA), 50 mg/mL L-ascorbic-2-2phosphate (Sigma-Aldrich, USA), 40 mg/mL L-proline (Sigma-Aldrich, USA), 0.1 μM dexamethasone (Sigma-Aldrich, USA), 1% insulin-transferrin-selenium (Thermo Fisher Scientific, USA), 10 ng/mL transforming growth factor beta 3 (TGF-β3) (Sigma-Aldrich, USA), and 15% FBS (Thermo Fisher Scientific, USA). The medium was changed every 48 h. At day 21, a 0.1% alcian blue solution (Sigma-Aldrich, USA) was used to stain acidic polysaccharide. Chondrogenic marker gene expression was analyzed by RT-qPCR.

2.8. Alkaline phosphatase activity

At day 7, 14, and 21 after osteogenic induction, the alkaline phosphatase (ALP) activity was assessed following to our previous reports (Sawangmake et al., 2016; Nantavisai et al., 2020). Briefly, cell lysate was collected using lysis buffer containing 0.1% Triton X-100, 1 M Tris-HCl, and 5 mM $MgCl_2$. Cell lysate was further incubated with *p*-nitrophenol phosphate (PNPP) (Life Technologies Corp., USA), 2-amino-2-methyl-1-propanolol (Sigma-Aldrich, USA), and 2 mM of $MgCl_2$ for 15 min at 37 °C. Addition of 0.1 M NaOH was done to stop the reaction. The absorbance was measured at wavelength of 410 nm. Furthermore, total protein concentration was determined by using Qubit® (Thermo Fisher Scientific, USA) following the manufacturer's protocol. The ALP activity was calculated in U/mg protein unit.

2.9. Alizarin Red S staining

At day 7, 14, and 21 after osteogenic induction, Alizarin Red S staining was performed following to the published report (Sawangmake et al., 2016). Samples were washed with PBS and fixed with cold methanol for 20 min at 4 °C. Next, distilled water (pH 4.2) was used to wash the cells for 3 times. Samples were then stained with 2% solution of Alizarin Red S (Sigma-Aldrich, USA) and subsequently washed with distilled water for 2–3 times. Mineralization was visualized under an inverted microscope as red nodules. Quantification of Alizarin Red S staining was then performed by eluting each well used 10% cetylpyridinium chloride (CPC) and measured the absorbance at 550 nm.

2.10. AlamarBlue™ assay

After culturing cells in the condition with or without OGP for 1, 5, and 7 days, cells were seeded at the density of 5×10^3 cells per well in a 24-well culture plate (Corning, USA) and incubated in growth medium supplemented with 5% alamarBlue™ (Invitrogen, USA) for 3 h. Absorbance of reduced and oxidized from alamarBlue™ was then measured by spectrophotometer at wavelength of 570 and 600 nm, respectively. Next, reduction percentage was calculated following to the manufacturer's protocol.

2.11. Live/dead assay

After OGP treatment for 1, 5, and 7 days in growth medium, live/dead assay was performed by using calcein-AM and propidium iodide (PI) to stain OGP-untreated and -treated hPDLSCs according to the manufacturer's protocol. Initially, cells were seeded at the density of 5×10^3 cells per well in a 24-well culture plate (Corning, USA). Carl Zeiss™ Apoptome.2 apparatus-equipped fluorescent microscope (Carl Zeiss, USA) was used to evaluate the stained cells. Viable cells were visualized in green color, while dead cells were seen in red color.

2.12. Statistical analysis

The results were analyzed by SPSS Statistics Program Ver 22.0 (IBM, USA). For two independent groups, the independent two-tailed *t*-test was employed. Analysis comparison of more than two groups was performed by using Kruskal-Wallis and post-hoc Mann-Whitney U test. A *p*-value$<$0.05 was set to be significant difference.

3. Results

3.1. hPDLSCs presented MSCs properties

The isolated hPDLSCs adhered on plastic surface and exhibited fibroblast-like morphology. Cells also expressed MSCs-related markers (CD73, CD90, CD44, and CD105) and diminutively expressed hematopoietic stem cells markers (CD45) as determined by flow cytometry analysis (Figure 1A-B). Cells expressed the pluripotent stem cells: *REX1*, *NANOG,* and *OCT4* as well as the proliferative marker: *KI67* (Figure 1C). These isolated cells were able to form colonies *in vitro* (Figure 1D-E). In addition, hPDLSCs showed the multilineage differentiation potential toward osteogenic, adipogenic, and chondrogenic lineages as shown by the accumulation of mineralization, intracellular lipid droplets, and acidic polysaccharide, respectively. These phenotypes also corresponded with the increased expression of mRNA levels of genes related to osteogenic (*RUNX2*, *OSX*, and *OCN*), adipogenic (*PPARG* and *LPL*), and chondrogenic differentiation (*COL10A1* and *SOX9*) (Figure 1F-K).

3.2. Low-dose OGP promoted hPDLSCs proliferation

The schematic on proliferation effect of OGP is illustrated in Figure 2A. The proliferation assay result at day 1 and 5 revealed that 0.01, 0.1, and 1 nM OGP significantly enhanced cell proliferation comparing with the control, while 10 and 100 nM OGP did not increase the proliferation rate of hPDLSCs. At day 7, 0.01 and 0.1 nM of OGP showed the trend to increase cell proliferation comparing with control group. However, there was no statistically significant difference (Figure 2B). Live/dead staining results were correlated to proliferation assay. Dead cells were found in a few of number from all OGP groups at day 1, 5, and 7 (Figure 2C). Therefore, low concentration of OGP has the

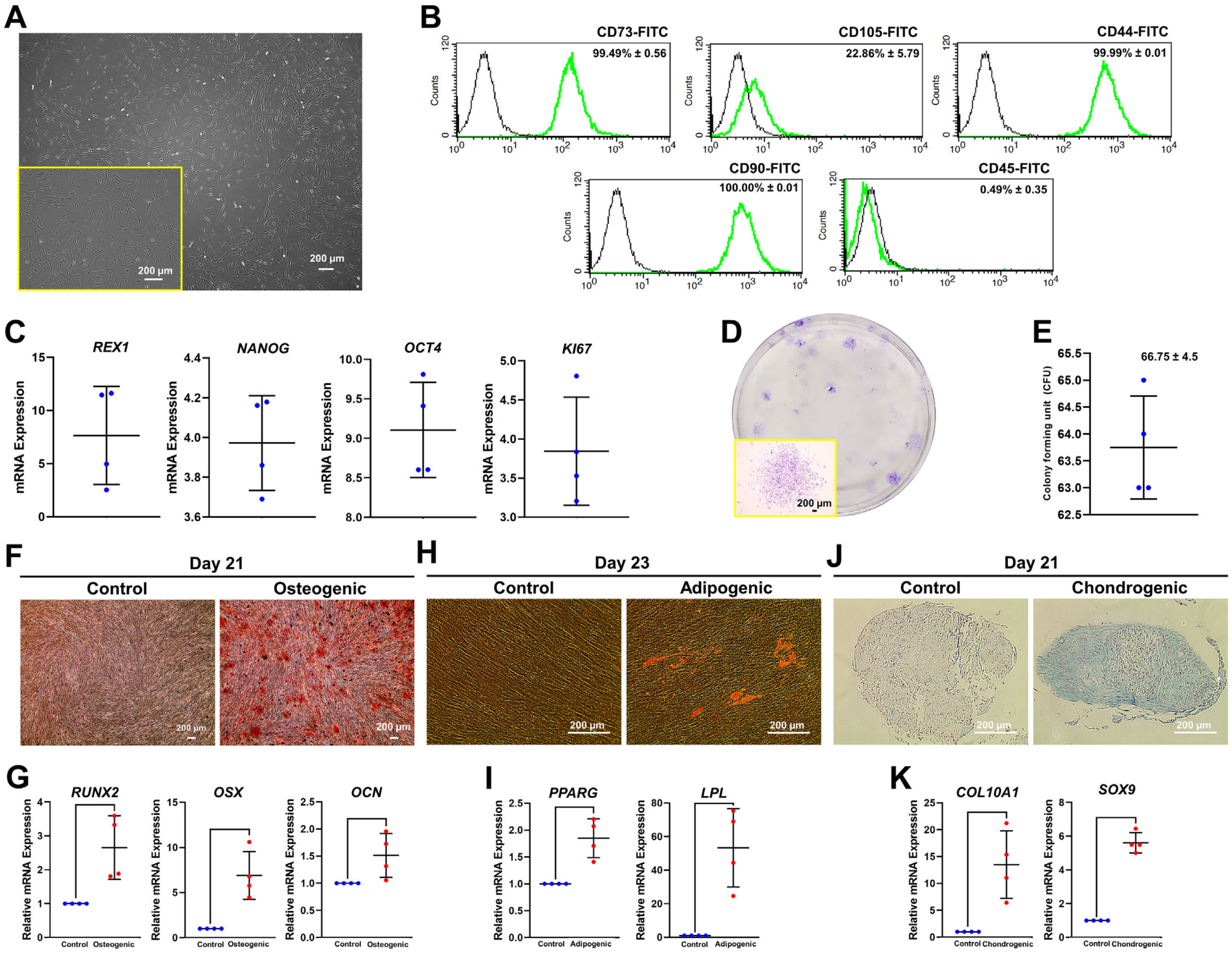

Figure 1. Characterization of hPDLSCs. (A) Plastic-adherent ability and spindle-shaped morphology, (B) flow cytometry analysis, (C) stemness and proliferative markers, and (D–E) colony forming ability and colony count were investigated. (F–G) Osteogenic differentiation was examined using Alizarin Red S staining and osteogenic marker gene expression. (H–I) Adipogenic differentiation was investigated using Oil Red O staining and adipogenic marker gene expression. (J–K) Chondrogenic differentiation was examined using alcian blue staining and chondrogenic marker gene expression. The mRNA expression was examined using qPCR. Bars indicated the significant difference between groups ($p<0.05$).

potential to promote cell proliferation without cytotoxic effect on hPDLSCs.

3.3. Low-dose OGP enhanced osteogenic differentiation potential by hPDLSCs

To observe osteogenic induction potential under OGP treatment, hPDLSCs were treated with different concentration of OGP range from 0.01-100 nM under osteogenic induction. The schematic of OGP treatment in osteogenic differentiation of hPDLSCs is illustrated in Figure 3A. The ALP activity revealed that although undifferentiated control group had the highest ALP activity comparing with all experiment groups, low-dose (0.01 and 0.1 nM) of OGP illustrated the trend to be higher ALP activity than high-dose (1, 10, and 100 nM) of OGP at day 7 post-induction. Interestingly, at day 14 and 21 post-induction, low-dose OGP (0.01 and 0.1 nM) showed the increased trend of ALP activity comparing with the control, OM, and high-dose OGP groups (1–100 nM) (Figure 3B). In addition, high-dose (1, 10, and 100 nM) of OGP showed significantly lower ALP activity than OM group at day 21 post-induction. The osteogenic mRNA expression showed that low-dose of OGP induced higher mRNA expression of *ALP*, *OPN*, and *OCN* than high-dose of OGP (Figure 3C). In addition, OGP at concentration 1 nM showed higher expressions of *RUNX2*, *OSX*, and *COL1A1* comparing with 0.01 and 0.1 nM of OGP. Mineral deposition demonstrated that the concentration 0.01 and 0.1 nM of OGP had greater mineral deposition than those treated with high-dose (1, 10, and 100 nM) of OGP (Figure 3D). The results of Alizarin Red S staining quantification revealed that 0.01, 0.1, and 1 nM of OGP had superior capability in increasing mineralization comparing the other concentrations (Figure 3E). These findings suggested that the exposure of low concentration of OGP enhanced osteogenic differentiation more than high concentration on hPDLSCs.

3.4. OGP governed osteogenic differentiation in hPDLSCs via TGF-β pathway

Due to the osteogenic differentiation potential of low-dose of OGP, 0.1 nM of OGP was considered and selected to extensively explore for osteogenic relating pathway including bone morphogenetic protein (BMP), Hedgehog, Notch, transforming growth factor-beta (TGF-β), and Wingless-related (Wnt) signaling pathways (Vanella et al., 2010; Chen et al., 2012; Houschyar et al., 2019; Manokawinchoke et al., 2020; Men et al., 2020). The schematic of OGP on the mRNA expression of gene

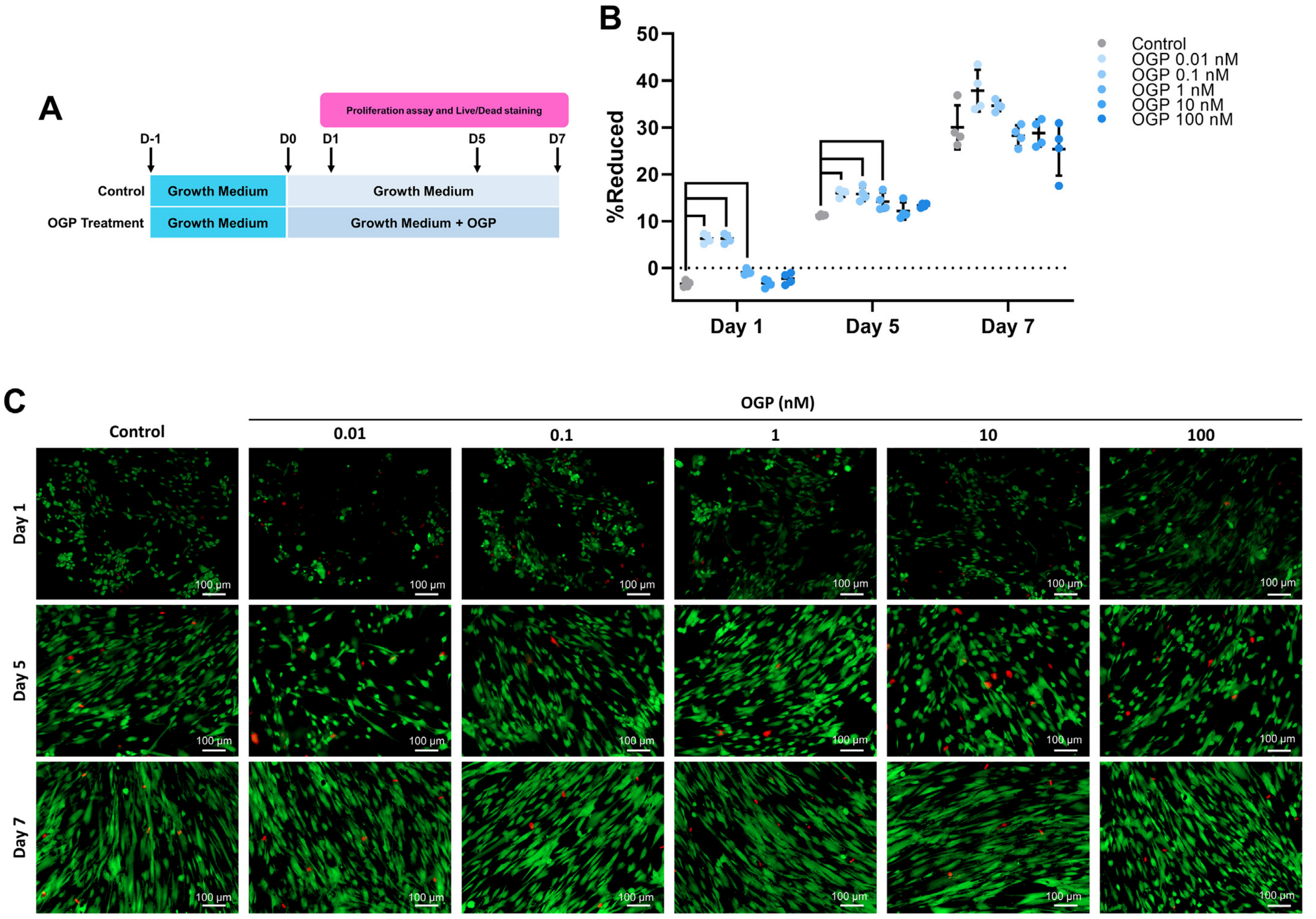

Figure 2. Proliferation and cytotoxic effects from OGP treatment on hPDLSCs. (A) Schematic experiment and (B) proliferation assay for mitogenic activity using alamarBlue™ assay were presented. (C) Live cells stained with calcein-AM and dead cells stained with propidium iodide were illustrated in green and red, respectively. Bars on proliferation assay result indicated the significant difference ($p<0.05$).

related to osteogenic induction signaling is illustrated in Figure 4A. The mRNA expression of target genes related to BMP, Hedgehog, Notch, TGF-β, and Wnt signaling are illustrated in Figure 4B-F. In BMP signaling, OGP upregulated the target genes higher than OM, but only *DLX5* was found significantly difference both at day 1 and 3. The *PTCH1* and *GLI1* of Hedgehog signaling were expressed significantly higher in OGP than OM at day 1 and 3. In addition, *HHIP* of OGP was found to be downregulated at day 3 and lower comparing with OM. Interestingly, different expression of Notch specific target genes was observed. Although OGP expressed *HES1* higher than OM at day 1, the result was in contrast at day 3. Moreover, *HES1* and *LFNG* were downregulated extremely in OGP group at day 3. Further investigation in TGF-β signaling showed that OGP was able to significantly express *BMP2*, *TMEFF1*, and *CXXC5* higher than OM both at day 1 and 3. Also, *LEF1* and *TCF7* in OGP were found upregulated higher than OM, even though only *LEF1* at day 1 was significant difference. Thus, among mRNA expression from selected pathways, target gene expression related to TGF-β signaling seemed the most potent OGP-regulated pathway during osteogenic differentiation, considering the higher TGF-β target gene expression at day 1 and 3.

To confirm the involvement of TGF-β signaling in OGP-induced osteogenic differentiation in hPDLSCs, SB-431542 was added in osteogenic induction medium concurrently with 0.1 nM of OGP on hPDLSCs. The schematic of this experiment is illustrated in Figure 5A.

In ALP activity result, SB-431542 supplementation in OGP-treated condition did not attenuate the ALP activity at day 7, 14, and 21 (Figure 5B). However, SB-431542 attenuated OGP-induced osteogenic marker gene expression by hPDLSCs (Figure 5C). For mineralization, SB-431542 did not markedly decrease mineral deposition in OGP treated conditions at all time points (Figure 5D). However, the staining quantification showed that cell treated with OGP only had greater result than cells treated with OGP and SB-431542 (Figure 5E). This implies the partial involvement of TGF-β signaling on OGP-induced osteogenic differentiation in hPDLSCs.

4. Discussion

This study explored the osteogenic differentiation effects of OGP on hPDLSCs. Confirming previous studies in other cell types, OGP-induced osteogenic differentiation in hPDLSCs as determined by the increased ALP activity, osteogenic marker gene expression, and mineral deposition. However, the osteogenic inductive effects were specific to low concentration of OGP.

hPDLSCs used in this study were defined as MSCs based on minimal criteria from International Society for Cellular Therapy (ISCT) (Dominici et al., 2006). The criteria include plastic adherent, expressed specific surface markers, and multipotential differentiation ability. We observed high percentage of CD44, CD90, and CD73 expression while CD105 positive cells were relatively low. However, this result was supported by the other markers to define our isolated hPDLSCs as MSCs. Similar result of lowly expressed CD105 was also found in previous published report (Kuncorojakti et al., 2020). The isolated cells expressed pluripotent stem cell markers and proliferative marker similar to previous report

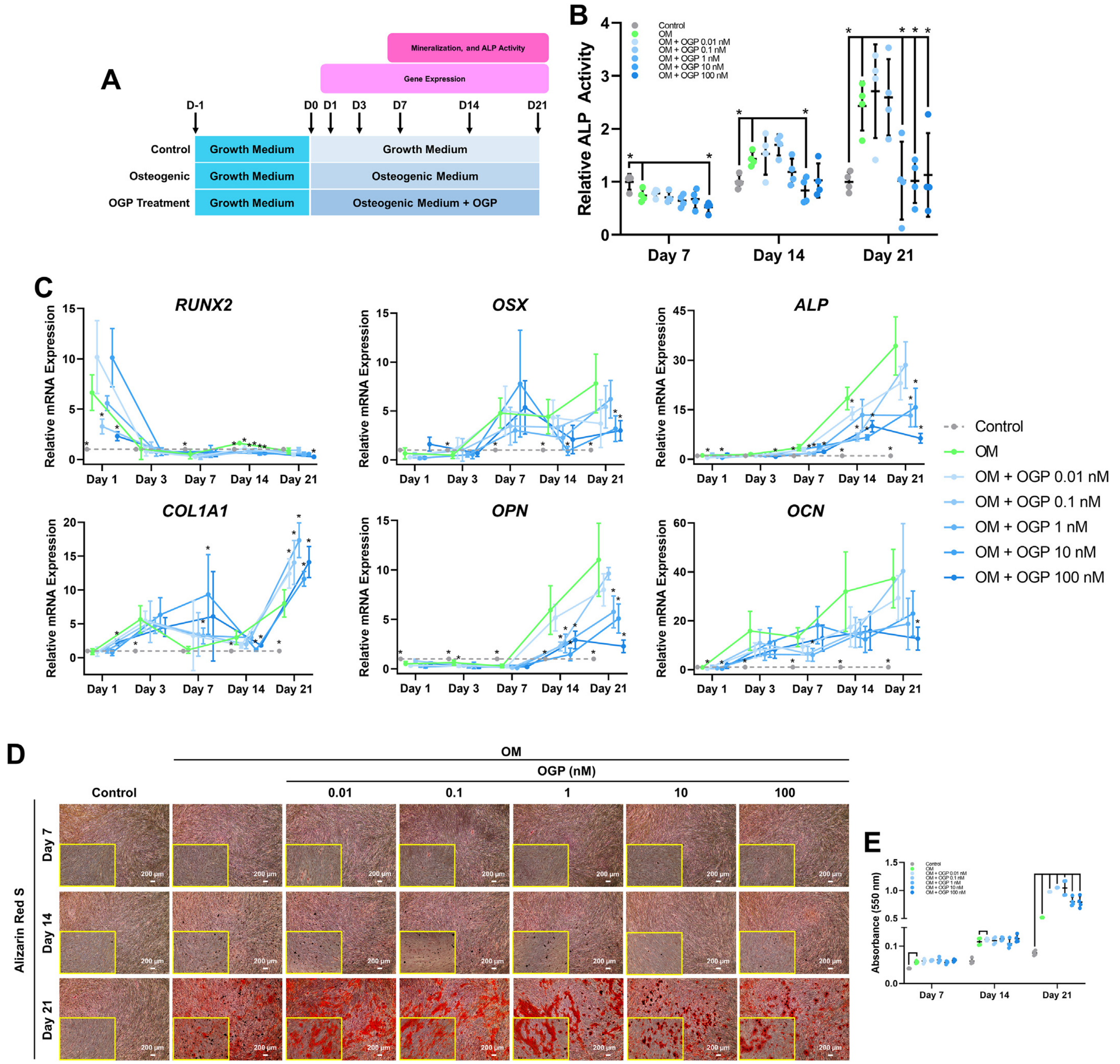

Figure 3. Osteogenic differentiation effects of OGP on hPDLSCs. (A) Schematic experiment of OGP treatment on hPDLSCs toward osteogenic lineage. (B) The ALP activity, (C) osteogenic-related marker expression, (D) mineral deposition, and (E) Alizarin Red S staining quantification were presented. Bars on ALP activity results and Alizarin Red S staining quantification and asterisks on gene expression results indicated the statistically significant difference between OM and other groups ($p<0.05$).

(Sawangmake et al., 2014b). The markers have also been reported to be expressed in different origin of MSCs, such as bone marrow and dental pulp, from different species (Nantavisai et al., 2019, 2020; Purwaningrum et al., 2021). Moreover, it is supported by the self-renewal and proliferative capabilities the cells possessed (Aponte and Caicedo, 2017). Corresponding to previous publications, the isolated hPDLSCs were found as well having the differentiation capability of hPDLSCs toward osteogenic, adipogenic, and chondrogenic lineages (Gay et al., 2007; Sawangmake et al., 2014a, 2014b).

The present study described that the low concentration of OGP promoted hPDLSCs proliferation. This could be due to the activation of mitogen-activated protein kinase (MAPK) pathways. The active form of OGP can be formed after extracellular proteolysis and subsequently binds G_i protein-coupled receptor (GPCR), leading to the initiation of signaling transduction via mitogen-activated protein kinase (MAPK) pathways (Gabarin et al., 2001). The activation is occurred due to G_α, a G protein subunit included with G_i, has a domain that similar to Ras (McCudden et al., 2005). The stimulation of Ras is associated further with the MAPK pathway activation (Goldsmith and Dhanasekaran, 2007). Through the activation of GPCR, MAPK pathways are able to regulate cell proliferation via its complex cascades. Moreover, it has previously reported that OGP stimulated cell proliferation via the modulation of CDK2/CyclinA pathway (Fei et al., 2010). Therefore, the increased metabolic activity in OGP treatment groups depict the proliferation effect on hPDLSCs that lead to the increased amount of reductase production.

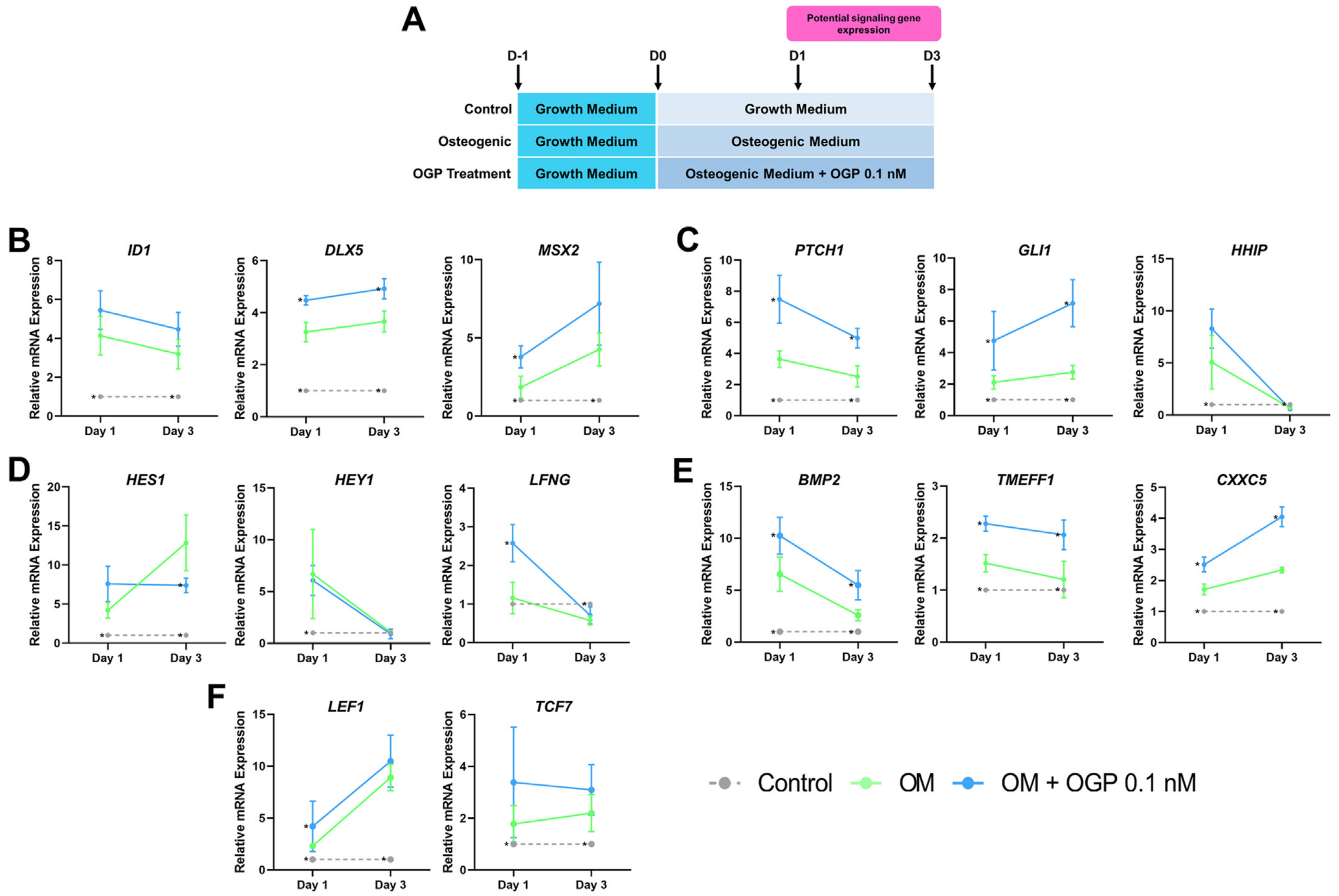

Figure 4. Osteogenic-regulated signaling pathway screening of OGP treatment on hPDLSCs toward osteogenic lineage. (A) Schematic experiment of OGP treatment on hPDLSCs to evaluate osteogenic-related pathways was indicated. (B) Bone morphogenetic protein (BMP), (C) Hedgehog, (D) Notch, (E) transforming growth factor-beta (TGF-β), and (F) Wnt signalings related gene expression were presented. Asterisks beside the dot of experimental group on gene expression results indicated the significant difference between OM and other groups ($p<0.05$).

The increased reduction of alamarBlue™ is known to be correlated by the increased number of live cells (Aslantürk, 2018). Thus, correlating with result, OGP has the mitogenic activity on hPDLSCs, especially at lower dose. Another study also found that OGP promoted proliferation on hPDLSCs (Xu et al., 2009). In contrast, they found 1 nM of OGP has the highest proliferation effect, whereas we found lower concentration showed higher result. However, it has been reported OGP at lower concentration, ranging 0.01–1 nM, is more potent in having proliferation effect in mice-origin bone marrow-derived MSCs (BM-MSCs), osteoblastic MC3T3 E1, and fibroblastic NIH 3T3 cells (Greenberg et al., 1993; Fei et al., 2010). The mitogenic activity of OGP has been also found in other cell types, such as osteogenic ROS 17/2.8 (Bab et al., 1992), rabbit- (Robinson et al., 1995), and human-origin BM-MSCs (Robinson et al., 1995).

Insignificant results on day 7 might be happened due to OGP treatment groups had reached stationary phase which resulted in stable cell growth, while control group was still in log phase. Log and stationary growth phase on hPDLSCs have been mentioned to occur 3–8 days and after 8 days of culture, respectively (Yu et al., 2019). Moreover, stable result on day 7 means that the reductase number in control group was relatively similar with treated groups as they might share similar number of cells. In addition, qualitative live/dead assay result demonstrated that OGP is not toxic to hPDLSCs, since more viable cells were observed than dead cells.

The hPDLSCs have been mentioned to be one of sources for bone regeneration. Previous published reports showed that hPDLSCs treated with scaffolds, such as hydroxyapatite ceramic and titanium implant surface increased osteogenic differentiation (Pizzicannella et al., 2018; Marconi et al., 2021). A thorough exploration of osteogenic differentiation effects on hPDLSCs, previous study showed that small molecule, namely OGP, increases the osteogenic differentiation. As OGP activates MAPK pathways, not only control proliferation, but also the complex cascades of MAPK pathways regulate osteogenic differentiation (Zhang and Liu, 2002).

The mRNA expression of osteogenic-related genes were upregulated in OGP-treated cells. Previous report demonstrated that the Ras activation promoted the expression of *RUNX2*, one of important osteogenic transcription factors (TFs) (Wang et al., 2001). The *RUNX2* phosphorylation and transcriptional activity have been reported to be regulated by the contribution of MAPK (Xiao et al., 2000). In addition, the expression of *OSX*, another important osteogenic TF, was reported to be mediated by MAPK pathways (Celil and Campbell, 2005).

Another study reported that MAPK pathways have the ability in regulating the expression of *COL1*, *ALP*, and *OPN* (Kim et al., 2014). In support of this, one study demonstrated the activation of MAPK can regulate *COL1* (Wang et al., 2001). They also found that *OCN* was upregulated. The increased *OCN* expression is induced because of Runx2 phosphorylation by MAPK (Xiao et al., 2000). Regarding to ALP activity, several studies reported that MAPK can promote ALP activity (Wang et al., 2001; Celil and Campbell, 2005). In addition, one published report found that the increased ALP activity is influenced, specifically, by the activation of ERK of MAPK pathways (Takeuchi et al., 1997). Another study also reported that p38 MAPK takes a contribution in expressing *ALP* (Suzuki et al., 2002).

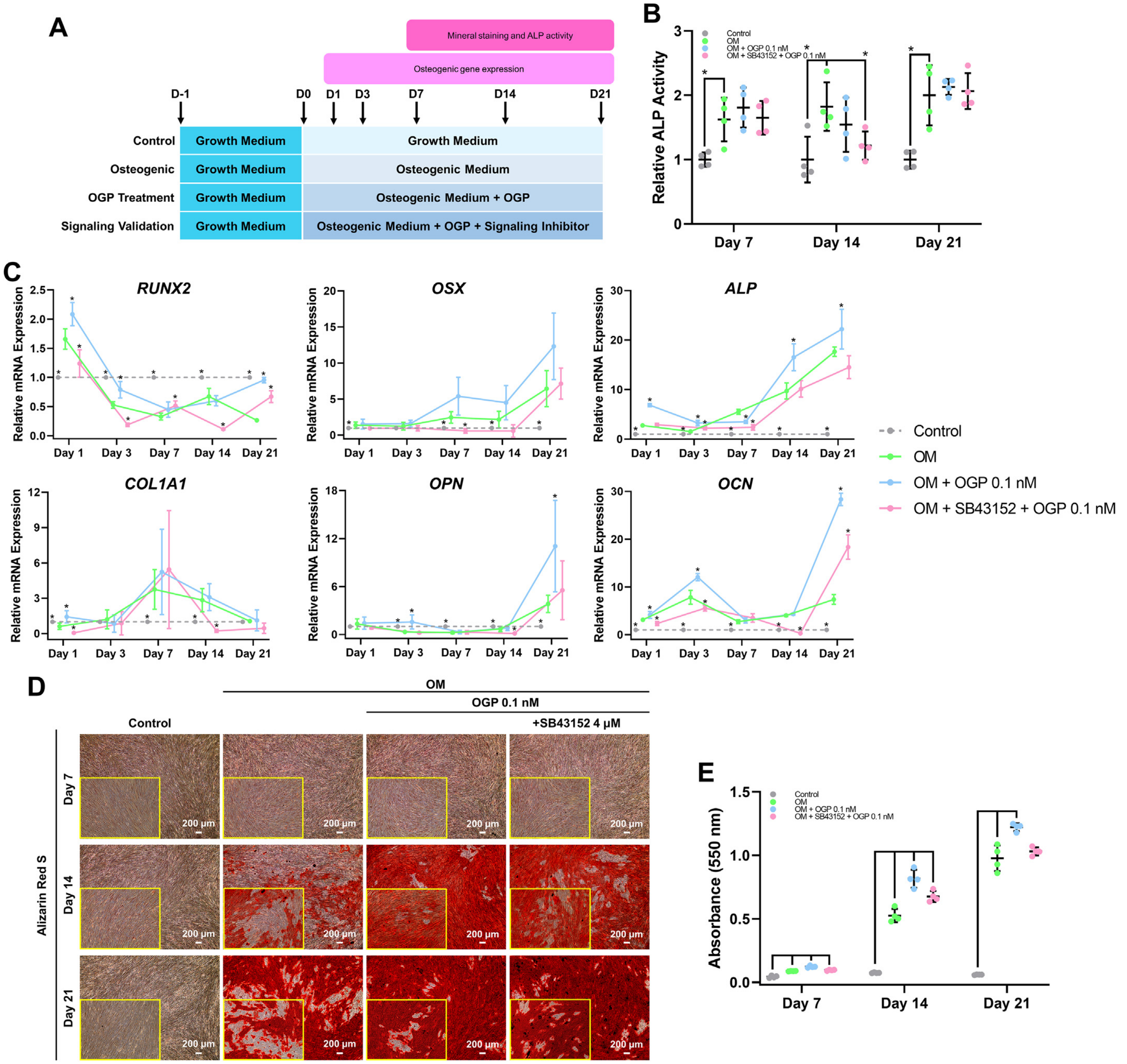

Figure 5. Effect of SB-431542 on OGP-induced osteogenic differentiation in hPDLSCs. (A) Schematic experiment was illustrated. (B) The ALP activity, (C) osteogenic-related marker expression, (D) mineral deposition, and (E) Alizarin Red S staining quantification were presented. Bars and asterisks indicated the significant difference between OM and other groups ($p<0.05$).

Besides all, OGP was reported involving the other molecules and pathways in promoting osteogenic differentiation. One study demonstrated OGP forms stress fibers with the involvement of RhoA/ROCK pathway (Chen et al., 2011). Furthermore, they found that OGP concomitantly enhances osteogenesis and suppresses adipogenesis. It has been proposed that RhoA/ROCK pathway can be mediated by the activation of G_i protein (Huang et al., 2001). In addition, an inducible molecule heme oxygenase-1 (HO-1) is upregulated during OGP treatment and leads to osteogenic stimulation and adipogenic inhibition (Vanella et al., 2010). Interestingly, OGP has also been mentioned to upregulate the expression of long non-coding RNA (lncRNA) *AK141205* and *CXCL13* that is regulated by acetylation modification H4 histone (Li et al., 2015). Corroborate with our findings, these mentioned studies above proved the increased osteogenesis by finding the elevated expression of osteogenic markers during OGP treatment.

As shown in Figure 4, most of target genes were upregulated. These results indicated that OGP regulates the most of evaluated signaling pathways. It is suggested that the pathways were activated via OGP-induced MAPK pathways. Further studies are needed to investigate the phenomenon. However, previous published reports mentioned that MAPK pathway(s) is able to regulate towards TGF-β (Brown et al., 1999; Mori et al., 2004; Tan et al., 2014), BMP (Sapkota et al., 2007), Hedgehog (Riobo et al., 2006; Atkinson et al., 2009; Kakisaka et al., 2012; Liu et al., 2014), Notch (Yamashita et al., 2013), and Wnt pathways (Liao et al., 2006; Bikkavilli et al., 2008). For BMP, it is suggested that the signaling is activated due to the upregulation of *BMP2* in TGF-β signaling. Moreover, the activation of Notch signaling was reported to promote the expression

of TGF-β and BMP ligands, but only BMP signaling can influence Notch signaling-induced osteogenic differentiation (Manokawinchoke et al., 2021). However, it may be assumed that Notch signaling is activated in the earlier time after induction, not until day 3. Thus, it is suggested that Notch signaling is not the main signaling pathway in OGP treatment among the others. In addition, Smad3 of TGF-β signaling pathway can facilitate the β-catenin of Wnt signaling pathway to translocate into the nucleus and start the transcription activity by its coordination with the TFs, T-cell factor(TCF)/lymphoid enhancer binding factor (LEF), to bind with the promoter, thus increases proliferation (Jian et al., 2006). This correlates with our proliferation result, whereas OGP enhances proliferation on hPDLSCs.

Collectively, these results implied that the activation of each signaling is mediated by G_i protein, a protein that OGP activates, through MAPK pathways to further transduce several osteogenic-regulated signaling pathways.

TGF-β signaling is well known for its transduction action through a dual receptor system of transmembrane serine/threonine kinases, namely type 1 and type II receptors (Janssens et al., 2005). Activin receptor-like kinase 5 (ALK5) or TβRI was mentioned to be the main type I receptor to transduce the signal (Larsson et al., 2001). Moreover, ALK5 was suggested to be the only type I receptor of TGF-β signaling pathway in bone cells (Janssens et al., 2005). With this knowledge, SB-431542 was used as it inhibits selectively ALK5 that results in the suppression of Smad2 phosphorylation (Inman et al., 2002).

According to the results, SB-431542 in this study was shown to decrease unremarkably the osteogenic differentiation effect of OGP on ALP activity, osteogenic-related gene expression, and mineralization. Previously, SB-431542 was reported failed to attenuate the Jagged1-induced osteogenesis in human dental pulp stem cells (hDPSCs) (Manokawinchoke et al., 2021). However, the decreased phenomenon can be occurred due to SB-431542 was able to inhibit the osteogenic differentiation during proliferation phase, as TGF-β enhances osteoblast proliferation (Matsunobu et al., 2009). Yet, the action of TGF-β signaling during the matrix maturation and mineralization is to inhibit osteogenic differentiation by regulating the expressions of *COL1A1*, *OCN*, *OPN*, and *ALP* and ALP activity (Breen et al., 1994; Harris et al., 1994). Therefore, inhibiting the signaling pathway during these phases can increase the osteogenic differentiation, implying the unremarked inhibition effect of SB-431542 in hPDLSCs during OGP treatment. Despite of that, it is suggested that OGP mineralizes the cells more not later than the cells treated with SB-431542 due to late differentiation. According to this, the effect of OGP on hPDLSCs partially involves TGF-β signaling toward osteogenic lineage by regulating the ALP activity, osteogenic-related gene expression, and mineral deposition. In addition, further investigation is needed to confirm this present study findings by using a Western blot analysis.

5. Conclusions

OGP is shown as a non-cytotoxic and proliferative peptide on hPDLSCs. Many osteogenic-related signaling pathways were found to be governed to enhance osteogenic differentiation during OGP treatment. In this study, OGP was also found to regulate the osteogenic differentiation partially via TGF-β signaling pathway. With these findings, the authors suggest further use of OGP both in research and clinical application as it has the potential for osteoporosis and/or periodontitis.

Declarations

Author contribution statement

Steven Dwi Purbantoro: Conceived and designed the experiments; Performed the experiments; Analyzed and interpreted the data; Wrote the paper.

Thanaphum Osathanon, Sirirat Nantavisai, Chenphop Sawangmake: Conceived and designed the experiments; Analyzed and interpreted the data; Wrote the paper.

Funding statement

This work was supported by National Research Council of Thailand (NRCT, N41A640135), and Thailand Research Fund (TRF, RTA6180001).

Steven Dwi Purbantoro was supported by the Graduate Scholarship Program for ASEAN countries and The 90th Anniversary Chulalongkorn University Fund.

Chenphop Sawangmake was supported by Chulalongkorn Academic Advancement into Its 2nd Century Project, The Veterinary Stem Cell and Bioengineering Research Unit, The Ratchadaphiseksomphot Endowment Fund, Chulalongkorn University, and the Government Research Fund.

Data availability statement

Data will be made available on request.

Declaration of interests statement

The authors declare no conflict of interest.

Additional information

No additional information is available for this paper.

Acknowledgements

All authors acknowledge Mr. Noppadol Sa-ard-iam, Immunology Research Center, Faculty of Dentistry, Chulalongkorn University for providing on flow cytometry facility, and Assoc. Prof. Sayamon Srisuwatanasagul and K. Earth, Department of Anatomy, Faculty of Veterinary Science, Chulalongkorn University for providing histological purpose facility.

References

Aponte, P.M., Caicedo, A., 2017. Stemness in cancer: stem cells, cancer stem cells, and their microenvironment. Stem Cell. Int. 2017.

Aslantürk, Ö.S., 2018. In Vitro Cytotoxicity and Cell Viability Assays: Principles, Advantages, and Disadvantages, 2. InTech.

Atkinson, P.J., Dellovade, T., Albers, D., Von Schack, D., Saraf, K., Needle, E., Reinhart, P.H., Hirst, W.D., 2009. Sonic Hedgehog signaling in astrocytes is dependent on p38 mitogen-activated protein kinase and G-protein receptor kinase 2. J. Neurochem. 108 (6), 1539–1549.

Bab, I., Gazit, D., Chorev, M., Muhlrad, A., Shteyer, A., Greenberg, Z., Namdar, M., Kahn, A., 1992. Histone H4-related osteogenic growth peptide (OGP): a novel circulating stimulator of osteoblastic activity. EMBO J. 11 (5), 1867–1873.

Bab, I., Gazit, D., Muhlrad, A., Shteyer, A., 1988. Regenerating bone marrow produces a potent growth-promoting activity to osteogenic cells. Endocrinology 123 (1), 345–352.

Bikkavilli, R.K., Feigin, M.E., Malbon, C.C., 2008. p38 mitogen-activated protein kinase regulates canonical Wnt–β-catenin signaling by inactivation of GSK3β. J. Cell Sci. 121 (21), 3598–3607.

Breen, E.C., Ignotz, R.A., McCabe, L., Stein, J.L., Stein, G.S., Lian, J.B., 1994. TGFβ alters growth and differentiation related gene expression in proliferating osteoblasts in vitro, preventing development of the mature bone phenotype. J. Cell. Physiol. 160 (2), 323–335.

Brown, J.D., DiChiara, M.R., Anderson, K.R., Gimbrone, M.A., Topper, J.N., 1999. MEKK-1, a component of the stress (stress-activated protein kinase/c-Jun N-terminal kinase) pathway, can selectively activate Smad2-mediated transcriptional activation in endothelial cells. J. Biol. Chem. 274 (13), 8797–8805.

Celil, A.B., Campbell, P.G., 2005. BMP-2 and insulin-like growth factor-I mediate Osterix (Osx) expression in human mesenchymal stem cells via the MAPK and protein kinase D signaling pathways. J. Biol. Chem. 280 (36), 31353–31359.

Chen, G., Deng, C., Li, Y.-P., 2012. TGF-β and BMP signaling in osteoblast differentiation and bone formation. Int. J. Biol. Sci. 8 (2), 272.

Chen, Z., Wang, X., Shao, Y., Shi, D., Chen, T., Cui, D., Jiang, X., 2011. Synthetic osteogenic growth peptide promotes differentiation of human bone marrow mesenchymal stem cells to osteoblasts via RhoA/ROCK pathway. Mol. Cell. Biochem. 358 (1-2), 221–227.

Dave, J.R., Tomar, G.B., 2018. Dental Tissue– derived mesenchymal stem cells: applications in tissue engineering. Crit. Rev. Biomed. Eng. 46 (5).

Dominici, M., Le Blanc, K., Mueller, I., Slaper-Cortenbach, I., Marini, F., Krause, D., Deans, R., Keating, A., Prockop, D., Horwitz, E., 2006. Minimal criteria for defining multipotent mesenchymal stromal cells. The International Society for Cellular Therapy position statement. Cytotherapy 8 (4), 315–317.

Fei, Q., Guo, C., Xu, X., Gao, J., Zhang, J., Chen, T., Cui, D., 2010. Osteogenic growth peptide enhances the proliferation of bone marrow mesenchymal stem cells from osteoprotegerin-deficient mice by CDK2/cyclin A. Acta Biochim. Biophys. Sin. 42 (11), 801–806.
Gabarin, N., Gavish, H., Muhlrad, A., Chen, Y.C., Namdar-Attar, M., Nissenson, R.A., Chorev, M., Bab, I., 2001. Mitogenic Gi protein-MAP kinase signaling cascade in MC3T3-E1 osteogenic cells: activation by C-terminal pentapeptide of osteogenic growth peptide [OGP (10–14)] and attenuation of activation by cAMP. J. Cell. Biochem. 81 (4), 594–603.
Gay, I.C., Chen, S., MacDougall, M., 2007. Isolation and characterization of multipotent human periodontal ligament stem cells. Orthod. Craniofac. Res. 10 (3), 149–160.
Goldsmith, Z., Dhanasekaran, D., 2007. G protein regulation of MAPK networks. Oncogene 26 (22), 3122–3142.
Greenberg, Z., Chorev, M., Muhlrad, A., Shteyer, A., Namdar, M., Mansur, N., Bab, I., 1993. Mitogenic action of osteogenic growth peptide (OGP) Role of amino and carboxy-terminal regions and charge. Biochim. Biophys. Acta Mol. Cell Res. 1178 (3), 273–280.
Harris, S., Bonewald, L., Harris, M., Sabatini, M., Dallas, S., Feng, J., Ghosh-Choudhury, N., Wozney, J., Mundy, G., 1994. Effects of transforming growth factor β on bone nodule formation and expression of bone morphogenetic protein 2, osteocalcin, osteopontin, alkaline phosphatase, and type I collagen mRNA in long-term cultures of fetal rat calvarial osteoblasts. J. Bone Miner. Res. 9 (6), 855–863.
Houschyar, K.S., Tapking, C., Borrelli, M.R., Popp, D., Duscher, D., Maan, Z.N., Chelliah, M.P., Li, J., Harati, K., Wallner, C., 2019. Wnt pathway in bone repair and regeneration–what do we know so far. Front. Cell Dev. Biol. 6, 170.
Huang, G.-J., Gronthos, S., Shi, S., 2009. Mesenchymal stem cells derived from dental tissues vs. those from other sources: their biology and role in regenerative medicine. J. Dent. Res. 88 (9), 792–806.
Huang, S., Chen, L.-Y., Zuraw, B.L., Richard, D.Y., Pan, Z.K., 2001. Chemoattractant-stimulated NF-κB activation is dependent on the low molecular weight GTPase RhoA. J. Biol. Chem. 276 (44), 40977–40981.
Inman, G.J., Nicolás, F.J., Callahan, J.F., Harling, J.D., Gaster, L.M., Reith, A.D., Laping, N.J., Hill, C.S., 2002. SB-431542 is a potent and specific inhibitor of transforming growth factor-β superfamily type I activin receptor-like kinase (ALK) receptors ALK4, ALK5, and ALK7. Mol. Pharmacol. 62 (1), 65–74.
Janssens, K., ten Dijke, P., Janssens, S., Van Hul, W., 2005. Transforming growth factor-β1 to the bone. Endocr. Rev. 26 (6), 743–774.
Jian, H., Shen, X., Liu, I., Semenov, M., He, X., Wang, X.-F., 2006. Smad3-dependent nuclear translocation of β-catenin is required for TGF-β1-induced proliferation of bone marrow-derived adult human mesenchymal stem cells. Genes Dev. 20 (6), 666–674.
Kakisaka, K., Cazanave, S.C., Werneburg, N.W., Razumilava, N., Mertens, J.C., Bronk, S.F., Gores, G.J., 2012. A hedgehog survival pathway in 'undead'lipotoxic hepatocytes. J. Hepatol. 57 (4), 844–851.
Kim, H.K., Kim, M.-G., Leem, K.-H., 2014. Effects of egg yolk-derived peptide on osteogenic gene expression and MAPK activation. Molecules 19 (9), 12909–12924.
Kuncorojakti, S., Rodprasert, W., Yodmuang, S., Osathanon, T., Pavasant, P., Srisuwatanasagul, S., Sawangmake, C., 2020. Alginate/Pluronic F127-based encapsulation supports viability and functionality of human dental pulp stem cell-derived insulin-producing cells. J. Biol. Eng. 14 (1), 1–15.
Langer, R., Vacanti, J.P., 1993. Tissue engineering. Science 260 (5110), 920–926.
Larsson, J., Goumans, M.J., Sjöstrand, L.J., Van Rooijen, M.A., Ward, D., Levéen, P., Xu, X., Ten Dijke, P., Mummery, C.L., Karlsson, S., 2001. Abnormal angiogenesis but intact hematopoietic potential in TGF-β type I receptor-deficient mice. EMBO J. 20 (7), 1663–1673.
Lems, W.F., Raterman, H.G., 2017. Critical issues and current challenges in osteoporosis and fracture prevention. An overview of unmet needs. Therap. Adv. Musculoskelet. Disease 9 (12), 299–316.
Li, H., Zhang, Z., Chen, Z., Zhang, D., 2015. Osteogenic growth peptide promotes osteogenic differentiation of mesenchymal stem cells mediated by LncRNA AK141205-induced upregulation of CXCL13. Biochem. Biophys. Res. Commun. 466 (1), 82–88.
Liao, G., Tao, Q., Kofron, M., Chen, J.-S., Schloemer, A., Davis, R.J., Hsieh, J.-C., Wylie, C., Heasman, J., Kuan, C.-Y., 2006 Jun. NH2-terminal kinase (JNK) prevents nuclear β-catenin accumulation and regulates axis formation in Xenopus embryos. Proc. Natl. Acad. Sci. USA 103 (44), 16313–16318.
Liu, Z., Li, T., Reinhold, M., Naski, M., 2014. MEK1–RSK2 contributes to hedgehog signaling by stabilizing GLI2 transcription factor and inhibiting ubiquitination. Oncogene 33 (1), 65–73.
Manokawinchoke, J., Pavasant, P., Sawangmake, C., Limjeerajarus, N., Limjeerajarus, C.N., Egusa, H., Osathanon, T., 2019. Intermittent compressive force promotes osteogenic differentiation in human periodontal ligament cells by regulating the transforming growth factor-β pathway. Cell Death Dis. 10 (10), 1–21.
Manokawinchoke, J., Sumrejkanchanakij, P., Boonprakong, L., Pavasant, P., Egusa, H., Osathanon, T., 2020. NOTCH2 participates in Jagged1-induced osteogenic differentiation in human periodontal ligament cells. Sci. Rep. 10 (1), 1–12.
Manokawinchoke, J., Watcharawipas, T., Ekmetipunth, K., Jiamjirachart, M., Osathanon, T., 2021. Dorsomorphin attenuates Jagged1-induced mineralization in human dental pulp cells. Int. Endod. J.
Marconi, G.D., Fonticoli, L., Della Rocca, Y., Rajan, T.S., Piattelli, A., Trubiani, O., Pizzicannella, J., Diomede, F., 2021. Human periodontal ligament stem cells response to titanium implant surface: extracellular matrix deposition. Biology 10 (9), 931.
Matsunobu, T., Torigoe, K., Ishikawa, M., De Vega, S., Kulkarni, A.B., Iwamoto, Y., Yamada, Y., 2009. Critical roles of the TGF-β type I receptor ALK5 in perichondrial formation and function, cartilage integrity, and osteoblast differentiation during growth plate development. Dev. Biol. 332 (2), 325–338.
McCudden, C., Hains, M., Kimple, R., Siderovski, D., Willard, F., 2005. G-protein signaling: back to the future. Cell. Mol. Life Sci. 62 (5), 551–577.
Men, Y., Wang, Y., Yi, Y., Jing, D., Luo, W., Shen, B., Stenberg, W., Chai, Y., Ge, W.-P., Feng, J.Q., 2020. Gli1+ periodontium stem cells are regulated by osteocytes and occlusal force. Dev. Cell 54 (5), 639–654 e636.
Mori, S., Matsuzaki, K., Yoshida, K., Furukawa, F., Tahashi, Y., Yamagata, H., Sekimoto, G., Seki, T., Matsui, H., Nishizawa, M., 2004. TGF-β and HGF transmit the signals through JNK-dependent Smad2/3 phosphorylation at the linker regions. Oncogene 23 (44), 7416–7429.
Nantavisai, S., Pisitkun, T., Osathanon, T., Pavasant, P., Kalpravidh, C., Dhitavat, S., Makjaroen, J., Sawangmake, C., 2020. Systems biology analysis of osteogenic differentiation behavior by canine mesenchymal stem cells derived from bone marrow and dental pulp. Sci. Rep. 10 (1), 1–18.
Nantavisai, S., Rodprasert, W., Pathanachai, K., Wikran, P., Kitcharoenthaworn, P., Smithiwong, S., Archasappawat, S., Sawangmake, C., 2019. Simvastatin enhances proliferation and pluripotent gene expression by canine bone marrow-derived mesenchymal stem cells (cBM-MSCs) in vitro. Heliyon 5 (10), e02663.
Nazir, M.A., 2017. Prevalence of periodontal disease, its association with systemic diseases and prevention. Int. J. Health Sci. 11 (2), 72.
Nowwarote, N., Manokawinchoke, J., Kanjana, K., Fournier, B.P., Sukarawan, W., Osathanon, T., 2020. Transcriptome analysis of basic fibroblast growth factor treated stem cells isolated from human exfoliated deciduous teeth. Heliyon 6 (6), e04246.
Pizzicannella, J., Cavalcanti, M., Trubiani, O., Diomede, F., 2018. MicroRNA 210 mediates VEGF upregulation in human periodontal ligament stem cells cultured on 3Dhydroxyapatite ceramic scaffold. Int. J. Mol. Sci. 19 (12), 3916.
Purwaningrum, M., Jamilah, N.S., Purbantoro, S.D., Sawangmake, C., Nantavisai, S., 2021. Comparative characteristic study from bone marrow-derived mesenchymal stem cells. J. Vet. Sci. 22.
Riobo, N.A., Haines, G.M., Emerson, C.P., 2006. Protein kinase C-δ and mitogen-activated protein/extracellular signal–regulated kinase-1 control GLI activation in Hedgehog signaling. Cancer Res. 66 (2), 839–845.
Robinson, D., Bab, I., Nevo, Z., 1995. Osteogenic growth peptide regulates proliferation and osteogenic maturation of human and rabbit bone marrow stromal cells. J. Bone Miner. Res. 10 (5), 690–696.
Sapkota, G., Alarcón, C., Spagnoli, F.M., Brivanlou, A.H., Massagué, J., 2007. Balancing BMP signaling through integrated inputs into the Smad1 linker. Mol. Cell 25 (3), 441–454.
Sawangmake, C., Nantavisai, S., Osathanon, T., Pavasant, P., 2016. Osteogenic differentiation potential of canine bone marrow-derived mesenchymal stem cells under different β-glycerophosphate concentrations in vitro. Thai J. Vet. Med. 46 (4), 617–625.
Sawangmake, C., Nowwarote, N., Pavasant, P., Chansiripornchai, P., Osathanon, T., 2014a. A feasibility study of an in vitro differentiation potential toward insulin-producing cells by dental tissue-derived mesenchymal stem cells. Biochem. Biophys. Res. Commun. 452 (3), 581–587.
Sawangmake, C., Pavasant, P., Chansiripornchai, P., Osathanon, T., 2014b. High glucose condition suppresses neurosphere formation by human periodontal ligament-derived mesenchymal stem cells. J. Cell. Biochem. 115 (5), 928–939.
Seo, B.-M., Miura, M., Gronthos, S., Bartold, P.M., Batouli, S., Brahim, J., Young, M., Robey, P.G., Wang, C.Y., Shi, S., 2004. Investigation of multipotent postnatal stem cells from human periodontal ligament. Lancet 364 (9429), 149–155.
Song, I.S., Han, Y.S., Lee, J.-H., Um, S., Kim, H.Y., Seo, B.M., 2015. Periodontal ligament stem cells for periodontal regeneration. Curr. Oral Health Rep. 2 (4), 236–244.
Spreafico, A., Frediani, B., Capperucci, C., Leonini, A., Gambera, D., Ferrata, P., Rosini, S., Di Stefano, A., Galeazzi, M., Marcolongo, R., 2006. Osteogenic growth peptide effects on primary human osteoblast cultures: potential relevance for the treatment of glucocorticoid-induced osteoporosis. J. Cell. Biochem. 98 (4), 1007–1020.
Suzuki, A., Guicheux, J., Palmer, G., Miura, Y., Oiso, Y., Bonjour, J.-P., Caverzasio, J., 2002. Evidence for a role of p38 MAP kinase in expression of alkaline phosphatase during osteoblastic cell differentiation. Bone 30 (1), 91–98.
Takeuchi, Y., Suzawa, M., Kikuchi, T., Nishida, E., Fujita, T., Matsumoto, T., 1997. Differentiation and transforming growth factor-β receptor down-regulation by collagen-α2β1 integrin interaction is mediated by focal adhesion kinase and its downstream signals in murine osteoblastic cells. J. Biol. Chem. 272 (46), 29309–29316.
Tan, Y., Xu, Q., Li, Y., Mao, X., Zhang, K., 2014. Crosstalk between the p38 and TGF-β signaling pathways through TβRI, TβRII and Smad3 expression in plancental choriocarcinoma JEG-3 cells. Oncol. Lett. 8 (3), 1307–1311.
Tang, R., Wei, F., Wei, L., Wang, S., Ding, G., 2014. Osteogenic differentiated periodontal ligament stem cells maintain their immunomodulatory capacity. J. Tissue Eng. Regen. Med. 8 (3), 226–232.
Tonetti, M.S., Jepsen, S., Jin, L., Otomo-Corgel, J., 2017. Impact of the global burden of periodontal diseases on health, nutrition and wellbeing of mankind: a call for global action. J. Clin. Periodontol. 44 (5), 456–462.
Vanella, L., Kim, D.H., Asprinio, D., Peterson, S.J., Barbagallo, I., Vanella, A., Ikehara, S., Kappas, A., Abraham, N.G., 2010. HO-1 expression increases mesenchymal stem cell-derived osteoblasts but decreases adipocyte lineage. Bone 46 (1), 236–243.
Wang, F.-S., Wang, C.-J., Huang, H.-J., Chung, H., Chen, R.-F., Yang, K.D., 2001. Physical shock wave mediates membrane hyperpolarization and Ras activation for osteogenesis in human bone marrow stromal cells. Biochem. Biophys. Res. Commun. 287 (3), 648–655.
Xiao, G., Jiang, D., Thomas, P., Benson, M.D., Guan, K., Karsenty, G., Franceschi, R.T., 2000. MAPK pathways activate and phosphorylate the osteoblast-specific transcription factor, Cbfa1. J. Biol. Chem. 275 (6), 4453–4459.

Xu, L., Huang, J., Xiang, X-r, 2009. Osteoblastic growth peptide regulates proliferation of human periodontal ligament cells and alkaline phosphatase activities. J. Clin. Rehabilitative Tissue Eng. Res. 13 (24), 4627–4631.

Yamashita, A.S., Geraldo, M.V., Fuziwara, C.S., Kulcsar, M.A.V., Friguglietti, C.U.M., da Costa, R.B., Baia, G.S., Kimura, E.T., 2013. Notch pathway is activated by MAPK signaling and influences papillary thyroid cancer proliferation. Transl. Oncol. 6 (2), 197-IN122.

Yu, B., Li, Q., Zhou, M., 2019. LPS-induced upregulation of the TLR4 signaling pathway inhibits osteogenic differentiation of human periodontal ligament stem cells under inflammatory conditions. Int. J. Mol. Med. 43 (6), 2341–2351.

Zhang, W., Liu, H.T., 2002. MAPK signal pathways in the regulation of cell proliferation in mammalian cells. Cell Res. 12 (1), 9–18.
