# Supplementary material for: Molecular Regulators for the Wnt/β-Catenin Signalling Pathway for Bone Remodelling in Periodontitis: A Scoping Review
Source: Int J Mol Sci. 2026 Oct 14;27(20):9117. doi: 10.3390/ijms27209117 (PMC13705578; doi:10.3390/ijms27209117)
Supplement: Supplementary file 1 [file ijms-27-09117-s001.zip › Supplementary Data/Papers/in vitro/The impact of Wnt signalling and hypoxia on osteogenic and cementogenic differentiation in human periodontal ligament cells.pdf]

MOLECULAR MEDICINE REPORTS 14: 4975-4982, 2016

The impact of Wnt signalling and hypoxia on osteogenic and cementogenic differentiation in human periodontal ligament cells

SHUIGEN LI[1,2*], JIN SHAO[1,3*], YINGHONG ZHOU[3,4], THOR FRIIS[3], JIANGWU YAO[2], BIN SHI[1] and YIN XIAO[1,3,4]

[1]State Key Laboratory Breeding Base of Basic Science of Stomatology (Hubei-MOST) & Key Laboratory for Oral Biomedical Ministry of Education, School and Hospital of Stomatology, Wuhan University, Wuhan, Hubei 430079; [2]Department of Oral Biology and Biomaterials, Xiamen Stomatology Research Institute, Xiamen, Fujian 361000, P.R. China; [3]Institute of Health and Biomedical Innovation (IHBI); [4]The Australia-China Centre for Tissue Engineering and Regenerative Medicine (ACCTERM), Queensland University of Technology, Brisbane, Queensland 4059, Australia

Received March 16, 2016; Accepted August 26, 2016

DOI: 10.3892/mmr.2016.5909

Abstract. Cementum is a periodontal support tissue that is directly connected to the periodontal ligament. It shares common traits with bone tissues, however, unlike bone, the cementum has a limited capacity for regeneration. As a result, following damage the cementum rarely, if ever, regenerates. Periodontal ligament cells (PDLCs) are able to differentiate into osteoblastic and cementogenic lineages according to specific local environmental conditions, including hypoxia, which is induced by inflammation or activation of the Wnt signalling pathway by local loading. The interactions between the Wnt signalling pathway and hypoxia during cementogenesis are of particular interest to improve the understanding of periodontal tissue regeneration. In the present study, osteogenic and cementogenic differentiation of PDLCs was investigated under hypoxic conditions in the presence and absence of Wnt pathway activation. Protein and gene expression of the osteogenic markers type 1 collagen (COL1) and runt-related transcription factor 2 (RUNX2), and cementum protein 1 (CEMP1) were used as markers for osteogenic and cementogenic differentiation, respectively. Wnt signalling activation inhibited cementogenesis, whereas hypoxia alone did not affect PDLC differentiation. However, hypoxia reversed the inhibition of cementogenesis that resulted from overexpression of Wnt signalling. Cross-talk between hypoxia and Wnt signalling pathways was, therefore, demonstrated to be involved in the differentiation of PDLCs to the osteogenic and cementogenic lineages. In summary, the present study suggests that the differentiation of PDLCs into osteogenic and cementogenic lineages is partially regulated by the Wnt signalling pathway and that hypoxia is also involved in this process.

Correspondence to: Professor Bin Shi, State Key Laboratory Breeding Base of Basic Science of Stomatology (Hubei-MOST) & Key Laboratory for Oral Biomedical Ministry of Education, School and Hospital of Stomatology, Wuhan University, 237 Luoyu Road, Wuhan, Hubei 430079, P.R. China
E-mail: shibin_dentist@126.com

Professor Yin Xiao, Institute of Health and Biomedical Innovation (IHBI), Queensland University of Technology, 60 Musk Avenue, Kelvin Grove, Brisbane, Queensland 4059, Australia
E-mail: yin.xiao@qut.edu.au

[*]Contributed equally

Key words: Wnt, hypoxia, osteogenesis, cementogenesis, periodontal ligament

Introduction

Cementum is a central component of periodontal tissues with a very limited capacity for regeneration (1). This lack of regenerative potential of an important functional periodontal tissue represents a major challenge for dental clinicians. Periodontal ligament cells (PDLCs) can differentiate towards both osteoblastic and cementogenic lineage cells, which are responsible for bone and cementum formation, respectively (2). Cementum has many biochemical features in common with bone, but the primary distinction is that cementum lacks vascularization and a Haversian canal system (3). Compared with bone, which undergoes continuous remodelling throughout life, cementum is a more quiescent tissue (4). Cementoblasts express classical osteogenic markers, including alkaline phosphatase, type I collagen (COL1), runt-related transcription factor 2 (RUNX2) and noncollagenous proteins including bone sialoprotein and osteocalcin (1,5). Cementum protein 1 (CEMP1) and cementum attached protein (CAP) are also specific markers of cementum (6-8). There is debate as to whether or not cementoblasts and osteoblasts have a common precursor (9), and the different properties of these two cell types, including endogenous gene expression and the response to the extracellular environment, remain subjects of investigation.

The canonical Wnt/β-catenin pathway has a complex role in mineral tissue development and regeneration (10,11), with different functions depending on cell type and differentiation stage. Wnt promotes bone formation by enhancing both the proliferation and differentiation of bone marrow stromal cells (BMSCs), however is reported to be down-regulated during the terminal mineralisation stages (12). Wnt is also expressed by PDLCs and has a role in cell proliferation (13). The impact of Wnt on cementoblasts has been extensively investigated, however the results appear to be contradictory: A study using an immortalised murine cementoblast cell line (OCCM-30), demonstrated that the canonical Wnt signalling pathway inhibited cementoblast differentiation (14), whereas another study, using primary PDLCs cultured in osteogenic induction medium, demonstrated an increase in cementogenic markers following the activation of the canonical Wnt signalling (15). Potentially, the differences observed in these studies are due to the different cell types used (immortalised vs. primary cells), and the local environment of PDLC differentiation. During chronic periodontitis and orthodontic treatment, the cementum exhibits severe damage and its regeneration is greatly restricted compared with alveolar bone (16).

Local hypoxia may be an important factor, triggered by inflammation and over-loading (17). Hypoxia inducible factor-1α (HIF-1α), a principal mediator of hypoxia, interacts with the Wnt signalling pathway in a complex pattern (18). It has been previously reported that HIF-1α enhances Wnt signalling in undifferentiated cells and promotes the proliferation of stem cells (19,20). It has also been reported that hypoxia blocks Wnt/β-catenin signalling by interfering with the function of the endoplasmic reticulum, which prevents protein secretion in tumours (21). Furthermore, there is *in vivo* evidence from neural stem cells suggesting that hypoxia activates the transcription of β-catenin and the β-catenin signalling cascade by increasing the expression of lymphoid enhancer-binding factor 1 (LEF-1) and T-cell factor 1 (TCF-1), which are the nuclear targets of β-catenin (22). However, the role of HIF-1α in the regulation of Wnt signalling in differentiated cells remains unclear.

In the present study, primary human PDLCs in osteogenic culture conditions were used to investigate the interaction between hypoxia and Wnt signalling, and how this affects cementogenesis. Overexpression of Wnt signalling molecules was demonstrated to inhibit cementogenesis, and hypoxia rescued this inhibition by blocking the canonical Wnt signalling pathway. However, hypoxia and Wnt signalling promoted the osteogenic differentiation of PDLCs.

Materials and methods

Isolation and culture of human PDLCs (hPDLCs). Isolation and culture of hPDLCs was performed according to previously published protocols (23). Teeth were obtained from healthy patients (18-25 years old) undergoing third molar extraction surgery. Informed consent was provided by all patients involved and the research protocol was approved by the Human Ethics Committees of Queensland University of Technology (Brisbane, Australia). Briefly, periodontal ligament tissues were separated from the middle third of the root surface using a scalpel and were cultured in a T25 flask in Dulbecco's modified Eagle's medium (DMEM; Thermo Fisher Scientific, Inc., Waltham, MA, USA) supplemented with 10% v/v foetal bovine serum (FBS; Thermo Fisher Scientific, Inc.) and 50 U/ml penicillin and 50 mg/ml streptomycin (Thermo Fisher Scientific, Inc.) at 37˚C in a humidified CO_2 incubator. The osteogenic medium was supplemented with 10^{-8} M dexamethasone, 8 mM β-glycerol phosphate and 50 µg/ml ascorbic acid. Following incubation for 5 days, the medium was changed and the outgrown cells were passaged at ~80% confluence. Cells at passages 2-5 were used for subsequent experiments.

Hypoxia-mimicking culture conditions and activation of the Wnt signalling pathway. Dimethyloxalylglycine (DMOG, Sigma-Aldrich; Merck Millipore, Darmstadt, Germany) was added into the culture medium of the PDLCs at a final concentration of 1 mM to mimic a hypoxic environment, as described previously (24). To activate the Wnt signalling pathway, Wnt3a conditioned medium (Wnt-CM) was prepared using a genetically modified murine cell line overexpressing L Wnt-3a (ATCC CRL-2647; American Type Culture Collection, Manassas, VA, USA). Cells were cultured according to the supplier's guidelines in a T75 flask with ATCC-formulated DMEM, supplemented with 10% v/v FBS and 0.4 mg/ml G418 (Sigma-Aldrich; Merck Millipore) to select Wnt3a positive cells. The conditioned medium (CM) was prepared by splitting the cells 1:10 in 10 ml culture medium without G418 and incubating for 4 days. The first batch of CM was removed and filter sterilised, and 10 ml fresh culture medium added. The cells were cultured for a further 3 days before a second batch of CM was collected. The working CM consisted of a 1:1 mixture of the two batches.

Cell proliferation assay. PDLCs were seeded in 96-well plates at $4x10^3$ cells per well and cultured in either normal or hypoxic medium (1 mM DMOG), with or without the addition of Wnt-CM. On days 1 and 3, 20 µl of 3-(4,5-dimethylthiazol-2-yl)-2,5-diphenyltetrazolium bromide (MTT) solution (0.5 mg/ml; Sigma-Aldrich; Merck Millipore) was added to each well and incubated for 4 h at 37˚C. The supernatants were removed and replaced with 100 µl of dimethyl sulphoxide to solubilise the MTT-formazan product. Absorbances were measured at a wavelength of 495 nm using a microplate reader (Benchmark Plus; Bio-Rad Laboratories, Inc., Hercules, CA, USA).

Reverse transcription-quantitative polymerase chain reaction (RT-qPCR). hPDLCs were seeded in 6-well plates cultured in normoxic or hypoxic medium, with or without Wnt-CM, for 3 days. Total RNA was extracted in 1 ml TRIzol® Reagent (Thermo Fisher Scientific, Inc.) per well. Complementary DNA was synthesised using a DyNAmo™ cDNA Synthesis Kit (Finnzymes; Thermo Fisher Scientific, Inc.) following the manufacturer's protocols. qPCR was performed on an ABI 7500 Real-Time PCR system (Applied Biosystems; Thermo Fisher Scientific, Inc.) using SYBR Green detection reagent (Thermo Fisher Scientific, Inc.) according to a two-step protocol (initial denature at 95˚C for 2 min, followed by 45 cycles of 5 sec at 95˚C, 10 sec at 60˚C and 15 sec at 72˚C). Transcription levels of COL1, RUNX2 and CEMP1 were assayed and normalised against the housekeeping gene glyceraldehyde 3-phosphate dehydrogenase (GAPDH), using the primers listed in Table I. Each reaction was performed in triplicate and the mean cycle quantification (Cq)

MOLECULAR MEDICINE REPORTS 14: 4975-4982, 2016

Table I. Oligonucleotides sequences.

Gene	Forward primer (5'→3')	Reverse primer (5'→3')
CEMP1	GGGCACATCAAGCACTGACAG	CCCTTAGGAAGTGGCTGTCCAG
COL1	CTGACTGGAAGAGCGGAGAG	GAGTGGGGAACACACAGGTC
RUNX2	ACCAAGAAGGCACAGACAGAAGC	AGGATTGTGTCTGCCTGGGATC
GAPDH	TCAGCAATGCCTCCTGCAC	TCTGGGTGGCAGTGATGGC

CEMP1, cementum protein 1; COL1, type I collagen; RUNX2, runt-related transcription factor 2; GAPDH, glyceraldehyde 3-phosphate dehydrogenase.

value of each target gene was normalised against the Cq value of GAPDH, and the relative expression calculated using the following formula: $2^{(-\text{normalised average Cqs})}$ x10^4 (25).

Western blotting. Whole cell lysates for western blot analysis were harvested in 250 μl cell lysis buffer (2 mM Tris-HCl, pH 7.5, 15 mM NaCl, 0.1 mM Na_2EDTA, 0.1 mM EGTA, 0.1% Triton, 0.25 mM sodium pyrophosphate, 0.1 mM β-glycerophosphate, 0.1 mM Na_3VO_4, 0.1 μg/ml leupeptin). Protein lysates (15 μg per lane) were separated by 10% sodium dodecyl sulphate-polyacrylamide gel electrophoresis, and transferred onto nitrocellulose membranes (Pall Life Sciences, Port Washington, NY, USA). Membranes were blocked for 1 h at room temperature in Odyssey blocking buffer (cat. no. 927-40000; LI-COR, Inc., Lincoln, NE, USA), then incubated overnight at 4˚C with primary antibodies against β-catenin (1:1,000, rabbit anti-human/rat; cat. no. 9581; Cell Signaling Technology, Inc., Danvers, MA, USA); CEMP1 (1:1,000, rabbit polyclonal antibody; cat. no. ab134231; Abcam, Cambridge, UK); COL1 (1:1,000, rabbit anti-human/rat; cat. no. ab34710; Abcam); RUNX2 (1:1,000, rabbit polyclonal antibody; cat. no. sc-10758; Santa Cruz Biotechnology, Inc., Dallas, TX, USA); HIF-1α (1:1,000, mouse monoclonal antibody; cat. no. sc-13515; Santa Cruz Biotechnology, Inc.); and α-Tubulin (1:2,000, rabbit anti-human/rat; cat. no. ab15246; Abcam). The membranes were incubated with anti-mouse/rabbit fluorescently labelled secondary antibodies (P/N 925-32211 or P/N 925-68070; LI-COR, Inc.) at 1:10,000 dilutions for 1 h at room temperature. Protein bands were visualised using the Odyssey Infrared Imaging System (LI-COR, Inc.). The relative intensity of protein bands compared with α-Tubulin was quantified using Image J software version 1.47 (National Institutes of Health, Bethesda, MD, USA).

Statistical analysis. Data are presented as the mean ± standard deviation of 3 independent experiments (with each experiment containing 3 technical replicates). Analysis was performed using SPSS software version 22.0 (SPSS Inc., Chicago, IL, USA). Nonparametric Wilcoxon test was carried out to distinguish the differences between different groups. Comparison tests were performed as indicated. $P<0.05$ was considered to indicate a statistically significant difference.

Results

Confirmation of cellular hypoxia. To determine whether PDLCs responded to the hypoxia-mimicking culture conditions of DMOG, protein expression levels of HIF-1α were examined by western blot analysis. This revealed a distinct increase in HIF-1α expression in cells exposed to 1 mM DMOG, with densitometric quantification of the bands demonstrating a statistically significant increase compared with the normoxic control condition (P=0.011; Fig. 1A). This confirmed that DMOG generates hypoxia-like culture conditions for PDLCs. β-catenin was used to examine the efficiency of Wnt signalling induction by Wnt-CM. Compared with culture in DMEM, a significant increase in β-catenin protein expression was observed when PDLCs were cultured in Wnt-CM (P=0.014; Fig. 1B).

Effects of hypoxia and Wnt on cell proliferation. The proliferation rate of PDLCs cultured in normoxic and hypoxia-like conditions with/without Wnt-CM was determined by MTT assay. A significantly higher rate of proliferation was observed in the normoxia plus Wnt-CM condition compared with hypoxia plus Wnt-CM condition on days 1 and 3 (P=0.038 and P=0.029, respectively; Fig. 2), indicating that the effect of Wnt signalling on cell proliferation was inhibited by hypoxia. When cultured in osteogenic medium, hypoxic conditions significantly inhibited the proliferation rates of PDLCs in Wnt stimulated and non-stimulated groups on days 1 and 3 ($P<0.05$; Fig. 3).

Hypoxia combined with Wnt3a conditioned medium promotes osteogenic differentiation of PDLCs. RT-qPCR and western blot analysis were performed to determine the effects of hypoxia and Wnt signalling on osteogenic differentiation of PDLCs. Cells were cultured under normoxic and hypoxia-like conditions, with and without Wnt-CM. There were no significant differences between the protein (Fig. 4A) or mRNA expression levels (Fig. 4B) of COL1 and RUNX2 in PDLCs under normoxic and hypoxic conditions. However, when cells were cultured in Wnt-CM and hypoxic conditions, there was a significant up-regulation of the protein and mRNA expression levels of COL1 (P=0.021 and P=0.019, respectively; Fig. 4A and B, respectively) and RUNX2 (P=0.031 and P=0.029, respectively; Fig. 4A and B, respectively) compared with the normoxic cultures. In this context, as COL1 and RUNX2 are markers of osteogenic differentiation, hypoxic cells in which Wnt signalling has been induced appear to have a stronger osteogenic capacity than normoxic cells.

Furthermore, when cells were cultured in osteogenic medium, COL1 and RUNX2 were expressed and their

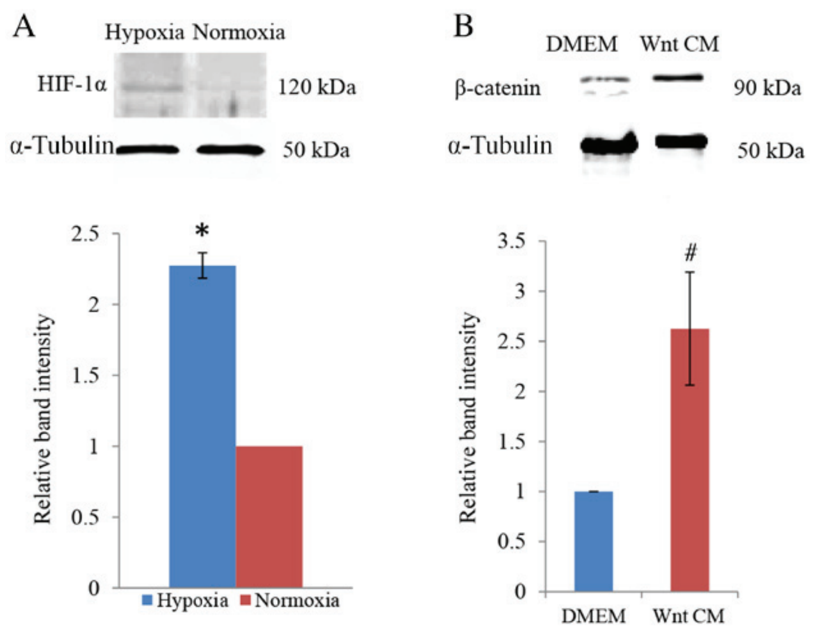

Figure 1. Confirmation of cellular hypoxia and activation of Wnt signalling. (A) Western blot analysis of HIF-1α, with quantification relative to α-Tubulin, revealed an increase of HIF-1α protein in periodontal ligament cells cultured with 1 mM dimethyloxalylglycine (hypoxia-mimicking conditions). (B) Western blot analysis of β-catenin, with quantification relative to α-Tubulin, demonstrated a significant increase of expression in the Wnt3a CM. *P<0.05 vs. normoxia. #P<0.05 vs. DMEM. DMEM, Dulbecco's modified Eagle's medium; CM, conditioned medium; HIF-1α, hypoxia inducible factor-1α.

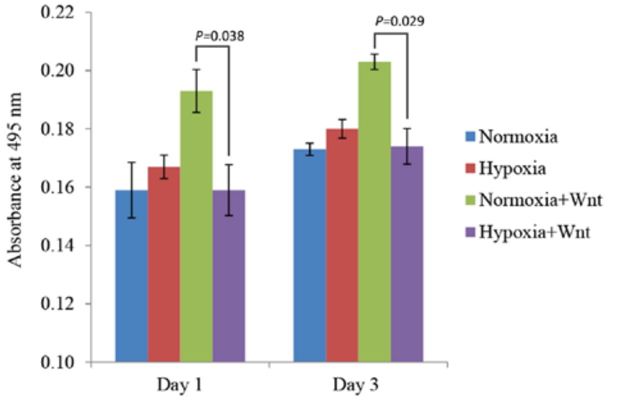

Figure 2. Cell proliferation of PDLCs in non-osteogenic medium in normoxic and hypoxic (1 mM dimethyloxalylglycine) conditions, with or without Wnt-CM, was assessed by 3-(4,5-dimethylthiazol-2-yl)-2,5-diphenyltetrazolium bromide (MTT) assay. In the Wnt-CM groups there was a significant difference in PDLC proliferation in normoxic conditions compared with hypoxia. PDLC, periodontal ligament cells; CM, conditioned medium.

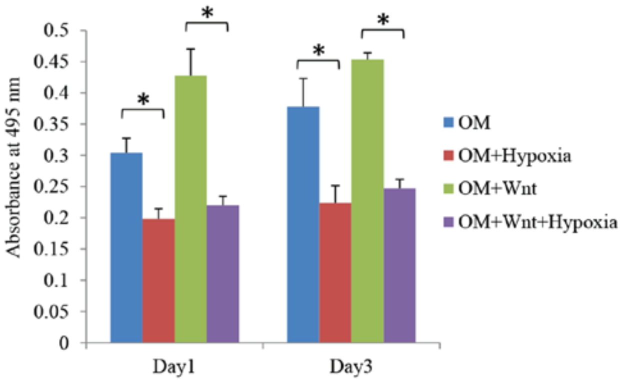

Figure 3. DMOG-induced hypoxia inhibits the proliferation of PDLCs in the OM and the Wnt-CM groups. Cell proliferation of PDLCs in OM in normoxic and hypoxic (1 mM dimethyloxalylglycine) conditions, with or without Wnt-conditioned medium, was assessed by 3-(4,5-dimethylthiazol-2-yl)-2,5-diphenyltetrazolium bromide (MTT) assay. *P<0.05, comparison indicated by brackets. DMOG, dimethyloxalylglycine; PDLC, periodontal ligament cells; OM, osteogenic medium.

expression was further increased in response to hypoxia compared with normoxic levels, with and without Wnt-CM (P<0.05; Fig. 4C and D).

Wnt signalling inhibits cementogenic differentiation of PDLCs. The cementum specific marker, CEMP1, was used to evaluate cementogenic differentiation of PDLCs. PDLCs were cultured in normal growth medium or in osteogenic medium to induce differentiation, then protein expression levels were analysed by western blot and mRNA expression levels by RT-qPCR. Wnt-CM stimulation inhibited the protein expression levels of CEMP1 in undifferentiated PDLCs compared with the levels without Wnt-CM stimulation in normoxic and hypoxic conditions (P=0.039 and P=0.042, respectively; Fig. 5A). mRNA expression levels of CEMP1 were also decreased by Wnt-CM in undifferentiated PDLCs in normoxic and hypoxic conditions compared with the levels without Wnt-CM stimulation (P=0.034 and P=0.018, respectively; Fig. 5B). Additionally, an increase in protein expression levels of β-catenin in PDLCs following osteogenic differentiation was observed (data not shown). When cultured in osteogenic medium, CEMP1 protein expression levels were significantly higher in hypoxic conditions compared with normoxia, both without and with Wnt (P=0.014 and P=0.025, respectively; Fig. 5C). This observation was further confirmed by analysis of mRNA transcription levels (Fig. 5D). Notably, the expression of

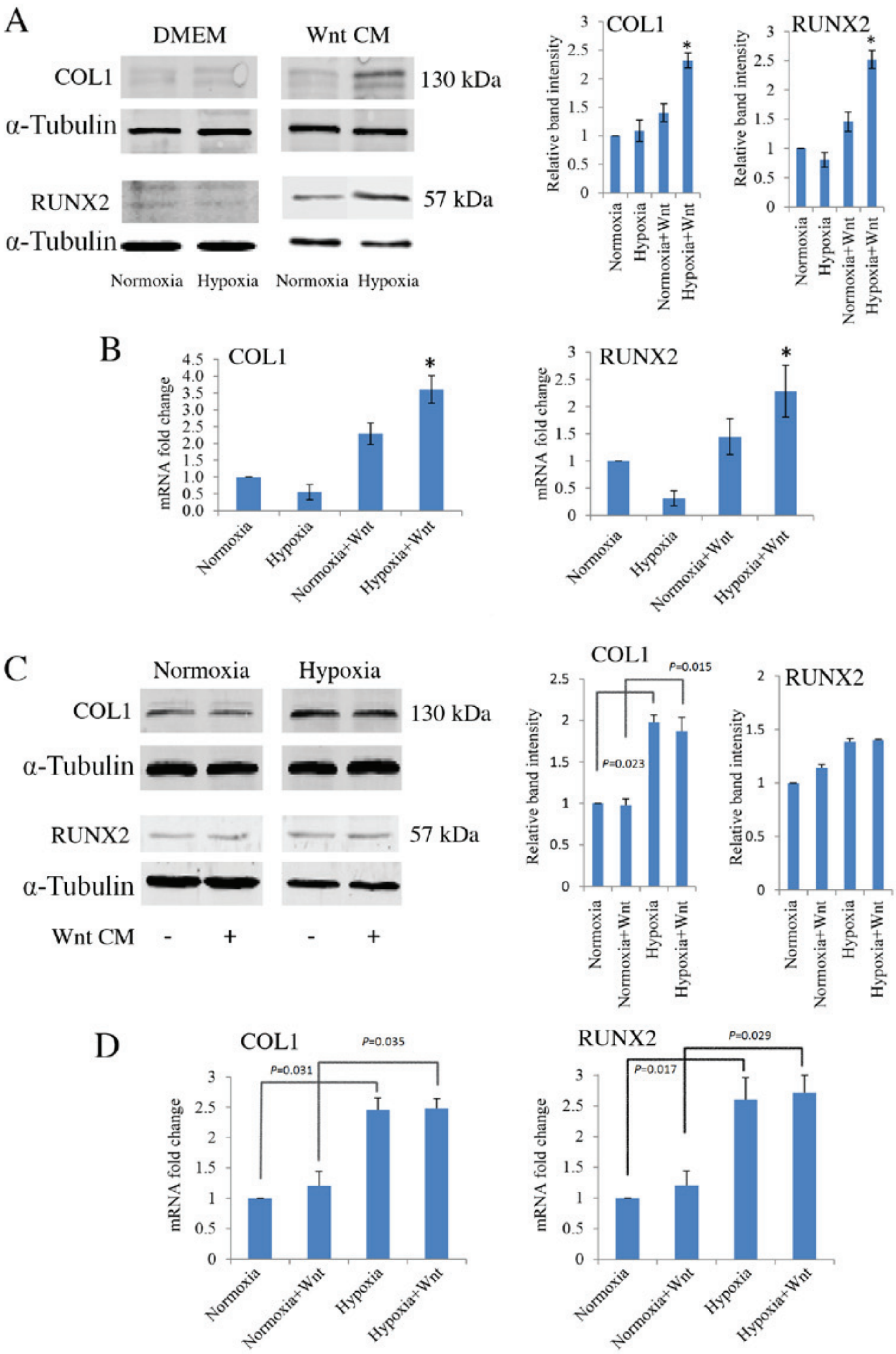

Figure 4. Effect of hypoxia and Wnt on the expression of osteogenic markers, COL1 and RUNX2, in PDLCs cultured in DMEM and osteogenic medium, in normoxic and hypoxic (1 mM dimethyloxalylglycine) conditions, with or without Wnt-CM. (A) Western blot analysis with quantification relative to α-tubulin, and (B) RT-qPCR analysis, with quantification relative to GAPDH, of COL1 and RUNX2 expression levels in PDLCs cultured in DMEM or Wnt-CM. (C) Western blot analysis, with quantification relative to α-tubulin, and (D) RT-qPCR analysis, with quantification relative to GAPDH, of COL1 and RUNX2 expression levels in PDLCs cultured in osteogenic medium. *P<0.05 vs. normoxia group. PDLC, periodontal ligament cells; DMEM, Dulbecco's modified Eagle's medium; CM, conditioned medium; COL1, type I collagen; RUNX2, runt-related transcription factor 2; GAPDH, glyceraldehyde 3-phosphate dehydrogenase.

CEMP1 is negatively associated with β-catenin expression, an active element of canonical Wnt signalling. These results suggest that Wnt signalling inhibits cementogenesis (Fig. 5A and B), whereas hypoxia promotes cementogenesis by down-regulating Wnt signalling (Fig. 5C and D).

Discussion

Cementum consists of the cellular intrinsic fibre cementum (CIFC) layer, located at the tip of the root, and the acellular extrinsic fibre cementum (AEFC) layer, located at the upper

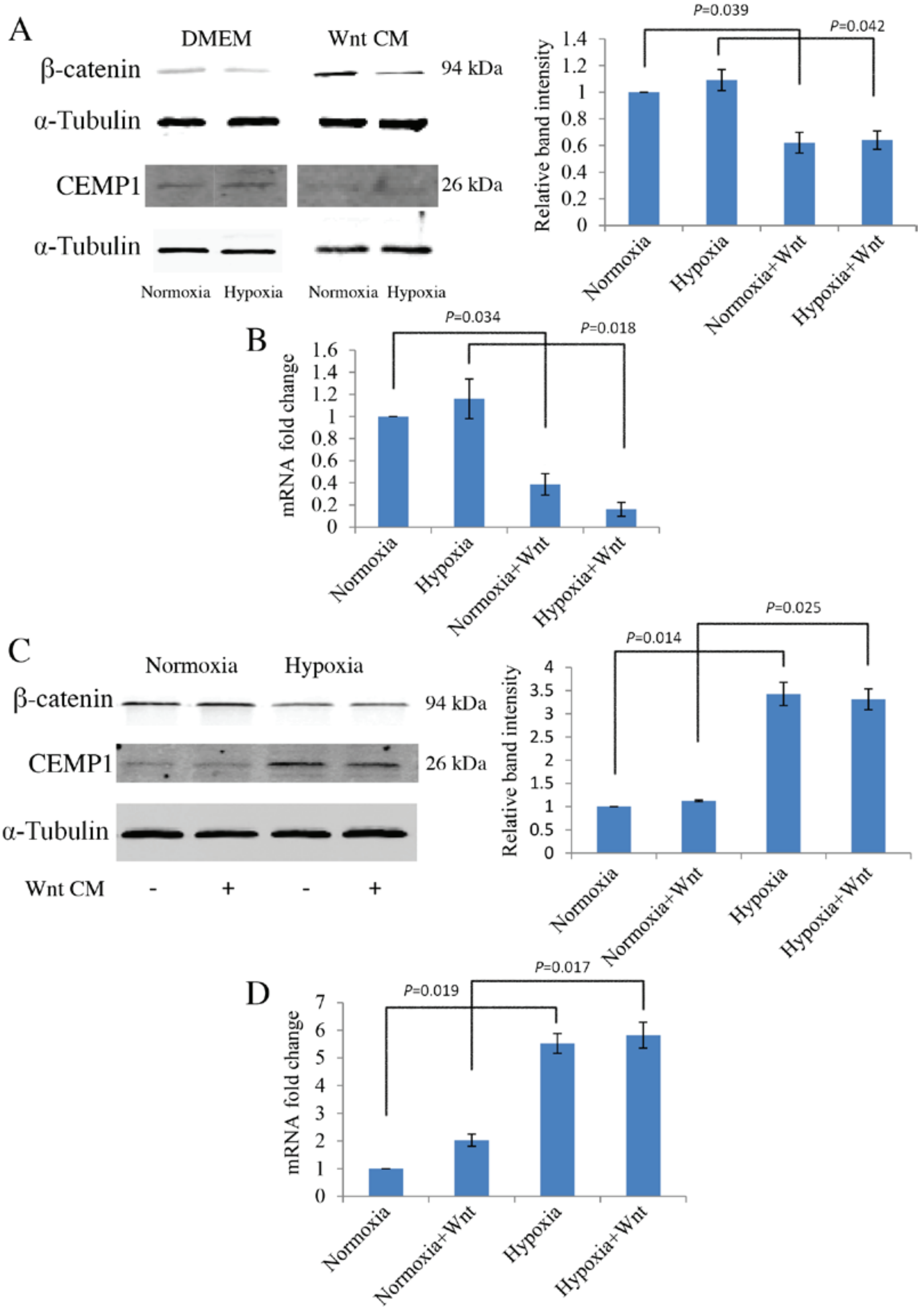

Figure 5. Effect of hypoxia and Wnt on the expression of cementogenic marker, CEMP1, in PDLCs in normoxic and hypoxic (1 mM dimethyloxalylglycine) conditions, with or without Wnt-CM. (A) Western blot analysis of CEMP1 protein expression levels in DMEM, with quantification relative to α-Tubulin. (B) RT-qPCR analysis of CEMP1 mRNA expression levels in DMEM, with quantification relative to GAPDH. (C) Western blot analysis of CEMP1 protein expression levels in OM, with quantification relative to α-Tubulin. (D) RT-qPCR of CEMP1 mRNA expression levels in OM, with quantification relative to GAPDH. PDLC, periodontal ligament cells; OM, osteogenic medium; DMEM, Dulbecco's modified Eagle's medium; CM, conditioned medium; CEMP1, cementum protein 1; GAPDH, glyceraldehyde 3-phosphate dehydrogenase.

two-thirds of the root (9). CIFC is continuously deposited at the tip of the root to compensate for normal physiological occlusive abrasion; however, it is the AEFC that predominantly contributes to periodontal attachment. Therefore, regenerated cementum would ideally resemble the AEFC (26). However, compared with the CIFC, the regenerative capacity of the AEFC is significantly lower (26). Cementum regeneration must also include reattachment of the periodontal ligament to the cementum. From a biochemical perspective, CIFC and bone share certain common features but are distinct tissues; as opposed to bone, CIFC has no lamellar organization, blood vessels or nerve innervation. The CIFC and AEFC are both

formed by cementoblasts, however, the specific mechanisms resulting in the production of these distinct types of cementum are of significant research interest. The cementoblasts that form the CIFC become embedded within the matrix they secrete, and a resemblance to bone formation is apparent. However, the reason for the lack of embedded cementoblasts within the AEFC, which resembles the tooth enamel formed by non-embedded ameloblasts, remains unclear. These are properties that distinguish cementum tissues from bone, and also CIFC and AEFC within the cementum.

It has been proposed that hypoxia maintains the stem-like properties of PDLCs by enhancing the expression of pluripotency markers (27). In the present study, an *in vitro* model was used to demonstrate that Wnt signalling inhibits PDLC differentiation towards a cementoblast lineage, instead promoting differentiation towards an osteoblastic lineage. Therefore, the regeneration of periodontal tissue cannot be realized by simply activating Wnt signalling (15). To improve the understanding the specific function of Wnt signalling and hypoxia in the regeneration of CIFC and AEFC, a site-specific *in vivo* model should be established to investigate the regeneration of CIFC and AEFC as separate phenomena.

Previous studies have demonstrated that the Wnt signalling pathway promotes cementogenesis in PDLCs cultured in osteogenic media (15). In the present study, PDLCs were cultured in non-osteogenic medium, revealing that Wnt signalling inhibits cementogenic differentiation of naive PDLCs. However, when PDLCs were cultured in osteogenic medium Wnt signalling was spontaneously up-regulated and any further stimulation of Wnt by the addition of Wnt3a conditioned medium had limited effects on β-catenin activation, and the expression of cementogenic marker, CEMP1. The association between hypoxia and the Wnt signalling pathway has been intensively investigated, resulting in a numerous contradictory conclusions. For example, it has been proposed that hypoxia can activate canonical Wnt signalling by up-regulating the expression of LEF-1 and TCF-1 in embryonic stem cells, thereby increasing proliferation (19). Hypoxia normally inhibits the formation of β-catenin-TCF-4 complex and transcriptional activity, however, in a certain microenvironment, HIF-1α can compete with TCF-4 for direct binding of β-catenin to promote cell survival and tumourigenesis (28). Another study suggests that HIF-1α can inhibit β-catenin signalling by interfering with human arrest defective 1, which would otherwise acetylate and activate β-catenin (29). The results of the present study indicated that in PDLCs, hypoxia inhibits β-catenin, as a marker of Wnt signalling, which contributes to the understanding of the cell- and tissue-specific effects of hypoxia and Wnt signalling. Unpublished data from our lab has also demonstrated that undifferentiated PDLCs have low intrinsic Wnt signalling and that CEMP1 expression decreases in spite of reduced β-catenin activity under hypoxia-like conditions. By contrast, PDLCs have relatively high Wnt signalling expression following osteogenic induction (data not shown). In this context, hypoxia inhibited β-catenin expression and promoted the expression of CEMP1. The role of Wnt signalling in cementogenesis as demonstrated in the present study is to inhibit cementogenic differentiation.

The Wnt signalling pathway has been considered as a therapeutic target for bone and mineral tissue regeneration. However, the results of the current and previous studies suggest that Wnt signalling has widely varying effects depending on the tissue, and in terms of regeneration of specific mineralised tissues, it is necessary to initially establish the precise function of Wnt signalling in the local environment.

PDLCs can differentiate to osteoblasts and cementoblasts, thus, the present study suggests that cementogenic and osteogenic differentiation of PDLCs may originate from the different local environments of the periodontal tissues. For example, an infection of the root surface may lead to an increased inflammatory response and induce hypoxic conditions. This, in turn, may affect the capacity of naive PDLCs to differentiate, resulting in inhibition of cementogenesis following periodontal treatment. Hypoxia increases CEMP1 expression in differentiated PDLCs, which may be crucial for cementum regeneration.

References

1. Diekwisch TG: The developmental biology of cementum. Int J Dev Biol 45: 695-706, 2001.
2. Freeman E: Peridontium. In: Ten Cate, A.R. ed. Mosby-Year Book, Inc, St. Louis, MO, USA, 1994.
3. Cool SM, Forwood MR, Campbell P and Bennett MB: Comparisons between bone and cementum compositions and the possible basis for their layered appearances. Bone 30: 386-392, 2002.
4. Dimitriou R, Jones E, McGonagle D and Giannoudis PV: Bone regeneration: Current concepts and future directions. BMC Med 9: 66, 2011.
5. Hirata A, Sugahara T and Nakamura H: Localization of runx2, osterix, and osteopontin in tooth root formation in rat molars. J Histochem Cytochem 57: 397-403, 2009.
6. Thomas HF: Root formation. Int J Dev Biol 39: 231-237, 1995.
7. Huang X, Bringas P Jr, Slavkin HC and Chai Y: Fate of HERS during tooth root development. Dev Biol 334: 22-30, 2009.
8. Cao Z, Zhang H, Zhou X, Han X, Ren Y, Gao T, Xiao Y, de Crombrugghe B, Somerman MJ and Feng JQ: Genetic evidence for the vital function of Osterix in cementogenesis. J Bone Miner Res 27: 1080-1092, 2012.
9. Bosshardt DD: Are cementoblasts a subpopulation of osteoblasts or a unique phenotype? J Dent Res 84: 390-406, 2005.
10. Westendorf JJ, Kahler RA and Schroeder TM: Wnt signaling in osteoblasts and bone diseases. Gene 341: 19-39, 2004.
11. Krishnan V, Bryant HU and Macdougald OA: Regulation of bone mass by Wnt signaling. J Clin Invest 116: 1202-1209, 2006.
12. Li X, Liu P, Liu W, Maye P, Zhang J, Zhang Y, Hurley M, Guo C, Boskey A, Sun L, *et al*: Dkk2 has a role in terminal osteoblast differentiation and mineralized matrix formation. Nat Genet 37: 945-952, 2005.
13. Rooker SM, Liu B and Helms JA: Role of Wnt signaling in the biology of the periodontium. Dev Dyn 239: 140-147, 2010.
14. Nemoto E, Koshikawa Y, Kanaya S, Tsuchiya M, Tamura M, Somerman MJ and Shimauchi H: Wnt signaling inhibits cementoblast differentiation and promotes proliferation. Bone 44: 805-812, 2009.
15. Han P, Wu C, Chang J and Xiao Y: The cementogenic differentiation of periodontal ligament cells via the activation of Wnt/β-catenin signalling pathway by Li+ ions released from bioactive scaffolds. Biomaterials 33: 6370-6379, 2012.
16. Grzesik WJ and Narayanan AS: Cementum and periodontal wound healing and regeneration. Crit Rev Oral Biol Med 13: 474-484, 2002.
17. Ng KT, Li JP, Ng KM, Tipoe GL, Leung WK and Fung ML: Expression of hypoxia-inducible factor-1α in human periodontal tissue. J Periodontol 82: 136-141, 2011.
18. Semenza GL: HIF-1 and human disease: One highly involved factor. Genes Dev 14: 1983-1991, 2000.
19. Mazumdar J, O'Brien WT, Johnson RS, LaManna JC, Chavez JC, Klein PS and Simon MC: O2 regulates stem cells through Wnt/β-catenin signalling. Nat Cell Biol 12: 1007-1013, 2010.
20. Grayson WL, Zhao F, Izadpanah R, Bunnell B and Ma T: Effects of hypoxia on human mesenchymal stem cell expansion and plasticity in 3D constructs. J Cell Physiol 207: 331-339, 2006.

21. Verras M, Papandreou I, Lim AL and Denko NC: Tumor hypoxia blocks Wnt processing and secretion through the induction of endoplasmic reticulum stress. Mol Cell Biol 28: 7212-7224, 2008.
22. Varela-Nallar L, Rojas-Abalos M, Abbott AC, Moya EA, Iturriaga R and Inestrosa NC: Chronic hypoxia induces the activation of the Wnt/β-catenin signaling pathway and stimulates hippocampal neurogenesis in wild-type and APPswe-PS1ΔE9 transgenic mice in vivo. Front Cell Neurosci 8: 17, 2014.
23. Zhou Y, Wu C and Xiao Y: The stimulation of proliferation and differentiation of periodontal ligament cells by the ionic products from Ca7Si2P2O16 bioceramics. Acta Biomater 8: 2307-2316, 2012.
24. Jaakkola P, Mole DR, Tian YM, Wilson MI, Gielbert J, Gaskell SJ, von Kriegsheim A, Hebestreit HF, Mukherji M, Schofield CJ, *et al*: Targeting of HIF-alpha to the von Hippel-Lindau ubiquitylation complex by O2-regulated prolyl hydroxylation. Science 292: 468-472, 2001.
25. Bookout AL and Mangelsdorf DJ: Quantitative real-time PCR protocol for analysis of nuclear receptor signaling pathways. Nucl Recept Signal 1: e012, 2003.
26. Bosshardt DD and Selvig KA: Dental cementum: The dynamic tissue covering of the root. Periodontol 2000 13: 41-75, 1997.
27. Zhou Y, Fan W and Xiao Y: The effect of hypoxia on the stemness and differentiation capacity of PDLC and DPC. Biomed Res Int 2014: 890675, 2014.
28. Kaidi A, Williams AC and Paraskeva C: Interaction between beta-catenin and HIF-1 promotes cellular adaptation to hypoxia. Nat Cell Biol 9: 210-217, 2007.
29. Lim JH, Chun YS and Park JW: Hypoxia-inducible factor-1alpha obstructs a Wnt signaling pathway by inhibiting the hARD1-mediated activation of beta-catenin. Cancer Res 68: 5177-5184, 2008.
