# Supplementary material for: Molecular Regulators for the Wnt/β-Catenin Signalling Pathway for Bone Remodelling in Periodontitis: A Scoping Review
Source: Int J Mol Sci. 2026 Oct 14;27(20):9117. doi: 10.3390/ijms27209117 (PMC13705578; doi:10.3390/ijms27209117)
Supplement: Supplementary file 1 [file ijms-27-09117-s001.zip › Supplementary Data/Papers/in vitro/TNF-α regulates the osteogenic differentiation of bone morphogenetic factor 9 adenovirus-transduced rat follicle stem cells via Wnt signaling.pdf]

MOLECULAR MEDICINE REPORTS 22: 3141-3150, 2020

TNF-α regulates the osteogenic differentiation of bone morphogenetic factor 9 adenovirus-transduced rat follicle stem cells via Wnt signaling

XINYUE LI[1-3], GE REN[1-3], CHANGJUN CAI[1-3], XIA YANG[1-3], LI NIE[1-3], XUEQIN JING[1-3] and CONGHUA LI[1-3]

[1]Department of Outpatients, Stomatological Hospital of Chongqing Medical University, Chongqing Medical University; [2]Chongqing Key Laboratory of Oral Diseases and Biomedical Sciences, Stomatological Hospital of Chongqing Medical University, Chongqing Medical University; [3]Chongqing Municipal Key Laboratory of Oral Biomedical Engineering of Higher Education, Stomatological Hospital of Chongqing Medical University, Chongqing Medical University, Chongqing 401147, P.R. China

Received January 29, 2020; Accepted June 1, 2020

DOI: 10.3892/mmr.2020.11439

Abstract. Periodontitis is a chronic infectious disease that alters the cellular microenvironment and promotes bone absorption. Bone morphogenetic protein 9 (BMP9) serves an important role in proliferation and differentiation, and tumor necrosis factor-alpha (TNF-α) is an important contributor to bone resorption. The present study aimed to investigate the effect of osteogenic differentiation in the presence of BMP9 and TNF-α in rat follicle stem cells (rDFCs). rDFCs were transfected with adenoviruses expressing BMP9 (AdBMP9) and the expression levels of important proteins [BMP9, β-catenin, glycogen synthase kinase 3β (GSK3β), phosphorylated-GSK3β, calcium/calmodulin dependent protein kinase II and nemo like kinase] were determined using western blotting. The effect of osteogenesis was analyzed using reverse transcription-quantitative PCR, in addition to alkaline phosphatase, Alizarin Red S, and hematoxylin and eosin staining methods. The results of the present study revealed that TNF-α activated the canonical Wnt signaling pathway and suppressed osteogenesis. High concentrations of Dickkopf 1 (DKK1) reduced the osteogenic differentiation of AdBMP9-transduced rDFCs, whereas low concentrations of DKK1 promoted BMP9-induced bone formation, which was discovered to partially act via the canonical and non-canonical Wnt signaling pathways. In conclusion, the findings of the present study suggested that the enhanced promoting effect of BMP9 alongside the treatment with low concentrations of DKK1 may be useful for treating periodontitis bone absorption.

Correspondence to: Professor Conghua Li, Chongqing Municipal Key Laboratory of Oral Biomedical Engineering of Higher Education, Stomatological Hospital of Chongqing Medical University, Chongqing Medical University, 426 Songshibei Street, Chongqing 401147, P.R. China
E-mail: liconghua@hospital.cqmu.edu.cn

Key words: rat follicle stem cells, bone morphogenetic protein, tumor necrosis factor-α, Wnt signaling, osteogenic differentiation

Introduction

Periodontitis is a chronic inflammatory disease, of which the initiating factor, bacterial plaque, causes irreparable damage to the tooth-supporting structures (1,2). Tissue regeneration is currently the most effective therapeutic method for severe periodontitis; however, the treatment effect is limited (3). In 1992, Wise *et al* (4) successfully cultured rDFCs for the first time. Since, the stem cell properties of self-renewal and multi-directional differentiation potential in DFCs have been verified (5-8). Bone morphogenetic protein (BMP) has been discovered to facilitate the differentiation of stem cells into osteoblasts, which subsequently increased osteogenesis (9,10). Moreover, BMP9 induces cell differentiation (11-14). Gene transfection using adenovirus vectors has previously demonstrated efficacy (15). In a previous study, TNF-α, a proinflammatory cytokine, lead to the absorption and destruction of periodontal bone and collagen fibers (16). Notably, Mukai *et al* (17) discovered that TNF-α suppressed BMP2-induced osteogenic differentiation.

Wnt signaling is involved in bone formation and bone resorption via two major molecular signaling pathways: The β-catenin-dependent canonical signaling pathway and the Ca^{2+}-dependent non-canonical Wnt pathway (18). Several studies have indicated that the canonical Wnt signaling served a two-directional regulatory role in osteogenic differentiation; for example, Qiu *et al* (19) discovered that the expression levels of osteogenesis-related factors alkaline phosphatase (ALP) and Runt-related transcription factor 2 (RUNX2) were upregulated in human mesenchymal stem cells via the activation of the canonical Wnt signaling pathway; and Jansen *et al* (20) conducted a tensile experiment on human pre-osteoblasts and discovered that the elevated concentrations of β-catenin at the early osteogenesis stagewere markedly reduced at the moderate and advanced osteogenesis stages, which subsequently induced the formation of a large amount of mineralized tissues (20).

These findings suggested that the canonical Wnt signaling pathway may suppress osteogenesis at the late differentiation stage. Additionally, numerous studies have also reported that osteogenic induction or an inflammatory microenvironment prompted canonical Wnt signaling to negatively regulate cell osteogenic differentiation; for instance, Liu *et al* (21) discovered that suppressing the Wnt/β-catenin pathway and upregulating the Wnt/Ca^{2+} non-canonical pathway enhanced the osteogenic differentiation of periodontal ligament stem cells under inflammation; and Xiang *et al* (22) further discovered that the Wnt5a protein promoted osteogenic differentiation and interacted with the BMP2 signaling pathway (22). However, the Wnt canonical signaling pathway was reported to be inhibited by Dickkopf 1 (DKK1) (23-25). The Wnt/β-catenin pathway was also identified to be involved in osteoclastogenesis, osteoclast differentiation and bone resorption by influencing the expression levels of osteoprotegerin (24,25).

The present study aimed to determine the effects of TNF-α on osteogenic differentiation and investigate the mechanisms of the Wnt signaling pathway in BMP9 adenovirus (AdBMP9)-transduced rDFCs.

Materials and methods

Cell culture and adenovirus-mediated transfection. Sprague Dawley rats (n=10; age, 6-8 days old; male:female, 1:1; weight, 20-30 g) were purchased from the Experimental Animal Center of Chongqing Medical University (Chongqing, China). The rats were housed in cages under a 12-h light/dark cycle with free access to food and water, at a temperature of 22-25˚C and 50-60% humidity; the rats were monitored every day. Rats were sacrificed with 35 mg/kg sodium pentobarbital (1%) followed by decapitation then soaked in 20% ethanol for 10 min; respiratory arrest was confirmed following decapitation. Dental sac tissues were obtained from the first and second mandibular molars, and rDFCs were obtained as previously described (6). rDFCs were cultured in DMEM (Invitrogen; Thermo Fisher Scientific, Inc.) supplemented with 10% FBS (Gibco; Thermo Fisher Scientific, Inc.), 100 U/ml penicillin and 100 mg/ml streptomycin (Sigma-Aldrich; Merck KGaA), and maintained at 37˚C in a humidified atmosphere containing 5% CO_2.

Flow cytometric analysis and multi-lineage differentiation were performed for the characterization of mesenchymal stem cells, as previously described (6). The identification of rDFCs and the determination of appropriate concentrations of TNF-α (10 ng/ml; GenScript) and DKK1 (0.1 and 0.4 μg/ml; Sino Biological, Beijing China) were determined based on previously published studies (6,8).

At 80% confluence (passage 3), cells were digested by 0.25% trypsin, re-suspended and re-cultured at 37˚C in a humidified atmosphere containing 5% CO_2 for 24 h. AdBMP9 (MOI=100) and polybrene (1 μm/ml) were added to the adherent culture according to previously described methods (6,14). Following the addition of AdBMP9, cells were incubated for 4 h, then 2 ml complete medium was added followed by incubation for 24 h at 37˚C in an atmosphere containing 5% CO_2. The green fluorescence was observed under a fluorescence microscope (magnification, x40) AdGFP(MOI=100) was used as the vector control. Cells were subsequently collected for further experimentation. Ad-BMP9 and Ad-GFP were provided by Dr Tong-Chuan He (Molecular Oncology Laboratory, University of Chicago Medical Centre, USA).

ALP staining. A total of 5x10^4 cells/well were plated into 24-well plates and cultured in a conventional osteogenic medium for 7 days at 37˚C (Beyotime Institute of Biotechnology). After transfection with AdBMP9, TNF-α (10 ng/ml, GenScript) and DKK1 (0.1 or 0.4 μg/ml; Sino Biological Inc.) were added into the medium and cultured at 37˚C in an atmosphere containing 5% CO_2. The medium was refreshed every three days. At 7 days post-culture, cells were fixed with 4% paraformaldehyde at room temperature for 2 h and washed with distilled, deionized water. Stained cells were observed under a light microscope (magnification, x100). ALP staining was performed using an ALP staining kit (Beyotime Institute of Biotechnology), according to the manufacturer's protocol.

Alizarin Red S staining. Cells (5x10^4 cells/well) were plated into 24-well plates and cultured in a conventional osteogenic medium at 37˚C for 14 days. TNF-α (10 ng/ml) and DKK1 (0.1 or 0.4 μg/ml) were added into the medium and cultured at 37˚C in an atmosphere containing 5% CO_2. The medium was refreshed every three days. Following 14 days of culture, mineralized matrix nodules were stained for calcium precipitation using Alizarin Red S. Briefly, cells were fixed with 4% paraformaldehyde at 20˚C for 15 min and then washed with distilled, deionized water. Subsequently, the cells were stained with Alizarin Red S (Beyotime Institute of Biotechnology) at room temperature for 30 min, followed by three washes with deionized water. Deionized water was added into each well to prevent cells from drying before they were observed under a light microscope (magnification, x40).

Reverse transcription-quantitative PCR (RT-qPCR). Total RNA was extracted using TRIzol® reagent (Invitrogen; Thermo Fisher Scientific, Inc.) and reverse transcribed into cDNA using the iScript™ cDNA Synthesis kit (Bio-Rad Laboratories, Inc.) according to the manufacturer's protocol. Reverse transcription was performed using the following temperature protocol: 85˚C for 2 min and 37˚C for 30 min. Subsequently, qPCR was performed using SYBR-Green Master mix (Thermo Fisher Scientific, Inc.). The sequences of the primers used for qPCR are presented in Table I. Primers used for qPCR amplification are presented in Table I, and β-actin was used as the internal control. The following thermocycling conditions were used for qPCR: 94˚C for 2 min; followed by 35 cycles of denaturation at 95˚C for 30 sec, annealing at 64˚C for 30 sec, and extension 72˚C for 30 sec. Relative gene expression was calculated using the $2^{-\Delta\Delta Cq}$ method (26) and normalized to the internal reference gene β-actin.

Western blotting. Cells were washed in precooled PBS twice and total protein was extracted from cells using RIPA lysis buffer (Pierce; Thermo Fisher Scientific, Inc.), containing protease inhibitor, phosphatase inhibitor and 1 mM PMSF. The cells were incubated on ice for 30 min and the lysate was subsequently collected in a 1.5 ml tube and centrifuged at 10,000 x g for 10 min at 4˚C. The supernatant was transferred

Table I. Primer sequences for reverse transcription-quantitative PCR.

Gene	Primer sequence (5'→3')
β-actin	F: TGCTATGTTGCCCTAGACTTCG
	R: GTTGGCATAGAGGTCTTTACG
Collagen type 1 α1	F: CAAGAAGTCCCTGCTCCTCCA
	R: GGGAGGTCTTGGTGGTTTTGTAT
Cyclin D1	F: GTTCATTTCCAACCCACCCTC
	R: CGTTGTGCGGTAGCAGGAGA
Alkaline phosphatase	F: GGCACCTGCCTTACCAACTCT
	R: GTTGTGGTGTAGCTGGCCCTTA
Osteocalcin	F: TTTCTGCTCACTCTGCTGACC
	R: CAGCACAACTCCTTCCTACCA
Runt-related transcription factor 2	F: CAGCTGCTTAGACGCTGGATT
	R: AGGCGGGACACCTACTCTCATA
c-Myc	F: CAGCTGCTTAGACGCTGGATT
	R: GTAGAAATACGGCTGCACCGA
β-catenin	F: GAGCCTGCCATCTGTGCTCT
	R: ACGCAAAGGTGCATGATTTG
Calcium/calmodulin-dependent protein kinase type II α chain	F: AACTGACCAGGCACAGACG
	R: CCCTAATGTCTTCCGCCTGC
Serine/threonine protein kinase NLK	F: TGGGCAACAACAGCCATATTT
	R: ATGGTGCGCCTTAACTGTAGC

F, forward; R, reverse.

into 1.5 ml new tubes, followed by the addition of 5X loading buffer (volume 1:4), which was then boiled for 5-8 min and stored at -20˚C until use. Total protein was quantified using a bicinchoninic acid protein assay kit (Beyotime Institute of Biotechnology) and proteins (30 µg) were separated via 8% SDS-PAGE. The separated proteins were subsequently transferred via the wet method (6,8,14) to PVDF membranes and blocked with PBS containing 0.2% Tween-20 and 5% non-fat milk at 4˚C for 2 h. The membranes were then incubated overnight at 4˚C with the following primary antibodies overnight at 4˚C: Anti-BMP9 (cat. no. ab35088; 1:1,000; Abcam), anti-β-catenin (cat. no. ab6302; 1:1,000; Abcam), anti-phosphorylated (p)-glycogen synthase kinase 3β (GSK3β; cat. no. ab131097; 1:1,000; Abcam), anti-GSK3β (cat. no. ab93926; 1:1,000; Abcam), anti-calcium/calmodulin dependent protein kinase II (CaMKII; cat. no. ab52476; 1:1,000; Abcam), anti-nemo like kinase (NLK; cat. no. ab26050; 1:1,000; Abcam) and anti-β-actin (cat. no. 4970; 1:1,000; Cell Signaling Technology, Inc.). Following the primary antibody incubation, the membranes were incubated at room temperature for 2 h with a HRP-conjugated goat anti-rabbit (cat. no. ab205718; 1:3,000; Abcam) and anti-mouse (cat. no. 58802; 1:3,000; Cell Signaling Technology, Inc.). Protein bands were visualized using an ECL chemiluminescent substrate kit (Beyotime Institute of Biotechnology) and densitometric analysis was performed using ImageJ software (version 1.52; National Institutes of Health).

Animal experimental design. The present study was approved by the Institutional Animal Experimental Ethics Committee of Chongqing Medical University (Chongqing, China). A total of 20 4-week-old Sprague Dawley rats (male; weight, 200-250 g) were purchased from the Experimental Animal Center of Chongqing Medical University. The rats were housed in cages under a 12-h light/dark cycle with free access to food and water, at 22-25˚C and 50-60% humidity. Animal health and behavior was monitored every day. The rats were randomly divided into 4 groups (n=5 per group): i) AdBMP9 group; ii) AdBMP9 + 0.1 µg/ml DKK1; iii) AdBMP9 + 0.4 µg/ml DKK1; and iv) AdBMP9 + TNF-α and received implants respectively as detailed below. All cells had been previously co-cultured for 7 days with hydroxyapatite (40 mg/ml; Biomaterials Engineering Research Center of Sichuan University), a scaffold material, at 37˚C (14).

Rats were anaesthetized using an intraperitoneal injection of 35 mg/kg sodium pentobarbital (1%). The periapical alveolar bone of the first and second molars was exposed by surgery and an osseous defect (2x3x3 mm) was created. Cells got 7-day-co-cultured with hydroxyapatite. The defect of was filled with rDFCs-hydroxyapatite in accordance with the grouping of each rat ($4x10^6$ cells; 20 mg; injection site, periapical defect; age of rats at injection, 8 weeks) then covered with a collagen membrane (5x5 mm; Bopei Biotech Co., Ltd). The wound was closed with interrupted sutures. The 4th week following surgery was chosen as the humane endpoint; although the rats were in a good mental state and exhibited good locomotor activity, the scientific goal of the research has been achieved, thus, it was of little significance to continue the experiment. The rats were transferred from the rearing room into the testing room at ≥30 min prior to the initiation

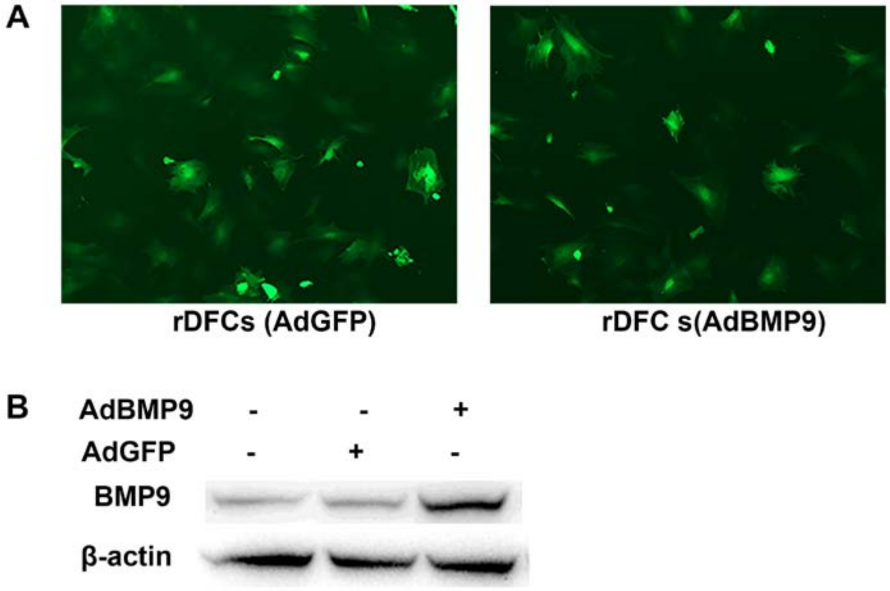

Figure 1. Ad-BMP9-transfected rDFCs. (A) The cell morphology and fluorescence activity of rDFCs transfected with AdBMP9 or AdGFP for 24 h (magnification, x200). (B) Western blotting was used to analyze the expression levels of BMP9 in rDFCs following transfection with AdBMP9. rDFCs, rat follicle stem cells; Ad, adenovirus; BMP9, bone morphogenetic protein 9.

of the experiment. Subsequently, rats were anesthetized with an intraperitoneal injection of 35 mg/kg sodium pentobarbital (1%) and sacrificed by decapitation; respiratory arrest and death were confirmed.

The alveolar bones were extracted and fixed in 4% paraformaldehyde for 48 h at room temperature. Subsequently, sections (1 mm^3) were stained with hematoxylin for 5 min at room temperature and eosin for 3 min at room temperature. Stained sections were observed under a light microscope (magnification, x100).

Statistical analysis. Data are presented as the mean ± SD of ≥3 independent experiments. Statistical analysis was performed using SPSS 21.0 software (IBM Corp.). Data from the different groups were compared and analyzed using a one-way ANOVA, followed by a Bonferroni's post hoc test. $P<0.05$ was considered to indicate a statistically significant difference.

Results

TNF-α suppresses the osteogenic differentiation of AdBMP9-transduced rDFCs. rDFCs are polygonal or fusiform in morphology (6,8,14). rDFCs were transfected with AdBMP9 or AdGFP and green fluorescence was detected at 24 h post-transfection (Fig. 1A). In addition, following AdBMP9 transfection for 24 h, the expression levels of BMP9 were upregulated compared with the AdGFP and blank groups (Fig. 1B). To determine the effect of TNF-α on AdBMP9-induced osteogenic differentiation, rDFCs were transfected with AdBMP9 prior to treatment with 10 ng/ml TNF-α, which was selected as the stimulatory concentration for use in the experiments (8). Cells were collected 7- and 14-days post-transfection and analyzed via ALP staining and Alizarin Red S staining, respectively. The results revealed that TNF-α treatment not only the reduced osteogenic medium-induced osteogenesis, but it also downregulated BMP9-induced osteogenic differentiation (Fig. 2A). The expression levels of bone-related genes, ALP, collagen type 1 α1 (COLI), and the late osteogenesis marker osteocalcin (OCN), were further analyzed, in addition to the osteogenic differentiation-related transcription factor, RUNX2. RT-qPCR analysis discovered that the expression levels of these genes were significantly upregulated in the rDFCs + AdBMP9 group compared with the rDFCs group; however, these expression levels were significantly downregulated in the rDFCs + AdBMP9 + TNF-α group (Fig. 2B-E). These results indicated that AdBMP9 may promote the osteogenic differentiation of rDFCs, which may be suppressed by TNF-α.

TNF-α regulates the Wnt signaling pathway during the BMP9-induced osteogenic differentiation of rDFCs. Wnt signaling has been discovered to be crucial for BMP9-induced osteogenic differentiation (8,24). AdGFP was used in preliminary western blotting experiments as the vector control as a comparison to the effects of AdBMP9 (Fig. 3A and C). After TNF-α stimulation, the expression levels of β-catenin and p-GSK3β in the TNF-α and BMP9 + TNF-α groups were upregulated compared with the control and BMP9 groups, respectively (Fig. 3B). The expression levels of CaMKII and NLK were also downregulated following TNF-α stimulation in the TNF-α and BMP9 + TNF-α groups compared with the control and BMP9 groups, respectively (Fig. 3D). CaMKII and NLK are parts of the non-canonical Wnt signaling pathway (21,23) Thus, these results suggested that TNF-α may inhibit the non-canonical Wnt signaling pathway. Cyclin D1 and c-Myc are downstream genes in the canonical Wnt signaling pathway (27,28). The RT-qPCR results demonstrated that the expression levels of cyclin D1 and c-Myc were significantly upregulated in the TNF-α stimulation group compared with the non-TNF-α stimulation group (Fig. 3E), indicating that the canonical Wnt signaling pathway of rDFCs may be activated by TNF-α.

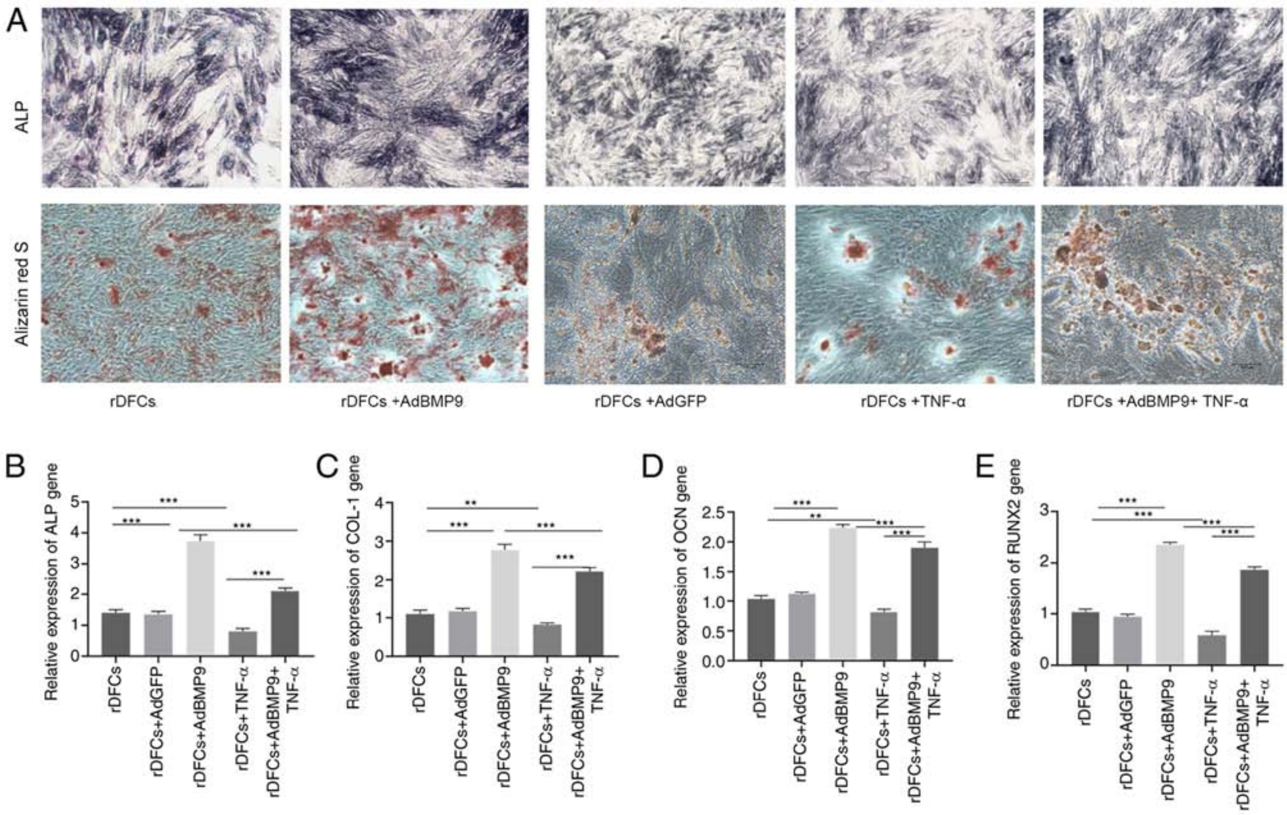

Figure 2. TNF-α suppresses AdBMP9-induced osteogenic differentiation of rDFCs. (A) ALP (magnification, x40) and Alizarin Red S staining (magnification, x100) of cells were performed on days 7 and 14, respectively. BMP9 enhanced ALP activity and matrix mineralization and TNF-α reduced the effect of osteogenesis. Reverse transcription-quantitative PCR was used to analyze the expression levels of the osteogenic markers (B) ALP, (C) COL1, (D) OCN and (E) RUNX2 on day 7 post-transfection. The expression levels of these markers were upregulated by BMP9 but downregulated by TNF-α. **P<0.01 and ***P<0.001. rDFCs, rat follicle stem cells; Ad, adenovirus; BMP9, bone morphogenetic protein 9; TNF-α, tumor necrosis factor α; ALP, alkaline phosphatase; COL1, collagen type 1 α1; OCN, osteocalcin; RUNX2, runt-related transcription factor 2.

Effect of DKK1 treatment on AdBMP9-induced osteogenic differentiation of rDFCs. DKK1 is an inhibitor of the Wnt/β-catenin signaling pathway (8,23). The present study selected 0.1 and 0.4 µg/ml DKK1 as the stimulatory concentrations to treat the rDFCs with. Following the treatment with the higher concentration of DKK1 or TNF-α, AdBMP9-induced osteogenic differentiation was reduced compared with the AdBMP9 group (Fig. 4A); however, the addition of low concentrations of DKK1 promoted osteogenesis. The RT-qPCR analysis revealed a similar result; the expression levels of RUNX2, OCN and COLI were significantly upregulated following the treatment with lose dose DKK1 in AdBMP9-transduced rDFCs compared with the rDFCs + AdBMP9 group (Fig. 4E). However, TNF-α treatment and high dose DKK1 treatment significantly downregulated the expression levels of these genes compared with the rDFCs + AdBMP9 group (Fig. 4E). In addition, the expression levels of β-catenin, p-GSK3β and cyclin D1 were downregulated following the treatment with 0.1 and 0.4 µg/ml DKK1 compared with the rDFC + AdBMP9 group (Fig. 4B and D). Thus, these findings suggested that DKK1 may suppress the canonical Wnt/β-catenin signaling pathway. Furthermore, following the treatment with 0.4 µg/ml DKK1, the expression levels of CaMKII and NLK were upregulated compared with the rDFCs + AdBMP9 group, which suggested the partial enhancement of the non-canonical Wnt signaling pathway (Fig. 4C and D). Conversely, low dose DKK1 (0.1 µg/ml) downregulated the expression levels of CaMKII and NLK compared with the rDFCs + AdBMp9 group, which suggested the inhibition of the non-canonical Wnt signaling pathway. The abovementioned results suggested that high concentrations of DKK1 may activate non-canonical Wnt signaling and suppress canonical Wnt signaling, whereas low concentrations of DKK1 may suppress both canonical and non-canonical Wnt signaling in AdBMP9-transduced rDFCs.

Furthermore, the effect of restoring a bone defect was evaluated in a Sprague Dawley rat model. H&E staining demonstrated that TNF-α and high dose DKK1 treatment promoted a small amount of bone formation from AdBMP9-transduced rDFCs. Conversely, a mass of newly formed bone and numerous blood vessels were observed in Sprague Dawley rats that were treated with AdBMP9-transduced rDFCs and low dose DKK1 (Fig. 4F).

These results suggested that high concentrations of DKK1 and TNF-α may act via the non-canonical and canonical Wnt signaling pathways to downregulate bone formation, whereas low concentrations of DKK-1 may promote BMP-9-induced osteogenic differentiation via inhibition of the non-canonical and canonical Wnt signaling pathways.

Discussion

rDFCs are the precursor cells of periodontal ligament cells, cement cells and osteoblasts, which form periodontal tissue at

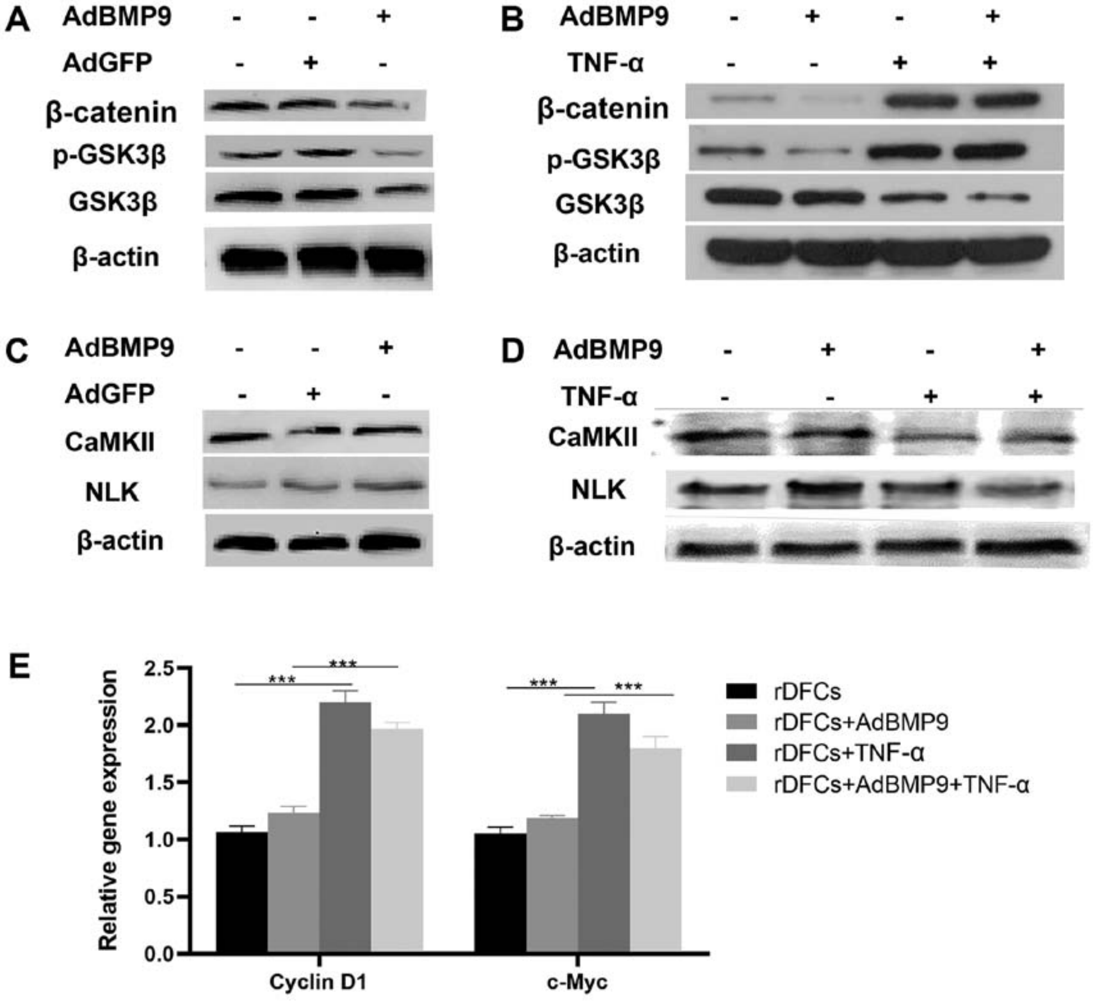

Figure 3. TNF-α influences the Wnt signaling pathways. (A) Western blotting was used to analyze the expression levels of β-catenin, p-GSK3β and GSK3β in the blank, AdGFP and AdBMP9 groups. (B) Western blotting revealed that TNF-α (10 ng/ml) treatment upregulated the expression levels of β-catenin and p-GSK3β. (C) Western blotting was used to analyze the expression levels of CaMKII and NLK in the blank, AdGFP and AdBMP9 groups. (D) Western blotting revealed that the expression levels of CaMKII and NLK were downregulated by TNF-α (10 ng/ml) treatment. (E) Cyclin D1 and c-Myc expression levels were detected using reverse transcription quantitative PCR. TNF-α upregulated the expression levels of cyclin D1 and c-Myc. ***$P<0.001$. rDFCs, rat follicle stem cells; Ad, adenovirus; BMP9, bone morphogenetic protein 9; TNF-α, tumor necrosis factor α; GSK3β, glycogen synthase kinase 3β; p-, phosphorylated; CaMKII, calcium/calmodulin-dependent protein kinase type II α chain; NLK, serine/threonine protein kinase NLK.

the late dental development stage via cellular migration and differentiation (5-7). AdBMP9 acts on cells as an exogenous factor to safely and effectively transfect the target gene into the target cell (8-13). The present study transfected cells with an adenovirus to construct an AdBMP9-transduced rDFC model.

TNF-α levels were reported to be markedly increased in the gingival crevicular fluid of patients with periodontitis and the expression levels of TNF-α have been closely associated with the severity of periodontitis (29,30). Previous studies have identified that TNF-α suppressed cell osteogenic differentiation (8,21,31,32). It was also discovered that high concentrations of TNF-α suppressed the expression of osteogenesis-related factors, such as ALP, OCN and RUNX2 in mesenchymal stem cells (33,34).

The effect of TNF-α on AdBMP-induced osteogenic differentiation has been discovered to be associated with periodontal tissue engineering (17,32,34). Thus, the purpose of the present study was to investigate the effect of TNF-α on the AdBMP9-induced osteogenic differentiation of rDFCs. The results of the present study indicated that AdBMP9 promoted osteogenesis in rDFCs. However, the osteogenic effect was markedly reduced following TNF-α stimulation, indicating that TNF-α may suppress the AdBMP9-induced early and advanced stages of osteogenesis in rDFCs. Similarly, some studied reported that TNF-α prevented the osteogenic differentiation of cells under BMP2/7 induction. BMP2 and BMP9 belong to the same BMP family (12,35,36). BMP9 has been proven to be one of the strongest osteogenic factors, demonstrating an ability to repair and regenerate bone defects (12,13). Consequently, BMP9 was also suggested to potentially promote bone regeneration in TNF-α-induced bone defects.

The present study preliminarily discovered that TNF-α suppressed the osteogenic differentiation of rDFCs. However, the regulatory molecular mechanisms of this pathway remain unclear. A large number of previous studies have indicated that the Wnt signaling pathways served an important role in the osteogenic differentiation of stem cells (18,21,23,25,37,38). Therefore, the present study analyzed the expression levels of important proteins involved in the Wnt signaling pathways.

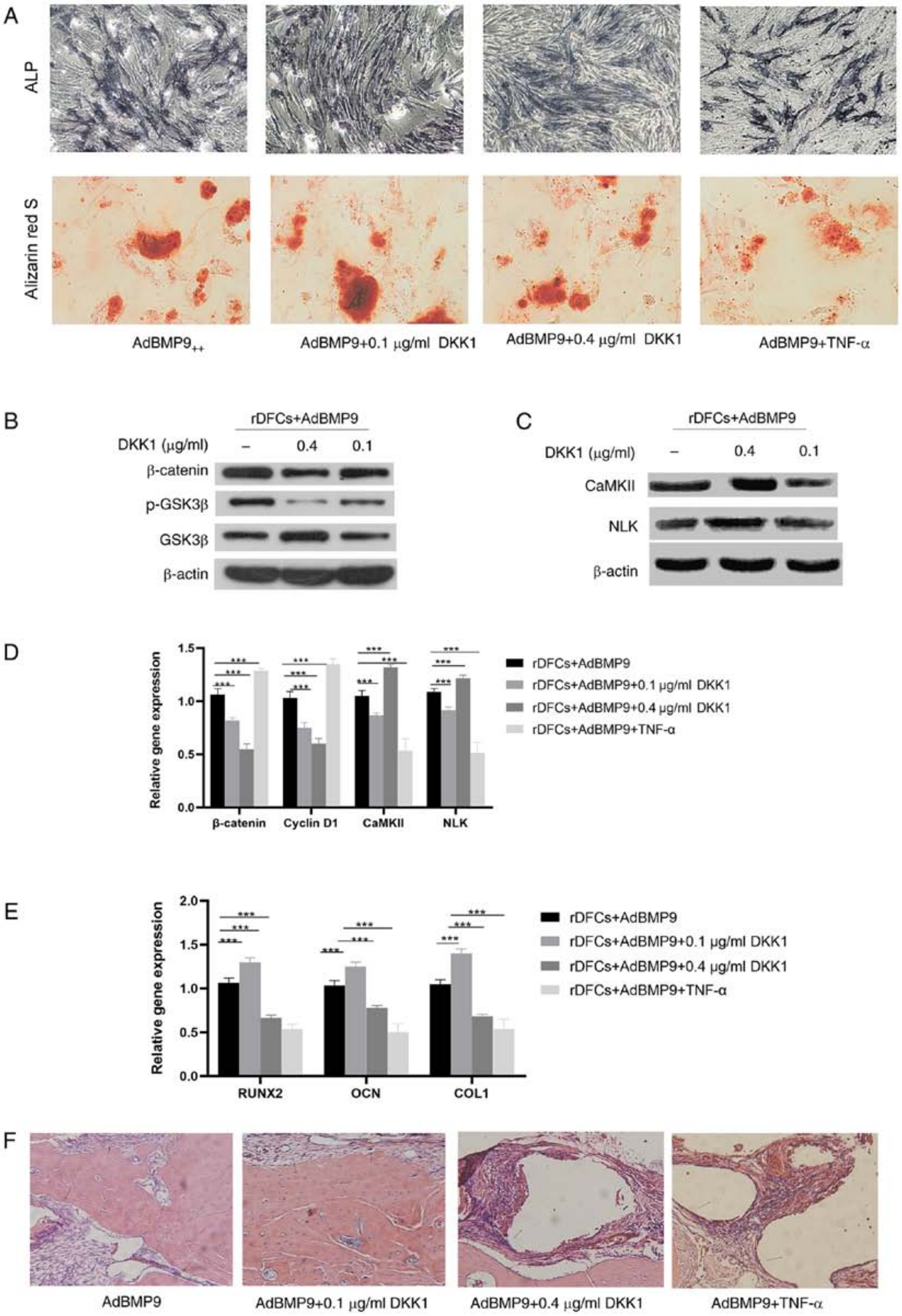

Figure 4. Effect of DKK1 on AdBMP9-induced osteogenic differentiation of rDFCs. (A) Alkaline phosphatase staining (magnification, x40) was performed on day 7 post-transfection and Alizarin Red S staining (magnification, x100) was performed on day 14 post-transfection. ALP staining intensity was enhanced in BMP9 group and AdBMP9+DKK1 (0.1 μg/ml) group when compared with the BMP9+TNF-α group and AdBMP9+DKK1 (0.4 μg/ml) group. More calcified nodules were found in BMP9 group and AdBMP9+ DKK1 (0.1 μg/ml) group compared with the BMP9+TNF-α group and AdBMP9+ DKK1 (0.4 μg/ml) group. (B) Effect of DKK1 on canonical Wnt signaling in AdBMP9-transduced rDFCs. Western blotting revealed that DKK1 downregulated the expression levels of β-catenin and p-GSK3β. (C) Effect of DKK1 on non-canonical Wnt signaling in AdBMP9-transduced rDFCs. The results revealed that 0.4 ng/ml DKK1 upregulated the expression levels of CaMKII and NLK, whereas 0.1 ng/ml DKK1 downregulated CaMKII expression levels. No obvious differences were observed in NLK expression levels. (D) RT-qPCR was used to analyze the mRNA expression levels of β-catenin, cyclin D1, CaMKII and NLK. (E) RT-qPCR was used to determine mRNA expression levels of the bone markers RUNX2, OCN and COLI. The expression levels of these markers were upregulated following 0.1 ng/ml DKK1 treatment but downregulated with 0.4 ng/ml DKK1 and TNF-α (10 ng/ml). (F) Hematoxylin and eosin staining of sections of new bone formation from Sprague Dawley rats (magnification, x40). ***P<0.001. rDFCs, rat follicle stem cells; Ad, adenovirus; BMP9, bone morphogenetic protein 9; TNF-α, tumor necrosis factor α; DKK1, Dickkopf 1; GSK3β, glycogen synthase kinase 3β; p-, phosphorylated; CaMKII, calcium/calmodulin-dependent protein kinase type II α chain; NLK, serine/threonine protein kinase NLK; COL1, collagen type 1 α1; OCN, osteocalcin; RUNX2, runt-related transcription factor 2; RT-qPCR, reverse transcription-quantitative PCR.

The experimental results revealed that following TNF-α interference, the canonical Wnt/β-catenin signaling pathway was activated and the Wnt/Ca^{2+} non-canonical signaling pathway was inhibited. Previous studies have also suggested that the canonical Wnt/β-catenin pathway may prevent the differentiation process of certain stem cells, such as the odontoblast-like differentiation of dental pulp stem cell and the osteogenic differentiation of adipose derived stromal cells (37,39), which is consistent with the results of the present study.

The primary mechanism of action of the canonical Wnt/β-catenin signaling pathway is as follows: The Wnt protein binds to the cell surface membrane Frizzled receptor, which activates the Dishevelled receptor family, suppresses the downstream Axin/GSK3β/adenomatous polyposis coli complex and thus, represses the degradation of β-catenin (21,23). Subsequently, β-catenin enters the cell nucleus, interacts with the TCL/LEF transcription factor and promotes the expression of specific genes (40,41). GSK3β is a protein kinase which phosphorylates β-catenin, thus inducing its degradation through the ubiquitin proteasome pathway (42). The results of the present study revealed that compared with the AdBMP-transfected cells without TNF-α stimulation, the TNF-α treatment upregulated the expression levels of β-catenin and p-GSK3β. TNF-α upregulated expression levels of cyclin D1 and c-Myc, which are downstream target genes of the canonical Wnt signaling pathway.

Wnt signaling can be categorized into the canonical and non-canonical Wnt/Ca^{2+} signaling pathway (23). However, to the best of our knowledge, whether the non-canonical Wnt pathway is involved in the osteogenic differentiation of stem cells following TNF-α stimulation remained unclear. CaMKII and NLK are key proteins of wnt non-canonical signaling pathway (23,43) NLK translocates into the nucleus and suppress the regulatory effect of the β-catenin/TCF/LEF polymer on gene transcription, thus affecting cellular function (43,44). The present study discovered that TNF-α treatment downregulated the expression levels of CaMKII and NLK in AdBMP9-transduced rDFCs. These results indicated that the Wnt/β-catenin signaling pathway may be activated by TNF-α, whereas the non-canonical Wnt/Ca^{2+} signaling pathway may be suppressed.

The aforementioned results of the present study preliminarily verified that TNF-α may suppress the osteogenic differentiation of rDFCs, which was closely associated with the activation of the canonical Wnt/β-catenin signaling pathway and the suppression of the Wnt/Ca^{2+} non-canonical signaling pathway. To further verify the roles of the Wnt signaling pathways in AdBMP9-indued osteogenic differentiation of rDFCs with TNF-α, the Wnt canonical signaling inhibitor DKK1 was used. The results of the western blotting experiments revealed that high concentrations of DKK1 upregulated the expression levels of CaMKII and NLK, while low concentrations of DKK1 downregulated the expression levels of these proteins. β-catenin expression levels were downregulated by DKK1 at both high and low concentrations; however, the inhibitory effect was higher following high dose DKK1 treatment compared with low dose DKK1. These results suggested that Wnt/β-catenin signaling may be suppressed by both high and low concentrations of DKK1, whereas the Wnt/Ca^{2+} non-canonical pathway may be activated by high concentrations of DKK1 and suppressed by low concentrations of DKK1. The RT-qPCR results revealed that low concentrations of DKK1 significantly upregulated the expression levels of RUNX2, OCN and COLI. Conversely, TNF-α treatment and high concentrations of DKK1 downregulated the expression levels of these genes. These results suggested that low concentrations of DKK1 may promote AdBMP9-induced osteogenic differentiation.

These results highlighted the close association between the canonical and Wnt/Ca^{2+} non-canonical signaling pathways, suggesting that the two pathways may exert their functions simultaneously to regulate stem cell differentiation. Under an inflammatory microenvironment, the dynamic balance between the canonical and non-canonical signaling pathways is reportedly broken, which thereby affects the stem cell function (21,25,31,34).

TNF-α has been reported as an important inflammatory factor responsible for periodontal bone defects resulting from periodontitis (17,32). In the present study, TNF-α suppressed the AdBMP9-induced osteogenic differentiation of rDFCs by activating the canonical Wnt signaling pathway and repressing the non-canonical signaling pathway. The results indicated that DKK1 also influenced canonical and non-canonical signaling pathways, thereby affecting the osteogenic differentiation of cells. These findings suggested that regulating the balance between the Wnt canonical and non-canonical signaling pathways may be promising for restoring the osteogenic differentiation of rDFCs.

Collectively, the results of the present study indicated that TNF-α may interact with the Wnt signaling pathway to suppress osteogenesis. Thus, the enhanced promoting effect of BMP9 following treatment with low concentrations of DKK1 may be useful for treating periodontitis bone absorption.

In conclusion, the results of the present study provided novel evidence for studying stem cell function in relation to TNF-α and DKK1, thus providing an opportunity for stem cells to be applied in clinical studies in the future. However, the present study did not investigate the effects of the feedback loop of TNF-α and DKK1, which may provide an additional mechanism of crosstalk between BMPs and Wnt signaling. Therefore, further scientific research is required to determine the interaction between signaling pathways *in vivo* and *in vitro*, which will enable the optimization of therapies for bone tissue engineering.

Acknowledgements

The authors would like to thank Dr Tong-Chuan He (Molecular Oncology Laboratory, Department of Surgery, The University of Chicago Medical Center, Chicago, IL, USA) for providing AdBMP9 and AdGFP. The authors would also like to thank Dr Guo Ye (Chongqing Key Laboratory of Oral Diseases and Biomedical Sciences, Chongqing Medical University, Chongqing, China) for providing hydroxyapatite.

Funding

The study was funded by The Natural Science Foundation of Chongqing, China (grant no. cstc2016jcyjA0243), The Chongqing Municipal Health and Family Planning commission

(grant no. 2016MSXM049) and The Program for Innovation Team Building at the Institutions of Higher Education in Chongqing in 2016 (grant no. CXTDG201602006).

Availability of data and materials

The datasets used and/or analyzed during the current study are available from the corresponding author on reasonable request.

Authors' contributions

CL conceived the project; XL, GR, CC and CL designed the experiments and wrote the manuscript; XY, XL, GR, LN and XJ performed the experiments; and CL supervised the experiment and revised the manuscript. All authors read and approved the final manuscript.

Ethics approval and consent to participate

All experimental procedures involving animals were in accordance with the Guide for the Care and Use of Laboratory Animals (45) and approved by the Ethics Committee of Chongqing Medical University (approval no. CQLA2019-0066; Chongqing, China). All animals were purchased from the Experimental Animal Center of Chongqing Medical University [license no. SCXK (Yu) 20012-0001].

Patient consent for publication

Not applicable.

Competing interests

The authors declare that they have no competing interests.

References

1. Pihlstrom BL, Michalowicz BS and Johnson NW: Periodontal diseases. Lancet 366: 1809-1820, 2005.
2. Lim JC and Mitchell CH: Inflammation, pain, and pressure-purinergic signaling in oral tissues. J Dent Res 91: 1103-1109, 2012.
3. Kodama T, Minabe M, Sugiyama T, Mitarai E, Fushimi H, Kitsugi D, Tsutsumi K and Katsuki M: Guided tissue regeneration using a collagen barrier and bone swaging technique in noncontained infrabony defects. Int J Periodontics Restorative Dent 33: 805-812, 2013.
4. Wise GE, Lin F and Fan W: Culture and characterization of dental follicle cells from rat molars. Cell Tissue Res 267: 483-492, 1992.
5. Guo W, Chen L, Gong K, Ding B, Duan Y and Jin Y: Heterogeneous dental follicle cells and the regeneration of complex periodontal tissues. Tissue Eng Part A 18: 459-470, 2012.
6. Li C, Yang X, He Y, Ye G, Li X, Zhang X, Zhou L and Deng F: Bone morphogenetic protein-9 induces osteogenic differentiation of rat dental follicle stem cells in P38 and ERK1/2 MAPK dependent manner. Int J Med Sci 9: 862-871, 2012.
7. Yao S, Pan F, Prpic V and Wise GE: Differentiation of stem cells in the dental follicle. J Dent Res 87: 767-771, 2008.
8. Li X, Chen D, Jing X and Li C: Dkk1 and TNF-alpha influence osteogenic differentiation of adBMP9-infected-rDFCs. Oral Dis 26: 360-369, 2020.
9. Carreira AC, Alves GG, Zambuzzi WF, Sogayar MC and Granjeiro JM: Bone Morphogenetic Proteins: Structure, biological function and therapeutic applications. Arch Biochem Biophys 561: 64-73, 2014.
10. Chen D, Zhao M and Mundy GR: Bone morphogenetic proteins. Growth Factors 22: 233-241, 2004.
11. Cheng H, Jiang W, Phillips FM, Haydon RC, Peng Y, Zhou L, Luu HH, An N, Breyer B, Vanichakarn P, *et al*: Osteogenic activity of the fourteen types of human bone morphogenetic proteins (BMPs). J Bone Joint Surg Am 85-A: 1544-1552, 2003.
12. Kang Q, Sun MH, Cheng H, Peng Y, Montag AG, Deyrup AT, Jiang W, Luu HH, Luo J, Szatkowski JP, *et al*: Characterization of the distinct orthotopic bone-forming activity of 14 BMPs using recombinant adenovirus-mediated gene delivery. Gene Ther 11: 1312-1320, 2004.
13. Peng Y, Kang Q, Cheng H, Li X, Sun MH, Jiang W, Luu HH, Park JY, Haydon RC and He TC: Transcriptional characterization of bone morphogenetic proteins (BMPs)-mediated osteogenic signaling. J Cell Biochem 90: 1149-1165, 2003.
14. Nie L, Yang X, Duan L, Huang E, Zhou PF, Luo W, Zhang Y, Zeng X, Qiu Y, Cai T, *et al*: The healing of alveolar bone defects with novel bio-implants composed of Ad-BMP9-transfected rDFCs and CHA scaffolds. Sci Rep 7: 6373, 2017.
15. Kimelman Bleich N, Kallai I, Lieberman JR, Schwarz EM, Pelled G and Gazit D: Gene therapy approaches to regenerating bone. Adv Drug Deliver Rev 64: 1320-1330, 2012.
16. Graves DT and Cochran D: The contribution of interleukin-1 and tumor necrosis factor to periodontal tissue destruction. J Periodontol 74: 391-401, 2003.
17. Mukai T, Otsuka F, Otani H, Yamashita M, Takasugi K, Inagaki K, Yamamura M and Makino H: TNF-alpha inhibits BMP-induced osteoblast differentiation through activating SAPK/JNK signaling. Biochem Biophys Res Commun 356: 1004-1010, 2007.
18. Maeda K, Takahashi N and Kobayashi Y: Roles of Wnt signals in bone resorption during physiological and pathological states. J Mol Med (Berl) 91: 15-23, 2013.
19. Qiu W, Andersen TE, Bollerslev J, Mandrup S, Abdallah BM and Kassem M: Patients with high bone mass phenotype exhibit enhanced osteoblast differentiation and inhibition of adipogenesis of human mesenchymal stem cells. J Bone Miner Res 22: 1720-1731, 2007.
20. Jansen JH, Eijken M, Jahr H, Chiba H, Verhaar JA, Van LJ and Weinans H: Stretch-induced inhibition of Wnt/beta-catenin signaling in mineralizing osteoblasts. J Orthop Re 28: 390-396, 2010.
21. Liu N, Shi HG, Zhang W and Gu B: The crosstalk between canonical and noncanonical Wnt signaling pathway in osteoblast differentiation of periodontal ligament stem cells in inflammatory microenvironments. Zhonghua Kou Qiang Yi Xue Za Zhi 51: 673-679. 2016 (In Chinese).
22. Xiang L, Chen M, He L, Cai B, Du Y, Zhang X, Zhou C, Wang C, Mao JJ and Ling J: Wnt5a regulates dental follicle stem/progenitor cells of the periodontium. Stem Cell Res Ther 5: 135, 2014.
23. Lerner UH and Ohlsson C: The WNT system: Background and its role in bone. J Intern Med 277: 630-649, 2015.
24. Salazar VS, Ohte S, Capelo LP, Gamer L and Rosen V: Specification of osteoblast cell fate by canonical wnt signaling requires Bmp2. Development 143: 4352-4367, 2016.
25. Weivoda MM, Ruan M, Hachfeld CM, Pederson L, Howe A, Davey RA, Zajac JD, Kobayashi Y, Williams BO, Westendorf JJ, *et al*: Wnt signaling inhibits osteoclast differentiation by activating canonical and noncanonical cAMP/PKA pathways. J Bone Miner Res 31: 65-75, 2016.
26. Livak KJ and Schmittgen TD: Analysis of relative gene expression data using real-time quantitative PCR and the 2(-Delta Delta C(T)) method. Methods 25: 402-408, 2001.
27. Shtutman M, Zhurinsky J, Simcha I, Albanese C, D'Amico M, Pestell R and Ben-Ze'ev A: The cyclin D1 gene is a target of the beta-catenin/LEF-1 pathway. Proc Natl Acad Sci USA 96: 5522-5527, 1999.
28. Pelengaris S and Khan M: The many faces of c-MYC. Arch Biochem Biophys 416: 129-136, 2003.
29. Tan J, Zhou L, Xue P, An Y, Luo L, Zhang R, Wu G, Wang Y, Zhu H and Wang Q: Tumor necrosis factor-α attenuates the osteogenic differentiation capacity of periodontal ligament stem cells by Activating PERK Signaling. J Periodontal 87: e159-e171, 2016.
30. Ding C, Ji X, Chen X, Xu Y and Zhong L: TNF-α gene promoter polymorphisms contribute to periodontitis susceptibility: Evidence from 46 studies. J Clin Periodontol 41: 748-759, 2014.
31. Liu X, Tan GR, Yu M, Cai X, Zhou Y, Ding H, Xie H, Qu F, Zhang R, Lam CU, *et al*: The effect of tumour necrosis factor-α on periodontal ligament stem cell differentiation and the related signaling pathways. Curr Stem Cell Res Ther 11: 593-602, 2016.

32. Wang F, Jiang Y, Huang X, Liu Q, Zhang Y, Luo W, Zhang F, Zhou P, Lin J and Zhang H: Pro-inflammatory cytokine TNF-α attenuates BMP9-induced osteo/odontoblastic differentiation of the stem cells of dental apical papilla (SCAPs). Cell Physiol Biochem 41: 1725-1735, 2017.
33. Lacey DC, Simmons PJ, Graves SE and Hamilton JA: Proinflammatory cytokines inhibit osteogenic differentiation from stem cells: Implications for bone repair during inflammation. Osteoarthri Cartilage 17: 735-742, 2009.
34. Qin Z, Fang Z, Zhao L, Chen J, Li Y and Liu G: High dose of TNF-α suppressed osteogenic differentiation of human dental pulp stem cells by activating the Wnt/β-catenin signaling. J Mol Histol 46: 409-420, 2015.
35. Huang RL, Yuan Y, Tu J, Zou GM and Li Q: Opposing TNF-α/IL-1β-and BMP-2-activated MAPK signaling pathways converge on Runx2 to regulate BMP-2-induced osteoblastic differentiation. Cell Death Dis 5: e1187, 2014.
36. Jo JY, Jeong SI, Shin YM, Kang SS, Kim SE, Jeong CM and Huh JB: Sequential delivery of BMP-2 and BMP-7 for bone regeneration using a heparinized collagen membrane. Int J Oral Max Surg 44: 921-928, 2015.
37. Cho HH, Kim YJ, Kim SJ, Kim JH, Bae YC, Ba B and Jung JS: Endogenous Wnt signaling promotes proliferation and suppresses osteogenic differentiation in human adipose derived stromal cells. Tissue Eng 12: 111-121, 2006.
38. Wang Y, Li YP, Paulson C, Shao JZ, Zhang X, Wu M and Chen W: Wnt and the Wnt signaling pathway in bone development and disease. Front Biosci (Landmark Ed) 19: 379-407, 2014.
39. Scheller EL, Chang J and Wang CY: Wnt/beta-catenin inhibits dental pulp stem cell differentiation. J Den Res 87: 126-30, 2008.
40. Kim W, Kim M and Jho EH: Wnt/beta-catenin signalling: from plasma membrane to nucleus. Biochem J 450: 9-21, 2013.
41. Gao C, Xiao G and Hu J: Regulation of Wnt/β-catenin signaling by posttranslational modifications. Cell Biosci 4: 13, 2014.
42. Tejeda-Muñoz N and Robles-Flores M: Glycogen synthase kinase 3 in Wnt signaling pathway and cancer. IUBMB Life 67: 914-922, 2015.
43. De A: Wnt/Ca2+ signaling pathway: A brief overview. Acta Biochim Biophys Sin (Shanghai) 43: 745-756, 2011.
44. Rharass T, Lemcke H, Lantow M, Kuznetsov SA, Weiss DG and Panáková D: Ca2+-mediated mitochondrial reactive oxygen species metabolism augments Wnt/β-catenin pathway activation to facilitate cell differentiation. J Biol Chem 289: 27937-29951, 2014.
45. Committee for the Update of the Guide for the Care and Use of Laboratory Animals, Institute for Laboratory Animal Research, Division on Earth and Life Studies, National Research Council: Guide for the Care and Use of Laboratory Animals. The National Academies Press, Washington, DC, 1998.

This work is licensed under a Creative Commons Attribution-NonCommercial-NoDerivatives 4.0 International (CC BY-NC-ND 4.0) License.
