# Supplementary material for: Molecular Regulators for the Wnt/β-Catenin Signalling Pathway for Bone Remodelling in Periodontitis: A Scoping Review
Source: Int J Mol Sci. 2026 Oct 14;27(20):9117. doi: 10.3390/ijms27209117 (PMC13705578; doi:10.3390/ijms27209117)
Supplement: Supplementary file 1 [file ijms-27-09117-s001.zip › Supplementary Data/Papers/in vivo/Cathelicidin LL-37 improves bone metabolic balance in rats with ovariectomy-induced osteoporosis via the Wntbeta-catenin pathway..pdf]

Cathelicidin LL-37 Improves Bone Metabolic Balance in Rats With Ovariectomy-Induced Osteoporosis via the Wnt/β-Catenin Pathway

Jianwei LIANG[1], Jianjun CHEN[1], Zhan YE[1], Dandan BAO[2]

[1]Department of Orthopedics, The First People's Hospital of Taizhou, Taizhou, China, [2]Department of Pharmacy, The First People's Hospital of Taizhou, Taizhou, China

Received October 13, 2021
Accepted March 15, 2022
Epub Ahead of Print May 26, 2022

Summary

Osteoporosis is a bone disease characterized by low bone mineral density (BMD) and impaired bone microarchitecture due to the abnormal activity of osteoclasts. Cathelicidins are antimicrobial peptides present in the lysosomes of macrophages and polymorphonuclear leukocytes. LL-37, a cathelicidin, induces various biological effects, including modulation of the immune system, angiogenesis, wound healing, cancer growth, as well as inflammation, and bone loss. A previous study reported direct involvement of LL-37 suppressing osteoclastogenesis in humans. Here, we examined the role of LL-37 in the treatment of osteoporosis using an ovariectomy (OVX) rat model. Our results showed that LL-37 significantly reduced bone loss and pathological injury in OVX rats with osteoporosis. Furthermore, we found that LL-37 significantly increased the activity of the Wnt/β-catenin pathway in OVX rats with osteoporosis, including the increased expression of β-catenin, Osterix (Osx), and Runt-related transcription factor 2 (Runx2), whereas XAV-939, an inhibitor of the Wnt/β-catenin pathway, significantly blocked the effects of LL-37 on bone loss and abnormal bone metabolism. Altogether, our findings suggested that LL-37 exerted a protective role in regulating bone loss and abnormal bone metabolism in rats with osteoporosis by activating the Wnt/β-catenin pathway.

Key words

LL-37 • Osteoporosis • Bone metabolism • Wnt/β-catenin pathway

Corresponding author

D. Bao, Department of Pharmacy, The First People's Hospital of Taizhou, 218 Hengjie Road, Huangyan District, Taizhou, Zhejiang Province, China. E-mail: baodandan-007@163.com

Introduction

Osteoporosis, a systemic metabolic bone disease (BMD) characterized by reduced bone mineral density, low bone mass, microarchitectural disturbance of bone tissue, and increased bone fragility predisposing to fragility fractures, becomes a major global health problem [1]. Osteoporosis is divided into primary osteoporosis and secondary osteoporosis based on their etiology. Primary osteoporosis includes postmenopausal osteoporosis (PMOP) (type I), senile osteoporosis (type II). Among them, type I osteoporosis is further divided into PMOP (type IA) and male osteoporosis (type IB). Osteoporosis tends to occur in people of advanced age and in postmenopausal women, and patients mostly suffer from circumferential body pain and fragility fractures, which are the main clinical features and seriously affect the quality of life [2]. PMOP is characterized by the decreased levels of sex hormones in menopause of women causing a weakened inhibitory effect on osteoclasts, resulting in bone mass loss, bone microarchitectural changes, increased bone fragility, and stronger bone resorption function than bone formation, causing an imbalance in bone remodeling, thereby leading to decreased bone strength, has been considered as the most common type of osteoporosis [3-5]. At present, clinical anti osteoporosis drugs are mainly divided into two main classes, one is the bone promoting agents, such as teriparatide, ramucirumab; the other is bone resorption inhibitor, such as bisphosphonates, estrogen, selective estrogen receptor modulators, denosumab, etc. [6-10]. Unfortunately, the long-term use

PHYSIOLOGICAL RESEARCH • ISSN 1802-9973 (online) - an open access article under the CC BY-NC-ND 4.0 license
© 2022 Institute of Physiology of the Czech Academy of Sciences, Prague, Czech Republic
Fax +420 241 062 164, e-mail: physres@fgu.cas.cz, www.biomed.cas.cz/physiolres

of these drugs causes many potential side effects, such as dyspepsia, constipation and so on [7,11]. Therefore, the development of safe and effective treatment strategies for osteoporosis without excessive side effects is urgently required.

Wnt/β-catenin signaling pathway exerts an important role in normal bone growth and metabolism, such as promoting the differentiation of bone marrow mesenchymal stem cells (BMSCs) into osteoblasts, stimulating the proliferation of osteoblasts, inhibiting the activity of osteoclasts, and maintaining the balance between bone formation and resorption [12]. It has been shown that activating Wnt/β-catenin signaling pathway can promote osteogenesis, increase bone mineral density (BMD) and bone quality, improve bone structure and bone metabolism, thereby to play the therapeutic role of osteoporosis [13,14]. Therefore, the Wnt/β-catenin signaling pathway may be a potential target for the treatment of osteoporosis, and expected to be used in clinical practice in the future and achieve better curative effects.

LL-37 is the only human member of the cathelicidin family. It is an amphipathic, positively charged, 37-residue peptide generated from the precursor hCAP18 protein. LL-37 is stored in the secondary granules of neutrophils, from where it is released upon activation [15,16]. It exerts activity against most gram-negative and gram- positive bacteria with the primary role to exterminate the pathogens [17]. Numerous studies have shown that LL-37 participates in several host immune reactions, such as inflammatory responses and tissue repair, in addition to its antibacterial properties [18]. LL-37 has been shown to enhance the immune response by inducing the production of selective cytokines and chemokines [17]. Moreover, it is implicated in several key biological processes involving non-immune cells such as angiogenesis, re-epithelialization, wound closure, and the maintenance of intestinal epithelial barrier integrity [19-22]. In addition, LL-37 can directly suppress osteoclastogenesis in humans, thereby protecting against bone resorption induced by a bacterial infection in periodontal diseases [23].

In the present study, we hypothesized that LL-37 regulates the bone metabolic balance to attenuate ovariectomy (OVX)-induced bone loss and pathological injury in ovariectomized rats. We studied bone formation and resorption, as well as the serum bone metabolism parameters, and investigated the activity of the Wnt/β-catenin pathway, which could be regulated by LL-37 as reported by a previous study [24,25].

Materials and Methods

Animals

Seventy-five 3-month old female Sprague-Dawley (SD) rats weighing 230 to 240 g were obtained from Chengdu Dossy Experimental Animals Co. Ltd. (certificate number: SCXK [Chuang] 2019-031). All animals were maintained in an animal house under controlled temperature (23±2 °C) and relative humidity (50-55 %) in a 12/12 h (light/dark) cycle. They were provided free access to tap water and commercially available standard rat chow. Animals were allowed to acclimatize for one week before the experiment. All animal experiments performed in this study were approved by the Animal Ethical Committee of Wuhan Cloud-Clone Technology Co., Ltd.

OVX-induced osteoporosis and drug administration

After one week of acclimatization, the rats were anesthetized and subsequently subjected to bilateral OVX to establish osteoporotic animal models. As a control, the rats in sham-operated (sham) group were only removed the same volume of fat tissues surrounding the ovaries. One week after recovering from the surgery, OVX rats were randomly divided into four groups of 15 rats each, according to their body weight and named as OVX group, LL-37 treatment (OVX+LL-37) group, XAV-939 treatment (OVX+XAV-939) group, and LL-37 and XAV-939 co-treatment (OVX+LL-37+XAV-939) group. During the treatment, the rats in the OVX+LL-37 group were intraperitoneally administrated with LL-37 (1.5 mg/kg), those in the OVX+XAV-939 group were intraperitoneally administrated with XAV-939 (1.0 mg/ml), those in the OVX+LL-37+XAV-939 group were intraperitoneally co-administrated with LL-37 and XAV-939, and the rats in the OVX group and sham group were intraperitoneally administrated with an equal volume of saline. The treatments were performed once every 2 days. After 12 weeks of administration, the rats were anesthetized with pentobarbital sodium (1 %, 0.4 ml/100 g; i.p.). Next, the blood was harvested from the heart, and the serum sample was stored at -80 °C until biochemical analyses were performed. The bilateral femurs and tibias were dissected from the animal body and kept at -80 °C until histological and biochemical analyses.

Serum biochemical marker analysis

The levels of serum Ca (S-Ca), and serum P (S-P) were measured by standard colorimetric methods using commercially available test kits. The serum concentration of tartrate-resistant acid phosphatase-5b (TRACP-5b), type I collagen C-terminal telopeptide (CTx-1), bone-specific alkaline phosphatase (BALP), and procollagen type I N-terminal propeptide (PINP) were determined using an appropriate enzyme-linked immunosorbent assay (ELISA) kit according to the manufacturer's instructions.

Bone histomorphometry analysis

The left proximal femurs (0.5 cm below the femoral head) were used to detect the bone mineral density (BMD, g/cm^2), bone volume per tissue volume (BV/TV, %), the thickness of trabeculae (Tb.Th, mm), number of trabeculae (Tb.N, 1/mm), and separating degree of trabeculae (Tb.Sp, mm). BMD was measured using dual-energy X-ray absorptiometry (DXA) (InAlyzer, Korea). BV/TV, Tb.Th, Tb.N, and Tb.Sp were measured by a microcomputed tomography (micro-CT) system (SkyScan, Belgium).

Hematoxylin and eosin staining

The morphology of the femur bone tissues was evaluated by hematoxylin and eosin (H&E) staining under a light microscope. Briefly, tissue samples were treated with 10 % formaldehyde, decalcified in 15 % neutral EDTA, followed by dehydration, paraffin embedding, and sectioning into 5 mm-thick sections. H&E staining was performed after rehydration following the protocols of Beijing Solarbio Science & Technology (China). Sections were differentiated in hydrochloric acid ethanol, rinsed in water, recovered in ammonia water, and then stained with eosin. Next, all samples were dehydrated, rendered transparent and sealed. Histological changes were observed using a light microscope. ImageJ software was used to visualize the stained trabecular bone sections.

Western blotting

Rat femur bone tissue was first pulverized using liquid nitrogen and subsequently immersed in the radioimmunoprecipitation (RIPA) lysis buffer (Beyotime, China) containing protease inhibitor (Beyotime, China) for 15 min on ice. Following centrifugation (12000 rpm, 20 min, and 4 °C), the resulting supernatants were harvested, and the protein concentration was measured using the Bradford assay. Each equal amount of protein sample (20 µg) was electrophoresed on a 12 % SDS-PAGE and transferred onto a polyvinylidene difluoride (PVDF) membrane (Millipore, USA), which was afterward blocked in 5 % skim milk for 2 h at room temperature. Subsequently, the membrane was incubated overnight at 4 °C with the following diluted primary antibodies: rat polyclonal anti-β-catenin antibody (1:2000), rat monoclonal anti-RUNX2 antibody (1:2000), rat monoclonal anti-Osterix antibody (1:2000), and rat monoclonal anti-β-actin antibody (1:5000) (Abcam, Cambridge, UK). Subsequently, blots were cultured at 25 °C for 1 h with secondary antibodies. Finally, blots were visualized using an Enhanced Chemiluminescence Substrate kit (Millipore, USA). The ImageJ software was used for densitometry analysis of the band intensity.

Statistical analysis

Data are expressed as the mean ± standard deviation (SD) and analyzed by GraphPad Prism 8.0. Comparisons between different groups were performed using the one-way analysis of variance (ANOVA) with Tukey's *post hoc* test. $p<0.05$ was considered statistically significant.

Results

LL-37 improved BMD and bone microstructure in OVX rats

As shown in Figure 1A-E, OVX significantly reduced the femoral BMD and impaired the bone microstructure in rats, including decreased BV/TV, Tb.Th, and Tb.N and increased Tb.Sp, whereas the administration of LL-37 for 12 weeks significantly increased the BMD and bone microstructure strength. Interestingly, XAV-939, a Wnt/β-catenin pathway inhibitor, significantly blocked the effect of LL-37 on BMD and bone microstructure in OVX rats. These results indicated that LL-37 functions to maintain the bone quality in OVX rats, and the Wnt/β-catenin pathway is an important regulator of LL-37 in osteoporosis.

LL-37 attenuated the bone loss in OVX rats

We further examined the levels of serum biochemical biomarkers closely related to bone metabolism and found that the levels of S-Ca and S-P remained unchanged in all groups. Compared with the sham group, the bone resorption markers TRACP-5b and CTx-1 and the bone formation markers PINP and BALP were significantly increased in OVX group, indicated

a high turnover pathology which has been always combined with a net bone loss in OVX-induced osteoporosis. Twelve weeks after the LL-37 treatment, the changes in these biochemical biomarkers were significantly attenuated, which were blocked by XAV-939 (Fig. 2A-F).

LL-37 attenuated the pathological injury in OVX rats

As shown in Figure 3, OVX resulted in disordered and thin trabeculae, empty bone lacunae, slight fractures, and considerably lower trabecular area as compared with the sham group, whereas the aberrant trabecular architecture was improved by LL-37 treatment, which was blocked by XAV-939. These results indicated that the osteoprotective effect of LL-37 was mediated by the maintenance of bone metabolism homeostasis, including the increase in bone formation and a reduction in bone resorption in OVX rats.

LL-37 activated the Wnt/β-catenin pathway in OVX rats

As shown in Figure 4, we examined the activity of the Wnt/β-catenin pathway using western blotting. We found that the expressions of β-catenin, Runx2, and Osterix were significantly decreased in OVX rats, indicating that the decreased activity of Wnt/β-catenin pathway may mediate the abnormal bone turnover in OVX rats. Whereas LL-37 significantly increased the expression of Wnt/β-catenin pathway when compared with the OVX group. Similarly, XAV-939 markedly blocked the effect of LL-37 on the Wnt/β-catenin pathway (Fig. 4A). These results indicated that LL-37 improved bone metabolic balance and promoted normal bone turnover in rats with OVX-induced osteoporosis by activating the Wnt/β-catenin pathway.

Discussion

Osteoporosis is a metabolic bone disease and is characterized by imbalanced bone formation and resorption [26,27]. Here, we showed that OVX induced osteoporosis in rats, along with pathological changes in BMD and trabecular microstructure, including the increased Tb.Sp and the decreased BMD, BV/TV, Tb.Th, and Tb.N. Twelve weeks after the LL-37 treatment, these changes in serum biochemical parameters and BMD and trabecular structure were significantly improved. In addition, the level of serum biochemical parameters, such as the bone resorption markers TRACP-5b and CTx-1 and the bone formation markers PINP and BALP were significantly decreased as compared with those in the OVX group after LL-37 treatment. These results indicated that LL-37 plays an anti-osteoporosis activity through inhibition of the high bone turnover in OVX-rats.

Cathelicidin has a variety of unique biological functions against pathogens and contributes to the induction and progression of infection, inflammation and cancer [28]. LL-37 is the mature form of human cathelicidin and has been reported to regulate bone homeostasis. A previous study reported that LL-37 directly suppressed osteoclastogenesis in humans and acted as a protector against bone resorption induced by a bacterial infection in periodontal diseases [29]. We found that LL-37 significantly attenuated bone loss and pathological injury by reducing overactive bone turnover and maintaining serum biochemical parameter homeostasis in OVX rats.

The formation of osteoporosis is an extremely complicated biological process involving multiple genes and factors. The Wnt/β-catenin pathway plays a crucial role in osteoporosis and significantly regulates bone formation and destruction by stimulating osteoblast generation and decreasing osteoclast differentiation. In the canonical Wnt/β-catenin pathway, β-catenin accumulates in the cytoplasm and enters the nucleus, where it activates the transcription of target genes and promotes bone formation [30]. Previous study has shown that Wnt/β-catenin pathway activity inhibition reduces osteogenic differentiation [31,32], whereas the activation of Wnt/β-catenin pathway accelerates osteogenic mineralization by promotion of the β-catenin nuclear accumulation [1]. Thus, the factors involved in this pathway could serve as potential targets of anti-osteoporosis drugs. LL-37 has been reported to regulate the Wnt/β-catenin pathway during tumorigenesis [25,33] and differentiation of adipose-derived stem cells [24]. However, whether the therapeutic role of LL-37 in OVX-induced osteoporosis is exerted *via* Wnt/β-catenin pathway remains unclear. We found that LL-37 significantly promoted the activation of β-catenin, whereas the Wnt/β-catenin pathway inhibitor XAV-939 blocked the effect of LL-37 on β-catenin, indicating that LL-37 protected against OVX-induced osteoporosis by activating the Wnt/β-catenin pathway. Meanwhile, the activated Wnt/β-catenin may inhibit overactive bone turnover and promote normal osteogenesis and osteoblast differentiation by inducing the expression of Runx2 [34]. Runx2 plays a key role in regulating osteoblastic function by controlling the transcription of its target genes. Recent

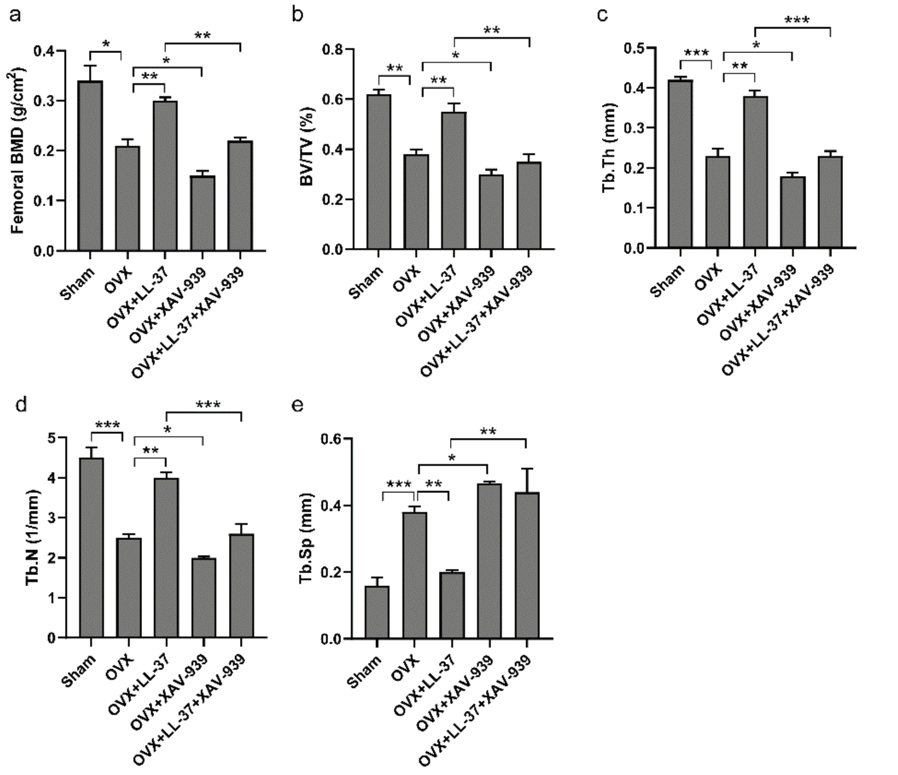

Fig. 1. Effect of LL-37 on BMD and bone microstructure in OVX rats. (**a**) Bone mineral density (BMD, g/cm^2). (**b**) Bone volume/tissue volume (BV/TV, %). (**c**) Trabecular thickness (Tb.Th, mm). (**d**) Trabecular number (Tb.N, 1/mm). (**e**) Trabecular separation (Tb.Sp, mm). All bar graphs are presented as mean ± SD. * p<0.05, ** p<0.01, *** p<0.01.

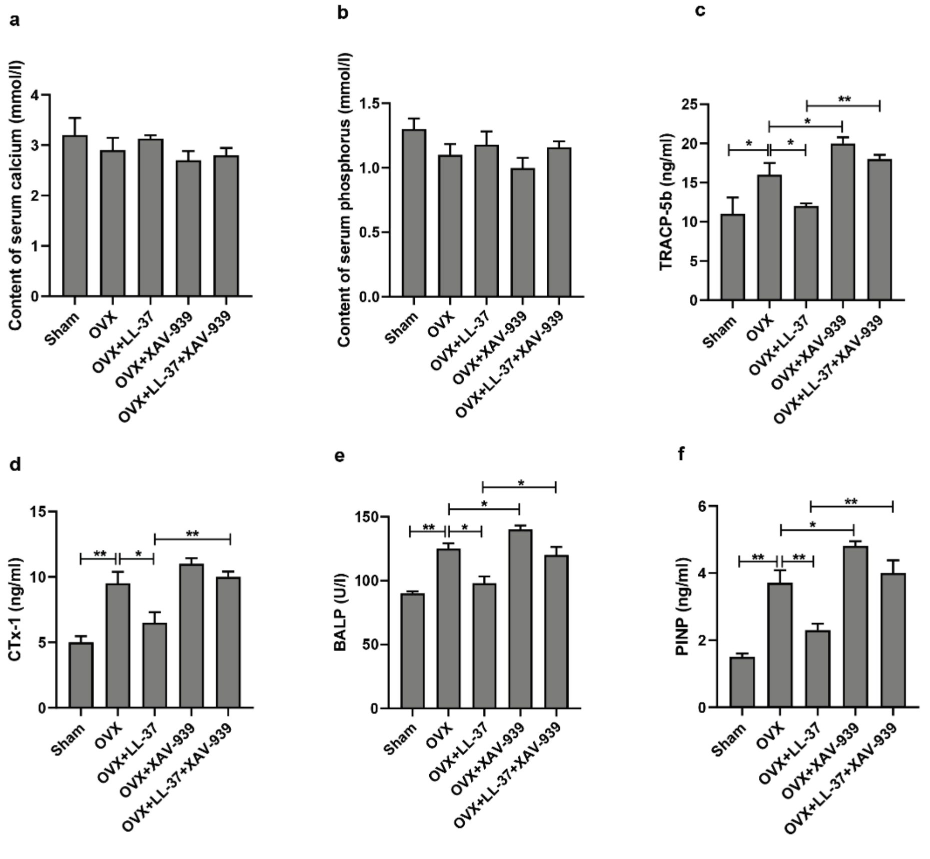

Fig. 2. Effect of LL-37 on bone loss in OVX rats. (**a**) The content of serum calcium (mmol/l). (**b**) The content of serum phosphorus (mmol/l). (**c**) The level of TRACP-5b (ng/ml). (**d**) The level of CTx-1 (ng/ml). (**e**) The level of BALP activity (U/l). (**f**) The level of PINP (ng/ml). All bar graphs are presented as mean ± SD. * p<0.05, ** p<0.01.

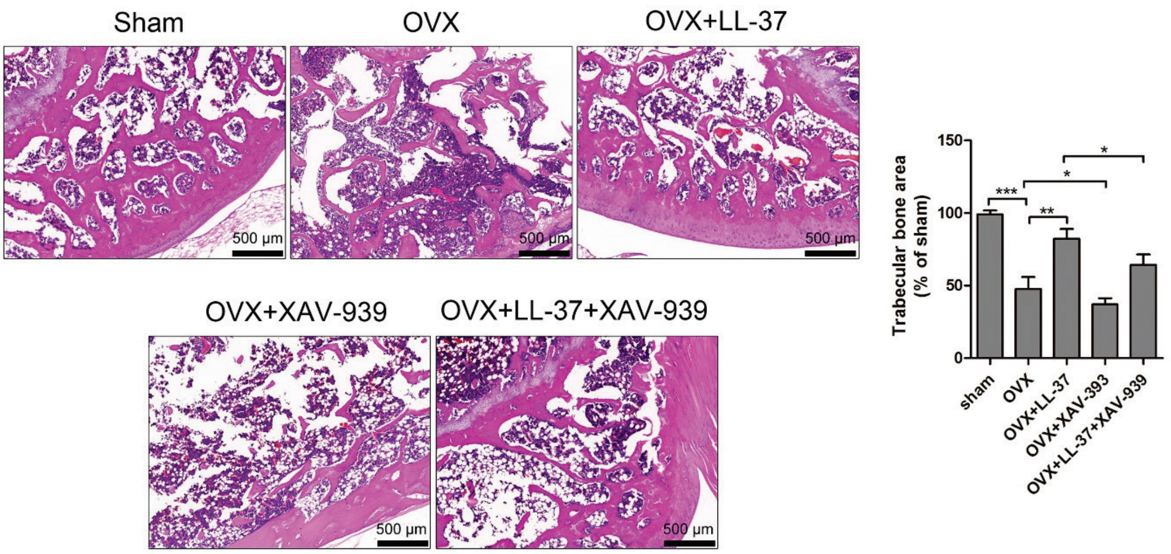

Fig. 3. Effect of LL-37 on pathological injury in OVX rats. Light microscopy of cortical and trabecular structures of the femur head (H&E staining, scale bar=500 μm).

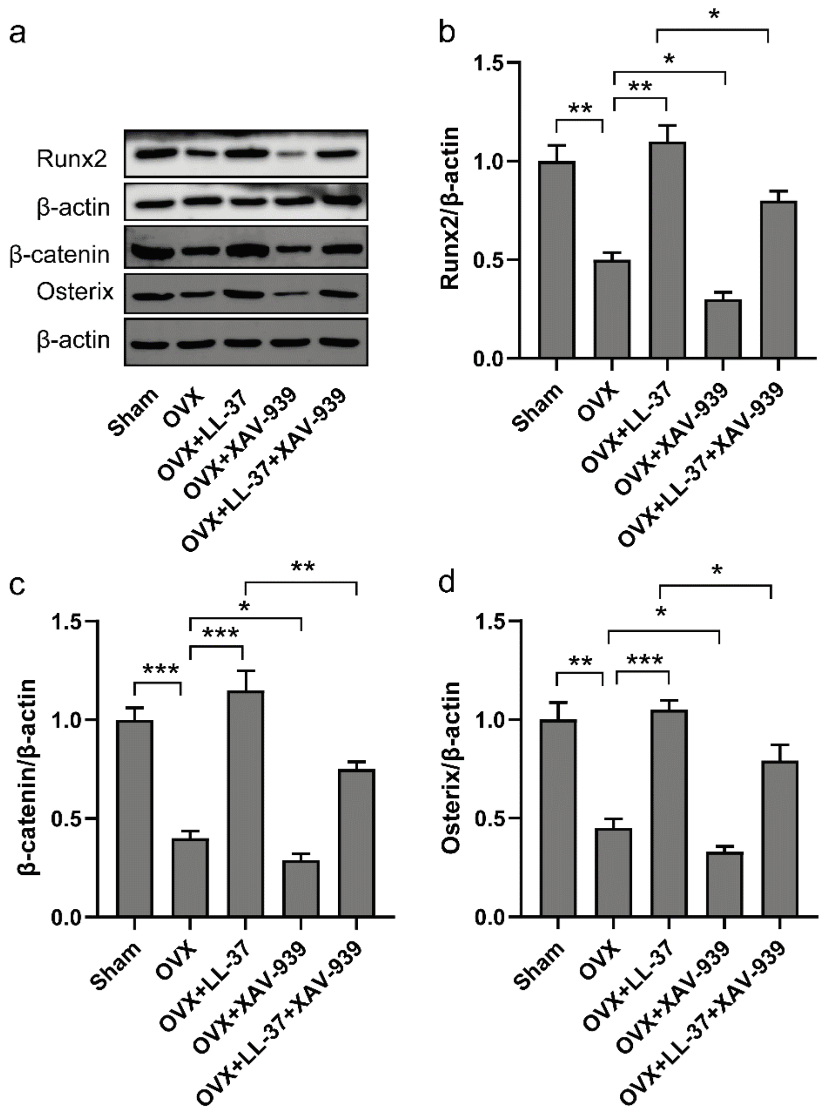

Fig. 4. Effect of LL-37 on the activity of Wnt/β-catenin pathway in OVX rats. (**a**) The expression of Runx2, β-catenin, and Osterix in the femur of rats. (**b**) Quantitative graphs of western blotting. All bar graphs are presented as mean ± SD. * p<0.05, ** p<0.01, *** p<0.01.

study has been reported that Runx2 induces the expression of the *COL1A1* gene encoding the primary component of collagen type I by interacting with Osterix [35], and plays an important role in bone homeostasis [36]. We showed that the expression of Runx2 and Osterix was significantly decreased in rats with osteoporosis, whereas LL-37 increased the expression of Runx2 and Osterix, which was blocked by XAV-939. In addition, XAV-939 abolished the effects of LL-37 on OVX rats. These results indicated that LL-37 attenuated bone loss and pathological injury by activating the Wnt/β-catenin pathway in an experimental animal model with osteoporosis.

In conclusion, our study revealed an important role of LL-37 in regulating OVX-induced osteoporosis. The results suggested that the Wnt/β-catenin pathway primarily mediates the protective role of LL-37.

Conflict of Interest

There is no conflict of interest.

References

1. Wang CG, Hu YH, Su SL, Zhong D. LncRNA DANCR and miR-320a suppressed osteogenic differentiation in osteoporosis by directly inhibiting the Wnt/beta-catenin signaling pathway. Exp Mol Med 2020;52:1310-1325. https://doi.org/10.1038/s12276-020-0475-0
2. Jin SL, Bai YM, Zhao BY, Wang QH, Zhang HS. Silencing of miR-330-5p stimulates osteogenesis in bone marrow mesenchymal stem cells and inhibits bone loss in osteoporosis by activating Bgn-mediated BMP/Smad pathway. Eur Rev Med Pharmacol Sci 2020;24:4095-4102. https://doi.org/10.26355/eurrev_202004_20987
3. Black DM, Rosen CJ. Postmenopausal osteoporosis. N Engl J Med 2016;374:2096-2097. https://doi.org/10.1056/NEJMc1602599
4 Jackson RD, Mysiw WJ. Insights into the epidemiology of postmenopausal osteoporosis: the Women's Health Initiative. Semin Reprod Med 2014;32:454-462. https://doi.org/10.1055/s-0034-1384629
5. Zhu S, He H, Zhang C, Wang H, Gao C, Yu X, He C. Effects of pulsed electromagnetic fields on postmenopausal osteoporosis. Bioelectromagnetics 2017;38:406-424. https://doi.org/10.1002/bem.22065
6. Baron R, Hesse E. Update on bone anabolics in osteoporosis treatment: rationale, current status, and perspectives. J Clin Endocrinol Metab 2012;97:311-325. https://doi.org/10.1210/jc.2011-2332
7. Brewer L, Williams D, Moore A. Current and future treatment options in osteoporosis. Eur J Clin Pharmacol 2011;67:321-331.https://doi.org/10.1007/s00228-011-0999-2
8. Anastasilakis AD, Polyzos SA, Makras P. Therapy of endocrine disease: Denosumab vs bisphosphonates for the treatment of postmenopausal osteoporosis. Eur J Endocrinol 2018;179:R31-R45. https://doi.org/10.1530/EJE-18-0056
9. Miller PD, Pannacciulli N, Brown JP, Czerwinski E, Nedergaard BS, Bolognese MA, Malouf J, Bone HG, Reginster JY, Singer A, Wang C, Wagman RB, Cummings SR. Denosumab or zoledronic acid in postmenopausal women with osteoporosis previously treated with oral bisphosphonates. J Clin Endocrinol Metab 2016;101:3163-3170. https://doi.org/10.1210/jc.2016-1801
10. Cosman F, Miller PD, Williams GC, Hattersley G, Hu MY, Valter I, Fitzpatrick LA, Riis BJ, Christiansen C, Bilezikian JP, Black D. Eighteen months of treatment with subcutaneous abaloparatide followed by 6 months of treatment with alendronate in postmenopausal women with osteoporosis: results of the ACTIVExtend trial. Mayo Clin Proc 2017;92:200-210. https://doi.org/10.1016/j.mayocp.2016.10.009
11. Bolognese M, Krege JH, Utian WH, Feldman R, Broy S, Meats DL, Alam J, Lakshmanan M, Omizo M. Effects of arzoxifene on bone mineral density and endometrium in postmenopausal women with normal or low bone mass. J Clin Endocrinol Metab 2009;94:2284-2289. https://doi.org/10.1210/jc.2008-2143
12. Yao Q, Yu C, Zhang X, Zhang K, Guo J, Song L. Wnt/beta-catenin signaling in osteoblasts regulates global energy metabolism. Bone 2017;97:175-183. https://doi.org/10.1016/j.bone.2017.01.028
13. Shen G, Ren H, Shang Q, Zhao W, Zhang Z, Yu X, Tang K, Tang J, Yang Z, Liang, Jiang X. Foxf1 knockdown promotes BMSC osteogenesis in part by activating the Wnt/beta-catenin signalling pathway and prevents ovariectomy-induced bone loss. EBioMedicine 2020;52:102626. https://doi.org/10.1016/j.ebiom.2020.102626

14. Huang Y, Xiao D, Huang S, Zhuang J, Zheng X, Chang Y, Yin D. Circular RNA YAP1 attenuates osteoporosis through up-regulation of YAP1 and activation of Wnt/beta-catenin pathway. Biomed Pharmacother 2020;129:110365. https://doi.org/10.1016/j.biopha.2020.110365
15. Sørensen O, Arnljots K, Cowland JB, Bainton DF, Borregaard N. The human antibacterial cathelicidin, hCAP-18, is synthesized in myelocytes and metamyelocytes and localized to specific granules in neutrophils. Blood 1997;90:2796-2803. https://doi.org/10.1182/blood.V90.7.2796
16. Pahar B, Madonna S, Das A, Albanesi C, Girolomoni G. Immunomodulatory role of the antimicrobial LL-37 peptide in autoimmune diseases and viral infections. Vaccines (Basel) 2020;8:517. https://doi.org/10.3390/vaccines8030517
17. Fabisiak A, Murawska N, Fichna J. LL-37: Cathelicidin-related antimicrobial peptide with pleiotropic activity. Pharmacol Rep 2016;68:802-808. https://doi.org/10.1016/j.pharep.2016.03.015
18. Kuroda K, Okumura K, Isogai H, Isogai E. The human cathelicidin antimicrobial peptide LL-37 and mimics are potential anticancer drugs. Front Oncol 2015;5:144. https://doi.org/10.3389/fonc.2015.00144
19. Koczulla R, von Degenfeld G, Kupatt C, Krötz F, Zahler S, Gloe T, Issbrücker K, Unterberger P, Zaiou M, Lebherz C, Karl A, Raake P, Pfosser A, Boekstegers P, Welsch U, Hiemstra PS, Vogelmeier C, Gallo RL, Clauss M, Bals R. An angiogenic role for the human peptide antibiotic LL-37/hCAP-18. J Clin Invest 2003;111:1665-1672. https://doi.org/10.1172/JCI17545
20. Barlow PG, Beaumont PE, Cosseau C, Mackellar A, Wilkinson TS, Hancock RE, Haslett C, Govan JR, Simpson AJ, Davidson DJ. The human cathelicidin LL-37 preferentially promotes apoptosis of infected airway epithelium. Am J Respir Cell Mol Biol 2010;43:692-702. https://doi.org/10.1165/rcmb.2009-0250OC
21. Ramos R, Silva JP, Rodrigues AC, Costa R, Guardão L, Schmitt F, Soares R, Vilanova M, Domingues L, Gama M. Wound healing activity of the human antimicrobial peptide LL37. Peptides 2011;32:1469-1476. https://doi.org/10.1088/1475-7516/2011/06/005
22. Otte JM, Zdebik AE, Brand S, Chromik AM, Strauss S, Schmitz F, Steinstraesser L, Schmidt WE. Effects of the cathelicidin LL-37 on intestinal epithelial barrier integrity. Regul Pept 2009;156:104-117. https://doi.org/10.1016/j.regpep.2009.03.009
23. Supanchart C, Thawanaphong S, Makeudom A, Bolscher JG, Nazmi K, Kornak U, Krisanaprakornkit S. The antimicrobial peptide, LL-37, inhibits in vitro osteoclastogenesis. J Dent Res 2012;91:1071-1077. https://doi.org/10.1177/0022034512460402
24. Cheng Q, Zeng K, Kang Q, Qian W, Zhang W, Gan Q, Xia W. The antimicrobial peptide LL-37 promotes migration and odonto/osteogenic differentiation of stem cells from the apical papilla through the Akt/Wnt/β-catenin signaling pathway. J Endod 2020;46:964-972. https://doi.org/10.1016/j.joen.2020.03.013
25. Ji P, Zhou Y, Yang Y, Wu J, Zhou H, Quan W, Sun J, Yao Y, Shang A, Gu C, Zeng B, Firrman J, Xiao W, Bals R, Sun Z, Li D. Myeloid cell-derived LL-37 promotes lung cancer growth by activating Wnt/β-catenin signaling. Theranostics 2019;9:2209-2223. https://doi.org/10.7150/thno.30726
26. Mei F, Meng K, Gu Z, Yun Y, Zhang W, Zhang C, Zhong Q, Pan F, Shen X, Xia G, Chen H. Arecanut (Areca catechu L.) seed polyphenol-ameliorated osteoporosis by altering gut microbiome via LYZ and the immune system in estrogen-deficient rats. J Agric Food Chem 2021;69:246-258. https://doi.org/10.1021/acs.jafc.0c06671
27. Behera J, Ison J, Tyagi SC, Tyagi N. The role of gut microbiota in bone homeostasis. Bone 2020;135:115317. https://doi.org/10.1016/j.bone.2020.115317
28. Chow JY, Li ZJ, Wu WK, Cho CH. Cathelicidin a potential therapeutic peptide for gastrointestinal inflammation and cancer. World J Gastroenterol 2013;19:2731-2735. https://doi.org/10.3748/wjg.v19.i18.2731
29. Nakamichi Y, Horibe K, Takahashi N, Udagawa N. Roles of cathelicidins in inflammation and bone loss. Odontology 2014;102:137-146. https://doi.org/10.1007/s10266-014-0167-0
30. Liu H, Guo Y, Zhu R, Wang L, Chen B, Tian Y, Li R, Ma R, Jia Q, Zhang H, Xia B, Li Y, Wang X, Zhu X, Zhang R, Brömme D, Gao S, Zhang D, Pei X. Fructus Ligustri Lucidi preserves bone quality through induction of canonical Wnt/β-catenin signaling pathway in ovariectomized rats. Phytother Res 2021;35:424-441. https://doi.org/10.1002/ptr.6817
31. Manolagas SC. Wnt signaling and osteoporosis. Maturitas 2014;78:233-237. https://doi.org/10.1016/j.maturitas.2014.04.013

32. Saidak Z, Le Henaff C, Azzi S, Marty C, Da Nascimento S, Sonnet P, Marie PJ. Wnt/β-catenin signaling mediates osteoblast differentiation triggered by peptide-induced α5β1 integrin priming in mesenchymal skeletal cells. J Biol Chem 2015;290:6903-6912. https://doi.org/10.1074/jbc.M114.621219
33. Pan XH, Quan WW, Wu JL, Xiao WD, Sun ZJ, Li D. Antimicrobial peptide LL-37 in macrophages promotes colorectal cancer growth. (Article in Chinese) Zhonghua Zhong Liu Za Zhi 2018;40:412-417. https://doi.org/10.3760/cma.j.issn.0253-3766.2018.06.003
34. Komori T. Regulation of proliferation, differentiation and functions of osteoblasts by Runx2. Int J Mol Sci 2019;20:1694. https://doi.org/10.3390/ijms20071694
35. Ortuño MJ, Susperregui AR, Artigas N, Rosa JL, Ventura F. Osterix induces Col1a1 gene expression through binding to Sp1 sites in the bone enhancer and proximal promoter regions. Bone 2013;52:548-556. https://doi.org/10.1016/j.bone.2012.11.007
36. Perez-Campo FM, Santurtun A, Garcia-Ibarbia C, Pascual MA, Valero C, Garces C, Sanudo C, Zarrabeitia MT, Riancho JA. Osterix and RUNX2 are transcriptional regulators of sclerostin in human bone. Calcif Tissue Int 2016;99:302-309. https://doi.org/10.1007/s00223-016-0144-4
