# Supplementary material for: Molecular Regulators for the Wnt/β-Catenin Signalling Pathway for Bone Remodelling in Periodontitis: A Scoping Review
Source: Int J Mol Sci. 2026 Oct 14;27(20):9117. doi: 10.3390/ijms27209117 (PMC13705578; doi:10.3390/ijms27209117)
Supplement: Supplementary file 1 [file ijms-27-09117-s001.zip › Supplementary Data/Papers/in vivo/Comparative evaluation of sclerostin levels in gingival crevicular fluid in the treatment of chronic periodontitis patients using diode laser as an adjunct to scaling and root planing A clinico-biochemical study.pdf]

Original Article

Comparative Evaluation of Sclerostin Levels in Gingival Crevicular Fluid in the Treatment of Chronic Periodontitis Patients Using Diode Laser as an Adjunct to Scaling and Root Planing: A Clinico-biochemical Study

Abstract

Aims: Sclerostin is an inhibitor of bone formation, and laser irradiation enhances osteoblast proliferation. The objective of this study was to assess and compare the gingival crevicular fluid (GCF) sclerostin level and clinical parameters of chronic periodontitis patients following the application of diode laser (810 nm) as an adjunct to scaling and root planing (SRP). **Subjects and Methods:** Fifteen systemically healthy chronic periodontitis patients (age 35–55 years) with probing pocket depth ≥5mm were included in this split-mouth study. SRP and pocket irradiation with diode laser were done in the test group and SRP alone in the control group at baseline. Low-level laser therapy application and saline irrigation were done in both the groups, respectively, in the 2nd and 3rd visits. Two microliters of GCF samples was collected from both the groups at baseline before treatment and on the 90th day for the assessment of sclerostin concentration. **Results:** This study showed a statistically significant reduction of clinical parameters in the test and control groups at the end of 3 months. Both the groups showed a statistically significant reduction of sclerostin levels in GCF after 3 months, in which the test group (125.80 ± 28.21 to 82.80 ± 9.31) showed a highly significant reduction (P = 0.000). **Conclusions:** The adjunctive use of laser had shown a beneficial effect in terms of clinical parameters and osteoblast proliferation by the reduction in the levels of sclerostin in GCF. From the observations of this study, it can be concluded that the therapeutic effectiveness of diode laser as an adjunct to SRP is having a beneficial effect and sclerostin can be used as a potent biomarker.

Keywords: *Biomarker, chronic periodontitis, enzyme-linked immunosorbent assay, gingival crevicular fluid, low-level laser therapy, nonsurgical periodontal therapy, pocket irradiation, sclerostin*

B. S. Jagadish Pai, Nithya R. Krishnan, Amit Walveker, Sreedevi Keeneri, Ansu Emmanuel, Neethi R Krishnan, Menezes Anosca Lira

Department of Periodontics and Implantology, Coorg Institute of Dental Sciences, Virajpet, Karnataka, India

Submitted: 09-Jan-2020
Revised: 17-Apr-2020
Accepted: 23-May-2020
Published: 21-Sep-2021

Address for correspondence:
Dr. Sreedevi Keeneri, Department of Periodontics and Implantology, Coorg Institute of Dental Sciences, Virajpet, Karnataka, India.
E-mail: Sreedevikeeneri@gmail.com

Access this article online
Website: www.contempclindent.org
DOI: 10.4103/ccd.ccd_19_20
Quick Response Code:

Introduction

Periodontal disease is a biologic process related to the interaction between microorganisms and the host immune response.[1] When the balance is disturbed, periodontal breakdown occurs[1,2] resulting in activation of the bone resorption.[2,3] The pathway of bone resorption is closely related to interaction of the tumor necrosis factor superfamily receptor-associated nuclear factor kappa-beta, receptor-associated nuclear factor kappa-beta ligand (RANKL), and osteoprotegerin (OPG).[4]

Bone metabolism is regulated by the Wnt signaling pathway by increasing bone formation and regeneration.[5-8] Sclerostin, a SOST gene product, is a secreted glycoprotein that binds with low-density lipoprotein receptor-related protein-5 and blocks this pathway[9,10] which is produced by osteocytes and is a negative regulator of osteoblast differentiation[11] and a marker of mature osteocyte by promoting osteoclast formation. The studies on the use of Wnt signal-enhancing agents to prevent bone loss and regenerate supporting tissue showed as a promising alternative therapy.[12]

Homeostasis of bone is maintained by a balance between osteoblastic and osteoclastic bone formation and bone resorption, respectively, where intracellular reactive oxygen species (ROS) are crucial mediators of osteoclastogenesis. Light-emitting diode irradiation downregulates osteoclastogenesis by reducing ROS production. Recently,

This is an open access journal, and articles are distributed under the terms of the Creative Commons Attribution-NonCommercial-ShareAlike 4.0 License, which allows others to remix, tweak, and build upon the work non-commercially, as long as appropriate credit is given and the new creations are licensed under the identical terms.

For reprints contact: WKHLRPMedknow_reprints@wolterskluwer.com

How to cite this article: Pai BS, Krishnan NR, Walveker A, Keeneri S, Emmanuel A, Krishnan NR, *et al.* Comparative evaluation of sclerostin levels in gingival crevicular fluid in the treatment of chronic periodontitis patients using diode laser as an adjunct to scaling and root planing: A clinico-biochemical study. Contemp Clin Dent 2021;12:276-81.

low-level light therapy, used in various clinical fields, was shown to alleviate oxidative stress by scavenging intracellular ROS.[13]

Considering the potential role of sclerostin in bone metabolism, till date no studies have evaluated the level of sclerostin in gingival crevicular fluid (GCF) after laser irradiation and low-level laser therapy (LLLT) in chronic periodontitis patients. So the aim of this study was to evaluate the role of sclerostin as a biomarker in nonsurgical periodontal therapy, the effect of laser irradiation on osteoblastic proliferation, and the level of sclerostin. The study also aims to evaluate the benefits of LLLT as an adjunctive therapy. Hence, the aim of this study is to assess the effect of diode laser as an adjunctive therapy in the treatment of periodontitis by comparative evaluation of sclerostin in GCF.

Subjects and Methods

Source of data

Fifteen patients with chronic periodontitis (age 35–55) reporting to the Outpatient Department of Periodontics, Coorg Institute of Dental Sciences, Virajpet, were enrolled for the study. The nature and purpose of the study and the treatment protocol was explained to the participants included in the study, and written consent was obtained before commencing the study.

Inclusion criteria

- Patients diagnosed with chronic periodontitis and systemically healthy untreated chronic periodontitis
- Patients with minimum of 20 numbers of teeth excluding third molar, with pocket depth ≥5 mm
- Patients ranging from 35 to 55 years of age
- Patients willing to participate in the study.

Exclusion criteria

- Patients with systemic diseases (e.g., diabetes mellitus, bone-related diseases that compromise sclerostin, OPG, or RANKL levels: osteoporosis and collagen-metabolic diseases or disorders), pregnant and lactating women, smokers (within the past 5 years), and patients not willing for surgery.

Method of collection of data

This was a split-mouth, single-blinded, randomized clinical trial in which 15 patients (with total of 30 sites) with probing pocket depth (PPD) more than or equal to 5 mm were included [Figures 1 and 2]. The sites for control and test groups were selected by coin toss method wherein head was grouped into control group and tail as test group. Individual sites were categorized into two groups as follows:

- Control (Group 1) – Chronic periodontitis patients (sites having more than or equal to 5mm of PPD) treated by scaling and root planing (SRP) alone
- Test (Group 2) – Chronic periodontitis patients (sites having more than or equal to 5 mm of PPD) treated by diode laser as an adjunct to SRP [Figure 3]
- The patients were reviewed in a series of 4 appointments. In the first visit, GCF collection was

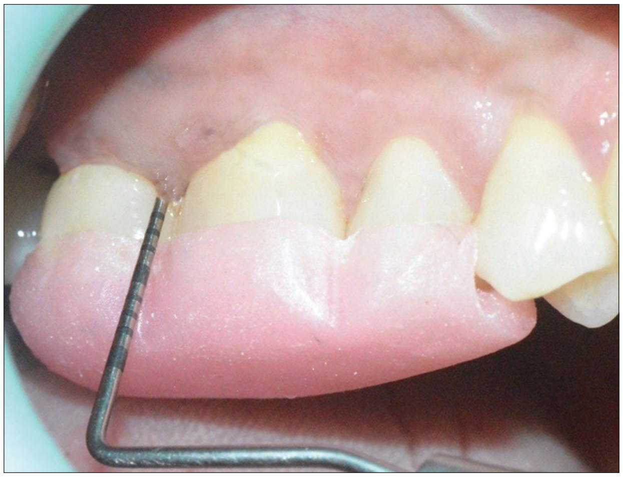

Figure 1: Baseline probing depth of the test group

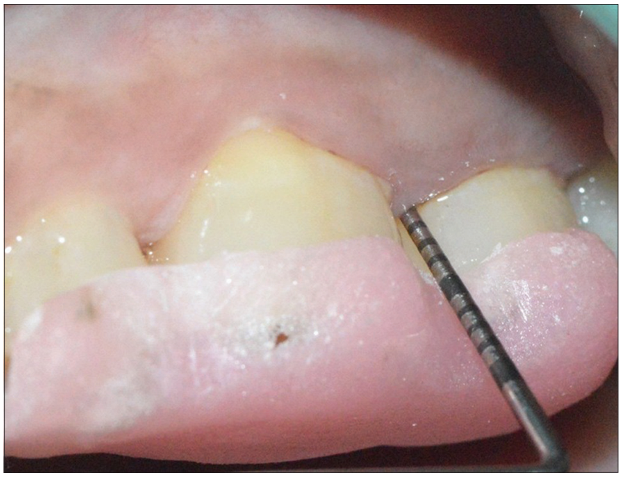

Figure 2: Baseline probing depth of the control group

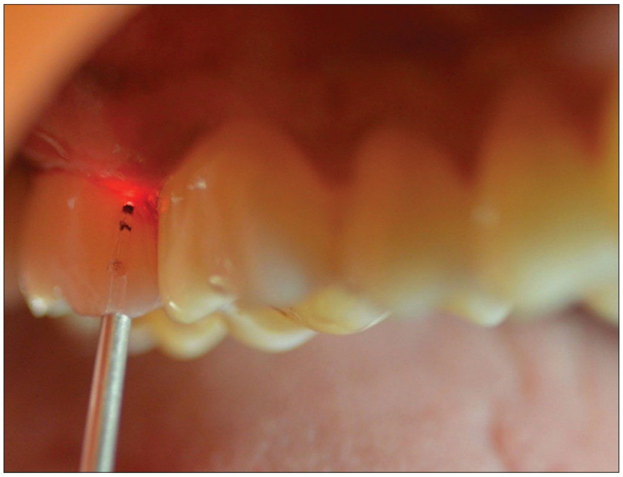

Figure 3: Laser application on the test group

done in both the test and control groups before the treatment. The test group was treated by diode laser as an adjunct to SRP and the control group was treated by SRP alone. The 2nd and 3rd appointments were scheduled at the 7th and 30th days, respectively, from the baseline. LLLT application and saline irrigation were done in the test and control groups, respectively, in the 2nd and 3rd visits [Figures 4 and 5].

Clinical parameters assessed were:
- Gingival index (Loe and Sillness, 1963) (GI)
- Bleeding index (BI) (Muhlemann and Son, 1971)
- PPD
- Clinical attachment level (CAL).

Collection of sample

Samples of GCF were obtained using microcapillary pipettes. GCF samples were collected from the same site of the test and control groups at baseline, before treatment, and on the 90th day for the assessment of sclerostin concentration in GCF. From each site, a standardized volume of 2μL GCF was collected and transferred to vials containing phosphate-buffered saline. These samples are then stored in a freezer (liquid nitrogen) at −80°C.

Laser application procedure:
- The gingival mucosa was subjected to treatment with a diode laser (AMD Picasso® DENTSPLY India Pvt. Ltd.) operating at a wavelength of 810 nm, 0.1 W output power, continuous wave, which is equipped with an optical fiber (300 μm). Irradiation was performed in contact mode, the fiber tip touching the gingiva, to remove the junctional, sulcular, and outer gingival epithelium (approximately 0.5 mm from the gingival margin) all around the teeth. To minimize gingival damage, the tip was moved at a constant speed of 1 mm/s. Laser application was done on the 7th and 30th days using the same diode laser for 3 cycles [Figure 3]
- Clinical measurements and treatment were performed by a single examiner, whereas biochemical assessment was done by another individual.

Biomarker analysis

Concentration of sclerostin in GCF was determined using enzyme-linked immunosorbent assay (ELISA) based on the biotin double-antibody sandwich technology according to manufacturer's directions. SSamples were then assayed for sclerostin using ELISA kit (ElabScience)obtained from Everon Life Sciences(India).

Statistical analysis

All the samples were analyzed by paired *t*-test. Descriptive and inferential analysis had been carried out in the present study. Results on continuous measurements were presented as mean ± standard deviation (SD) and results on categorical measurements were presented in percentages. The following assumptions on data were made:

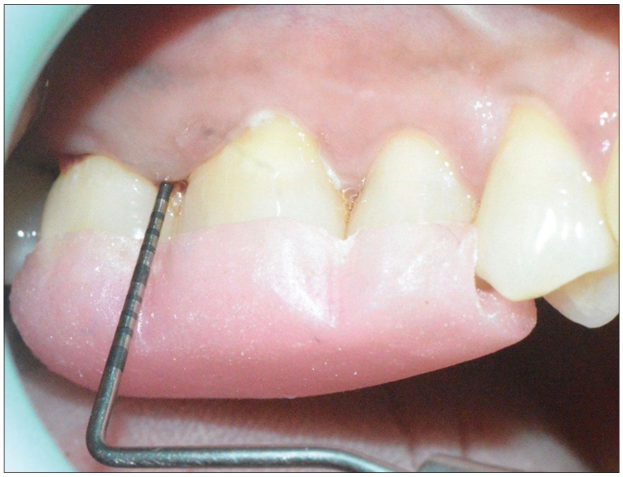

Figure 4: 3 months postoperative probing depth on the test group

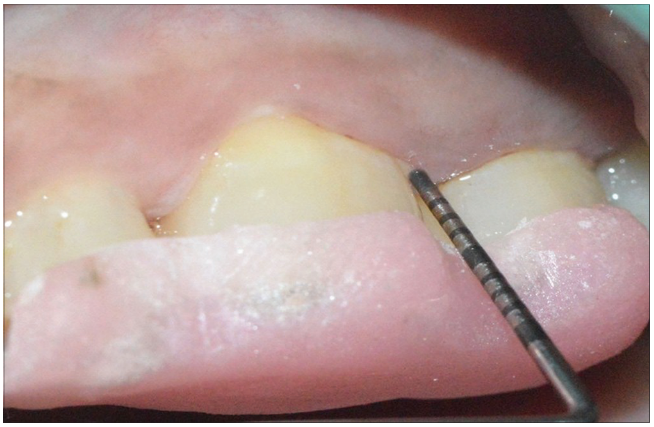

Figure 5: 3 months postoperative probing depth on the control group

1. Dependent variables should be normally distributed
2. Samples drawn from the population should be random, and cases of the samples should be independent
3. The data were collected, coded, and fed in statistical software SPSS (IBM version 23), IBM Corp., Armonk, NY, USA) and Microsoft Excel for statistical analysis. Descriptive statistics include mean, SD, frequency, and percentage. Inferential statistics include paired *t*-test, independent *t*-test, and Chi-square test for the comparison. The level of significance was set at 0.05 at 95% confidence interval

- Suggestive significant: $P < 0.05$
- Highly significant: $P < 0.0$.

Results

In a total of 15 participants, 8 were females and 7 were males, with a mean age of 38.33 ± 3.14. The intragroup comparison of clinical parameters and sclerostin levels in the test and control groups at baseline and 3 months was given in Tables 1 and 2, respectively. The intergroup comparison of clinical parameters and levels of sclerostin between the test and control groups at baseline and 3 months was given in Tables 3 and 4, respectively.

Discussion

This is the first clinical study that has examined the changes in GCF sclerostin levels in patients with chronic periodontitis treated with diode laser as an adjunct to nonsurgical therapy.

The effectiveness of SRP in the treatment of periodontal disease in order to reduce bacterial plaque on the root surface is universally accepted. However, conventional methods for the treatment of periodontal disease are not completely effective in eliminating all types of bacteria and inflammatory response of the tissue. The photophysical characteristics of lasers and laser irradiation exhibit strong ablation, hemostasis, detoxification, and bactericidal effects on the human body. Thus, in periodontal therapy, laser treatment may serve as an alternative or adjunctive therapy to mechanical approaches.[14] A study by Gold *et al.*[15] demonstrated that the application of laser for curettage of pocket epithelium does not cause damage to underlying tissue layers. Histologic sections revealed complete removal of the pocket epithelium without necrosis and carbonization of the connective tissue structures in 83% of the cases.

In view with the above, this study was carried out with the objective to evaluate the efficacy of diode laser therapy in periodontal pockets with regard to its action on levels of sclerostin, which is a marker of mature osteocytes and affects bone metabolism by inhibiting osteoblast differentiation, as an adjunct to SRP and to compare the results with SRP alone over a period of 90 days.

This clinical trial shows that the adjunctive use of diode laser with nonsurgical periodontal therapy in patients with chronic periodontitis did enhance the response of clinical parameters such as bleeding on probing, PPD, and CAL as measured 90 days after treatment.

In this study, a statistically significant reduction of gingival index is shown in both the groups at the end of 3 months; however, the test group showed a mean gingival index of 1.433 ± 0.671 at baseline and 0.366 ± 0.498 at 3 months and the control group showed a value of 1.283 ± 0.507 at baseline and 0.533 ± 0.351 at 3 months.

Bleeding on probing reduced significantly in both the groups at the end of 3 months, however, the test group showed a highly significant reduction compared to test group reduction of BI at baseline to 3rd month: 3.33-5.33 control croup BI at baseline to 3rd month 3.4-2.53 better significant reduction in test group. Reduced bleeding on probing can be attributed to SRP and patient education and motivation in both the groups, however, greater reduction in the test group can be attributed to the use of diode laser as an adjunct to SRP. Our findings are in accordance with Badersten *et al.*[16] and Claffey *et al.*[17] who suggested the potential role of diode laser as a modulatory therapy in the treatment of periodontal disease.

Table 1: Intragroup comparison of clinical parameters and sclerostin levels in the test group at baseline and 3 months

Test group	Mean±SD	*T*	*P*
GI			
Baseline	1.4333±0.67126	5.101	0.000 (HS)
3rd month	0.3667±0.49881		
BI			
Baseline	3.3333±0.48795	16.039	0.000 (HS)
3rd month	0.5333±0.63994		
PPD (in mm)			
Baseline	5.20±0.414	12.160	0.000 (HS)
3rd month	2.60±0.737		
CAL (in mm)			
Baseline	5.40±0.507	9.539	0.000 (HS)
3rd month	2.80±1.014		
SOST (in pg/ml)			
Baseline	125.80±28.21145	5.581	0.000 (HS)
3rd month	82.80±9.31358		

P<0.05, HS. HS: Highly significant, SD: Standard deviation; CAL: Clinical attachment level; GI: Gingival index; BI: Bleeding index; PPD: Probing pocket depth; SOST: Sclerostin

Table 2: Intragroup comparison of clinical parameters and sclerostin levels in the control group at baseline and 3 months

Control	Mean±SD	*T*	*P*
GI			
Baseline	1.2833±0.50768	5.196	0.000 (HS)
3rd month	0.5333±0.35187		
BI			
Baseline	3.4000±0.63246	4.516	0.000 (HS)
3rd month	2.5333±0.74322		
PPD (in mm)			
Baseline	5.13±0.352	10.458	0.000 (HS)
3rd month	3.47±0.640		
CAL (in mm)			
Baseline	5.33±0.488	7.906	0.000 (HS)
3rd month	3.67±0.724		
SOST (in pg/ml)			
Baseline	146.4667±49.78506	2.266	0.040 (S)
3rd month	140±41.930		

P<0.05. HS: Highly significant; S: Significant; SD: Standard deviation; CAL: Clinical attachment level; GI: Gingival index; BI: Bleeding index; PPD: Probing pocket depth; SOST: Sclerostin

A significant reduction in PPD was found in both the groups at 90 days postoperative in this study. However, when compared, the test group (5.20 ± 0.414 at baseline and 2.60 ± 0.737 after 3 months) showed a more significant reduction in PPD than the control group (5.13 ± 0.352 at baseline and 3.47 ± 0.640 after 3 months) with $P = 0.002$. These results are in accordance with the findings of Mortiz *et al.*[18] in 1998 who found a significant reduction in BOP and PPD values in the laser-treated sites than sites treated with SRP with normal saline irrigation alone.

Table 3: Intergroup comparison of clinical parameters and levels of sclerostin between the test and control groups at baseline

Baseline	Mean±SD	*T*	*P*
GI			
Control	1.2833±0.50768	−0.6990	0.496 (NS)
Test	1.4333±0.67126		
BI			
Control	3.4000±0.63246	0.323	0.749 (NS)
Test	3.3333±0.48795		
PPD (in mm)			
Control	5.13±0.352	−0.475	0.345 (NS)
Test	5.20±0.414		
CAL (in mm)			
Control	5.33±0.488	−0.367	0.478 (NS)
Test	5.40±0.507		
SOST (in pg/ml)			
Control	146.4667±49.78506	1.399	0.015 (S)
Test	125.80±28.21145		

$P<0.05$. S: Significant; NS: Nonsignificant; SD: Standard deviation; CAL: Clinical attachment level; GI: Gingival index; BI: Bleeding index; PPD: Probing pocket depth; SOST: Sclerostin

Table 4: Intergroup comparison of clinical parameters and levels of sclerostin between the test and control groups at 3 month

3rd month	Mean±SD	*T*	*P*
GI			
Control	0.5333±0.35187	1.057	0.247 (NS)
Test	0.3667±0.49881		
BI			
Control	2.5333±0.74322	7.898	0.000 (HS)
Test	0.5333±0.63994		
PPD (in mm)			
Control	3.47±0.640	3.439	0.002 (HS)
Test	2.60±0.737		
CAL (in mm)			
Control	3.67±0.724	2.649	0.012 (S)
Test	2.80±1.014		
SOST (in pg/ml)			
Control	140.6±41.930	5.212	0.000 (HS)
Test	82.80±9.314		

$P<0.05$. HS: Highly significant; S: Significant; SD: Standard deviation; CAL: Clinical attachment level; GI: Gingival index; BI: Bleeding index; PPD: Probing pocket depth; SOST: Sclerostin

Although CAL gain was achieved in both the groups, the test group showed a significant gain in CAL (5.40 ± 0.507 at the baseline and 2.80 ± 1.014 after 3 months) than the control group (5.33 ± 0.488 at baseline and 3.67 ± 0.724 after 3 months) at the end of 3 months. Our results are in accordance with those of Kiesler *et al.* 2005 who found a greater reduction in probing depth and increase of attachment gain with the adjunctive application of laser compared to SRP alone.

In this study, the levels of sclerostin were analyzed in GCF samples by ELISA test. The GCF was collected by placing calibrated volumetric microcapillary pipettes and transferred to vials containing phosphate-buffered saline. The collected GCF samples were stored in a freezer (liquid nitrogen) at −80°C.

The result of this study showed that sclerostin levels in GCF were higher in the control group (146.46 ± 49.78) than in the test group (125.80 ± 28.21) at baseline. After 3 months, sclerostin levels in GCF of the control group were reduced to 140.6 ± 41.93 and the test group to 82.80 ± 9.31. Both the groups have shown a statistically significant reduction of sclerostin levels in GCF after 3 months in which the test group has shown a highly significant reduction with P = of 0.000. The present study results are consistent with the findings of Balli *et al.* that nonsurgical periodontal therapy resulted in decreased levels of sclerostin but had no effect on the RANKL/OPG ratio, despite an improvement in clinical parameters. In addition, a reduction of the level of sclerostin was directly associated with improved clinical outcomes. Considering the levels of sclerostin in periodontitis patients before and after treatment, it is possible to speculate that sclerostin is involved in alveolar bone loss and that measurement of this protein is useful for monitoring the response to nonsurgical periodontal treatment.[19]

It was also reported that sclerostin levels were correlated positively with PD and CAL.[20] This is consistent with the finding of our study that GCF sclerostin levels had a strong positive correlation with PPD, CAL, and BI. Sufia *et al.*[21] observed a higher reduction of sclerostin levels in intrabony defects treated with open-flap debridement and LLLT compared to open-flap debridement alone. This could be due to added effect of LLLT biostimulation on osteoblasts, thereby leading to increased cell proliferation. LLLT irradiation induces enhanced osteoblast proliferation, intracellular metabolic changes resulting in faster cell division, proliferation, and migration of fibroblasts.

Conclusions

From the abovementioned studies, it is evident that the sclerostin can be used as a potent biomarker for monitoring the response of nonsurgical periodontal therapy. A beneficial effect from adjunctive laser application in terms of clinical parameters was observed. The effect of lasers on osteoblast proliferation is shown in this study by the reduction in the levels of sclerostin in GCF. The application of laser can constitute an alternative device as an adjunct to maintain periodontal health in chronic periodontitis patients.

Financial support and sponsorship

Nil.

Conflicts of interest

There are no conflicts of interest.

References

1. Kornman KS, Page RC, Tonetti MS. The host response to the microbial challenge in periodontitis: assembling the players. Periodontol 2000 1997;14:33-53.
2. Bascones-Martínez A, Muñoz-Corcuera M, Noronha S, Mota P, Bascones-Ilundain C, Campo-Trapero J. Host defence mechanisms against bacterial aggression in periodontal disease: Basic mechanisms. Med Oral Patol Oral Cir Bucal 2009;14:e680-5.
3. Buduneli N, Kinane DF. Host-derived diagnostic markers related to soft tissue destruction and bone degradation in periodontitis. J Clin Periodontol 2011;38 Suppl 11:85-105.
4. Hofbauer LC, Heufelder AE. Role of receptor activator of nuclear factor-kappaB ligand and osteoprotegerin in bone cell biology. J Mol Med (Berl) 2001;79:243-53.
5. Karim R, Tse G, Putti T, Scolyer R, Lee S. The significance of the Wnt pathway in the pathology of human cancers. Pathology 2004;36:120-8.
6. Monroe DG, McGee-Lawrence ME, Oursler MJ, Westendorf JJ. Update on Wnt signaling in bone cell biology and bone disease. Gene 2012;492:1-8.
7. Galli C, Passeri G, Macaluso GM. Osteocytes and WNT: The mechanical control of bone formation. J Dent Res 2010;89:331-43.
8. Zuo C, Huang Y, Bajis R, Sahih M, Li YP, Dai K, *et al*. Osteoblastogenesis regulation signals in bone remodeling. Osteoporos Int 2012;23:1653-63.
9. Baron R, Rawadi G. Targeting the Wnt/beta-catenin pathway to regulate bone formation in the adult skeleton. Endocrinology 2007;148:2635-43.
10. Silverman SL. Sclerostin. J Osteoporos 2010;2010:941419.
11. Taut AD, Jin Q, Chung JH, Galindo-Moreno P, Yi ES, Sugai JV, *et al*. Sclerostin antibody stimulates bone regeneration after experimental periodontitis. J Bone Miner Res 2013;28:2347-56.
12. Khosla S. Minireview: The OPG/RANKL/RANK system. Endocrinology 2001;142:5050-5.
13. Sohn H, Ko Y, Park M, Kim D, Moon YL, Jeong YJ, *et al*. Effects of light-emitting diode irradiation on RANKL-induced osteoclastogenesis Lasers Surg Med. 2015;47:745-5.
14. Lang NP, Brägger U. Periodontal diagnosis in the 1990s. J Clin Periodontol 1991;18:370-9.
15. Gold SI, Vilardi MA. Pulsed laser beam effects on gingiva. J Clin Periodontol 1994;21:391-6.
16. Badersten A, Nilvéus R, Egelberg J. Effect of nonsurgical periodontal therapy. I. Moderately advanced periodontitis. J Clin Periodontol 1981;8:57-72.
17. Claffey N, Loos B, Gantes B, Martin M, Heins P, Egelberg J. The relative effects of therapy and periodontal disease on loss of probing attachment after root debridement. J Clin Periodontol 1988;15:163-9.
18. Moritz A, Schoop U, Goharkhay K, Schauer P, Doertbudak O, Wernisch J, *et al*. Treatment of periodontal pockets with a diode laser. Lasers Surg Med 1998;22:302-11.
19. Balli U, Aydogdu A, Dede FO, Turer CC, Guven B. Gingival crevicular fluid levels of sclerostin, osteoprotegerin, and receptor activator of nuclear factor-κB ligand in periodontitis. J Periodontol 2015;86:1396-404.
20. Baron R, Kneissel M. WNT signaling in bone homeostasis and disease: From human mutations to treatments. Nat Med 2013;19:179-92.
21. Sufia SS, Parthasarathy H, Ponnaiyan D, Tadepalli A. Effects of low level laser therapy (LLLT) biostimulation on GCF levels of sclerostin in surgical management of intrabony defects. Int J Sci Res 2018;7:1-5.
