# Supplementary material for: Molecular Regulators for the Wnt/β-Catenin Signalling Pathway for Bone Remodelling in Periodontitis: A Scoping Review
Source: Int J Mol Sci. 2026 Oct 14;27(20):9117. doi: 10.3390/ijms27209117 (PMC13705578; doi:10.3390/ijms27209117)
Supplement: Supplementary file 1 [file ijms-27-09117-s001.zip › Supplementary Data/Papers/in vivo/Lithium carbonate accelerates the healing of apical periodontitis.pdf]

scientific reports

Check for updates

OPEN

Lithium carbonate accelerates the healing of apical periodontitis

Takumi Kagioka[1], Shousaku Itoh[1]✉, Mai Thi Hue[1], Makoto Abe[2] & Mikako Hayashi[1]

Apical periodontitis is a disease caused by bacterial invasions through the root canals. Our previous study reported that lithium chloride (LiCl) had a healing effect on apical periodontitis. The aim of this report is to investigate the healing properties and mechanism of lithium ion (Li^+) for apical periodontitis using rat root canal treatment model. 10-week-old male Wistar rat's mandibular first molars with experimentally induced apical periodontitis underwent root canal treatment and were applied lithium carbonate (Li_2CO_3) containing intracanal medicament. Base material of the medicament was used as a control. Subject teeth were scanned by micro-CT every week and the periapical lesion volume was evaluated. The lesion volume of Li_2CO_3 group was significantly smaller than that of the control group. Histological analysis showed that in Li_2CO_3 group, M2 macrophages and regulatory T cells were induced in the periapical lesion. In situ hybridization experiments revealed a greater expression of *Col1a1* in Li_2CO_3 group compared with the control group. At 24 h after application of intracanal medicament, Axin2-positive cells were distributed in Li_2CO_3 group. In conclusion, Li_2CO_3 stimulates Wnt/β-catenin signaling pathway and accelerate the healing process of apical periodontitis, modulating the immune system and the bone metabolism.

Apical periodontitis is generally caused by the host's immune response to bacterial invasions through the root canals, resulting in the subsequent alveolar bone resorption by osteoclasts via immune cells' production of inflammatory cytokines[1–4]. In the case of apical periodontitis, the disrupted bone homeostasis causes alveolar bone resorption, resulting in the formation of periapical lesion which is observed as a radiolucent area around the root apex. Root canal treatment is intended to mechanically remove infected dentin and chemically reduce the number of bacteria. One of the chemical methods to remove bacterial is the usage of intracanal medicaments with bactericidal properties. Calcium hydroxide, which has antibacterial properties due to its high pH, is currently used as a common intracanal medicament in root canal treatment[5,6]. However, the application of calcium hydroxide, which is primarily an antibacterial agent, alone may cause prolonged healing of periapical lesions and, in some cases, lesions may not heal. Currently, the success rate of root canal treatment, especially retreatment, varies from one case to another; for example, the success rate for retreatment of teeth with periapical lesions is reported to be 65.7–80%[7–9]. Since we speculated that there might be a new approach to heal apical periodontitis, we tried to establish a new intracanal medicament.

In recent years, many disease-related genes have been identified in various multifactorial diseases by single nucleotide polymorphisms (SNPs). Since some cases were observed in which root canal cleaning and filling conditions were inadequate but no periapical lesions formed, it was speculated that there may be individual differences in the development of apical periodontitis. Our recent report demonstrated that the A1330V variant of LDL Receptor Related Protein 5 (LRP5), which was one of the co-receptors of Wnt proteins in the canonical Wnt pathway, was associated with apical periodontitis[10]. Previous reports have shown that the Wnt/β-catenin signaling pathway is involved in somatic axis formation during ontogeny and in the development of various diseases[11–13]. This pathway also has important functions in maintaining bone homeostasis[14–16]. Wnt protein inhibits glycogen synthase kinase-3β (GSK-3β)-mediated phosphorylation of β-catenin by binding to its frizzled receptor and co-receptor LRP 5/6. Stabilized β-catenin is accumulated in the cytoplasm. Accumulated β-catenin is transferred into the nucleus where it associates with the transcription factor LEF-1/TCF. The complex of β-catenin and LEF-1/TCF activates various target genes. Previous reports have shown that lithium chloride (LiCl) can stimulate the Wnt/β-catenin signaling pathway and promotes mineralization[17]. To determine the details of the role of this pathway in the development of apical periodontitis, we performed the root canal treatment on mice with induced apical periodontitis. The results of our in vivo experiments demonstrated that the application of LiCl into the mice's root canals accelerated the healing of apical periodontitis[10]. Thus, these results imply that lithium ion (Li^+) diffused from LiCl has the healing ability for apical periodontitis.

[1]Department of Restorative Dentistry and Endodontology, Osaka University Graduate School of Dentistry, 1-8 Yamadaoka, Suita, Osaka 565-0871, Japan. [2]Department of Oral Anatomy and Developmental Biology, Osaka University Graduate School of Dentistry, Osaka, Japan. ✉email: itoh.shousaku.dent@osaka-u.ac.jp

Moreover, the clinical use of LiCl was questionable in terms of its safety. In this report, we applied lithium carbonate (Li_2CO_3) into root canals instead of LiCl aiming to confirm the safety of Li^+ in clinical application and the healing ability of Li^+ for apical periodontitis. This is because Li_2CO_3 has already been used as a primary therapeutic agent for bipolar disorder. Furthermore, rats were used in this study to determine if the healing ability of Li^+ can be also observed in another animal. Because of the larger size of mandibular first molar of rats than that of mice, rubber dam could be used for root canal treatment, enabling treatment sterility. Alternatively, another objective of our study was to analyze the mechanism of the healing ability of Li^+ because the mechanism remained unclear in our previous study. To elucidate this mechanism, histological analysis was performed on rat mandibular tissue targeting immune cells, osteoblast, and the Wnt/β-catenin signaling pathway.

Results

Verify the safety during application of Li_2CO_3 into root canal. We applied a 12% Li_2CO_3 paste into the root canals to verify the safety for periapical tissue. The experimental procedure is shown in Fig. 1a. The hematoxylin and eosin (H&E) staining images showed that there was no difference in the periapical tissues between the control group and the 12% Li_2CO_3 group (Fig. 1b). In detail, there was no inflammatory cell infiltration around the apical foramen and the area that came into contact with the intracanal medicament in both the control and 12% Li_2CO_3 groups. In addition, there was no pathological alveolar bone and root resorption in these two groups. Further, to evaluate the systemic effects of the application of 12% Li_2CO_3 paste into root canals, we monitored the blood concentration of Li^+ for 72 h. The intraperitoneal group showed the transient increase in the blood concentration of Li^+ up to around 2 mM at 1 h after the administration (Fig. 1c). After that, the blood concentration of Li^+ was gradually decreased until 72 h. On the other hand, in cases of the application of 12% Li_2CO_3 into the root canals, there was no increase in the blood concentration of Li^+ throughout the observation period. These results demonstrated that applied Li^+ did not diffuse into the blood (Fig. 1c); therefore, indicating that the Li_2CO_3 was safe to apply into the root canal.

Li_2CO_3 reduced the volume of periapical lesions. To evaluate the applicability of Li_2CO_3 for the treatment of apical periodontitis, we applied a 12% Li_2CO_3 paste into root canals using a rat root canal treatment model. Figure 2a shows the experimental procedure. The results of H&E staining at 28 d after intracanal medication showed that the size of periapical lesions of the 12% Li_2CO_3 group was much smaller than that of the control group (Fig. 2b,c). In the 12% Li_2CO_3 group, there was hardly any infiltration of inflammatory cells. However, in the control group, many inflammatory cells were observed inside the periapical lesion. Additionally, some alveolar bone areas in the 12% Li_2CO_3 group appeared to have undergone healing of the lesions with bone tissue.

Next, we analyzed periapical lesion volumes using micro-CT. Throughout the study, we were able to check whether the intracanal medicament reached the apex of the root canal because of the X-ray contrast given to the base material of the control and 12% Li_2CO_3 paste (Fig. 2b,c). Periapical lesion volumes were not significantly different between the control and the 12% Li_2CO_3 groups from 0 ($p=0.932$) to 7 d ($p=0.075$). In contrast, the periapical lesion volume of the 12% Li_2CO_3 group was significantly smaller than that of the control group (Fig. 2d) on 14 d ($p=0.022$), 21 d ($p=0.005$) and 28 d ($p=0.009$).

Li_2CO_3 has the healing ability for apical periodontitis through the stimulation of Wnt/β-catenin signaling pathway. To elucidate the mechanism of the ameliorative effect of Li_2CO_3 on apical periodontitis, histological experiments were performed on periapical tissues. Though many CD86-positive cells were observed in the control group at 7, 14, and 21 d, there were very few CD86-positive cells in the 12% Li_2CO_3 group at the same time points (Fig. 3). At 28 d, there were few CD86-positive cells in both the control and 12% Li_2CO_3 groups. In contrast, many CD68/CD206-double positive cells were detected in the 12% Li_2CO_3 group compared with the control group from 0 to 28 d (Fig. 4). Furthermore, many Foxp3-positive cells were detected in the 12% Li_2CO_3 group at 7, 14, and 21 d, while there were very few Foxp3-positive cells in the control group at those time points (Fig. 5a–c). At 28 d, Foxp3-positive cells were observed in both groups (Fig. 5d). In situ hybridization experiments revealed a greater expression of *Col1a1* in the 12% Li_2CO_3 group compared with the control group from 7 to 28 d (Fig. 6). At 24 h after application of intracanal medicament, Axin2-positive cells were distributed in the 12% Li_2CO_3 group, but not in the control group (Fig. 7).

Discussion

Our recent study demonstrated that a SNP in LRP5 was associated with the development of apical periodontitis[10]. According to the results of the SNP analysis, we hypothesized that the development of apical periodontitis might be related with the Wnt/β-catenin signaling pathway. To clarify the role of this pathway in the development of apical periodontitis, we practiced root canal treatment on mice. In this previous experiment, the application of LiCl, which was reported to regulate this signaling pathway, into the murine root canals promoted the healing of apical periodontitis. This result implied that Li^+ regulates apical periodontitis development and healing. However, the clinical practices of root canal treatments on mice were difficult because of the teeth size. Even when the root canal treatment was successfully practiced, subject teeth were often broken during follow-up due to their fragility. In the current study, we applied Li_2CO_3 that released Li^+ to confirm the role of Li^+ in the regulation of apical periodontitis development. Considering future clinical applications for humans, we used Li_2CO_3, because Li_2CO_3 has been already used as a primary therapeutic agent for bipolar disorder for a long time[18]. However, because a high concentration of Li^+ induces side effects, such as lithium toxicity, bradycardia, and renal symptoms, patients who take Li_2CO_3 need Therapeutic Drug Monitoring of their Li^+ blood concentrations. To ensure the safety of Li_2CO_3 application into root canals, we continually monitored the blood Li^+ concentrations of the rat experimental model. As seen in previous reports[19–21], the systemic administration group showed transient

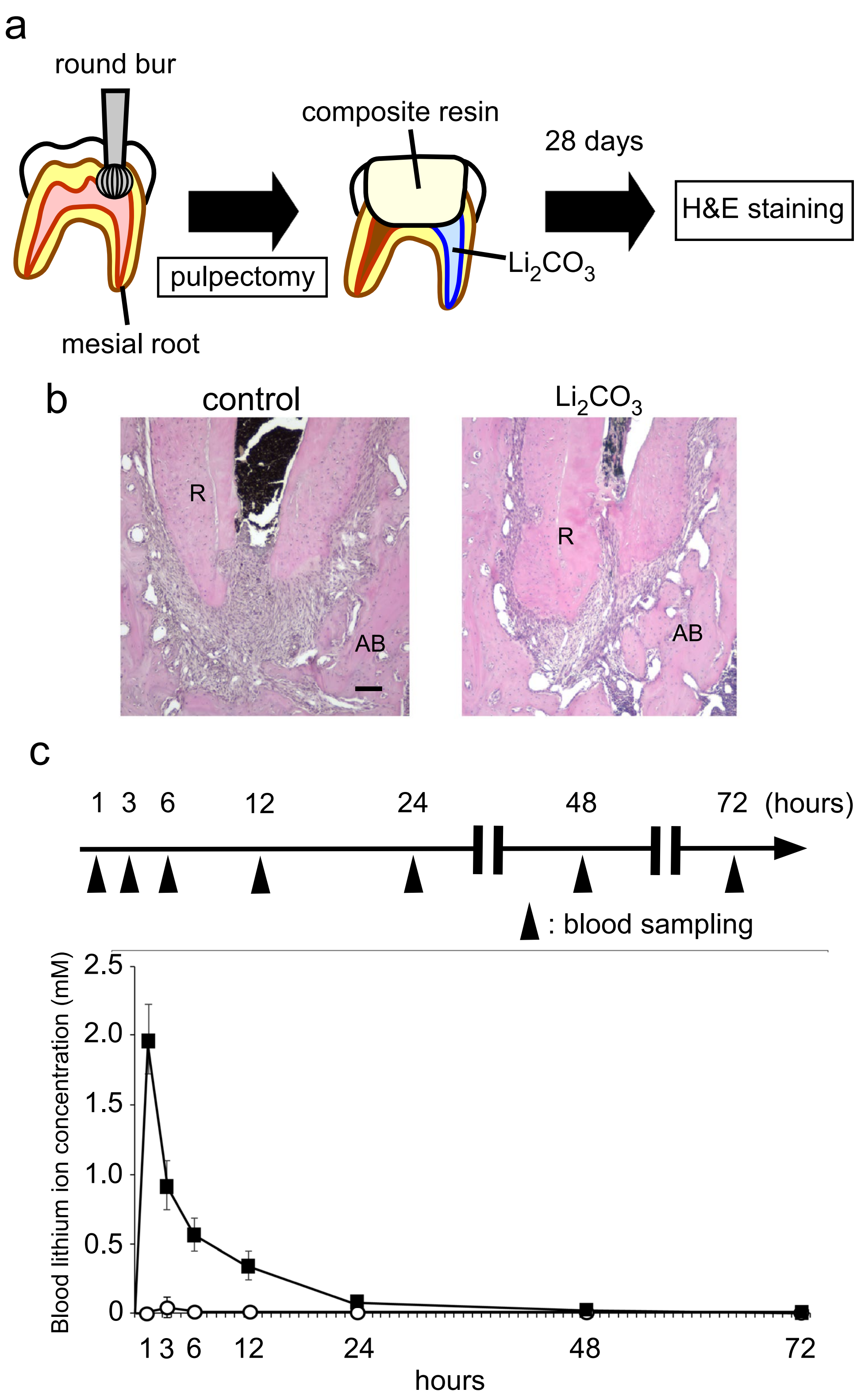

Figure 1. Li_2CO_3 had no harmful effects on periapical tissue. (**a**) Rat's mandibular first molar underwent pulpectomy and intracanal medicament was applied in the mesial root canal (n = 4). (**b**) At 28 d, H&E staining was carried out on the control and the 12% Li_2CO_3 groups. AB: Alveolar bone, R: Root. Scale bars = 500 µm. (**c**) The root canal treatment group underwent application of 12% Li_2CO_3 into the root canals. The intraperitoneal group underwent intraperitoneal administration of Li_2CO_3. Peripheral blood was collected at 1, 3, 6, 12, 24, 48, and 72 h, and Li^+ concentration of the samples was then determined. The average concentrations of Li^+ in the root canal group: white circle (n = 4) and the intraperitoneal group: black square (n = 4) are shown. Error bars indicate SD.

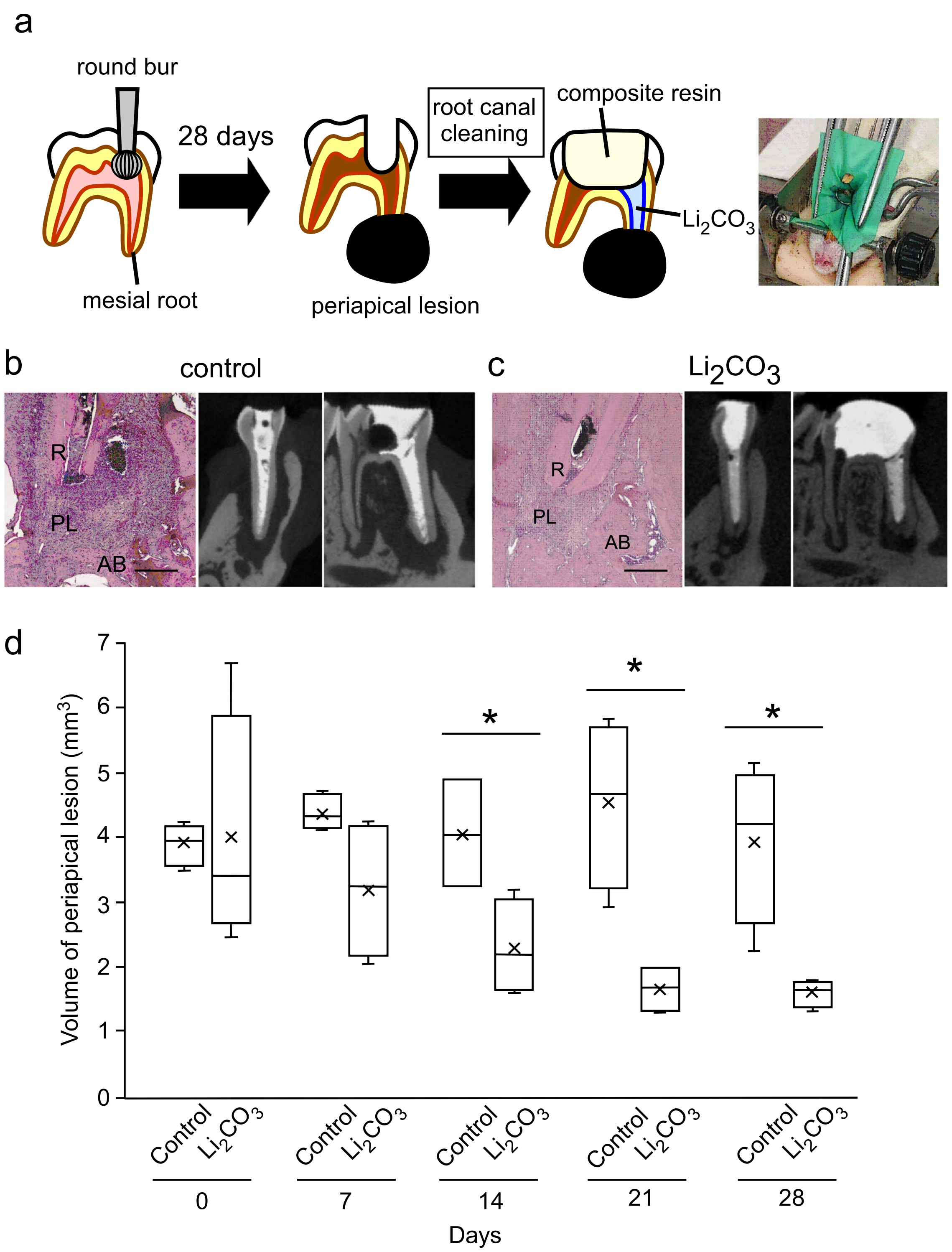

Figure 2. The 12% Li_2CO_3 group revealed the reduction of periapical lesion volume. (**a**) The experimental apical periodontitis was induced on rat's mandibular first molar. Root canal treatment was performed and intracanal medicament was applied in the mesial root canal. (**b**, **c**) At 28 d after root canal treatment, the control and the 12% Li_2CO_3 groups were subjected to H&E staining. AB: Alveolar bone, PL: Periapical Lesion, R: Root. Scale bars = 500 μm. Representative images for the micro-CT at 28 days are shown. (**d**) Periapical lesion volumes of the control group (n = 4) and the 12% Li_2CO_3 group (n = 4) were quantified. Student's *t*-test, *: $p < 0.05$.

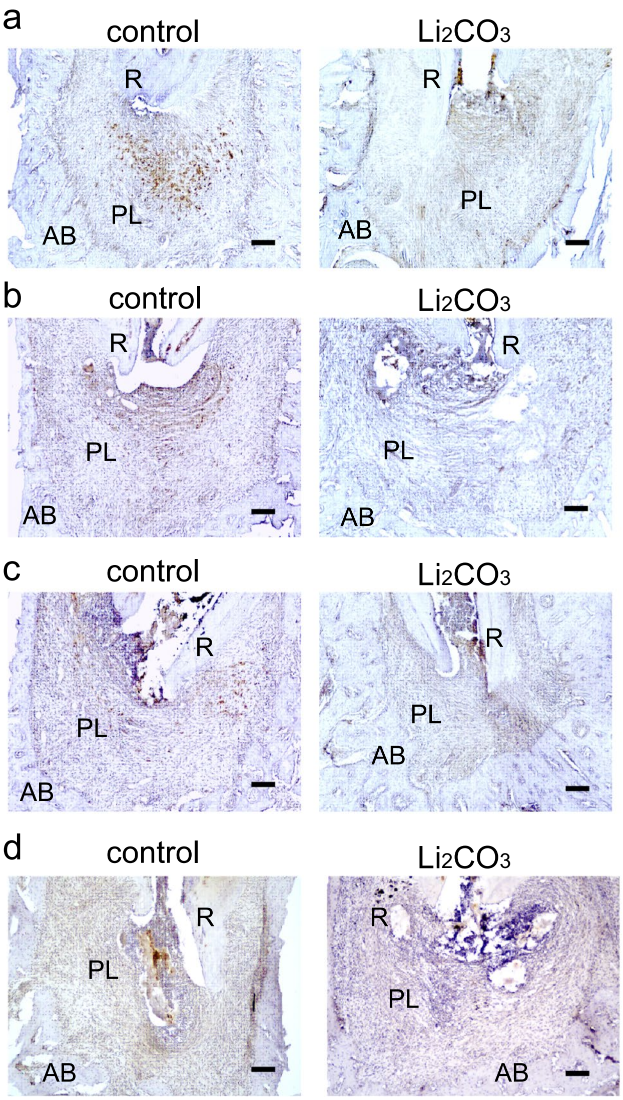

Figure 3. CD86-positive cells were detected in the control group. Mandibular tissues of the control group and the 12% Li_2CO_3 group were stained with an anti-CD86 antibody. At (**a**) 7, (**b**) 14, (**c**) 21, and (**d**) 28 d. AB: Alveolar bone, PL: Periapical Lesion, R: Root. Scale bars = 100 µm.

increases in Li^+ concentration in the blood (Fig. 1c). Comparatively, there was no increase in Li^+ concentration in the blood (Fig. 1c). These results implied that patients who were administered Li_2CO_3 into their root canals would not require Therapeutic Drug Monitoring of blood Li^+ levels.

To evaluate the healing ability of Li_2CO_3 for apical periodontitis, we practiced a recently developed rat root canal treatment model[22–24]. From H&E staining experiments, the size of the periapical lesion of the 12% Li_2CO_3 group at 28 d appeared smaller than that of the control group (Fig. 2b,c). To quantify the periapical lesion volume, the volume of radiolucent area around the root apex was analyzed using micro-CT. The periapical lesion volume in the 12% Li_2CO_3 group was significantly smaller than that in the control group (Fig. 2d). Since the intracanal medicament applied in the control group contained only the base material without medicinal properties, healing of the periapical lesion was dependent on the rats' inherent healing ability due to the removal of bacteria in the root canal. On the other hand, in the 12% Li_2CO_3 group, in addition to the removal of bacteria, the Li^+ released from the intracanal medicament could diminish the periapical lesion volume compared to the control group.

Next, we analyzed the mechanism of action of Li_2CO_3 for improving apical periodontitis with histopathological procedures. The healing of inflammatory diseases, including apical periodontitis, involves anti-inflammatory effects that occur at the inflammation site, such as the polarization of M1 to M2 macrophages and the induction of regulatory T cells[25,26]. Furthermore, previous reports have shown that the Wnt/β-catenin pathway is associated with the orchestration of anti-inflammatory effects[27–30]. At the early stage of inflammation, M1 macrophages are predominant and are induced by LPS and inflammatory cytokines, such as TNF-α and IFN-γ. M1 macrophages secrete pro-inflammatory cytokines and regulate the differentiation of Th1 and Th17 cells[25]. Whereas M1 macrophages are predominant in the early stage of inflammation, M2 macrophages increase in the middle to late

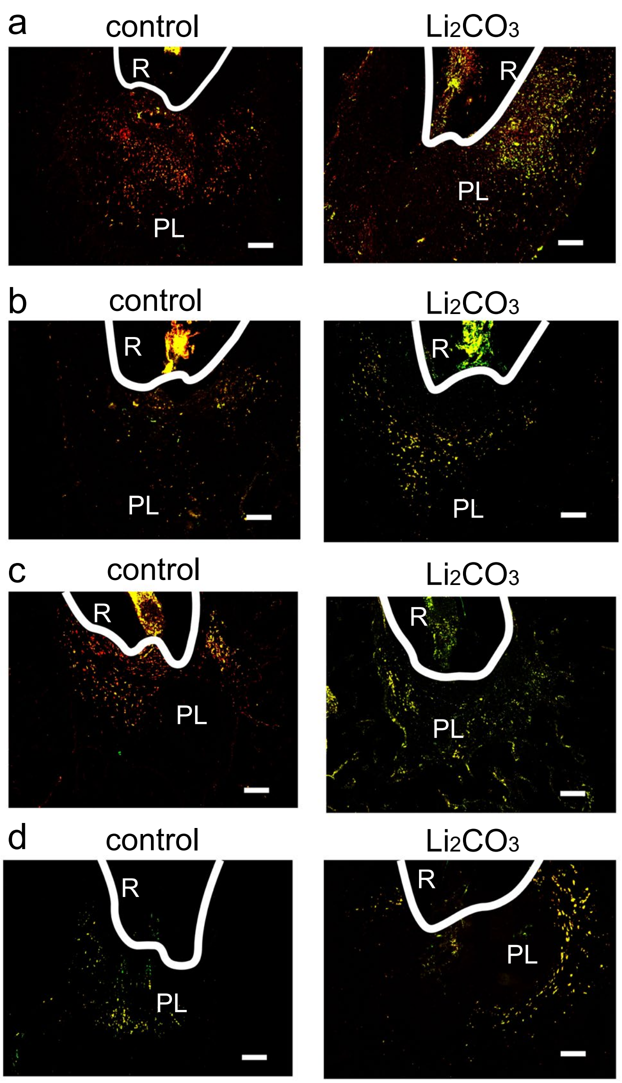

Figure 4. Many CD68/CD206-double positive cells were detected in the 12% Li_2CO_3 group. mandibular tissues of the control group and the Li_2CO_3 group were stained with an anti-CD68 antibody (red), anti-CD206 antibody (green), and DAPI (blue). At (**a**) 7, (**b**) 14, (**c**) 21, and (**d**) 28 d. PL: Periapical Lesion, R: Root. Scale bars = 100 µm.

stages of inflammation and are induced by IL-4, IL-10, and IL-13. They secrete anti-inflammatory cytokines and regulate the differentiation of Th2 and regulatory T cells[31,32]. Regulatory T cells have the capacity to downregulate all T cell-mediated immune responses[33–35]. Our histological data showed that there were many CD86-positive cells from 7 to 21 d in the control group and very few at 28 d (Fig. 3). CD68/CD206-double positive cells were observed from 28 d in the control group (Fig. 4). These results implied that the control group was in a pro-inflammatory state from 7 to 21 d and in an anti-inflammatory or healing state after 28 d. In contrast, there were very few CD86-positive cells in the 12% Li_2CO_3 group at 7 d (Fig. 3). CD68/CD206-double positive cells were observed from 7 to 28 d in the Li_2CO_3 application group (Fig. 4). These results showed that Li_2CO_3 induced polarization from M1 to M2 macrophages, and the Li_2CO_3 group was already in an anti-inflammatory or healing state at 7 d. Furthermore, though regulatory T cells were observed only after 28 d in the control group, they were already observed after 7 d in the 12% Li_2CO_3 group (Fig. 5). Regulatory T cells induced by Li_2CO_3 might downregulate immune responses in periapical lesions. Previous studies have reported that regulatory T cells secrete anti-inflammatory cytokines such as IL-10, IL-35 and TGF-β, and transfer the polarization from M1 to M2 macrophages[36,37]. Thus, regulatory T cells induced by Li_2CO_3 may suppress M1 macrophages' differentiation and enhance M2 macrophages' differentiation at the healing stages of apical periodontitis. Li_2CO_3 application was considered to activate the Wnt/β-catenin signaling pathway. This activation then induced the suppression of M1 macrophages, and the induction of M2 macrophages and regulatory T cells[15,38].

We employed in situ hybridization for *Col1a1* to evaluate osteoblast differentiation in the periapical lesion. In the control group, some level of *Col1a1* expression on the alveolar bone surface was observed throughout the entire experiment (Fig. 6). In contrast, in the 12% Li_2CO_3 group, *Col1a1* expression was higher than in the

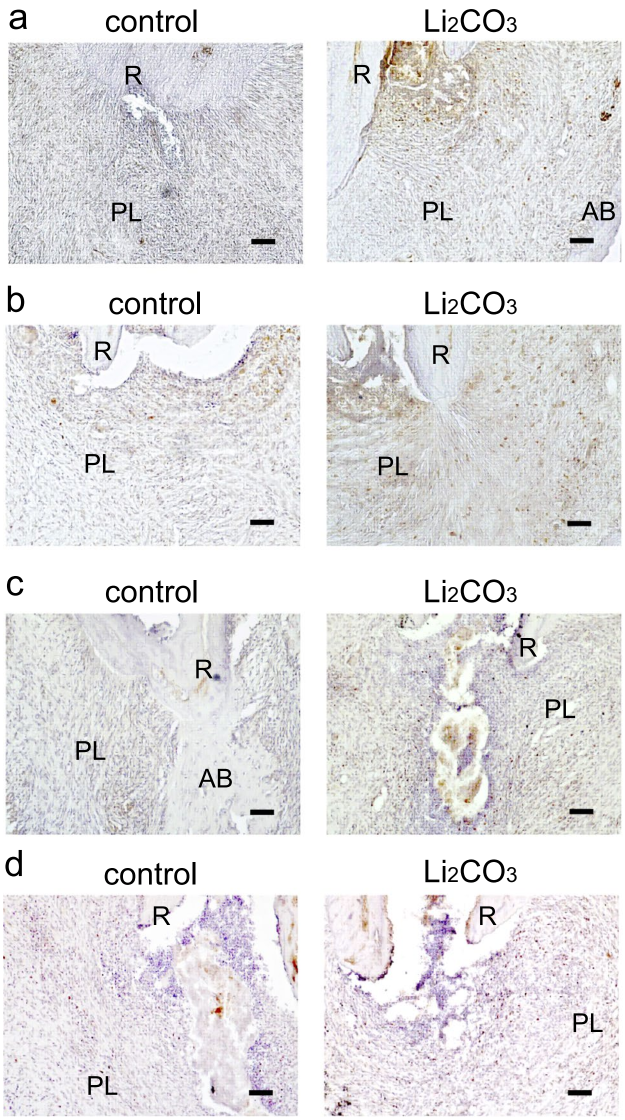

Figure 5. Foxp3-positive cells were detected in the 12% Li_2CO_3 group in the early stages of treatment. Periapical lesion tissues of the control group and the Li_2CO_3 group were stained with an anti-Foxp3 antibody. At (**a**) 7, (**b**) 14, (**c**) 21, and (**d**) 28 d. AB: Alveolar bone, PL: Periapical Lesion, R: Root. Scale bars = 100 µm.

control group throughout the experiment. Since *Col1a1* is known as a marker gene of osteoblast differentiation, a strong expression of *Col1a1* was indicative of accelerated bone healing in the periapical lesions of the 12% Li_2CO_3 group. According to the above results, regulatory T cells induced by Li_2CO_3 might support alveolar bone healing[39]. Finally, we confirm whether Li_2CO_3 activates the Wnt/β-catenin signaling pathway.

Finally, we performed the immunohistochemical staining for Axin2 to investigate the behavior of the canonical Wnt/β-catenin signaling pathway. Axin2 is well known as a target-gene product of canonical Wnt signaling. At 24 h after 12% Li_2CO_3 application into the root canals, sections were stained with an anti-Axin2 antibody, and many Axin2-positive cells were distributed in the periapical lesion of 12% Li_2CO_3 group (Fig. 7). This result demonstrated that Li_2CO_3 application induces the stimulation of the Wnt/β-catenin signaling pathway.

In conclusion, the role of Li_2CO_3 and its mechanism in the promotion of healing responses in apical periodontitis were determined. Li_2CO_3 application first activates the Wnt/β-catenin signaling pathway. This activation then induces Axin2 to regulate immune responses, such as the suppression of M1 macrophages, and the induction of M2 macrophages and regulatory T cells. Li_2CO_3 medication may change periapical lesions' inflammatory states to healing states at early stages of inflammation. Moreover, regulatory T cells induced by Li_2CO_3 may support alveolar bone healing through osteoblast differentiation and reduce the volume of periapical lesions. These results propose that Li_2CO_3 could be a bioactive medicament in root canal treatments.

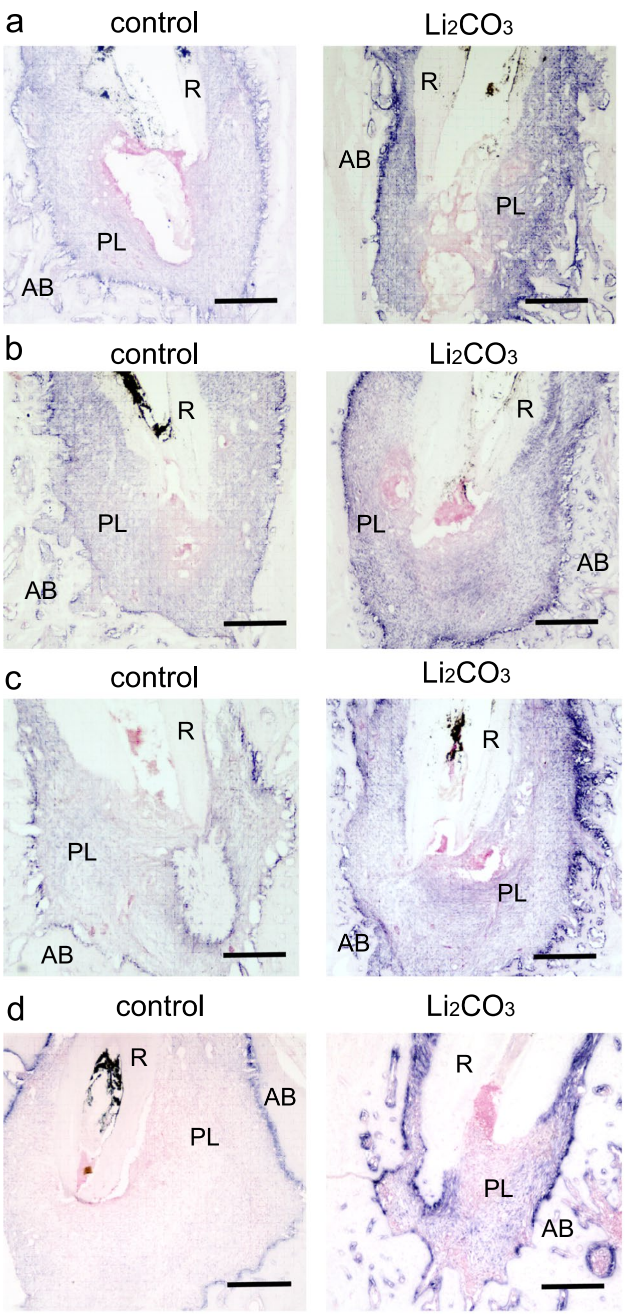

Figure 6. The 12% Li_2CO_3 group showed high expression levels of *Col1a1*. Mandibular tissues of the control group and the Li_2CO_3 group were subjected to in situ hybridization for *Col1a1*. At (**a**) 7, (**b**) 14, (**c**) 21, and (**d**) 28 d. AB: Alveolar bone, PL: Periapical Lesion, R: Root. Scale bars = 500 μm.

Methods

Ethics statement. This study was approved by the Research Ethics Committee of Osaka University Graduate School of Dentistry. All experiments were practiced according to the committee's guidelines related to animal care (AD-26-011-0) and were compliant with the guidelines of ARRIVE guidelines (http://www.nc3rs.org.uk/page.asp?id=1357).

Root canal treatment model of rat. Male Wistar rats (10 weeks old) were intraperitoneally anesthetized using Domitor (0.3 mg/kg: Nippon Zenyaku Kogyo Co., Fukushima, Japan), Dolmicam (4 mg/kg: Astellas Pharma Inc., Tokyo, Japan), and Betorphale (5 mg/kg: Meiji Seika Pharma, Tokyo, Japan). Experimental periapical lesion formation was performed following previous reports[40,41]. To induce apical periodontitis, the pulp chambers of the mandibular first molars were opened by a #1/2 round bur equipped with an electric engine (VIVA MATE G 5: NSK, Tochigi, Japan). Next, the root canals were penetrated with a #08 K-file (Dentsply

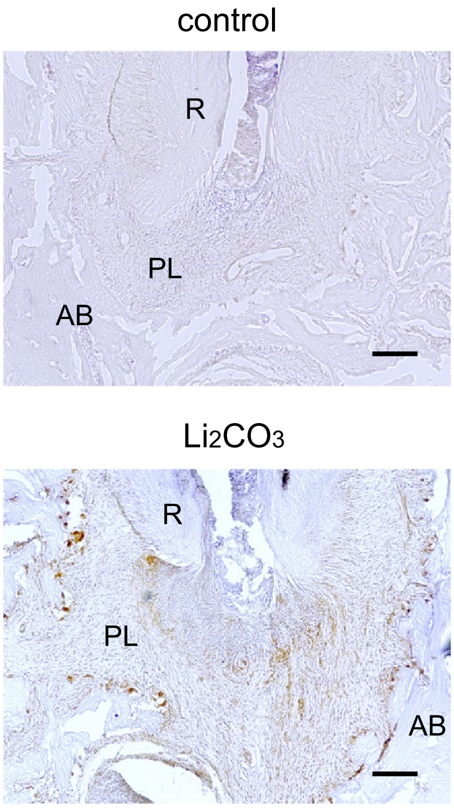

Figure 7. Li_2CO_3 application stimulated the canonical Wnt/β-catenin signaling pathway. Mandibular tissues of the control group and the 12% Li_2CO_3 group were stained with an anti-Axin2 antibody. AB: Alveolar bone, PL: Periapical Lesion, R: Root. Scale bars = 100 µm.

Maillefer) under the operating microscope (Stemi DV4 SPOT: Carl Zeiss, Oberkochen, Germany) and exposed to the oral cavity. At 28 d after pulp exposure, the cleaning of root canals was practiced as described below. The tooth was isolated using a custom-made rubber dam clamp (YDM, Tokyo, Japan) and a rubber dam sheet (Heraeus Kulzer, South Bend, USA). Necrotic coronal pulp and infected dentin were taken away using a #1/2 round bur. Infected dentin in the pulpal floor was taken away with a microexcavator (OK Micro-exca: Seto, Ibaraki, Japan) to avoid perforation. Root canal enlargement was performed to the level of 0.5 as indicated by an electric root canal meter (Root ZX: J Morita, Tokyo, Japan) using K-files (Dentsply Maillefer) up to a #20 file. Root canals were irrigated with 2.5% sodium hypochlorite (Neo Dental Chemical Products, Tokyo, Japan) using 30-gauge needles (NaviTip, Ultradent Products, South Jordan, UT). After root canal enlargement, the root canal was dried with a sterile paper point. Intracanal medicaments, which was prepared as paste filled in syringe, were applied into the mesial root canals under the operating microscope. After treatment with a bonding system (CLEARFIL Universal Bond Quick: Kuraray Noritake Dental, Tokyo, Japan), the pulp chamber was filled with the flowable composite resin (MI FLOW: GC, Tokyo, Japan).

Evaluation of Li_2CO_3 medication safety. To verify the safety of Li_2CO_3 medicament, we modified the rat root canal treatment model described above. Rats without induced apical periodontitis underwent pulpectomy (n = 4). Pulp tissue in coronal area was taken away using a #1/2 round bur. Root canal enlargement was practiced in the same procedure as described above. After root canal irrigation and drying, 12% Li_2CO_3 was applied into the mesial root canal. 12% was the maximum concentration of Li_2CO_3 that can be contained while maintaining adequate fluidity of the intracanal medicament. The control group were filled with base material (barium sulfate, aluminum oxide, titanium oxide, purified water) of the medicament. Finally, the coronal cavity was capped with the flowable composite resin. At 28 d after medication, rats were sacrificed for the histological analysis. At least two randomly selected samples from each group were used.

Further, to monitor the blood concentration of Li^+, rats were classified into two groups. Rats in the first group underwent application of 12% Li_2CO_3 into their mesial root canals (n = 4). The rats in the second group underwent an intraperitoneal administration of Li_2CO_3 (74 mg/kg), which was dissolved in saline (n = 4). Peripheral blood was collected from the subclavian vein at 1, 3, 6, 12, 24, 48, and 72 h after intracanal medication, and then centrifuged at 1000 × g for 20 min to collect the serum. Collected serum was stored at – 80 °C until used in experiments. The concentration of Li^+ in the serum was measured using the quantification reagent (LI01M; Metallogenics, Chiba, Japan) and the microplate reader (Wallac 1420 ARVO MX: PerkinElmer, Waltham, MA, USA).

Micro-CT analysis. To measure the periapical lesion volume, a micro-CT (R_mCT 2: Science Mechatronics, Tokyo, Japan) was used to scan the area around the mandibular first molar. Rats were sacrificed and scanned every week. The imaging conditions were adjusted as follows: 160 μA tube current, 90 kV tube voltage, and 5 μm slice width. The obtained images were analyzed by Simple Viewer software (Science Mechatronics). According to the methods described Yoneda et al.[24] and Kalatizis-Sousa et al.[42], the volume of periapical lesion was defined as the volume of radiolucent area around the root apex and calculated using the bone morphometrics software (TRI 3D-BON: RATOC, Osaka, Japan). Then, the lesion volume was compared between experimental groups (n = 4 each) as previously reported[22].

Sample preparation for the histological analysis. After the rats were subjected to the above experiments, they were perfused with 4% paraformaldehyde solution. The mandibles of the rats were collected and immersed in 4% paraformaldehyde solution and fixed for 24–48 h, followed by decalcification with Kalkitox (Fujifilm, Tokyo, Japan) for 14 d with gentle agitation. For H&E staining, the tissues were dehydrated with ascending ethanol series, penetrated with xylene, and finally embedded in paraffin. Samples were then sliced to 7 μm thickness. For immunostaining and in situ hybridization, decalcified mandibular tissues were embedded in O.C.T compound (Sakura Finetek, Tokyo, Japan), frozen at − 80 °C, and sliced to 14 μm thickness. At least two randomly selected samples from each group were used.

H&E staining. Paraffin sections were deparaffinized, rinsed with tap water, and stained with Mayer's hematoxylin solution (Muto, Osaka, Japan) for 5 min. The sections were then rinsed with tap water for 20 min and stained with eosin solution (Merck, Darmstadt, Germany) for 5 min. Finally, sections were dehydrated and decolorized with ethanol series, penetrated with xylene, and sealed with MOUNT-QUICK (Daido Sangyo, Saitama, Japan). The periapical lesion and the surrounding bone tissue were observed under the optical microscope (BZ-X810: KEYENCE, Osaka, Japan).

Immunohistochemistry and immunofluorescence. The frozen sections were rinsed with Tris-buffered saline (TBS) at room temperature for 10 min. After blocking with 10% normal goat serum (NGS) containing blocking solution (10% NGS/TBS) for 1 h, each antibody was reacted in the humid chamber at 4 °C overnight. The concentration of each antibody used in the experiments was 1:500 for CD86 (bs-1035R: Bioss, Boston, MA, USA), 1:1000 for Foxp3 (NB100-39002: Novus Biologicals, Centennial, CO, USA) and 1:500 for Axin2 (ab32197: Abcam, Cambridge, UK). After the reaction with these primary antibodies, the bound antibodies were visualized with a VECTASTAIN Elite ABC kit (VECTOR LABORATORIES, Burlingame, CA, USA) and Diaminobenzidine (DAB) (ImmPACT DAB: VECTOR LABORATORIES). The tissues were counter stained with Mayer's hematoxylin solution, and then dehydrated, penetrated, and sealed with MOUNT-QUICK.

For immunofluorescence analysis, the sections were sequentially reacted with the anti-CD68 antibody (1:500, ab125212: Abcam) and then reacted with the biotinylated secondary antibody. After the reactions, the sections were reacted with APC-streptavidin (405,207: BioLegend, San Diego, CA, USA) and the FITC-anti-CD206 antibody (1:200: bs-4747R-FITC, Bioss). Finally, the sections were stained with DAPI (Sigma) and observed under a fluorescence microscope (BZ-X810: KEYENCE).

In situ hybridization. Frozen sections were rinsed with 10 mM PBS at room temperature for 10 min, fixed with 4% PFA for 30 min at 37 °C, and washed again with distilled water. Then, sections were treated with 0.2% HCl for 10 min, followed by the reaction with 1 μg/ml proteinase K (Takara Bio, Shiga, Japan) for 10 min at 37 °C. After rinsing with PBS, sections were washed with G-Wash (Genostaff, Tokyo, Japan). Hybridization was performed in a humid chamber at 50 °C overnight using digoxigenin-labeled RNA probes, *Col1a1* (NM_053304, nt3946-4887) diluted with G-Hybo (Genostaff). After the hybridization, the sections were washed with 50% formamide in G-Wash for at least 30 min at 50 °C and rinsed with TBST. Blocking was then performed with G-Block (Genostaff) for 15–30 min. After reaction with alkaline phosphatase (AP)-labeled anti-digoxigenin antibody (1:2000, Roche, Basel, Switzerland) at room temperature for 1 h, the sections were rinsed with TBST, followed by rinse with distilled water. The sections were reacted with BM Purple AP (Roche) as a substrate for 1 h at room temperature, after which they rinsed with PBS and were stained with Nuclear Fast Red solution as a counter staining. The sections were finally sealed with G-Mount (Genostaff). Periapical lesion and alveolar bone were observed under the optical microscope.

Statistical analyses. Sample size was calculated using PS software (Power and Sample Size; Vanderbilt University Medical Center). Power analysis was also performed. The Shapiro–Wilk test was used to confirm that the data were normally distributed. All data were shown as the mean ± standard deviation. In a series of experiments, statistical analysis was carried out using the Student's *t*-test; $p < 0.05$ was considered significant. IBM SPSS Statistics software (IBM, Chicago, IL, USA) was used for statistical analysis.

Data availability

All data relevant to this study are presented in full in this paper.

Received: 2 February 2023; Accepted: 5 May 2023
Published online: 16 May 2023

References

1. Graunaite, I., Lodiene, G. & Maciulskiene, V. Pathogenesis of apical periodontitis: a literature review. *J. Oral Maxillofac. Res.* **2**, e1. https://doi.org/10.5037/jomr.2011.2401 (2012).
2. Graves, D. T., Oates, T. & Garlet, G. P. Review of osteoimmunology and the host response in endodontic and periodontal lesions. *J. Oral Microbiol.* **3**, 5304. https://doi.org/10.3402/jom.v3i0.5304 (2011).
3. Stashenko, P., Teles, R. & D'Souza, R. Periapical inflammatory responses and their modulation. *Crit. Rev. Oral Biol. Med.* **9**, 498–521. https://doi.org/10.1177/10454411980090040701 (1998).
4. Marton, I. J. & Kiss, C. Protective and destructive immune reactions in apical periodontitis. *Oral Microbiol. Immunol.* **15**, 139–150. https://doi.org/10.1034/j.1399-302x.2000.150301.x (2000).
5. Estrela, C., Sydney, G. B., Bammann, L. L. & Felippe Júnior, O. Mechanism of action of calcium and hydroxyl ions of calcium hydroxide on tissue and bacteria. *Braz. Dent. J.* **6**, 85–90 (1995).
6. Siqueira, J. F. & Lopes, H. P. Mechanisms of antimicrobial activity of calcium hydroxide: A critical review. *Int. Endod. J.* **32**, 361–369. https://doi.org/10.1046/j.1365-2591.1999.00275.x (1999).
7. de Chevigny, C. *et al.* Treatment outcome in endodontics: The Toronto Study—Phases 3 and 4: Orthograde retreatment. *J. Endod.* **34**, 131–137. https://doi.org/10.1016/j.joen.2007.11.003 (2008).
8. Ng, Y. L., Mann, V. & Gulabivala, K. Outcome of secondary root canal treatment: A systematic review of the literature. *Int. Endod. J.* **41**, 1026–1046. https://doi.org/10.1111/j.1365-2591.2008.01484.x (2008).
9. Farzaneh, M., Abitbol, S. & Friedman, S. Treatment outcome in endodontics: The Toronto Study. Phases I and II: Orthograde retreatment. *J. Endod.* **30**, 627–633. https://doi.org/10.1097/01.don.0000129958.12388.82 (2004).
10. Naruse, H. *et al.* The Wnt/beta-catenin signaling pathway has a healing ability for periapical periodontitis. *Sci. Rep.* https://doi.org/10.1038/s41598-021-99231-x (2021).
11. Liu, F. & Millar, S. E. Wnt/beta-catenin signaling in oral tissue development and disease. *J. Dent. Res.* **89**, 318–330. https://doi.org/10.1177/0022034510363373 (2010).
12. Steinhart, Z. & Angers, S. Wnt signaling in development and tissue homeostasis. *Development* https://doi.org/10.1242/dev.146589 (2018).
13. Duchartre, Y., Kim, Y. M. & Kahn, M. The Wnt signaling pathway in cancer. *Crit. Rev. Oncol. Hematol.* **99**, 141–149. https://doi.org/10.1016/j.critrevonc.2015.12.005 (2016).
14. Komori, T. Signaling networks in RUNX2-dependent bone development. *J. Cell. Biochem.* **112**, 750–755. https://doi.org/10.1002/jcb.22994 (2011).
15. Baron, R. & Kneissel, M. WNT signaling in bone homeostasis and disease: From human mutations to treatments. *Nat. Med.* **19**, 179–192. https://doi.org/10.1038/nm.3074 (2013).
16. Duan, P. & Bonewald, L. F. The role of the wnt/β-catenin signaling pathway in formation and maintenance of bone and teeth. *Int. J. Biochem. Cell Biol.* **77**, 23–29. https://doi.org/10.1016/j.biocel.2016.05.015 (2016).
17. Han, P. P., Ivanovski, S., Crawford, R. & Xiao, Y. Activation of the canonical Wnt signaling pathway induces cementum regeneration. *J. Bone Miner. Res.* **30**, 1160–1174. https://doi.org/10.1002/jbmr.2445 (2015).
18. Licht, R. W. Lithium: Still a major option in the management of bipolar disorder. *CNS Neurosci. Ther.* **18**, 219–226. https://doi.org/10.1111/j.1755-5949.2011.00260.x (2012).
19. Kling, M. A., Manowitz, P. & Pollack, I. W. Rat-brain and serum lithium concentrations after acute injections of lithium-carbonate and orotate. *J. Pharm. Pharmacol.* **30**, 368–370. https://doi.org/10.1111/j.2042-7158.1978.tb13258.x (1978).
20. Hanak, A. S. *et al.* Study of blood and brain lithium pharmacokinetics in the rat according to three different modalities of poisoning. *Toxicol. Sci.* **143**, 185–195. https://doi.org/10.1093/toxsci/kfu224 (2015).
21. Kang, K. *et al.* Lithium pretreatment reduces brain injury after intracerebral hemorrhage in rats. *Neurol. Res.* **34**, 447–454. https://doi.org/10.1179/1743132812y.0000000015 (2012).
22. Goldman, E. *et al.* A mouse model for studying the development of apical periodontitis with age. *Cells* **10**, 671. https://doi.org/10.3390/cells10030671 (2021).
23. da Silva, R. A. B., Ferreira, P. D. F., De Rossi, A., Nelson, P. & Silva, L. A. B. Toll-like receptor 2 knockout mice showed increased periapical lesion size and osteoclast number. *J. Endod.* **38**, 803–813. https://doi.org/10.1016/j.joen.2012.03.017 (2012).
24. Yoneda, N. *et al.* Development of a root canal treatment model in the rat. *Sci. Rep.* **7**, 9. https://doi.org/10.1038/s41598-017-03628-6 (2017).
25. Lei, H., Schmidt-Bleek, K., Dienelt, A., Reinke, P. & Volk, H. D. Regulatory T cell-mediated anti-inflammatory effects promote successful tissue repair in both indirect and direct manners. *Front. pharmacol.* **6**, 184. https://doi.org/10.3389/fphar.2015.00184 (2015).
26. Mosser, D. M. & Edwards, J. P. Exploring the full spectrum of macrophage activation. *Nat. Rev. Immunol.* **8**, 958–969. https://doi.org/10.1038/nri2448 (2008).
27. Sha, H., Zhang, D. Z., Zhang, Y. F., Wen, Y. H. & Wang, Y. C. ATF3 promotes migration and M1/M2 polarization of macrophages by activating tenascin-C via Wnt/beta-catenin pathway. *Mol. Med. Rep.* **16**, 3641–3647. https://doi.org/10.3892/mmr.2017.6992 (2017).
28. Yuan, C. *et al.* Modulation of Wnt/beta-catenin signaling in IL-17A-mediated macrophage polarization of RAW264.7 cells. *Braz. J. Med. Biol. Res.* **53**, 10. https://doi.org/10.1590/1414-431x20209488 (2020).
29. Staal, F. J. T., Luis, T. C. & Tiemessen, M. M. WNT signalling in the immune system: WNT is spreading its wings. *Nat. Rev. Immunol.* **8**, 581–593. https://doi.org/10.1038/nri2360 (2008).
30. Ding, Y., Shen, S. Q., Lino, A. C., de Lafaille, M. A. C. & Lafaille, J. J. Beta-catenin stabilization extends regulatory T cell survival and induces anergy in nonregulatory T cells. *Nat. Med.* **14**, 162–169. https://doi.org/10.1038/nm1707 (2008).
31. Shapouri-Moghaddam, A. *et al.* Macrophage plasticity, polarization, and function in health and disease. *J. Cell. Physiol.* **233**, 6425–6440. https://doi.org/10.1002/jcp.26429 (2018).
32. Biswas, S. K. & Mantovani, A. Macrophage plasticity and interaction with lymphocyte subsets: Cancer as a paradigm. *Nat. Immunol.* **11**, 889–896. https://doi.org/10.1038/ni.1937 (2010).
33. Hori, S., Nomura, T. & Sakaguchi, S. Control of regulatory T cell development by the transcription factor Foxp3. *Science* **299**, 1057–1061. https://doi.org/10.1126/science.1079490 (2003).
34. Sakaguchi, S., Wing, K. & Miyara, M. Regulatory T cells—A brief history and perspective. *Eur. J. Immunol.* **37**, S116–S123. https://doi.org/10.1002/eji.200737593 (2007).
35. Wing, J. B., Tanaka, A. & Sakaguchi, S. Human FOXP3(+) regulatory T cell heterogeneity and function in autoimmunity and cancer. *Immunity* **50**, 302–316. https://doi.org/10.1016/j.immuni.2019.01.020 (2019).
36. Tan, J. L. *et al.* Amnion cell-mediated immune modulation following bleomycin challenge: Controlling the regulatory T cell response. *Stem Cell Res. Ther.* **6**, 12. https://doi.org/10.1186/scrt542 (2015).
37. Weirather, J. *et al.* Foxp3(+) CD4+ T cells improve healing after myocardial infarction by modulating monocyte/macrophage differentiation. *Circ. Res.* **115**, 55–67. https://doi.org/10.1161/circresaha.115.303895 (2014).
38. Nusse, R. & Clevers, H. Wnt/beta-catenin signaling, disease, and emerging therapeutic modalities. *Cell* **169**, 985–999. https://doi.org/10.1016/j.cell.2017.05.016 (2017).

39. Li, J. T., Tan, J., Martino, M. M. & Lui, K. O. Regulatory T-cells: Potential regulator of tissue repair and regeneration. *Front. Immunol.* https://doi.org/10.3389/fimmu.2018.00585 (2018).
40. Kawahara, T. *et al.* Effects of cyclosporin-A-induced immunosuppression on periapical lesions in rats. *J. Dent. Res.* **83**, 683–687. https://doi.org/10.1177/154405910408300905 (2004).
41. Kuremoto, K. *et al.* Promotion of endodontic lesions in rats by a novel extraradicular biofilm model using obturation materials. *Appl. Environ. Microbiol.* **80**, 3804–3810. https://doi.org/10.1128/aem.00421-14 (2014).
42. Kalatzis-Sousa, N. G., Spin-Neto, R., Wenzel, A., Tanomaru, M. & Faria, G. Use of micro-computed tomography for the assessment of periapical lesions in small rodents: a systematic review. *Int. Endod. J.* **50**, 352–366. https://doi.org/10.1111/iej.12633 (2017).

Acknowledgements
This study was supported by Grant-in-aid for Scientific Research (20K09952 and 21K21083) from the Japan Society for the Promotion of Science.

Author contributions
T.K., M.T.H., H.N., and S.I. conceived the experiments. T.K., M.T.H., S.I., and M.A. performed the experiments. T.K., M.T.H., S.I., and M.A. analyzed the data. T.K., S.I., and M.H. wrote the manuscript. All authors reviewed this manuscript.

Competing interests
The authors declare no competing interests.

Additional information
Correspondence and requests for materials should be addressed to S.I.

Reprints and permissions information is available at www.nature.com/reprints.

Publisher's note Springer Nature remains neutral with regard to jurisdictional claims in published maps and institutional affiliations.

Open Access This article is licensed under a Creative Commons Attribution 4.0 International License, which permits use, sharing, adaptation, distribution and reproduction in any medium or format, as long as you give appropriate credit to the original author(s) and the source, provide a link to the Creative Commons licence, and indicate if changes were made. The images or other third party material in this article are included in the article's Creative Commons licence, unless indicated otherwise in a credit line to the material. If material is not included in the article's Creative Commons licence and your intended use is not permitted by statutory regulation or exceeds the permitted use, you will need to obtain permission directly from the copyright holder. To view a copy of this licence, visit http://creativecommons.org/licenses/by/4.0/.

© The Author(s) 2023
