# Supplementary material for: Molecular Regulators for the Wnt/β-Catenin Signalling Pathway for Bone Remodelling in Periodontitis: A Scoping Review
Source: Int J Mol Sci. 2026 Oct 14;27(20):9117. doi: 10.3390/ijms27209117 (PMC13705578; doi:10.3390/ijms27209117)
Supplement: Supplementary file 1 [file ijms-27-09117-s001.zip › Supplementary Data/Search Strategy.pdf]

1. IDEA: ~ what is it that you want to search/study? Key points

Investigating the role of the Wnt/β-catenin signalling pathway in bone remodelling

How periodontitis disrupts Wnt/β-catenin activity in the context of bone metabolism

2. SCOPE:

clinical dentistry, biochemistry

3. OBJECTIVE:

To determine the role of the Wnt/β-catenin signalling pathway on bone remodelling in periodontitis rats

4. RESEARCH QUESTION:

MAIN QUESTION:
What is the effect of periodontitis on the Wnt/β-catenin signalling pathway in terms of bone remodelling?
Dah ada: wnt pathway in osteoporosis, harness wnt β-catenin in dentin regen,
Could be: bone remodelling in perio

SUB-QUESTIONS ~ if any (*must be relevant to and within the scope of the Main RQ*):

- How does the Wnt/β-catenin controls bone remodelling
- How does the Wnt/β-catenin affected by periodontal inflammation
- How is the Wnt/β-catenin activity regulated

**PICO**	***Main items***
**P** Population / Participant / Process	Wnt/β-catenin signalling pathway
**I** Intervention	Periodontitis
**C** Comparison	Uncontrolled signalling pathway
**O** Outcome measures	Bone remodelling activities

Inclusion & Exclusion criteria related to RQ and Objectives

**Inclusion**	***Exclusion***
Studies between 2007-2025	Retracted papers
Document type: Primary article	
English language	
Open access	

5. MAIN KEYWORDS:

**Main (from RQ or Objective)**	**definition**	**MeSH**	**Synonym / other names**
Wnt/β-catenin signalling system			Wnt/β-catenin pathway<br>Wnt pathway
Bone remodelling	*Bone matrix turnover*		Bone resorption<br>Bone formation<br>Calcium homeostasis<br>Osteoclastic activity<br>Osteoblastic activity<br>Bone metabolism
Periodontal inflammation			Periodontitis

6. Search Strategy

Database #1: web of science Date & Time: 11/2/2026

**Steps**	**Search string/strategy**	**No of hits/ search results**
1	**Wnt/b-catenin OR Wnt pathway OR Wnt/b-catenin signalling system** (Topic) and **2016-2025** (Year Published) and **Article** (Document Types) and **English** (Languages)<br><br>https://www.webofscience.com/wos/woscc/summary/deb91906-8cd1-4e38-8f8b-050f7d859a5c-01c7d8f47c/date-descending/1	24,119
2	**"bone remodelling" OR "bone metabolism" AND osteoblast OR osteoclast** (Topic) and **2016-2025** (Year Published) and **English** (Languages)<br><br>https://www.webofscience.com/wos/woscc/summary/bd82aa1a-db23-4cb7-8cbb-eedad25e1171-01c7d93014/date-descending/1	38,071
3	**Periodontal inflammation OR periodontitis (Topic)** (Topic) and **2016-2025** (Year Published) and **Article** (Document Types) and **English** (Languages)<br><br>https://www.webofscience.com/wos/woscc/summary/0ba5b0aa-560e-4c0b-82da-45d49ac1e32b-01c7d93d31/date-descending/1	18,774
4	1 AND 2	1,460
5	1 AND 3	85
6	1 AND 2 AND 3	32

Database #2: scopus Date & Time:1/2/2026

**Steps**	**Search string/strategy**	**No of hits/ search results**
1	*(TITLE-ABS-KEY (Wnt / b-catenin OR Wnt / beta-catenin) AND NOT TITLE-ABS-KEY (cancer OR neuro* OR gastrulation OR embryo* OR carcinoma OR muscle OR intestin*)) AND PUBYEAR > 2006 AND PUBYEAR < 2026 AND (LIMIT-TO (OA , "all")) AND (LIMIT-TO (*	4,449

	EXACTKEYWORD , "Signal Transduction") OR LIMIT-TO (EXACTKEYWORD , "Beta Catenin") OR LIMIT-TO (EXACTKEYWORD , "Wnt Protein") OR LIMIT-TO (EXACTKEYWORD , "Wnt Signaling Pathway") OR LIMIT-TO (EXACTKEYWORD , "Wnt Signaling") OR LIMIT-TO (EXACTKEYWORD , "Wnt Proteins") OR LIMIT-TO (EXACTKEYWORD , "Canonical Wnt Signaling") OR LIMIT-TO (EXACTKEYWORD , "Β-catenin") OR LIMIT-TO (EXACTKEYWORD , "Wnt/β-catenin") OR LIMIT-TO (EXACTKEYWORD , "Wnt3a Protein") OR LIMIT-TO (EXACTKEYWORD , "Wnt")) AND (LIMIT-TO (DOCTYPE , "ar")) AND (LIMIT-TO (LANGUAGE , "English"))	
2	TITLE-ABS-KEY ("bone remodelling" OR "bone metabolism") AND PUBYEAR > 2006 AND PUBYEAR < 2026 AND (LIMIT-TO (EXACTKEYWORD , "Bone Metabolism") OR LIMIT-TO (EXACTKEYWORD , "Osteoporosis") OR LIMIT-TO (EXACTKEYWORD , "Osteoblast") OR LIMIT-TO (EXACTKEYWORD , "Bone Remodeling") OR LIMIT-TO (EXACTKEYWORD , "Osteoclast") OR LIMIT-TO (EXACTKEYWORD , "Cell Differentiation") OR LIMIT-TO (EXACTKEYWORD , "Osteoclast Differentiation Factor") OR LIMIT-TO (EXACTKEYWORD , "Osteogenesis") OR LIMIT-TO (EXACTKEYWORD , "Osteoblasts") OR LIMIT-TO (EXACTKEYWORD , "Osteoclastogenesis") OR LIMIT-TO (EXACTKEYWORD , "Osteoprotegerin") OR LIMIT-TO (EXACTKEYWORD , "Osteoclasts") OR LIMIT-TO (EXACTKEYWORD , "Bone Resorption") OR LIMIT-TO (EXACTKEYWORD , "Osteopenia") OR LIMIT-TO (EXACTKEYWORD , "Homeostasis") OR LIMIT-TO (EXACTKEYWORD , "Bone Regeneration") OR LIMIT-TO (EXACTKEYWORD , "Bone Remodelling") OR LIMIT-TO (EXACTKEYWORD , "Mesenchymal Stem Cell") OR LIMIT-TO (EXACTKEYWORD , "Bone Tissue") OR LIMIT-TO (EXACTKEYWORD , "Osteocyte") OR LIMIT-TO (EXACTKEYWORD , "Osteoarthritis")) AND (LIMIT-TO (DOCTYPE , "ar")) AND (LIMIT-TO (LANGUAGE , "English")) AND (LIMIT-TO (OA , "all"))	8,267
3	TITLE-ABS-KEY (periodontal inflammation OR periodontitis OR inflammation) AND PUBYEAR > 2006 AND PUBYEAR < 2026 AND (LIMIT-TO (EXACTKEYWORD , "Periodontitis") OR LIMIT-TO (EXACTKEYWORD , "Periodontal Disease") OR LIMIT-TO (EXACTKEYWORD , "Inflammation") OR LIMIT-TO (EXACTKEYWORD , "Periodontal Diseases") OR LIMIT-TO (EXACTKEYWORD , "Alveolar Bone Loss") OR LIMIT-TO (EXACTKEYWORD , "Periodontal Ligament") OR LIMIT-TO (EXACTKEYWORD , "Periodontium") OR LIMIT-TO (EXACTKEYWORD , "Periodontal Attachment Loss")) AND (LIMIT-TO (DOCTYPE , "ar")) AND (LIMIT-TO (LANGUAGE , "English")) AND (LIMIT-TO (OA , "all"))	11,676
4	1 AND 2	441
5	1 AND 2 AND 3	5

Database #3: Ovid medline Date & Time:1/2/2026

**Steps**	**Search string/strategy**	**No of hits/ search results**
1	Wnt beta-catenin or Wnt b-catenin).af. AND limit 5 to (english language and full text and yr="2007 - 2025" and journal article)	8372

2	("bone remodelling" or "bone metabolism").af AND limit 3 to (english language and full text and yr="2007 - 2025" and journal article)	6009
3	(Periodontal inflammation or periodontitis or inflammation).af. AND imit 5 to (english language and full text and yr="2007 - 2025" and journal article)	304088
4	1 AND 2 AND 3	12
